# Asymmetric synthesis of unnatural α-amino acids through photoredox-mediated C–O bond activation of aliphatic alcohols†

**DOI:** 10.1039/d4sc00403e

**Published:** 2024-04-17

**Authors:** Gregory R. Alvey, Elena V. Stepanova, Andrey Shatskiy, Josefin Lantz, Rachel Willemsen, Alix Munoz, Peter Dinér, Markus D. Kärkäs

**Affiliations:** a Department of Chemistry, KTH Royal Institute of Technology SE-100 44 Stockholm Sweden karkas@kth.se; b Chemical Technology, Materials Sciences, Metallurgy, Tomsk Polytechnic University Lenin Avenue 30 634050 Tomsk Russia

## Abstract

Unnatural α-amino acids constitute a fundamental class of biologically relevant compounds. However, despite the interest in these motifs, synthetic strategies have traditionally employed polar retrosynthetic disconnections. These methods typically entail the use of stoichiometric amounts of toxic and highly sensitive reagents, thereby limiting the substrate scope and practicality for scale up. In this work, an efficient protocol for the asymmetric synthesis of unnatural α-amino acids is realized through photoredox-mediated C–O bond activation in oxalate esters of aliphatic alcohols as radical precursors. The developed system uses a chiral glyoxylate-derived *N*-sulfinyl imine as the radical acceptor and allows facile access to a range of functionalized unnatural α-amino acids through an atom-economical redox-neutral process with CO_2_ as the only stoichiometric byproduct.

## Abstract 2

A practical protocol for the stereoselective synthesis of unnatural α-amino acids is disclosed, using ubiquitous aliphatic alcohols as radical precursors to form the α-amino acid backbone under redox-neutral photoredox-mediated conditions.

## Graphical abstract

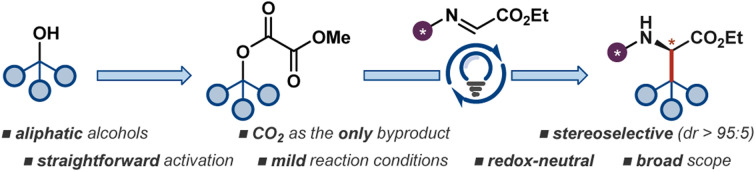

## Introduction

**Fig. 1:**
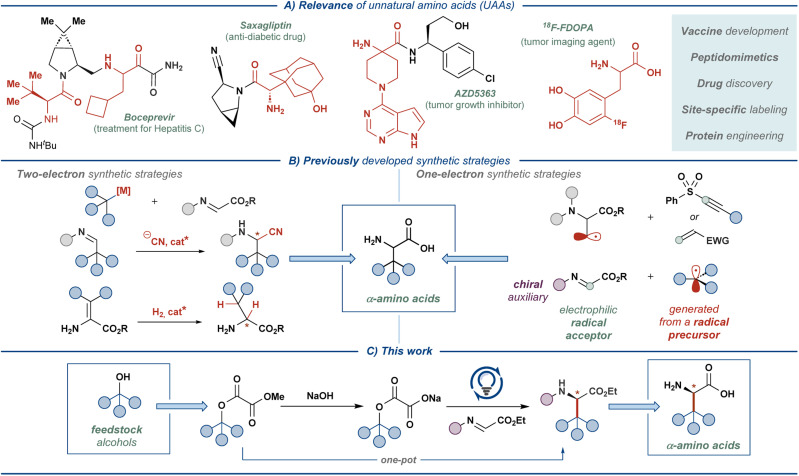
Relevance of unnatural amino acids (UAAs) and their synthesis.

Unnatural amino acids (UAAs) represent a class of bioactive compounds with diverse applications in the pharmaceutical industry, biomedical research, and materials science.^1,2^ Most commonly, UAAs serve as the building blocks for the synthesis of small-molecule drugs or as the property-modulating moieties in peptide and peptidomimetic-based medicines (Fig. 1A).^2^ Additionally, UAA-decorated peptides are used in the development of biomaterials, biosensors, and drug delivery systems, capitalizing on the tunable non-covalent interactions in specifically designed UAA residues.^3^ Furthermore, UAAs can be used for appending NMR-active or radioactive tracers to proteins, enabling detailed studies of protein function as well as medical applications, such as oncological imaging.^4,5^

The broad applicability of UAAs has stimulated the development of a multitude of (non)stereoselective approaches for their synthesis (Fig. 1B).^1,6^ The majority of such synthetic strategies rely on well-established reaction manifolds proceeding through closed-shell intermediates, such as asymmetric hydrogenation, electrophilic amination, Mannich and Strecker-type alkylations, and Petasis borono–Mannich reaction.^7,8^ In recent years, reaction manifolds featuring open-shell intermediates have also gained significant attention, prompted by the advances in photoredox catalysis^9^ and electrosynthesis.^10^ In these manifolds, UAAs are typically accessed through the addition of carbon-centered radicals (C-radicals) to glyoxylate imine or dehydroalanine derivatives using redox-active C-radical precursors, such as *N*-phthalimidoyl esters, trifluoroborates, amines, and others.^11,12^ Alternatively, the radicals are generated at the amino acid backbone, enabling appending of redox-inactive molecules onto the amino acid side-chain.^13^ Our previous work saw the entrance to such one-electron reaction manifolds with feedstock carboxylic acids as radical precursors and a chiral glyoxylate-derived *N*-sulfinyl imine as the radical acceptor.^14^ The chiral-at-sulfur *N*-sulfinyl functionality served as an effective chiral auxiliary, providing β-branched UAAs with excellent stereoselectivity (>95 : 5 dr) at the α-stereogenic center. Direct oxidative activation of unfunctionalized carboxylic acids allowed realizing the developed transformation as an overall redox-neutral reaction, providing stereoselective access to a range of amino acid derivatives with high atom economy and under mild reaction conditions. In the current work, we sought to translate this synthetic approach to a more challenging yet equally ubiquitous class of substrates — aliphatic alcohols (Fig. 1C).

Mesolytic activation of the C–O bond in aliphatic alcohols presents a formidable challenge and typically requires stoichiometric activating agents, such as phosphines and various redox-active esters and thioesters.^15–17^ Among these, oxalate esters emerged as a prominent traceless activating group. Initially, Overman and co-workers demonstrated reductive activation of alkyl oxalates with an appended redox-active *N*-phthalimidoyl group, realizing several Giese-type radical addition reactions under photocatalytic conditions.^18,19^ Significant drawbacks of these systems stemmed from the use of additional redox-active activating groups and the need for stoichiometric reducing agents, significantly limiting the applicability of such reactions. Subsequently, these drawbacks were eliminated by employing unfunctionalized alkyl oxalate salts as the radical precursors, allowing entry to the complementary redox-neutral Giese-type manifolds.^20^ Under these conditions, the oxalate salts are activated through one-electron oxidation by the photocatalyst to furnish a carboxylate radical, which eliminates two molecules of CO_2_ and delivers the key C-radical intermediate. Alkyl oxalate salts were successfully employed as radical precursors in numerous transformations, such as Giese-type addition reactions,^21–24^ alkynylation,^25–27^ arylation,^28,29^ halogenation,^30–33^ and Minisci-type manifolds,^34^ as well as in total synthesis.^30–33^ Additionally, these radical precursors were incorporated into several metallaphotoredox manifolds.^35–39^ Cognizant of the versatile reactivity of alkyl oxalate salts, we sought to extend their utility to the synthesis of UAAs. Herein, we disclose a redox-neutral stereoselective strategy for constructing a diverse array of UAAs by activating feedstock alcohols *via* oxalate esters.

## Results and discussion

### Reaction design and development

**Fig. 2:**
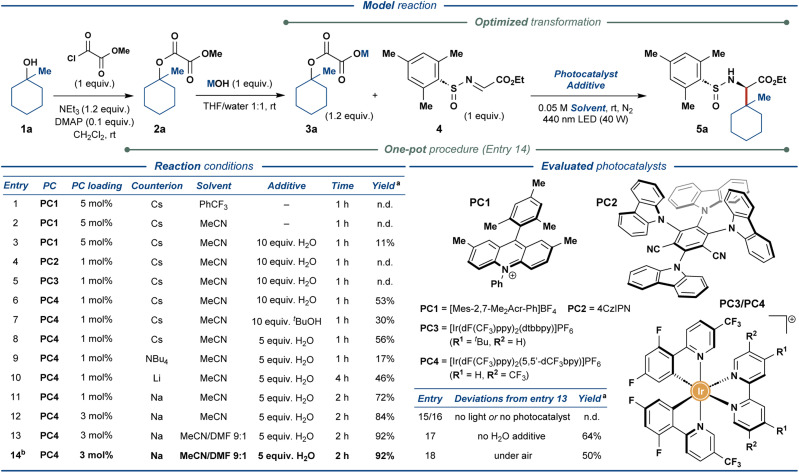
Optimization of the reaction conditions for photoredox-mediated synthesis of UAAs from aliphatic alcohols. ^a^ NMR yields are specified. ^b^ One-pot synthesis of 5a from methyl oxalate ester 2a.

To realize the outlined traceless activation strategy, tertiary alcohol 1a was converted to the corresponding methyl oxalate ester 2a, followed by hydrolysis of the methyl ester functionality to furnish the model oxalate radical precursor 3a. The model substrate 3a was then used to optimize the envisioned photocatalytic reaction with the chiral *N*-sulfinyl imine 4 as the radical acceptor (Fig. 2). Exposing cesium oxalate salt 3a-Cs to the photocatalytic reaction conditions optimized for the carboxylic acids as the radical precursors^14^ provided no desired product 5a (entry 1), presumably due to the markedly lower solubility of oxalate relative to carboxylate salts in the employed solvent (PhCF_3_). Changing the solvent to MeCN (entry 2) and addition of 10 equiv. of water (entry 3) greatly increased the solubility of the oxalate radical precursor; however, only minimal amounts of the desired product were observed (11% yield, entry 3). Gratifyingly, the screening of photoredox catalysts (entries 3–6) revealed a sharp increase in the product yield up to 53% when utilizing [Ir(dF(CF_3_)ppy)_2_(5,5′-dCF_3_bpy)]PF_6_ (PC4) as photocatalyst (entry 6). Alternative proton sources proved less effective than water (entry 7) while lowering the amount of water proved marginally beneficial to the reaction (entry 8). The screening of oxalate counterions (entries 8–11) revealed the sodium oxalate salt as the most effective substrate, providing the desired product in 72% yield (entry 11). Increasing the photocatalyst loading resulted in a further increase in the yield of the reaction up to 84% (entry 12). Finally, increasing the solubility of the starting oxalate by utilizing DMF as a co-solvent delivered the desired product in an excellent yield of 92% (entry 13). To improve the practicality of the disclosed transformation, the reaction was also conducted in a one-pot fashion with methyl oxalate ester 2a as the substrate, demonstrating no adverse effects on the reaction outcome (92% yield, entry 14). Control experiments without light (entry 15) or photocatalyst (entry 16) displayed no product formation while excluding the water additive diminished the yield of the reaction down to 64% (entry 17). Conducting the reaction open to air still provided the desired product in 50% yield (entry 18), demonstrating a markable resilience of the disclosed protocol. Notably, the above reactions furnished the desired product 5a with excellent stereoselectivity (>95 : 5 dr).

### Reaction scope

**Fig. 3:**
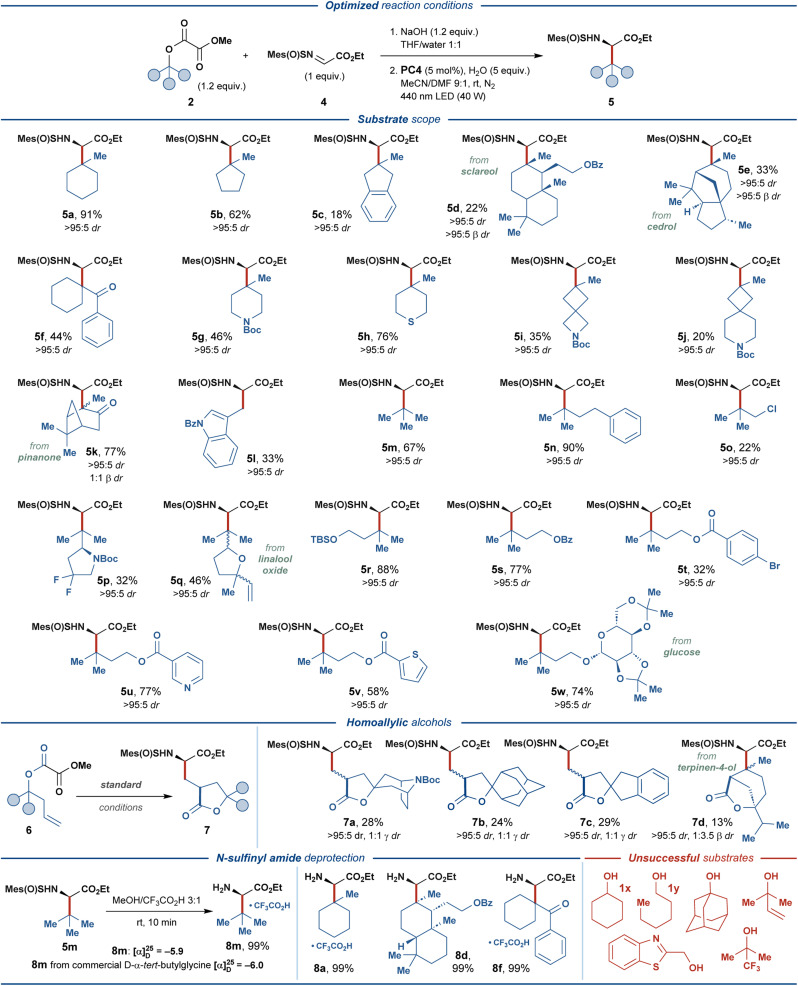
Substrate scope of photoredox-mediated synthesis of UAAs 5 and 7, and deprotection of the *N*-sulfinyl amide-functionalized products 5.

With the optimized reaction conditions (entry 14, Fig. 2), the generality of the disclosed protocol was investigated for a range of alkyl methyl oxalate substrates 2, derived from the corresponding aliphatic alcohols 1 (Fig. 3). The model tertiary oxalate substrate 2a delivered the desired amino acid product 5a with an excellent isolated yield of 91%. Unfortunately, all attempts to realize the disclosed transformation for simple secondary and primary alcohols, such as cyclohexanol (1x) and *n*-hexanol (1y) proved unsuccessful, and the subsequent investigation of the scope of the reaction focused on substrates derived from tertiary alcohols.

Carbocyclic alcohols 1b–k displayed varying compatibility with the disclosed transformation. Generally, higher isolated yields were observed for less sterically-encumbered substrates, such as 2b, 2f, 2g, and 2h (44–76% yields). In contrast, complex polycyclic substrates 2d and 2e were less effective, providing 5d and 5e in 22% and 33% yields, respectively. Contrary to this trend, bicyclo[3.1.1]heptane-containing substrate 2k and 2-indanol-based substrate 2c provided the respective amino acid products in 77% and 18% isolated yields. Spirocyclic substrates 2i and 2j proved compatible with the disclosed protocol, despite delivering the respective products in lower yields (35% and 20%, respectively). Acyclic tertiary oxalate substrates 2m and 2n provided the desired products in good and excellent yields of 67% and 90%, respectively, while the related chloride-substituted substrate 2o was less effective (22% yield). Intriguingly, the terminal alkene–containing substrate 2q and the primary alcohol substrate 2l successfully delivered the respective products, albeit in moderate yields (46% and 33%, respectively). Substrate 2p, containing endocyclic fluoro-functionality and a stereocenter adjacent to the reactive C-center, delivered the expected product in 32% yield with no stereoerosion.

To further investigate the functional group compatibility of the disclosed transformation, we prepared a series of functionalized oxalate substrates 2r–w derived from 3-methyl-1,3-butanediol. The primary alcohol functionality in this diol was selectively decorated with various functional groups, while the tertiary alcohol group was activated as the methyl oxalate ester. Gratifyingly, silyl ether (2r), benzoate (2s), nicotinate (2u), 2-thiophenecarboxylate (2v), and glycoside (2w) diol-derived substrates provided the expected amino acid products in good to excellent yields (58–88%). As expected, bromine-containing substrate 2t proved less effective and delivered the amino acid product 5t in 32% yield. Inspired by the previously developed photocatalytic systems featuring the oxalate activating group,^40^ we investigated several substrates derived from homoallylic alcohols 6. For such substrates, eliminating the second CO_2_ molecule from the one-electron oxidized oxalate (*vide supra*) is outcompeted by the intramolecular radical addition of the transient oxyacyl radical to the double bond. The resulting primary C-radical then undergoes addition to *N*-sulfinyl imine 4 to furnish γ-branched amino acid products 7. The carbocyclic homoallylic substrates 6a–c engaged in the reaction to provide synthetically challenging spirocyclic products 7a–c, albeit in relatively low yields (24–29%). Interestingly, a substrate containing an endocyclic alkene functionality 6d could still provide a bicyclic cyclization/radical addition product 7d in 13% yield, despite unfavorable sterical characteristics (*cf.* product 5e).

To conclude, while displaying suboptimal yields for some products, the disclosed transformation proved compatible with various structural motives and functional groups, such as ketone, ketal, *N-tert*-butyloxycarbonyl, alkenyl, thioether, silyl ether, fluorine, spirocycles, aromatic esters, pyridine, thiophene, and indole. Additionally, several biologically-relevant substrates derived from sclareol, cedrol, pinanone, linalool oxide, glucose, and terpinene were compatible with the developed protocol. Excellent diastereoselectivity was observed for the α-stereogenic center (>95 : 5 dr) in all of the produced amino acid products, while the sterically encumbered products 5d and 5e also displayed excellent diastereoselectivity at the β-stereogenic center (>95 : 5 β dr). The practicality of the developed protocol was highlighted through the straightforward removal of the chiral auxiliary. The *N*-sulfinyl group was removed from the amino acid adducts 5a, 5d, 5f, and 5m under mildly acidic conditions, providing the respective amino acid products 8 in quantitative isolated yields. Comparing the specific optical rotation for product 8m and the corresponding commercial amino acid derivative revealed full retention of the α-stereogenic center in 8m during *N*-sulfinyl deprotection and confirmed the proposed absolute configuration of the product (*R*).

The main limitation of the disclosed protocol derived from secondary and primary alcohols, such as 1x and 1y. For these substrates, full consumption of the starting materials was observed by ^1^H NMR under optimized conditions, while only trace amounts of the desired products could be detected (for further details on unsuccessful substrates, see Fig. S4†).

### Mechanistic considerations

**Fig. 4:**
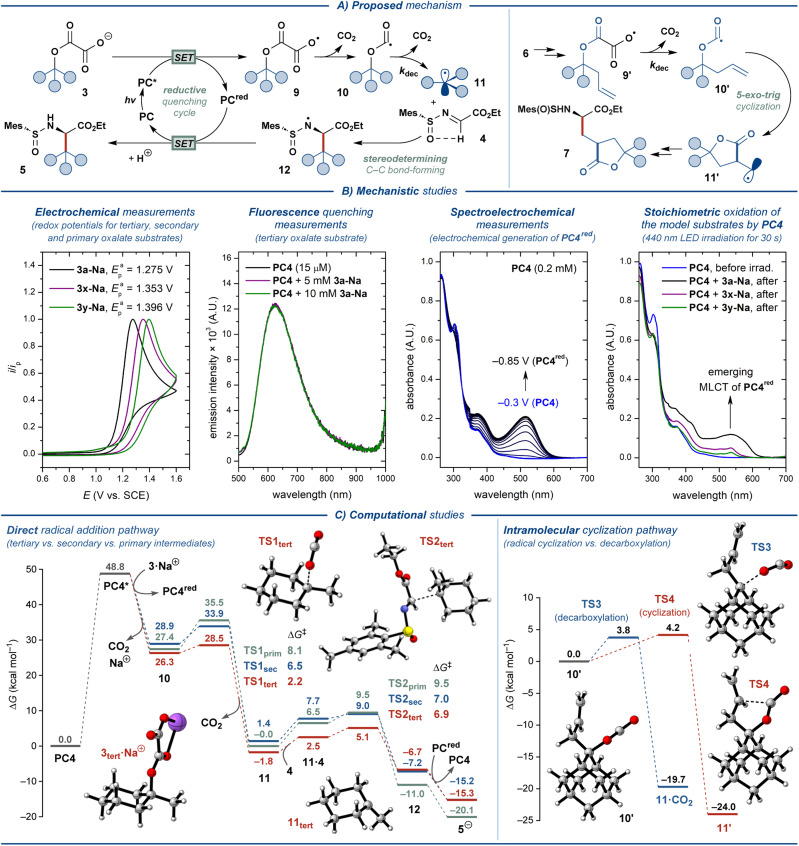
Mechanistic considerations.

Based on the literature precedents, a plausible mechanism for the disclosed transformation was proposed (Fig. 4A).^14,23,41^ The photocatalytic cycle is onset by excitation of the photocatalyst PC by blue light (*λ* ≈ 440 nm), followed by quenching of the excited-state photocatalyst PC* by oxalate ester salt 3 through single-electron transfer (SET). This step furnishes a reduced ground-state photocatalyst PC^red^ and carboxylate radical 9, which readily eliminates CO_2_ to form oxyacyl radical 10. The latter eliminates the second molecule of CO_2_ to produce the key C-radical intermediate 11. This radical engages in the stereodetermining C–C bond-forming step with *N*-sulfinyl imine 4, furnishing *N*-radical intermediate 12. As has been detailed previously,^14^ the stereochemical outcome of this step is defined by the conformation of the radical acceptor 4, which is set by intramolecular hydrogen bonding between the α-C–H hydrogen and the O-atom of the sulfone moiety. Finally, intermediate 12 transforms into the desired product 5 upon SET from the reduced photocatalyst PC^red^ and protonation from solvent, concluding the photocatalytic cycle. Oxalate ester substrates 6 derived from homoallylic alcohols follow a complementary mechanistic pathway (Fig. 4A). For this class of substrates, elimination of CO_2_ from oxyacyl radical 10′ is outcompeted by 5-*exo-trig* cyclization to furnish a primary C-radical 11′, which subsequently engages in the key C–C bond-forming reaction with 4 to deliver the lactone-containing amino acid product 7.

The feasibility of the outlined mechanism and the observed reactivity patterns were investigated with a series of spectroscopical, electrochemical, and computational studies (Fig. 4B and C, see the ESI† for additional data). The oxalate ester salt 3a-Na displayed sufficiently low oxidation potential (*E*_pa_ = 1.28 V; all potentials are specified *vs.* SCE) to quench the excited states of all of the evaluated oxidizing photocatalysts (PC1–PC4, *E*(PC*/PC^red^ ≈ 1.2–2.1 V)).^41–45^ However, only photocatalyst PC4 promoted the desired reaction. Similar to the previously described decarboxylative photocatalytic system,^14^PC2 and PC3 are likely unsuitable due to the considerably reductive character of their reduced ground state (*E*(PC/PC^red^) = −1.21 V and −1.37 V, respectively), leading to deleterious one-electron reduction of the imine substrate 4 (*E*^c^_p_ = −1.34 V). The less negative redox potentials for PC1 and PC4 (*E*(PC/PC^red^) = −0.58 V and −0.69 V, respectively) allow avoiding such reductive side-reaction. While PC1 proved highly effective for the decarboxylative synthesis of UAAs from carboxylate salts,^14^ it could only promote the reaction with PhCF_3_ as the solvent. The lower solubility of oxalate relative to carboxylate salts in PhCF_3_ is likely to be the basis for the impeded reactivity of PC1 for decarboxylative activation of oxalate ester salts. To our surprise, the steady-state fluorescence quenching experiments displayed no quenching of the excited-state PC4 by the model substrate 3a-Na (Fig. 4B). Thereby, we sought to support the outlined reductive quenching cycle through alternative stoichiometric experiments. Electroreduction of PC4 in a spectroelectrochemical cell upon sweeping the potential of the working electrode from −0.3 V to −0.85 V resulted in the gradual appearance of an MLCT absorption band (*λ*_max_ = 515 nm), as expected for reduction of an Ir(iii) polypyridyl complex to the Ir(ii) state (PC4^red^). Gratifyingly, the formation of a similar absorption band (*λ*_max_ = 535 nm) was observed for the solution of PC4 in the presence of 3a after 30 s of irradiation with 440 nm LED, supporting the proposed reductive quenching cycle, yet implying that the exact operating mechanism is more complex. Conducting the same experiments with the oxalates derived from secondary (1x) and primary alcohols (1y) displayed the highly impeded ability of these substrates to engage in electron transfer with PC4. This observation is in agreement with the increased oxidation potentials for these substrates (*E*^a^_p_ ≈ 1.35 V and 1.40 V for 3x-Na and 3y-Na, respectively). Therefore, the primary factor precluding the use of secondary and primary alcohol substrates in the disclosed transformation is the unfavorable oxidative SET. As has been proposed previously,^24^ the secondary factor for the decreased reactivity of primary and secondary alcohol substrates is likely the reduced rate constant for decarboxylation of the respective oxyacyl radicals 10 (*k*_dec_ ≈ 10^5^ s^−1^ and 10^2^ s^−1^ for the oxalates derived from tertiary and secondary/primary alcohols, respectively).^46^ Finally, the fluorescence quenching experiments with PC4 and *N*-sulfinyl imine 4 displayed no quenching (Fig. S2†), supporting that a radical–radical coupling pathway does not operate under the optimized reaction conditions.

The proposed mechanism was further investigated by computational DFT (density functional theory) studies with oxalate substrates 3a, 3x, and 3y, designated as 3_tert_, 3_sec_, and 3_prim_, respectively, using *N*-sulfinyl imine 4 as the radical acceptor (Fig. 4C, left; see the ESI† for details).^47^ The oxidation of the oxalate salts to the corresponding radicals by the excited photocatalyst leads to a barrierless decarboxylation to the oxyacyl radicals through an exothermic process (even though the computational approach does not take into account any barriers that arise from single-electron transfer processes). The second decarboxylation step revealed a clear reactivity trend for the three model substrates, displaying the highest energy barrier for the primary (Δ*G*^‡^ ≈ 8.1 kcal mol^−1^) and secondary (Δ*G*^‡^ ≈ 6.5 kcal mol^−1^) substrates, and the lowest barrier for the tertiary substrate (Δ*G*^‡^ ≈ 2.2 kcal mol^−1^). The formed C-centered radicals react with the *N*-sulfinyl imine in the stereodetermining C–C bond-forming step that was found to be rate-limiting, yet facile (Δ*G*^‡^ ≈ 6.9, 7.0, and 9.5 kcal mol^−1^, for the tertiary, secondary and primary model substrates, respectively). Notably, the low absolute values of the computed energy barriers for both decarboxylation and C–C bond-forming steps contest the experimentally observed difference in reactivity for different classes of substrates 3. Nevertheless, the calculations support the observed reactivity trend and indicate that the compatibility of the substrate with the disclosed protocol is defined by the steps preceding the C–C bond formation.

Additionally, computational investigation of the key intramolecular cyclization step for model substrate 7, derived from a homoallylic alcohol, revealed nearly equal reaction barriers for the cyclization and decarboxylation pathways (Δ*G*^‡^ ≈ 4.2 and 3.8 kcal mol^−1^, respectively, Fig. 4C, right). Thereby, the suboptimal observed yields for substrates 7a–d (<30%) are likely due to the significant contribution from deleterious decarboxylation reaction.

## Conclusions

The developed photocatalytic system allows straightforward access to a diverse set of unnatural amino acids using ubiquitous alcohols as radical precursors, which are activated through photoinduced oxidation of the corresponding alkyl oxalates. Utilizing homoallylic alcohols as substrates further extends the scope of the disclosed protocol to encompass synthetically challenging spirocyclic lactone–decorated amino acids. Mechanistic insight highlights the intricate dependence of the reaction efficiency on the nature of the alcohol substrates.

## Data availability

Detailed synthetic procedures, complete characterization data for all new compounds and computational details can be found in the ESI.†

## Author contributions

M. D. K. conceptualized and directed the project. G. R. A., E. V. S., A. S. and M. D. K. designed the experiments described in this article. G. R. A., E. V. S., A. S., J. L., R. W. and A. M. conducted and analyzed the experiments described in this article. P. D. designed, conducted and analyzed the computational studies. G. R. A., E. V. S., A. S., P. D. and M. D. K. contributed to discussing the results and drafting the manuscript.

## Conflicts of interest

There are no conflicts to declare.

## Supplementary Material

SC-015-D4SC00403E-s001
