# Supplementary material for: Asymmetric synthesis of unnatural α-amino acids through photoredox-mediated C–O bond activation of aliphatic alcohols
Source: Chem Sci. 2024 Apr 17;15(19):7316–23. doi: 10.1039/d4sc00403e (PMC11095513; doi:10.1039/d4sc00403e)
Supplement: SC-015-D4SC00403E-s001 [file SC-015-D4SC00403E-s001.pdf]

Electronic Supplementary Material (ESI) for Chemical Science.
This journal is © The Royal Society of Chemistry 2024

*Supplementary Information*

# Asymmetric synthesis of unnatural α-amino acids through photoredox-mediated C–O bond activation of aliphatic alcohols

*Gregory R. Alvey,*[a] *Elena V. Stepanova,*[a,b] *Andrey Shatskiy,*[a] *Josefin Lantz,*[a] *Rachel Willemsen,*[a] *Alix Munoz,*[a] *Peter Dinér,*[a] *Markus D. Kärkäs**[a]

[a] Department of Chemistry, KTH Royal Institute of Technology, SE-100 44 Stockholm, Sweden

[b] Tomsk Polytechnic University, Lenin Avenue 30, 634050 Tomsk, Russia

** E-mail:* karkas@kth.se

## *Table of Contents*


**1. *General methods*** ........ **S1**

**2. *Electrochemical, fluorescence quenching and spectroelectrochemical studies*** ........ **S1**

**3. *Computational studies*** ........ **S2**

**4. *Synthetic procedures*** ........ **S4**

4.1 Synthesis of starting materials ........ S4

Compound **1r** (4-((*tert*-butyldimethylsilyl)oxy)-2-methylbutan-2-ol) ........ S4

Compound **1s** (4-(benzoyloxy)-2-methylbutan-2-ol) ........ S4

Compound **1u** (4-(benzoyloxy)-2-methylbutan-2-ol) ........ S5

Compound **1v** (4-(2-thiophenecarboxy)-2-methylbutan-2-ol) ........ S5

Compound **1t** (3-hydroxy-3-methylbutyl 4-bromobenzoate) ........ S5

Compound **1d** (2-(2-hydroxy-2,5,5,8a-tetramethyldecahydronaphthalen-1-yl)ethyl benzoate) ........ S6

Compound **1w** (1-(2:3,4:6-diacetone β-D-glucopyranosyloxy) 3-hydroxy-3-methylbutane) ........ S6

Compound **1l** ((3-(hydroxymethyl)-1H-indol-1-yl)(phenyl)methanone) ........ S7

Compounds **2i**, **2j**, **2p** (*tert*-alcohols from ketones) ........ S7

Compounds **SI-3**, **SI-4**, **SI-5** (homoallylic alcohols) ........ S8

Compounds **2a**–**2w**, **6a**–**6d** (alkyl methyl oxalates) ........ S8

Compounds **3a-Na**, **3x-Na**, **3y-Na** (alkyl oxalate salts) ........ S8

4.2 General procedure for the photoredox-mediated synthesis of unnatural amino acids ........ S9

Compounds **5a**–**5w**, **7a–7d** (unnatural α-amino acids) ........ S9

4.3 General procedure for *N*-sulfinyl amide deprotection ........ S9

Compounds **8a**, **8d**, **8f**, **8i** (*N*-deprotected unnatural α-amino acids) ........ S9

4.4 Unsuccessful substrates ........ S9

**5. *Analytical data*** ........ **S11**

Compound **SI-1** (3-hydroxy-3-methylbutan-1-ol β-D-glucopyranoside) ........ S11

Compound **SI-2** (3-(((*tert*-butyldimethylsilyl)oxy)methyl)-1*H*-indole) ........ S11

Compound **SI-3** (*tert*-butyl (1*R*,3*r*,5*S*)-3-allyl-3-hydroxy-8-azabicyclo[3.2.1]octane-8-carboxylate) S11

Compound **SI-4** ((1*r*,3*r*,5*r*,7*r*)-2-allyladamantan-2-ol) ........ S12

Compound **SI-5** (2-allyl-2,3-dihydro-1*H*-inden-2-ol) ........ S12

Compound **1d** (2-((1*R*,2*R*,4a*S*,8a*S*)-2-hydroxy-2,5,5,8a-tetramethyldecahydronaphthalen-1-yl)ethyl benzoate) ........ S12

Compound **1i** (*tert*-butyl 6-hydroxy-6-methyl-2-azaspiro[3.3]heptane-2-carboxylate) ........ S13

Compound **1j** (*tert*-butyl 2-hydroxy-2-methyl-7-azaspiro[3.5]nonane-7-carboxylate) ........ S13

Compound **1l** ((3-(hydroxymethyl)-1*H*-indol-1-yl)(phenyl)methanone)........................................ S13
Compound **1p** (*tert*-butyl (S)-4,4-difluoro-2-(2-hydroxypropan-2-yl)pyrrolidine-1-carboxylate) .... S14
Compound **1r** (4-((*tert*-butyldimethylsilyl)oxy)-2-methylbutan-2-ol)............................................ S14
Compound **1s** (4-(benzoyloxy)-2-methylbutan-2-ol) ..................................................................... S14
Compound **1t** (3-hydroxy-3-methylbutyl 4-bromobenzoate)........................................................ S15
Compound **1u** (4-(nicotynoyloxy)-2-methylbutan-2-ol) ................................................................ S15
Compound **1v** (4-(2-thiophenecarboxy)-2-methylbutan-2-ol) ....................................................... S15
Compound **1w** (1-(2:3,4:6-diacetone β-D-glucopyranosyloxy) 3-hydroxy-3-methylbutane)........... S16
Compound **2a** (methyl (1-methylcyclohexyl) oxalate)................................................................... S16
Compound **2b** (methyl (1-methylcyclopentyl) oxalate).................................................................. S16
Compound **2c** (methyl (2-methyl-2,3-dihydro-1*H*-inden-2-yl) oxalate) ......................................... S17
Compound **2d** ((1*R*,2*R*,4a*S*,8a*S*)-1-(2-(benzoyloxy)ethyl)-2,5,5,8a-tetramethyldecahydronaphthalen-2-yl methyl oxalate) .............................................................................................................. S17
Compound **2e** (methyl ((3*R*,3a*S*,6*R*,7*R*,8a*S*)-3,6,8,8-tetramethyloctahydro-1*H*-3a,7-methanoazulen-6-yl) oxalate) ........................................................................................................................ S18
Compound **2f** (1-benzoylcyclohexyl methyl oxalate)...................................................................... S18
Compound **2g** (1-(*tert*-butoxycarbonyl)-4-methylpiperidin-4-yl methyl oxalate) ........................... S19
Compound **2h** (methyl (4-methyltetrahydro-2*H*-thiopyran-4-yl) oxalate) ...................................... S19
Compound **2i** (2-(*tert*-butoxycarbonyl)-6-methyl-2-azaspiro[3.3]heptan-6-yl methyl oxalate)....... S19
Compound **2j** (2-(*tert*-butoxycarbonyl)-6-methyl-2-azaspiro[3.3]heptan-6-yl methyl oxalate)....... S20
Compound **2k** ((1*R*,2*R*,5*R*)-(+)-2-hydroxy-3-pinanone methyl oxalate).......................................... S20
Compound **2l** ((1-benzoyl-1*H*-indol-3-yl)methyl methyl oxalate).................................................. S21
Compound **2m** (*tert*-butyl methyl oxalate)..................................................................................... S21
Compound **2n** (methyl (2-methyl-4-phenylbutan-2-yl) oxalate) ..................................................... S21
Compound **2o** (1-chloro-2-methylpropan-2-yl methyl oxalate) ..................................................... S22
Compound **2p** ((*S*)-2-(1-(*tert*-butoxycarbonyl)-4,4-difluoropyrrolidin-2-yl)propan-2-yl methyl oxalate) ........................................................................................................................................ S22
Compound **2q** (methyl (2-(5-methyl-5-vinyltetrahydrofuran-2-yl)propan-2-yl) oxalate) ................ S22
Compound **2r** (4-((*tert*-butyldimethylsilyl)oxy)-2-methylbutan-2-yl methyl oxalate)...................... S23
Compound **2s** (4-(benzoyloxy)-2-methylbutan-2-yl methyl oxalate) .............................................. S23
Compound **2t** (4-(4-bromobenzoyloxy)-2-methylbutan-2-yl methyl oxalate)................................. S24
Compound **2u** (4-(nicotinoyloxy)-2-methylbutan-2-yl methyl oxalate) .......................................... S24
Compound **2v** (4-(2-thiophenecarboxy)-2-methylbutan-2-yl methyl oxalate)................................ S24

Compound **2w** (2:3,4:5-diacetylidene-β-D-glucopyranosyloxy)-2-methylbutan-2-yl methyl oxalate) ........ S25

Compound **2x** (cyclohexyl methyl oxalate) ........ S25

Compound **2y** (*n*-hexyl methyl oxalate) ........ S25

Compound **3a-Na** (sodium 2-(1-methyl-cyclohexyloxy)-2-oxoacetate) ........ S26

Compound **3x-Na** (sodium 2-(cyclohexyloxy)-2-oxoacetate) ........ S26

Compound **3y-Na** (sodium 2-(hexyloxy)-2-oxoacetate) ........ S26

Compound **5a** (ethyl (*R*)-2-(((*R*)-mesitylsulfinyl)amino)-2-(1-methylcyclohexyl)acetate) ........ S27

Compound **5b** (ethyl (*R*)-2-(((*R*)-mesitylsulfinyl)amino)-2-(1-methylcyclopentyl)acetate) ........ S27

Compound **5c** (ethyl (*R*)-2-(((*R*)-mesitylsulfinyl)amino)-2-(2-methyl-2,3-dihydro-1*H*-inden-2-yl)acetate) ........ S27

Compound **5d** (2-((1*R*,2*R*,4a*S*,8a*S*)-2-((*R*)-2-ethoxy-1-(((*R*)-mesitylsulfinyl)amino)-2-oxoethyl)-2,5,5,8a-tetramethyldecahydronaphthalen-1-yl)ethyl benzoate) ........ S28

Compound **5e** (ethyl (*R*)-2-(((*R*)-mesitylsulfinyl)amino)-2-((3*R*,3a*S*,6*S*,7*R*,8a*S*)-3,6,8,8-tetramethyloctahydro-1*H*-3a,7-methanoazulen-6-yl)acetate) ........ S28

Compound **5f** (ethyl (*R*)-2-(1-benzoylcyclohexyl)-2-(((*R*)-mesitylsulfinyl)amino)acetate) ........ S29

Compound **5g** (*tert*-butyl 4-((*R*)-2-ethoxy-1-(((*R*)-mesitylsulfinyl)amino)-2-oxoethyl)-4-methylpiperidine-1-carboxylate) ........ S29

Compound **5h** (ethyl (2*R*,3*R*)-2-(((*R*)-mesitylsulfinyl)amino)-3-(tetrahydro-2*H*-thiopyran-4-yl)butanoate) ........ S30

Compound **5i** (*tert*-butyl 6-((*R*)-2-ethoxy-1-(((*R*)-mesitylsulfinyl)amino)-2-oxoethyl)-6-methyl-2-azaspiro[3.3]heptane-2-carboxylate) ........ S30

Compound **5j** (*tert*-butyl 2-((*R*)-2-ethoxy-1-(((*R*)-mesitylsulfinyl)amino)-2-oxoethyl)-2-methyl-7-azaspiro[3.5]nonane-7-carboxylate) ........ S30

Compound **5k** (ethyl (2*R*)-2-(((*R*)-mesitylsulfinyl)amino)-2-(2,6,6-trimethyl-3-oxobicyclo[3.1.1]heptan-2-yl)acetate) ........ S31

Compound **5l** (ethyl 1-benzoyl-$N^{\alpha}$-((*R*)-mesitylsulfinyl)-*D*-tryptophanate) ........ S32

Compound **5m** (ethyl (*R*)-2-(((*R*)-mesitylsulfinyl)amino)-3,3-dimethylbutanoate) ........ S32

Compound **5n** (ethyl (*R*)-2-(((*R*)-mesitylsulfinyl)amino)-3,3-dimethyl-5-phenylpentanoate) ........ S33

Compound **5o** (ethyl (*R*)-4-chloro-2-(((*R*)-mesitylsulfinyl)amino)-3,3-dimethylbutanoate) ........ S33

Compound **5p** (*tert*-butyl (*S*)-2-((*R*)-4-ethoxy-3-(((*R*)-mesitylsulfinyl)amino)-2-methyl-4-oxobutan-2-yl)-4,4-difluoropyrrolidine-1-carboxylate) ........ S33

Compound **5q** (ethyl (2*R*)-2-(((*R*)-mesitylsulfinyl)amino)-3-methyl-3-(5-methyl-5-vinyltetrahydrofuran-2-yl)butanoate) ........ S34

Compound **5r** (ethyl (*R*)-5-((tert-butyldimethylsilyl)oxy)-2-(((*R*)-mesitylsulfinyl)amino)-3,3-dimethylpentanoate) ........ S35

Compound **5s** ((*R*)-5-ethoxy-4-(((*R*)-mesitylsulfinyl)amino)-3,3-dimethyl-5-oxopentyl benzoate) .. S35

Compound **5t** ((*R*)-5-ethoxy-4-(((*R*)-mesitylsulfinyl)amino)-3,3-dimethyl-5-oxopentyl 4-bromobenzoate) ........ S35

Compound **5u** ((*R*)-5-ethoxy-4-(((*R*)-mesitylsulfinyl)amino)-3,3-dimethyl-5-oxopentyl nicotinate) S36

Compound **5v** ((*R*)-5-ethoxy-4-(((*R*)-mesitylsulfinyl)amino)-3,3-dimethyl-5-oxopentyl thiophene-2-carboxylate) ........ S36

Compound **5w** (ethyl (*R*)-2-(((*R*)-mesitylsulfinyl)amino)-3,3-dimethyl-5-(((3a*R*,4*R*,5a*R*,9a*R*,9b*S*)-2,2,8,8-tetramethylhexahydro-[1,3]dioxolo[4',5':4,5]pyrano[3,2-*d*][1,3]dioxin-4-yl)oxy)pentanoate) ........ S37

Compound **6a** ((1*R*,3*r*,5*S*)-3-allyl-8-(*tert*-butoxycarbonyl)-8-azabicyclo[3.2.1]octan-3-yl methyl oxalate) ........ S37

Compound **6b** ((1*r*,3*r*,5*r*,7*r*)-2-allyladamantan-2-yl methyl oxalate) ........ S38

Compound **6c** (2-allyl-2,3-dihydro-1*H*-inden-2-yl methyl oxalate) ........ S38

Compound **6d** ((*R*)-1-isopropyl-4-methylcyclohex-3-en-1-yl methyl oxalate, (−)-terpineolyl methyloxalate) ........ S38

Compound **7a** (*tert*-butyl 4'-((*R*)-3-ethoxy-2-(((*R*)-mesitylsulfinyl)amino)-3-oxopropyl)-5'-oxodihydro-3'*H*-8-azaspiro[bicyclo[3.2.1]octane-3,2'-furan]-8-carboxylate) ........ S39

Compound **7b** (ethyl (*R*)-2-(((*R*)-mesitylsulfinyl)amino)-3-((1*R*,3*R*)-5'-oxodihydro-3'*H*-spiro[adamantane-2,2'-furan]-4'-yl)propanoate) ........ S39

Compound **7c** (ethyl (*R*)-2-(((*R*)-mesitylsulfinyl)amino)-3-(5-oxo-1',3',4,5-tetrahydro-3*H*-spiro[furan-2,2'-inden]-4-yl)propanoate) ........ S40

Compound **7d** (ethyl (2*R*)-2-((1*S*,2*R*)-5-isopropyl-2-methyl-7-oxo-6-oxabicyclo[3.2.1]octan-2-yl)-2-(((*R*)-mesitylsulfinyl)amino)acetate) ........ S40

Compound **8a** (ethyl (*R*)-2-amino-2-(1-methylcyclohexyl)acetate, trifluoroacetate salt) ........ S41

Compound **8d** (2-((1*R*,2*R*,4a*S*,8a*S*)-2-((*R*)-1-amino-2-ethoxy-2-oxoethyl)-2,5,5,8a-tetramethyldecahydronaphthalen-1-yl)ethyl benzoate) ........ S41

Compound **8f** (ethyl (*R*)-2-amino-2-(1-benzoylcyclohexyl)acetate, trifluoroacetate salt) ........ S42

Compound **8m** (ethyl (*R*)-2-amino-3,3-dimethylbutanoate, trifluoroacetate salt) ........ S42

**6. *NMR spectra* ........ S43**

**$^1$H NMR** (500 MHz, MeOD) of compound **SI-1** ........ S43

**$^{13}$C NMR** (126 MHz, MeOD) of compound **SI-1** ........ S44

**COSY** of compound **SI-1** ........ S45

**$^1$H NMR** (500 MHz, $CDCl_3$) of compound **SI-2** ........ S46

**$^{13}$C NMR** (126 MHz, $CDCl_3$) of compound **SI-2** ........ S47

**$^1$H NMR** (500 MHz, $CDCl_3$) of compound **SI-3** ........ S48

**$^{13}$C NMR** (126 MHz, $CDCl_3$) of compound **SI-3** ........ S49

$^{1}$**H NMR** (500 MHz, $CDCl_3$) of compound **SI-4** ........ S50
$^{13}$**C NMR** (126 MHz, $CDCl_3$) of compound **SI-4** ........ S51
$^{1}$**H NMR** (500 MHz, $CDCl_3$) of compound **SI-5** ........ S52
$^{13}$**C NMR** (126 MHz, $CDCl_3$) of compound **SI-5** ........ S53
$^{1}$**H NMR** (500 MHz, $CDCl_3$) of compound **1d** ........ S54
$^{13}$**C NMR** (126 MHz, $CDCl_3$) of compound **1d** ........ S55
$^{1}$**H NMR** (500 MHz, $CDCl_3$) of compound **1i** ........ S56
$^{13}$**C NMR** (126 MHz, $CDCl_3$) of compound **1i** ........ S57
$^{1}$**H NMR** (500 MHz, $CDCl_3$) of compound **1j** ........ S58
$^{13}$**C NMR** (126 MHz, $CDCl_3$) of compound **1j** ........ S59
$^{1}$**H NMR** (500 MHz, $CDCl_3$) of compound **1l** ........ S60
$^{13}$**C NMR** (126 MHz, $CDCl_3$) of compound **1l** ........ S61
$^{1}$**H NMR** (500 MHz, $CDCl_3$) of compound **1p** ........ S62
$^{13}$**C NMR** (126 MHz, $CDCl_3$) of compound **1p** ........ S63
$^{1}$**H NMR** (500 MHz, $CDCl_3$) of compound **1r** ........ S64
$^{13}$**C NMR** (126 MHz, $CDCl_3$) of compound **1r** ........ S65
$^{1}$**H NMR** (500 MHz, $CDCl_3$) of compound **1s** ........ S66
$^{13}$**C NMR** (126 MHz, $CDCl_3$) of compound **1s** ........ S67
$^{1}$**H NMR** (500 MHz, $CDCl_3$) of compound **1t** ........ S68
$^{13}$**C NMR** (126 MHz, $CDCl_3$) of compound **1t** ........ S69
$^{1}$**H NMR** (500 MHz, $CDCl_3$) of compound **1u** ........ S70
$^{13}$**C NMR** (126 MHz, $CDCl_3$) of compound **1u** ........ S71
$^{1}$**H NMR** (500 MHz, $CDCl_3$) of compound **1v** ........ S72
$^{13}$**C NMR** (126 MHz, $CDCl_3$) of compound **1v** ........ S73
$^{1}$**H NMR** (500 MHz, $CDCl_3$) of compound **1w** ........ S74
$^{13}$**C NMR** (126 MHz, $CDCl_3$) of compound **1w** ........ S75
**COSY** of compound **1w** ........ S76
**HSQC** of compound **1w** ........ S77
$^{1}$**H NMR** (500 MHz, $CDCl_3$) of compound **2a** ........ S78
$^{13}$**C NMR** (126 MHz, $CDCl_3$) of compound **2a** ........ S79
$^{1}$**H NMR** (500 MHz, $CDCl_3$) of compound **2b** ........ S80
$^{13}$**C NMR** (126 MHz, $CDCl_3$) of compound **2b** ........ S81
$^{1}$**H NMR** (500 MHz, $CDCl_3$) of compound **2c** ........ S82

**$^{13}$C NMR** (126 MHz, $CDCl_3$) of compound **2c** ........ S83
**$^{1}$H NMR** (500 MHz, $CDCl_3$) of compound **2d** ........ S84
**$^{13}$C NMR** (126 MHz, $CDCl_3$) of compound **2d** ........ S85
**$^{1}$H NMR** (500 MHz, $CDCl_3$) of compound **2e** ........ S86
**$^{13}$C NMR** (126 MHz, $CDCl_3$) of compound **2e** ........ S87
**$^{1}$H NMR** (500 MHz, $CDCl_3$) of compound **2f** ........ S88
**$^{13}$C NMR** (126 MHz, $CDCl_3$) of compound **2f** ........ S89
**$^{1}$H NMR** (500 MHz, $CDCl_3$) of compound **2g** ........ S90
**$^{13}$C NMR** (126 MHz, $CDCl_3$) of compound **2g** ........ S91
**$^{1}$H NMR** (500 MHz, $CDCl_3$) of compound **2h** ........ S92
**$^{13}$C NMR** (126 MHz, $CDCl_3$) of compound **2h** ........ S93
**$^{1}$H NMR** (500 MHz, $CDCl_3$) of compound **2i** ........ S94
**$^{13}$C NMR** (126 MHz, $CDCl_3$) of compound **2i** ........ S95
**$^{1}$H NMR** (500 MHz, $CDCl_3$) of compound **2j** ........ S96
**$^{13}$C NMR** (126 MHz, $CDCl_3$) of compound **2j** ........ S97
**$^{1}$H NMR** (500 MHz, $CDCl_3$) of compound **2k** ........ S98
**$^{13}$C NMR** (126 MHz, $CDCl_3$) of compound **2k** ........ S99
**$^{1}$H NMR** (500 MHz, $CDCl_3$) of compound **2l** ........ S100
**$^{13}$C NMR** (126 MHz, $CDCl_3$) of compound **2l** ........ S101
**$^{1}$H NMR** (500 MHz, $CDCl_3$) of compound **2m** ........ S102
**$^{13}$C NMR** (126 MHz, $CDCl_3$) of compound **2m** ........ S103
**$^{1}$H NMR** (500 MHz, $CDCl_3$) of compound **2n** ........ S104
**$^{13}$C NMR** (126 MHz, $CDCl_3$) of compound **2n** ........ S105
**$^{1}$H NMR** (500 MHz, $CDCl_3$) of compound **2o** ........ S106
**$^{13}$C NMR** (126 MHz, $CDCl_3$) of compound **2o** ........ S107
**$^{1}$H NMR** (500 MHz, $CDCl_3$) of compound **2p** ........ S108
**$^{13}$C NMR** (126 MHz, $CDCl_3$) of compound **2p** ........ S109
**$^{1}$H NMR** (500 MHz, $CDCl_3$) of compound **2q** ........ S110
**$^{13}$C NMR** (126 MHz, $CDCl_3$) of compound **2q** ........ S111
**$^{1}$H NMR** (500 MHz, $CDCl_3$) of compound **2r** ........ S112
**$^{13}$C NMR** (126 MHz, $CDCl_3$) of compound **2r** ........ S113
**$^{1}$H NMR** (500 MHz, $CDCl_3$) of compound **2s** ........ S114
**$^{13}$C NMR** (126 MHz, $CDCl_3$) of compound **2s** ........ S115

**$^{1}$H NMR** (500 MHz, $CDCl_3$) of compound **2t** ........ S116
**$^{13}$C NMR** (126 MHz, $CDCl_3$) of compound **2t** ........ S117
**$^{1}$H NMR** (500 MHz, $CDCl_3$) of compound **2u** ........ S118
**$^{13}$C NMR** (126 MHz, $CDCl_3$) of compound **2u** ........ S119
**$^{1}$H NMR** (500 MHz, $CDCl_3$) of compound **2v** ........ S120
**$^{13}$C NMR** (126 MHz, $CDCl_3$) of compound **2v** ........ S121
**$^{1}$H NMR** (500 MHz, $CDCl_3$) of compound **2w** ........ S122
**$^{13}$C NMR** (126 MHz, $CDCl_3$) of compound **2w** ........ S123
**COSY** of compound **2w** ........ S124
**$^{1}$H NMR** (500 MHz, $CDCl_3$) of compound **2x** ........ S125
**$^{13}$C NMR** (126 MHz, $CDCl_3$) of compound **2x** ........ S126
**$^{1}$H NMR** (500 MHz, $CDCl_3$) of compound **2y** ........ S127
**$^{13}$C NMR** (126 MHz, $CDCl_3$) of compound **2y** ........ S128
**$^{1}$H NMR** (400 MHz, DMSO) of compound **3a-Na** ........ S129
**$^{13}$C NMR** (101 MHz, DMSO) of compound **3a-Na** ........ S130
**$^{1}$H NMR** (500 MHz, DMSO) of compound **3x-Na** ........ S131
**$^{13}$C NMR** (126 MHz, DMSO) of compound **3x-Na** ........ S132
**$^{1}$H NMR** (500 MHz, DMSO) of compound **3y-Na** ........ S133
**$^{13}$C NMR** (126 MHz, DMSO) of compound **3y-Na** ........ S134
**$^{1}$H NMR** (500 MHz, $CDCl_3$) of compound **5a** ........ S135
**$^{13}$C NMR** (126 MHz, $CDCl_3$) of compound **5a** ........ S136
**$^{1}$H NMR** (500 MHz, $CDCl_3$) of compound **5b** ........ S137
**$^{13}$C NMR** (126 MHz, $CDCl_3$) of compound **5b** ........ S138
**$^{1}$H NMR** (500 MHz, $CDCl_3$) of compound **5c** ........ S139
**$^{13}$C NMR** (126 MHz, $CDCl_3$) of compound **5c** ........ S140
**COSY** of compound **5c** ........ S141
**HSQC** of compound **5c** ........ S142
**$^{1}$H NMR** (500 MHz, $CDCl_3$) of compound **5d** ........ S143
**$^{13}$C NMR** (126 MHz, $CDCl_3$) of compound **5d** ........ S144
**COSY** of compound **5d** ........ S145
**HSQC** of compound **5d** ........ S146
**HMBC** of compound **5d** ........ S147
**$^{1}$H NMR** (500 MHz, $CDCl_3$) of compound **5e** ........ S148

**$^{13}$C NMR** (126 MHz, $CDCl_3$) of compound **5e** .......... S149
**COSY** of compound **5e** .......... S150
**HSQC** of compound **5e** .......... S151
**$^{1}$H NMR** (500 MHz, $CDCl_3$) of compound **5f** .......... S152
**$^{13}$C NMR** (126 MHz, $CDCl_3$) of compound **5f** .......... S153
**COSY** of compound **5f** .......... S154
**HSQC** of compound **5f** .......... S155
**$^{1}$H NMR** (500 MHz, $CDCl_3$) of compound **5g** .......... S156
**$^{13}$C NMR** (126 MHz, $CDCl_3$) of compound **5g** .......... S157
**$^{1}$H NMR** (500 MHz, $CDCl_3$) of compound **5h** .......... S158
**$^{13}$C NMR** (126 MHz, $CDCl_3$) of compound **5h** .......... S159
**COSY** of compound **5h** .......... S160
**HSQC** of compound **5h** .......... S161
**$^{1}$H NMR** (500 MHz, $CDCl_3$) of compound **5i** .......... S162
**$^{13}$C NMR** (126 MHz, $CDCl_3$) of compound **5i** .......... S163
**COSY** of compound **5i** .......... S164
**HSQC** of compound **5i** .......... S165
**$^{1}$H NMR** (500 MHz, $CDCl_3$) of compound **5j** .......... S166
**$^{13}$C NMR** (126 MHz, $CDCl_3$) of compound **5j** .......... S167
**COSY** of compound **5j** .......... S168
**HSQC** of compound **5j** .......... S169
**$^{1}$H NMR** (500 MHz, $CDCl_3$) of compound **5k**, β-diastereomer **5k-1** .......... S170
**$^{13}$C NMR** (126 MHz, $CDCl_3$) of compound **5k**, β-diastereomer **5k-1** .......... S171
**COSY** of compound **5k**, β-diastereomer **5k-1** .......... S172
**HSQC** of compound **5k**, β-diastereomer **5k-1** .......... S173
**HMBC** of compound **5k**, β-diastereomer **5k-1** .......... S174
**$^{1}$H NMR** (500 MHz, $CDCl_3$) of compound **5k**, β-diastereomer **5k-2** .......... S175
**$^{13}$C NMR** (126 MHz, $CDCl_3$) of compound **5k**, β-diastereomer **5k-2** .......... S176
**COSY** of compound **5k**, β-diastereomer **5k-2** .......... S177
**HSQC** of compound **5k**, β-diastereomer **5k-2** .......... S178
**HMBC** of compound **5k**, β-diastereomer **5k-2** .......... S179
**$^{1}$H NMR** (500 MHz, $CDCl_3$) of compound **5l** .......... S180
**$^{13}$C NMR** (126 MHz, $CDCl_3$) of compound **5l** .......... S181

**COSY** of compound **5l** ........ S182
**HSQC** of compound **5l** ........ S183
**HMBC** of compound **5l** ........ S184
**$^{1}$H NMR** (500 MHz, $CDCl_3$) of compound **5m** ........ S185
**$^{13}$C NMR** (126 MHz, $CDCl_3$) of compound **5m** ........ S186
**$^{1}$H NMR** (500 MHz, $CDCl_3$) of compound **5n** ........ S187
**$^{13}$C NMR** (126 MHz, $CDCl_3$) of compound **5n** ........ S188
**COSY** of compound **5n** ........ S189
**HSQC** of compound **5n** ........ S190
**$^{1}$H NMR** (500 MHz, $CDCl_3$) of compound **5o** ........ S191
**$^{13}$C NMR** (126 MHz, $CDCl_3$) of compound **5o** ........ S192
**$^{1}$H NMR** (500 MHz, $CDCl_3$) of compound **5p** ........ S193
**$^{13}$C NMR** (126 MHz, $CDCl_3$) of compound **5p** ........ S194
**$^{1}$H NMR** (500 MHz, $CDCl_3$) of compound **5q** ........ S195
**$^{13}$C NMR** (126 MHz, $CDCl_3$) of compound **5q** ........ S196
**COSY** of compound **5q** ........ S197
**HSQC** of compound **5q** ........ S198
**HMBC** of compound **5q** ........ S199
**$^{1}$H NMR** (500 MHz, $CDCl_3$) of compound **5r** ........ S200
**$^{13}$C NMR** (126 MHz, $CDCl_3$) of compound **5r** ........ S201
**COSY** of compound **5r** ........ S202
**HSQC** of compound **5r** ........ S203
**$^{1}$H NMR** (500 MHz, $CDCl_3$) of compound **5s** ........ S204
**$^{13}$C NMR** (126 MHz, $CDCl_3$) of compound **5s** ........ S205
**COSY** of compound **5s** ........ S206
**HSQC** of compound **5s** ........ S207
**$^{1}$H NMR** (500 MHz, $CDCl_3$) of compound **5t** ........ S208
**$^{13}$C NMR** (126 MHz, $CDCl_3$) of compound **5t** ........ S209
**COSY** of compound **5t** ........ S210
**HSQC** of compound **5t** ........ S211
**$^{1}$H NMR** (500 MHz, $CDCl_3$) of compound **5u** ........ S212
**$^{13}$C NMR** (126 MHz, $CDCl_3$) of compound **5u** ........ S213
**COSY** of compound **5u** ........ S214

**HSQC** of compound **5u** ........ S215
**$^{1}$H NMR** (500 MHz, $CDCl_3$) of compound **5v** ........ S216
**$^{13}$C NMR** (126 MHz, $CDCl_3$) of compound **5v** ........ S217
**COSY** of compound **5v** ........ S218
**HSQC** of compound **5v** ........ S219
**$^{1}$H NMR** (500 MHz, $CDCl_3$) of compound **5w** ........ S220
**$^{13}$C NMR** (126 MHz, $CDCl_3$) of compound **5w** ........ S221
**COSY** of compound **5w** ........ S222
**HSQC** of compound **5w** ........ S223
**$^{1}$H NMR** (500 MHz, $CDCl_3$) of compound **6a** ........ S224
**$^{13}$C NMR** (126 MHz, $CDCl_3$) of compound **6a** ........ S225
**$^{1}$H NMR** (500 MHz, $CDCl_3$) of compound **6b** ........ S226
**$^{13}$C NMR** (126 MHz, $CDCl_3$) of compound **6b** ........ S227
**$^{1}$H NMR** (500 MHz, $CDCl_3$) of compound **6c** ........ S228
**$^{13}$C NMR** (126 MHz, $CDCl_3$) of compound **6c** ........ S229
**$^{1}$H NMR** (500 MHz, $CDCl_3$) of compound **6d** ........ S230
**$^{13}$C NMR** (126 MHz, $CDCl_3$) of compound **6d** ........ S231
**$^{1}$H NMR** (500 MHz, $CDCl_3$) of compound **7a** ........ S232
**$^{13}$C NMR** (126 MHz, $CDCl_3$) of compound **7a** ........ S233
**COSY** of compound **7a** ........ S234
**HSQC** of compound **7a** ........ S235
**$^{1}$H NMR** (500 MHz, $CDCl_3$) of compound **7b** ........ S236
**$^{13}$C NMR** (126 MHz, $CDCl_3$) of compound **7b** ........ S237
**COSY** of compound **7b** ........ S238
**HSQC** of compound **7b** ........ S239
**$^{1}$H NMR** (500 MHz, $CDCl_3$) of compound **7c** ........ S240
**$^{13}$C NMR** (126 MHz, $CDCl_3$) of compound **7c** ........ S241
**COSY** of compound **7c** ........ S242
**HSQC** of compound **7c** ........ S243
**$^{1}$H NMR** (500 MHz, $CDCl_3$) of compound **7d** ........ S244
**$^{13}$C NMR** (126 MHz, $CDCl_3$) of compound **7d** ........ S245
**COSY** of compound **7d** ........ S246
**HSQC** of compound **7d** ........ S247

**HMBC** of compound **7d** ..... S248
**$^{1}$H NMR** (500 MHz, MeOD) of compound **8a** ..... S249
**$^{13}$C NMR** (126 MHz, MeOD) of compound **8a** ..... S250
**$^{19}$F NMR** (377 MHz, MeOD) of compound **8a** ..... S251
**$^{1}$H NMR** (500 MHz, $CDCl_3$) of compound **8d** ..... S252
**$^{13}$C NMR** (126 MHz, $CDCl_3$) of compound **8d** ..... S253
**COSY** of compound **8d** ..... S254
**HSQC** of compound **8d** ..... S255
**$^{1}$H NMR** (500 MHz, MeOD) of compound **8f** ..... S256
**$^{13}$C NMR** (126 MHz, MeOD) of compound **8f** ..... S257
**$^{19}$F NMR** (377 MHz, MeOD) of compound **8f** ..... S258
**$^{1}$H NMR** (500 MHz, MeOD) of compound **8m** ..... S259
**$^{13}$C NMR** (126 MHz, $CDCl_3$) of compound **8m** ..... S260
**$^{19}$F NMR** (377 MHz, MeOD) of compound **8m** ..... S261
**7. *Cartesian coordinates and energies*** ..... **S262**
***PC4*** ..... S262
***PC4**** ..... S263
***$PC4^{red}$*** ..... S264
***Sodium 1-methylcyclohexyloxalate ($3_{tert}$·$Na^+$)*** ..... S265
***1-Methylcyclohexyl oxyacyl radical — $CO_2$ — sodium complex ($10_{tert}$·$CO_2$·Na)*** ..... S266
***1-Methylcyclohexyl oxyacyl radical (axial) ($10_{tert}$-ax)*** ..... S266
***1-Methylcyclohexyl oxyacyl radical (equatorial) ($10_{tert}$-eq)*** ..... S267
***$CO_2$*** ..... S267
***TS for $CO_2$ elimination from 1-methylcyclohexyl oxyacyl radical (axial) ($TS1_{tert}$-ax)*** ..... S268
***TS for $CO_2$ elimination from 1-methylcyclohexyl oxyacyl radical (equatorial) ($TS1_{tert}$-eq)*** ..... S268
***1-Methylcyclohexyl radical — $CO_2$ complex ($11_{tert}$·$CO_2$)*** ..... S269
***1-Methylcyclohexyl radical (equatorial) ($11_{tert}$-eq)*** ..... S269
***1-Methylcyclohexyl radical (axial) ($11_{tert}$-ax)*** ..... S270
***s-cis conformer of N-sulfinyl imine (4)*** ..... S270
***1-Methylcyclohexyl radical (equatorial) — N-sulfinyl imine 4 re-precomplex ($11_{tert}$·4-eq)*** ..... S271
***1-Methylcyclohexyl radical (axial) — N-sulfinyl imine 4 re-precomplex ($11_{tert}$·4-ax)*** ..... S272
***re-TS for 1-methylcyclohexyl radical (equatorial) addition to N-sulfinyl imine 4 ($TS2_{tert}$-eq)*** ..... S273
***re-TS for 1-methylcyclohexyl radical (axial) addition to N-sulfinyl imine 4 ($TS2_{tert}$-ax)*** ..... S274

*(R)-configured adduct of 4 with 1-methylcyclohexyl radical (equatorial) ($12_{tert}$-eq)* ..... S275
*(R)-configured adduct of 4 with 1-methylcyclohexyl radical (axial) ($12_{tert}$-ax)* ..... S276
*Reduced (R)-configured adduct of 4 with 1-methylcyclohexyl radical ($5_{tert}^{-}$)* ..... S277
*Sodium cyclohexyloxalate (axial) ($3_{sec}·Na^{+}$-ax)* ..... S278
*Sodium cyclohexyloxalate (equatorial) ($3_{sec}·Na^{+}$-eq)* ..... S278
*Cyclohexyl oxyacyl radical (axial) – $CO_2$ – sodium complex ($10_{sec}·CO_2·Na^{+}$-ax)* ..... S279
*Cyclohexyl oxyacyl radical (axial) ($10_{sec}$-ax)* ..... S279
*Cyclohexyl oxyacyl radical (equatorial) ($10_{sec}$-eq)* ..... S280
*TS for $CO_2$ elimination from cyclohexyl oxyacyl radical (axial) ($TS1_{sec}$-ax)* ..... S280
*TS for $CO_2$ elimination from cyclohexyl oxyacyl radical (equatorial) ($TS1_{sec}$-eq)* ..... S281
*Cyclohexyl radical — $CO_2$ complex ($11_{sec}·CO_2$)* ..... S281
*Cycloxehyl radical ($11_{sec}$)* ..... S282
*Cyclohexyl radical (axial) — N-sulfinyl imine 4 re-precomplex ($11_{sec}·4$-ax)* ..... S282
*Cyclohexyl radical (equatorial) — N-sulfinyl imine 4 re-precomplex ($11_{sec}·4$-eq)* ..... S283
*re-TS for cyclohexyl radical (axial) addition to N-sulfinyl imine 4 ($TS2_{sec}$-ax)* ..... S284
*re-TS for cyclohexyl radical (equatorial) addition to N-sulfinyl imine 4 ($TS2_{sec}$-eq)* ..... S285
*(R)-configured adduct of 4 with cycloxehyl radical ($12_{sec}$)* ..... S286
*Reduced (R)-configured adduct of 4 with cycloxehyl radical ($5_{sec}^{-}$)* ..... S287
*Sodium n-hexyloxalate ($3_{prim}·Na^{+}$)* ..... S288
*n-Hexyl oxoacyl radical – $CO_2$ – sodium complex ($10_{prim}·CO_2·Na^{+}$)* ..... S288
*n-Hexyl oxyacyl radical ($10_{prim}$)* ..... S289
*TS for $CO_2$ elimination from n-hexyl oxyacyl radical ($TS1_{prim}$)* ..... S289
*n-Hexyl radical — $CO_2$ complex ($11_{prim}·CO_2$)* ..... S290
*n-Hexyl radical ($11_{prim}$)* ..... S290
*n-Hexyl radical — N-sulfinyl imine 4 re-precomplex ($11_{prim}·4$)* ..... S291
*re-TS for n-hexyl radical addition to N-sulfinyl imine 4 ($TS2_{prim}$)* ..... S292
*(R)-configured adduct of 4 with n-hexyl radical ($12_{prim}$)* ..... S293
*Reduced (R)-configured adduct of 4 with n-hexyl radical ($5_{prim}^{-}$)* ..... S294
*2-Allyladamantan-2-yl oxyacyl radical (10')* ..... S295
*TS for $CO_2$ elimination from 2-allyladamantan-2-yl oxyacyl radical (TS3)* ..... S295
*2-Allyladamantan-2-yl radical — $CO_2$ complex ($11·CO_2$)* ..... S296
*TS for intramolecular cyclization of 2-allyladamantan-2-yl oxyacyl radical (TS4)* ..... S297
*Intramolecular cyclization product from 2-allyladamantan-2-yl oxyacyl radical (11')* ..... S297

**8.** ***References*** **............................................................ S298**

## 1. ***General methods***

*N*-sulfinyl imine **4**,[1] reference compound **8m**[1] and 4CzIPN (**PC2**) photocatalyst[2] were synthesized according to the previously published procedures. NMR spectra were recorded in $CDCl_3$, MeOH-$d_4$ or DMSO-$d_6$ on Bruker Avance DMX 500 MHz or Bruker Ascend 400 MHz NMR spectrometers and internally calibrated against the residual undeuterated solvent peaks ($CHCl_3$: δ 7.26 for $^1$H NMR and δ 77.16 for $^{13}$C NMR; $CHD_2OD$: δ 3.31 for $^1$H NMR and δ 49.00 for $^{13}$C NMR; DMSO-$d_5$: δ 2.50 for $^1$H NMR and δ 39.52 for $^{13}$C NMR). The photocatalytic reactions were carried out in 8 mL or 12 mL vials equipped with a stirring bar and a septum. The reaction vials were placed in a 3D-printed polypropylene holder to maintain the distance between the reaction vial and the lamp at ca. 2 cm and illuminated with 440 nm LED (40 W, Kessil PR160L, set to maximum intensity) with continuous stirring at 1200 rpm and fan cooling. The isolated products were purified by column chromatography with silica gel (high-purity grade, 60 Å, 130–270 mesh, Sigma-Aldrich, Art. No. 288608-1KG) or by preparative thin-layer chromatography (1 mm silica gel layer on glass, 60 Å, Merk, Art. No. 1.13895.0001).

## 2. ***Electrochemical, fluorescence quenching and spectroelectrochemical studies***

Electrochemical measurements were performed under Ar in a one-compartment electrochemical cell with glassy carbon as the working electrode (⌀ 3 mm), Pt coil as the auxiliary electrode, and saturated calomel electrode (SCE) as the reference electrode, using CHI750E bipotentiostat (CH Instruments). The cyclic voltammetry (CV) measurements of alkyl oxalate salts **3a-Na**, **3x-Na** and **3y-Na** were performed at 0.05 V $s^{-1}$ scan rate on 3 mM solutions of the salts in DMF/MeCN/water 5.4/0.6/0.027 (vol.) with 0.1 M $TBAPF_6$ as the supporting electrolyte (Fig. 4B and S1).

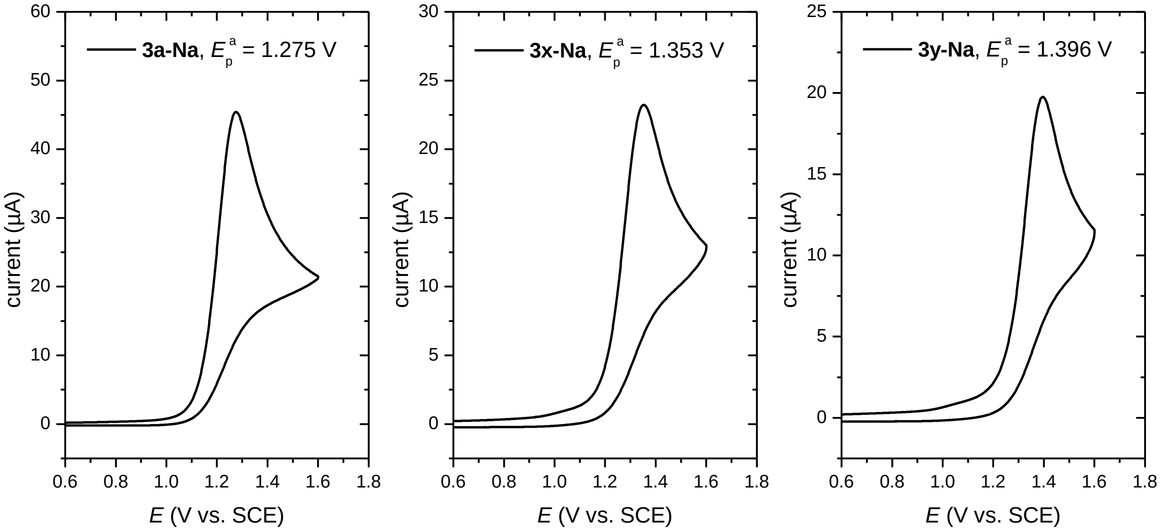


**Fig. S1.** Electrochemical measurements for alkyl oxalate salts **3a-Na**, **3x-Na** and **3y-Na**.

The steady-state fluorescence quenching measurements were performed under Ar on FS5 spectrofluorometer (Edinburgh Instruments) using 10 x 10 mm quartz cuvettes. The measurements were carried out on the solutions of photocatalyst **PC4** (15 µM) in DMF/MeCN/water 5.4/0.6/0.027 (vol.) with varying concentration of the oxalate salt **3y-Na** (0 mM, 5 mM, 10 mM) or imine **4**. The emission spectra were recorded at 500–1000 nm with excitation at 420 nm (Fig. 4B and S2).

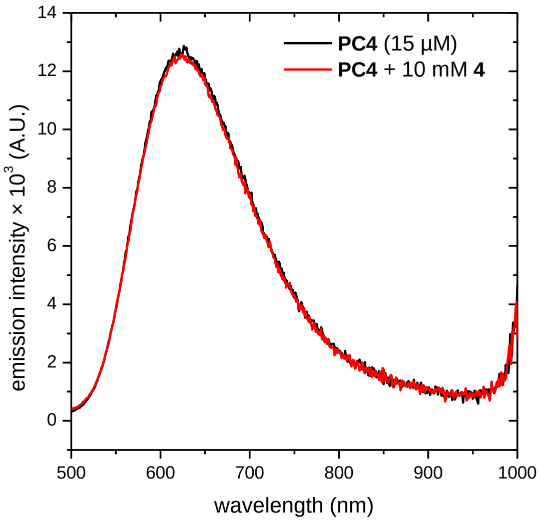


**Fig. S2.** Fluorescence quenching measurements for photocatalyst **PC4** and imine **4**.

Spectroelectrochemical measurements aiming at obtaining the UV-vis spectrum of unstable **PC4**$^{red}$ species were performed using CHI750E bipotentiostat (CH Instruments), Varian Cary 50 UV-vis Spectrophotometer, and SEC-C Spectroelectrochemical Cell (ALS, 1 mm optical path length, Pt mesh as the working and the auxiliary electrodes, and Ag/AgCl reference electrode, externally calibrated against SCE for conversion of the potential values). The measurements were performed on solution of **PC4** (0.2 mM) in DMF/MeCN/water 5.4/0.6/0.027 (vol.) with 0.1 M $TBAPF_6$ as the supporting electrolyte (Fig. 4B).

Photoinduced reduction of oxalate salts **3a-Na**, **3x-Na** and **3y-Na** by the **PC4** photocatalyst were performed under Ar on Varian Cary 50 UV-vis Spectrophotometer, using a quartz cuvette from the SEC-C Spectroelectrochemical Cell (ALS, 1 mm optical path length). The measurements were carried out on 1 mM solutions of **3a-Na**, **3x-Na** and **3y-Na** with 0.2 mM **PC4** in DMF/MeCN/water 5.4/0.6/0.027 (vol.). The UV-vis spectra were recorded before and after irradiation of the solutions with 440 nm LED (40 W, Kessil PR160L, set to maximum intensity) for 30 s (Fig. 4B). The LED was positioned ca. 2 cm from the UV-vis cuvette.

## 3. *Computational studies*

All stationary points were optimized, first at the B3LYP/6-311+G(d,p) level of theory, and further re-optimized at the B3LYP/6-311+G(d,p) level of theory,[3,4,5,6] as implemented in Gaussian 16 Rev D.01. In the optimizations, the Grimme correction for dispersion (D3) was used in combination with the Conductor-like Polarizable Continuum Model (CPCM) using the parameters for acetonitrile and the default Unified Force Field radii (UFF)[7,8] as implemented in Gaussian 16 Rev D.01.[9] All geometries were characterized as

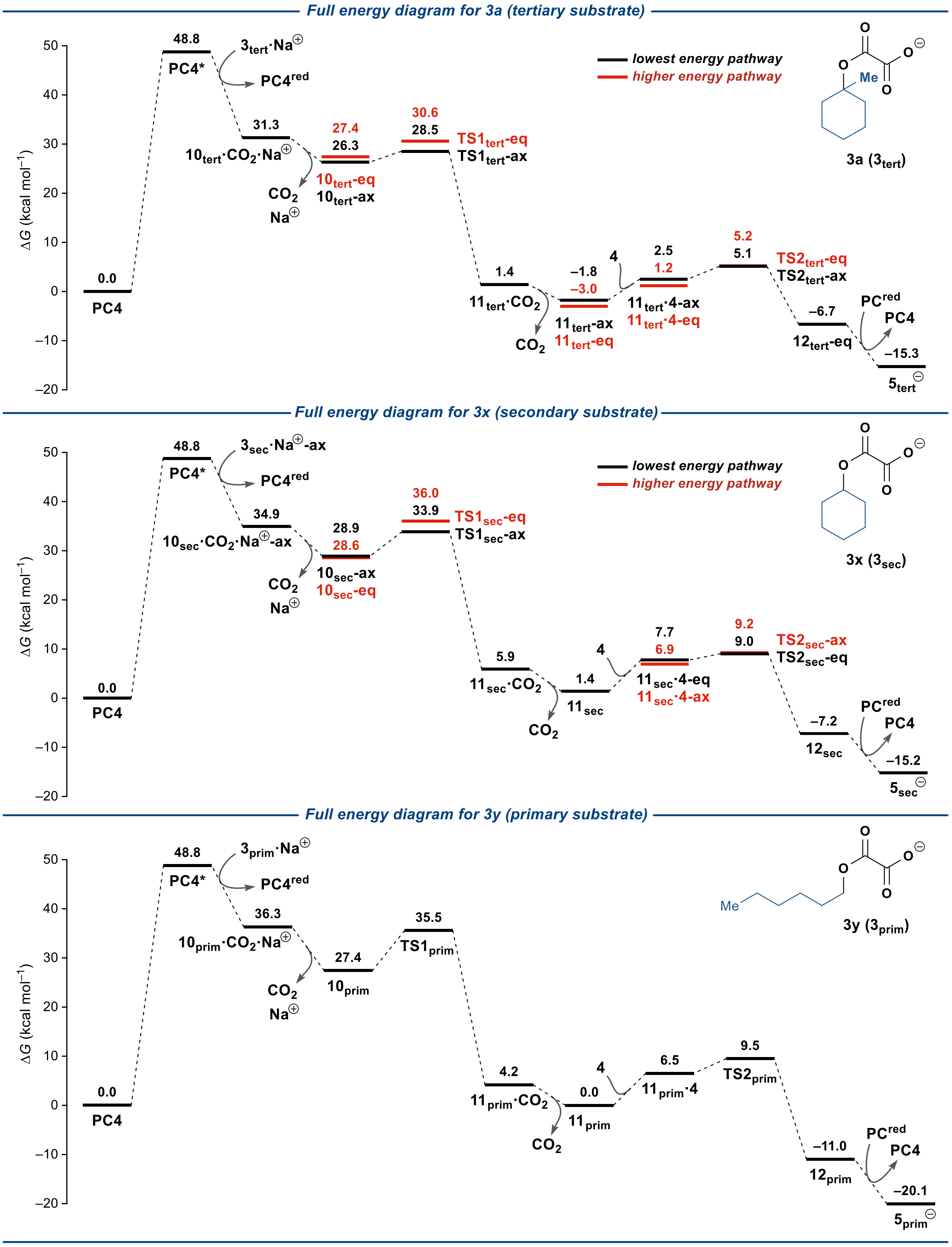


**Fig. S3.** Full calculated energy diagrams for substrates **3a** (**3**$_{tert}$), **3x** (**3**$_{sec}$), and **3y** (**3**$_{prim}$).

minima or saddle points on the potential-energy surface (PES) by using the sign of the eigenvalues of the force-constant matrix obtained from a frequency calculation. Transition states with one imaginary frequency were confirmed to describe the correct movement on the PES by mode analysis and by intrinsic reaction coordinate (IRC) calculations connecting the correct reactants and products (Fig. 4C and S3).

# 4. *Synthetic procedures*

## 4.1 *Synthesis of starting materials*

Compound **1r** (4-((*tert*-butyldimethylsilyl)oxy)-2-methylbutan-2-ol)

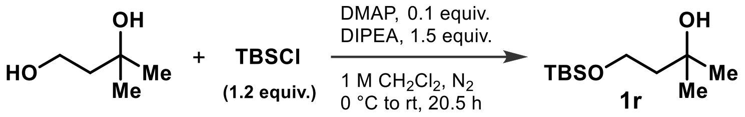


3-Methyl-1,3-butanediol (1.07 mL, 10 mmol, 1 equiv.) and DMAP (122 mg, 0.1 mmol, 0.1 equiv.) were dissolved in dry $CH_2Cl_2$ (10 mL) under $N_2$ and cooled with an ice bath. Thereafter, DIPEA (2.6 mL, 15 mmol,1.5 equiv.) and *tert*-butyldimethylsilyl chloride (1.808 g, 12 mmol, 1.2 equiv.) were added sequentially and the reaction mixture was stirred with an ice bath cooling under $N_2$. After 30 min the reaction mixture was allowed to warm to r.t. and stirred for 20 h. The reaction was quenched by sat. $NH_4Cl$ (10 mL), the organic phase was separated, and the water phase was extracted with $CH_2Cl_2$ (2 × 10 mL). The combined organic phases were dried over $Na_2SO_4$, filtered and concentrated *in vacuo* to obtain the crude product as a brown oil. The crude product was purified by column chromatography (petroleum ether/EtOAc 20:1 → 4:1) to give 2.08 g (95%) of compound **1r**.

Compound **1s** (4-(benzoyloxy)-2-methylbutan-2-ol)

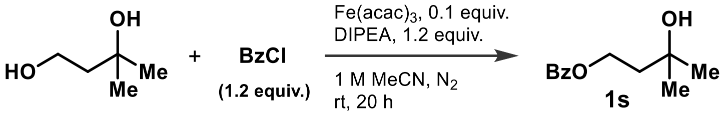


3-Methyl-1,3-butanediol (1.07 mL, 10 mmol, 1 equiv.) and $Fe(acac)_3$ (353 mg, 0.1 mmol, 0.1 equiv.) were dissolved in MeCN (10 mL) under $N_2$. Thereafter, DIPEA (2.09 mL, 12 mmol, 1.2 equiv.) and benzoyl chloride (1.4 mL, 12 mmol, 1.2 equiv.) were added sequentially and the reaction mixture was stirred for 20 h at r.t. The reaction mixture was concentrated *in vacuo*, the residue was dissolved in $CH_2Cl_2$ (20 mL) and washed with sat. aq. $Na_2CO_3$ (3 × 20 mL). The organic phase was dried over $Na_2SO_4$, filtered and concentrated *in vacuo* to obtain the crude product as a brown oil. The crude product was purified by column chromatography (petroleum ether/EtOAc 10:1→2:1) to give compound **1s** as a yellow oil (1.847 g, 89% yield).

Compound **1u** (4-(benzoyloxy)-2-methylbutan-2-ol)

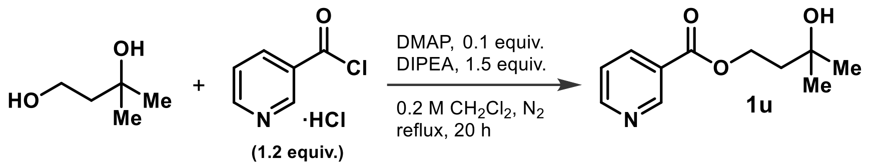


Nicotinoyl chloride hydrochloride (213.6 mg, 1.2 mmol, 1.2 equiv.) and DMAP (12.2 mg, 1.2 mmol, 0.1 equiv.) were dried *in vacuo*, then 3-methyl-1,3-butanediol (107 µL, 1 mmol, 1 equiv.) was added, dissolved in dry $CH_2Cl_2$ (5 mL), DIPEA (260 µL, 1.5 mmol, 1.5 equiv.) was added and the rection was refluxed for 20 h. Then it was cooled, $CH_2Cl_2$ (20 mL) and sat. aq. $Na_2CO_3$ (10 mL) were added, organic layer was separated and washed with sat.aq. $Na_2CO_3$ (3 × 10 mL), dried over $Na_2SO_4$, filtered, and concentrated *in vacuo*. The residue was purified on column chromatography ($CH_2Cl_2$/MeOH 10:1) to give compound **1u** as yellow oil (170 mg, 81% yield).

Compound **1v** (4-(2-thiophenecarboxy)-2-methylbutan-2-ol)

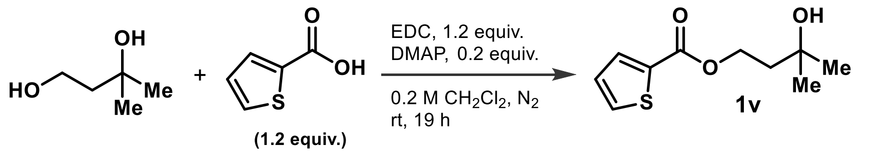


2-Thiophene-carboxylic acid (153.6 mg, 1.2 mmol, 1.2 equiv.) and DMAP (24.4 mg, 0.2 mmol, 0.2 equiv.) were dried *in vacuo*, then 3-methyl-1,3-butanediol (107 µL, 1 mmol, 1 equiv.) was added, dissolved in dry $CH_2Cl_2$ (5 mL), EDC (212 µL, 1.2 mmol, 1.2 equiv.) was added and the rection was stirred at r.t. for 19 h. $CH_2Cl_2$ (20 mL) and sat. aq. $Na_2CO_3$ (10 mL) were added, organic layer was separated and washed with sat. aq. $Na_2CO_3$ (3 × 10 mL), 5% aq. $H_2SO_4$ (3 × 10 mL), dried over $Na_2SO_4$, filtered, and concentrated *in vacuo* to give compound **1v** as a yellowish oil (185 mg, 89% yield).

Compound **1t** (3-hydroxy-3-methylbutyl 4-bromobenzoate)

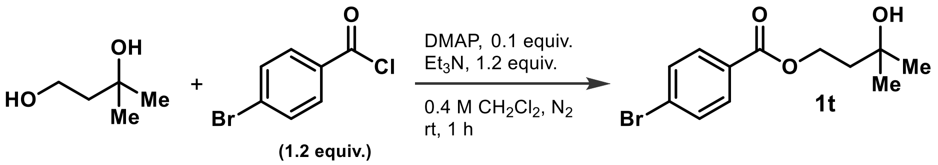


(4-bromo)benzoylchloride (526.7 mg, 2.4 mmol, 1.2 equiv.) and DMAP (22.4 mg, 0.2 mmol, 0.1 equiv.) were dried *in vacuo*, then 3-methyl-1,3-butanediol (213 µL, 2 mmol, 1 equiv.) was added, dissolved in dry $CH_2Cl_2$ (5 mL), $Et_3N$ (335 µL, 2.4 mmol, 1.2 equiv.) was added and stirred for 1 h. $CH_2Cl_2$ (20 mL) and sat. aq. $Na_2CO_3$ (10 mL) were added, organic layer was separated and washed with sat. aq. $Na_2CO_3$ (3 × 10 mL), 5% aq. $H_2SO_4$ (3 × 10 mL), dried over $Na_2SO_4$, filtered, and concentrated *in vacuo*. The residue was purified by silica gel column chromatography $CH_2Cl_2$/MeOH 10:1 to give compound **1t** as a yellow oil (561 mg, 98% yield).

Compound **1d** (2-(2-hydroxy-2,5,5,8a-tetramethyldecahydronaphthalen-1-yl)ethyl benzoate)

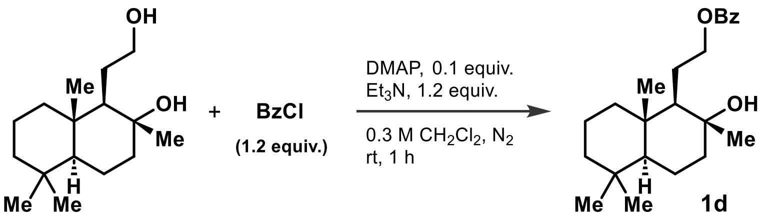


Sclareol glycol (381.6 mg, 1.5 mmol) and DMAP (18.3 mg, 0.15 mmol, 0.1 equiv.) were dried *in vacuo*, dissolved in dry $CH_2Cl_2$ (5 mL), $Et_3N$ (251 µL, 1.8 mmol, 1.2 equiv.) and benzoyl chloride (210 µL, 1.8 mL, 1.2 equiv.) were added sequentially and the reaction mixture was stirred for 1 h at r.t. $CH_2Cl_2$ (20 mL) and $Na_2CO_3$ sat (10 mL) were added, organic layer was separated and washed with sat. aq. $Na_2CO_3$ (3 × 10 mL), dried over $Na_2SO_4$, filtered and concentrated *in vacuo*. The residue was purified by silica gel column chromatography petroleum ether/EtOAc 2:1 to give compound **1d** as a colorless oil (538 mg, quantitative yield).

Compound **1w** (1-(2:3,4:6-diacetone β-D-glucopyranosyloxy) 3-hydroxy-3-methylbutane)

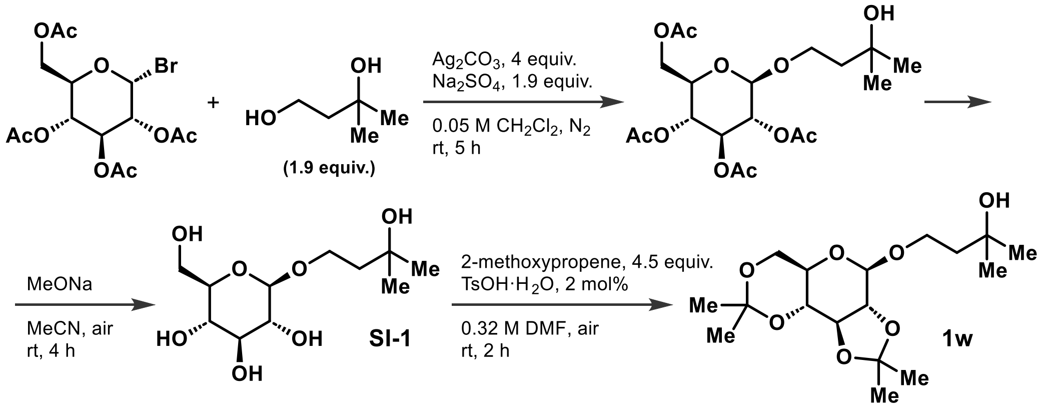


To a solution of the glucopyranosyl bromide (870 mg, 2.11 mmol, 1 equiv.) in dry $CH_2Cl_2$ (40 mL), anhydrous $Na_2SO_4$ (568 mg, 4 mmol, 1.9 equiv.) and 3-methyl-1,3-butanediol (0.426 mL, 4 mmol, 1.9 equiv.) were added and stirred at r.t. for 15 min under $N_2$. $Ag_2CO_3$ (1.103 mg, 4 equiv) was added and stirring continued for 5 h. The reaction mixture was filtered through silica gel plug, washed with $CH_2Cl_2$/MeOH 10:1 mixture (150 mL), filtrate was collected and concentrated *in vacuo*. The residue was purified on silica gel column chromatography petroleum ether/EtOAc 3:1 → 1:1 to give the desired glucoside as a colorless oil (580 mg) which was used directly in the next step.

The residue was dissolved in MeOH (20 mL), MeONa (10 mg) was added and stirred for 4 h at r.t. The reaction mixture was concentrated *in vacuo* and the residue was purified by silica gel column chromatography $CH_2Cl_2$/MeOH 5:1 → 3:1 to give glucoside **SI-1** as a colorless oil (213 mg, 38% yield over 2 steps).

To the solution of glucoside **SI-1** (213 mg, 0.8 mmol, 1 equiv.) and 4-toluenesulphonic acid monohydrate (3 mg, 0.016 mmol, 0.02 equiv.) in DMF (2.5 mL) 2-methoxypropene (345 µL, 3.6 mmol, 4.5 equiv.) was added, and the reaction mixture was stirred at r.t. for 2 h. The reaction mixture was concentrated *in vacuo* and DMF was co-evaporated with toluene (3 × 10 mL). The residue was purified by silica gel column

chromatography petroleum ether/EtOAc 5:1 → 2:1 to give diacetonide **1w** as colorless powder (159 mg, 57% yield).

## Compound **1l** ((3-(hydroxymethyl)-1H-indol-1-yl)(phenyl)methanone)

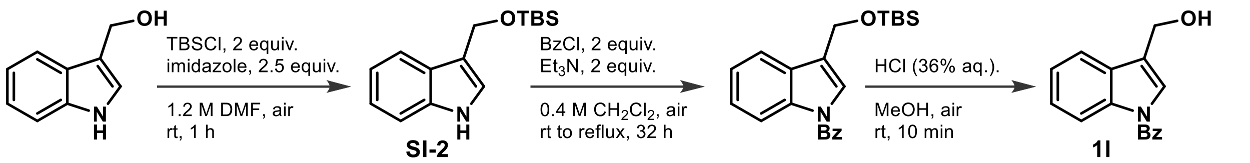


To a solution of indole-3-carbinol (883 mg, 6 mmol, 1 equiv.) and imidazole (1.021 g, 15 mmol, 2.5 equiv.) in DMF (5 mL) *tert*-butyldimethylsilyl chloride (1.808 g, 12 mmol, 2 equiv.) was added in one portion. The reaction was stirred at r.t. for 1 h, water (30 mL) was added and extracted with EtOAc (3 × 30 mL). The organic layer was washed with brine (2 × 30 mL), dried over anhydrous $Na_2SO_4$ and concentrated *in vacuo*. The residue was purified by silica gel column chromatography petroleum ether/EtOAc 5:1 → 2:1 to give compound **SI-2** as a colorless solid (1.224 g, 78% yield).

To a solution of intermediate compound **SI-2** (522 mg, 2 mmol, 1 equiv.) and $Et_3N$ (560 µL, 4 mmol, 2 equiv.) in $CH_2Cl_2$ (5 mL) benzoyl chloride (465 µL, 4 mmol, 2 equiv.) was added. The reaction mixture was stirred at r.t. for 16 h and then refluxed for 16 h. Water (20 mL) was added, extracted with $CH_2Cl_2$ (3 × 20 mL), organic extracts were combined and washed with sat. aq. $Na_2CO_3$ (2 × 30 mL). The organic extract was dried over $Na_2SO_4$, filtered and concentrated *in vacuo* to give the desired benzoylated intermediate product. The residue was dissolved in MeOH (6.5mL) and 36% aq. HCl (450 µL) was added. The reaction mixture was stirred at r.t. for 10 min until bright pink color, then sat. aq. $Na_2CO_3$ (20 mL) was added. The resulted aqueous solution was extracted with EtOAc (3 × 20 mL), the organic layers were combined, dried over $Na_2SO_4$ and concentrated *in vacuo*. The residue was purified by silica gel column chromatography petroleum ether/EtOAc 2:1 → 1:1 to give compound **1l** as a colorless oil (250 mg, 50% yield over two steps).

## Compounds **2i**, **2j**, **2p** (*tert*-alcohols from ketones)

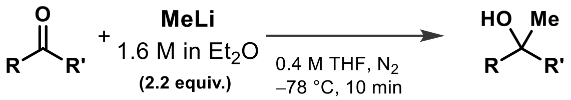


**General procedure A**. A ketone (4 mmol, 1.0 equiv.), was dissolved in dry THF (10 mL) and was cooled on dry $CO_2$-acetone bath to −78 °C. Thereafter, methyl lithium 1.6 M solution in $Et_2O$ (2.75 mL, 4.4 mmol, 2.2 equiv.) was added dropwise and the reaction mixture was stirred until warmed to r.t. The reaction mixture was quenched by sat. aq. $NH_4Cl$ (40 mL), the organic layer was separated, and the water layer was extracted with EtOAc (3 × 25 mL). The combined organic phases were dried over $Na_2SO_4$, filtered, and concentrated *in vacuo* and the residue was purified by silica gel column chromatography.

Compounds **SI-3**, **SI-4**, **SI-5** (homoallylic alcohols)

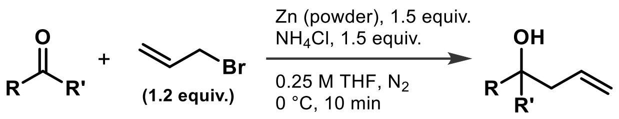


**General procedure B**. A suspension of ketone (2 mmol, 1.0 equiv.), zinc powder (196 mg, 3 mmol, 1.5 equiv.), and ammonium acetate (231 mg, 3 mmol, 1.5 equiv.) in dry THF (8 mL) was cooled with an ice bath. Thereafter, allyl bromide (260 µL, 3 mmol, 1.5 equiv.) was added dropwise and the reaction mixture was stirred for 10 min. The reaction mixture was quenched by sat. aq. $NaHCO_3$ (20 mL), ice bath was removed, and the reaction allowed to warm to r.t. Water (20 mL) was added and extracted with EtOAc (3 × 40 mL). The combined organic phases were dried over $Na_2SO_4$, filtered, and concentrated *in vacuo*.

Compounds **2a**–**2w**, **6a**–**6d** (alkyl methyl oxalates)

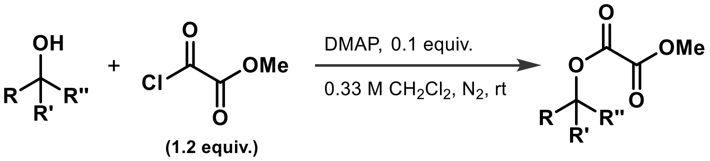


**General procedure C**. An alcohol (1 mmol) and DMAP (12.5 mg, 0.1 mmol, 0.1 equiv.) were dissolved in dry $CH_2Cl_2$ (3 mL) under $N_2$. $Et_3N$ (0.170 mL, 1.2 equiv.) and methyl chlorooxoacetate (110 µL, 1.2 equiv.) were added sequentially and the reaction mixture was stirred at r.t. for 5 min to 4 h until full conversion of the starting material. The reaction mixture was concentrated *in vacuo* and the residue was purified by silica gel column chromatography.

Compounds **3a-Na**, **3x-Na**, **3y-Na** (alkyl oxalate salts)

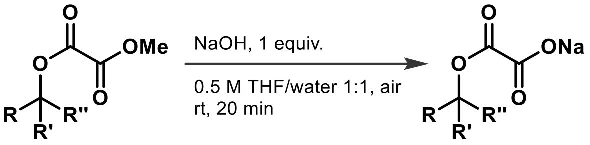


**General procedure D**. Methyloxalate (1.5 mmol) was dissolved in THF (1.5 mL), then aq. 1M NaOH (1.5 mL) was added dropwise. The reaction mixture was stirred for 20 min, water (20 mL) was added, extracted with EtOAc (2 × 20 mL). Water layers were combined and evaporated in vacuo to give white powder.

### 4.2 *General procedure for the photoredox-mediated synthesis of unnatural amino acids*

Compounds **5a**–**5w**, **7a–7d** (unnatural α-amino acids)

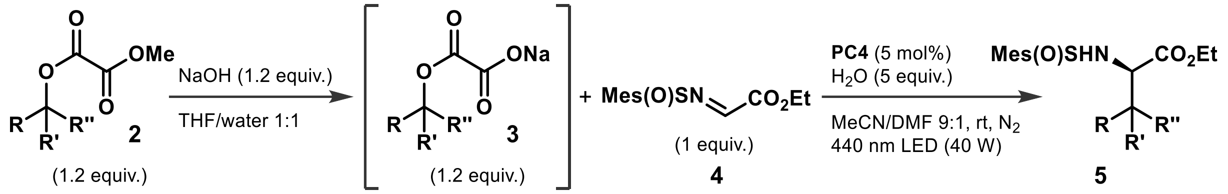


Methyloxalate (0.3 mmol, 1.2 equiv.) was placed in the 8 mL reaction vial, dissolved in tetrahydrofuran (0.36 mL), aq. 1M NaOH (0.36 mL, 0.36 mmol, 1.2 equiv.) was added dropwise and stirred at room temperature for 30 min. The reaction mixture was concentrated and dried *in vacuo* overnight. The *N*-sulfinyl imine **4** (80.2 mg, 0.30 mmol, 1 equiv.), $[Ir(dFCF_3ppy)_2(5,5'\text{-}dCF_3bpy)]PF_6$ photocatalyst (**PC4**, 10.4 mg, 3 mol%), were added to the resultant sodium salt, the reaction vial was equipped with a stirring bar and a septum, the solids were evacuated and back-filled with $N_2$ three times followed by addition of acetonitrile (5.4 mL), dimethylformamide (0.6 mL), and water (27 µL, 0.015 mmol, 0.05 equiv.). The vial was sealed with parafilm and sonicated for 15 min. The vial was placed in a holder ca. 2 cm from the light source (440 nm LED) and stirred (1200 rpm) under illumination with a fan cooling for 2–4 h. After the reaction was complete, it was transferred to round-bottom flask, concentrated *in vacuo* and purified by silica gel column chromatography.

### 4.3 *General procedure for N-sulfinyl amide deprotection*

Compounds **8a**, **8d**, **8f**, **8i** (*N*-deprotected unnatural α-amino acids)

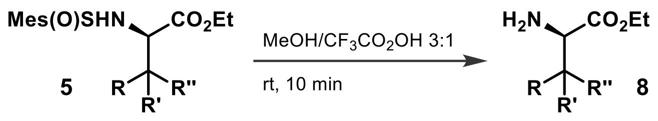


*N*-sulfinyl amide **5** (0.1 mmol) was placed in a 5 mL round-bottom flask, dissolved in 1 mL MeOH and a mixture of $CF_3CO_2H$ and MeOH (1:1 v/v, 1 mL) was added. The reaction mixture was stirred at room temperature under nitrogen for 10 min and the solvent was removed under nitrogen flow. The residue was purified by column chromatography with a gradient $CH_2Cl_2$/MeOH 50:1 → 10:1 as eluent and dried in vacuo overnight, resulting in the deprotected amino acid **8** or as its salt with trifluoroacetic acid.

### 4.4 *Unsuccessful substrates*

During investigation of the substrate scope, several alcohol substrates proved non-compatible with the disclosed protocols. For various substrates, the lack of desired reactivity was observed at the stage of methyl oxalate ester installation, methyl ester hydrolysis, or the photoreaction (Fig. S4).

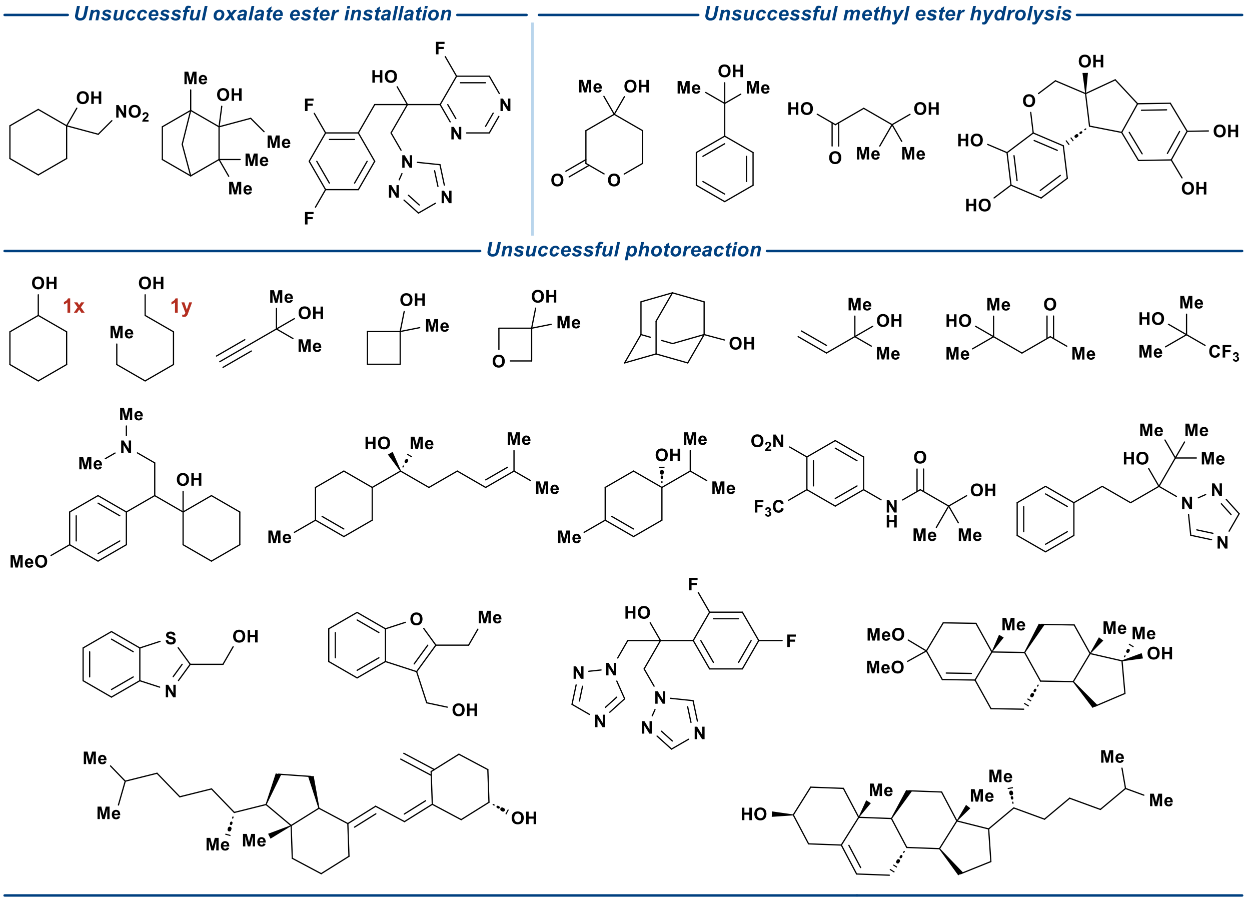


**Fig. S4.** Unsuccessful substrates.

## 5. ***Analytical data***

Compound **SI-1** (3-hydroxy-3-methylbutan-1-ol β-D-glucopyranoside)

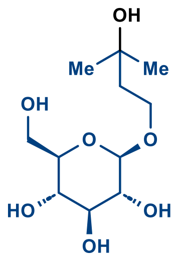


Synthesized according to the procedure described in **Section 4.1**.

**$^{1}$H NMR** (500 MHz, MeOD) δ 4.26 (d, *J* = 7.8 Hz, 1H), 4.07 (dt, *J* = 9.8, 7.1 Hz, 1H), 3.87 (d, *J* = 11.5 Hz, 1H), 3.76–3.62 (m, 2H), 3.30–3.21 (m, 2H), 3.16 (dd, *J* = 9.1, 7.8 Hz, 1H), 1.83 (td, *J* = 7.1, 2.5 Hz, 2H), 1.23 (s, 6H).

**$^{13}$C NMR** (126 MHz, MeOD) δ 104.4, 78.1, 78.0, 75.1, 71.7, 70.8, 67.5, 62.8, 43.4, 29.7, 29.6.

***R*$_f$** = 0.33 ($CH_2Cl_2$/MeOH 4:1, brown color upon treatment with 5% methanolic $H_3PO_4$ and heating)

Compound **SI-2** (3-(((*tert*-butyldimethylsilyl)oxy)methyl)-1*H*-indole)

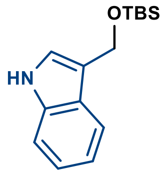


Synthesized according to the procedure described in **Section 4.1**.

**$^{1}$H NMR** (500 MHz, $CDCl_3$) δ 7.99 (s, 1H), 7.69 (d, *J* = 7.8 Hz, 1H), 7.34 (d, *J* = 8.0 Hz, 1H), 7.21 (t, *J* = 7.6 Hz, 1H), 7.18–6.98 (m, 2H), 4.96 (s, 2H), 0.96 (s, 9H), 0.13 (s, 3H).

**$^{13}$C NMR** (126 MHz, $CDCl_3$) δ 136.5, 126.5, 122.1, 119.5, 119.3, 116.7, 111.1, 58.3, 26.1, 18.5, −5.1.

***R*$_f$** = 0.52 (petroleum ether/EtOAc 5:1)

The spectroscopic data is in agreement with the literature.[10]

Compound **SI-3** (*tert*-butyl (1*R*,3*r*,5*S*)-3-allyl-3-hydroxy-8-azabicyclo[3.2.1]octane-8-carboxylate)

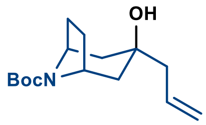


Synthesized according to the **General procedure B** described in **Section 4.1** on 2 mmol scale from *N*-Boc-nortropinone. The product is a colorless oil (539 mg, **quantitative yield**).

**$^{1}$H NMR** (500 MHz, $CDCl_3$) δ 5.80 (ddt, *J* = 17.5, 10.1, 7.5 Hz, 1H), 5.20 (dd, *J* = 10.2, 2.0 Hz, 1H), 5.16–5.10 (dd, *J* = 17.5, 2.0 Hz, 1H), 4.24 (br.s, 1H), 2.15–2.07 (m, 4H), 2.06–1.95 (m, 1H), 1.93–1.81 (m, 3H), 1.61–1.56 (m, 2H), 1.46 (s, 9H).

**$^{13}$C NMR** (126 MHz, $CDCl_3$) δ 153.5, 132.6, 120.1, 79.1, 70.5, 53.2, 52.5, 50.9, 42.7, 28.5, 28.1, 27.4.

***R*$_f$** = 0.47 (petroleum ether/EtOAc 2:1)

**HRMS** (ESI): calcd for $C_{15}H_{25}NaNO_3$ $[M + Na]^+$: 290.1726, found: 290.1727.

The spectroscopic data is in agreement with the literature.[11]

Compound **SI-4** ((1*r*,3*r*,5*r*,7*r*)-2-allyladamantan-2-ol)

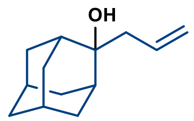


Synthesized according to the **General procedure B** described in **Section 4.1** on 2 mmol scale from 2-adamantanone. The product is a colorless amorphous solid (384 mg, **quantitative yield**).

**$^1$H NMR** (500 MHz, $CDCl_3$) δ 5.90 (ddt, *J* = 17.6, 10.2, 7.5 Hz, 1H), 5.22–5.11 (m, 2H), 2.45 (d, *J* = 7.5 Hz, 2H), 2.27–2.16 (m, 2H), 1.94–1.84 (m, 2H), 1.82 (dt, *J* = 11.6, 3.0 Hz, 2H), 1.76–1.62 (m, 7H), 1.54 (ddd, *J* = 12.6, 3.1, 1.7 Hz, 2H).

**$^{13}$C NMR** (126 MHz, $CDCl_3$) δ 133.7, 118.8, 74.5, 42.7, 38.4, 37.1 (2 × $CH_2$), 34.4 (2 × $CH_2$), 32.9 (2 × $CH_2$), 27.4, 27.3.

***R*$_f$** = 0.43 (petroleum ether/EtOAc 10:1)

**HRMS** (EI): calcd for $C_{13}H_{18}$ $[M - OH]^+$: 175.1482, found: 175.1480.

The spectroscopic data is in agreement with the literature.[11]

Compound **SI-5** (2-allyl-2,3-dihydro-1*H*-inden-2-ol)

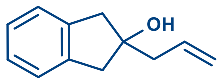


Synthesized according to the **General procedure B** described in **Section 4.1** on 2 mmol scale from 2-indenone. The crude product was purified by column chromatography using *n*-hexane/EtOAc 20:1 ⟶ 5:1 as eluent. The product is a colorless amorphous solid (263 mg, **76% yield**).

**$^1$H NMR** (500 MHz, $CDCl_3$) δ 7.25–7.12 (m, 4H), 6.04–5.89 (m, 1H), 5.27–5.16 (m, 2H), 3.09 (d, *J* = 16.2 Hz, 2H), 2.96 (d, *J* = 16.2 Hz, 2H), 2.52 (dt, *J* = 7.4, 1.2 Hz, 2H), 1.90 (s, 1H).

**$^{13}$C NMR** (126 MHz, $CDCl_3$) δ 141.1, 133.9, 126.6, 125.0, 119.1, 81.5, 46.5, 45.0.

***R*$_f$** = 0.44 (petroleum ether/EtOAc 4:1, brown color upon treatment with 5% $H_3PO_4$ in MeOH and heating)

**HRMS** (ESI): calcd for $C_{12}H_{11}$ $[M - OH]^+$: 155.0861, found: 155.0854.

The spectroscopic data is in agreement with the literature.[12]

Compound **1d** (2-((1*R*,2*R*,4a*S*,8a*S*)-2-hydroxy-2,5,5,8a-tetramethyldecahydronaphthalen-1-yl)ethyl benzoate)

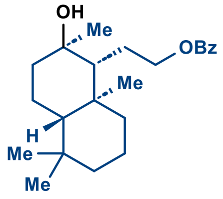


Synthesized according to the procedure described in **Section 4.1**.

**$^1$H NMR** (500 MHz, $CDCl_3$) δ 8.13–7.90 (m, 2H), 7.61–7.49 (m, 1H), 7.44 (dd~t, *J* = 7.8 Hz, 2H), 4.38 (qdd, *J* = 10.5, 8.5, 6.5 Hz, 2H), 1.96–1.84 (m, 2H), 1.84–1.76 (m, 1H), 1.76–1.71 (m, 1H), 1.68 (dq, *J* = 13.8, 3.3 Hz, 1H), 1.60 (dt, *J* = 13.7, 3.5 Hz, 1H), 1.49–1.34 (m, 3H), 1.35–1.22 (m, 1H), 1.22–1.18 (m, 3H), 1.20–1.07 (m, 2H), 0.96 (ddd, *J* = 14.9, 12.7, 3.2 Hz, 2H), 0.87 (s, 3H), 0.82 (s, 3H), 0.80 (s, 3H).

**$^{13}$C NMR** (126 MHz, $CDCl_3$) δ 166.8, 132.8, 130.5, 129.5, 128.4, 73.7, 67.1, 58.1, 56.1, 44.5, 41.9, 39.7, 38.8, 33.4, 33.3, 24.6, 24.0, 21.5, 20.5, 18.4, 15.4.

***R*$_f$** = 0.59 (petroleum ether/EtOAc 2:1)

**HRMS** (ESI): calcd for $C_{23}H_{34}NaO_3$ $[M + Na]^+$: 381.2400, found: 381.2401.

## Compound **1i** (*tert*-butyl 6-hydroxy-6-methyl-2-azaspiro[3.3]heptane-2-carboxylate)

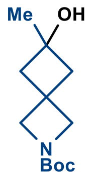


Synthesized according to the **General procedure A** described in **Section 4.1** on 4 mmol scale from *tert*-butyl 6-oxo-2-azaspiro[3.3]heptane-2-carboxylate. The crude product was purified by column chromatography using *n*-hexane/EtOAc 5:1 → 1:1 as eluent. The product is a white solid (662 mg, **73% yield**).

**$^1$H NMR** (500 MHz, $CDCl_3$) δ 3.92 (s, 2H), 3.89 (s, 2H), 2.20–2.31 (m, 4H), 1.43 (s, 9H), 1.32 (s, 3H).

**$^{13}$C NMR** (126 MHz, $CDCl_3$) δ 156.1, 79.3, 68.8, 48.3, 28.5, 28.4, 28.0.

***R*$_f$** = 0.24 (hexanes/EtOAc 1:1)

**HRMS** (ESI): calcd for $C_{12}H_{21}NaNO_3$ $[M + Na]^+$: 250.1423, found: 250.1423.

## Compound **1j** (*tert*-butyl 2-hydroxy-2-methyl-7-azaspiro[3.5]nonane-7-carboxylate)

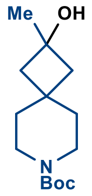


Synthesized according to the **General procedure A** described in **Section 4.1** on 4 mmol scale from 2-oxo-7-azaspiro[3.5]nonane-7-carboxylate *tert*-butyl ester. The crude product was purified by column chromatography using *n*-hexane/EtOAc 5:1 → 2:1 as eluent. The product is a white solid (470 mg, **46% yield**).

**$^1$H NMR** (500 MHz, $CDCl_3$) δ 3.35–3.23 (m, 4H), 2.00–1.85 (m, 4H), 1.64–1.59 (m, 2H), 1.52–1.47 (m, 2H), 1.44 (s, 9H), 1.39 (s, 3H).

**$^{13}$C NMR** (126 MHz, $CDCl_3$) δ 155.0, 79.3, 68.9, 46.9, 40.8, 40.6, 39.0, 37.3, 31.0, 28.5, 28.2.

***R*$_f$** = 0.43 (hexanes/EtOAc 1:1)

**HRMS** (ESI): calcd for $C_{14}H_{25}NaNO_3$ $[M + Na]^+$: 278.1721, found: 278.1712.

## Compound **1l** ((3-(hydroxymethyl)-1*H*-indol-1-yl)(phenyl)methanone)

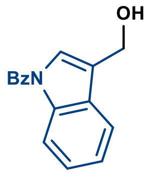


Synthesized according to the procedure described in **Section 4.1**.

**$^1$H NMR** (500 MHz, $CDCl_3$) δ 8.41 (d, *J* = 8.2 Hz, 1H), 7.78–7.71 (m, 2H), 7.69 (d, *J* = 7.6 Hz, 1H), 7.62 (tt, *J* = 6.8, 1.2 Hz, 2H), 7.57–7.51 (m, 2H), 7.42 (td, *J* = 8.3, 7.8, 1.2 Hz, 1H), 7.36 (td, *J* = 7.5, 1.1 Hz, 1H), 7.30 (s, 1H), 4.85 (s, 2H).

**$^{13}$C NMR** (126 MHz, $CDCl_3$) δ 168.6, 136.7, 134.5, 131.9, 129.5, 129.1, 128.6, 125.4, 125.2, 124.0, 121.7, 119.3, 116.7, 57.2.

$R_f$ = 0.67 (petroleum ether/EtOAc 1:1)

**HRMS** (ESI): calcd for $C_{16}H_{13}NO_2$ [M + Na]$^+$: 274.0839, found: 274.0838.

Compound **1p** (*tert*-butyl (S)-4,4-difluoro-2-(2-hydroxypropan-2-yl)pyrrolidine-1-carboxylate)

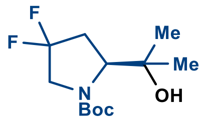


Synthesized according to the **General procedure A** described in **Section 4.1**, using MeMgBr instead of MeLi, on 2 mmol scale from 1-*tert*-butyl 2-methyl (2*S*)-4,4-difluoro-1,2-pyrrolidinedicarboxylate. The crude product was purified by column chromatography using *n*-hexane/EtOAc 5:1 ⟶ 2:1 as eluent. The product is a white solid (318 mg, **60% yield**).

**$^{1}$H NMR** (500 MHz, $CDCl_3$) δ 4.12 (t, *J* = 8.4 Hz, 1H), 4.07 – 3.92 (m, 1H), 3.49 (ddd, *J* = 24.6, 12.3, 5.9 Hz, 1H), 2.49 (dddt, *J* = 17.2, 13.9, 8.7, 2.5 Hz, 1H), 2.12 (br.s, 1H), 1.47 (s, 9H), 1.17 (s, 3H), 1.14 (s, 3H).

**$^{13}$C NMR** (126 MHz, $CDCl_3$) δ 156.8z, 81.8, 73.2 (br)z, 65.4 (br), 54.3 (t, *J* = 31.0 Hz), 36.9 (t, *J* = 24.7 Hz), 28.2, 27.2, 23.1 (br).

**$^{19}$F NMR** (377 MHz, $CDCl_3$) δ -97.9 (d, *J* = 230.3 Hz), -107.0 (d, *J* = 230.4 Hz).

$R_f$ = 0.41 (hexanes/EtOAc 3:1)

**HRMS** (ESI): calcd for $C_{12}H_{21}NaF2NO_3$ [M + Na]$^+$: 288.1392, found: 288.1394.

Compound **1r** (4-((*tert*-butyldimethylsilyl)oxy)-2-methylbutan-2-ol)

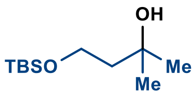


Synthesized according to the procedure described in **Section 4.1**.

**$^{1}$H NMR** (500 MHz, $CDCl_3$) δ 3.91 (t, *J* = 5.7 Hz, 2H, $OCH_2$), 3.83 (s, 1H, OH), 1.70 (t, *J* = 5.7 Hz, 2H, $CH_2$), 1.24 (s, 6H, $C(CH_3)_2$), 0.90 (s, 9H $C(CH_3)_3$), 0.09 (s, 6H, $Si(CH_3)_2$).

**$^{13}$C NMR** (126 MHz, $CDCl_3$) δ 70.8, 61.0, 42.9, 29.2, 25.8, 18.0, −5.6.

$R_f$ = 0.49 (petroleum ether/EtOAc 6:1, yellow color upon treatment with $KMnO_4$ stain and heating)

The spectroscopic data is in agreement with the literature.[13]

Compound **1s** (4-(benzoyloxy)-2-methylbutan-2-ol)

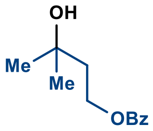


Synthesized according to the procedure described in **Section 4.1**.

**H NMR** (500 MHz, $CDCl_3$) δ 8.13–8.00 (m, 2H), 7.56 (tt, *J* = 7.8, 1.3 Hz, 1H), 7.44 (dd, *J* = 8.4, 7.2 Hz, 2H), 4.51 (t, *J* = 6.8 Hz, 2H), 1.99 (t, *J* = 6.8 Hz, 2H), 1.33 (s, 6H).

**$^{13}$C NMR** (126 MHz, $CDCl_3$) δ 166.6, 132.9, 130.2, 129.5, 128.4, 70.1, 61.9, 41.7, 29.8.

$R_f$ = 0.30 (petroleum ether/EtOAc 4:1)

The spectroscopic data is in agreement with the literature.[14]

## Compound **1t** (3-hydroxy-3-methylbutyl 4-bromobenzoate)

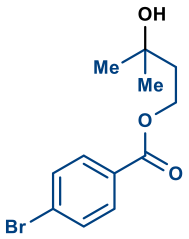


Synthesized according to the procedure described in **Section 4.1**.

**$^1$H NMR** (500 MHz, $CDCl_3$) δ 7.93–7.84 (m, 2H), 7.64–7.53 (m, 2H), 4.50 (t, *J* = 6.8 Hz, 2H), 1.98 (t, *J* = 6.8 Hz, 2H), 1.32 (s, 6H).

**$^{13}$C NMR** (126 MHz, $CDCl_3$) δ 165.9, 131.7, 131.0, 129.1, 128.1, 70.0, 62.2, 41.7, 29.8.

$R_f$ = 0.55 ($CH_2Cl_2$/MeOH 20:1)

The spectroscopic data is in agreement with the literature.[15]

## Compound **1u** (4-(nicotynoyloxy)-2-methylbutan-2-ol)

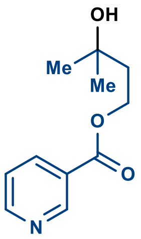


Synthesized according to the procedure described in **Section 4.1**.

**$^1$H NMR** (500 MHz, $CDCl_3$) δ 9.22 (s, 1H), 8.86–8.72 (m, 1H), 8.29 (dt, *J* = 8.0, 2.0 Hz, 1H), 7.40 (dd, *J* = 7.8, 4.9 Hz, 1H), 4.54 (t, *J* = 6.9 Hz, 2H), 2.00 (t, *J* = 6.9 Hz, 2H), 1.33 (s, 6H).

**$^{13}$C NMR** (126 MHz, $CDCl_3$) δ 165.3, 153.4, 150.9, 137.0, 126.2, 123.3, 70.0, 62.3, 41.7, 29.9.

$R_f$ = 0.43 ($CH_2Cl_2$/MeOH 5:1)

**HRMS** (ESI): calcd for $C_{11}H_{16}NO_3$ $[M + H]^+$: 210.1125, found: 210.1124.

## Compound **1v** (4-(2-thiophenecarboxy)-2-methylbutan-2-ol)

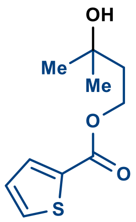


Synthesized according to the procedure described in **Section 4.1**.

**$^1$H NMR** (500 MHz, $CDCl_3$) δ 7.79 (dd, *J* = 3.7, 1.3 Hz, 1H), 7.55 (dd, *J* = 5.0, 1.3 Hz, 1H), 7.10 (dd, *J* = 5.0, 3.7 Hz, 1H), 4.48 (t, *J* = 6.7 Hz, 2H), 1.96 (t, *J* = 6.7 Hz, 2H), 1.32 (s, 6H).

**$^{13}$C NMR** (126 MHz, $CDCl_3$) δ 162.2, 133.8, 133.4, 132.3, 127.8, 70.1, 62.1, 41.7, 29.8.

$R_f$ = 0.49 ($CH_2Cl_2$/MeOH 5:1)

Compound **1w** (1-(2:3,4:6-diacetone β-D-glucopyranosyloxy) 3-hydroxy-3-methylbutane)

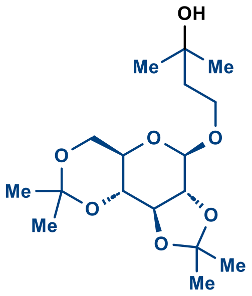


Synthesized according to the procedure described in **Section 4.1**.

**$^{1}$H NMR** (500 MHz, $CDCl_3$) δ 4.71 (d, *J* = 7.9 Hz, 1H), 4.11 (ddd, *J* = 9.7, 7.3, 6.1 Hz, 1H), 3.96 (dd, *J* = 10.8, 5.3 Hz, 1H), 3.94–3.85 (m, 2H), 3.82 (dt, *J* = 9.8, 6.3 Hz, 1H), 3.64 (t, *J* = 9.3 Hz, 1H), 3.38 (dd, *J* = 9.0, 7.9 Hz, 1H), 3.27 (ddd, *J* = 10.2, 8.8, 5.3 Hz, 1H), 1.99–1.74 (m, 2H), 1.54 (s, 3H), 1.46 (s, 3H), 1.45 (s, 3H), 1.44 (s, 3H), 1.25 (s, 6H).

**$^{13}$C NMR** (126 MHz, $CDCl_3$) δ 112.2, 102.2, 99.8, 77.8, 77.5, 72.7, 70.2, 69.8, 66.9, 62.2, 41.8, 29.7, 29.4, 28.9, 26.7, 26.4, 19.1.

***R*$_f$** = 0.38 (petroleum ether/EtOAc 4:1)

**HRMS** (ESI): calcd for $C_{17}H_{30}NaO_7$ $[M + Na]^+$: 369.1884, found: 369.1894.

Compound **2a** (methyl (1-methylcyclohexyl) oxalate)

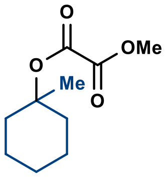


Synthesized according to the **General procedure C** described in **Section 4.1** on 5 mmol scale from 1-methyl-cyclohexan-1-ol with 5 min reaction time. The crude product was purified by column chromatography using petroleum ether/EtOAc 10:1 → 5:1 as eluent. The product is a colorless oil (940 mg, **94% yield**).

**$^{1}$H NMR** (500 MHz, $CDCl_3$) δ 3.83 (s, 1H), 2.24–2.08 (m, 2H), 1.52 (s, 3H), 1.60–1.39 (m, 7H), 1.33–1.19 (m, 1H).

**$^{13}$C NMR** (126 MHz, $CDCl_3$) δ 159.0, 156.6, 86.7, 53.1, 36.2, 25.1, 24.9, 21.9.

***R*$_f$** = 0.50 (petroleum ether/EtOAc 5:1)

**HRMS** (ESI): calcd for $C_{10}H_{16}NaO_4$ $[M + Na]^+$: 223.0941, found: 223.0943.

The spectroscopic data is in agreement with the literature.[16]

Compound **2b** (methyl (1-methylcyclopentyl) oxalate)

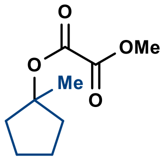


Synthesized according to the **General procedure C** described in **Section 4.1** on 2 mmol scale from 1-methyl-cyclopentan-1-ol with 2 h reaction time. The crude product was purified by column chromatography using hexane/EtOAc 7:1 as eluent. The product is a colorless oil (542 mg, **97% yield**).

**$^{1}$H NMR** (500 MHz, $CDCl_3$) δ 3.86 (s, 6H), 2.28–2.12 (m, 2H), 1.85–1.71 (m, 4H), 1.70–1.64 (m, 2H), 1.63 (s, 3H).

**$^{13}$C NMR** (126 MHz, $CDCl_3$) δ 159.0, 157.0, 94.3, 53.3, 38.8, 23.9, 23.7.

***R*$_f$** = 0.55 (hexane/EtOAc 7:1)

**HRMS** (ESI): calcd for $C_9H_{14}NaO_4$ $[M + Na]^+$: 209.0784, found: 209.0784.

The spectroscopic data is in agreement with the literature.[16]

Compound **2c** (methyl (2-methyl-2,3-dihydro-1*H*-inden-2-yl) oxalate)

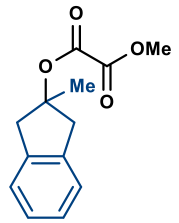


Synthesized according to the **General procedure C** described in **Section 4.1** on 2.1 mmol scale from 2-methyl-2,3-dihydro-1*H*-inden-2-ol with 20 min reaction time. The crude product was purified by column chromatography using petroleum ether/EtOAc 10:1 ⟶ 6:1 as eluent. The product is a white solid (446 mg, **91% yield**).

**$^1$H NMR** (500 MHz, $CDCl_3$) δ 7.21–7.16 (m, 4H), 3.87 (s, 3H), 3.51 (d, *J* = 16.6 Hz, 2H), 3.25 (d, *J* = 16.5 Hz, 2H), 1.74 (s, 3H).

**$^{13}$C NMR** (126 MHz, $CDCl_3$) δ 158.6, 157.0, 139.7, 126.9, 124.6, 92.1, 53.4, 45.7, 24.1.

***R*$_f$** = 0.49 (petroleum ether/EtOAc 5:1)

**HRMS** (ESI): calcd for $C_9H_{16}NaO_4$ $[M + Na]^+$: 257.0784, found: 257.0785.

The spectroscopic data is in agreement with the literature.[17]

Compound **2d** ((1*R*,2*R*,4a*S*,8a*S*)-1-(2-(benzoyloxy)ethyl)-2,5,5,8a-tetramethyldecahydronaphthalen-2-yl methyl oxalate)

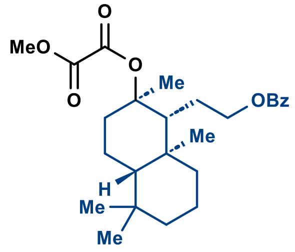


Synthesized according to the **General procedure C** described in **Section 4.1** on 1.4 mmol scale from benzoylated sclareol glycol **1d** with 2 h reaction time. The product is a colorless oil (444 mg, **70% yield**).

**$^1$H NMR** (500 MHz, $CDCl_3$) δ 8.18–7.94 (m, 2H), 7.63–7.49 (m, 1H), 7.44 (dd~t, *J* = 7.7 Hz, 2H), 4.51 (dt, *J* = 10.4, 7.7 Hz, 1H), 4.38 (dt, *J* = 10.4, 8.1 Hz, 1H), 3.83 (s, 3H), 2.84 (dt, *J* = 12.3, 3.9 Hz, 1H), 1.88 (td, *J* = 8.1, 4.4 Hz, 2H), 1.81 (d, *J* = 12.8 Hz, 1H), 1.73 (td, *J* = 13.1, 12.4, 4.4 Hz, 2H), 1.64 (s, 3H), 1.67–1.56 (m, 2H), 1.46 (dt, *J* = 14.4, 3.7 Hz, 1H), 1.39 (d, *J* = 13.3 Hz, 1H), 1.37–1.23 (m, 1H), 1.16 (td, *J* = 13.5, 4.3 Hz, 1H), 1.07–0.96 (m, 2H), 0.88 (s, 3H), 0.87 (s, 3H), 0.80 (s, 3H).

**$^{13}$C NMR** (126 MHz, $CDCl_3$) δ 166.5, 158.8, 155.9, 132.8, 130.5, 129.5, 128.3, 92.2, 66.1, 55.6, 55.3, 53.3, 41.7, 39.4, 39.2, 38.7, 33.3, 33.2, 25.0, 21.4, 19.90, 19.92, 18.3, 15.6.

***R*$_f$** = 0.54 (petroleum ether/EtOAc 5:1)

**HRMS** (ESI): calcd for $C_{26}H_{36}NaO_6$ $[M + Na]^+$: 467.2404, found: 467.2405.

The spectroscopic data is in agreement with the literature.[18]

Compound **2e** (methyl ((3*R*,3a*S*,6*R*,7*R*,8a*S*)-3,6,8,8-tetramethyloctahydro-1*H*-3a,7-methanoazulen-6-yl) oxalate)

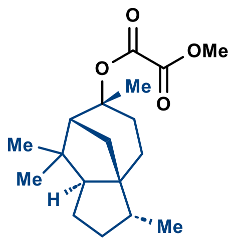


Synthesized according to the **General procedure C** described in **Section 4.1** on 5 mmol scale from cedrol with 1 h reaction time. The product is a white solid (1.411 g, **96% yield**).

**$^1$H NMR** (500 MHz, $CDCl_3$) δ 3.86 (s, 3H), 2.51–2.42 (m, 1H), 2.20–2.13 (m, 1H), 2.07 (td, *J* = 13.0, 6.8 Hz, 1H), 1.88 (dq, *J* = 12.1, 6.0 Hz, 1H), 1.83 (t, *J* = 8.0 Hz, 1H), 1.75–1.65 (m, 2H), 1.62 (d, *J* = 1.0 Hz, 3H), 1.56 (s, 3H), 1.56–1.51 (m, 1H), 1.51–1.47 (m, 1H), 1.46–1.34 (m, 3H), 1.33–1.24 (m, 1H), 1.17 (s, 3H), 0.99 (s, 3H), 0.84 (d, *J* = 7.1 Hz, 3H).

**$^{13}$C NMR** (126 MHz, $CDCl_3$) δ 159.0, 156.5, 91.1, 56.7, 56.6, 53.8, 53.1, 43.4, 41.1, 41.0, 36.8, 32.8, 31.2, 28.3, 26.9, 25.3, 25.2, 15.4.

***R*$_f$** = 0.74 (petroleum ether/EtOAc 3:1)

**HRMS** (ESI): calcd for $C_{18}H_{28}NaO_4$ $[M + Na]^+$: 331.1880, found: 331.1879.

The spectroscopic data is in agreement with the literature.[19]

Compound **2f** (1-benzoylcyclohexyl methyl oxalate)

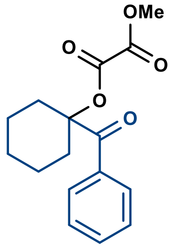


Synthesized according to the **General procedure C** described in **Section 4.1** on 1.5 mmol scale from (1-hydroxycyclohexyl)(phenyl)methanone with 1 h reaction time. The crude product was purified by column chromatography using petroleum ether/EtOAc 10:1 → 5:1 as eluent. The product is a colorless oil (433 mg, **99% yield**).

**$^1$H NMR** (500 MHz, $CDCl_3$) δ 8.00 (dd, *J* = 8.4, 1.2 Hz, 2H), 7.54–7.46 (m, 1H), 7.40 (dd, *J* = 8.4, 7.2 Hz, 2H), 3.85 (s, 3H), 2.48–2.43 (m, 2H), 2.02 (ddd, *J* = 15.3, 9.1, 3.0 Hz, 2H), 1.83–1.62 (m, 5H), 1.45–1.31 (m, 1H).

**$^{13}$C NMR** (126 MHz, $CDCl_3$) δ 198.3, 157.8, 156.2, 134.1, 132.8, 128.6, 128.5, 89.5, 53.5, 32.3, 24.9, 21.2.

***R*$_f$** = 0.46 (petroleum ether/EtOAc 5:1)

**HRMS** (ESI): calcd for $C_{16}H_{18}NaO_5$ $[M + Na]^+$: 313.1046, found: 313.1050.

The spectroscopic data is in agreement with the literature.[18]

## Compound **2g** (1-(*tert*-butoxycarbonyl)-4-methylpiperidin-4-yl methyl oxalate)

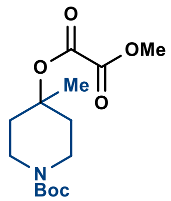


Synthesized according to the **General procedure C** described in **Section 4.1** on 2.45 mmol scale from tert-butyl 4-hydroxy-4-methylpiperidine-1-carboxylate with 1.5 h reaction time. The crude product was purified by column chromatography using hexane/EtOAc 3:1 as eluent. The product is a white solid (451 mg, **77% yield**).

**$^1$H NMR** (500 MHz, $CDCl_3$) δ 3.87 (s, 3H), 3.80 (br. s, 1H), 3.09 (t, *J* = 12.4 Hz, 2H), 2.41–2.20 (m, 2H), 1.70–1.55 (m, 3H), 1.58 (s, 3H), 1.45 (s, 9H).

**$^{13}$C NMR** (126 MHz, $CDCl_3$) δ 158.6, 156.6, 154.7, 84.0, 79.7, 53.4, 35.5, 28.4, 24.7.

***R*f** = 0.39 (hexane/EtOAc 3:1)

**HRMS** (ESI): calcd for $C_{14}H_{23}NaNO_6$ $[M + Na]^+$: 324.1418, found: 324.1418.

The spectroscopic data is in agreement with the literature.[20]

## Compound **2h** (methyl (4-methyltetrahydro-2*H*-thiopyran-4-yl) oxalate)

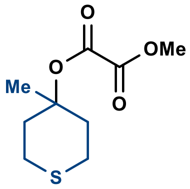


Synthesized according to the **General procedure C** described in **Section 4.1** on 2 mmol scale from 4-methyltetrahydro-2*H*-thiopyran-4-ol with 15 min reaction time. The crude product was purified by column chromatography using hexanes/EtOAc 5:1 → 3:1 as eluent. The product is a colorless oil (432 mg, **99% yield**).

**$^1$H NMR** (500 MHz, $CDCl_3$) δ 3.88 (s, 3H), 2.87 (t, *J* = 12.5 Hz, 2H), 2.58 (dt, *J* = 15.5, 3.2 Hz, 2H), 2.50–2.40 (m, 2H), 1.78 (ddd, *J* = 14.9, 12.0, 3.6 Hz, 2H), 1.57 (s, 3H).

**$^{13}$C NMR** (126 MHz, $CDCl_3$) δ 158.6, 156.4, 84.3, 53.4, 37.1, 25.9, 23.8.

***R*f** = 0.50 (hexane/EtOAc 4:1)

**HRMS** (ESI): calcd for $C_9H_{14}NaO_4S$ $[M + Na]^+$: 241.0525, found: 241.0525.

## Compound **2i** (2-(*tert*-butoxycarbonyl)-6-methyl-2-azaspiro[3.3]heptan-6-yl methyl oxalate)

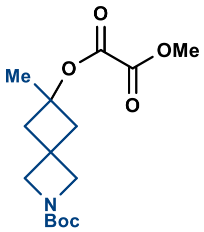


Synthesized according to the **General procedure C** described in **Section 4.1** on 4 mmol scale from *tert*-butyl 6-hydroxy-6-methyl-2-azaspiro[3.3]heptane-2-carboxylate **1i** with 1 h reaction time. The crude product was purified by column chromatography using hexanes/EtOAc 3:1 → 2:1 as eluent. The product is a colorless oil (1.0 g, **99% yield**).

**$^1$H NMR** (500 MHz, $CDCl_3$) δ 3.98 (s, 2H), 3.93 (s, 2H), 3.88 (s, 3H), 2.63 – 2.54 (m, 2H), 2.53 – 2.44 (m, 2H), 1.55 (s, 3H), 1.42 (s, 9H).

**$^{13}$C NMR** (126 MHz, $CDCl_3$) δ 158.3, 156.2, 156.1, 80.1, 78.7, 61.4, 60.1, 53.9, 53.5, 46.0, 30.3, 28.4, 23.6.

$R_f$ = 0.45 (hexane/EtOAc 2:1)

**HRMS** (ESI): calcd for $C_{15}H_{23}NaNO_6$ $[M + Na]^+$: 336.1426, found: 336.1425.

## Compound **2j** (2-(*tert*-butoxycarbonyl)-6-methyl-2-azaspiro[3.3]heptan-6-yl methyl oxalate)

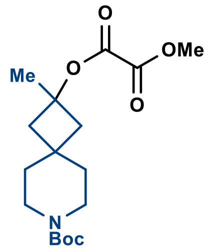


Synthesized according to the **General procedure C** described in **Section 4.1** on 1.5 mmol scale from *tert*-butyl 2-hydroxy-2-methyl-7-azaspiro[3.5]nonane-7-carboxylate **1j** with 1 h reaction time. The crude product was purified by column chromatography using hexanes/EtOAc 3:1 → 2:1 as eluent. The product is a colorless oil (395 mg, **78% yield**).

**$^1$H NMR** (500 MHz, $CDCl_3$) δ 3.89 (s, 3H), 3.31 (ddd, *J* = 11.5, 5.6, 3.5 Hz, 4H), 2.36–2.24 (m, 2H), 2.22–2.12 (m, 2H), 1.64 (s, 3H), 1.61–1.52 (m, 4H), 1.44 (s, 9H).

**$^{13}$C NMR** (126 MHz, $CDCl_3$) δ 158.5, 156.3, 154.8, 80.4, 79.4, 53.5, 44.7, 40.7, 40.5, 38.9, 36.8, 30.2, 28.4, 26.1.

$R_f$ = 0.59 (hexane/EtOAc 2:1)

**HRMS** (ESI): calcd for $C_{17}H_{27}NaNO_6$ $[M + Na]^+$: 364.1741, found: 364.1749.

## Compound **2k** ((1*R*,2*R*,5*R*)-(+)-2-hydroxy-3-pinanone methyl oxalate)

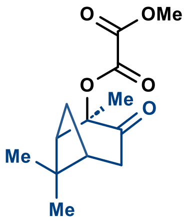


Synthesized according to the **General procedure C** described in **Section 4.1** on 1.5 mmol scale from (1*R*,2*R*,5*R*)-(+)-2-hydroxy-3-pinanone with 20 min reaction time. The crude product was purified by column chromatography using petroleum ether/EtOAc 4:1 → 3:1 as eluent. The product is a colorless oil (380 mg, **99% yield**).

**$^1$H NMR** (500 MHz, $CDCl_3$) δ 3.85 (s, 3H), 3.01 (dd~t, *J* = 6.1 Hz, 1H), 2.81 (dd, *J* = 19.2, 2.5 Hz, 1H), 2.70 (dt, *J* = 19.2, 3.2 Hz, 1H), 2.47 (dtd, *J* = 11.3, 6.1, 2.9 Hz, 1H), 2.17 (tt, *J* = 6.1, 2.9 Hz, 1H), 1.69 (s, 3H), 1.63–1.50 (m, 1H), 1.39 (s, 3H), 0.89 (s, 3H).

**$^{13}$C NMR** (126 MHz, $CDCl_3$) δ 204.7, 158.2, 156.1, 89.7, 53.4, 48.4, 43.2, 39.4, 38.1, 27.8, 27.3, 22.5, 20.8.

$R_f$ = 0.73 (petroleum ether/EtOAc 2:1)

**HRMS** (ESI): calcd for $C_{13}H_{18}NaO_5$ $[M + Na]^+$: 277.1046, found: 277.1050.

Compound **2l** ((1-benzoyl-1*H*-indol-3-yl)methyl methyl oxalate)

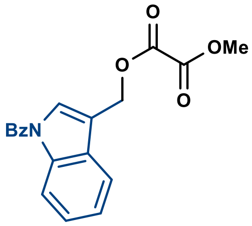

Synthesized according to the **General procedure C** described in **Section 4.1** on 1 mmol scale from alcohol **1l** with 30 min reaction time. The crude product was purified by column chromatography using petroleum ether/EtOAc 5:1 → 2:1 as eluent. The product is a white solid (337 mg, **quantitative yield**).

**$^{1}$H NMR** (500 MHz, $CDCl_3$) δ 8.39 (d, *J* = 8.2 Hz, 1H), 7.76–7.70 (m, 2H), 7.70 (d, *J* = 7.7 Hz, 1H), 7.64 (tt, *J* = 6.8, 1.1 Hz, 1H), 7.59–7.51 (m, 2H), 7.46 (s, 1H), 7.44 (ddd, *J* = 8.4, 7.3, 1.3 Hz, 1H), 7.38 (td, *J* = 7.5, 1.1 Hz, 1H), 5.46 (s, 3H), 3.87 (s, 2H).

**$^{13}$C NMR** (126 MHz, $CDCl_3$) δ 168.5, 157.8, 157.5, 136.4, 134.0, 132.2, 129.2, 129.2, 128.7, 128.4, 125.7, 124.3, 119.1, 116.6, 115.0, 60.2, 53.6.

***R*$_f$** = 0.73 (petroleum ether/EtOAc 5:1, brown color upon treatment with 5% $H_3PO_4$ in MeOH and heating)

**HRMS** (ESI): calcd for $C_{19}H_{15}NaNO_5$ $[M + Na]^+$: 360.0842, found: 360.0843.

Compound **2m** (*tert*-butyl methyl oxalate)

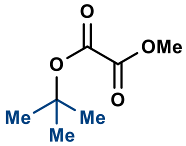

Synthesized according to the **General procedure C** described in **Section 4.1** on 3 mmol scale from *tert*-butanol with 3 h reaction time. The crude product was purified by column chromatography using hexane/EtOAc 3:1 as eluent. The product is a colorless oil (501 mg, **69% yield**).

**$^{1}$H NMR** (500 MHz, $CDCl_3$) δ 3.83 (s, 3H), 1.52 (s, 9H).

**$^{13}$C NMR** (126 MHz, $CDCl_3$) δ 158.9, 156.7, 84.9, 53.1, 27.6.

***R*$_f$** = 0.36 (petroleum ether/EtOAc 3:1).

The spectroscopic data is in agreement with the literature.[16]

Compound **2n** (methyl (2-methyl-4-phenylbutan-2-yl) oxalate)

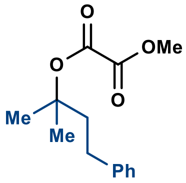

Synthesized according to the **General procedure C** described in **Section 4.1** on 2 mmol scale from 2-methyl-4-phenylbutan-2-ol with 1 h reaction time. The crude product was purified by column chromatography using hexane/EtOAc 4:1 as eluent. The product is a colorless oil (725 mg, **97% yield**).

**$^{1}$H NMR** (500 MHz, $CDCl_3$) δ 7.30–7.25 (m, 2H), 7.22–7.16 (m, 3H), 3.87 (s, 3H), 2.78–2.64 (m, 2H), 2.22–2.11 (m, 2H), 1.61 (s, 6H).

**$^{13}$C NMR** (126 MHz, $CDCl_3$) δ 158.9, 156.7, 141.5, 128.4, 128.3, 125.9, 86.8, 53.3, 42.4, 30.2, 25.7.

***R*$_f$** = 0.58 (petroleum ether/EtOAc 3:1)

**HRMS** (ESI): calcd for $C_{14}H_{18}NaO_4$ $[M + Na]^+$: 273.1098, found: 273.1095.

The spectroscopic data is in agreement with the literature.[21]

Compound **2o** (1-chloro-2-methylpropan-2-yl methyl oxalate)

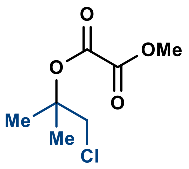


Synthesized according to the **General procedure C** described in **Section 4.1** on 1 mmol scale from 1-chloro-2-methyl-2-propanol with 1 h reaction time. The crude product was purified by column chromatography using petroleum ether/EtOAc 10:1 → 5:1 as eluent. The product is a colorless oil (151 mg, **78% yield**).

**$^{1}$H NMR** (500 MHz, $CDCl_3$) δ 3.88 (s, 3H), 3.84 (s, 2H), 1.63 (s, 6H).

**$^{13}$C NMR** (126 MHz, $CDCl_3$) δ 158.3, 156.3, 84.4, 53.5, 49.8, 24.0.

***R*$_f$** = 0.43 (petroleum ether/EtOAc 5:1)

**HRMS** (ESI): calcd for $C_7H_{11}ClNaO_4$ $[M + Na]^+$: 217.0238, found: 217.0241.

Compound **2p** ((*S*)-2-(1-(*tert*-butoxycarbonyl)-4,4-difluoropyrrolidin-2-yl)propan-2-yl methyl oxalate)

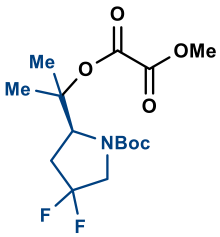


Synthesized according to the **General procedure C** described in **Section 4.1** on 1 mmol scale from *tert*-butyl (*S*)-4,4-difluoro-2-(2-hydroxypropan-2-yl)pyrrolidine-1-carboxylate **1p** with 1 h reaction time. The crude product was purified by column chromatography using hexanes/EtOAc 5:1 → 2:1 as eluent. The product is a colorless oil (395 mg, **78% yield**).

**$^{1}$H NMR** (500 MHz, $CDCl_3$) δ 4.32 – 4.00 (m, 1H), 3.87 (s, 3H), 3.73 – 3.58 (m, 1H), 2.70 – 2.28 (m, 2H), 1.63 (s, 3H), 1.55 (s, 3H), 1.47 (s, 9H).

**$^{13}$C NMR** (126 MHz, $CDCl_3$) δ 158.1, 155.9, 155.4, 128.1 (t, J = 250.6 Hz), 88.7, 81.0, 62.6, 54.3 (t, J = 32.1 Hz), 53.4, 35.6 (t, J = 23.8 Hz), 28.2, 22.4.

**$^{19}$F NMR** (377 MHz, $CDCl_3$) δ -93.7 (d, *J* = 228.2 Hz), -104.8 (d, *J* = 228.3 Hz).

***R*$_f$** = 0.52 (hexane/EtOAc 3:1)

**HRMS** (ESI): calcd for $C_{15}H_{23}NaF_2NO_6$ $[M + Na]^+$: 374.1397, found: 374.1411.

Compound **2q** (methyl (2-(5-methyl-5-vinyltetrahydrofuran-2-yl)propan-2-yl) oxalate)

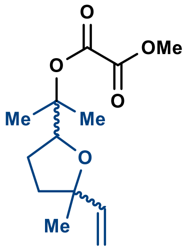


Synthesized according to the **General procedure C** described in **Section 4.1** on 1.5 mmol scale from linalool oxide (45:55 mixture of *cis*/*trans*-diastereomers) with 1 h reaction time. The crude product was purified by column chromatography using petroleum PhMe/EtOAc 10:1 as eluent. The product is a colorless oil (385 mg, **99% yield**).

**$^{1}$H NMR** (500 MHz, $CDCl_3$) δ 5.93 (dd, *J* = 17.4, 10.8 Hz, 1H, *cis*), 5.79 (dd, *J* = 17.2, 10.6 Hz, 1H, *trans*), 5.14 (dd, *J* = 17.4, 1.3 Hz, 1H, *cis*), 5.12 (dd, *J* = 17.2, 1.5 Hz, 1H, *trans*), 4.94 (dd, *J* = 7.0, 1.4 Hz, 1H, *trans*), 4.92 (dd, *J* = 7.2, 1.4 Hz, 1H, *cis*), 4.09–4.02 (m, 1H, *cis*), 4.00 (dd, *J* = 7.4, 6.2 Hz, 1H, *trans*), 3.79 (s, 3H, OMe, *trans*), 3.79 (s, 3H, OMe, *cis*), 2.02–1.63 (m, 8H, *cis* + *trans*), 1.524 (s, 3H, *trans*), 1.517 (s, 3H, *cis*), 1.493 (s, 3H, *cis*), 1.487 (s, 3H, *trans*), 1.28 (s, 3H, *trans*), 1.25 (s, 3H, *cis*).

**$^{13}$C NMR** (126 MHz, $CDCl_3$) δ 158.75 (*trans*), 158.73 (*cis*), 156.65 (*cis*), 156.58 (*trans*), 143.93 (*cis*), 143.50 (*trans*), 111.53 (*cis*), 111.43 (*trans*), 87.36 (*trans*), 87.30 (*cis*), 83.78 (*trans*), 83.75 (*cis*), 83.66 (*trans*), 83.43 (*cis*), 53.25 (*trans*), 53.22 (*cis*), 37.58 (*cis*), 36.89 (*trans*), 26.71 (*cis*), 26.47 (*trans*), 26.43 (*trans*), 25.62 (*cis*), 22.41 (*cis*), 22.27 (*trans*), 21.47 (*trans*), 21.33 (*cis*).

***R*$_f$** = 0.57, 0.64 (PhMe/EtOAc 10:1)

**HRMS** (ESI): calcd for $C_{13}H_{20}NaO_5$ $[M + Na]^+$: 279.1203, found: 279.1204.

The spectroscopic data is in agreement with the literature.[16]

## Compound **2r** (4-((*tert*-butyldimethylsilyl)oxy)-2-methylbutan-2-yl methyl oxalate)

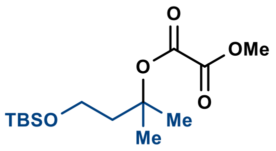


Synthesized according to the **General procedure C** described in **Section 4.1** on 5.8 mmol scale from alcohol **1r** with 1 h reaction time. The crude product was purified by column chromatography using petroleum ether/EtOAc 1:1 as eluent. The product is a colorless oil (1.77 g, **quantitative yield**).

**$^1$H NMR** (500 MHz, $CDCl_3$) δ 3.86 (s, 3H), 3.75 (t, *J* = 6.7 Hz, 2H), 2.10 (t, *J* = 6.7 Hz, 2H), 1.57 (s, 3H), 1.56 (s, 3H), 0.88 (s, 9H), 0.05 (s, 6H).

**$^{13}$C NMR** (126 MHz, $CDCl_3$) δ 158.9, 156.7, 86.5, 58.9, 53.2, 42.8, 26.2, 25.9, 18.2, −5.5.

***R*$_f$** = 0.66 (petroleum ether/EtOAc 5:1, visualized by treatment with basic $KMnO_4$ and heating)

**HRMS** (ESI): calcd for $C_{14}H_{28}NaO_5Si$ $[M + Na]^+$: 327.1598, found: 327.16025.

## Compound **2s** (4-(benzoyloxy)-2-methylbutan-2-yl methyl oxalate)

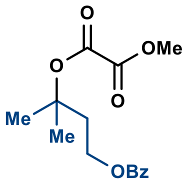


Synthesized according to the **General procedure C** described in **Section 4.1** on 7.54 mmol scale from alcohol **1s** with 1 h reaction time. The crude product was purified by column chromatography using petroleum ether/EtOAc 1:1 as eluent. The product is a yellowish oil (2.19 g, **99% yield**).

**$^1$H NMR** (500 MHz, $CDCl_3$) δ 8.08–7.92 (m, 2H), 7.63–7.50 (m, 1H), 7.44 (dd~t, *J* = 7.8 Hz, 2H), 4.48 (t, *J* = 6.6 Hz, 2H), 3.78 (s, 3H), 2.36 (t, *J* = 6.6 Hz, 2H), 1.65 (s, 6H).

**$^{13}$C NMR** (126 MHz, $CDCl_3$) δ 166.4, 158.6, 156.6, 133.0, 130.1, 129.5, 128.4, 85.4, 60.8, 53.2, 38.9, 26.1.

***R*$_f$** = 0.32 (petroleum ether/EtOAc 5:1)

**HRMS** (ESI): calcd for $C_{15}H_{18}NaO_6$ $[M + Na]^+$: 317.0996, found: 317.0998.

The spectroscopic data is in agreement with the literature.[22]

Compound **2t** (4-(4-bromobenzoyloxy)-2-methylbutan-2-yl methyl oxalate)

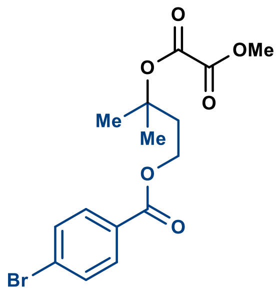

Synthesized according to the **General procedure C** described in **Section 4.1** on 1.5 mmol scale from alcohol **1t** with 30 min reaction time. The crude product was purified by column chromatography using petroleum ether/EtOAc 5:1 ⟶ 3:1 as eluent. The product is a colorless oil (545 mg, **97% yield**).

**$^1$H NMR** (500 MHz, $CDCl_3$) δ 7.93–7.80 (m, 2H), 7.65–7.49 (m, 2H), 4.46 (t, $J$ = 6.6 Hz, 2H), 3.80 (s, 3H), 2.35 (t, $J$ = 6.6 Hz, 2H), 1.64 (s, 6H).

**$^{13}$C NMR** (126 MHz, $CDCl_3$) δ 165.7, 158.6, 156.6, 131.7, 131.1, 128.9, 128.1, 85.3, 61.0, 53.3, 38.8, 26.1.

***R*$_f$** = 0.65 (petroleum ether/EtOAc 3:1)

**HRMS** (ESI): calcd for $C_{15}H_{17}BrNaO_6$ $[M + Na]^+$: 395.0101, found: 395.0102.

The spectroscopic data is in agreement with the literature.[23]

Compound **2u** (4-(nicotinoyloxy)-2-methylbutan-2-yl methyl oxalate)

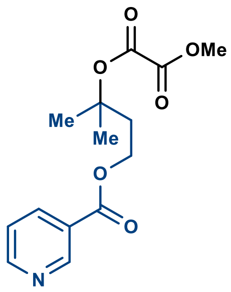

Synthesized according to the **General procedure C** described in **Section 4.1** on 1.4 mmol scale from alcohol **1u** with 2 h reaction time. The crude product was purified by column chromatography using $CH_2Cl_2$/MeOH 20:1 ⟶ 5:1 as eluent. The product is a yellowish oil (410 mg, **99% yield**).

**$^1$H NMR** (500 MHz, $CDCl_3$) δ 9.07 (s, 1H), 8.65 (d, $J$ = 3.9 Hz, 1H), 8.14 (dt, $J$ = 8.0, 2.0 Hz, 1H), 7.26 (dd, $J$ = 8.0, 4.8 Hz, 1H), 4.38 (t, $J$ = 6.7 Hz, 2H), 3.68 (s, 3H), 2.24 (t, $J$ = 6.7 Hz, 2H), 1.52 (s, 6H).

**$^{13}$C NMR** (126 MHz, $CDCl_3$) δ 165.1, 158.6, 156.6, 153.5, 150.9, 137.0, 125.9, 123.3, 85.2, 61.2, 53.3, 38.9, 26.1.

***R*$_f$** = 0.36 (petroleum ether/EtOAc 1:1); 0.49 ($CH_2Cl_2$/MeOH 10:1)

**HRMS** (ESI): calcd for $C_{14}H_{17}NaNO_6$ $[M + Na]^+$: 318.0948, found: 318.0953.

Compound **2v** (4-(2-thiophenecarboxy)-2-methylbutan-2-yl methyl oxalate)

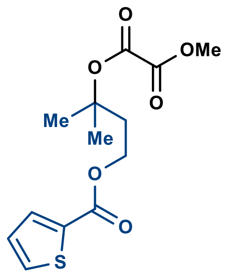

Synthesized according to the **General procedure C** described in **Section 4.1** on 0.79 mmol scale from alcohol **1v** with 2 h reaction time. The crude product was purified by column chromatography using petroleum ether/EtOAc 5:1 ⟶ 2:1 as eluent. The product is a yellowish oil (235 mg, **quantitative yield**).

**$^1$H NMR** (500 MHz, $CDCl_3$) δ 7.72 (dd, $J$ = 3.8, 1.3 Hz, 1H), 7.50 (dd, $J$ = 5.0, 1.3 Hz, 1H), 7.04 (dd, $J$ = 5.0, 3.8 Hz, 1H), 4.38 (t, $J$ = 6.6 Hz, 2H), 3.76 (s, 3H), 2.27 (t, $J$ = 6.6 Hz, 2H), 1.58 (s, 6H).

**$^{13}$C NMR** (126 MHz, $CDCl_3$) δ 162.0, 158.6, 156.6, 133.6, 133.5, 132.5, 127.8, 85.4, 60.9, 53.3, 39.0, 26.0.

***R*$_f$** = 0.67 (petroleum ether/EtOAc 2:1)

**HRMS** (ESI): calcd for $C_{13}H_{16}NaO_6S$ $[M + Na]^+$: 323.0560, found: 323.0563.

The spectroscopic data is in agreement with the literature.[23]

Compound **2w** (2:3,4:5-diacetylidene-β-D-glucopyranosyloxy)-2-methylbutan-2-yl methyl oxalate)

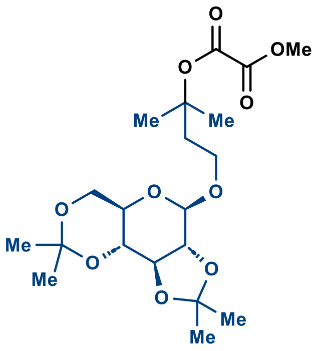


Synthesized according to the **General procedure C** described in **Section 4.1** on 0.43 mmol scale from alcohol **1w** with 1 h reaction time. The crude product was purified by column chromatography using petroleum ether/EtOAc 5:1 ⟶ 2:1 as eluent. The product is a white solid (186 mg, **quantitative yield**).

**$^1$H NMR** (500 MHz, $CDCl_3$) δ 4.30 (d, *J* = 7.7 Hz, 1H), 4.03 (dt, *J* = 10.0, 6.7 Hz, 1H), 3.87 (s, 3H), 3.91–3.86 (m, 2H, overlapping with $CH_3$ peak) 3.68 (dt, *J* = 10.0, 6.7 Hz, 1H), 3.60 (dd~t, *J* = 9.0 Hz, 1H), 3.54 (dd~t, *J* = 9.0 Hz, 1H), 3.40–3.28 (m, 2H), 2.27 (dt, *J* = 14.0, 6.8 Hz, 1H), 2.16 (dt, *J* = 14.0, 6.8 Hz, 1H), 1.57 (s, 6H), 1.56 (s, 6H).

**$^{13}$C NMR** (126 MHz, $CDCl_3$) δ 159.1, 156.7, 102.9 (C-1), 85.7 (2 × C), 76.3, 75.4, 73.4, 69.7, 65.8, 61.7, 53.5 (2 × $CH_3$), 39.2, 26.3 (2 × $CH_3$), 26.1 (2 × $CH_3$).

***$R_f$*** = 0.64 (petroleum ether/EtOAc 4:1)

**HRMS** (ESI): calcd for $C_{20}H_{32}NaO_{10}$ $[M + Na]^+$: 455.1887, found: 455.1894.

Compound **2x** (cyclohexyl methyl oxalate)

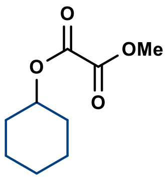


Synthesized according to the **General procedure C** described in **Section 4.1** on 2 mmol scale from cyclohexanol with 5 min reaction time. The crude product was purified by column chromatography using petroleum ether/EtOAc 10:1 ⟶ 5:1 as eluent. The product is a colorless oil (341 mg, **92% yield**).

**$^1$H NMR** (500 MHz, $CDCl_3$) δ 4.94 (td, *J* = 9.5, 4.7 Hz, 1H), 3.90 (s, 3H), 1.97–1.90 (m, 2H), 1.83–1.73 (m, 2H), 1.62–1.50 (m, 4H), 1.44–1.34 (m, 2H), 1.28 (tdd, *J* = 12.8, 9.0, 3.7 Hz, 1H).

**$^{13}$C NMR** (126 MHz, $CDCl_3$) δ 158.6, 157.1, 76.4, 53.4, 31.2, 25.1, 23.6.

***$R_f$*** = 0.64 (petroleum ether/EtOAc 5:1)

Compound **2y** (*n*-hexyl methyl oxalate)

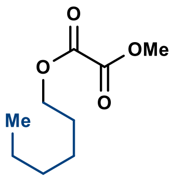


Synthesized according to the **General procedure B** described in **Section 4.1** on 2 mmol scale from *n*-hexanol with 20 min reaction time. The crude product was purified by column chromatography using petroleum ether/EtOAc 20:1 as eluent. The product is a colorless oil (377 mg, **99% yield**).

**$^1$H NMR** (500 MHz, $CDCl_3$) δ 4.29 (t, *J* = 6.8 Hz, 2H), 3.91 (s, 3H), 1.73 (p, *J* = 7.0 Hz, 2H), 1.45–1.24 (m, 6H), 0.97–0.70 (m, 3H).

**$^{13}$C NMR** (126 MHz, $CDCl_3$) δ 158.3, 157.7, 67.3, 53.5, 31.3, 28.2, 25.3, 22.5, 14.0.

***R*$_f$** = 0.50 (petroleum ether/EtOAc 10:1)

**HRMS** (ESI): calcd for $C_9H_{16}NaO_4$ [M + Na]$^+$: 211.0941, found: 211.0942.

## Compound **3a-Na** (sodium 2-(1-methyl-cyclohexyloxy)-2-oxoacetate)

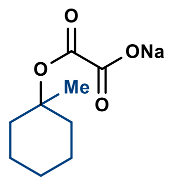


Synthesized according to the **General procedure D** described in **Section 4.1** on 0.72 mmol scale from methyl oxalate **2a** with 10 min reaction time. The product is a white solid (135.2 mg, **90% yield**).

**$^1$H NMR** (400 MHz, DMSO) δ 2.08–1.91 (m, 2H), 1.54–1.29 (m, 7H), 1.38 (s, 3H), 1.20 (tdd, *J* = 13.8, 9.7, 5.7 Hz, 1H).

**$^{13}$C NMR** (101 MHz, DMSO) δ 167.1, 163.7, 79.9, 36.1, 25.3, 24.9, 21.5.

***R*$_f$** = 0.00 (petroleum ether/EtOAc 10:1)

**HRMS** (ESI): calcd for $C_9H_{13}O_4$ [M − Na]$^-$: 185.0819, found: 185.0817.

## Compound **3x-Na** (sodium 2-(cyclohexyloxy)-2-oxoacetate)

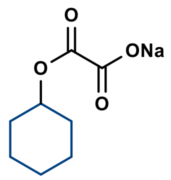


Synthesized according to the **General procedure D** described in **Section 4.1** on 1.5 mmol scale from methyl oxalate **2x** with 20 min reaction time. The product is a white solid (280 mg, **93% yield**).

**$^1$H NMR** (500 MHz, DMSO) δ 4.56 (dp, *J* = 9.1, 3.9 Hz, 1H), 1.76 (dt, *J* = 8.3, 3.8 Hz, 2H), 1.66 (dq, *J* = 12.4, 4.1 Hz, 2H), 1.50 (ddd, *J* = 12.4, 6.0, 3.1 Hz, 1H), 1.41–1.26 (m, 4H), 1.21 (td, *J* = 13.4, 6.4 Hz, 1H).

**$^{13}$C NMR** (126 MHz, DMSO) δ 166.7, 163.0, 70.7, 50.1, 31.3, 24.9, 23.4.

***R*$_f$** = 0.00 (petroleum ether/EtOAc 10:1)

**HRMS** (ESI): calcd for $C_8H_{11}O_4$ [M − Na]$^-$: 171.0663, found: 171.0659.

## Compound **3y-Na** (sodium 2-(hexyloxy)-2-oxoacetate)

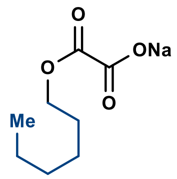


Synthesized according to the **General procedure D** described in **Section 4.1** on 1 mmol scale from methyl oxalate **2y** with 20 min reaction time. The product is a white solid (110 mg, **56% yield**).

**$^1$H NMR** (500 MHz, DMSO) δ 3.92 (t, *J* = 6.7 Hz, 2H), 1.60–1.49 (m, 2H), 1.35–1.20 (m, 6H), 0.91–0.82 (m, 3H).

**$^{13}$C NMR** (126 MHz, DMSO) δ 166.97, 162.61, 62.62, 50.01, 30.92, 28.21, 25.11, 22.02, 13.89.

***R*$_f$** = 0.00 (petroleum ether/EtOAc 10:1)

**HRMS** (ESI): calcd for $C_8H_{13}O_4$ [M − Na]$^-$: 173.0819, found: 173.0817.

Compound **5a** (ethyl (*R*)-2-(((*R*)-mesitylsulfinyl)amino)-2-(1-methylcyclohexyl)acetate)

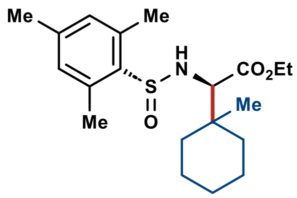

Synthesized according to the general procedure described in **Section 4.2** on 0.3 mmol scale from methyl oxalate **2a** with 2 h reaction time. The crude product was purified by column chromatography using hexane/EtOAc/$CH_2Cl_2$ 8:1:1 as eluent. The product is a brownish oil (98.7 mg, **90% yield**).

**$^1$H NMR** (500 MHz, $CDCl_3$) δ 6.87 (s, 2H), 5.01 (d, *J* = 10.1 Hz, 1H), 4.55–4.01 (m, 2H), 3.83 (d, *J* = 10.1 Hz, 0H), 2.57 (s, 6H), 2.29 (s, 3H), 1.68–1.38 (m, 7H), 1.38–1.22 (m, 3H), 1.30 (t, *J* = 7.2 Hz, 3H), 0.84 (s, 3H).

**$^{13}$C NMR** (126 MHz, $CDCl_3$) δ 172.7, 140.8, 138.2, 136.7, 130.7, 61.2, 37.6, 34.8, 34.7, 25.9, 21.6, 21.5, 21.0, 20.6, 19.3, 14.2.

***R*$_f$** = 0.25 (hexane/EtOAc/$CH_2Cl_2$ 8:1:1)

**HRMS** (ESI): calcd for $C_{20}H_{31}NaNO_3S$ $[M + Na]^+$: 388.1917, found: 388.1923.

The spectroscopic data is in agreement with the literature.[1]

Compound **5b** (ethyl (*R*)-2-(((*R*)-mesitylsulfinyl)amino)-2-(1-methylcyclopentyl)acetate)

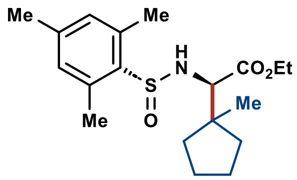

Synthesized according to the general procedure described in **Section 4.2** on 0.3 mmol scale from methyl oxalate **2b** with 2 h reaction time. The crude product was purified by column chromatography using hexane/$CH_2Cl_2$/EtOAc 8:1:1 as eluent. The product is a brownish oil (65 mg, **62% yield**).

**$^1$H NMR** (500 MHz, $CDCl_3$) δ 6.87 (s, 2H), 5.16 (d, *J* = 9.6 Hz, 1H), 4.44–3.93 (m, 2H), 3.74 (d, *J* = 9.6 Hz, 1H), 2.58 (s, 6H), 2.29 (s, 3H), 1.79 (dt, *J* = 12.5, 7.9 Hz, 1H), 1.73–1.57 (m, 5H), 1.42–1.31 (m, 2H), 1.30 (t, *J* = 7.2 Hz, 3H), 0.89 (s, 3H).

**$^{13}$C NMR** (126 MHz, $CDCl_3$) δ 172.8, 140.8, 138.1, 136.8, 130.7, 65.2, 61.3, 46.6, 37.3, 36.9, 24.4, 23.8, 22.3, 21.0, 19.3, 14.2.

***R*$_f$** = 0.13 (hexane/$CH_2Cl_2$/EtOAc 8:1:1)

**HRMS** (ESI): calcd for $C_{19}H_{29}NaNO_3S$ $[M + Na]^+$: 374.1760, found: 374.1772.

Compound **5c** (ethyl (*R*)-2-(((*R*)-mesitylsulfinyl)amino)-2-(2-methyl-2,3-dihydro-1*H*-inden-2-yl)acetate)

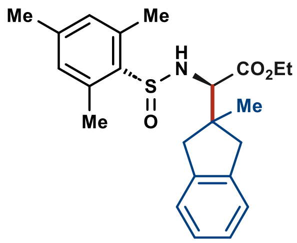

Synthesized according to the general procedure described in **Section 4.2** on 0.45 mmol scale from methyl oxalate **2b** (1.5 equiv.) with 4 h reaction time. The crude product was purified by column chromatography using petroleum ether/EtOAc 4:1 → 1:1 as eluent. The product is a brownish oil (31.2 mg, **21% yield**).

**$^1$H NMR** (500 MHz, $CDCl_3$) δ 7.21–7.09 (m, 4H), 6.88 (s, 2H), 5.26 (d, *J* = 9.5 Hz, 1H), 4.19 (qd, *J* = 7.1, 1.2 Hz, 2H), 3.97 (d, *J* = 9.5 Hz, 1H), 3.24 (d, *J* = 15.8 Hz, 1H), 3.13 (d, *J* = 15.8 Hz, 1H), 2.66 (d, *J* = 15.8 Hz, 1H), 2.60 (d, *J* = 15.8 Hz, 2H), 2.60 (s, 6H), 2.30 (s, 3H), 1.31 (t, *J* = 7.1 Hz, 3H), 1.04 (s, 3H).

**$^{13}$C NMR** (126 MHz, $CDCl_3$) δ 172.4, 141.9, 141.4, 141.0, 137.9, 136.8, 130.8, 126.4, 126.4, 124.8, 124.7, 64.7, 61.7, 47.4, 44.1, 43.5, 22.8, 21.0, 19.4, 14.2.

***R*$_f$** = 0.20 (petroleum ether/EtOAc 1:1)

**HRMS** (ESI): calcd for $C_{23}H_{29}NaNO_3S$ $[M + Na]^+$: 422.1760, found: 422.1760.

Compound **5d** (2-((1*R*,2*R*,4a*S*,8a*S*)-2-((*R*)-2-ethoxy-1-(((*R*)-mesitylsulfinyl)amino)-2-oxoethyl)-2,5,5,8a-tetramethyldecahydronaphthalen-1-yl)ethyl benzoate)

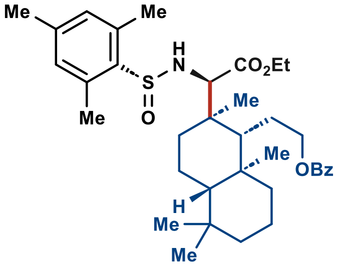


Synthesized according to the general procedure described in **Section 4.2** on 0.36 mmol scale from methyl oxalate **2d** with 4 h reaction time. The crude product was purified by column chromatography using petroleum ether/EtOAc 4:1 → 1:1 as eluent. The product is a brownish oil (87 mg, **48% yield**).

**$^1$H NMR** (500 MHz, $CDCl_3$) δ 8.17–8.04 (m, 2H), 7.61–7.52 (m, 1H), 7.46 (t, *J* = 7.7 Hz, 2H), 6.86 (s, 2H), 5.07 (d, *J* = 10.1 Hz, 1H), 4.48 (td, *J* = 10.8, 6.1 Hz, 1H), 4.41 (td, *J* = 10.7, 5.8 Hz, 1H), 4.36–4.22 (m, 2H), 3.83 (d, *J* = 10.1 Hz, 1H), 2.57 (s, 6H), 2.28 (s, 3H), 1.88–1.73 (m, 3H), 1.64–1.52 (m, 2H), 1.49–1.39 (m, 2H), 1.37 (d, *J* = 7.1 Hz, 4H), 1.31–1.18 (m, 2H), 1.17–1.07 (m, 2H), 1.04 (s, 3H), 0.94 (s, 3H), 0.91–0.85 (m, 1H), 0.83 (s, 4H), 0.78 (s, 3H), 0.70 (dd, *J* = 12.1, 2.0 Hz, 1H).

**$^{13}$C NMR** (126 MHz, $CDCl_3$) δ 172.7, 166.6, 141.0, 138.1, 136.7, 132.8, 130.8, 130.5, 129.6, 128.3, 66.2, 65.6, 61.8, 56.0, 51.5, 43.2, 41.7, 40.8, 39.6, 34.1, 33.2, 33.2, 25.9, 21.6, 21.1, 19.3, 18.4, 18.3, 17.9, 16.5, 14.2.

***R*$_f$** = 0.39 (petroleum ether/EtOAc 4:1)

**HRMS** (ESI): calcd for $C_{36}H_{51}NaNO_5S$ $[M + Na]^+$: 632.3380, found: 632.3372.

Compound **5e** (ethyl (*R*)-2-(((*R*)-mesitylsulfinyl)amino)-2-((3*R*,3a*S*,6*S*,7*R*,8a*S*)-3,6,8,8-tetramethyloctahydro-1*H*-3a,7-methanoazulen-6-yl)acetate)

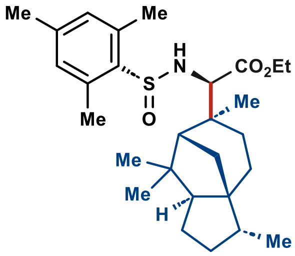


Synthesized according to the general procedure described in **Section 4.2** on 0.3 mmol scale from methyl oxalate **2e** with 2 h reaction time. The crude product was purified by column chromatography using petroleum ether/EtOAc 5:1 → 4:1 as eluent. The product is a brownish oil (30 mg, **21% yield**).

**$^1$H NMR** (500 MHz, $CDCl_3$) δ 6.87 (s, 2H), 5.04 (d, *J* = 9.7 Hz, 1H), 4.33 (d, *J* = 9.7 Hz, 1H), 4.28 (dq, *J* = 10.7, 7.1 Hz, 1H), 4.15 (dq, *J* = 10.7, 7.1 Hz, 1H), 2.58 (s, 6H), 2.29 (s, 3H), 1.98–1.86 (m, 3H), 1.75 (dd~t, *J* = 8.4 Hz, 1H), 1.71 (q, *J* = 7.1 Hz, 1H), 1.64 (ddd, *J* = 13.1, 4.8, 2.6 Hz, 1H), 1.56–1.46 (m, 2H), 1.45–1.35 (m, 3H), 1.35–1.30 (m, 2H), 1.28 (t, *J* = 7.1 Hz, 3H), 1.20 (s, 3H), 1.01 (s, 3H), 0.89 (s, 3H), 0.88 (d, *J* = 7.1 Hz, 3H).

**$^{13}$C NMR** (126 MHz, $CDCl_3$) δ 173.1, 140.8, 138.2, 136.7, 130.8, 61.2, 61.1, 57.8, 56.8, 53.5, 45.0, 43.1, 41.7, 40.2, 37.0, 30.0, 29.9, 29.5, 29.3, 25.4, 21.0, 20.2, 19.3, 15.5, 14.1.

$\boldsymbol{R_f}$ = 0.50 (petroleum ether/EtOAc 3:1)

**HRMS** (ESI): calcd for $C_{28}H_{43}NaNO_3S$ $[M + Na]^+$: 469.2856, found: 496.2853.

The spectroscopic data is in agreement with the literature.[24]

## Compound **5f** (ethyl (*R*)-2-(1-benzoylcyclohexyl)-2-(((*R*)-mesitylsulfinyl)amino)acetate)

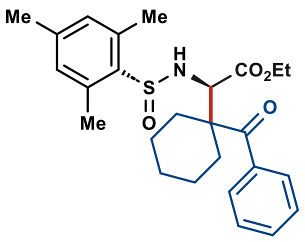


Synthesized according to the general procedure described in **Section 4.2** on 0.36 mmol scale from methyl oxalate **2f** with 2 h reaction time. The crude product was purified by column chromatography using petroleum ether/EtOAc 3:1 → 2:1 as eluent. The product is a brownish oil (60 mg, **44% yield**).

**$^1$H NMR** (500 MHz, $CDCl_3$) δ 7.66–7.59 (m, 2H), 7.49–7.42 (m, 1H), 7.37 (dd~t, *J* = 7.7 Hz, 2H), 6.86 (s, 1H), 5.37 (d, *J* = 9.5 Hz, 0H), 4.49 (d, *J* = 9.5 Hz, 0H), 4.20 (ABqq, *J* = 7.2, 4.1 Hz, 1H), 2.51 (s, 6H), 2.29 (s, 3H), 2.27–2.11 (m, 2H), 1.75–1.65 (m, 1H), 1.61–1.44 (m, 4H), 1.22 (t, *J* = 7.2 Hz, 3H), 1.20–1.11 (m, 3H).

**$^{13}$C NMR** (126 MHz, $CDCl_3$) δ 206.3, 171.2, 141.1, 139.7, 137.8, 136.7, 130.8, 128.1, 127.4, 62.5, 62.0, 56.4, 31.1, 30.5, 25.4, 22.5, 22.4, 21.0, 19.2, 13.9.

$\boldsymbol{R_f}$ = 0.18 (petroleum ether/EtOAc 5:1)

**HRMS** (ESI): calcd for $C_{26}H_{33}NaNO_4S$ $[M + Na]^+$: 478.2022, found: 478.2025.

## Compound **5g** (*tert*-butyl 4-((*R*)-2-ethoxy-1-(((*R*)-mesitylsulfinyl)amino)-2-oxoethyl)-4-methylpiperidine-1-carboxylate)

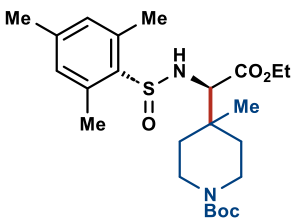


Synthesized according to the general procedure described in **Section 4.2** on 0.3 mmol scale from methyl oxalate **2g** with 2 h reaction time. The crude product was purified by column chromatography using hexane/$CH_2Cl_2$/EtOAc 8:1:1 as eluent. The product is a brownish oil (64 mg, **46% yield**).

**$^1$H NMR** (500 MHz, $CDCl_3$) δ 6.87 (s, 2H), 5.04 (d, *J* = 10.0 Hz, 1H), 4.31–4.12 (m, 2H), 3.77 (d, *J* = 10.0 Hz, 1H), 3.70 (br.s, 2H), 3.19–3.07 (m, 2H), 2.56 (s, 6H), 2.28 (s, 3H), 1.67 (ddd, *J* = 14.0, 10.0, 4.3 Hz, 1H), 1.55 (ddd, *J* = 14.0, 9.9, 4.8 Hz, 1H), 1.44 (s, 9H), 1.43–1.37 (m, 1H), 1.30 (t, *J* = 7.1 Hz, 3H), 1.33–1.23 (m, 1H), 0.91 (s, 3H).

**$^{13}$C NMR** (126 MHz, $CDCl_3$) δ 172.0, 154.8, 141.0, 137.7, 136.7, 130.8, 79.5, 64.7, 61.5, 36.3, 33.9, 33.8, 28.4, 21.0, 19.3, 18.8, 14.1.

$\boldsymbol{R_f}$ = 0.11 (hexane/$CH_2Cl_2$/EtOAc 8:1:1)

**HRMS** (ESI): calcd for $C_{24}H_{38}NaN_2O_5S$ $[M + Na]^+$: 489.2394, found: 489.2394.

The spectroscopic data is in agreement with the literature.[1]

Compound **5h** (ethyl (2*R*,3*R*)-2-(((*R*)-mesitylsulfinyl)amino)-3-(tetrahydro-2*H*-thiopyran-4-yl)butanoate)

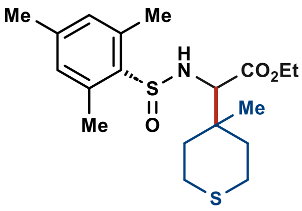


Synthesized according to the general procedure described in **Section 4.2** on 0.36 mmol scale from methyl oxalate **2h** with 2 h reaction time. The crude product was purified by column chromatography using petroleum ether/EtOAc 5:1 → 3:1 as eluent. The product is a brownish oil (72 mg, **76% yield**).

**$^1$H NMR** (500 MHz, $CDCl_3$) δ 6.86 (s, 2H), 5.00 (d, *J* = 10.2 Hz, 1H), 4.42 – 4.15 (m, 2H), 3.81 (d, *J* = 10.2 Hz, 1H), 2.80 – 2.57 (m, 4H), 2.54 (s, 6H), 2.27 (s, 3H), 1.94 – 1.77 (m, 2H), 1.72 – 1.55 (m, 2H), 1.29 (t, *J* = 7.2 Hz, 3H), 0.87 (s, 3H).

**$^{13}$C NMR** (126 MHz, $CDCl_3$) δ 172.0, 141.0, 137.7, 136.6, 130.8, 64.1, 61.5, 36.6, 35.3, 35.1, 23.2, 23.2, 21.0, 20.3, 19.2, 14.1.

$R_f$ = 0.40 (hexanes/EtOAc 3:1)

**HRMS** (ESI): calcd for $C_{19}H_{29}NaNO_3S_2$ $[M + Na]^+$: 406.1495, found: 406.1508.

Compound **5i** (*tert*-butyl 6-((*R*)-2-ethoxy-1-(((*R*)-mesitylsulfinyl)amino)-2-oxoethyl)-6-methyl-2-azaspiro[3.3]heptane-2-carboxylate)

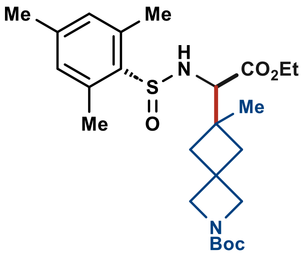


Synthesized according to the general procedure described in **Section 4.2** on 0.36 mmol scale from methyl oxalate **2i** with 2 h reaction time. The crude product was purified by column chromatography using petroleum ether/EtOAc 5:1 → 3:1 as eluent. The product is a brownish oil (38 mg, **27% yield**).

**$^1$H NMR** (500 MHz, $CDCl_3$) δ 6.87 (s, 2H), 5.14 (d, *J* = 8.4 Hz, 1H), 4.31 – 4.12 (m, 2H), 3.87 (d, *J* = 8.4 Hz, 1H), 2.57 (s, 6H), 2.51 – 2.43 (m, 1H), 2.29 (s, 4H, $CH_3$ overlapped with $CH_2$), 2.01 – 1.90 (m, 2H), 1.42 (s, 9H), 1.28 (t, J = 7.2 Hz, 3H), 0.96 (s, 3H).

**$^{13}$C NMR** (126 MHz, $CDCl_3$) δ 171.7, 156.0, 141.0, 137.8, 136.7, 131.8, 130.8, 79.3, 64.6, 61.7, 43.1, 43.0, 37.0, 31.6, 28.4, 28.4, 21.7, 21.0, 19.4, 14.2.

$R_f$ = 0.56 (hexanes/EtOAc 2:1)

**HRMS** (ESI): calcd for $C_{25}H_{38}NaN_2O_5S$ $[M + Na]^+$: 501.2409, found: 501.2412.

Compound **5j** (*tert*-butyl 2-((*R*)-2-ethoxy-1-(((*R*)-mesitylsulfinyl)amino)-2-oxoethyl)-2-methyl-7-azaspiro[3.5]nonane-7-carboxylate)

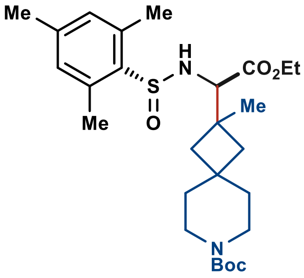


Synthesized according to the general procedure described in **Section 4.2** on 0.36 mmol scale from methyl oxalate **2j** with 3 h reaction time. The crude product was purified by column chromatography using petroleum ether/EtOAc 5:1 → 2:1 as eluent. The product is a brownish oil 39 mg, **26% yield**).

**$^1$H NMR** (500 MHz, $CDCl_3$) δ 6.87 (s, 2H), 5.14 (d, *J* = 8.7 Hz, 1H), 4.30 – 4.12 (m, 2H), 3.87 (d, *J* = 8.7 Hz, 1H), 3.47 – 3.20 (m, 4H), 2.58 (s, 6H), 2.28 (s, 3H), 2.18

(d, $J$ = 12.6 Hz, 1H), 1.95 (d, $J$ = 12.6 Hz, 1H), 1.69 – 1.53 (m, 4H), 1.50 – 1.45 (m, 2H), 1.44 (s, 9H), 1.27 (t, $J$ = 7.1 Hz, 3H), 1.05 (s, 3H).

**$^{13}$C NMR** (126 MHz, $CDCl_3$) δ 172.0, 154.9, 140.9, 137.9, 136.7, 131.8, 130.8, 79.3, 65.6, 61.5, 41.6 (br), 41.5, 40.2 (br), 38.7 (br), 35.2, 29.9, 28.4, 24.6 (br), 21.0, 19.4, 14.2.

***R*$_f$** = 0.55 (hexanes/EtOAc 2:1)

**HRMS** (ESI): calcd for $C_{27}H_{42}NaN_2O_5S$ $[M + Na]^+$: 529.2721, found: 529.2726.

Compound **5k** (ethyl (2*R*)-2-(((*R*)-mesitylsulfinyl)amino)-2-(2,6,6-trimethyl-3-oxobicyclo[3.1.1]heptan-2-yl)acetate)

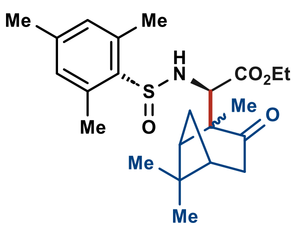


Synthesized according to the general procedure described in **Section 4.2** on 0.36 mmol scale from methyl oxalate **2k** with 2 h reaction time. The crude product was purified by column chromatography using petroleum ether/EtOAc 4:1 → 1:1 as eluent. Two β-diastereomers of **5k** were isolated (**5k-1** and **5k-2**) as brownish oils (**5k-1**: 47 mg, **37% yield**; **5k-2**: 50 mg, 40% yield, for **5k-2;** in total for **5k**: 97 mg, 77%).

**$^1$H NMR** for β-diastereomer **5k-1** (500 MHz, $CDCl_3$) δ 6.85 (s, 2H), 5.13 (d, $J$ = 8.5 Hz, 1H), 4.28 (d, $J$ = 8.5 Hz, 1H), 4.16–4.08 (m, 2H), 2.72–2.58 (m, 2H), 2.57 (s, 6H), 2.55–2.46 (m, 2H), 2.28 (s, 3H), 2.09 (tq, $J$ = 6.0, 2.6 Hz, 1H), 1.92 (t, $J$ = 6.2 Hz, 1H), 1.73 (d, $J$ = 11.6 Hz, 1H), 1.32 (s, 3H), 1.26 (t, $J$ = 7.2 Hz, 3H), 1.16 (s, 3H), 0.91 (s, 3H).

**$^{13}$C NMR** for β-diastereomer **5k-1** (126 MHz, $CDCl_3$) δ 211.6, 170.8, 140.9, 137.2, 130.8, 61.6, 59.6, 54.7, 48.8, 45.1, 40.6, 38.7, 30.1, 27.5, 23.1, 21.0, 19.3, 19.0, 14.0.

***R*$_f$** for β-diastereomer **5k-1** = 0.44 (petroleum ether/EtOAc 2:1)

**HRMS** (ESI) for β-diastereomer **5k-1**: calcd for $C_{23}H_{33}NaNO_4S$ $[M + Na]^+$: 442.2022, found: 442.2029.

**$^1$H NMR** for β-diastereomer **5k-2** (500 MHz, $CDCl_3$) δ 6.86 (s, 2H), 6.76 (dt, $J$ = 6.0, 1.7 Hz, 1H), 5.03 (d, $J$ = 10.3 Hz, 1H), 4.45–4.13 (m, 2H), 3.86 (d, $J$ = 10.3 Hz, 1H), 2.59–2.56 (m, 1H), 2.53 (s, 6H), 2.47 (dd, $J$ = 16.0, 6.0 Hz, 1H), 2.28 (s, 3H), 2.25–2.10 (m, 3H), 1.77 (s, 3H), 1.29 (t, $J$ = 7.1 Hz, 3H), 0.91 (s, 3H), 0.88 (s, 3H).

**$^{13}$C NMR** for β-diastereomer **5k-2** (126 MHz, $CDCl_3$) δ 199.6, 172.3, 144.9, 141.1, 137.8, 136.8, 135.3, 130.8, 63.0, 61.6, 41.1, 39.7, 39.6, 27.2, 21.0, 20.4, 20.0, 19.2, 15.5, 14.1.

***R*$_f$** for β-diastereomer **5k-2** = 0.38 (petroleum ether/EtOAc 2:1)

**HRMS** (ESI) for β-diastereomer **5k-2**: calcd for $C_{23}H_{33}NaNO_4S$ $[M + Na]^+$: 442.2022, found: 442.2031.

## Compound **5l** (ethyl 1-benzoyl-$N^{\alpha}$-((*R*)-mesitylsulfinyl)-*D*-tryptophanate)

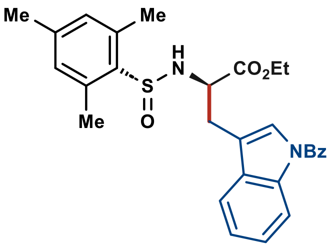


Synthesized according to the general procedure described in **Section 4.2** on 0.36 mmol scale from methyl oxalate **2l** with 2 h reaction time. The crude product was purified by column chromatography using petroleum ether/EtOAc 5:1 → 1:1 as eluent. The product is a brownish oil (50 mg, **33% yield**).

**$^1$H NMR** (500 MHz, $CDCl_3$) δ 8.37 (d, *J* = 8.2 Hz, 1H), 7.67–7.61 (m, 2H), 7.62–7.55 (m, 1H), 7.52 (d, *J* = 7.7 Hz, 1H), 7.46 (dd, *J* = 8.2, 7.3 Hz, 2H), 7.38 (dd, *J* = 7.9, 7.0 Hz, 1H), 7.29 (d, *J* = 7.9 Hz, 1H), 6.79 (s, 2H), 5.12 (d, *J* = 8.3 Hz, 1H), 4.32 (ddd, *J* = 8.3, 7.8, 5.0 Hz, 1H), 4.20–4.08 (m, 2H), 3.23 (dd, *J* = 14.8, 5.0 Hz, 1H), 3.03 (dd, *J* = 14.8, 7.7 Hz, 1H), 2.36 (s, 6H), 2.27 (s, 3H), 1.18 (t, *J* = 7.2 Hz, 3H).

**$^{13}$C NMR** (126 MHz, $CDCl_3$) δ 172.6, 168.3, 140.9, 137.6, 136.6, 136.2, 134.5, 131.8, 130.8, 130.6, 129.0, 128.6, 125.9, 125.3, 123.9, 118.9, 116.8, 116.5, 62.0, 57.2, 29.8, 21.0, 19.1, 14.0.

***$R_f$*** = 0.23 (petroleum ether/EtOAc 2:1)

**HRMS** (ESI): calcd for $C_{29}H_{30}NaN_2O_4S$ $[M + Na]^+$: 525.1819, found: 525.1816.

## Compound **5m** (ethyl (*R*)-2-(((*R*)-mesitylsulfinyl)amino)-3,3-dimethylbutanoate)

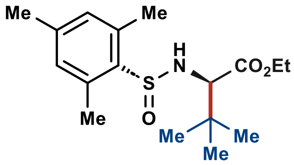


Synthesized according to the general procedure described in **Section 4.2** on 0.3 mmol scale from methyl oxalate **2m** with 2 h reaction time. The crude product was purified by column chromatography using hexane/$CH_2Cl_2$/EtOAc 8:1:1 as eluent. The product is a brownish oil (65 mg, **67% yield**).

**$^1$H NMR** (500 MHz, $CDCl_3$) δ 6.87 (s, 2H), 5.05 (d, *J* = 10.0 Hz, 1H), 4.47–4.10 (m, 2H), 3.60 (d, *J* = 10.0 Hz, 1H), 2.57 (s, 6H), 2.29 (s, 3H), 1.31 (t, *J* = 7.1 Hz, 3H), 0.96 (s, 9H).

**$^{13}$C NMR** (126 MHz, $CDCl_3$) δ 172.7, 140.8, 138.1, 136.7, 130.8, 66.5, 61.3, 35.0, 26.5, 21.0, 19.3, 14.2

***$R_f$*** = 0.32 (hexane/EtOAc 4:1)

**HRMS** (ESI): calcd for $C_{17}H_{27}NaNO_3S$ $[M + Na]^+$: 348.1604, found: 348.1609.

The spectroscopic data is in agreement with the literature.[1]

Compound **5n** (ethyl (*R*)-2-(((*R*)-mesitylsulfinyl)amino)-3,3-dimethyl-5-phenylpentanoate)

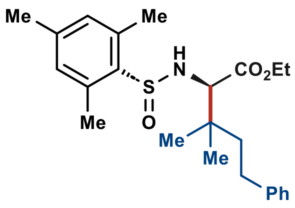


Synthesized according to the general procedure described in **Section 4.2** on 0.36 mmol scale from methyl oxalate **2n** with 2 h reaction time. The crude product was purified by column chromatography using petroleum ether/EtOAc 20:1 → 5:1 as eluent. The product is a brownish oil (117 mg, **94% yield**).

**$^1$H NMR** (500 MHz, $CDCl_3$) δ 7.30–7.23 (m, 2H), 7.21–7.10 (m, 3H), 6.87 (s, 2H), 5.08 (d, *J* = 10.1 Hz, 1H), 4.32–4.19 (m, 2H), 3.81 (d, *J* = 10.1 Hz, 1H), 2.71–2.57 (m, 2H), 2.56 (s, 6H), 2.29 (s, 3H), 1.66 (ddd, *J* = 13.6, 12.3, 5.3 Hz, 1H), 1.63–1.48 (m, 1H), 1.31 (t, *J* = 7.2 Hz, 3H), 1.00 (s, 6H).

**$^{13}$C NMR** (126 MHz, $CDCl_3$) δ 172.7, 142.5, 141.0, 138.0, 136.8, 130.8, 128.4, 128.3, 125.8, 64.7, 61.5, 41.5, 37.7, 30.3, 23.9, 23.7, 21.0, 19.3, 14.2.

***R*$_f$** = 0.32 (petroleum ether/EtOAc 5:1)

**HRMS** (ESI): calcd for $C_{24}H_{33}NaNO_3S$ $[M + Na]^+$: 438.2073, found: 438.2073.

Compound **5o** (ethyl (*R*)-4-chloro-2-(((*R*)-mesitylsulfinyl)amino)-3,3-dimethylbutanoate)

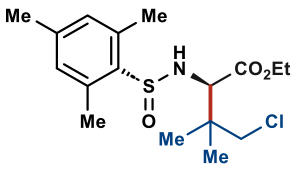


Synthesized according to the general procedure described in **Section 4.2** on 0.3 mmol scale from methyl oxalate **2o** with 2 h reaction time. The crude product was purified by preparative TLC using hexane/EtOAc 9:1 as eluent. The product is a brownish oil (24 mg, **22% yield**).

**$^1$H NMR** (500 MHz, $CDCl_3$) δ 6.88 (s, 2H), 5.07 (d, *J* = 10.2 Hz, 1H), 4.42–4.13 (m, 2H), 4.04 (d, *J* = 10.2 Hz, 1H), 3.61 (d, *J* = 10.8 Hz, 2H), 3.30 (d, *J* = 10.8 Hz, 2H), 2.58 (s, 6H), 2.30 (s, 3H), 1.33 (t, *J* = 7.1 Hz, 3H), 1.25 (s, 3H), 1.09 (s, 3H), 0.94 (s, 3H).

**$^{13}$C NMR** (126 MHz, $CDCl_3$) δ 172.3, 141.1, 137.9, 136.8, 130.8, 61.9, 61.8, 52.8, 39.6, 29.7, 22.7, 21.2, 21.0, 19.2, 14.1.

***R*$_f$** = 0.36 (hexane/EtOAc 9:1)

**HRMS** (ESI): calcd for $C_{17}H_{26}ClNaNO_3S$ $[M + Na]^+$: 382.1214, found: 382.1219.

Compound **5p** (*tert*-butyl (*S*)-2-((*R*)-4-ethoxy-3-(((*R*)-mesitylsulfinyl)amino)-2-methyl-4-oxobutan-2-yl)-4,4-difluoropyrrolidine-1-carboxylate)

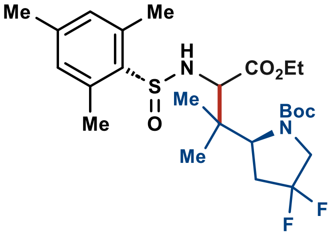


Synthesized according to the general procedure described in **Section 4.2** on 0.36 mmol scale from methyl oxalate **2p** with 4 h reaction time. The crude product was purified by column chromatography using petroleum ether/EtOAc 5:1 → 2:1 as eluent. The product is a brownish oil 50.0 mg, **32% yield**).

**$^1$H NMR** (500 MHz, $CDCl_3$) δ 6.88 (s, 2H), 5.11 (d, *J* = 9.9 Hz, 1H), 4.37 – 4.19 (m, 3H), 4.13 (t, *J* = 8.9 Hz, 1H), 3.66 (br.s, 1H), 3.50 (ddd, J = 25.3, 12.8, 6.1 Hz, 1H),

3.36 (ddd, J = 24.9, 13.3, 6.1 Hz, 1H), 2.59 (s, 6H), 2.29 (s, 3H), 1.48 (s, 6H), 1.43 (s, 9H), 1.34 (t, $J$ = 7.1 Hz, 3H).

**$^{13}$C NMR** (126 MHz, $CDCl_3$) δ 171.8, 171.7, 141.1, 136.8, 131.9, 130.8, 81.8, 65.3, 62.8, 61.8, 54.3, 43.7, 28.2, 28.2, 21.0, 19.3, 14.1.

***R*f** = 0.50 (hexanes/EtOAc 2:1)

**HRMS** (ESI): calcd for $C_{25}H_{38}NaF_2N_2O_5S$ $[M + Na]^+$: 539.2378, found: 539.2387.

Compound **5q** (ethyl (2*R*)-2-(((*R*)-mesitylsulfinyl)amino)-3-methyl-3-(5-methyl-5-vinyltetrahydrofuran-2-yl)butanoate)

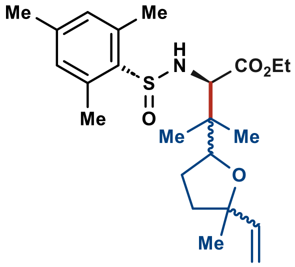


Synthesized according to the general procedure described in **Section 4.2** on 0.36 mmol scale from methyl oxalate **2q** with 2 h reaction time. The crude product was purified by column chromatography using petroleum ether/EtOAc 10:1 → 3:1 as eluent. The product is a brownish oil (58 mg, **46% yield**).

**$^{1}$H NMR** (500 MHz, $CDCl_3$) δ 6.87 (s, 2H, CH, Mes), 6.84 (s, 2H, CH, Mes), 5.84 (dd, $J$ = 17.2, 10.7 Hz, 1H, H-6), 5.80 (dd, $J$ = 17.2, 10.7 Hz, 1H, H-6), 5.40 (d, $J$ = 10.4 Hz, 1H, NH), 5.24 (dd, $J$ = 17.2, 1.5 Hz, 1H, H-7b), 5.20 (d, $J$ = 9.8 Hz, 1H, NH), 5.20 (dd, $J$ = 17.2, 1.7 Hz, 1H, H-7b), 4.96 (dd, $J$ = 8.2, 1.5 Hz, 1H, H-7a), 4.94 (dd, $J$ = 8.1, 1.7 Hz, 1H, H-7a), 4.25–4.14 (m, 4H, H-10), 3.99 (d, $J$ = 10.2 Hz, 1H, H-8), 3.97 (dd, $J$ = 9.2, 7.3 Hz, 1H, H-2), 3.93 (d, $J$ = 9.6 Hz, 1H, H-8), 3.83 (t, $J$ = 7.2 Hz, 1H, H-2), 2.571 (s, 6H, 2 × $CH_3$, Mes), 2.573 (s, 6H, 2 × $CH_3$, Mes), 2.29 (s, 3H, $CH_3$, Mes), 2.27 (s, 3H, $CH_3$, Mes), 1.87–1.58 (m, 8H, H-3, H-4), 1.30 (t, $J$ = 7.2 Hz, 6H, H-11), 1.27 (s, 3H, $CH_3$), 1.22 (s, 3H, $CH_3$), 1.01 (s, 3H, $CH_3$), 0.92 (s, 3H, $CH_3$), 0.85 (s, 3H, $CH_3$), 0.82 (s, 3H, $CH_3$).

**$^{13}$C NMR** (126 MHz, $CDCl_3$) δ 172.8, 172.6 (C-9), 143.9, 143.8 (C-6), 140.8, 140.5 (C, Mes), 138.4, 138.1 (C, Mes), 136.8, 136.7 (2 × C, Mes), 130.8, 130.7 (2 × CH, Mes), 111.2, 111.1 (C-7), 82.8, 82.7 (C-5), 81.8, 81.5 (C-2), 64.2, 63.6 (C-8), 61.4, 61.1 (C-10), 41.1, 40.2 (C-1), 37.2, 37.1 (C-3 or C-4), 26.96, 27.01 ($CH_3$), 26.4, 26.2 (C-3 or C-4), 21.0, 21.0 ($CH_3$, Mes), 20.2, 20.0 ($CH_3$), 19.4, 19.3 (2 × $CH_3$, Mes), 19.1, 18.5 ($CH_3$), 14.1 (C-11).

***R*f** = 0.49, 0.47 (petroleum ether/EtOAc 5:1)

**HRMS** (ESI): calcd for $C_{23}H_{35}NaNO_4S$ $[M + Na]^+$: 444.2179, found: 444.2181.

Compound **5r** (ethyl (*R*)-5-((tert-butyldimethylsilyl)oxy)-2-(((*R*)-mesitylsulfinyl)amino)-3,3-dimethylpentanoate)

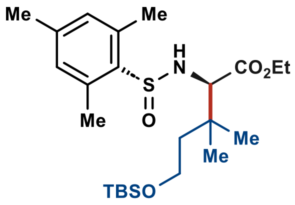


Synthesized according to the general procedure described in **Section 4.2** on 0.36 mmol scale from methyl oxalate **2r** with 2 h reaction time. The crude product was purified by column chromatography using petroleum ether/EtOAc 20:1 → 10:1 as eluent. The product is a brownish oil (124 mg, **88% yield**).

**$^1$H NMR** (500 MHz, $CDCl_3$) δ 6.87 (s, 2H), 5.07 (d, *J* = 10.1 Hz, 1H), 4.31–4.11 (m, 2H), 3.74 (d, *J* = 10.1 Hz, 1H), 3.71 (ddd, *J* = 7.6, 6.6, 1.3 Hz, 2H), 2.57 (s, 6H), 2.29 (s, 3H), 1.63 (dt, *J* = 14.3, 7.1 Hz, 1H), 1.57–1.50 (m, 1H), 1.31 (t, *J* = 7.1 Hz, 3H), 0.97 (s, 3H), 0.94 (s, 3H), 0.89 (s, 9H), 0.05 (s, 6H).

**$^{13}$C NMR** (126 MHz, $CDCl_3$) δ 172.6, 140.9, 138.0, 136.8, 130.8, 65.4, 61.3, 59.5, 41.2, 36.9, 25.9, 24.1, 23.8, 21.0, 19.3, 18.2, 14.1, −5.35, −5.38.

***R*$_f$** = 0.23 (petroleum ether/EtOAc 10:1)

**HRMS** (ESI): calcd for $C_{24}H_{43}NaNO_4SSi$ $[M + Na]^+$: 492.2574, found: 492.2575.

Compound **5s** ((*R*)-5-ethoxy-4-(((*R*)-mesitylsulfinyl)amino)-3,3-dimethyl-5-oxopentyl benzoate)

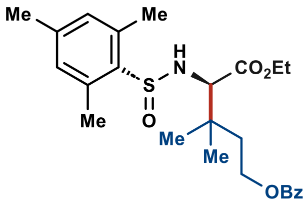


Synthesized according to the general procedure described in **Section 4.2** on 0.3 mmol scale from methyl oxalate **2s** with 2 h reaction time. The crude product was purified by column chromatography using petroleum ether/EtOAc 20:1 → 5:1 as eluent. The product is a brownish oil (106 mg, **77% yield**).

**$^1$H NMR** (500 MHz, $CDCl_3$) δ 8.07–7.96 (m, 2H), 7.64–7.51 (m, 1H), 7.44 (t, *J* = 7.7 Hz, 2H), 6.87 (s, 2H), 5.10 (d, *J* = 10.0 Hz, 1H), 4.42 (qt, *J* = 11.1, 7.0 Hz, 2H), 4.34–4.15 (m, 2H), 3.76 (d, *J* = 10.0 Hz, 1H), 2.57 (s, 6H), 2.29 (s, 3H), 1.84 (td, *J* = 7.2, 2.5 Hz, 2H), 1.38–1.28 (m, 3H), 1.06 (s, 3H), 1.04 (s, 3H).

**$^{13}$C NMR** (126 MHz, $CDCl_3$) δ 172.3, 166.5, 141.0, 137.8, 136.8, 132.9, 130.8, 130.2, 129.5, 128.4, 65.5, 61.6, 37.1, 37.0, 24.1, 23.7, 21.0, 19.3, 14.1.

**HRMS** (ESI): calcd for $C_{25}H_{33}NaNO_5S$ $[M + Na]^+$: 482.1972, found: 482.1972.

Compound **5t** ((*R*)-5-ethoxy-4-(((*R*)-mesitylsulfinyl)amino)-3,3-dimethyl-5-oxopentyl 4-bromobenzoate)

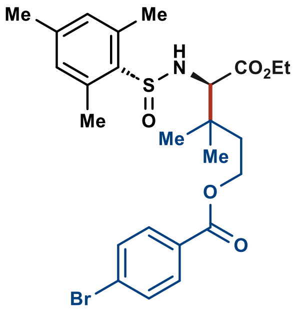


Synthesized according to the general procedure described in **Section 4.2** on 0.17 mmol scale from methyl oxalate **2t** (1.5 equiv.) with 4 h reaction time. The crude product was purified by column chromatography using petroleum ether/EtOAc 5:1 → 3:1 as eluent. The product is a brownish oil (46.2 mg, **58% yield**).

**$^1$H NMR** (500 MHz, $CDCl_3$) δ 7.92–7.82 (m, 2H), 7.64–7.53 (m, 2H), 6.87 (s, 2H), 5.10 (d, *J* = 10.0 Hz, 1H), 4.47–4.35 (m, 2H), 4.34–4.18 (m, 2H), 3.75 (d, *J* = 10.0 Hz, 1H), 2.56 (s, 6H), 2.29 (s, 3H), 1.83 (td, *J* = 7.2, 3.3 Hz, 2H), 1.31 (t, *J* = 7.2 Hz, 3H), 1.04 (d, *J* = 7.2 Hz, 6H).

**$^{13}$C NMR** (126 MHz, $CDCl_3$) δ 172.3, 165.8, 141.1, 137.8, 136.8, 131.7, 131.1, 130.9, 129.1, 128.1, 65.4, 61.9, 61.6, 37.0, 37.0, 24.1, 23.7, 21.1, 19.3, 14.2.

***R*f** = 0.48 (petroleum ether/EtOAc 3:1)

**HRMS** (ESI): calcd for $C_{25}H_{32}BrNaNO_5S$ $[M + Na]^+$: 560.1077, found: 560.1078.

Compound **5u** ((*R*)-5-ethoxy-4-(((*R*)-mesitylsulfinyl)amino)-3,3-dimethyl-5-oxopentyl nicotinate)

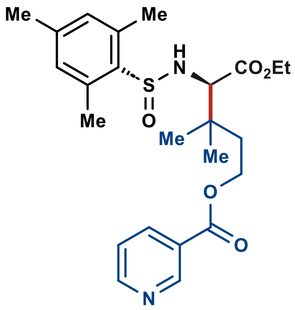


Synthesized according to the general procedure described in **Section 4.2** on 0.36 mmol scale from methyl oxalate **2u** with 2 h reaction time. The crude product was purified by column chromatography using petroleum ether/EtOAc 5:1 → 3:1 as eluent. The product is a brownish oil (98 mg, **71% yield**).

**$^{1}$H NMR** (500 MHz, $CDCl_3$) δ 9.20 (s, 1H), 8.78 (dd, *J* = 4.8, 1.9 Hz, 1H), 8.28 (dt, *J* = 8.0, 1.9 Hz, 1H), 7.40 (dd, *J* = 8.0, 4.8 Hz, 1H), 6.87 (s, 2H), 5.11 (d, *J* = 10.0 Hz, 1H), 4.51–4.39 (m, 2H), 4.34–4.17 (m, 2H), 3.74 (d, *J* = 10.0 Hz, 1H), 2.57 (s, 6H), 2.29 (s, 3H), 1.90–1.80 (m, 2H), 1.32 (t, *J* = 7.1 Hz, 3H), 1.06 (s, 3H), 1.05 (s, 3H).

**$^{13}$C NMR** (126 MHz, $CDCl_3$) δ 172.3, 165.1, 153.4, 150.8, 141.1, 137.8, 137.1, 136.8, 130.9, 126.1, 123.3, 65.4, 62.1, 61.7, 37.0, 37.0, 24.1, 23.7, 21.0, 19.3, 14.2.

***R*f** = 0.32 (petroleum ether/EtOAc 1:1)

**HRMS** (ESI): calcd for $C_{24}H_{32}NaN_2O_5S$ $[M + Na]^+$: 483.1924, found: 483.1924.

Compound **5v** ((*R*)-5-ethoxy-4-(((*R*)-mesitylsulfinyl)amino)-3,3-dimethyl-5-oxopentyl thiophene-2-carboxylate)

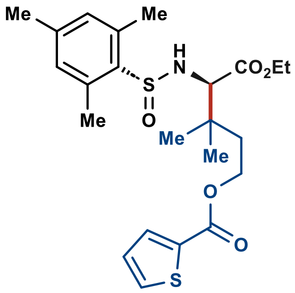


Synthesized according to the general procedure described in **Section 4.2** on 0.36 mmol scale from methyl oxalate **2v** with 2 h reaction time. The crude product was purified by column chromatography using petroleum ether/EtOAc 5:1 → 3:1 as eluent. The product is a brownish oil (81 mg, **58% yield**).

**$^{1}$H NMR** (500 MHz, $CDCl_3$) δ 8.13 (dd, *J* = 3.7, 1.3 Hz, 1H), 7.89 (dd, *J* = 5.0, 1.3 Hz, 1H), 7.44 (dd, *J* = 5.0, 3.7 Hz, 1H), 7.21 (s, 2H), 5.44 (d, *J* = 10.0 Hz, 1H), 4.79–4.66 (m, 2H), 4.68–4.51 (m, 2H), 4.09 (d, *J* = 10.0 Hz, 1H), 2.91 (s, 6H), 2.63 (s, 3H), 2.22–2.07 (m, 2H), 1.91 (s, 6H), 1.65 (t, *J* = 7.1 Hz, 3H), 1.39 (s, 3H), 1.37 (s, 3H).

**$^{13}$C NMR** (126 MHz, $CDCl_3$) δ 172.3, 162.1, 141.0, 137.9, 136.8, 133.8, 133.4, 132.4, 130.8, 127.8, 65.4, 61.8, 61.6, 37.0, 37.0, 24.1, 23.8, 23.7, 21.0, 19.3, 14.1.

***R*f** = 0.60 (petroleum ether/EtOAc 2:1)

**HRMS** (ESI): calcd for $C_{23}H_{31}NaNO_5S_2$ $[M + Na]^+$: 488.1536, found: 488.1535.

Compound **5w** (ethyl (*R*)-2-(((*R*)-mesitylsulfinyl)amino)-3,3-dimethyl-5-(((3a*R*,4*R*,5a*R*,9a*R*,9b*S*)-2,2,8,8-tetramethylhexahydro-[1,3]dioxolo[4',5':4,5]pyrano[3,2-*d*][1,3]dioxin-4-yl)oxy)pentanoate)

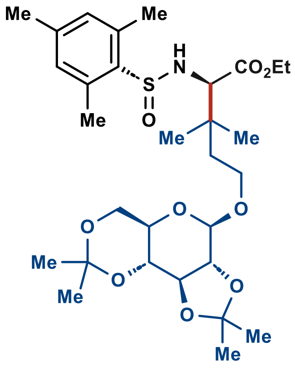


Synthesized according to the general procedure described in **Section 4.2** on 0.36 mmol scale from methyl oxalate **2w** with 2 h reaction time. The crude product was purified by column chromatography using petroleum ether/EtOAc 3:1 → 2:1 as eluent. The product is a brownish oil (132 mg, **74% yield**).

**$^1$H NMR** (500 MHz, $CDCl_3$) δ 6.87 (s, 2H), 5.04 (d, *J* = 10.2 Hz, 1H), 4.67 (d, *J* = 7.9 Hz, 1H), 4.23 (ABqq, *J* = 10.8, 7.1 Hz, 2H), 4.01–3.92 (m, 2H), 3.93–3.84 (m, 2H), 3.70 (d, *J* = 10.2 Hz, 1H), 3.70–3.64 (m, 1H), 3.63 (dd~t, *J* = 9.0 Hz, 1H), 3.38 (dd, *J* = 9.0, 7.9 Hz, 1H), 3.26 (ddd, *J* = 10.3, 8.8, 5.2 Hz, 1H), 2.56 (s, 6H), 2.29 (s, 3H), 1.75 (ddd, *J* = 13.7, 9.1, 6.2 Hz, 1H), 1.67 (ddd, *J* = 13.7, 9.1, 5.7 Hz, 1H), 1.54 (s, 3H), 1.46 (s, 6H), 1.44 (s, 3H), 1.31 (t, *J* = 7.1 Hz, 3H), 0.97 (s, 3H), 0.95 (s, 3H).

**$^{13}$C NMR** (126 MHz, $CDCl_3$) δ 172.4, 140.9, 137.9, 136.7, 130.8, 112.1, 102.3 (C-1), 99.7, 77.8, 77.6, 72.7, 69.7, 66.1, 65.2, 62.2, 61.5, 37.8, 36.8, 29.0, 26.7, 26.4, 24.1, 23.8, 21.0, 19.3, 19.1, 14.1.

***$R_f$*** = 0.47 (petroleum ether/EtOAc 2:1)

**HRMS** (ESI): calcd for $C_{30}H_{47}NaNO_9S$ $[M + Na]^+$: 620.2864, found: 620.2861.

Compound **6a** ((1*R*,3*r*,5*S*)-3-allyl-8-(*tert*-butoxycarbonyl)-8-azabicyclo[3.2.1]octan-3-yl methyl oxalate)

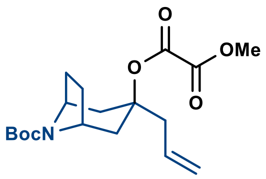


Synthesized according to the **General procedure C** described in **Section 4.1** on 1 mmol scale from alcohol **SI-3** with 2 h reaction time. The crude product was purified by column chromatography using petroleum ether/EtOAc 10:1 → 4:1 as eluent. The product is a colorless oil (140 mg, **40% yield**).

**$^1$H NMR** (500 MHz, $CDCl_3$) δ 5.66 (ddt, *J* = 17.5, 10.2, 7.4 Hz, 1H), 5.18–5.03 (m, 2H), 4.26 (br.s, 1H), 4.16 (br.s, 1H), 3.88 (s, 3H), 2.67 (dd, *J* = 12.8, 7.1 Hz, 2H), 2.38 (dd~t, *J* = 15.3 Hz, 2H), 2.07–1.82 (m, 6H), 1.46 (s, 9H).

**$^{13}$C NMR** (126 MHz, $CDCl_3$) δ 158.6, 156.4, 153.3, 131.0, 119.9, 87.1, 79.5, 53.4, 52.7 (br), 52.0 (br), 43.9, 38.8 (br), 38.2 (br), 28.5, 27.9 (br), 27.1 (br).

***$R_f$*** = 0.40 (petroleum ether/EtOAc 5:1)

The spectroscopic data is in agreement with the literature.[11]

Compound **6b** ((1*r*,3*r*,5*r*,7*r*)-2-allyladamantan-2-yl methyl oxalate)

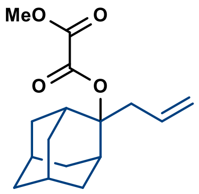


Synthesized according to the **General procedure C** described in **Section 4.1** on 1.8 mmol scale from alcohol **SI-4** with 10 min reaction time. The crude product was purified by column chromatography using petroleum ether/EtOAc 10:1 ⟶ 4:1 as eluent. The product is a colorless oil (330 mg, **66% yield**).

**$^{1}$H NMR** (500 MHz, $CDCl_3$) δ 5.81–5.68 (m, 1H), 5.12–5.05 (m, 2H), 3.87 (s, 3H), 2.97 (d, *J* = 7.3 Hz, 2H), 2.45 (s, 2H), 2.11–1.98 (m, 2H), 1.95–1.82 (m, 4H), 1.82–1.71 (m, 4H), 1.66–1.59 (m, 2H).

**$^{13}$C NMR** (126 MHz, $CDCl_3$) δ 159.0, 156.2, 131.9, 118.5, 93.2, 53.2, 38.1, 36.8, 34.2, 33.9, 32.9, 27.0, 26.7.

***R*f** = 0.50 (petroleum ether/EtOAc 10:1)

**HRMS** (ESI): calcd for $C_{16}H_{22}NaO_4$ $[M + Na]^+$: 301.1410, found: 301.1410.

Compound **6c** (2-allyl-2,3-dihydro-1*H*-inden-2-yl methyl oxalate)

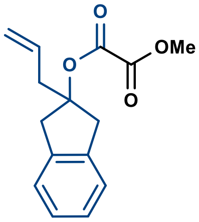


Synthesized according to the **General procedure C** described in **Section 4.1** on 1.4 mmol scale from alcohol **SI-5** with 10 min reaction time. The crude product was purified by column chromatography using petroleum ether/EtOAc 20:1 ⟶ 10:1 as eluent. The product is a colorless amorphous solid (354 mg, **97% yield**).

**$^{1}$H NMR** (500 MHz, $CDCl_3$) δ 7.23–7.15 (m, 4H), 5.86–5.73 (m, 1H), 5.20–5.08 (m, 2H), 3.85 (s, 3H), 3.49 (d, *J* = 16.9 Hz, 2H), 3.33 (d, *J* = 16.9 Hz, 2H), 2.90 (dt, *J* = 7.3, 1.3 Hz, 2H).

**$^{13}$C NMR** (126 MHz, $CDCl_3$) δ 158.4, 156.8, 139.5, 131.9, 126.9, 124.5, 119.3, 93.8, 53.3, 43.7, 40.7.

***R*f** = 0.29 (petroleum ether/EtOAc 20:1, brown color upon treatment with 5% $H_3PO_4$ in MeOH)

**HRMS** (ESI): calcd for $C_{15}H_{16}NaO_4$ $[M + Na]^+$: 283.0941, found: 283.0942.

Compound **6d** ((*R*)-1-isopropyl-4-methylcyclohex-3-en-1-yl methyl oxalate, (−)-terpineolyl methyloxalate)

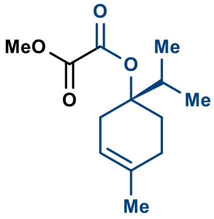


Synthesized according to the **General procedure C** described in **Section 4.1** on 1 mmol scale from (−)-terpinen-4-ol with 1 h reaction time. The crude product was purified by filtration through silica gel washing with $CH_2Cl_2$. The product is a yellowish oil (240 mg, **quantitative yield**).

**$^{1}$H NMR** (500 MHz, $CDCl_3$) δ 5.22 (br.s, 1H), 3.82 (s, 3H), 2.68 (hept, *J* = 6.9 Hz, 1H), 2.52–2.40 (m, 2H), 2.28–2.15 (m, 1H), 2.05–1.95 (m, 1H), 1.90 (dd, *J* = 17.8, 5.9 Hz, 1H), 1.70 (ddd, *J* = 13.4, 11.5, 6.1 Hz, 1H), 1.63 (br.s, 3H), 0.92 (d, *J* = 6.9 Hz, 3H), 0.91 (d, *J* = 6.9 Hz, 3H).

**$^{13}$C NMR** (126 MHz, $CDCl_3$) δ 159.1, 157.1, 133.7, 117.0, 91.2, 53.2, 32.6, 29.7, 27.7, 27.1, 23.1, 17.6, 17.0.

$R_f$ = 0.71 (petroleum ether/EtOAc 5:1)

Compound **7a** (*tert*-butyl 4'-((*R*)-3-ethoxy-2-(((*R*)-mesitylsulfinyl)amino)-3-oxopropyl)-5'-oxodihydro-3'*H*-8-azaspiro[bicyclo[3.2.1]octane-3,2'-furan]-8-carboxylate)

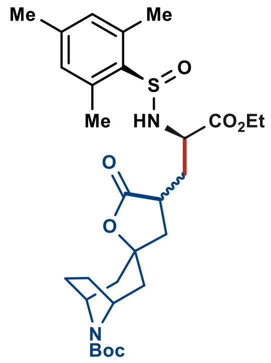


Synthesized according to the general procedure described in **Section 4.2** on 0.36 mmol scale from methyl oxalate **6a** with 4 h reaction time. The crude product was purified by column chromatography using petroleum ether/EtOAc 5:1 → 1:1 as eluent. The product is a brownish oil (43 mg, **28% yield**). The product is a mixture of two γ-diastereomers (1:1).

**$^1$H NMR** (500 MHz, $CDCl_3$) δ 6.88 (s, 2H), 6.87 (s, 2H), 5.13 (d, *J* = 8.9 Hz, 0.5H), 5.11 (d, *J* = 8.5 Hz, 0.5H), 4.32–4.25 (m, 1.5H), 4.24–4.16 (m, 3H), 3.92–3.84 (m, 0.5H), 2.97–2.75 (m, 1H), 2.58 (s, 6H), 2.57–2.50 (m, 1H), 2.38 (d, *J* = 13.8 Hz, 0.5H), 2.29 (s, 1.5H), 2.29 (s, 1.5H), 2.26–2.16 (m, 1H), 2.17–2.00 (m, 3.5H), 1.97–1.89 (m, 3H), 1.89–1.82 (m, 3H), 1.81–1.70 (m, 1.5H), 1.67–1.57 (m, 1.5H), 1.48 (s, 4.5H), 1.46 (s, 4.5H), 1.32–1.25 (m, 3H). *Mixture of two γ-diastereomers is reported.*

**$^{13}$C NMR** (126 MHz, $CDCl_3$) δ 177.5, 177.5, 172.7, 172.4, 153.3, 153.2, 141.3, 141.2, 137.3 (d, *J* = 21.5 Hz), 136.8, 136.6, 131.0, 130.9, 82.5, 82.3, 79.7, 79.6, 62.2, 62.0, 55.3, 55.1, 43.7, 43.4, 36.2, 35.7, 34.6, 28.5, 28.5, 21.0, 19.3, 14.1, 14.1. *Mixture of two γ-diastereomers is reported.*

$R_f$ = 0.45 (petroleum ether/EtOAc 1:1)

**HRMS** (ESI): calcd for $C_{29}H_{42}NaN_2O_7S$ $[M + Na]^+$: 585.2605, found: 585.2603.

Compound **7b** (ethyl (*R*)-2-(((*R*)-mesitylsulfinyl)amino)-3-((1*R*,3*R*)-5'-oxodihydro-3'*H*-spiro[adamantane-2,2'-furan]-4'-yl)propanoate)

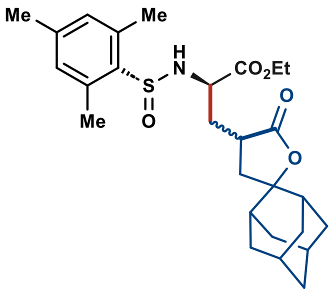


Synthesized according to the general procedure described in **Section 4.2** on 0.36 mmol scale from methyl oxalate **6b** with 4 h reaction time. The crude product was purified by column chromatography using petroleum ether/EtOAc 5:1 → 2:3 as eluent. The product is a brownish oil (35 mg, **24% yield**). The product is a mixture of two γ-diastereomers (1:1).

**$^1$H NMR** (500 MHz, $CDCl_3$) δ 6.87 (s, 2H), 5.15 (d, *J* = 8.9 Hz, 0.5H), 5.13 (d, *J* = 8.4 Hz, 0.5H), 4.32 (td, *J* = 7.9, 5.5 Hz, 0.5H), 4.28–4.17 (m, 2H), 2.98 (tdd, *J* = 11.7, 8.8, 3.8 Hz, 0.5H), 2.88 (dtd, *J* = 11.6, 8.5, 5.9 Hz, 0.5H), 2.75 (dd, *J* = 12.6, 8.9 Hz, 0.5H), 2.69–2.61 (m, 0.5H), 2.58 (s, 3H), 2.58 (s, 3H), 2.39 (dt, *J* = 14.2, 5.8 Hz, 0.5H), 2.29 (s, 3H), 2.26–2.18 (m, 1.5H), 2.18–2.11 (m, 1H), 1.92–1.69 (m, 11H), 1.68–1.58 (m, 5H), 1.29 (t, *J* = 7.1, Hz, 1.5H), 1.27 (t, *J* = 7.1, Hz, 1.5H). *Mixture of two γ-diastereomers is reported.*

**$^{13}$C NMR** (126 MHz, $CDCl_3$) δ 177.60, 177.52, 172.90, 172.60, 141.27, 141.10, 137.38, 136.81, 136.52, 130.97, 130.87, 88.70, 88.49, 62.13, 61.95, 55.43, 55.31, 39.28, 39.26, 38.28, 37.87, 37.35, 37.01, 36.48, 35.56, 35.49, 35.38, 35.28, 35.16, 33.76, 33.63, 33.60, 32.72, 32.70, 26.69, 26.67, 26.60, 21.03, 19.31, 19.24, 14.10, 14.08. *Mixture of two γ-diastereomers is reported.*

$R_f$ = 0.48 (petroleum ether/EtOAc 2:1)

**HRMS** (ESI): calcd for $C_{27}H_{37}NaNO_5S$ [M + Na]$^+$: 510.2285, found: 510.2284.

Compound **7c** (ethyl (*R*)-2-(((*R*)-mesitylsulfinyl)amino)-3-(5-oxo-1',3',4,5-tetrahydro-3*H*-spiro[furan-2,2'-inden]-4-yl)propanoate)

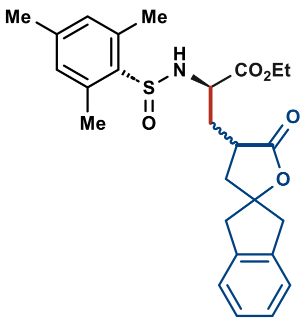

Synthesized according to the general procedure described in **Section 4.2** on 0.36 mmol scale from methyl oxalate **6c** with 4 h reaction time. The crude product was purified by column chromatography using petroleum ether/EtOAc 5:1 → 2:3 as eluent. The product is a brownish oil (40 mg, **29% yield**). The product is a mixture of two γ-diastereomers (1:1).

**$^1$H NMR** (500 MHz, $CDCl_3$) δ 7.25–7.15 (m, 4H), 6.89 (s, 1H), 6.88 (s, 1H), 5.16 (d, *J* = 9.2 Hz, 0.5H), 5.14 (d, *J* = 8.1 Hz, 0.5H), 4.39–4.30 (m, 1H), 4.22 (qd, *J* = 7.2, 4.3 Hz, 2H), 3.94 (ddd, *J* = 10.9, 9.1, 4.1 Hz, 0.5H), 3.40 (dd, *J* = 16.6, 14.3 Hz, 1H), 3.29–3.18 (m, 1H), 3.15 (d, *J* = 15.4 Hz, 0.5H), 3.13–3.05 (m, 1.5H), 3.05–2.97 (m, 0.5H), 2.69–2.62 (m, 0.5H), 2.60 (s, 3H), 2.59 (s, 3H), 2.58–2.49 (m, 1H), 2.49–2.42 (m, 1H), 2.30 (s, 1.5H), 2.29 (s, 1.5H), 2.28–2.23 (m, 1H), 2.13 (t, *J* = 12.3 Hz, 0.5H), 2.08–2.01 (m, 0.5H), 1.96 (ddd, *J* = 14.4, 10.6, 4.1 Hz, 0.5H), 1.87 (dt, *J* = 14.2, 8.0 Hz, 0.5H), 1.29 (d, *J* = 7.1, 4.5 Hz, 1.5H), 1.26 (d, *J* = 7.1, 4.5 Hz, 1.5H). *Mixture of two γ-diastereomers is reported.*

**$^{13}$C NMR** (126 MHz, $CDCl_3$) δ 177.33, 177.33, 172.76, 172.48, 141.32, 141.16, 139.51, 139.49, 139.36, 139.28, 137.44, 137.24, 136.81, 136.60, 131.00, 130.94, 130.91, 127.23, 127.16, 127.11, 124.62, 124.60, 124.47, 124.42, 91.29, 91.17, 62.15, 62.02, 55.34, 55.15, 45.35, 44.88, 44.79, 39.95, 39.59, 37.86, 37.27, 34.75, 34.50, 21.03, 19.30, 14.08, 14.06. *Mixture of two γ-diastereomers is reported.*

$R_f$ = 0.51 (petroleum ether/EtOAc 1:1)

**HRMS** (ESI): calcd for $C_{26}H_{31}NaNO_5S$ [M + Na]$^+$: 492.1815, found: 492.1815.

Compound **7d** (ethyl (2*R*)-2-((1*S*,2*R*)-5-isopropyl-2-methyl-7-oxo-6-oxabicyclo[3.2.1]octan-2-yl)-2-(((*R*)-mesitylsulfinyl)amino)acetate)

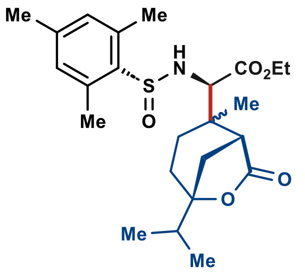

Synthesized according to the general procedure described in **Section 4.2** on 0.3 mmol scale from methyl oxalate **6d** with 2 h reaction time. The crude product was purified by column chromatography using hexane/EtOAc 9:1 as eluent. The product is a brown solid (17.5 mg, **13% yield**). The product is a mixture of two β-diastereomers (1:3.5).

**$^1$H NMR** (500 MHz, $CDCl_3$) δ 6.87 (s, 2H), 5.12 (d, *J* = 10.1 Hz, 1H), 4.34–4.20 (m, 2H), 4.18 (d, *J* = 10.1 Hz, 1H), 2.64 (dd, *J* = 5.6, 1.8 Hz, 1H), 2.53 (s, 6H), 2.28 (s, 3H), 2.16–2.05 (m, 2H), 2.01–1.92 (m, 2H), 1.83–1.74 (m, 2H), 1.43 (dt, *J* = 15.1, 9.7 Hz, 1H), 1.32 (d, *J* = 7.1 Hz, 3H), 1.00 (d, *J* = 6.8 Hz, 3H), 0.97 (d, *J* = 6.9 Hz, 3H), 0.90 (s, 1H). *Mixture of two β-diastereomers is reported.*

**$^{13}$C NMR** (126 MHz, $CDCl_3$) δ 177.3, 171.6, 141.5, 137.2, 136.7, 131.0, 89.1, 62.17, 62.03, 59.1, 48.8, 38.5, 38.1, 35.1, 35.1, 34.9, 34.2, 29.3, 26.0, 21.1, 20.0,

19.28, 19.17, 17.1, 16.8, 14.2, 14.1. *Mixture of two β-diastereomers is reported.*

***R*f** = 0.19 (hexane/EtOAc 9:1)

**HRMS** (ESI): calcd for $C_{24}H_{35}NaNO_5S$ $[M + Na]^+$: 472.2128, found: 472.2127.

## Compound **8a** (ethyl (*R*)-2-amino-2-(1-methylcyclohexyl)acetate, trifluoroacetate salt)

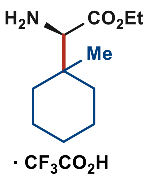


Synthesized according to the general procedure described in **Section 4.2** on 0.1 mmol scale from *N*-sulfinyl amide **5a**. The product is a colorless solid (31 mg, **99% yield**).

**$^1$H NMR** (500 MHz, MeOD) δ 4.30 (ABqq, *J* = 16.0, 7.1, Hz, 2H), 3.88 (s, 1H), 1.70–1.40 (m, 10H), 1.32 (t, *J* = 7.1 Hz, 3H), 1.04 (s, 3H).

**$^{13}$C NMR** (126 MHz, MeOD) δ 169.6, 63.4, 62.2, 37.1, 36.0, 35.2, 26.6, 22.4, 22.3, 19.9, 14.4.

**$^{19}$F NMR** (377 MHz, MeOD) δ -77.0.

***R*f** = 0.80 ($CH_2Cl_2$/MeOH 10:1, red color upon treatment with ninhydrin stain)

**$[\alpha]^{25}_D$** = −12.8 (*c* 3.4, MeOH)

**HRMS** (ESI): calcd for $C_{11}H_{22}NO_2$ $[M-CF_3CO_2H+H]^+$: 200.1645, found: 200.1644.

## Compound **8d** (2-((1*R*,2*R*,4a*S*,8a*S*)-2-((*R*)-1-amino-2-ethoxy-2-oxoethyl)-2,5,5,8a-tetramethyldecahydronaphthalen-1-yl)ethyl benzoate)

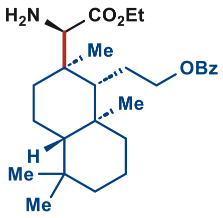


Synthesized according to the general procedure described in **Section 4.2** on 0.1 mmol scale from *N*-sulfinyl amide **5d**. The product is a colorless solid (44 mg, **99% yield**).

**$^1$H NMR** (500 MHz, $CDCl_3$) δ 8.04–7.97 (m, 2H), 7.59–7.51 (m, 1H), 7.42 (dd~t, *J* = 7.6 Hz, 2H), 4.52 (td, *J* = 11.0, 5.4 Hz, 1H), 4.38–4.27 (m, 1H), 4.30–4.17 (m, 2H), 4.15 (s, 1H), 1.84 (tt, *J* = 9.6, 4.8 Hz, 1H), 1.81–1.74 (m, 2H), 1.74–1.63 (m, 1H), 1.60 (dt, *J* = 13.4, 6.7 Hz, 2H), 1.54–1.41 (m, 2H), 1.39 (dd, *J* = 13.4, 3.4 Hz, 1H), 1.34 (m, 1H), 1.30 (t, *J* = 7.0 Hz, 3H), 1.21 (dd~t, *J* = 12.6 Hz, 1H), 1.15 (s, 3H), 1.11 (dd, *J* = 13.6, 4.2 Hz, 1H), 0.95 (s, 3H), 0.89 (ddd, *J* = 12.8, 9.0, 3.7 Hz, 1H), 0.84 (s, 3H), 0.79 (s, 3H), 0.73 (d, *J* = 11.5 Hz, 1H).

**$^{13}$C NMR** (126 MHz, $CDCl_3$) δ 168.5, 167.3, 133.2, 129.9, 129.5, 128.4, 65.9, 63.3, 61.3, 56.1, 50.1, 41.7, 41.6, 40.6, 39.6, 35.1, 33.1, 33.1, 25.5, 21.5, 18.1, 17.7, 17.6, 16.5, 13.8.

***R*f** = 0.80 ($CH_2Cl_2$/MeOH 10:1, red color upon treatment with ninhydrin stain)

**$[\alpha]^{25}_D$** = −0.8 (*c* 4.0, MeOH)

**HRMS** (ESI): calcd for $C_{27}H_{42}NO_4$ $[M + H]^+$: 444.3108, found: 444.3109.

Compound **8f** (ethyl (*R*)-2-amino-2-(1-benzoylcyclohexyl)acetate, trifluoroacetate salt)

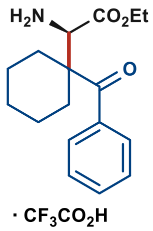


Synthesized according to the general procedure described in **Section 4.2** on 0.07 mmol scale from *N*-sulfinyl amide **5f**. The product is a colorless solid (28 mg, **99% yield**).

**$^{1}$H NMR** (500 MHz, MeOD) δ 7.76–7.64 (m, 2H), 7.65–7.52 (m, 1H), 7.49 (dd~t, *J* = 7.6 Hz, 2H), 4.65 (s, 1H), 4.26 (q, *J* = 7.1 Hz, 2H), 2.42–2.29 (m, 1H), 2.29–2.13 (m, 1H), 1.93 (ddd, *J* = 13.5, 9.6, 3.4 Hz, 1H), 1.67 (ddd, *J* = 13.3, 9.0, 3.7 Hz, 1H), 1.64–1.27 (m, 6H), 1.23 (t, *J* = 7.1 Hz, 3H).

**$^{13}$C NMR** (126 MHz, MeOD) δ 207.0, 169.0, 139.8, 132.7, 129.5, 128.8, 64.0, 58.0, 55.3, 32.7, 31.2, 26.1, 23.1, 23.0, 14.1.

**$^{19}$F NMR** (377 MHz, MeOD) δ -77.0.

***$R_f$*** = 0.80 ($CH_2Cl_2$/MeOH 10:1, red color upon treatment with ninhydrin stain)

**$[\alpha]^{25}_{D}$** = −20.1 (*c* 3.4, MeOH)

**HRMS** (ESI): calcd for $C_{17}H_{24}NO_3$ $[M-CF_3CO_2H+H]^+$: 290.1751, found: 290.1752.

Compound **8m** (ethyl (*R*)-2-amino-3,3-dimethylbutanoate, trifluoroacetate salt)

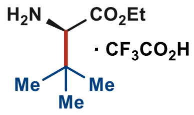


Synthesized according to the general procedure described in **Section 4.2** on 0.1 mmol scale from *N*-sulfinyl amide **5m**. The product is a colorless solid (27 mg, **99% yield**).

**$^{1}$H NMR** (500 MHz, MeOD) δ 5.87 (ABqq, *J* = 16.0, 7.2 Hz, 2H), 5.33 (s, 1H), 2.89 (t, *J* = 7.2 Hz, 3H), 2.67 (s, 9H).

**$^{13}$C NMR** (126 MHz, MeOD) δ 169.7, 63.4, 62.9, 34.3, 26.7, 14.4.

**$^{19}$F NMR** (377 MHz, MeOD) δ -77.04.

***$R_f$*** = 0.70 ($CH_2Cl_2$/MeOH 10:1, red color upon treatment with ninhydrin stain)

**$[\alpha]^{25}_{D}$** = −5.9 (*c* 4.0, MeOH)

**HRMS** (ESI): calcd for $C_8H_{18}NO_2$ $[M-CF_3CO_2H+H]^+$: 160.1332, found: 160.1331.

The spectroscopic data is in agreement with the literature.[1]

## 6. *NMR spectra*

$^1$**H NMR** (500 MHz, MeOD) of compound **SI-1**

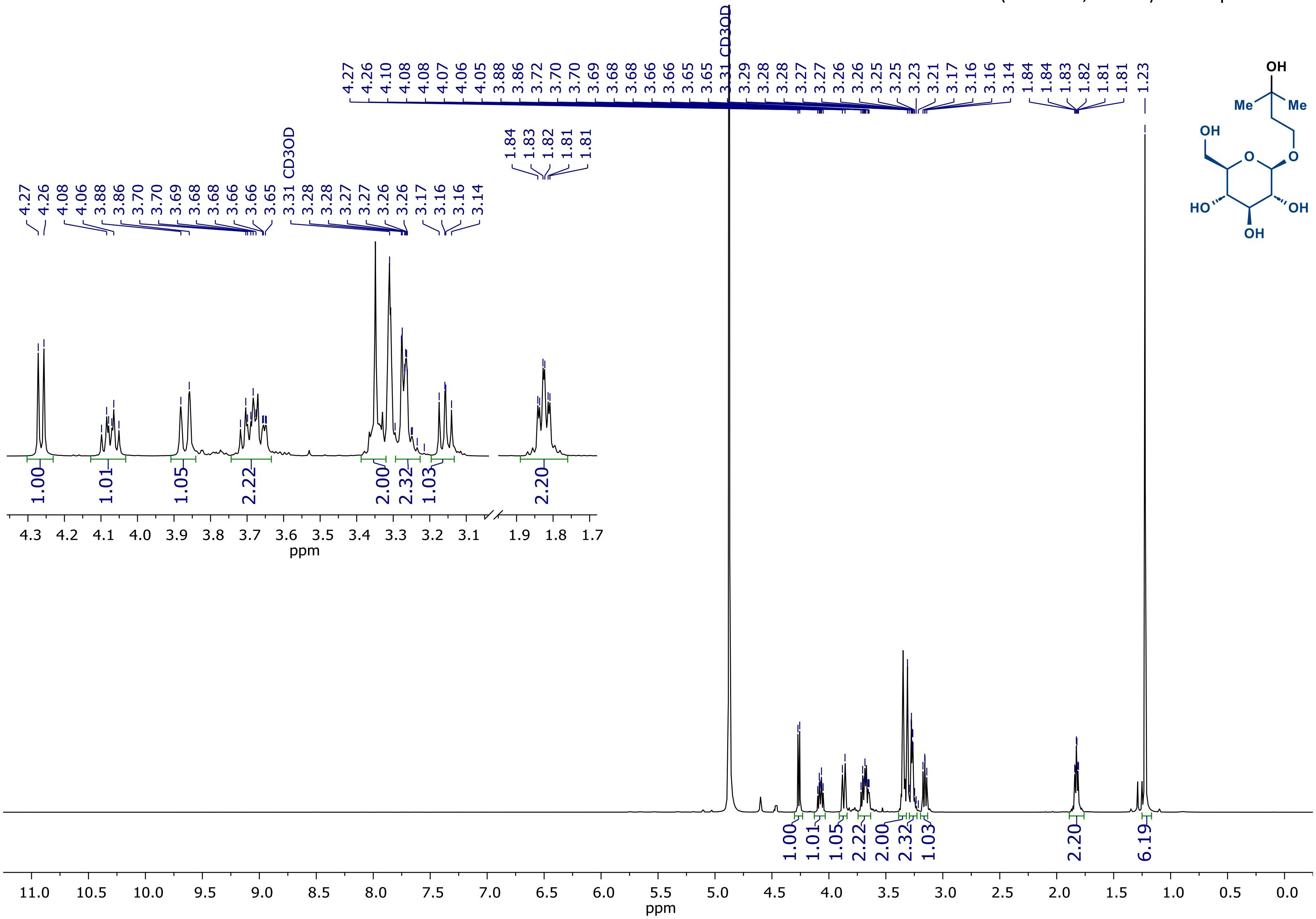

**$^{13}$C NMR** (126 MHz, MeOD) of compound **SI-1**

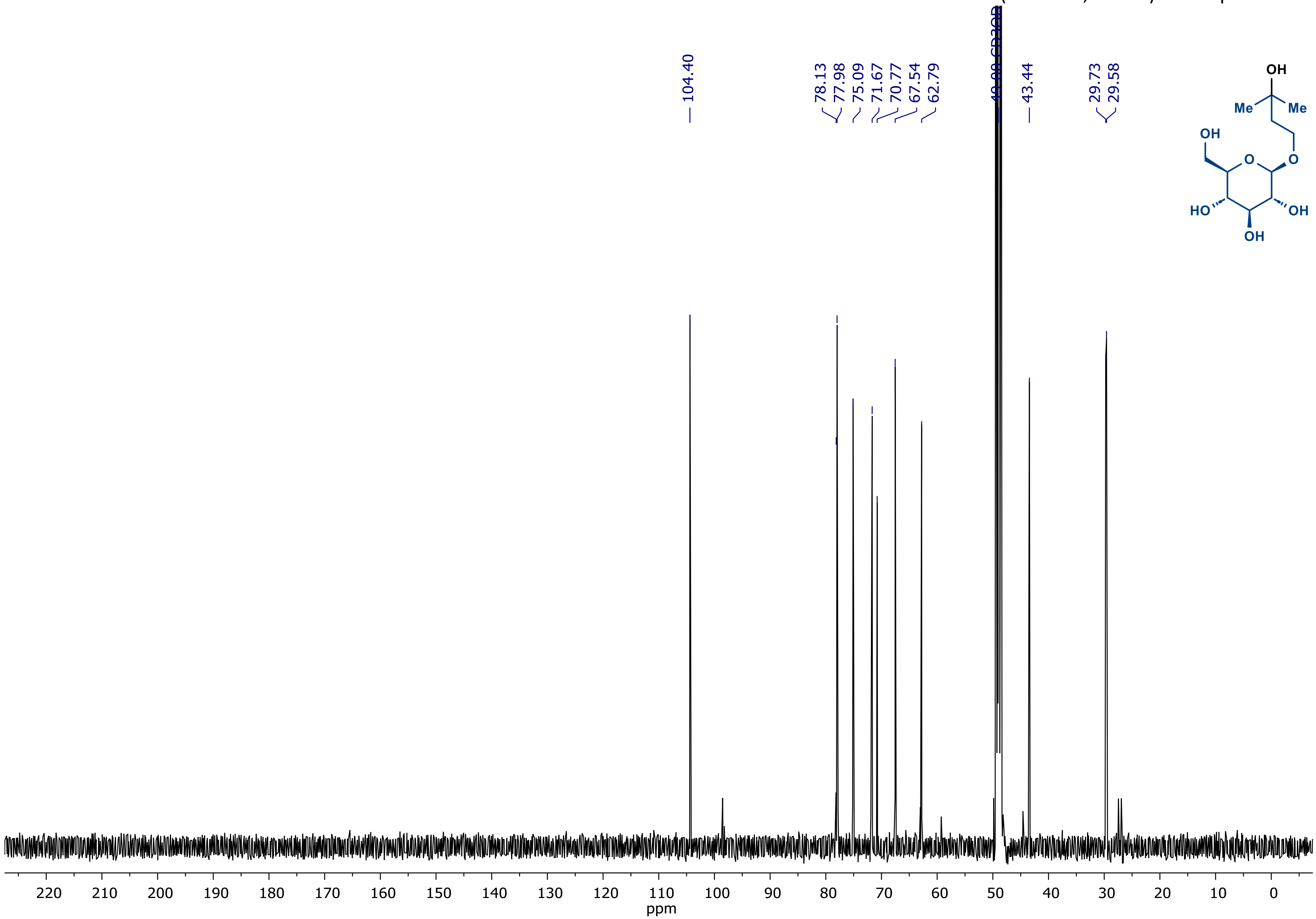

**COSY** of compound **SI-1**

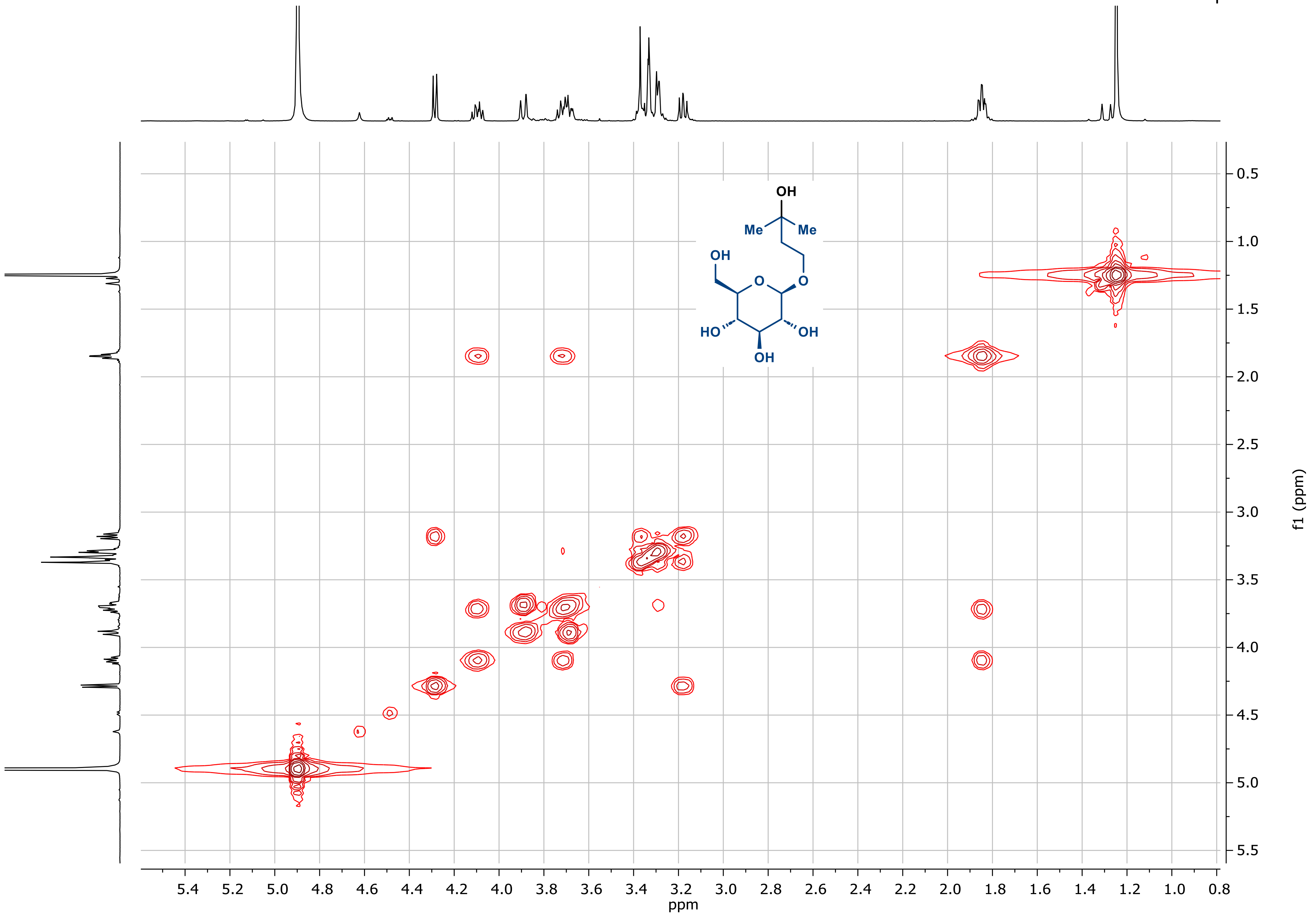

**$^1$H NMR** (500 MHz, $CDCl_3$) of compound **SI-2**

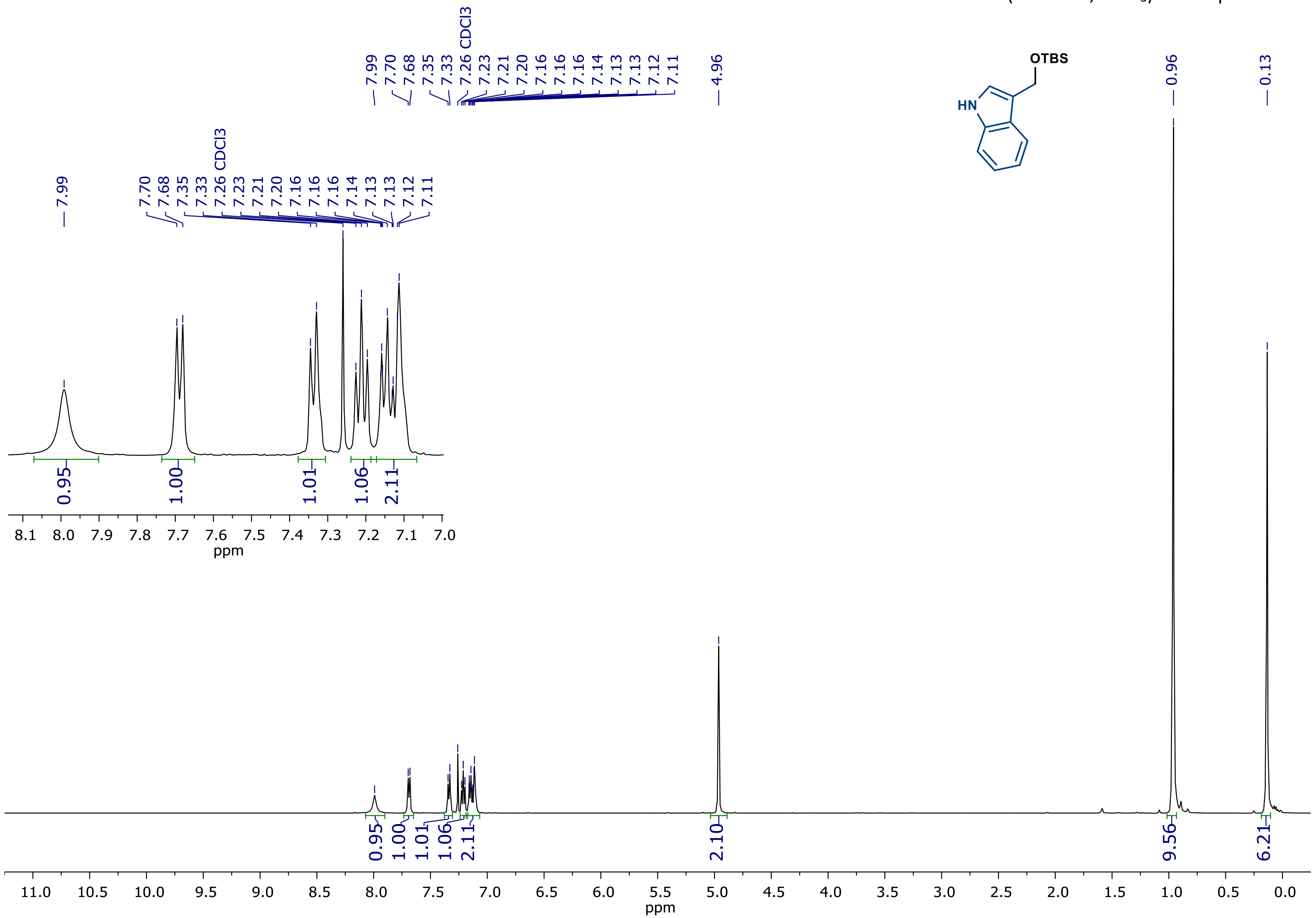

**$^{13}$C NMR** (126 MHz, $CDCl_3$) of compound **SI-2**

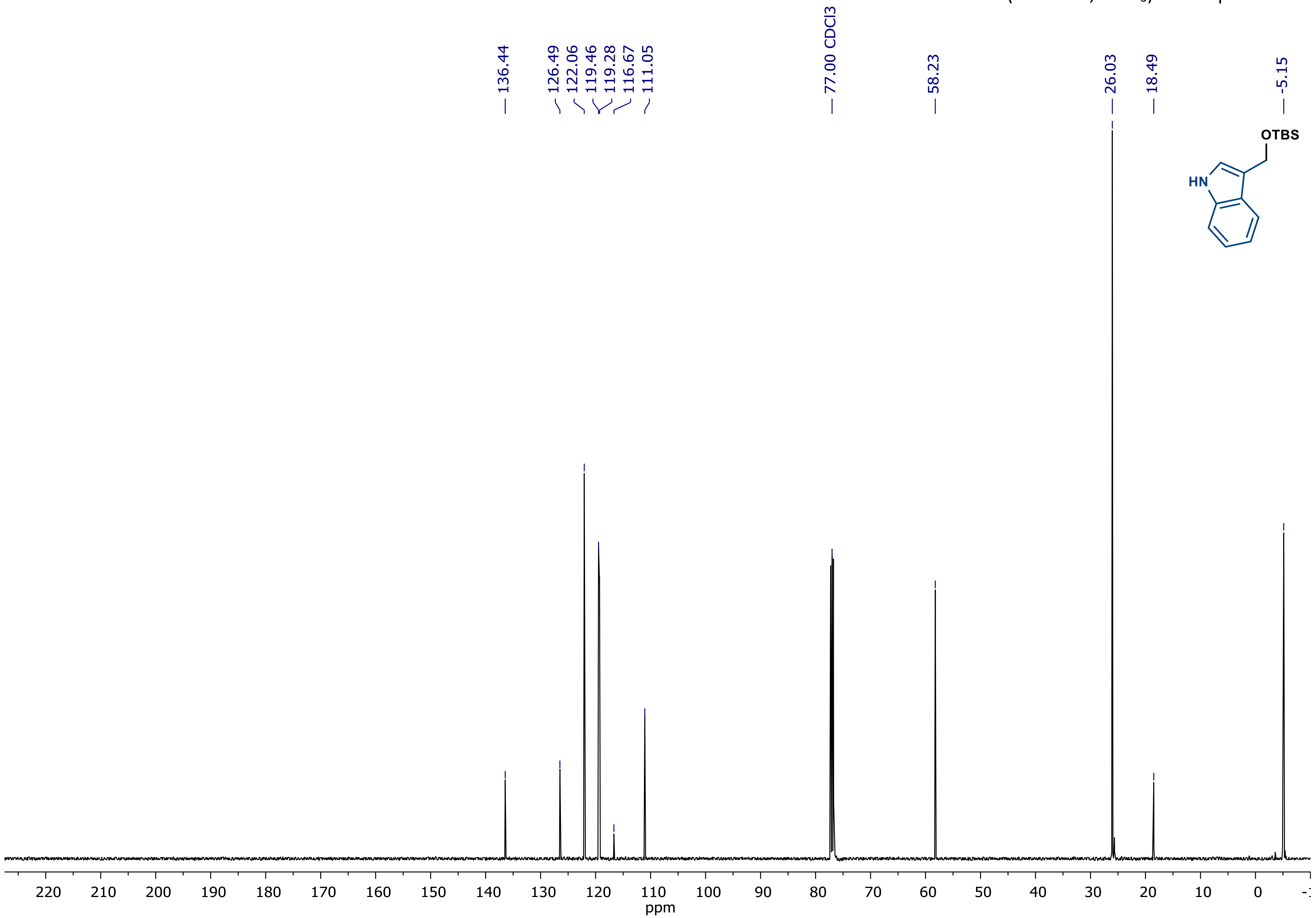

**$^1$H NMR** (500 MHz, $CDCl_3$) of compound **SI-3**

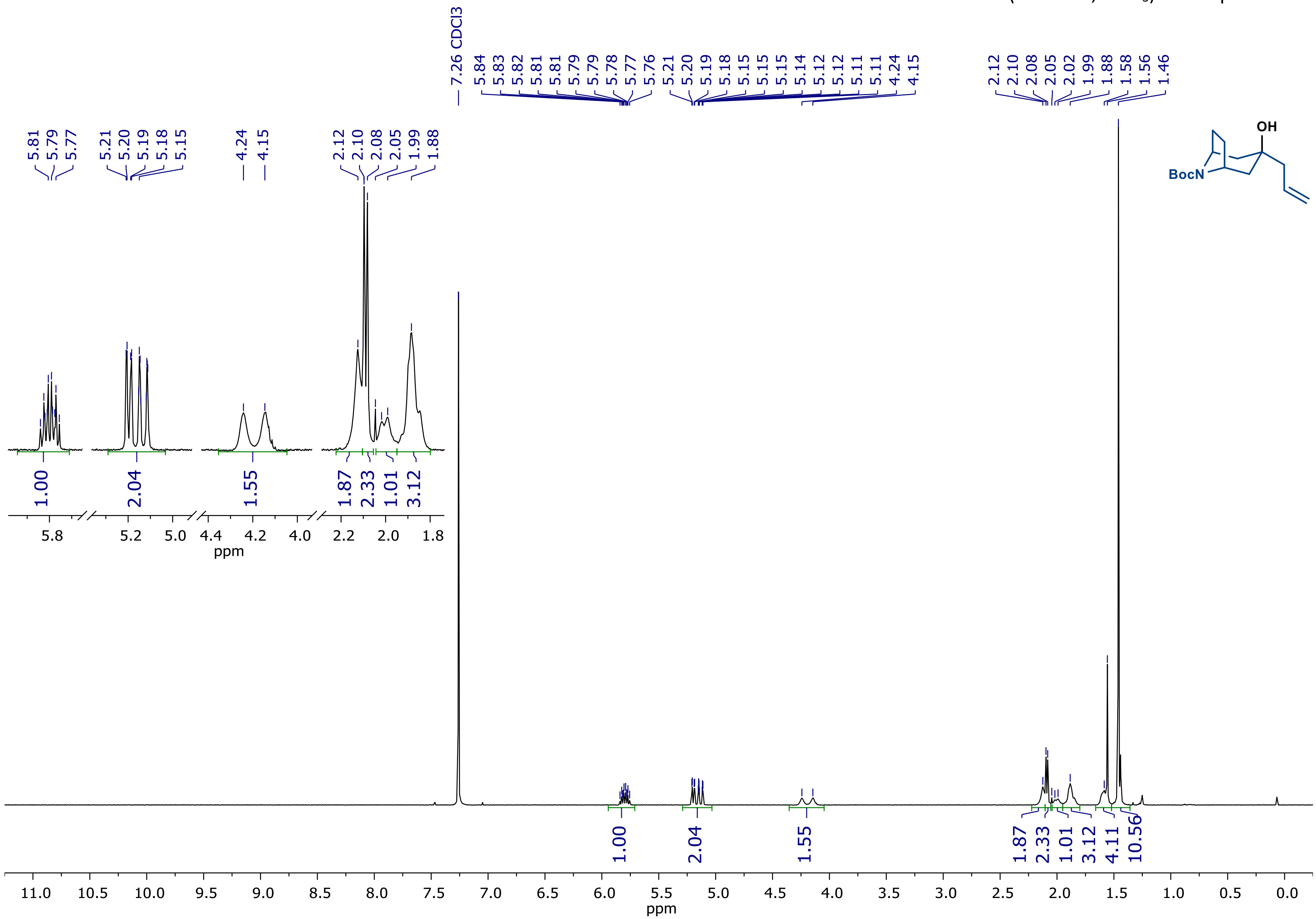

**$^{13}$C NMR** (126 MHz, $CDCl_3$) of compound **SI-3**

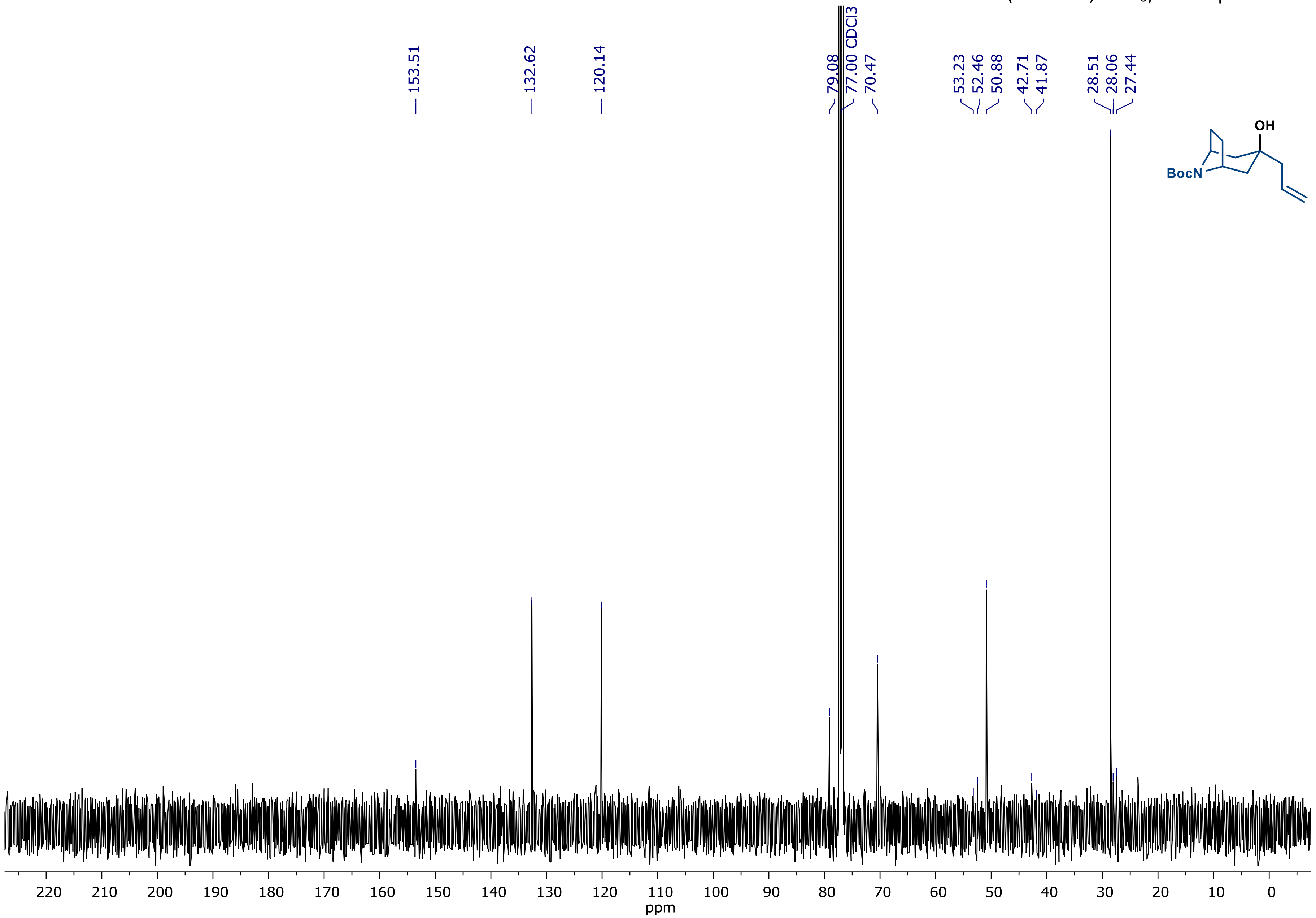

**$^1$H NMR** (500 MHz, $CDCl_3$) of compound **SI-4**

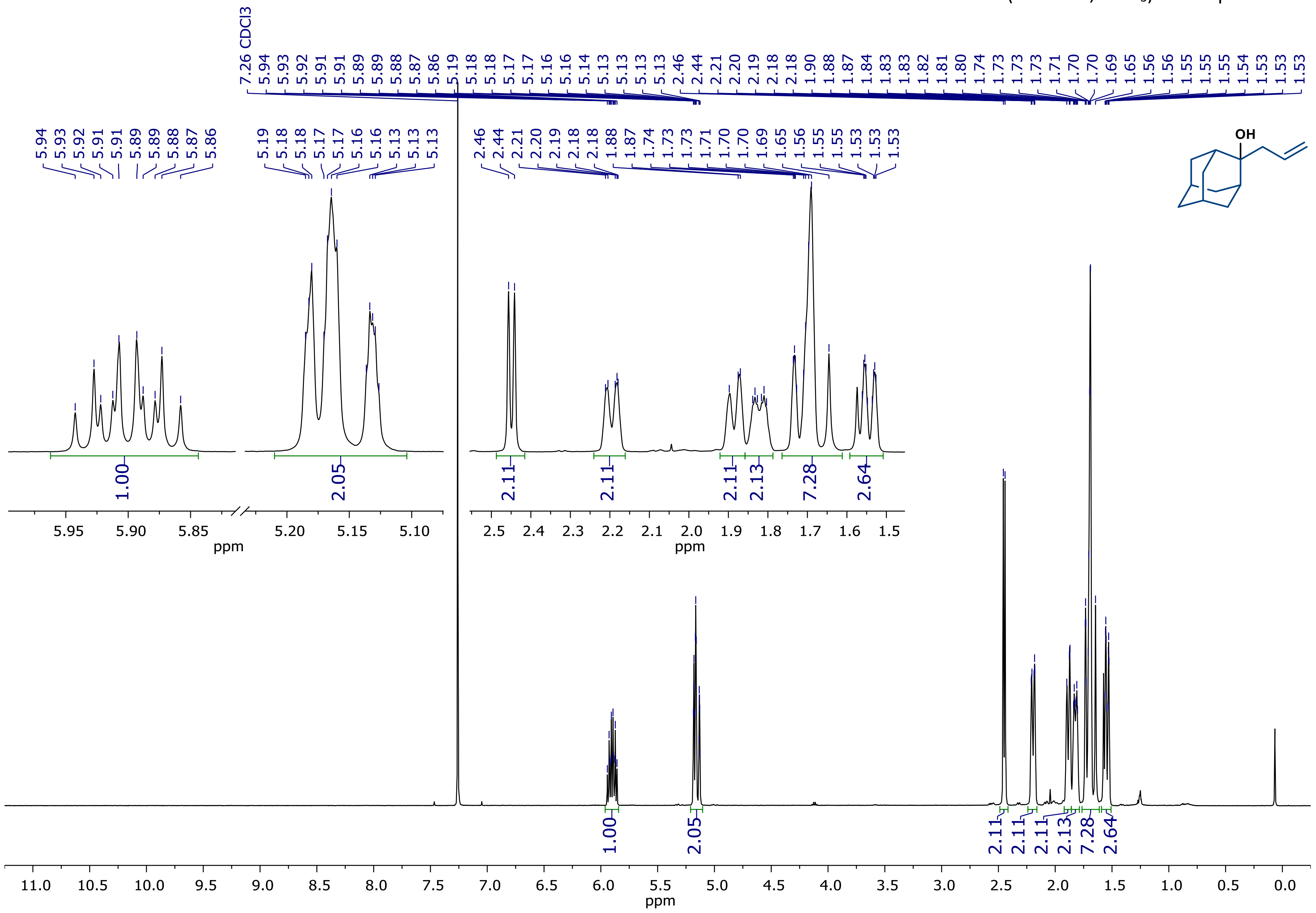

$^{13}$**C NMR** (126 MHz, $CDCl_3$) of compound **SI-4**

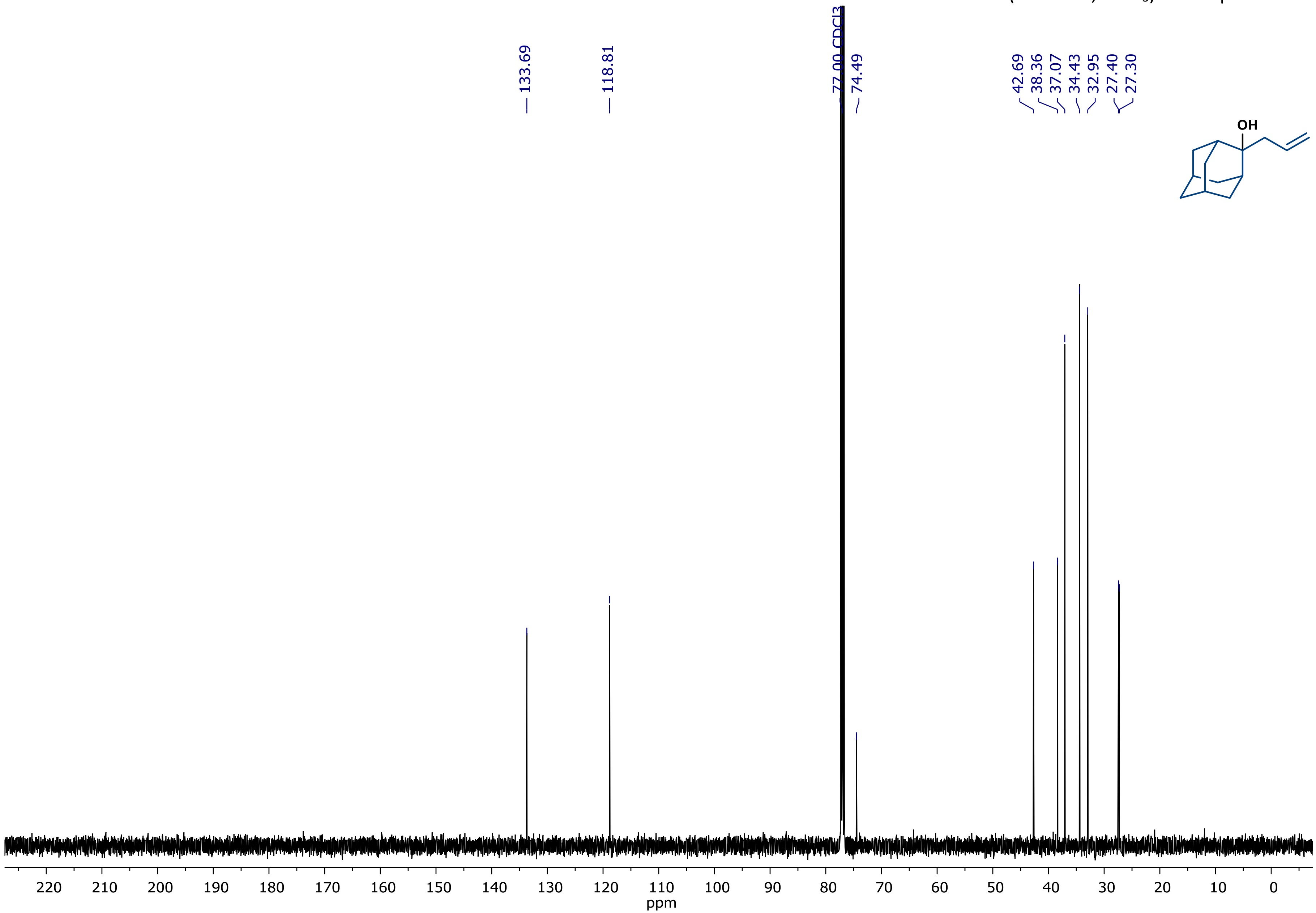

$^{1}$H NMR (500 MHz, $CDCl_3$) of compound **SI-5**

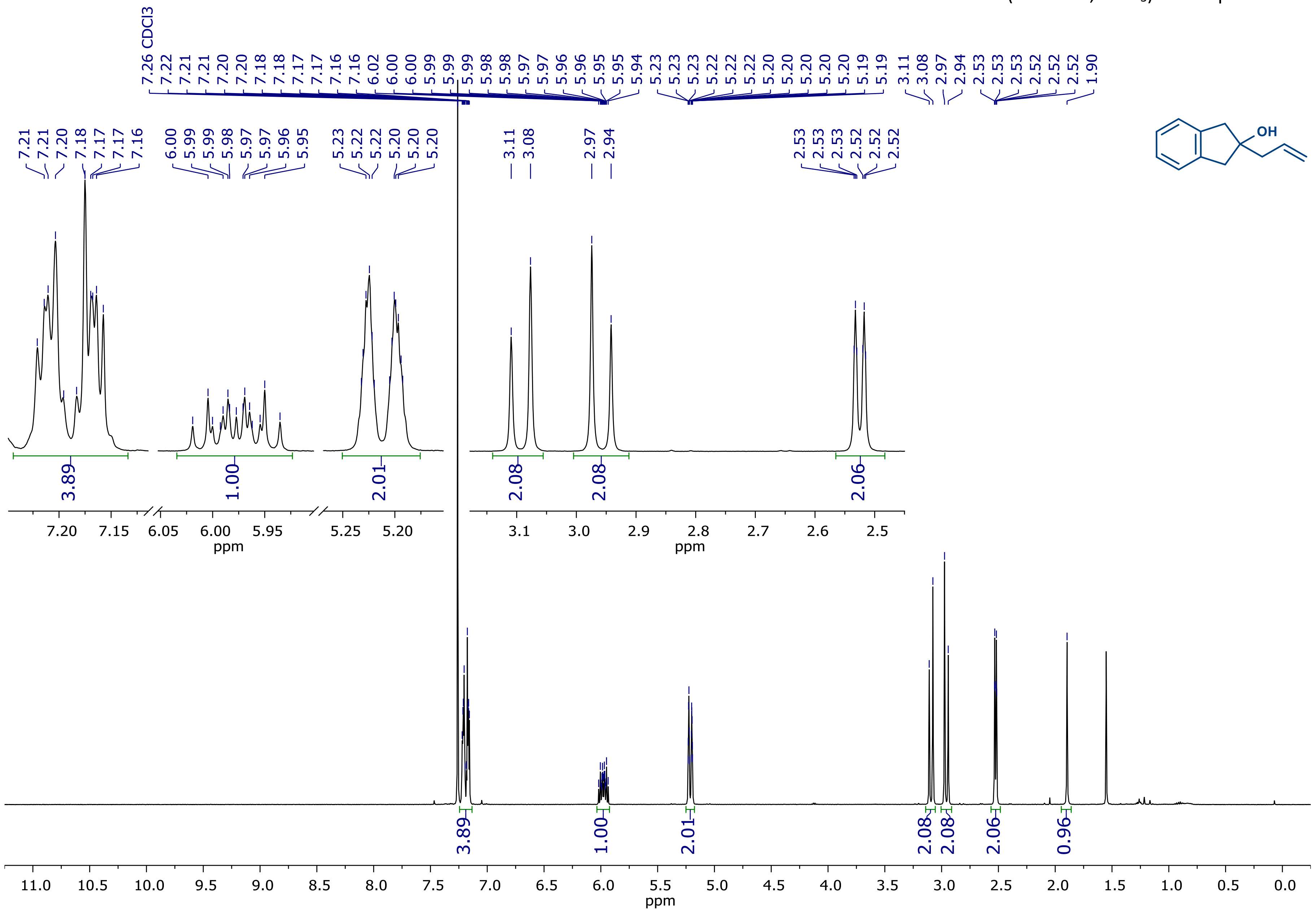

$^{13}$**C NMR** (126 MHz, $CDCl_3$) of compound **SI-5**

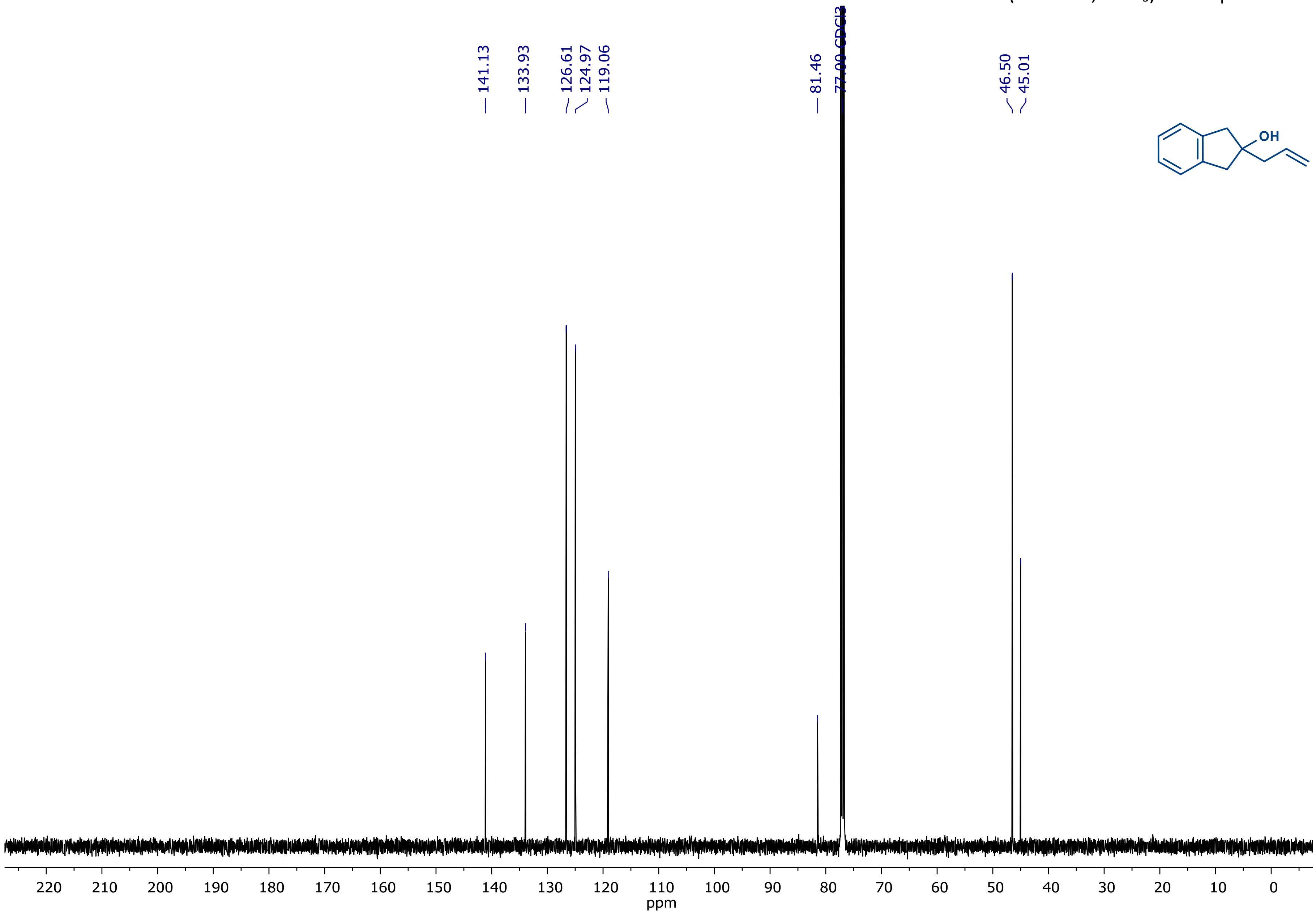

**$^1$H NMR** (500 MHz, $CDCl_3$) of compound **1d**

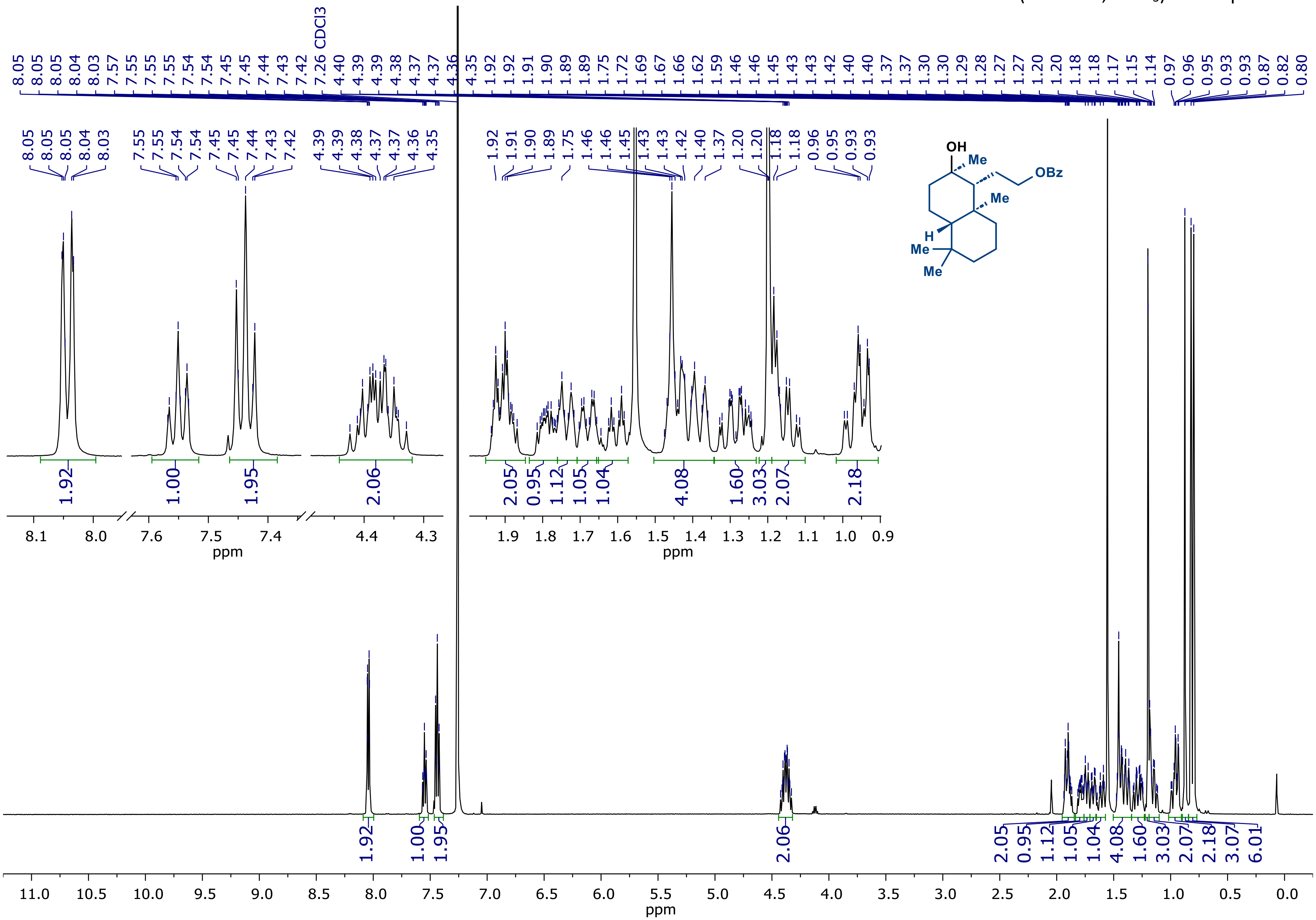

**$^{13}$C NMR** (126 MHz, $CDCl_3$) of compound **1d**

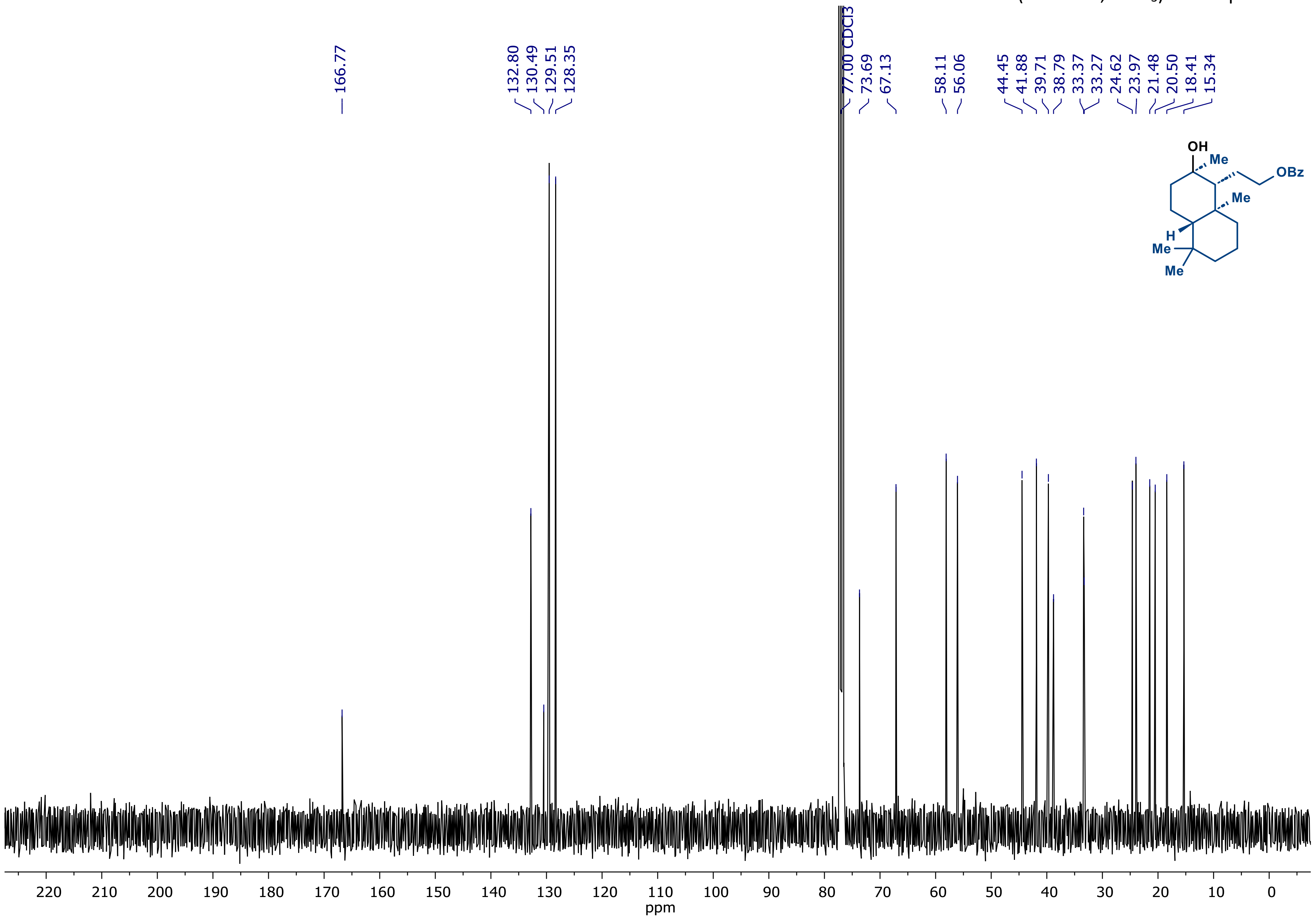

$^{1}$H NMR (500 MHz, $CDCl_3$) of compound **1i**

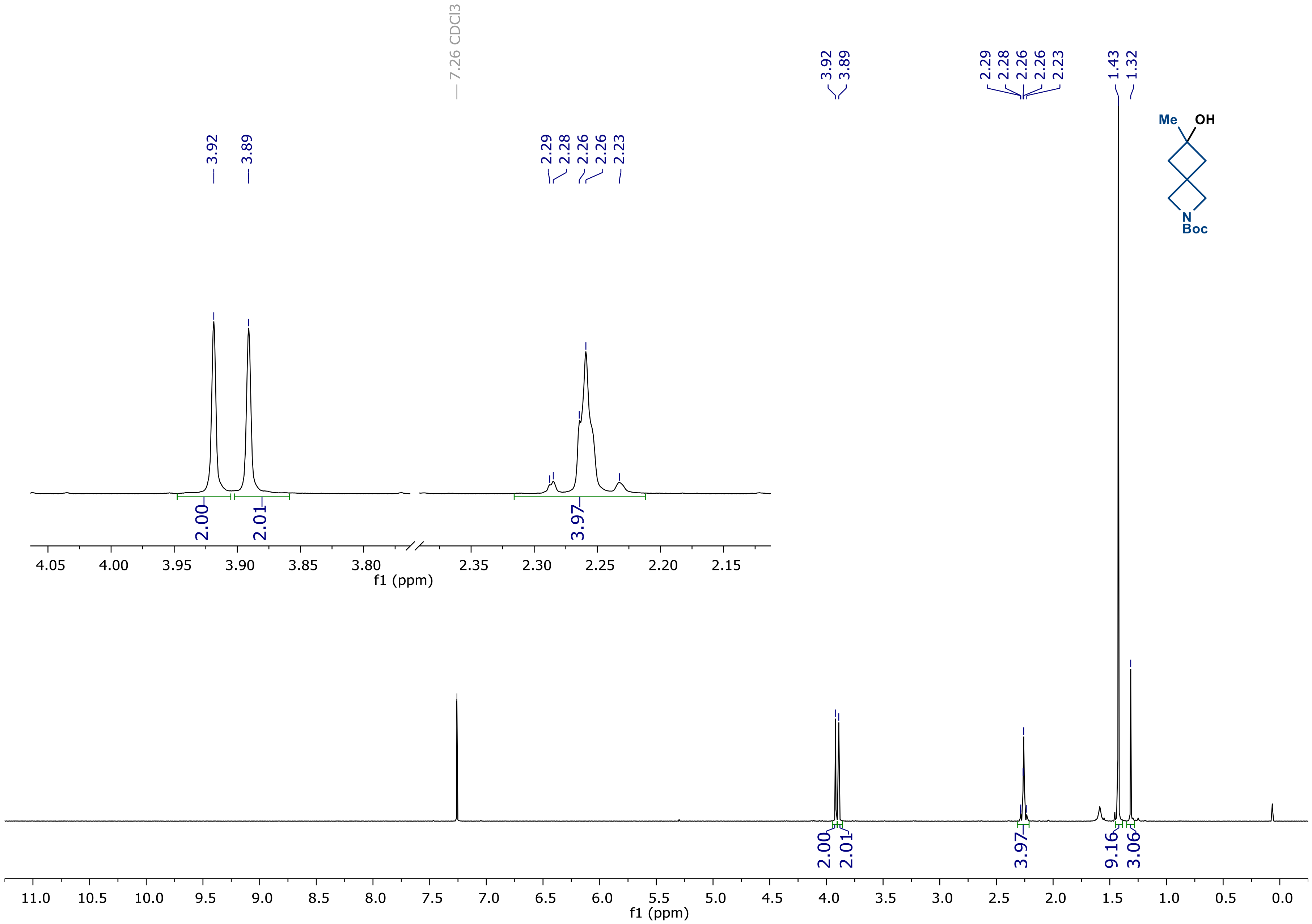

$^{13}$C NMR (126 MHz, $CDCl_3$) of compound **1i**

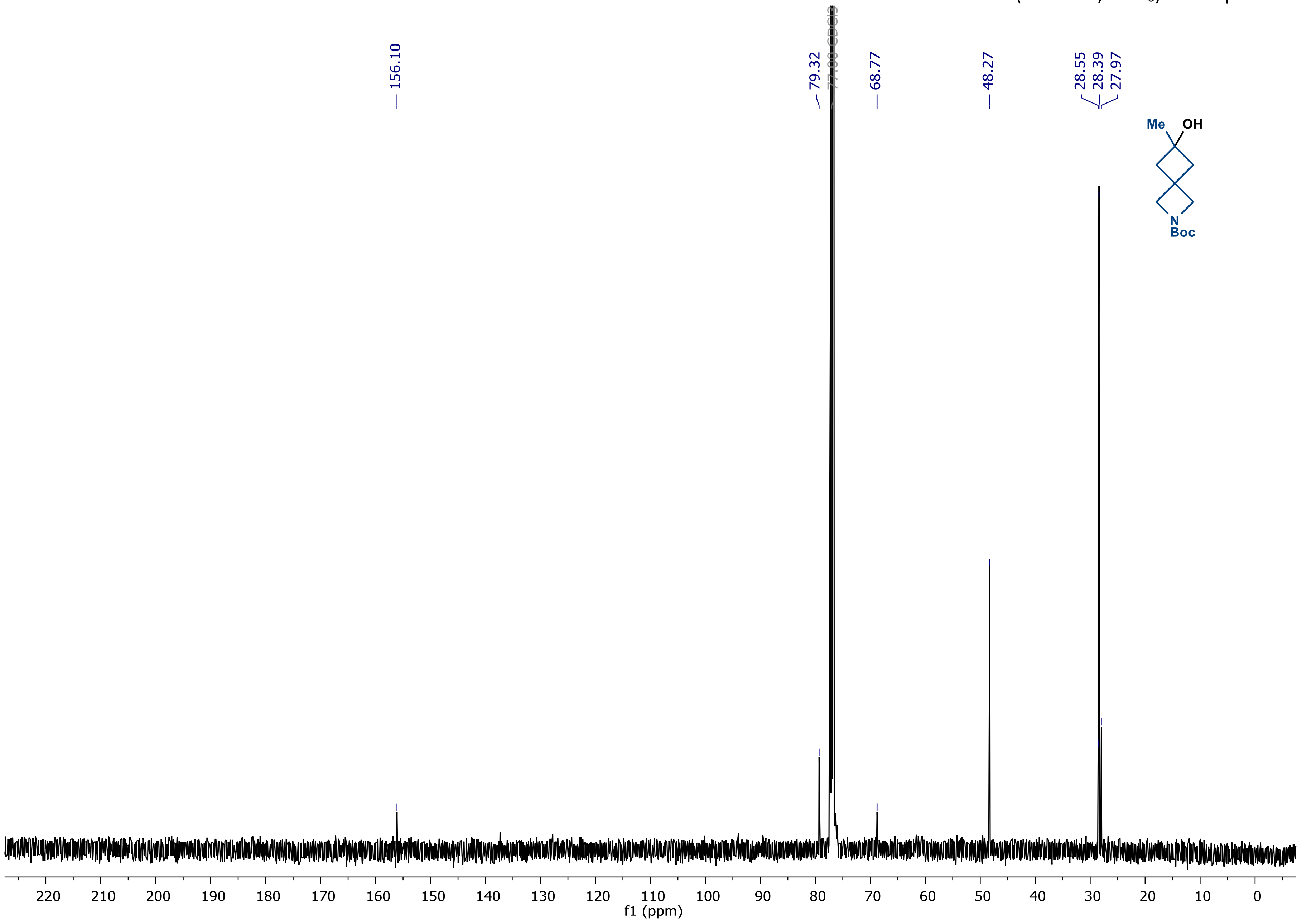

$^1$**H NMR** (500 MHz, $CDCl_3$) of compound **1j**

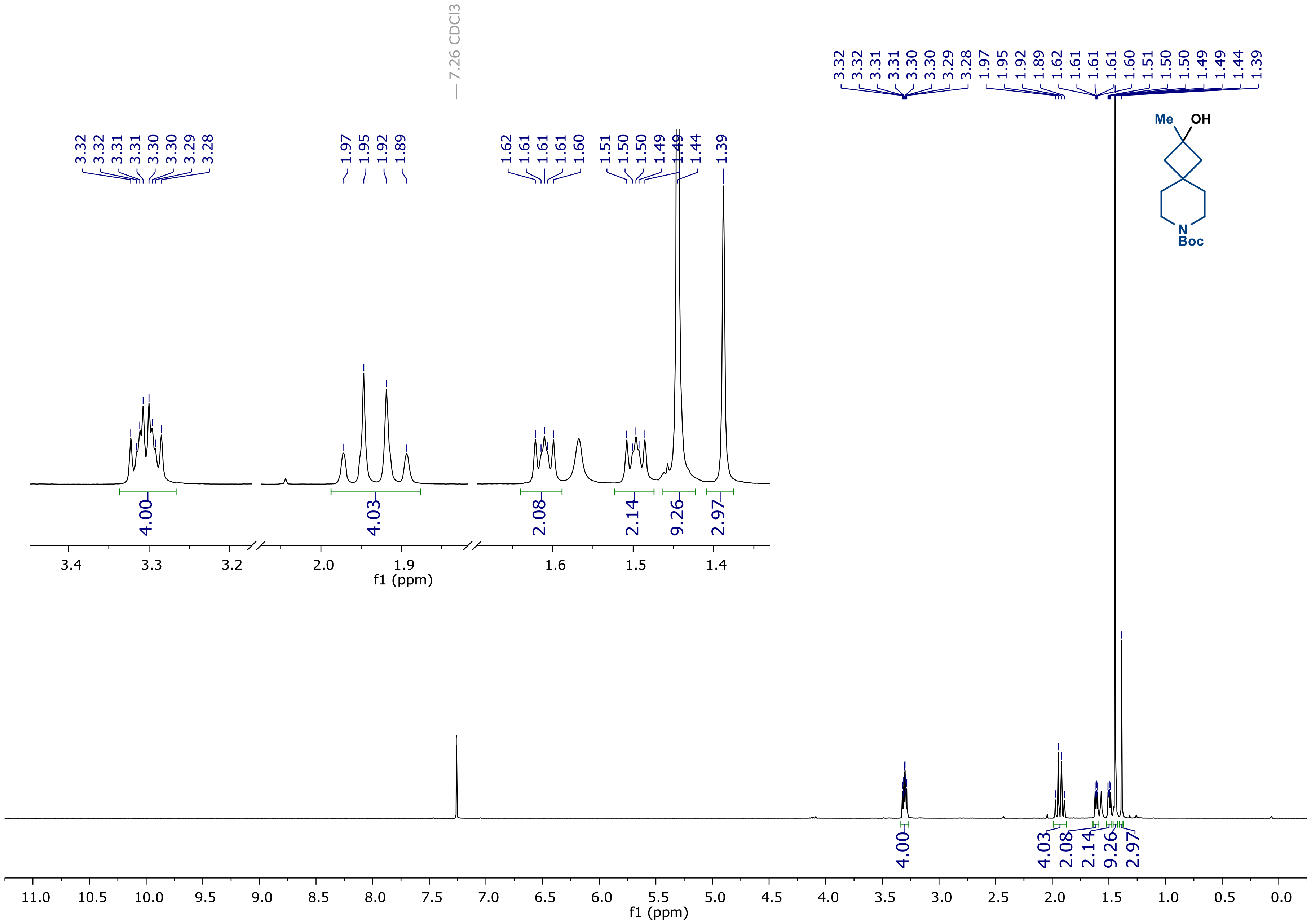

$^{13}$C NMR (126 MHz, $CDCl_3$) of compound **1j**

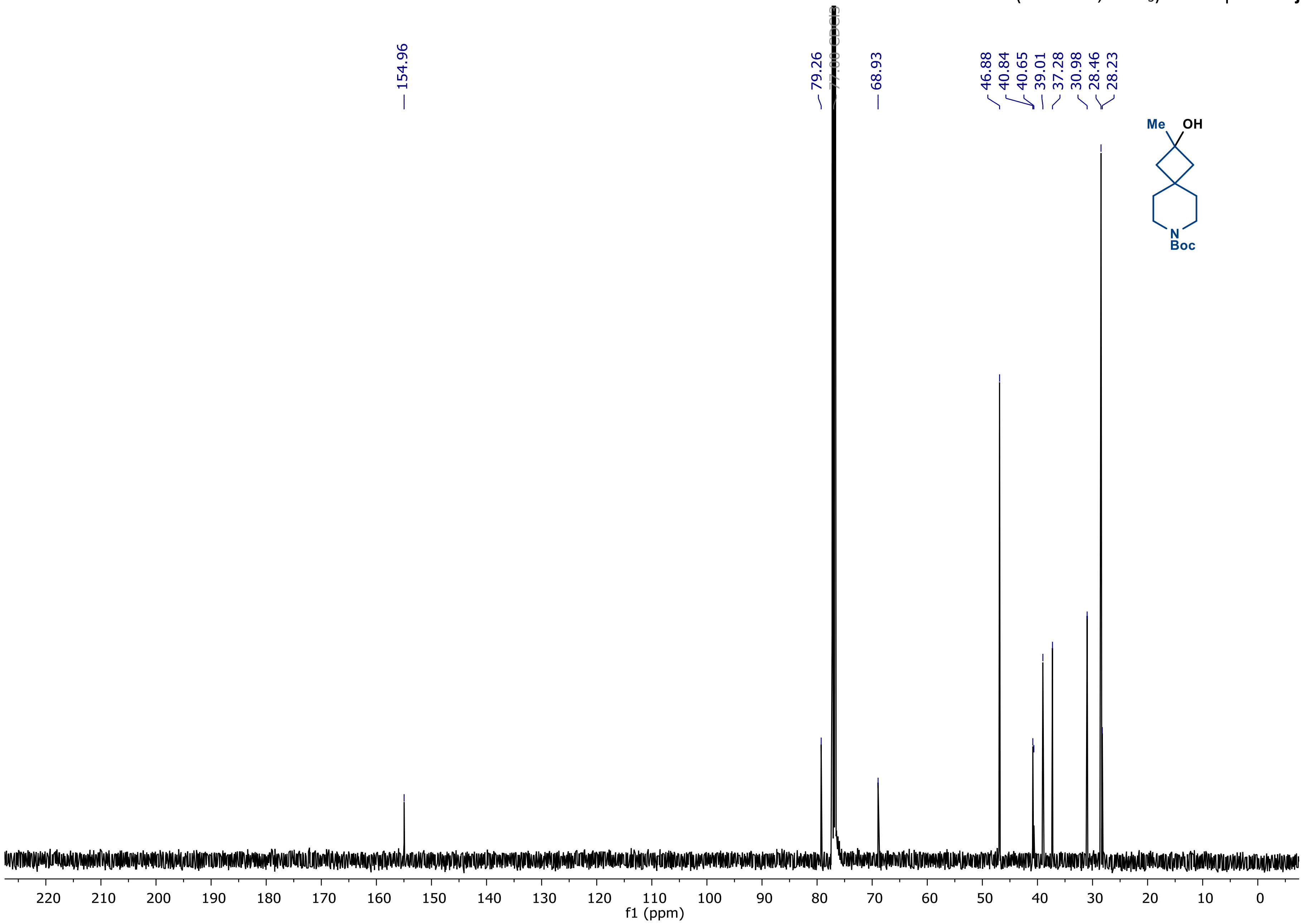

**$^1$H NMR** (500 MHz, $CDCl_3$) of compound **1l**

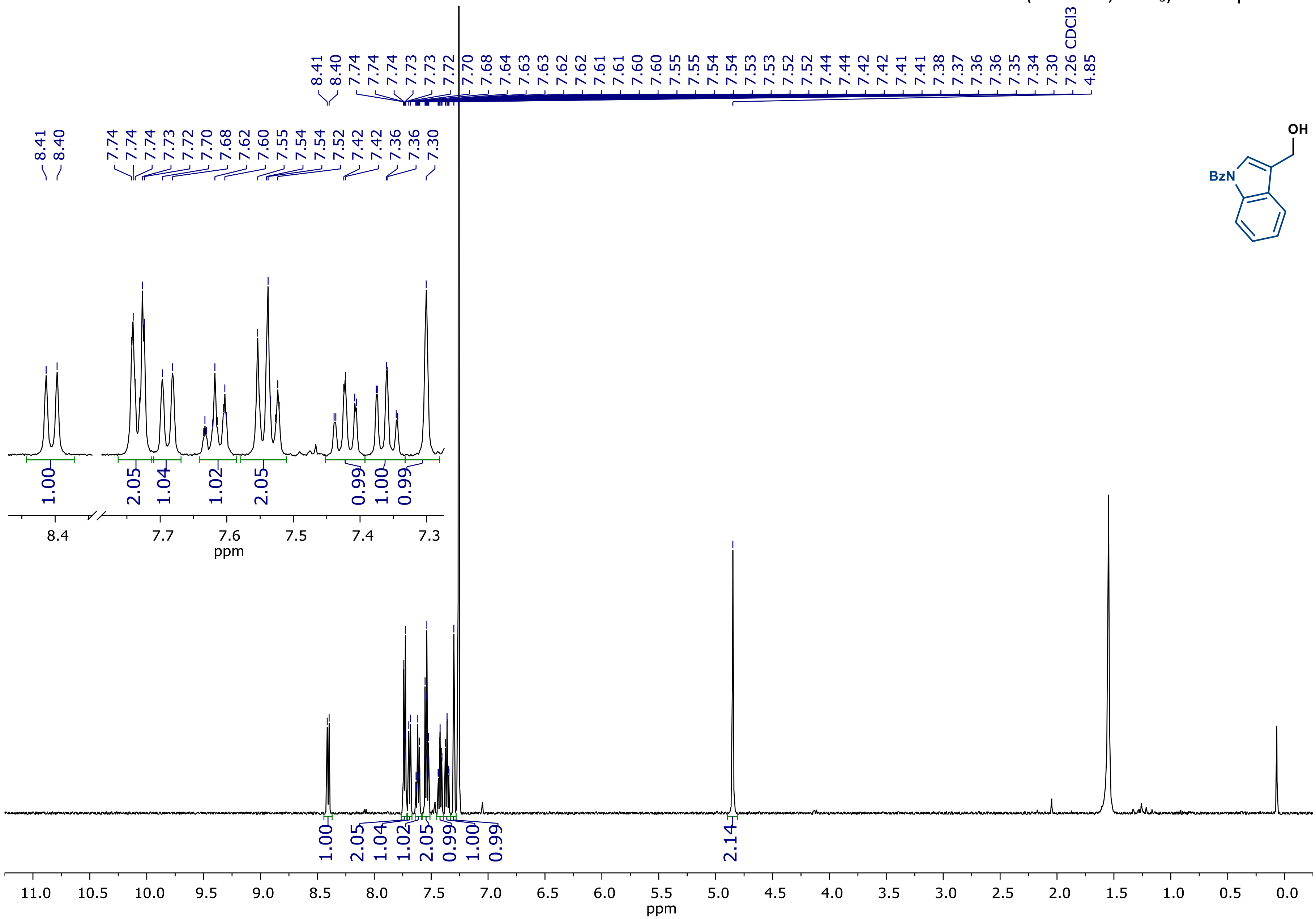

$^{13}$C NMR (126 MHz, $CDCl_3$) of compound **1l**

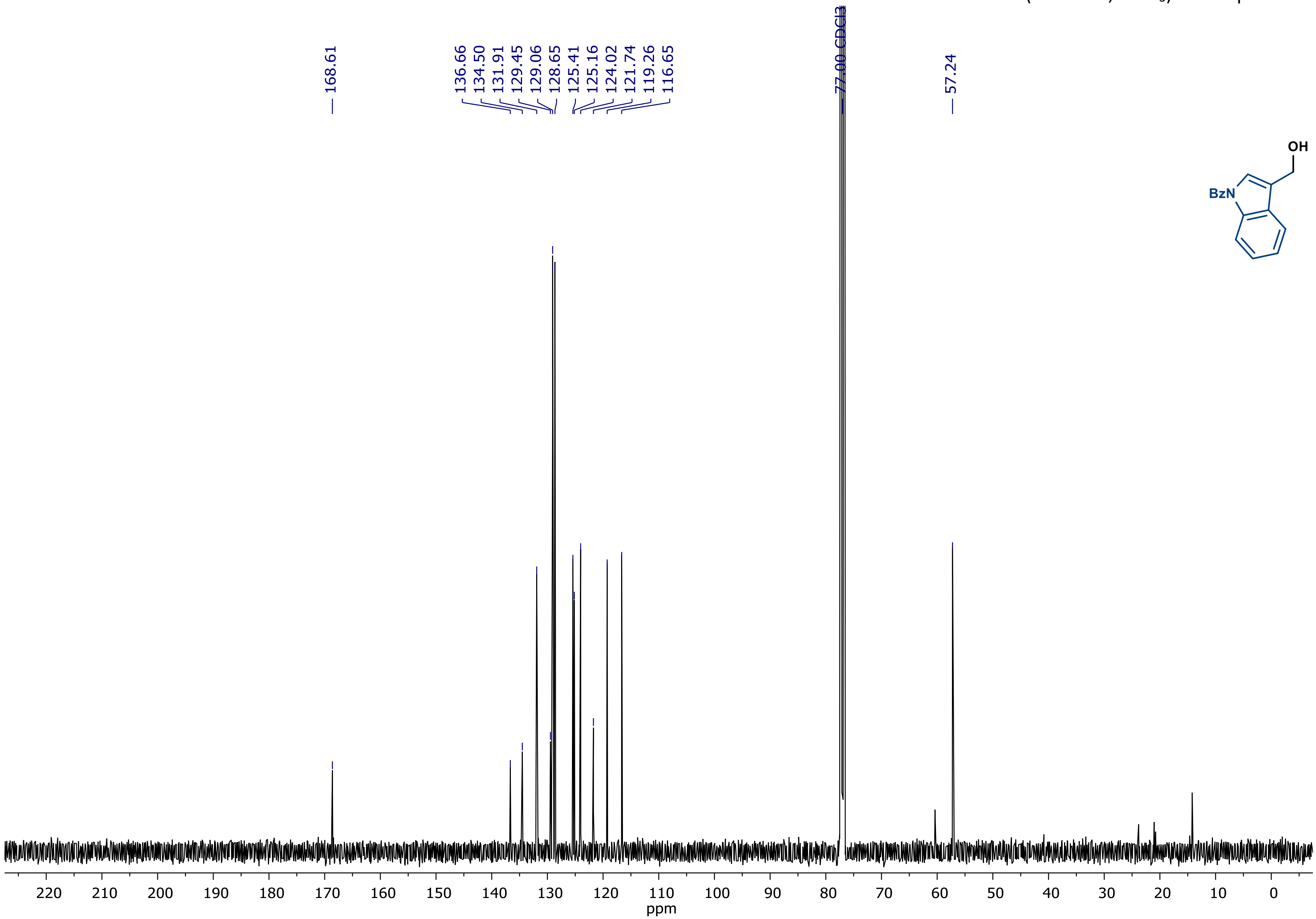

$^{1}$H NMR (500 MHz, $CDCl_3$) of compound **1p**

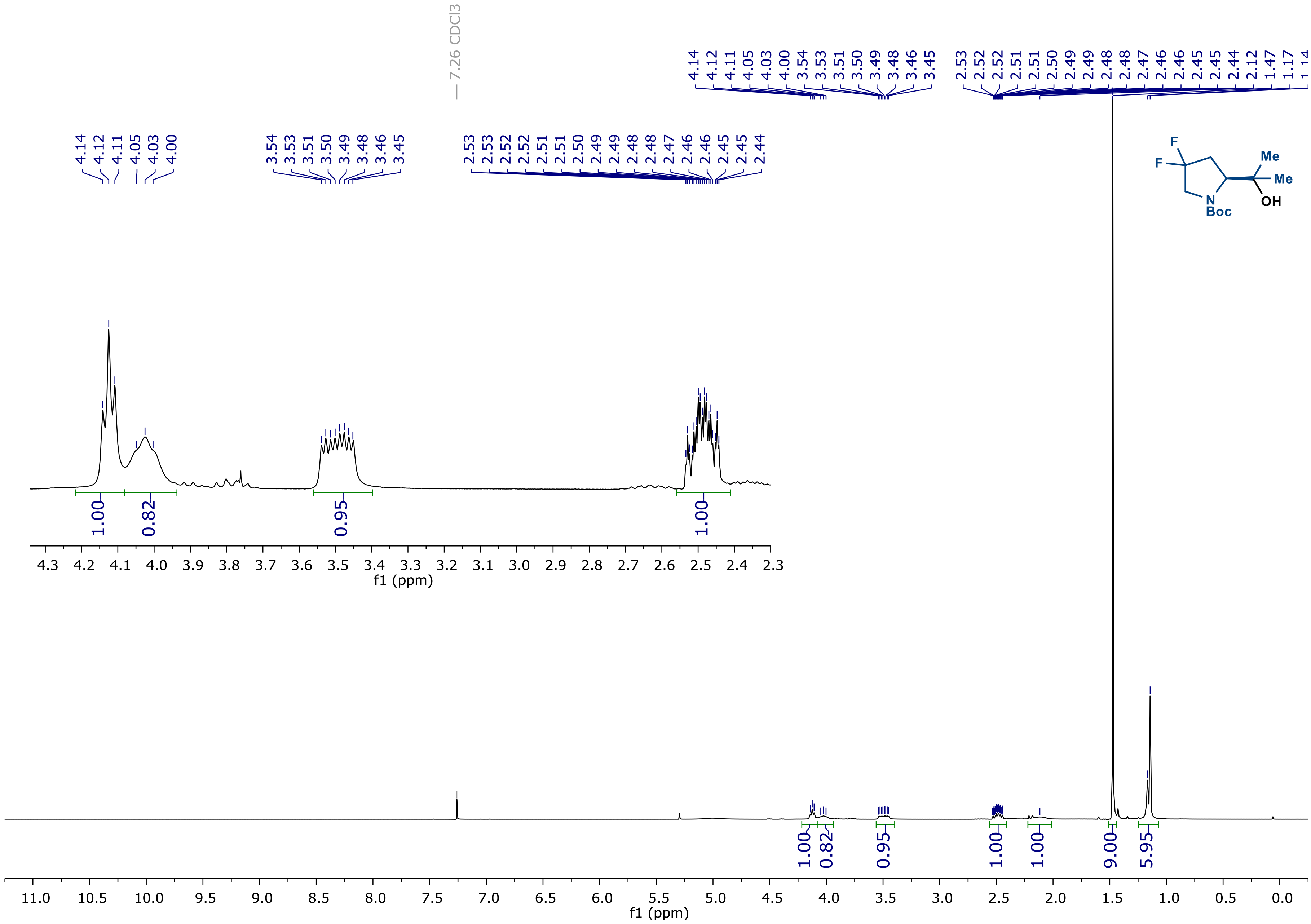

**$^{13}$C NMR** (126 MHz, $CDCl_3$) of compound **1p**

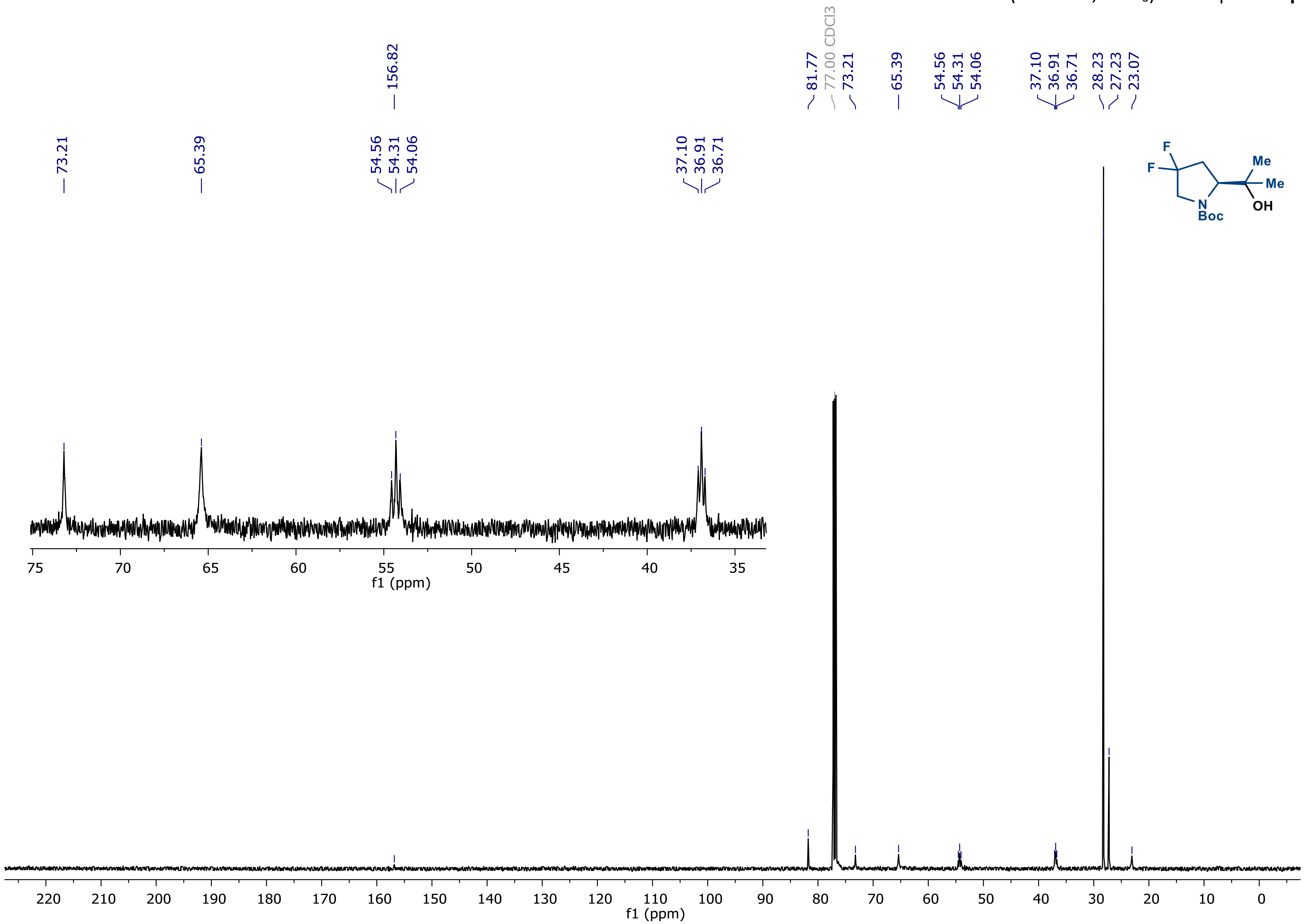

$^1$**H NMR** (500 MHz, $CDCl_3$) of compound **1r**

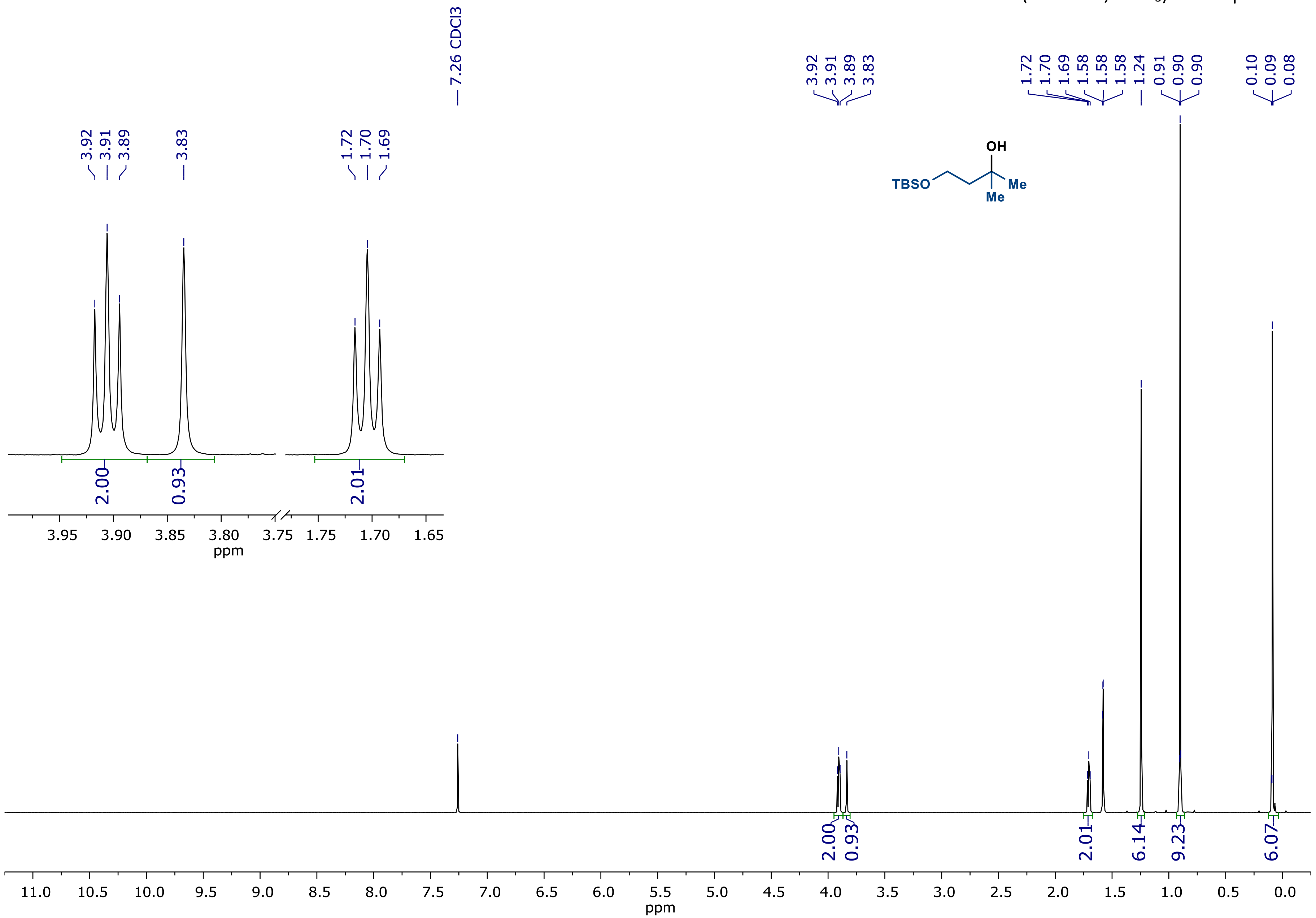

$^{13}$**C NMR** (126 MHz, $CDCl_3$) of compound **1r**

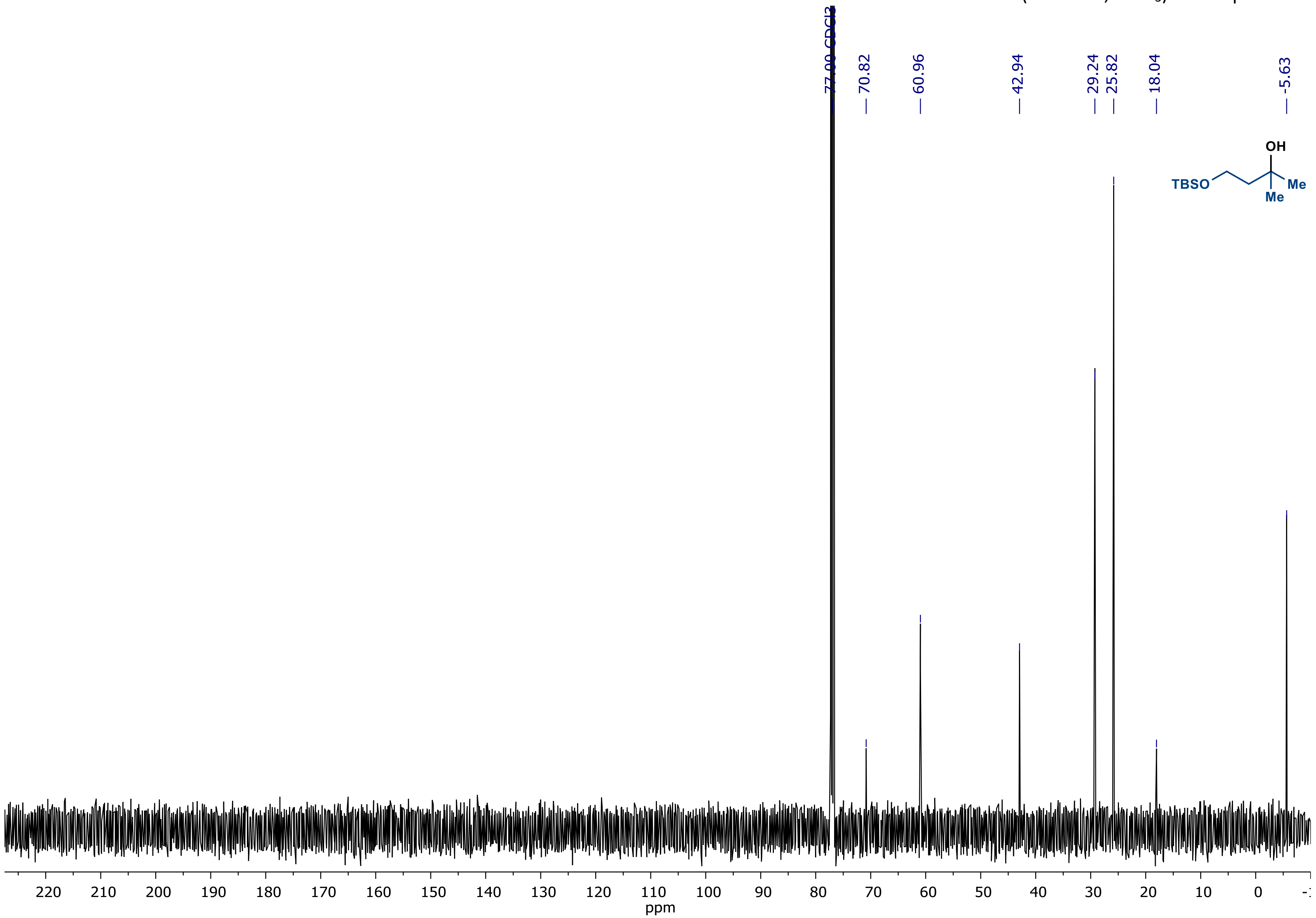

**$^1$H NMR** (500 MHz, $CDCl_3$) of compound **1s**

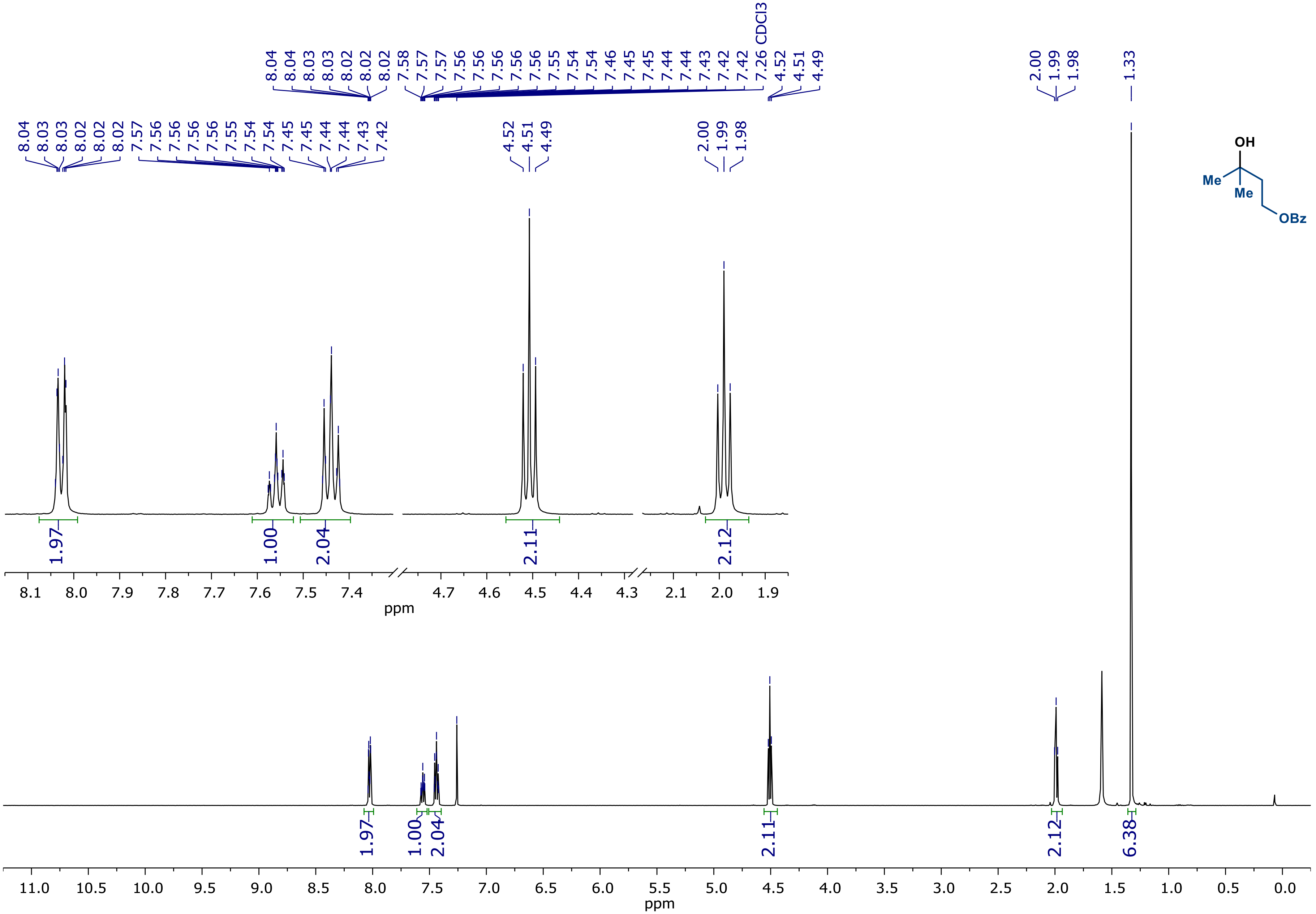

$^{13}$C NMR (126 MHz, $CDCl_3$) of compound **1s**

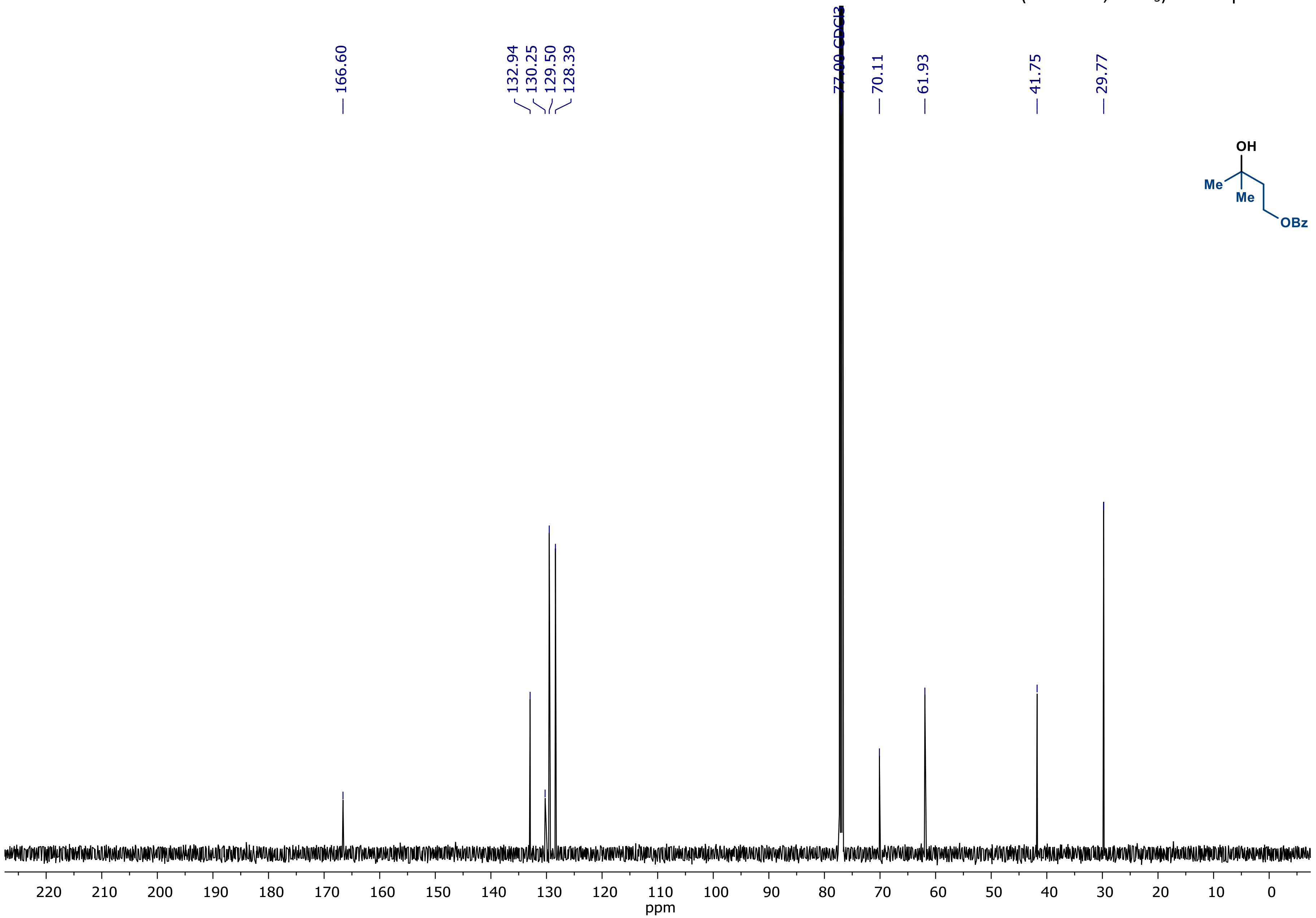

**¹H NMR** (500 MHz, $CDCl_3$) of compound **1t**

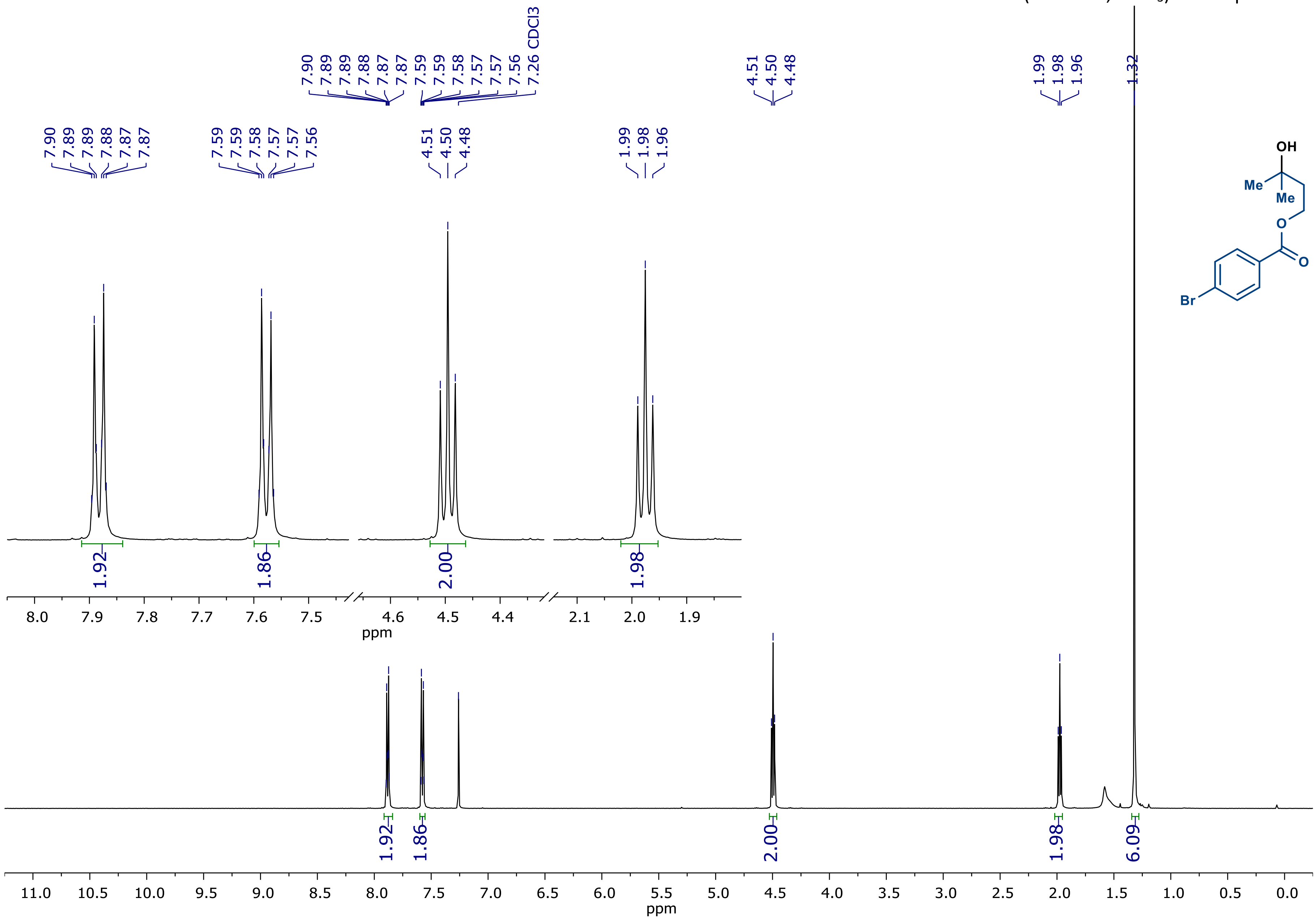

**$^{13}$C NMR** (126 MHz, $CDCl_3$) of compound **1t**

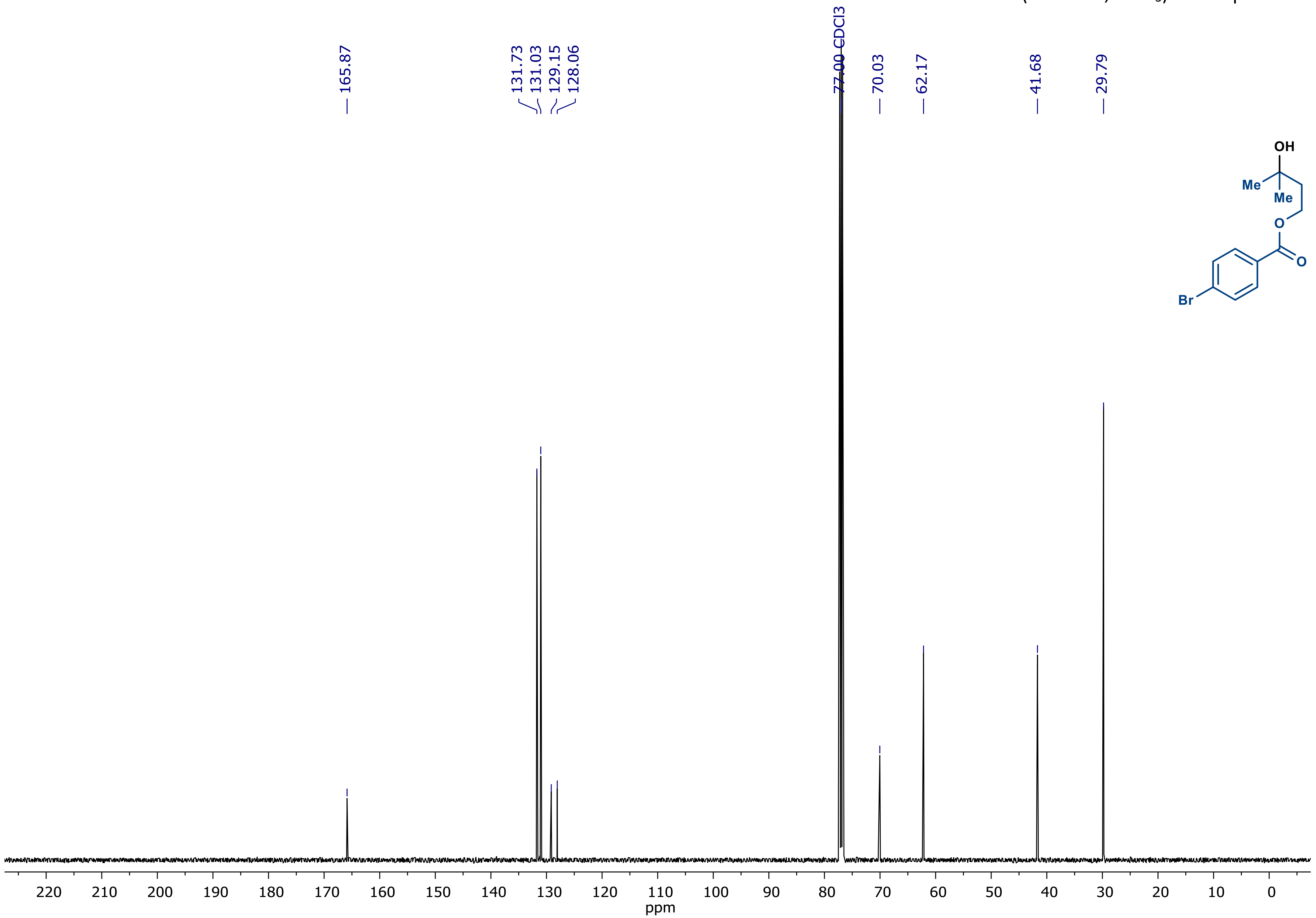

$^{1}$**H NMR** (500 MHz, $CDCl_3$) of compound **1u**

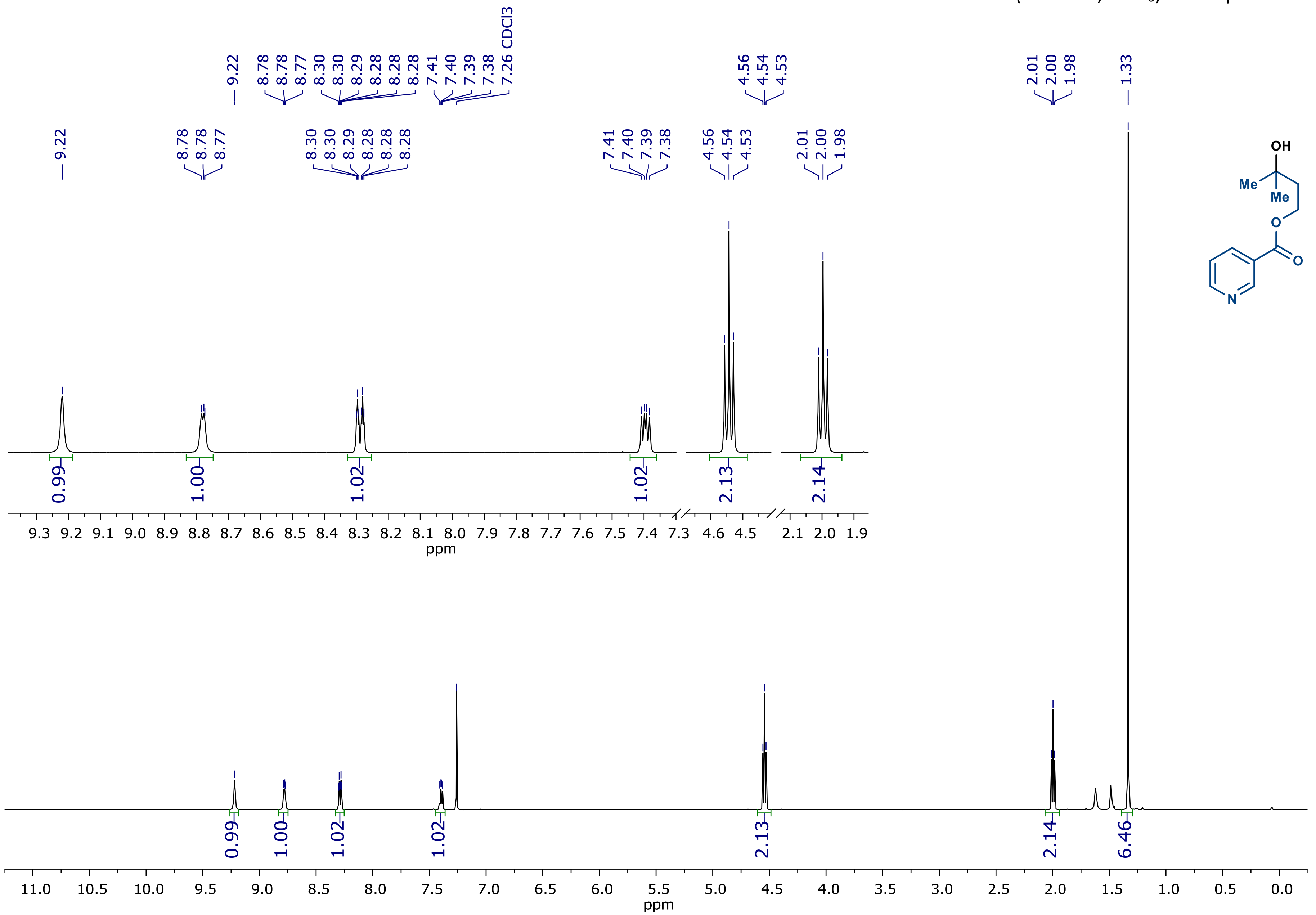

$^{13}$C NMR (126 MHz, $CDCl_3$) of compound **1u**

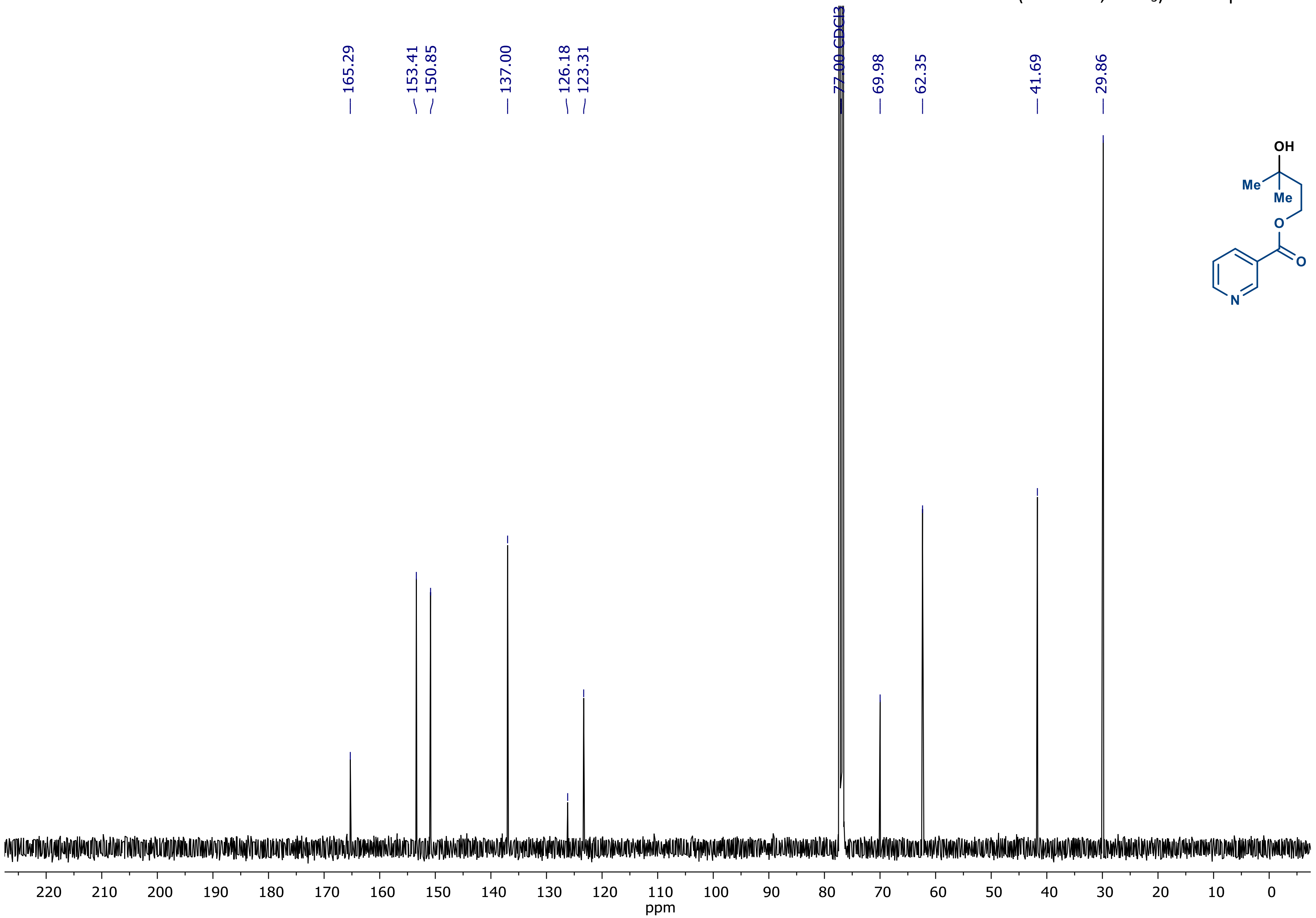

**¹H NMR** (500 MHz, $CDCl_3$) of compound **1v**

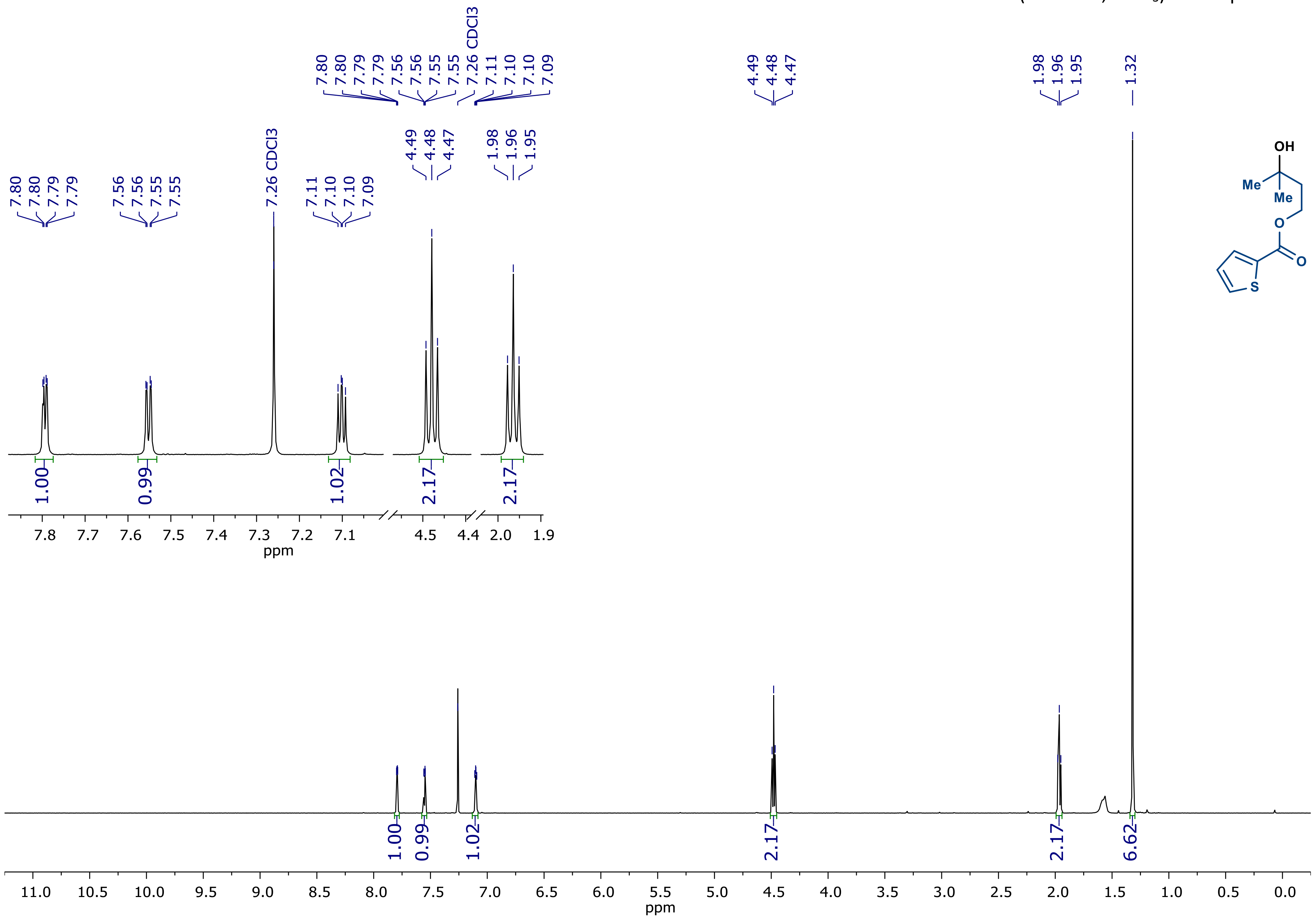

$^{13}$**C NMR** (126 MHz, $CDCl_3$) of compound **1v**

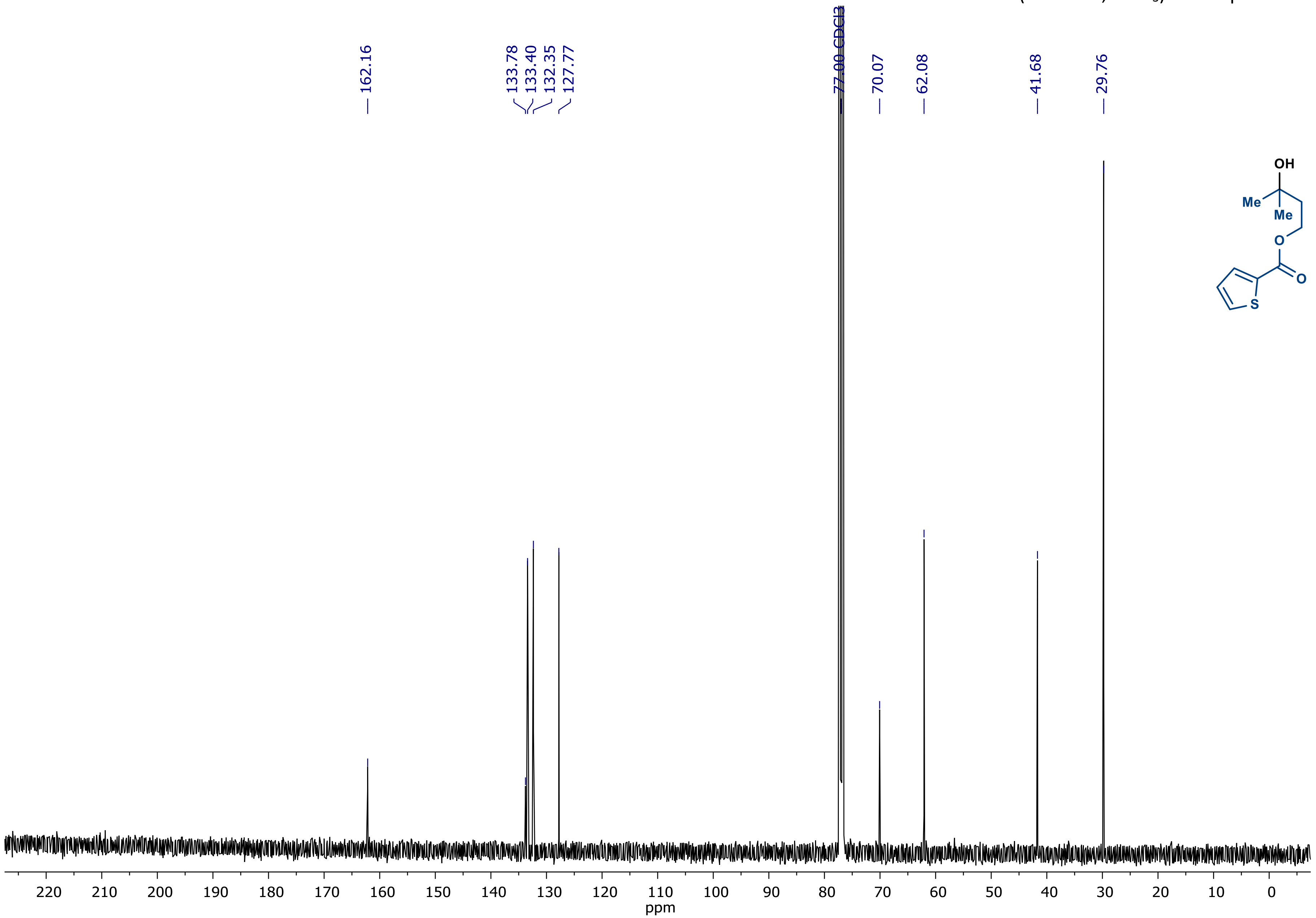

$^{1}$**H NMR** (500 MHz, $CDCl_3$) of compound **1w**

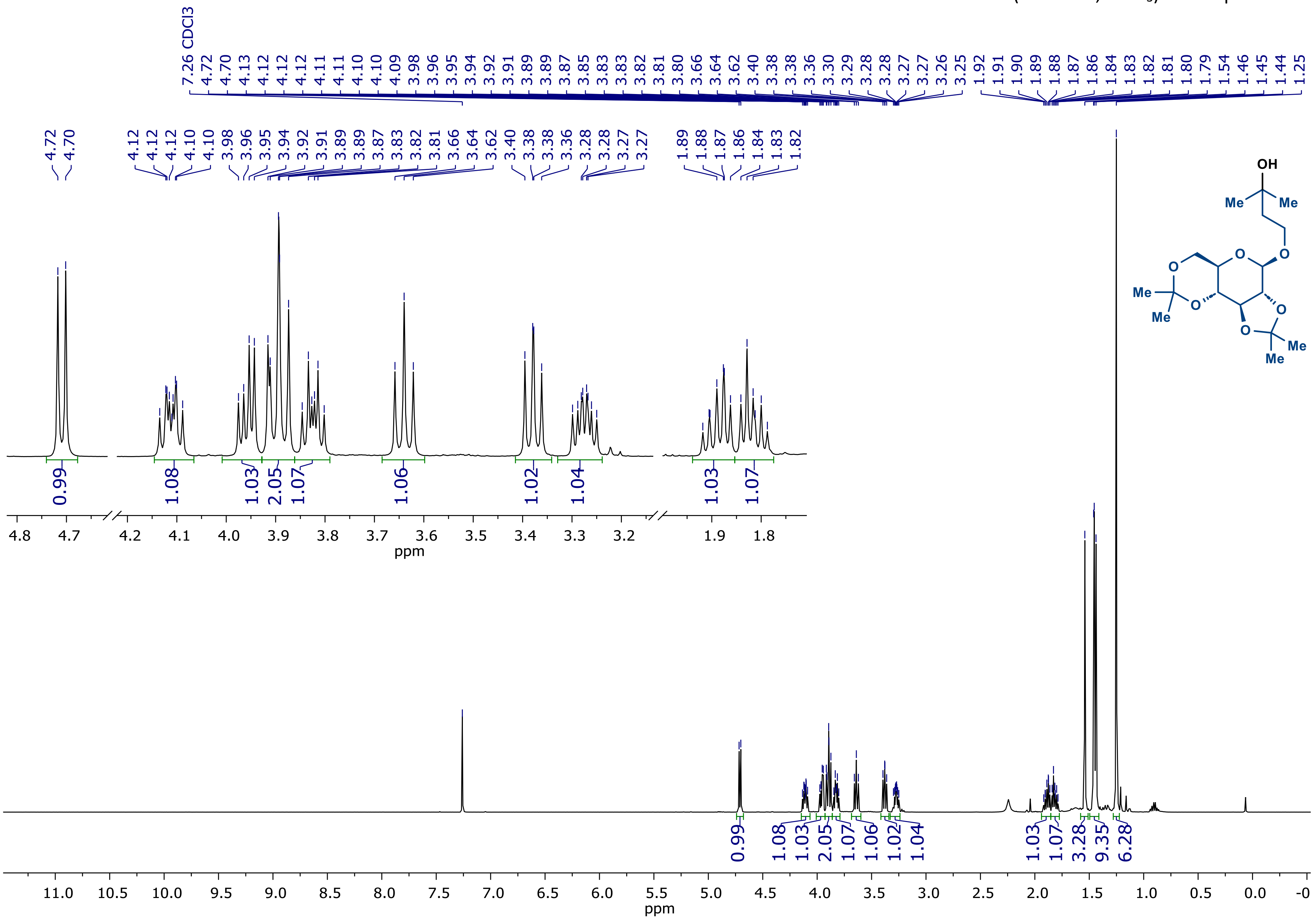

**$^{13}$C NMR** (126 MHz, $CDCl_3$) of compound **1w**

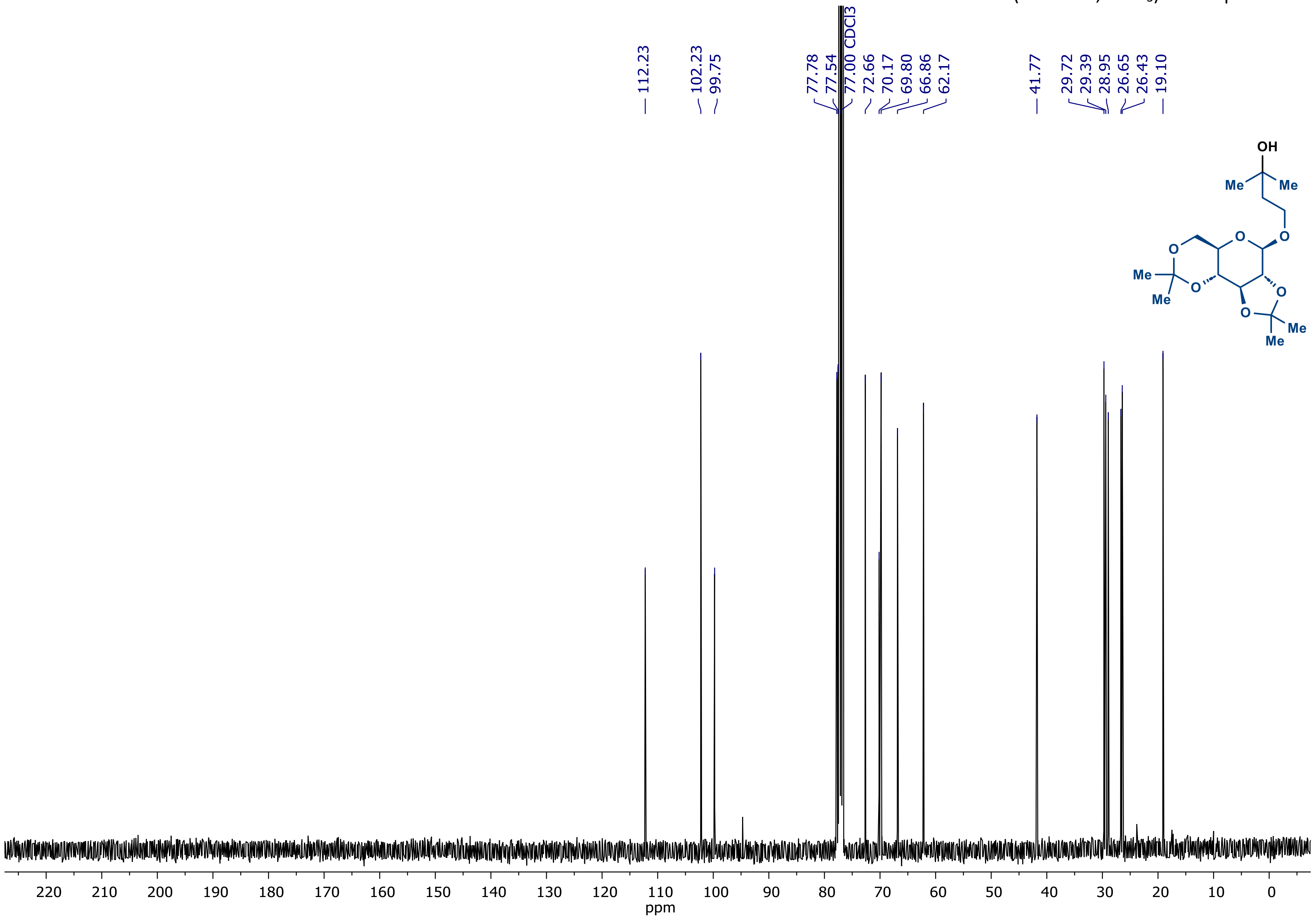

**COSY** of compound **1w**

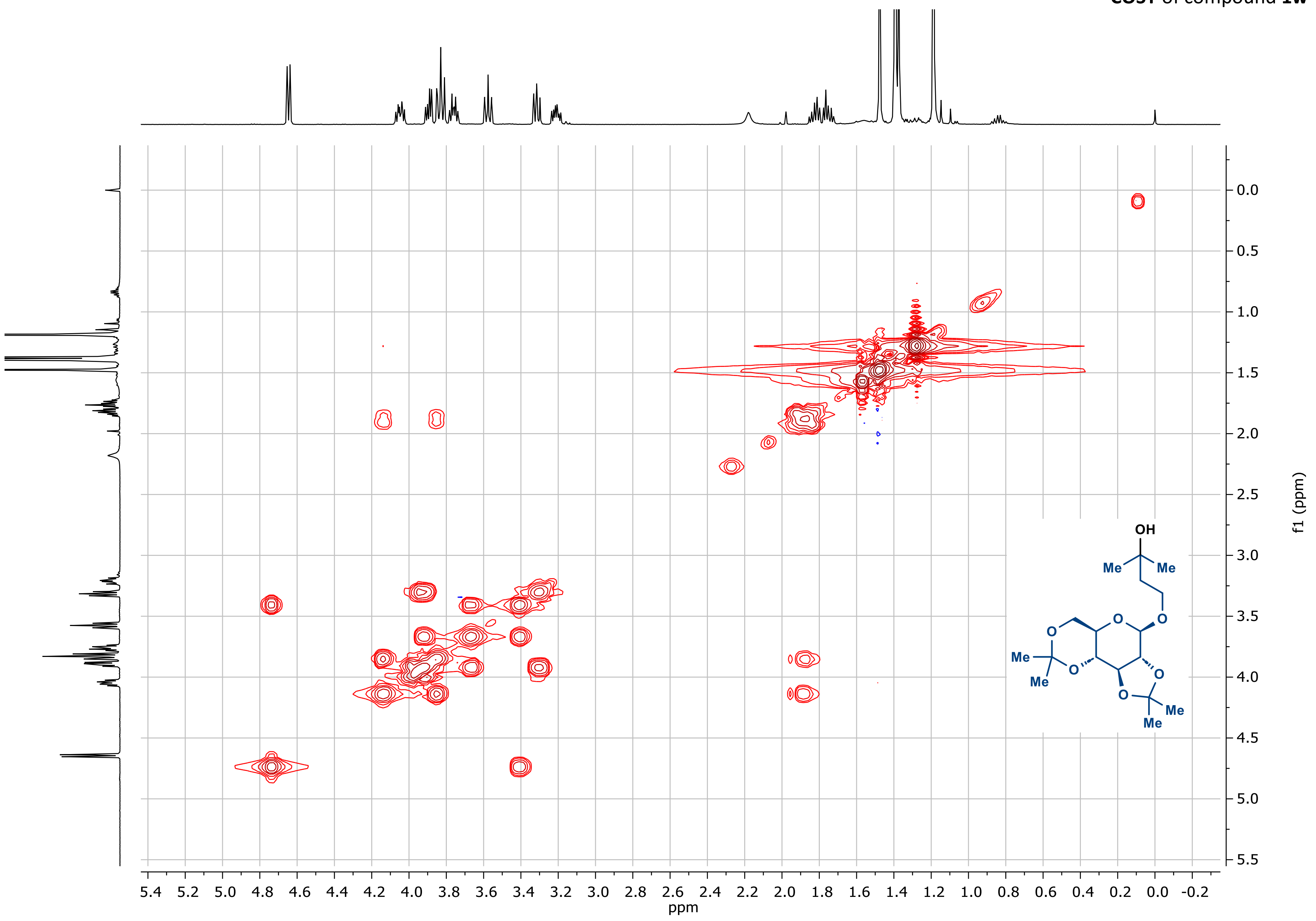

**HSQC** of compound **1w**

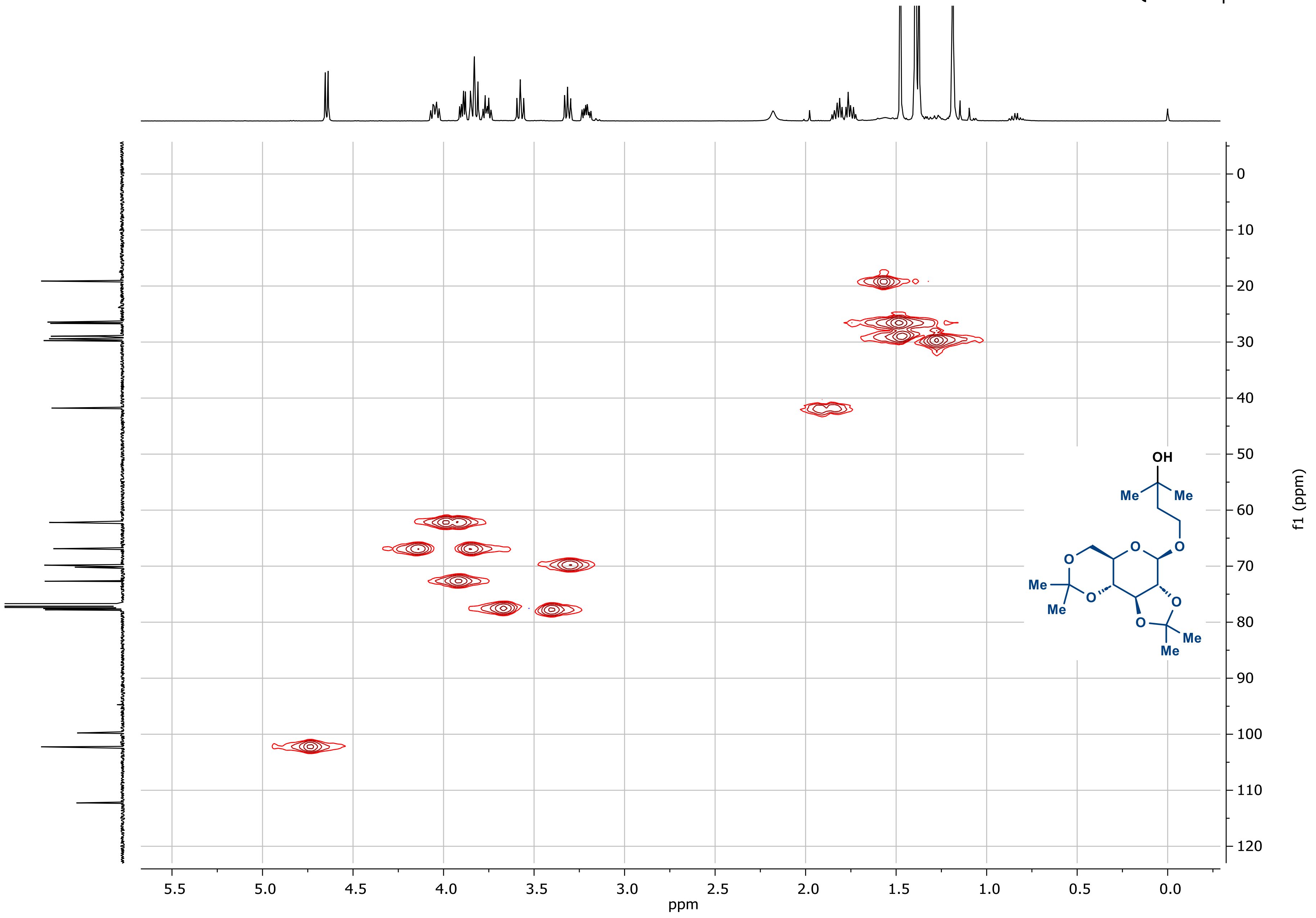

**$^1$H NMR** (500 MHz, $CDCl_3$) of compound **2a**

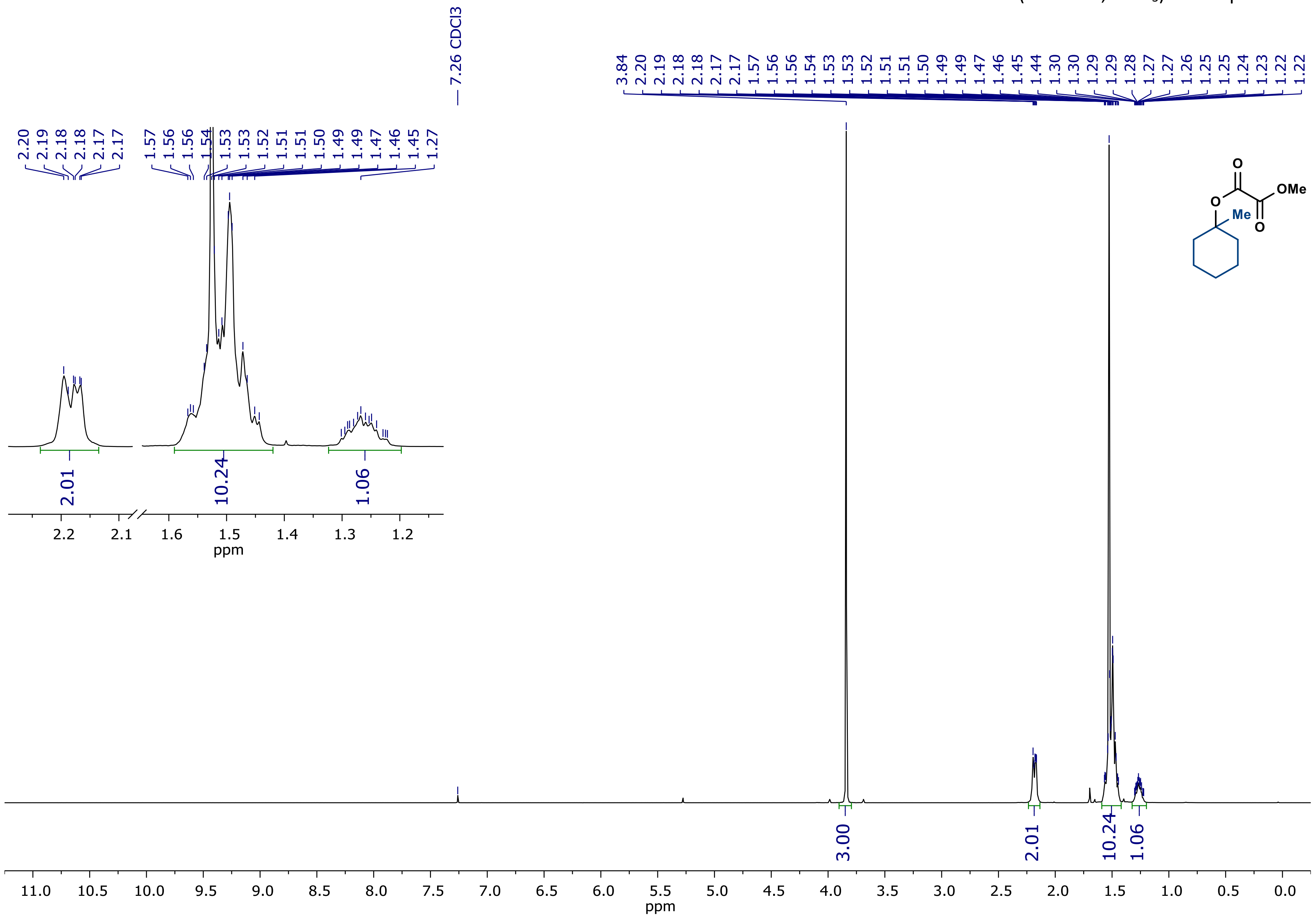

$^{13}$**C NMR** (126 MHz, $CDCl_3$) of compound **2a**

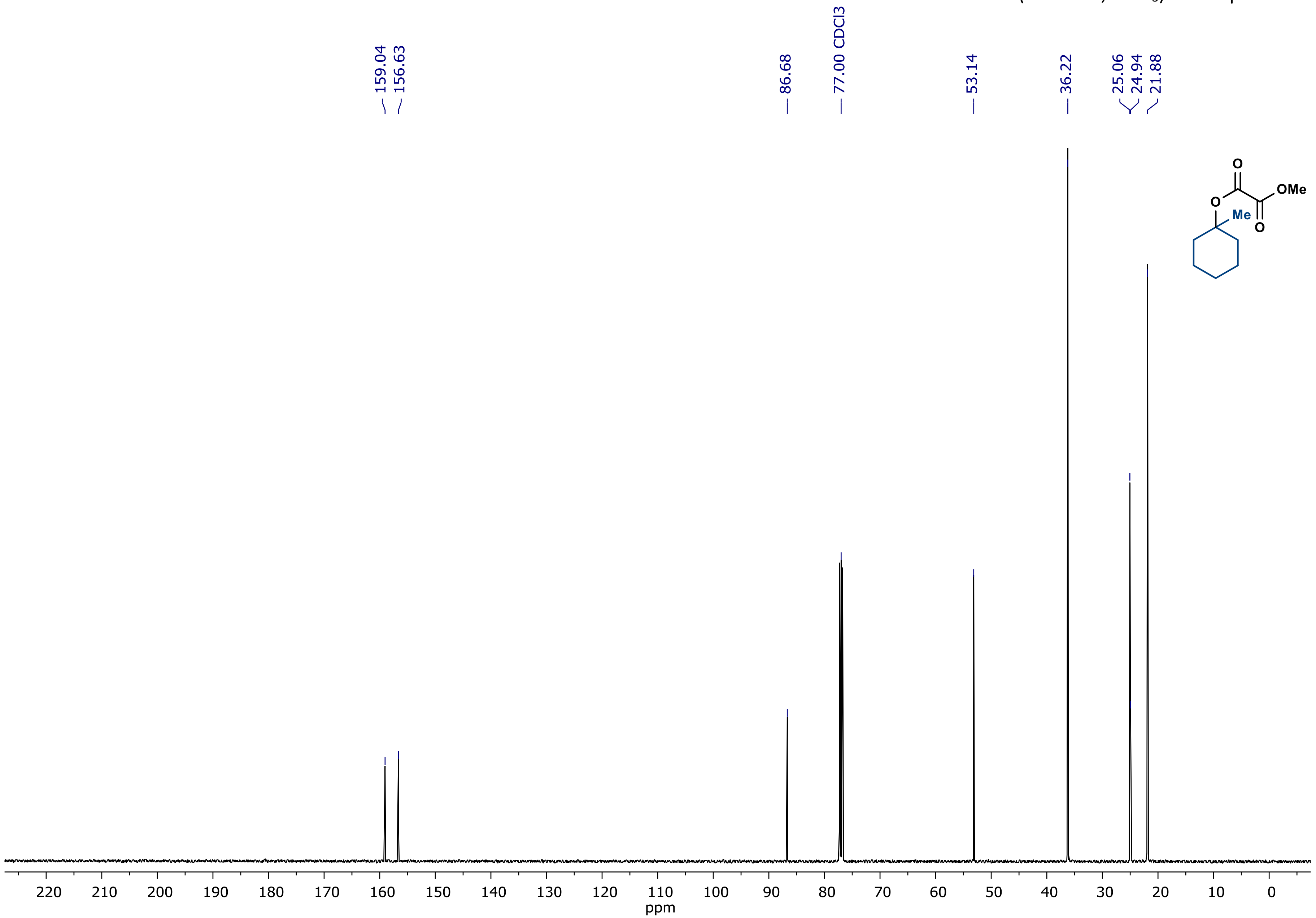

**$^{1}$H NMR** (500 MHz, $CDCl_3$) of compound **2b**

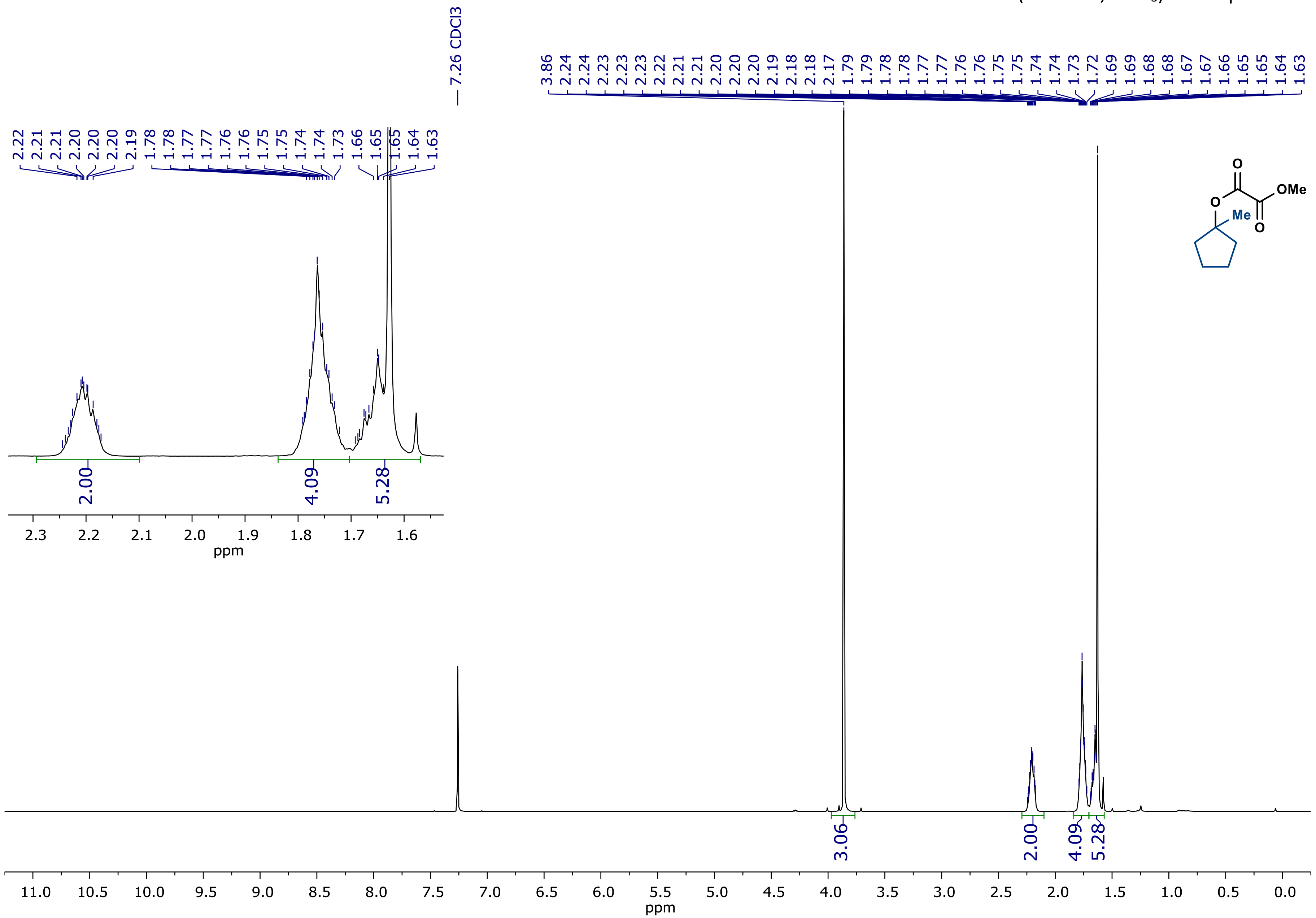

**$^{13}$C NMR** (126 MHz, $CDCl_3$) of compound **2b**

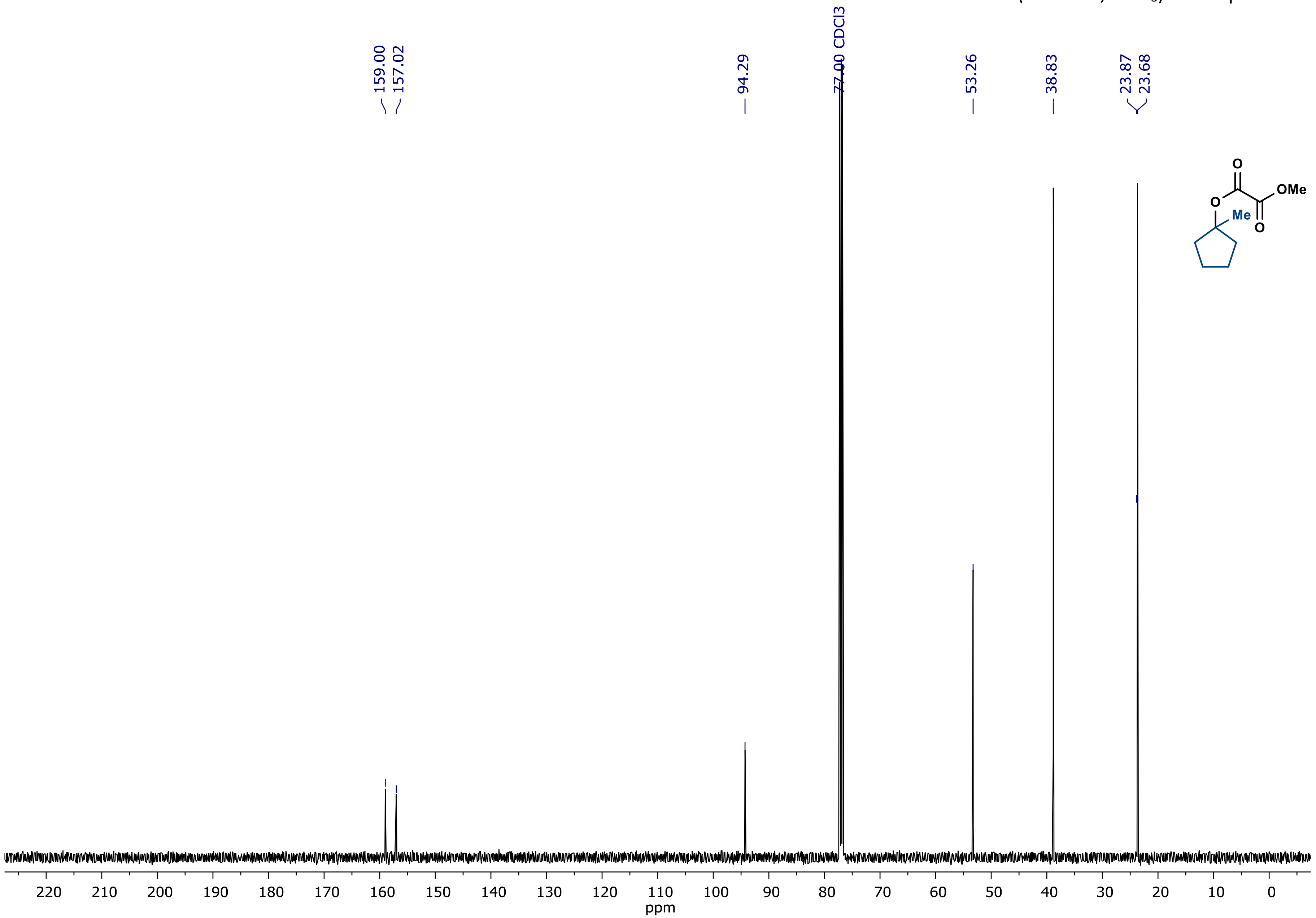

$^{1}$H NMR (500 MHz, $CDCl_3$) of compound **2c**

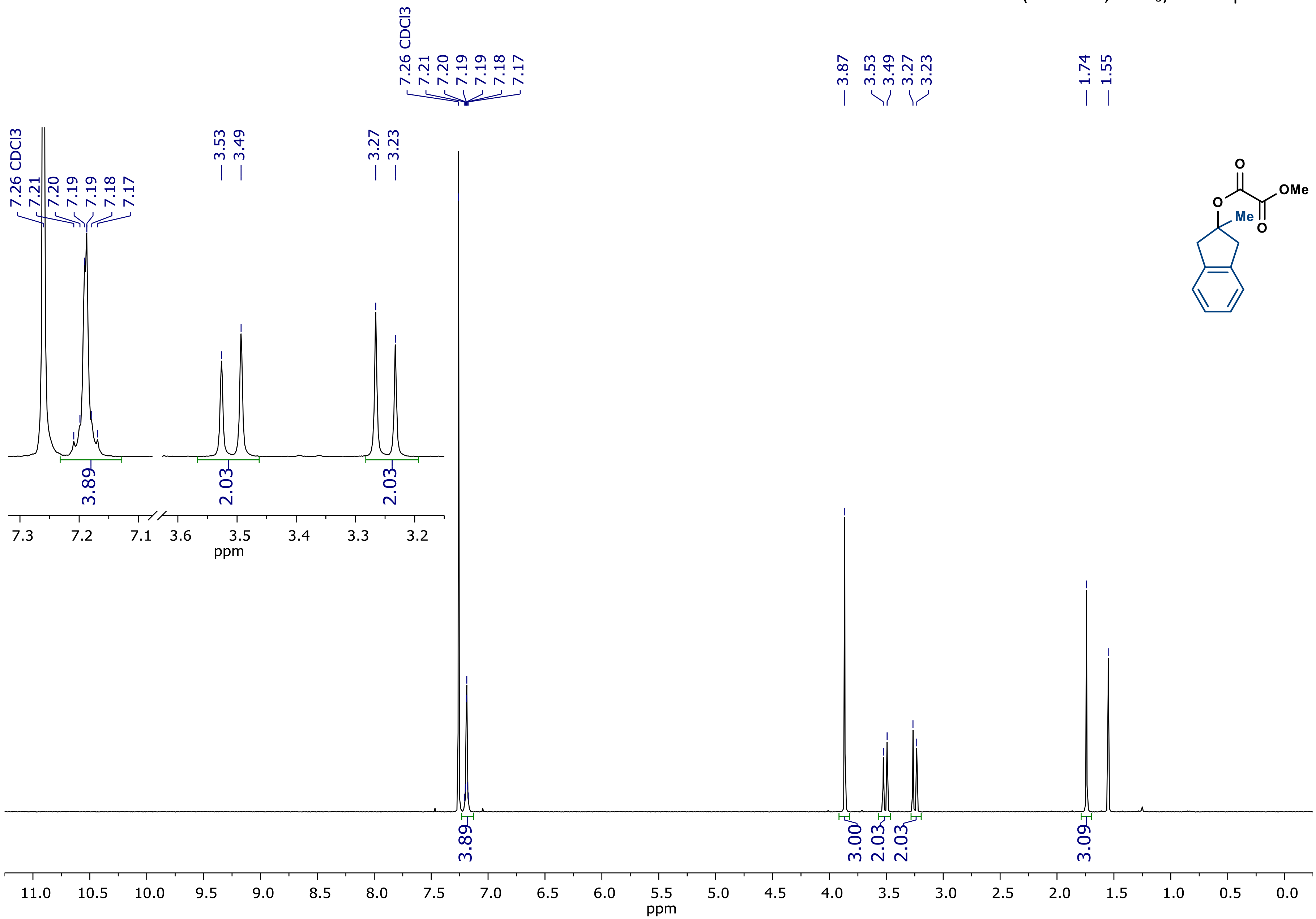

$^{13}$**C NMR** (126 MHz, $CDCl_3$) of compound **2c**

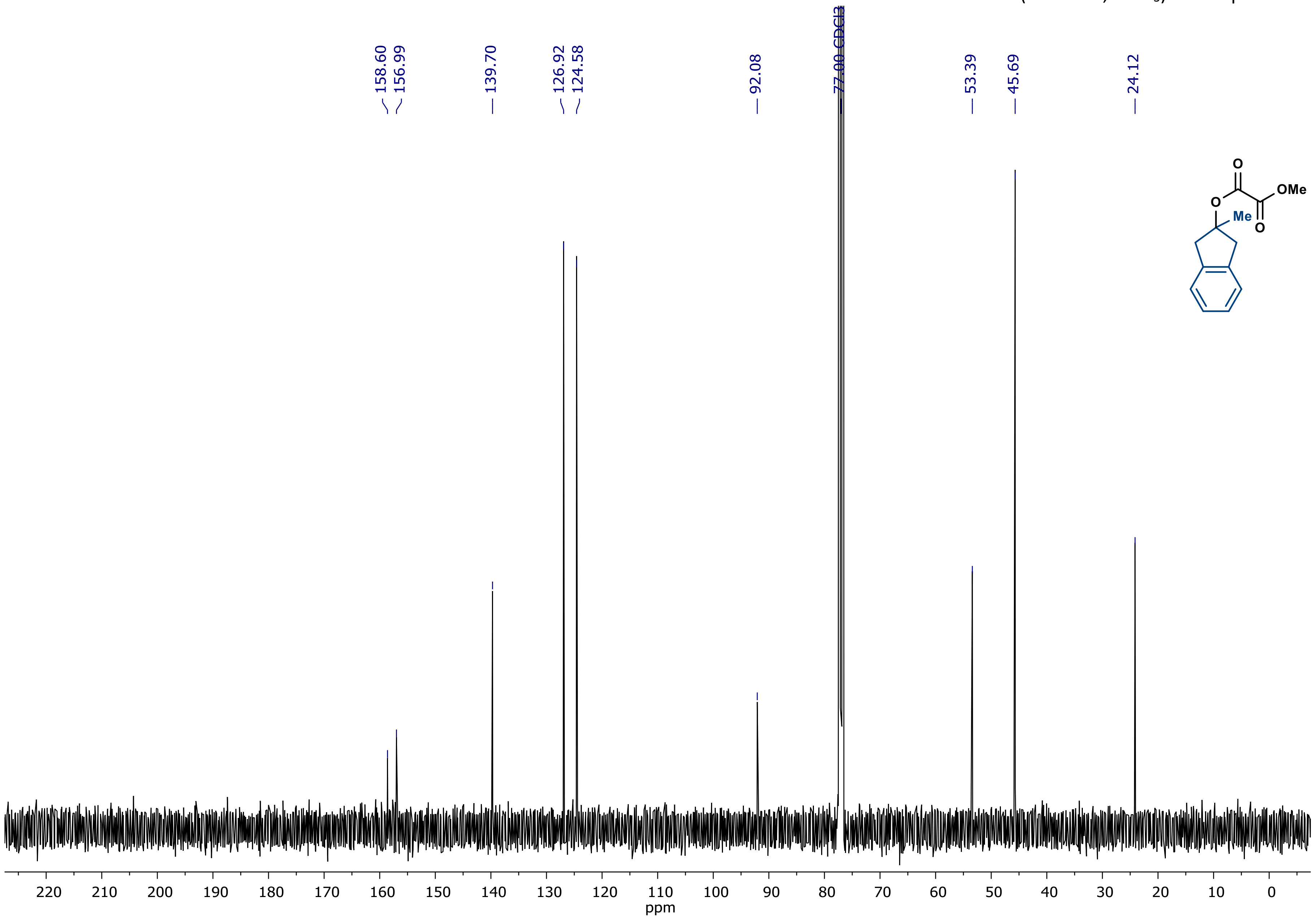

**$^{1}$H NMR** (500 MHz, $CDCl_3$) of compound **2d**

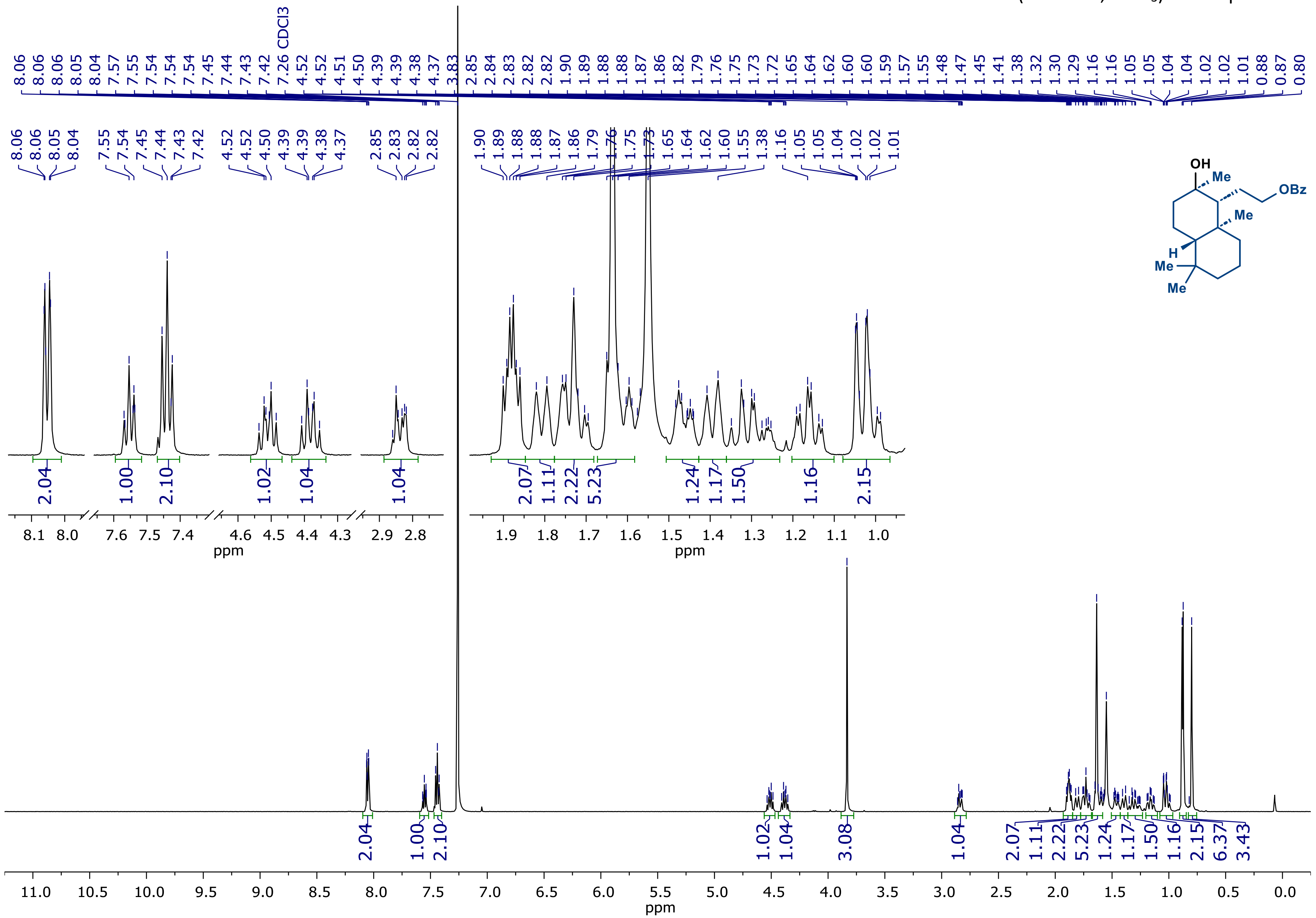

$^{13}$**C NMR** (126 MHz, $CDCl_3$) of compound **2d**

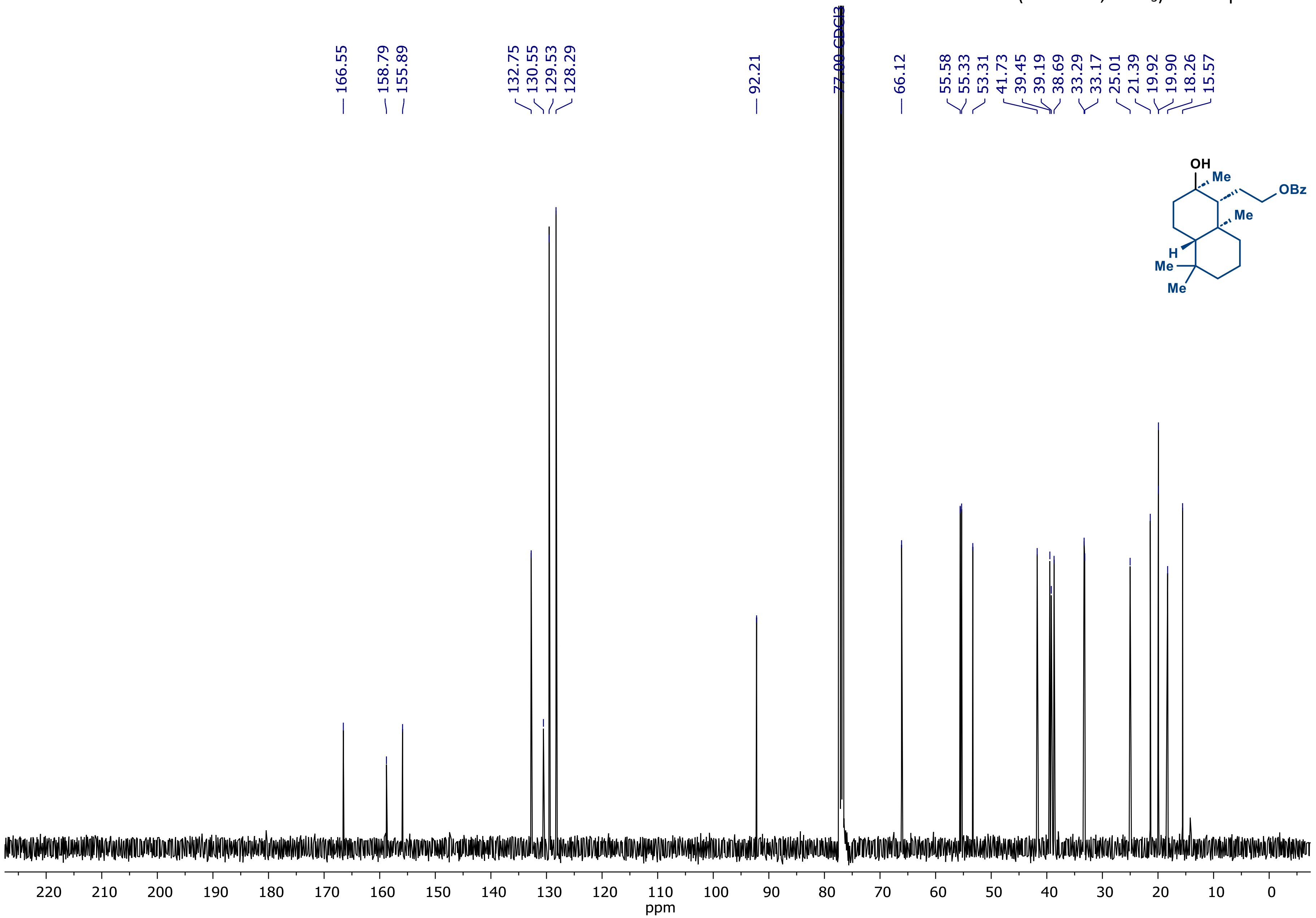

**$^1$H NMR** (500 MHz, $CDCl_3$) of compound **2e**

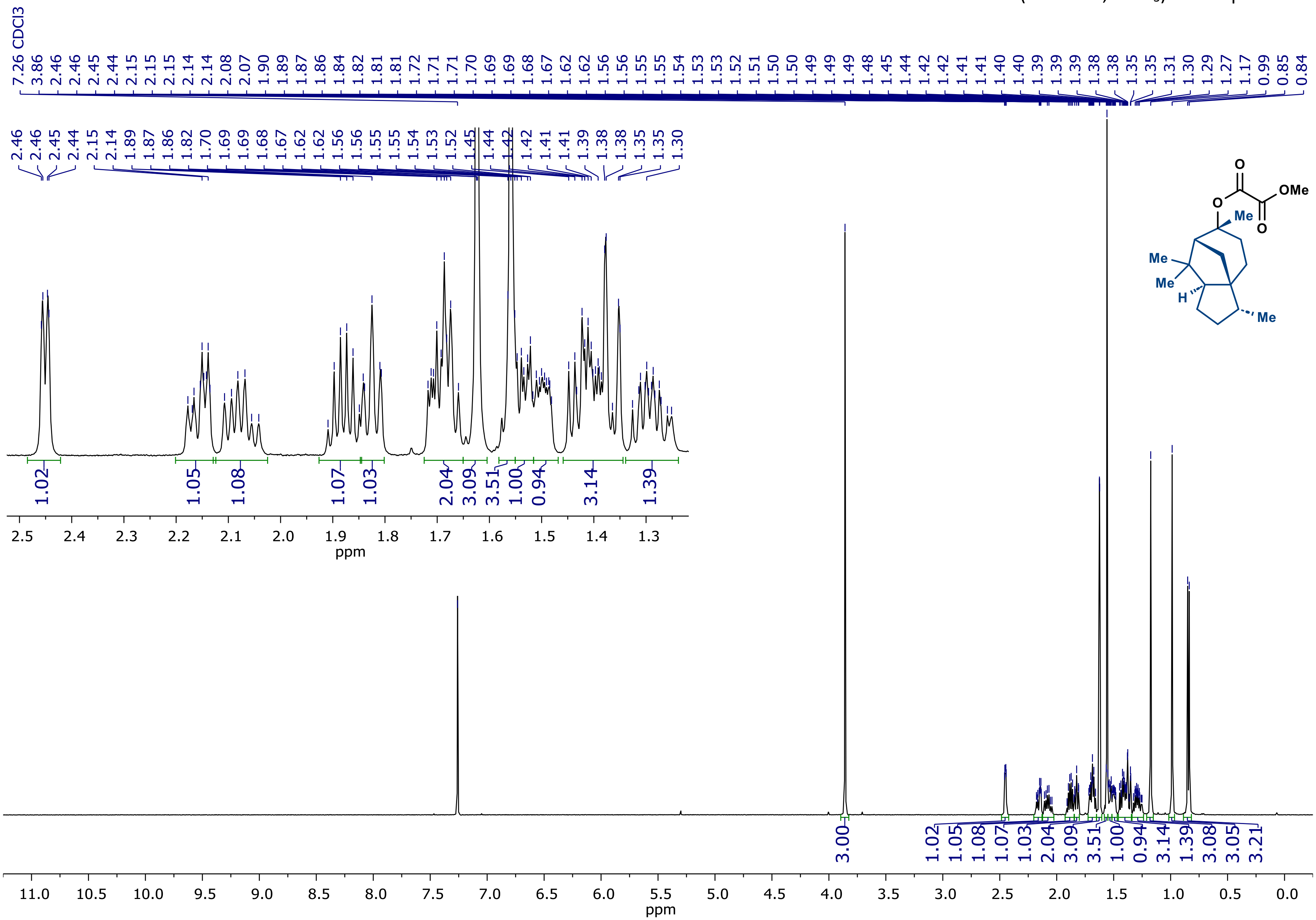

**$^{13}$C NMR** (126 MHz, $CDCl_3$) of compound **2e**

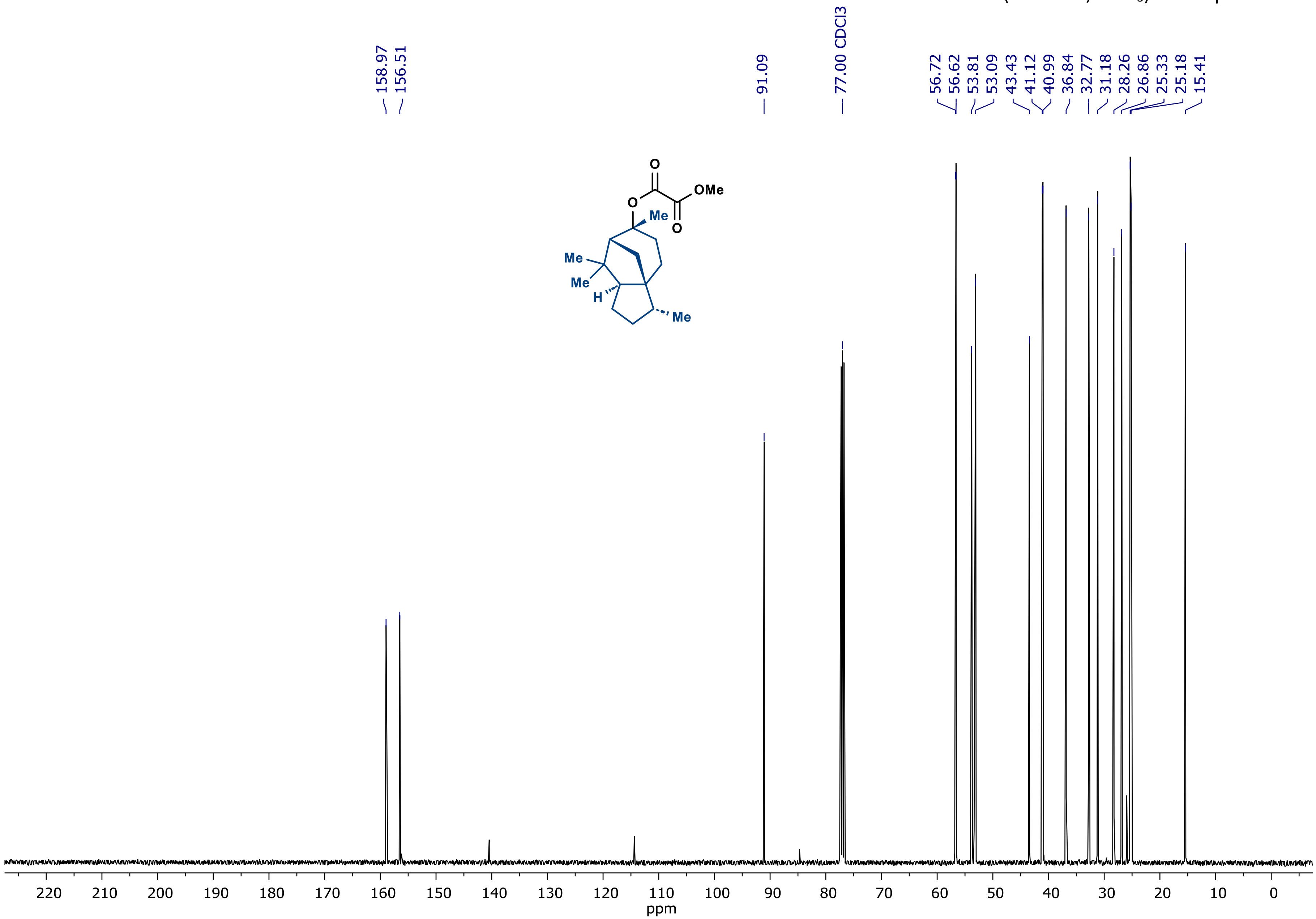

**$^{1}$H NMR** (500 MHz, $CDCl_3$) of compound **2f**

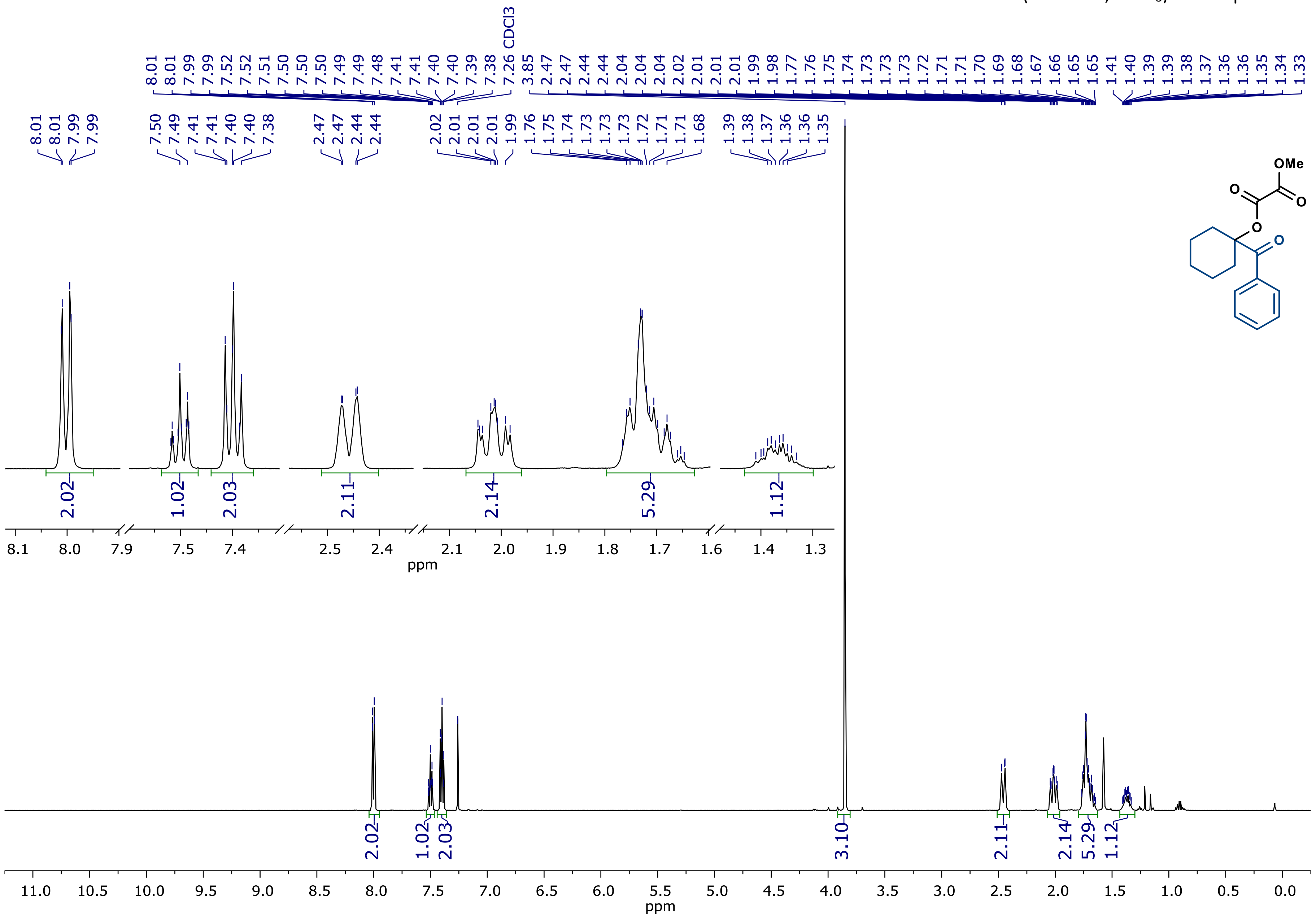

$^{13}$**C NMR** (126 MHz, $CDCl_3$) of compound **2f**

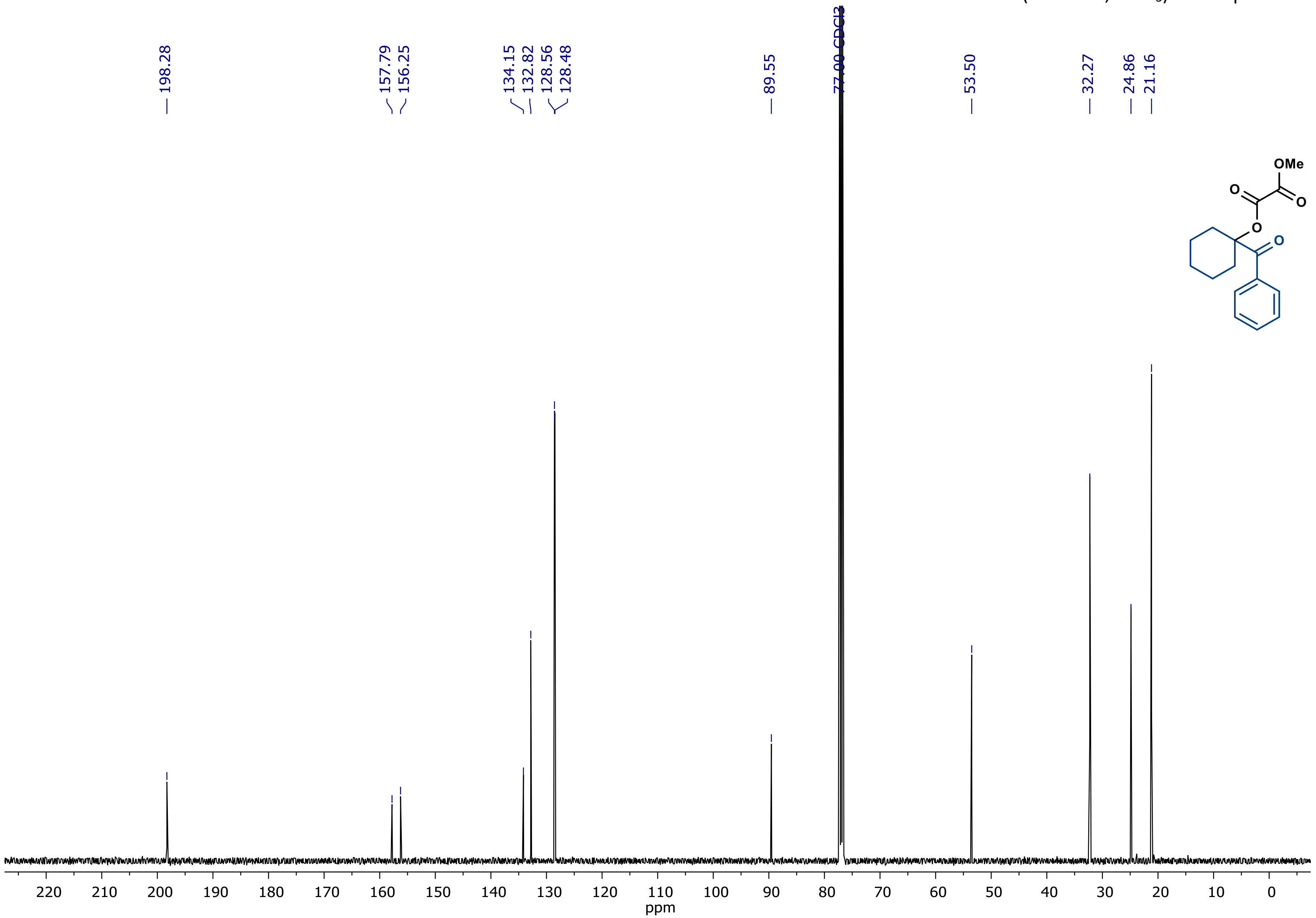

**$^1$H NMR** (500 MHz, $CDCl_3$) of compound **2g**

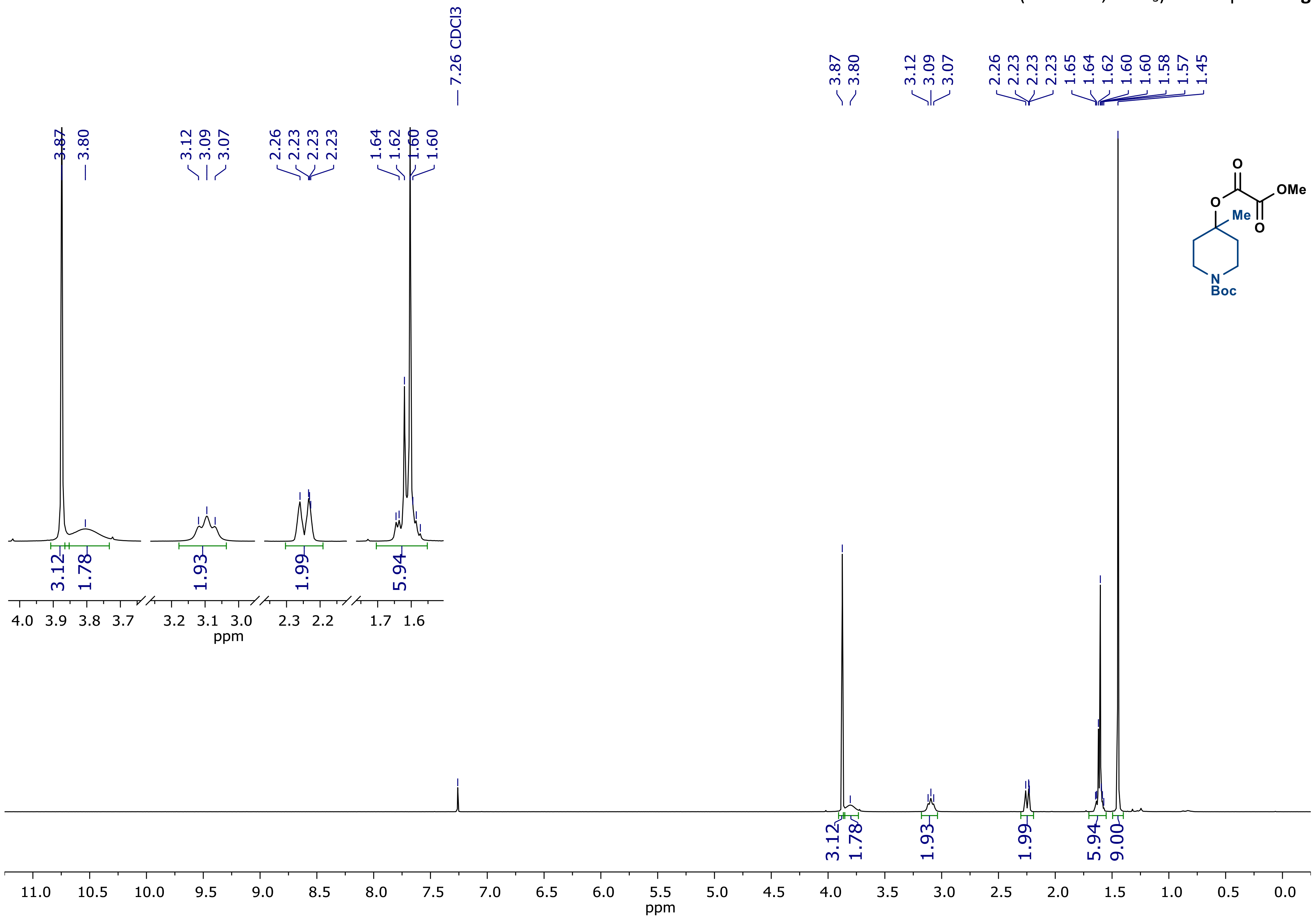

$^{13}$**C NMR** (126 MHz, $CDCl_3$) of compound **2g**

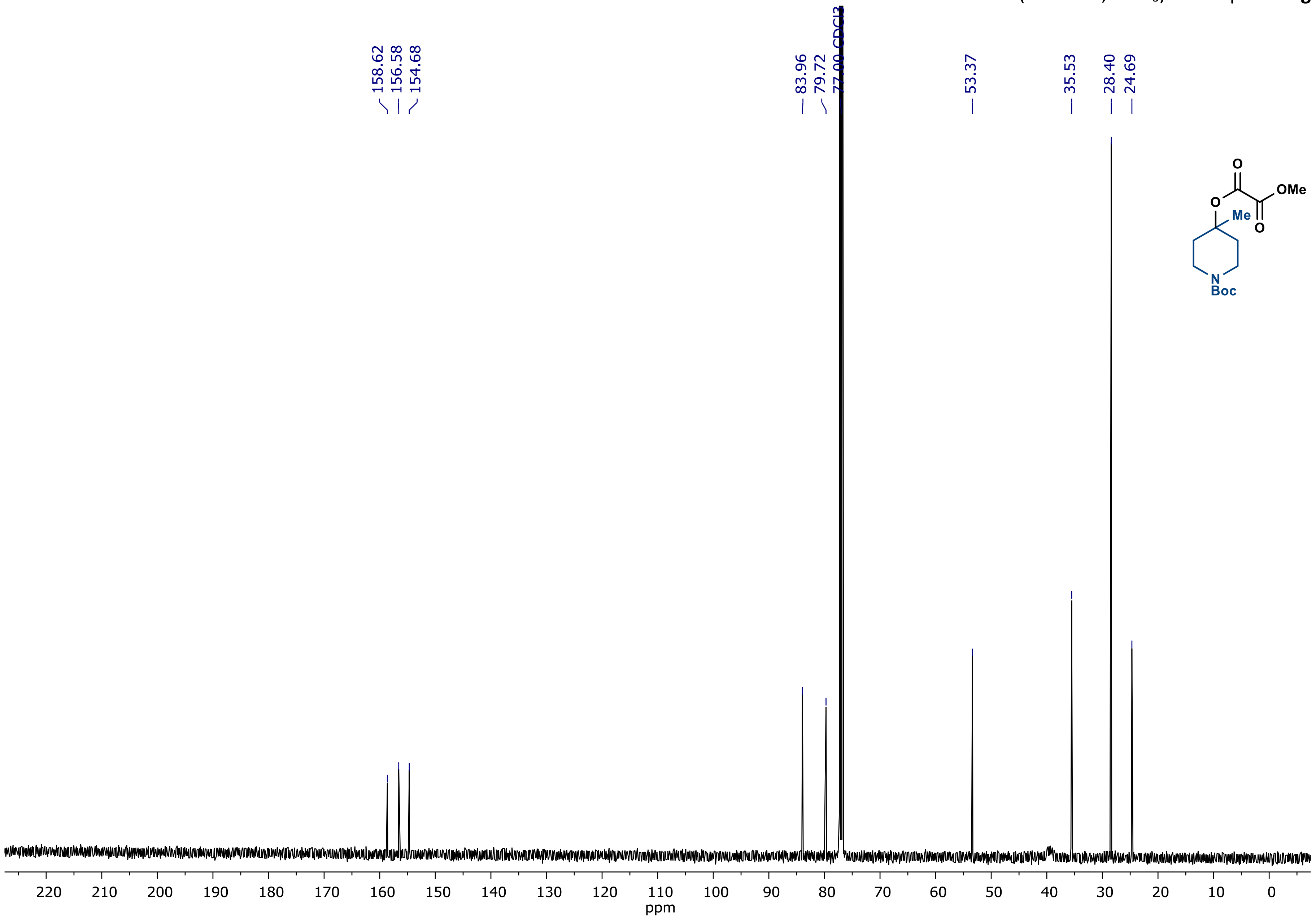

**$^1$H NMR** (500 MHz, $CDCl_3$) of compound **2h**

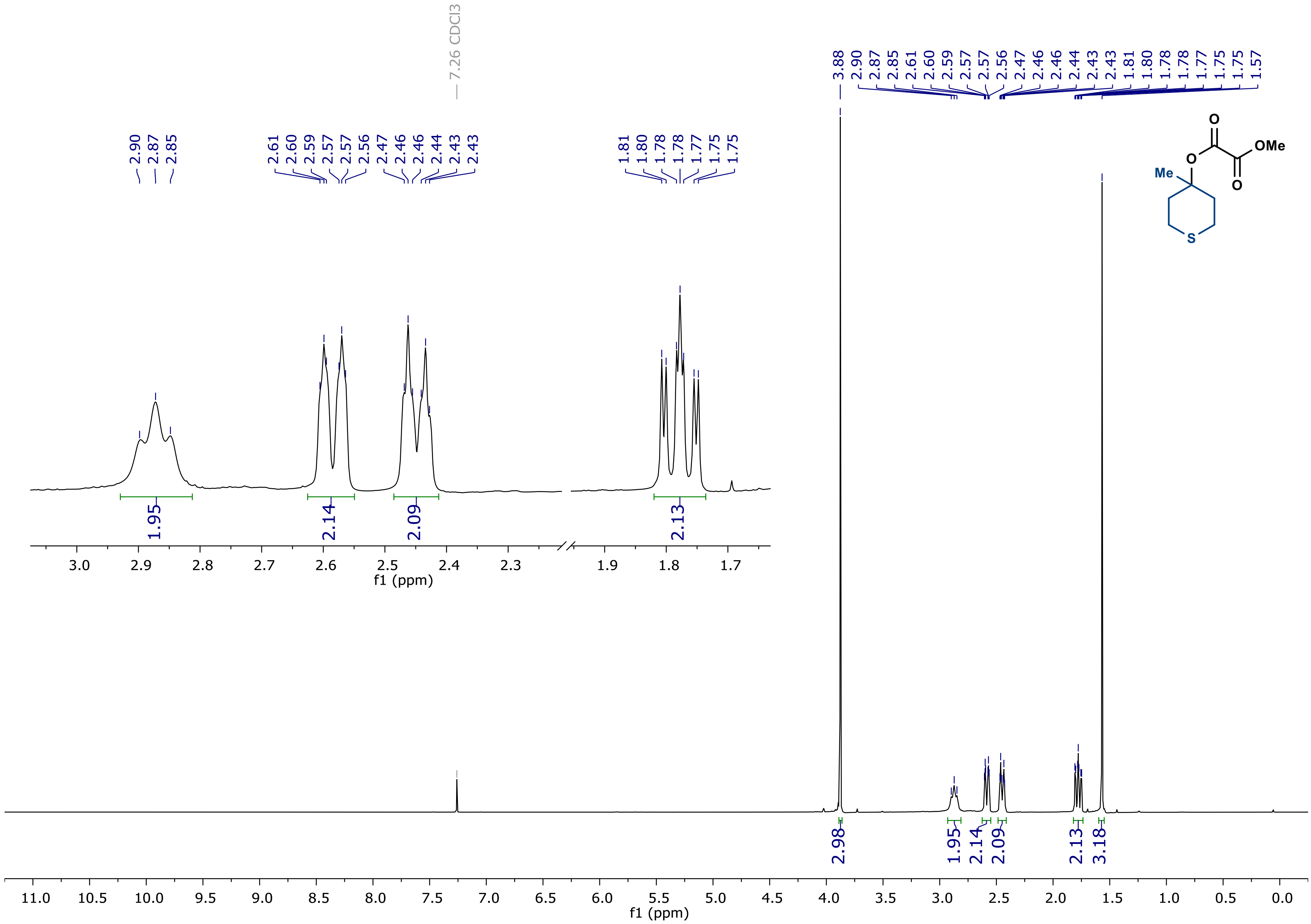

$^{13}$**C NMR** (126 MHz, $CDCl_3$) of compound **2h**

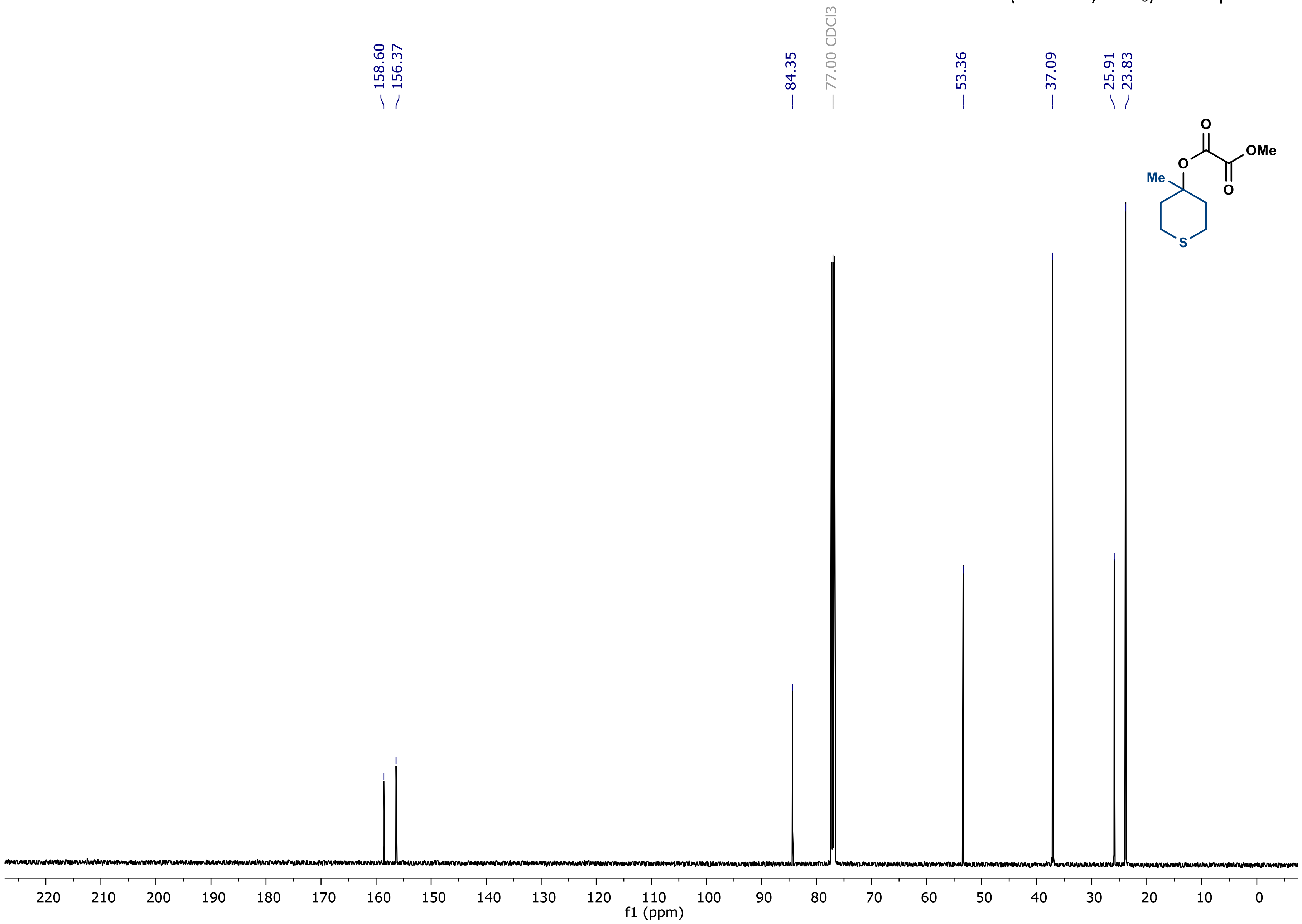

**$^1$H NMR** (500 MHz, $CDCl_3$) of compound **2i**

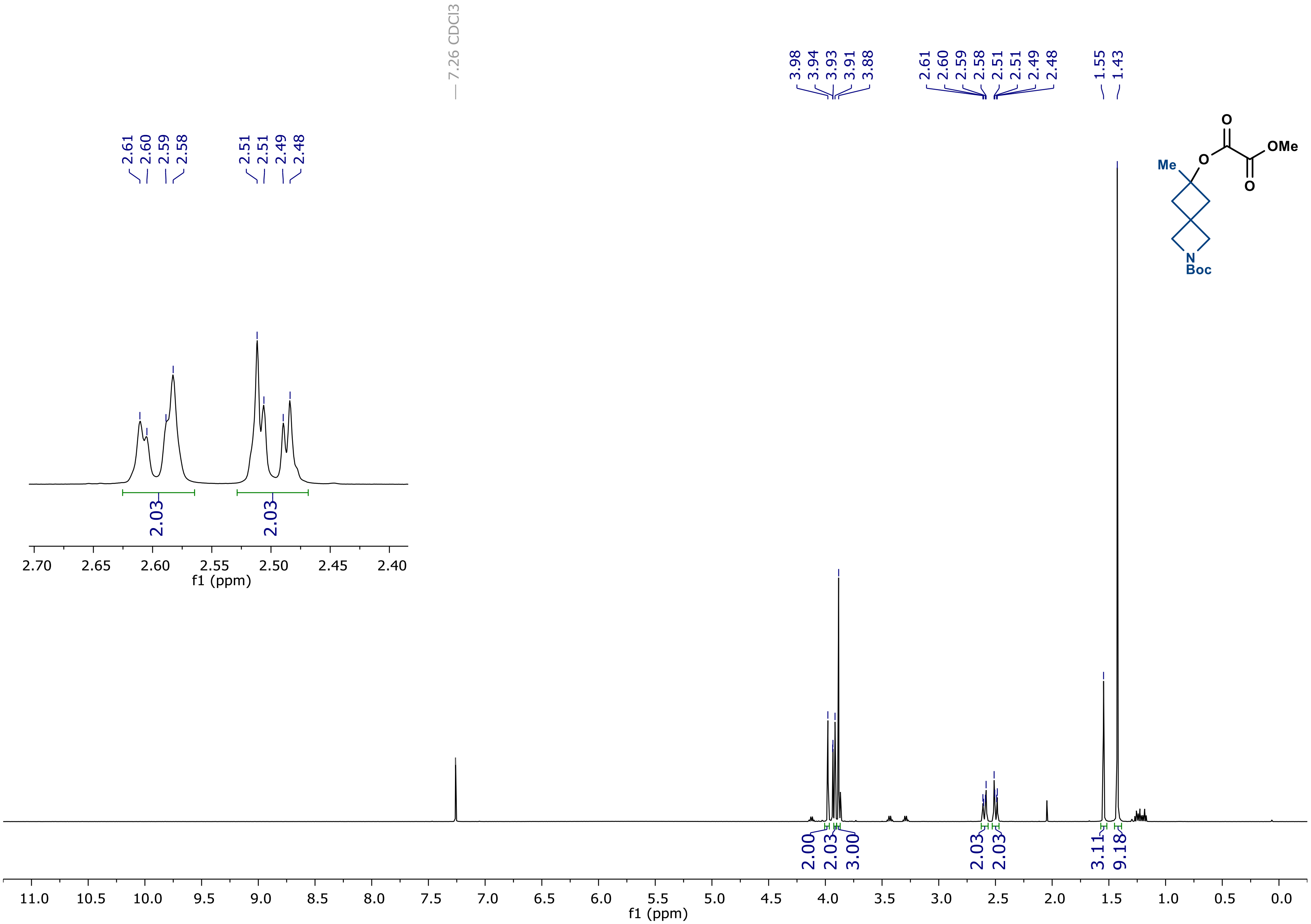

**$^{13}$C NMR** (126 MHz, $CDCl_3$) of compound **2i**

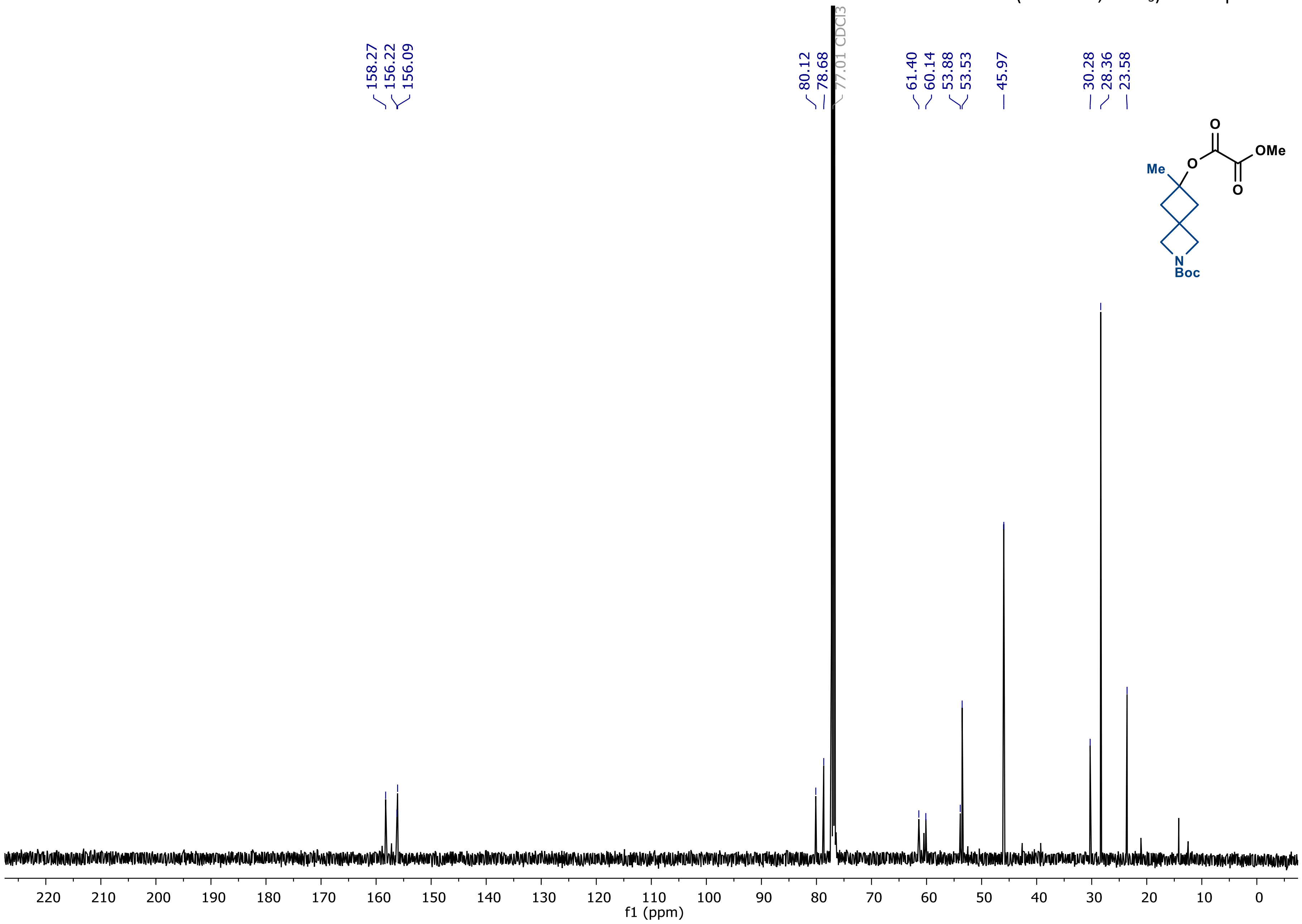

$^1$**H NMR** (500 MHz, $CDCl_3$) of compound **2j**

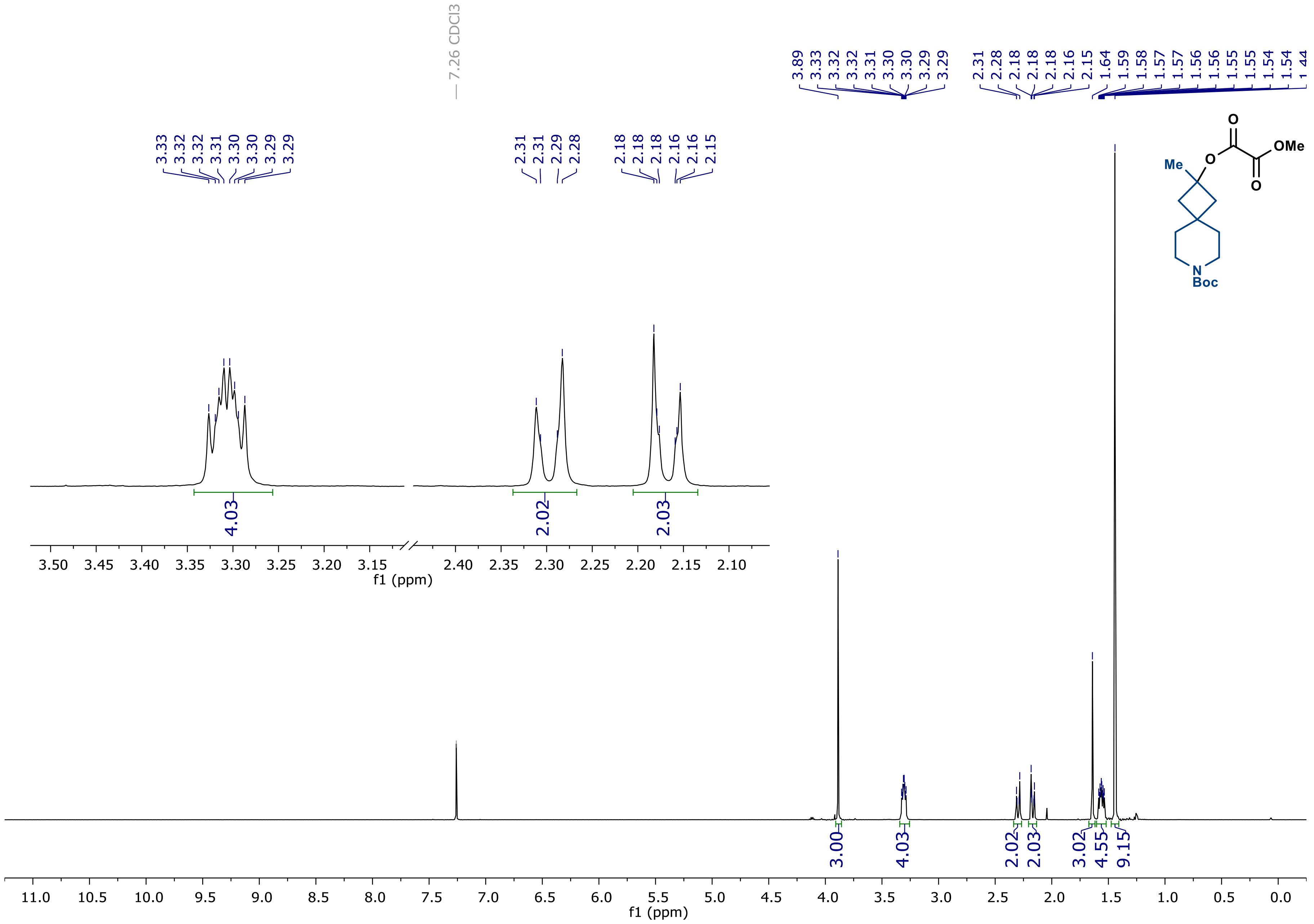

$^{13}$**C NMR** (126 MHz, $CDCl_3$) of compound **2j**

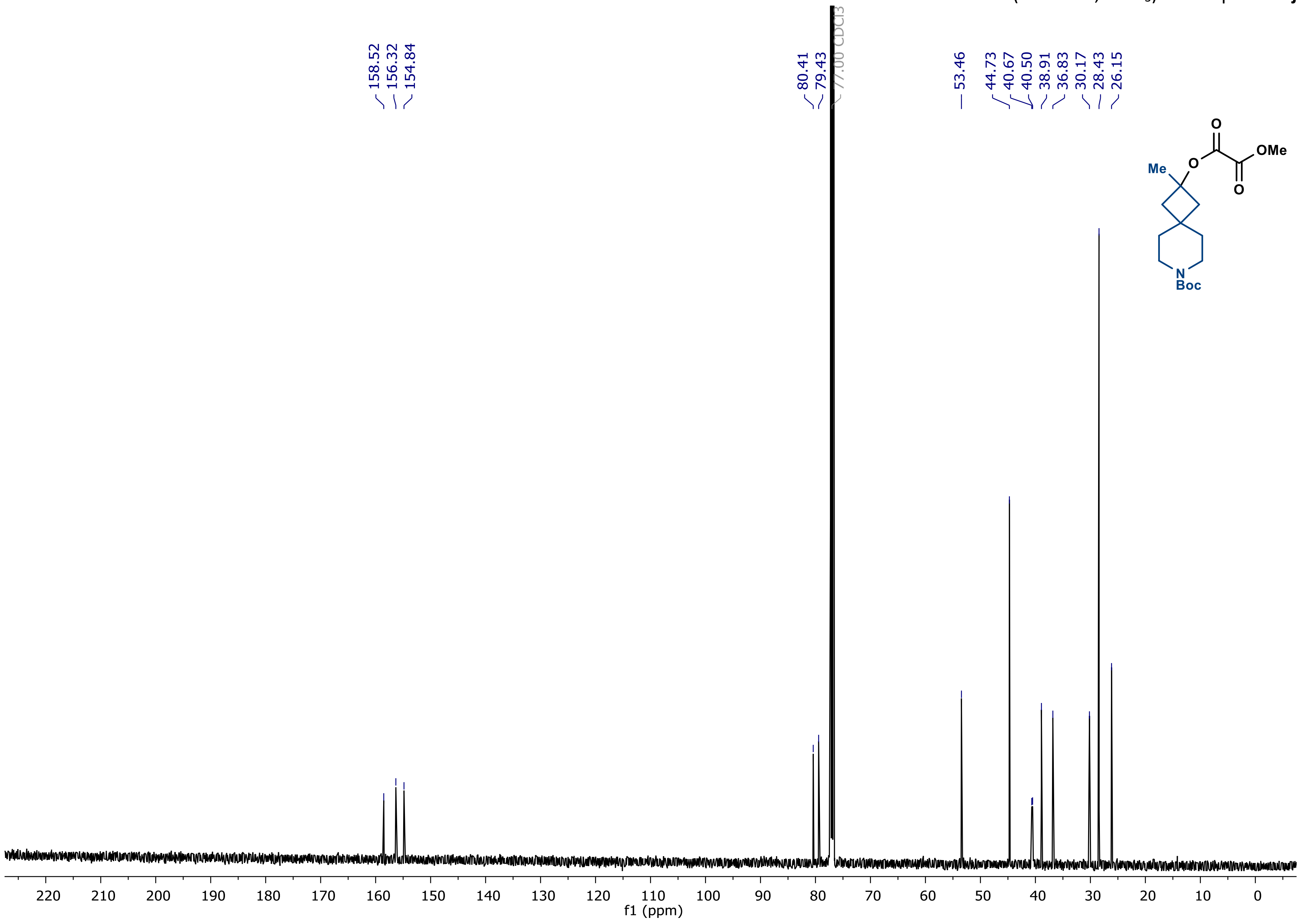

**$^{1}$H NMR** (500 MHz, $CDCl_3$) of compound **2k**

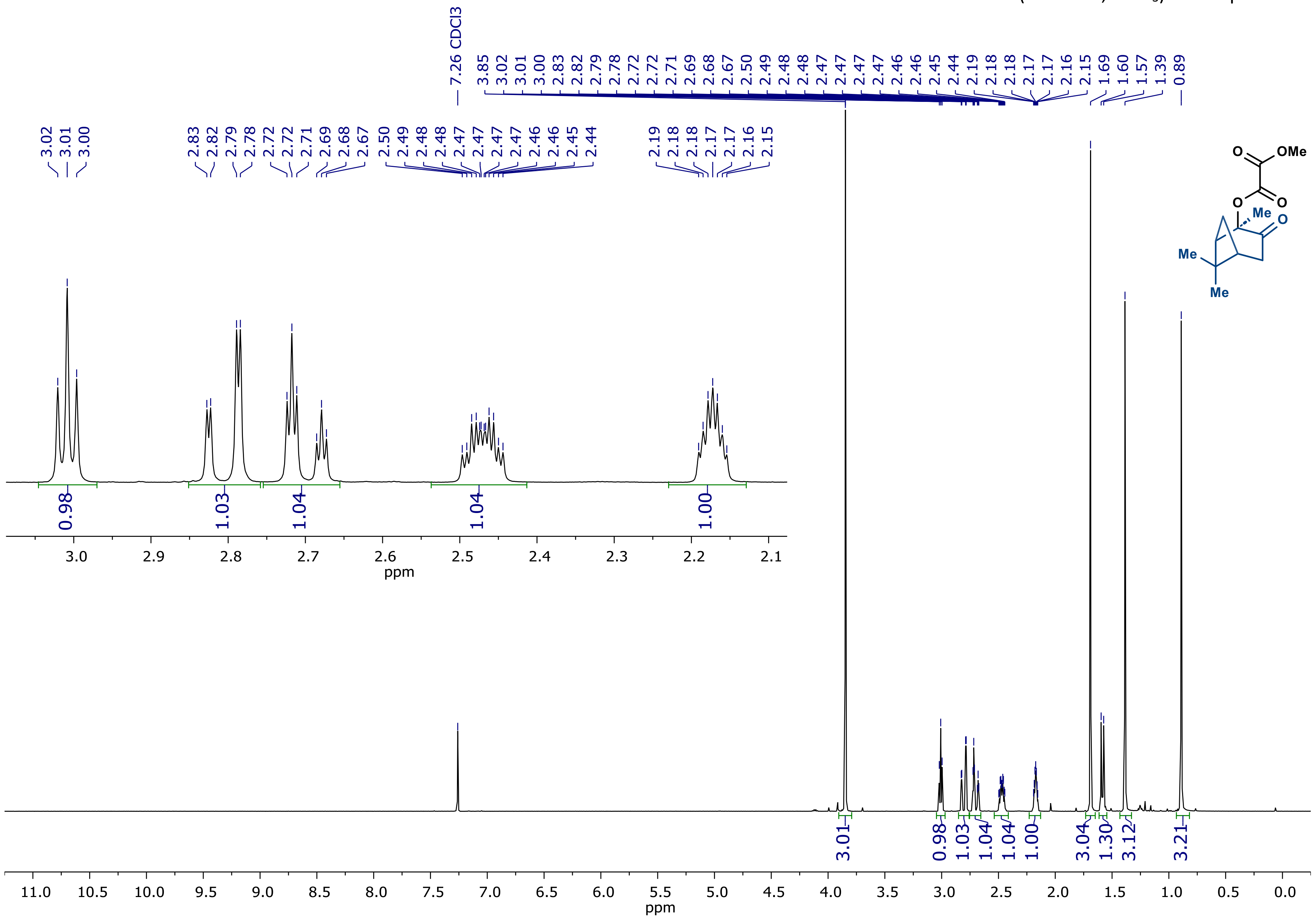

**$^{13}$C NMR** (126 MHz, $CDCl_3$) of compound **2k**

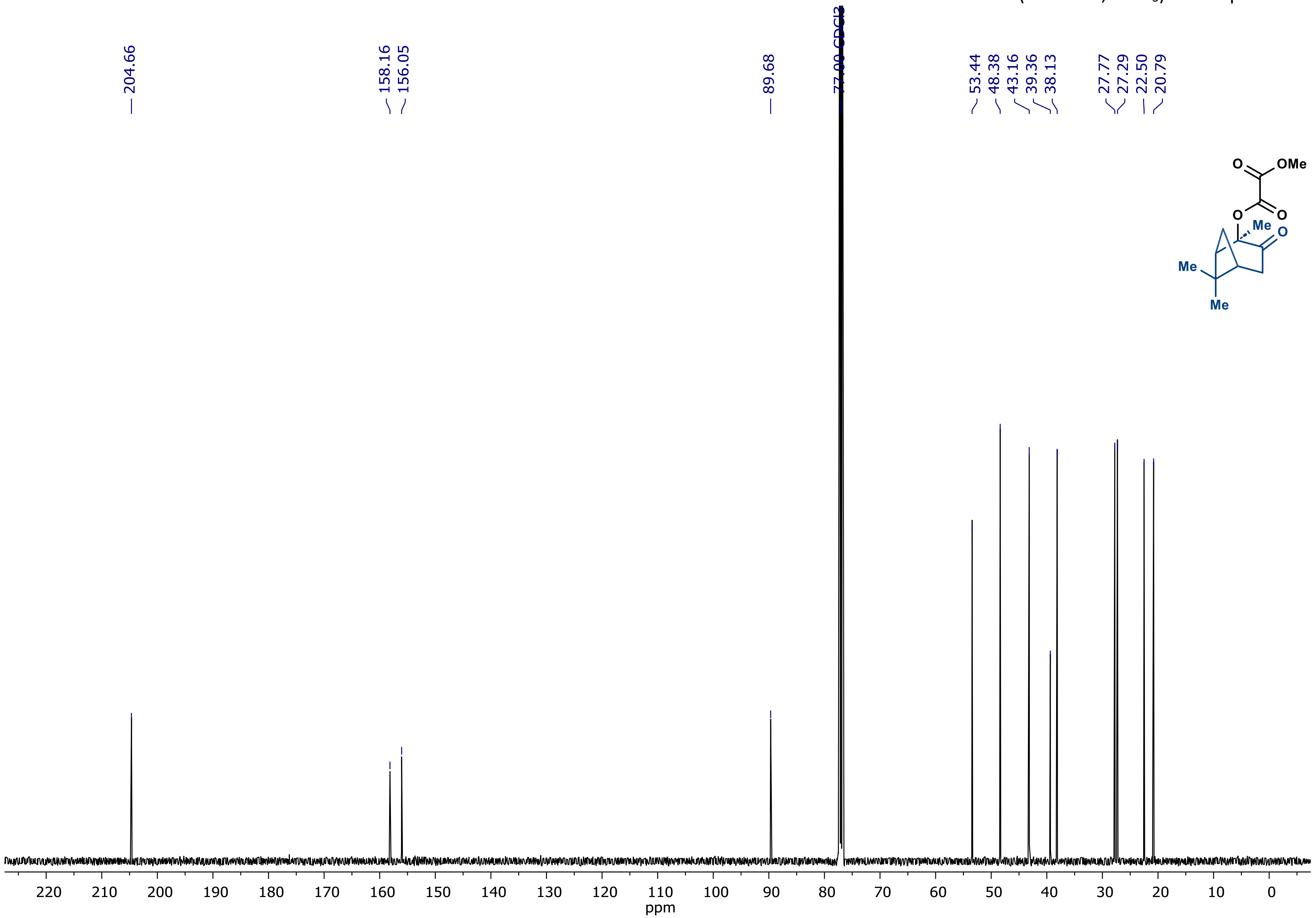

$^{1}$**H NMR** (500 MHz, $CDCl_3$) of compound **2l**

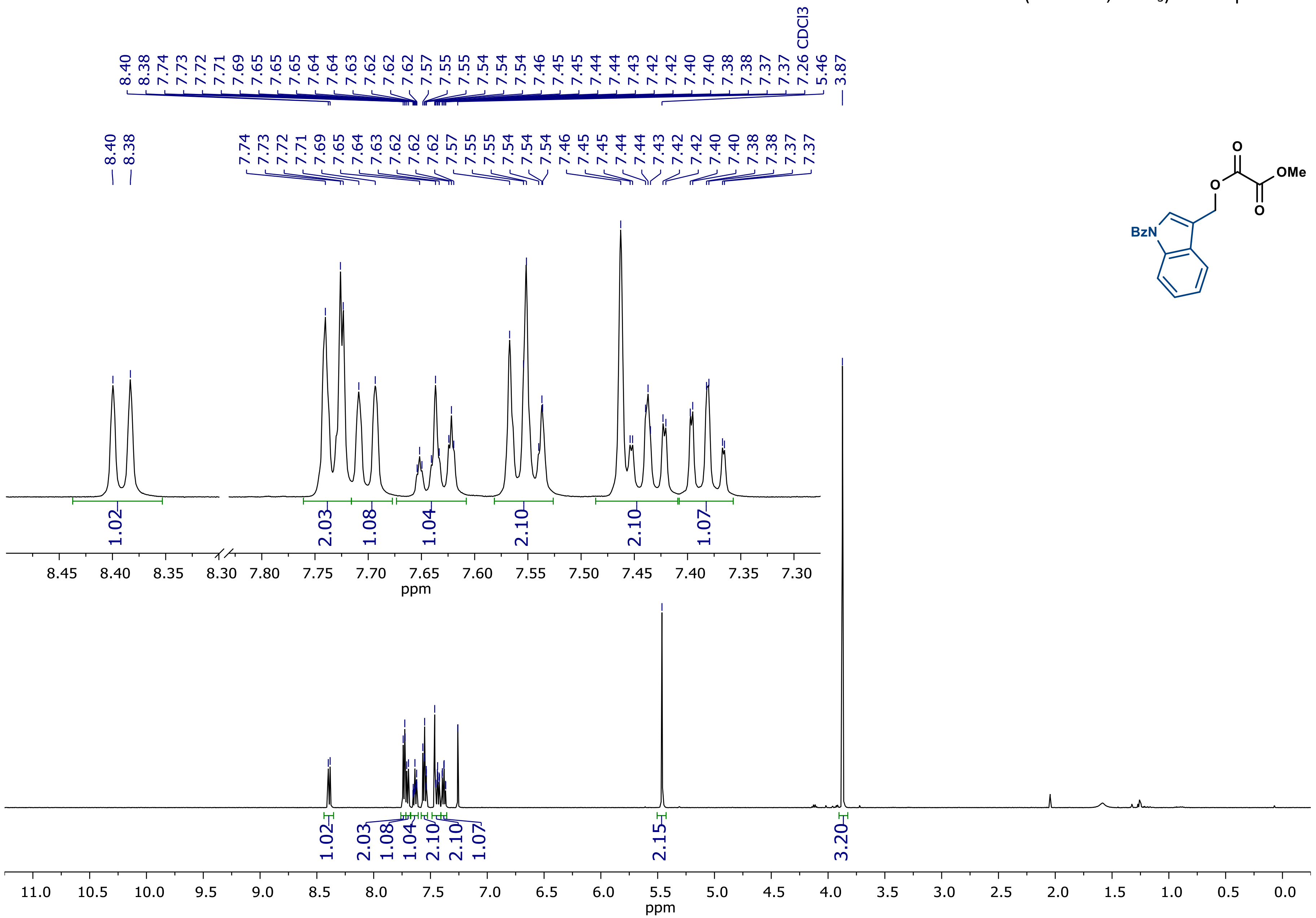

**$^{13}$C NMR** (126 MHz, $CDCl_3$) of compound **2l**

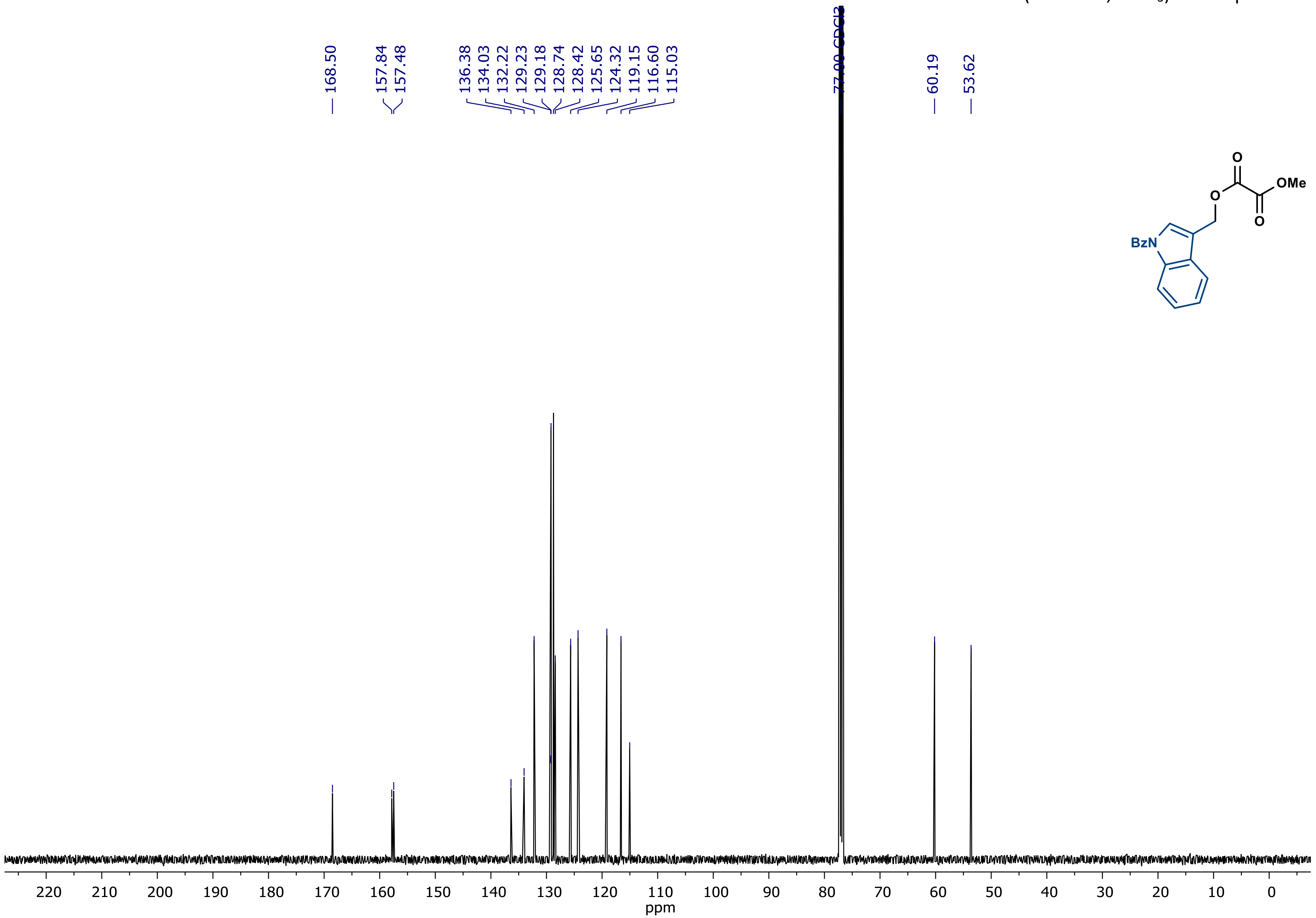

**$^1$H NMR** (500 MHz, $CDCl_3$) of compound **2m**

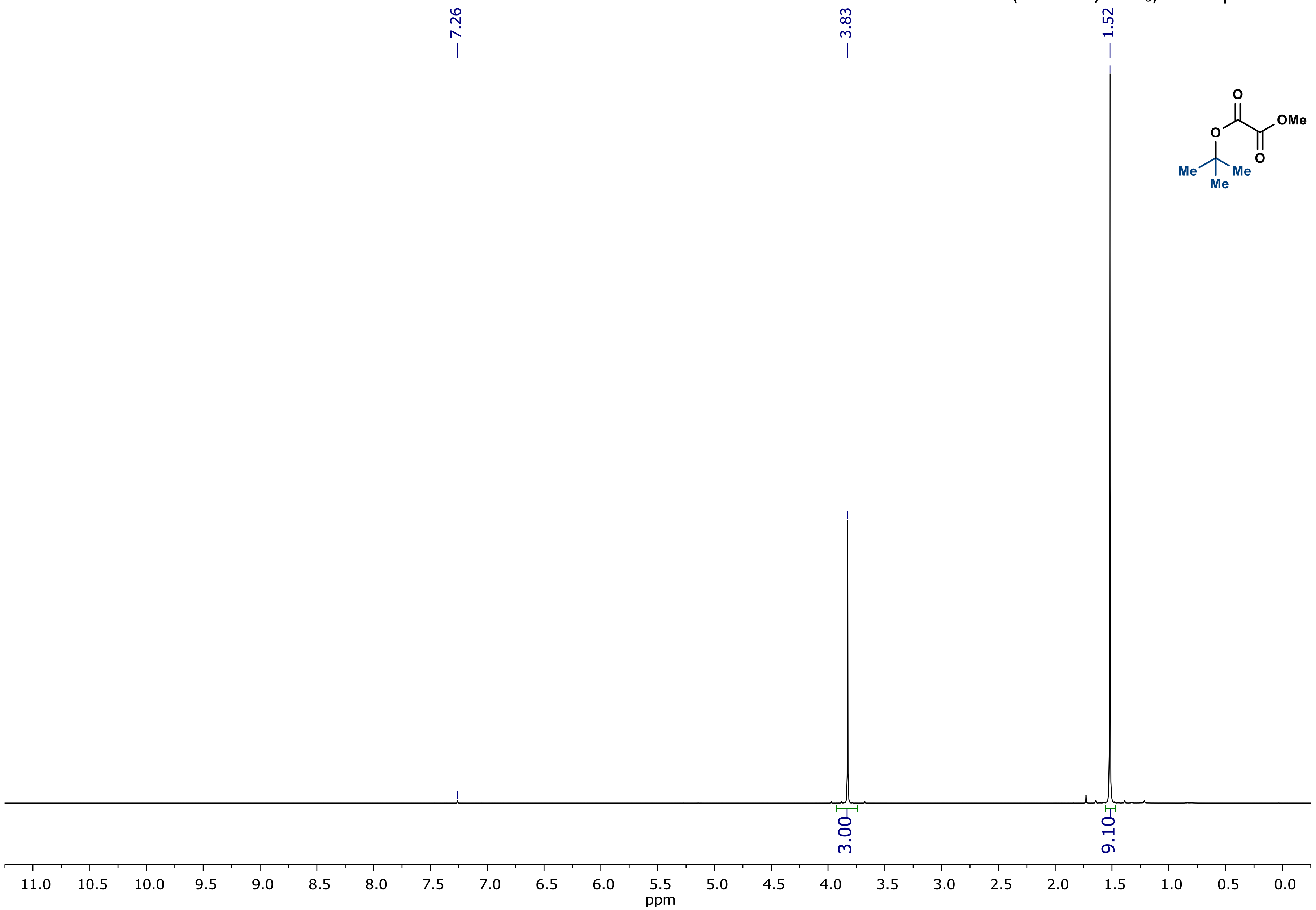

$^{13}$**C NMR** (126 MHz, $CDCl_3$) of compound **2m**

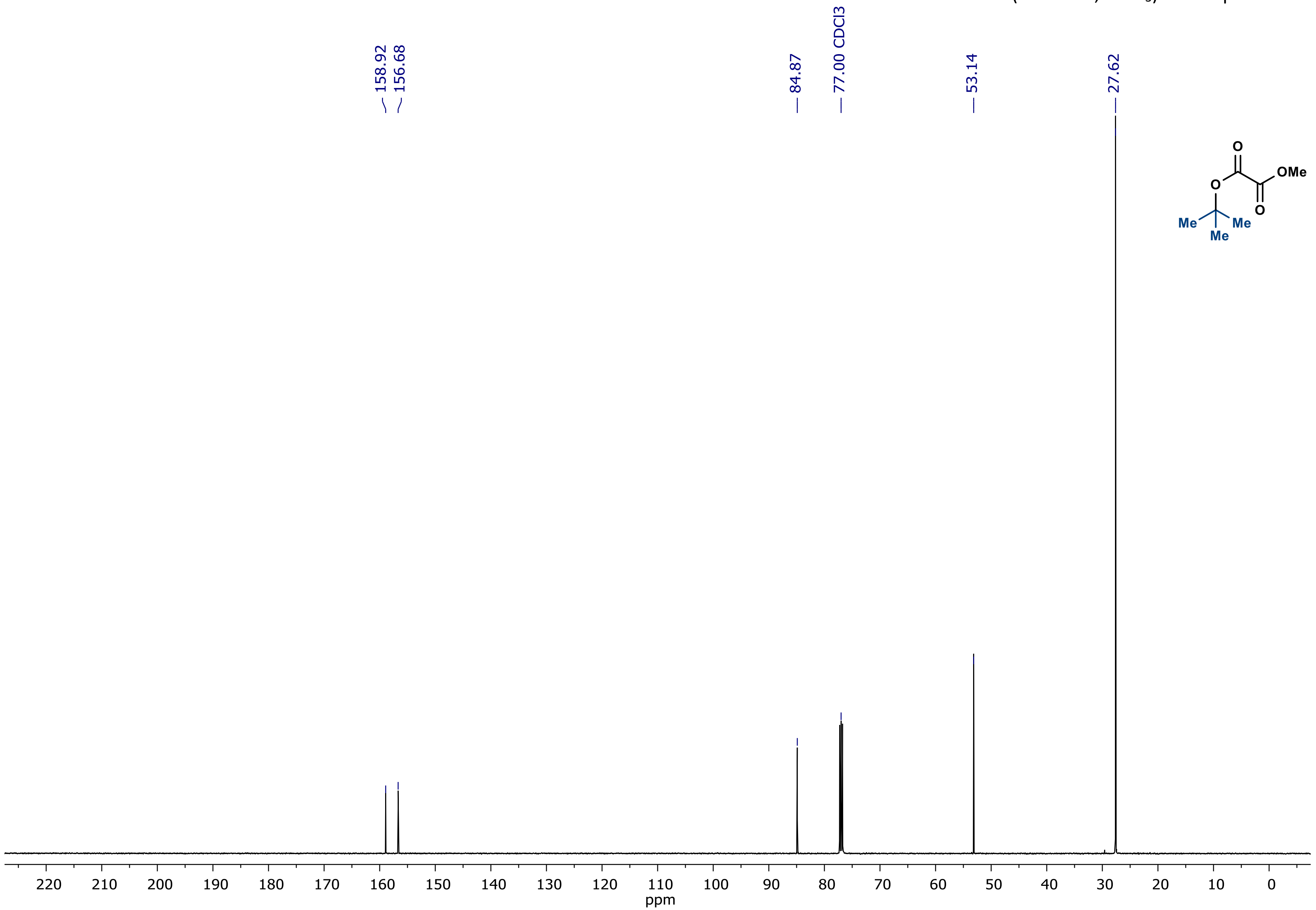

$^{1}$**H NMR** (500 MHz, $CDCl_3$) of compound **2n**

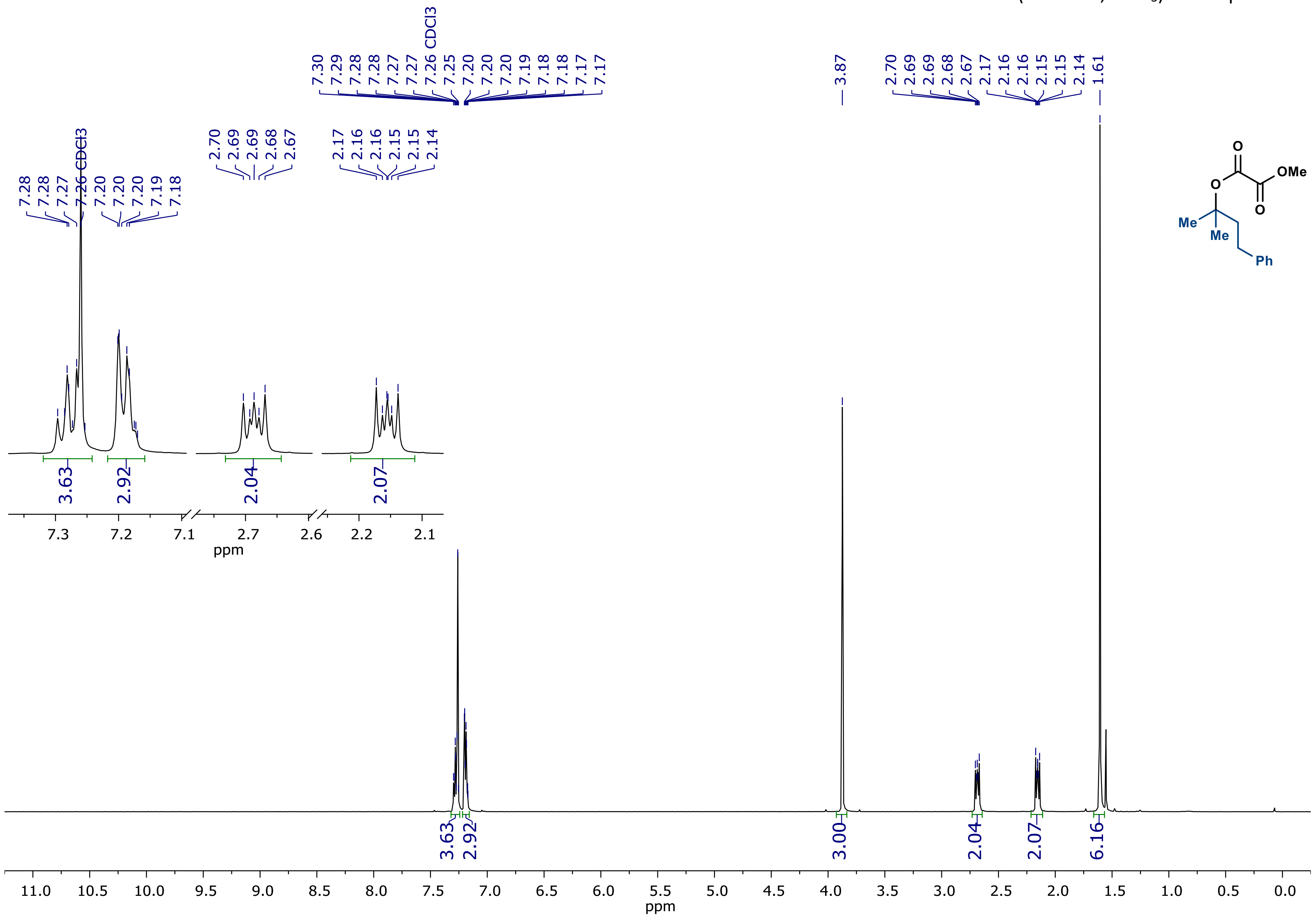

$^{13}$**C NMR** (126 MHz, $CDCl_3$) of compound **2n**

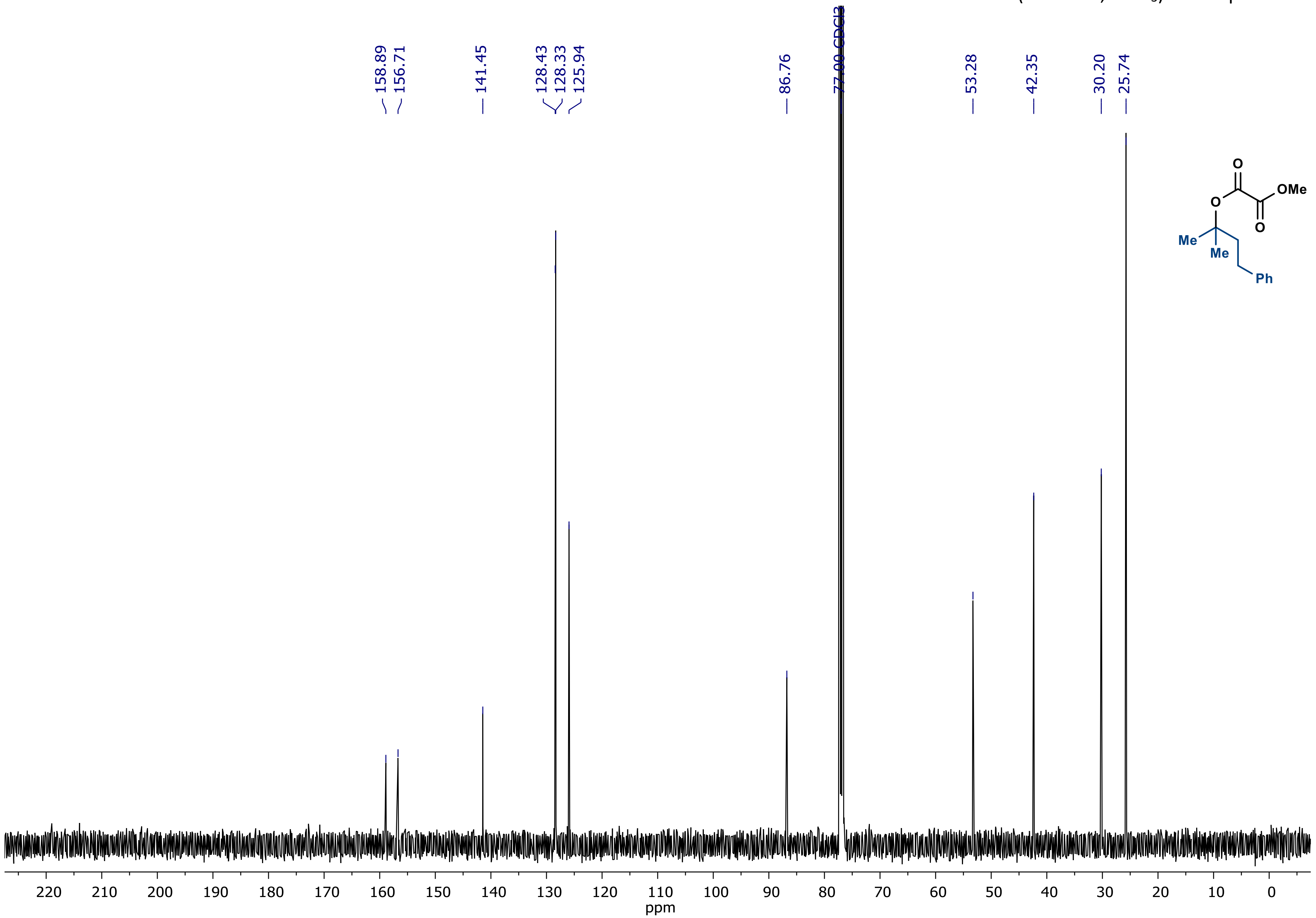

**$^1$H NMR** (500 MHz, $CDCl_3$) of compound **2o**

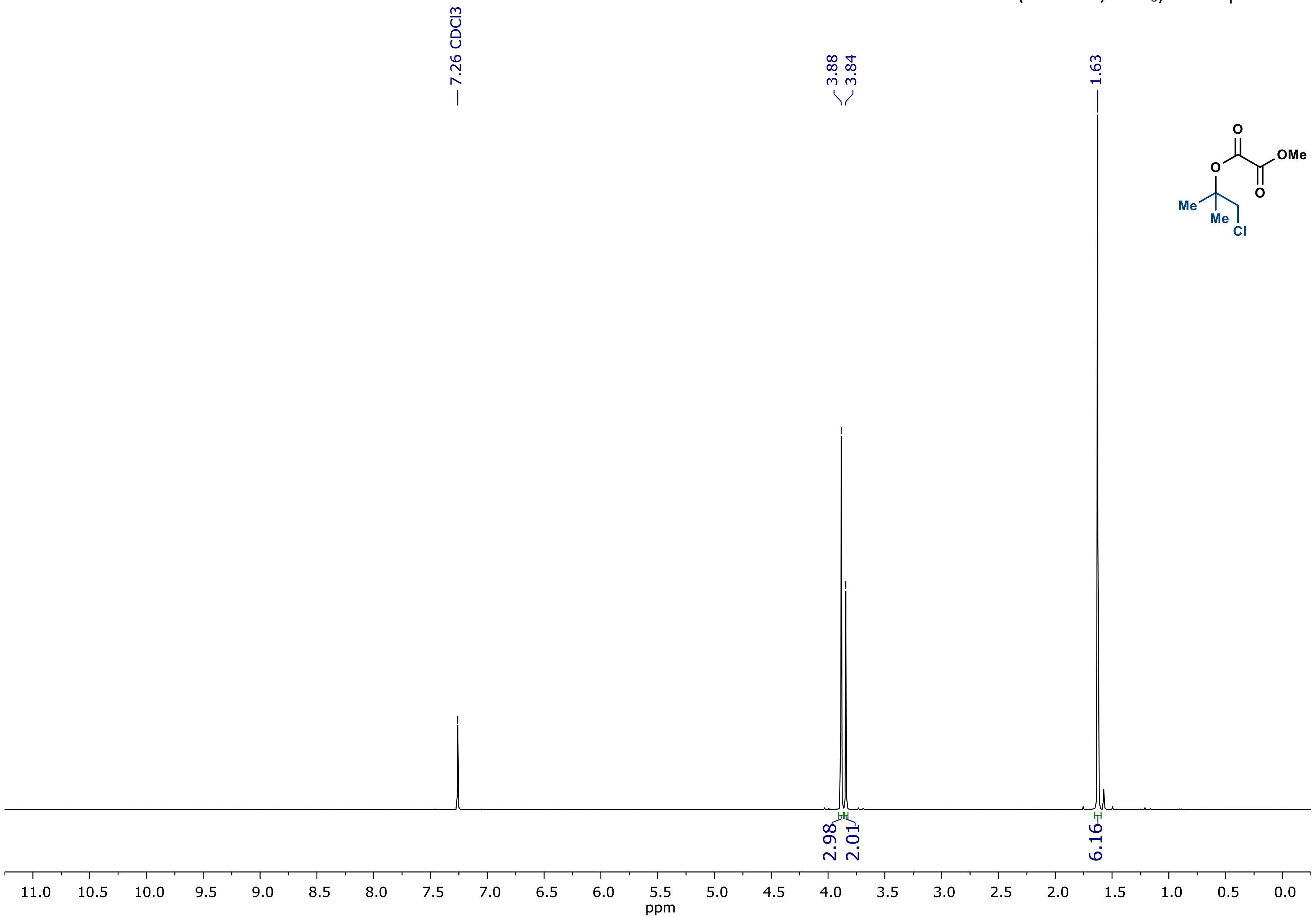

$^{13}$**C NMR** (126 MHz, $CDCl_3$) of compound **2o**

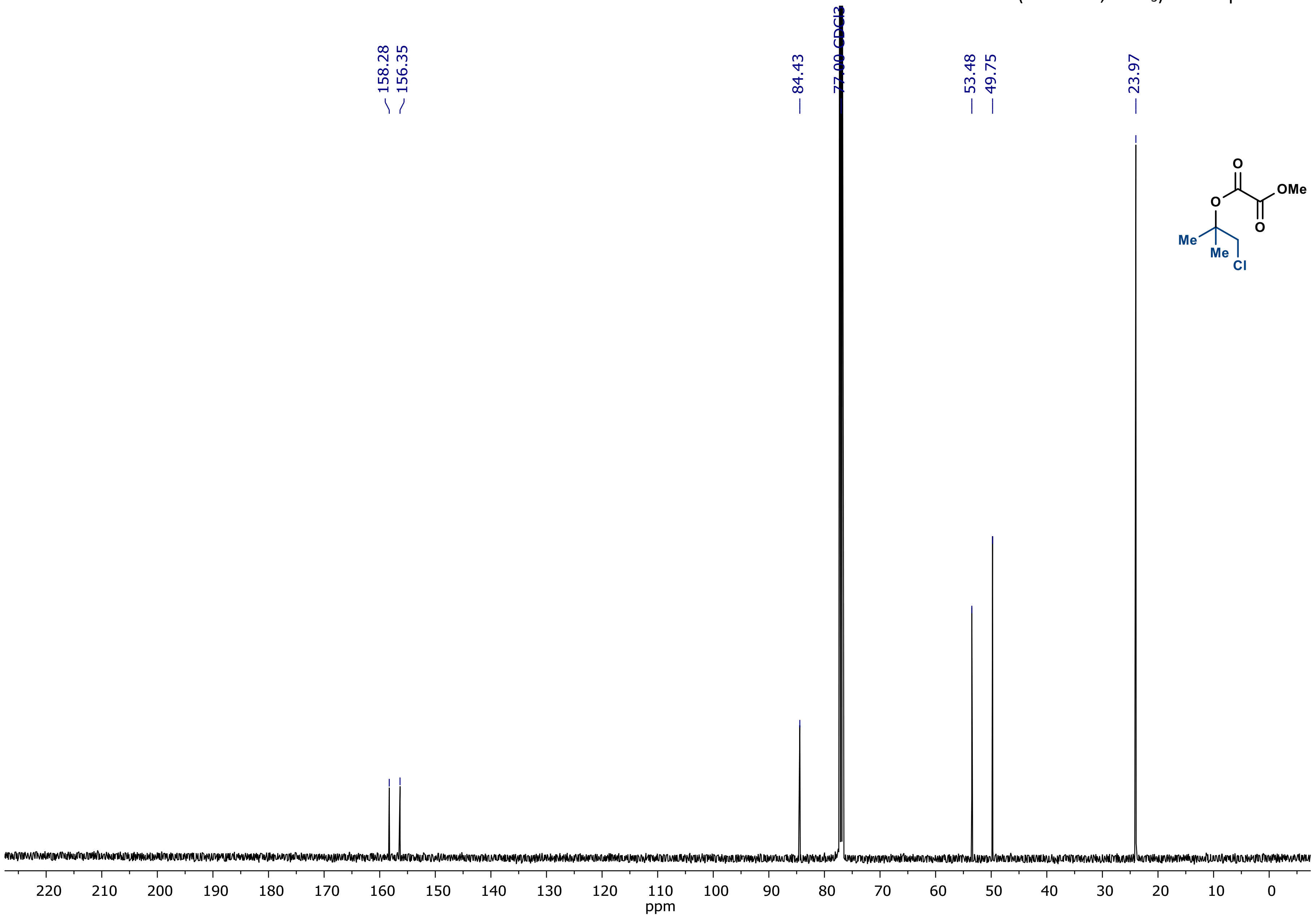

$^{1}$H NMR (500 MHz, CDCl$_3$) of compound **2p**

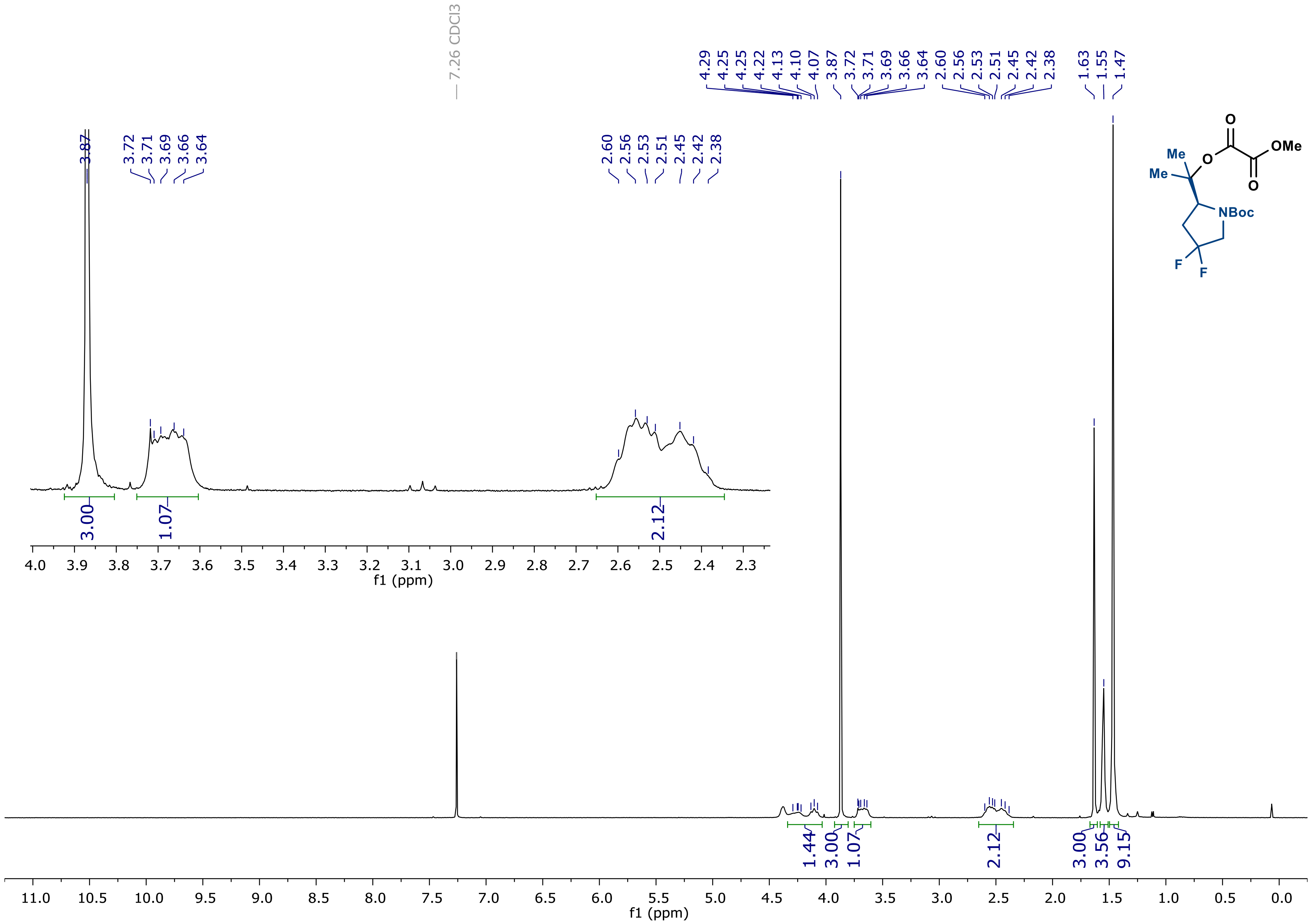

$^{13}$**C NMR** (126 MHz, $CDCl_3$) of compound **2p**

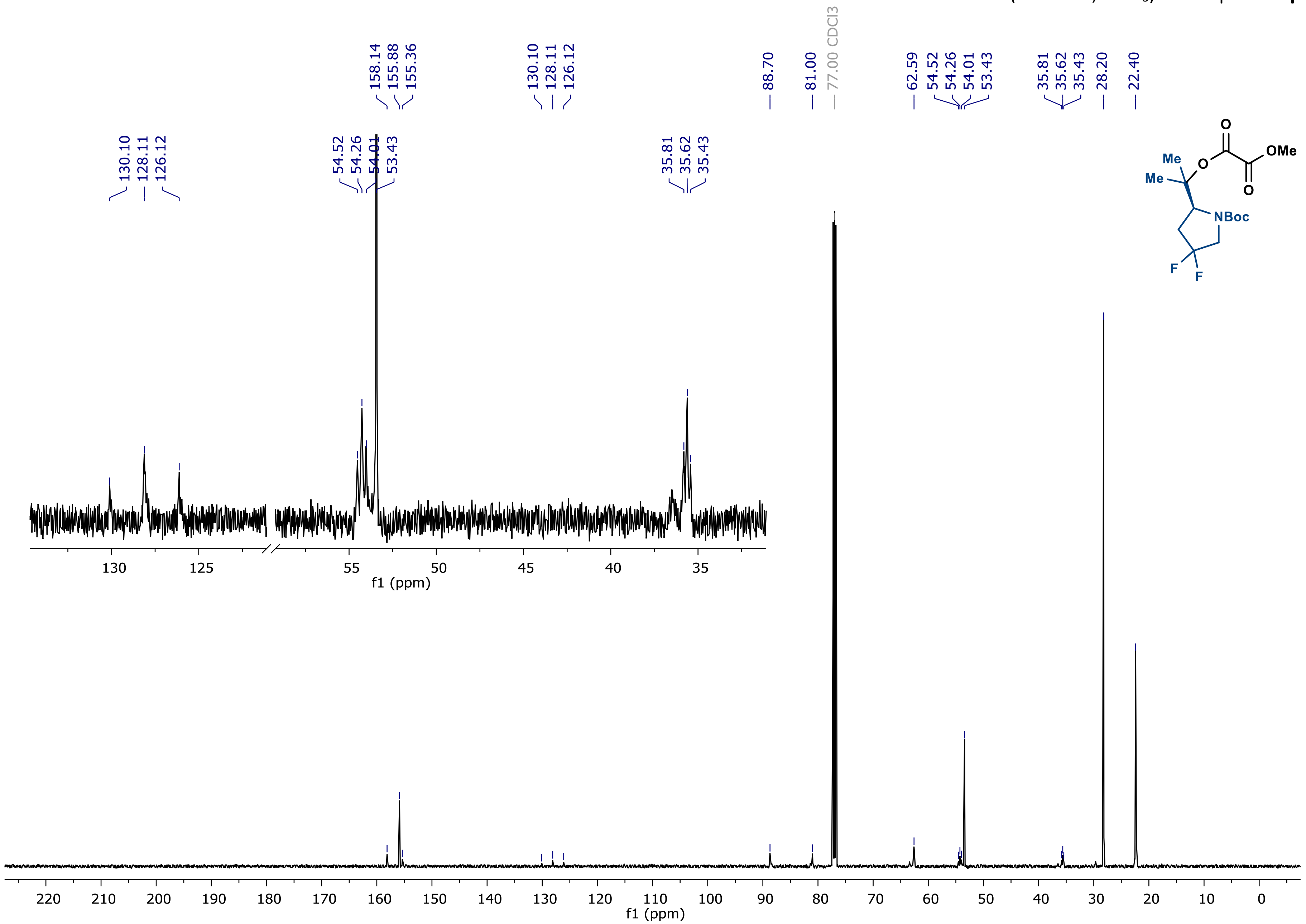

**$^{1}$H NMR** (500 MHz, $CDCl_3$) of compound **2q**

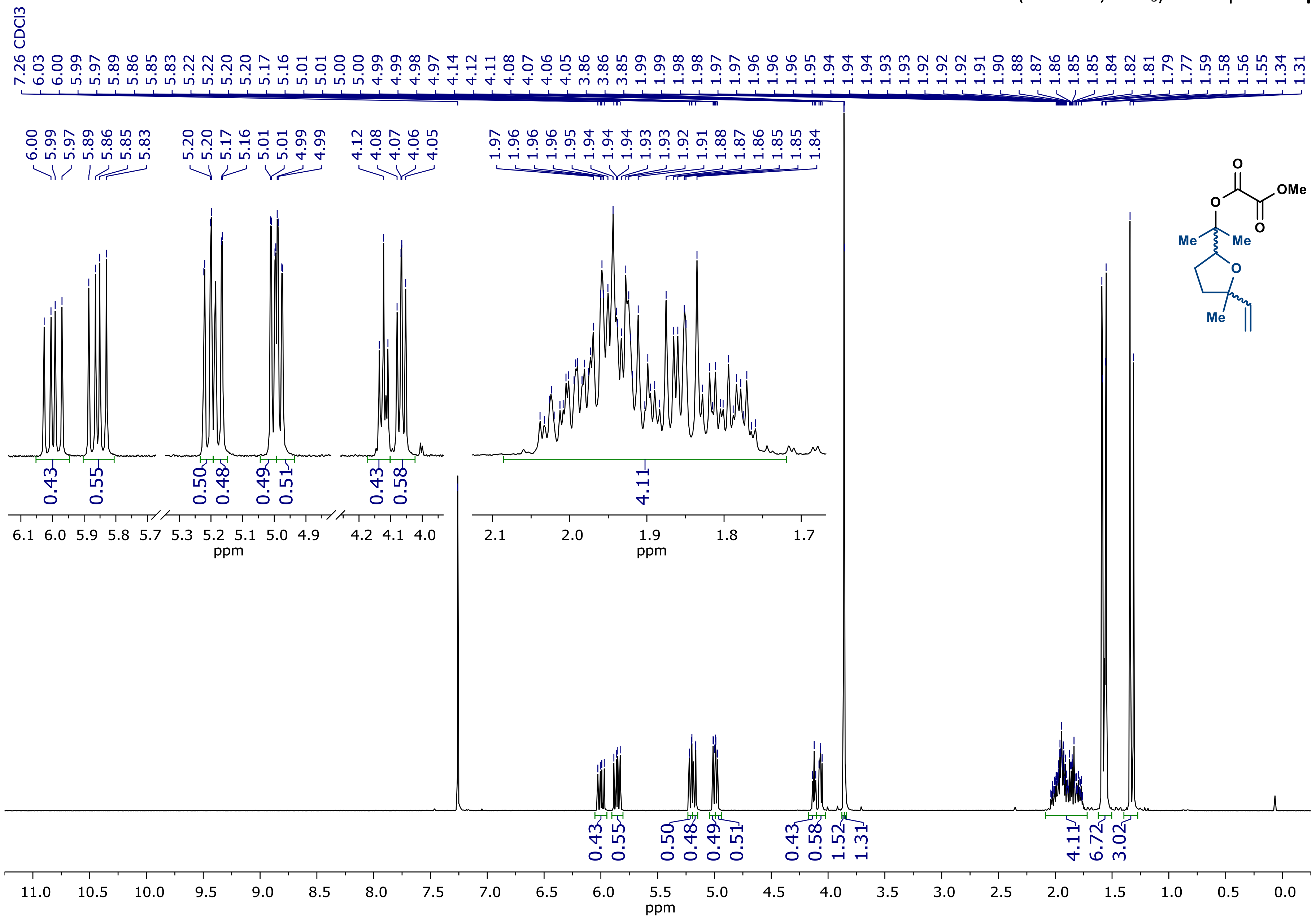

**$^{13}$C NMR** (126 MHz, $CDCl_3$) of compound **2q**

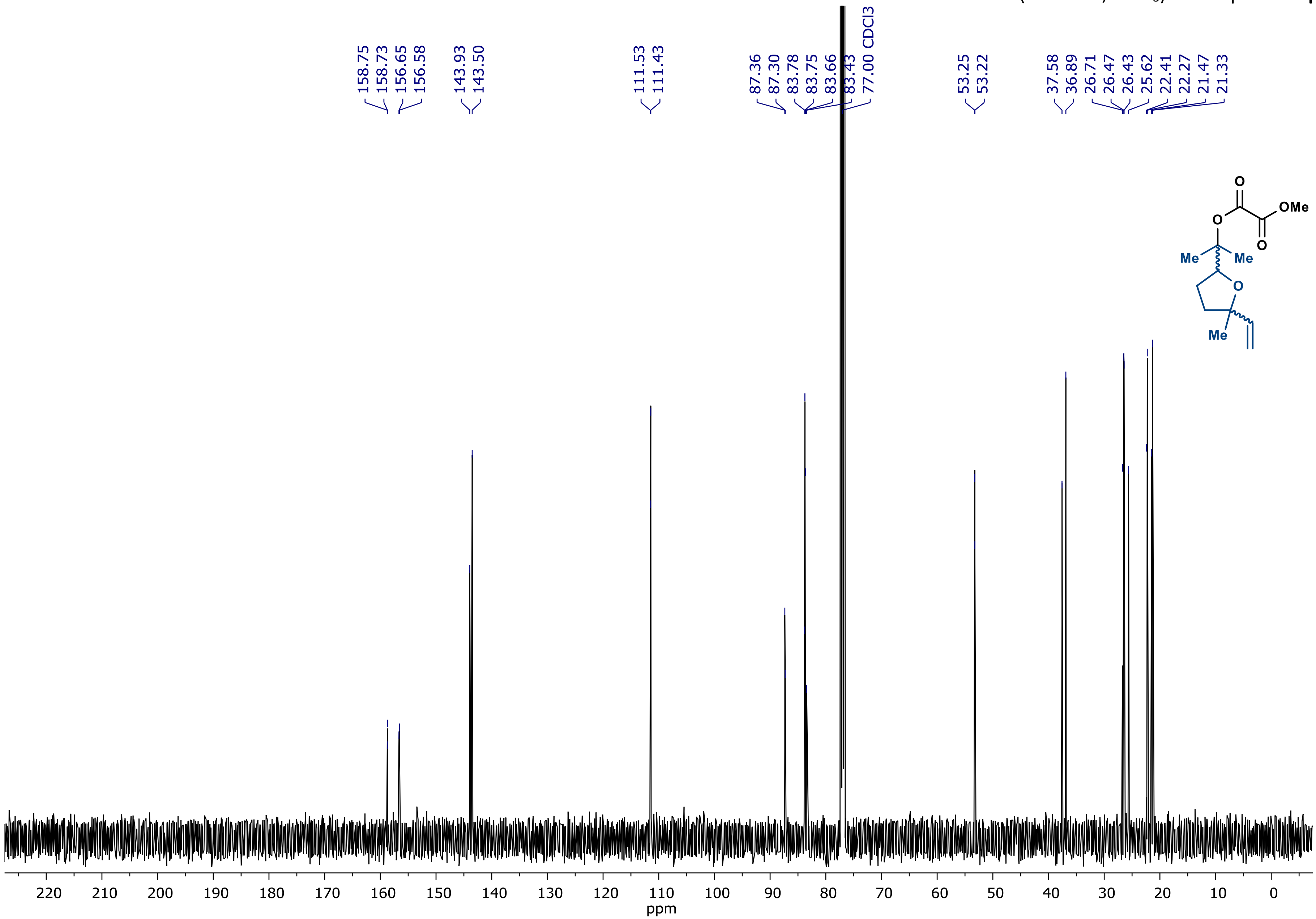

**$^1$H NMR** (500 MHz, $CDCl_3$) of compound **2r**

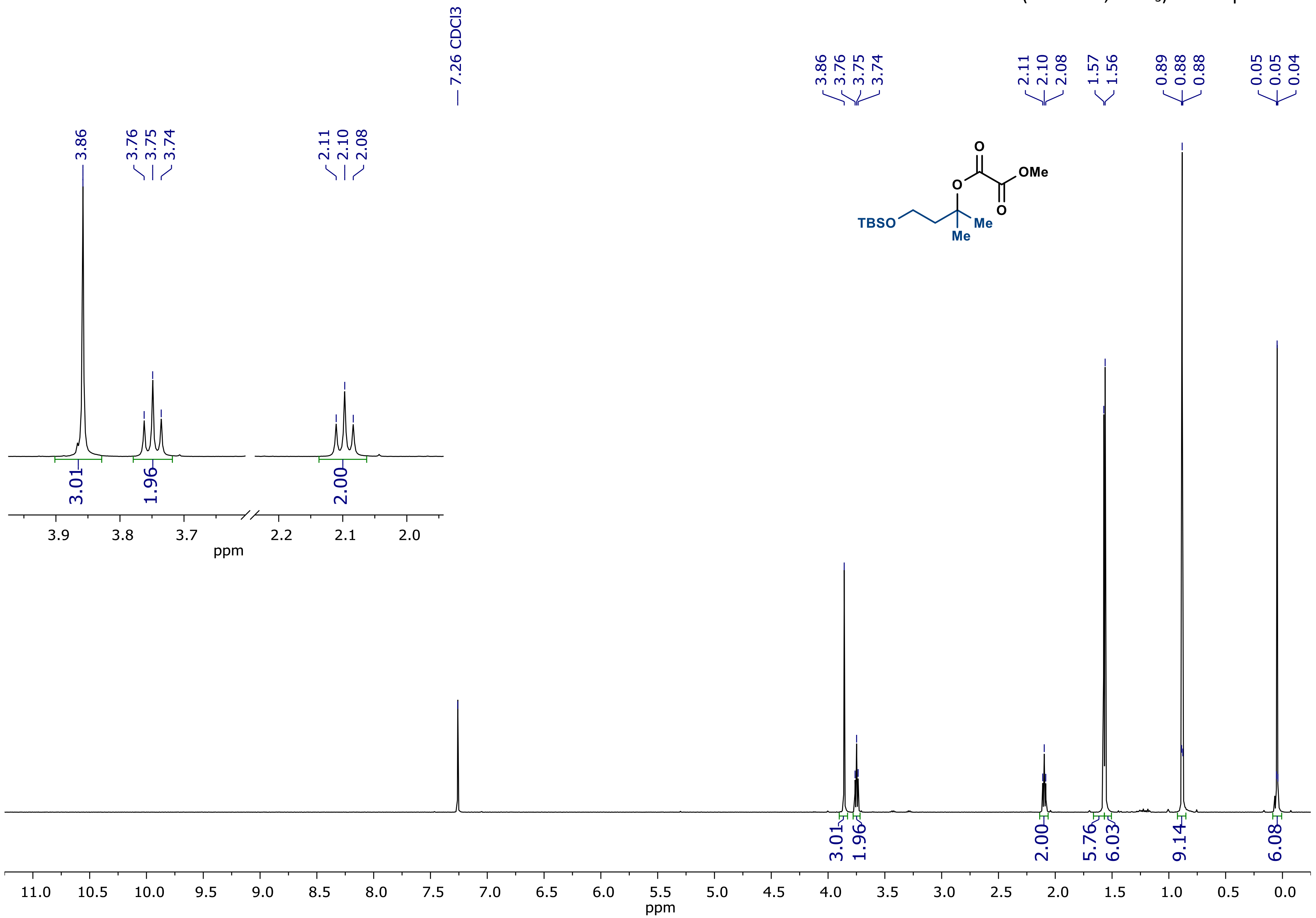

**$^{13}$C NMR** (126 MHz, $CDCl_3$) of compound **2r**

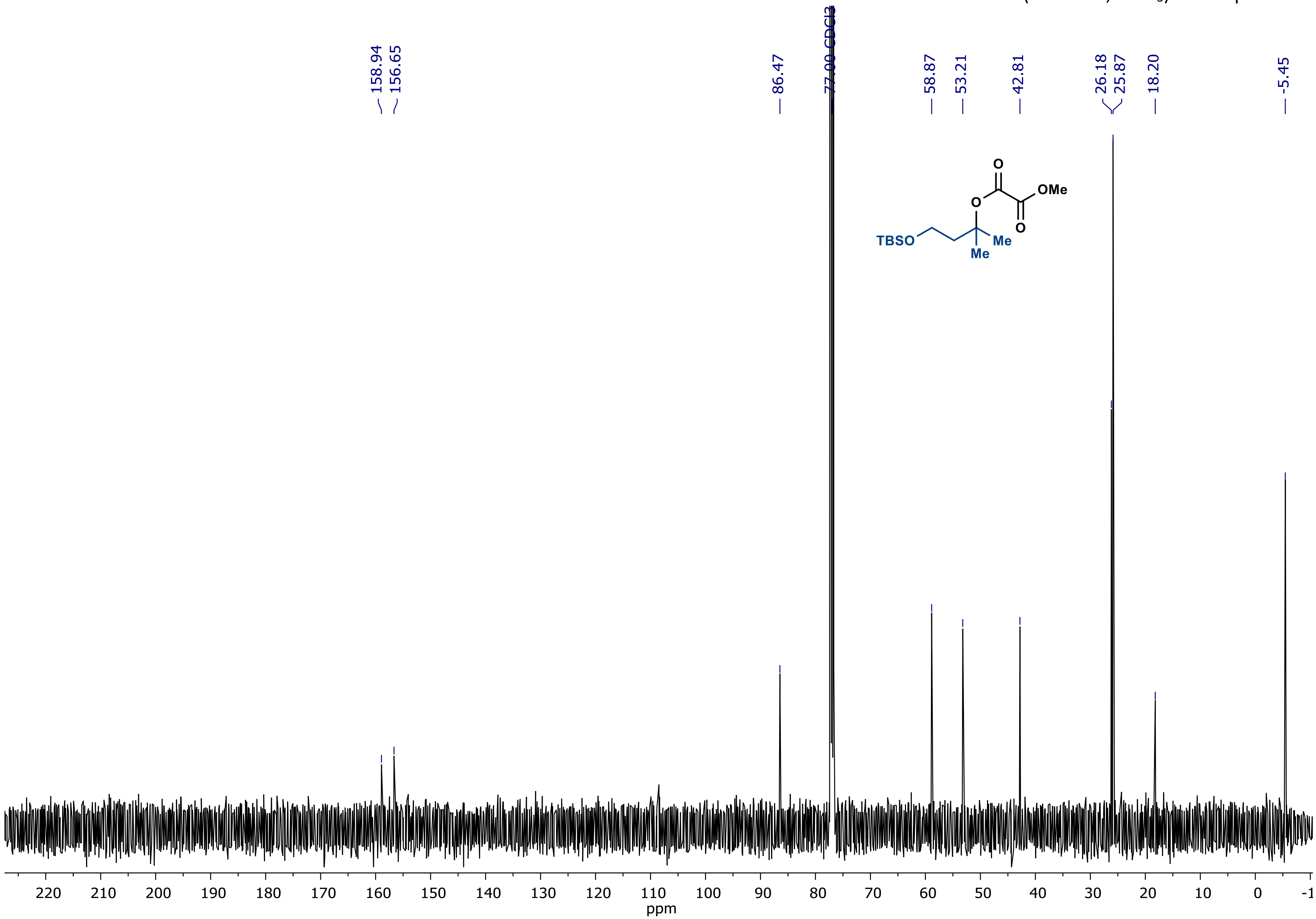

$^{1}$H NMR (500 MHz, $CDCl_3$) of compound **2s**

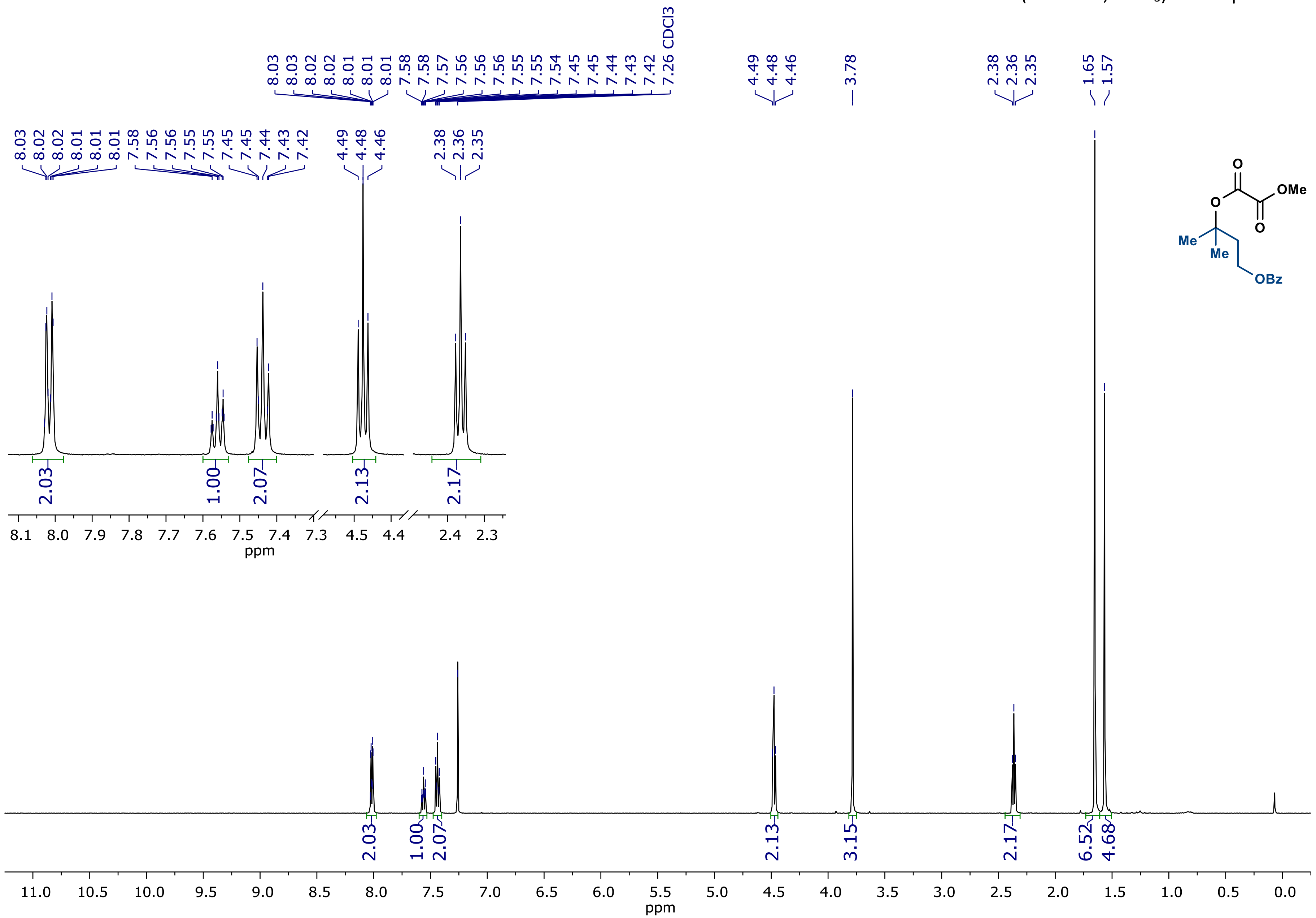

$^{13}$C NMR (126 MHz, $CDCl_3$) of compound **2s**

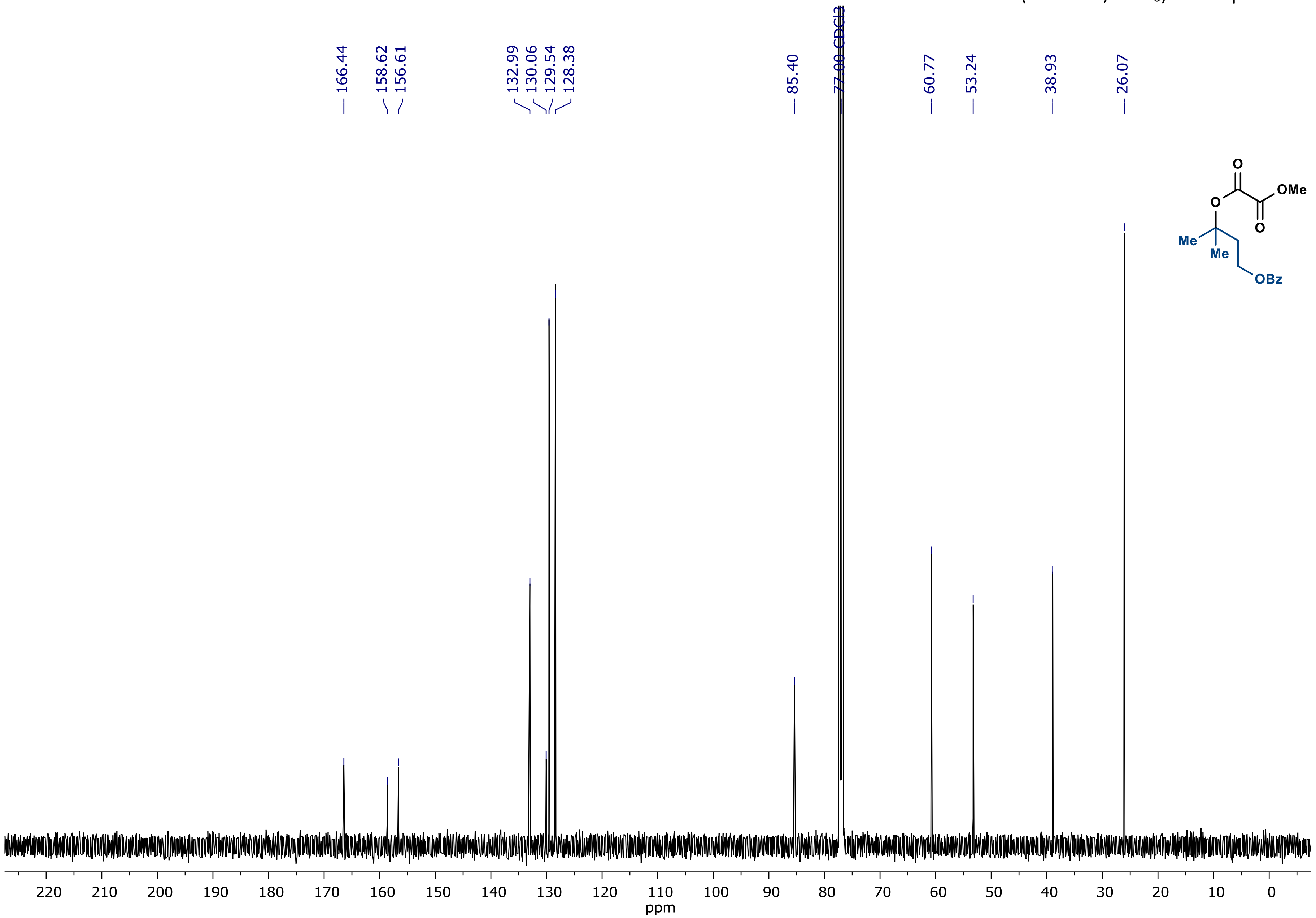

$^{1}$H NMR (500 MHz, $CDCl_3$) of compound **2t**

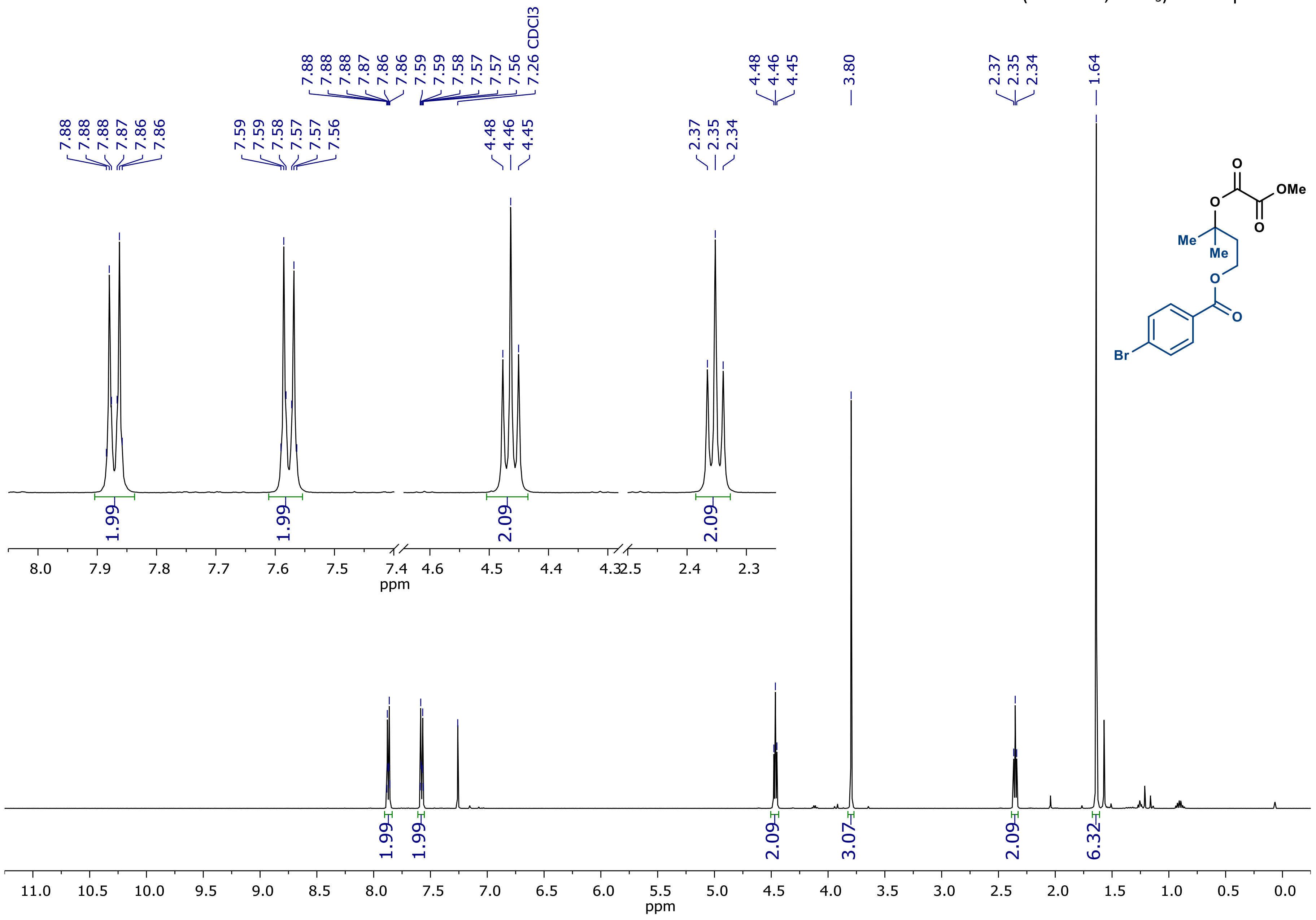

$^{13}$**C NMR** (126 MHz, $CDCl_3$) of compound **2t**

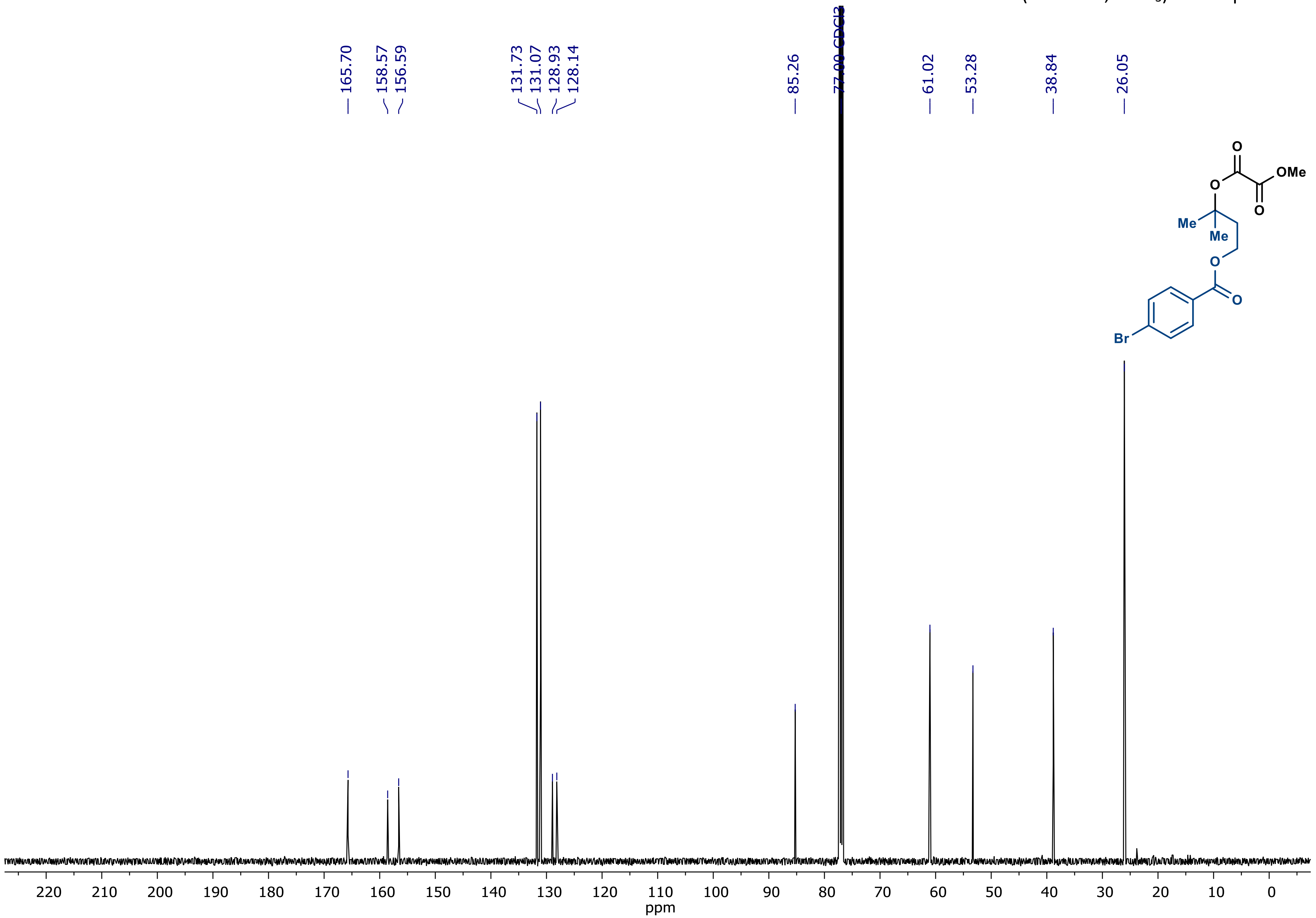

$^{1}$**H NMR** (500 MHz, $CDCl_3$) of compound **2u**

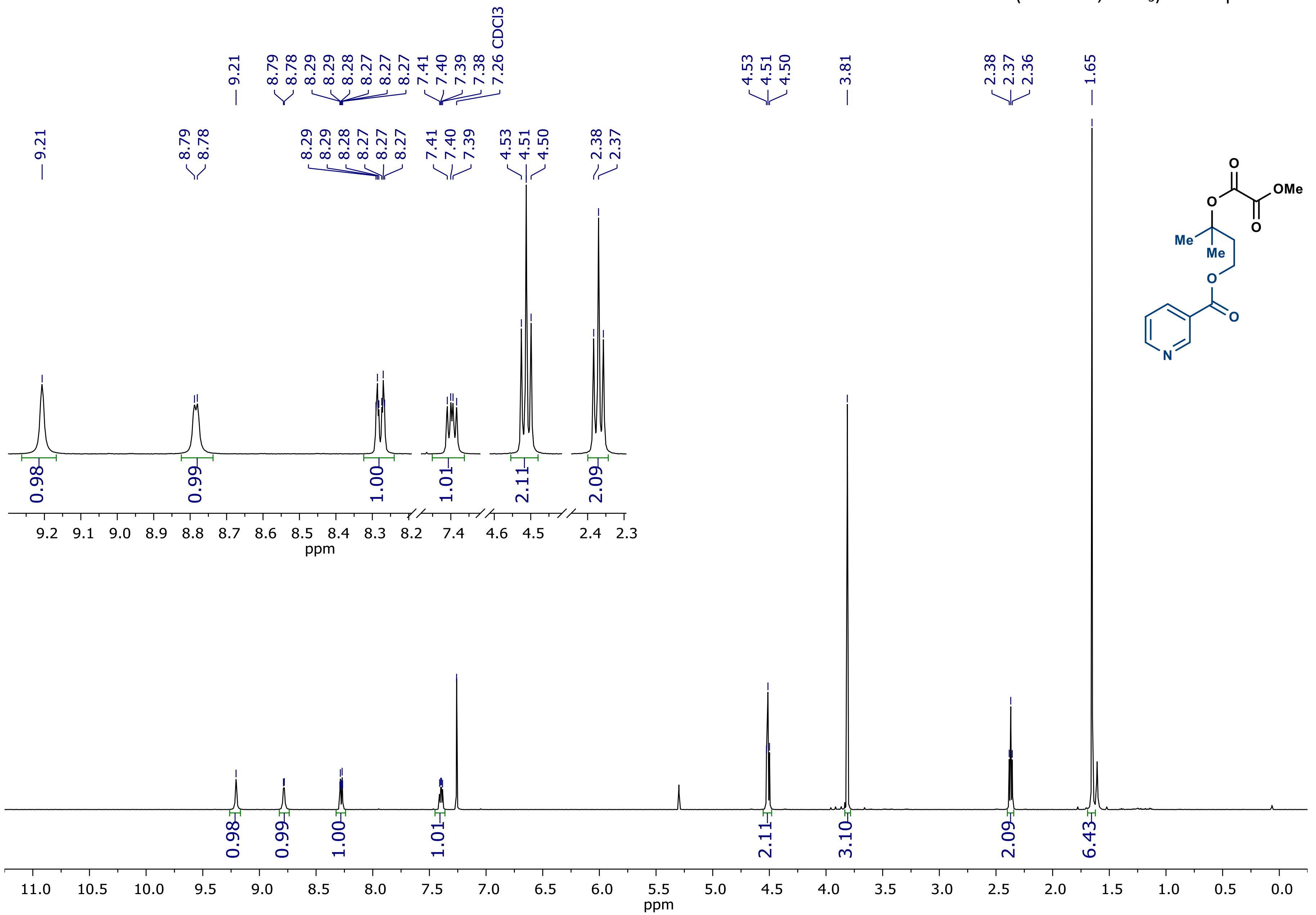

$^{13}$**C NMR** (126 MHz, $CDCl_3$) of compound **2u**

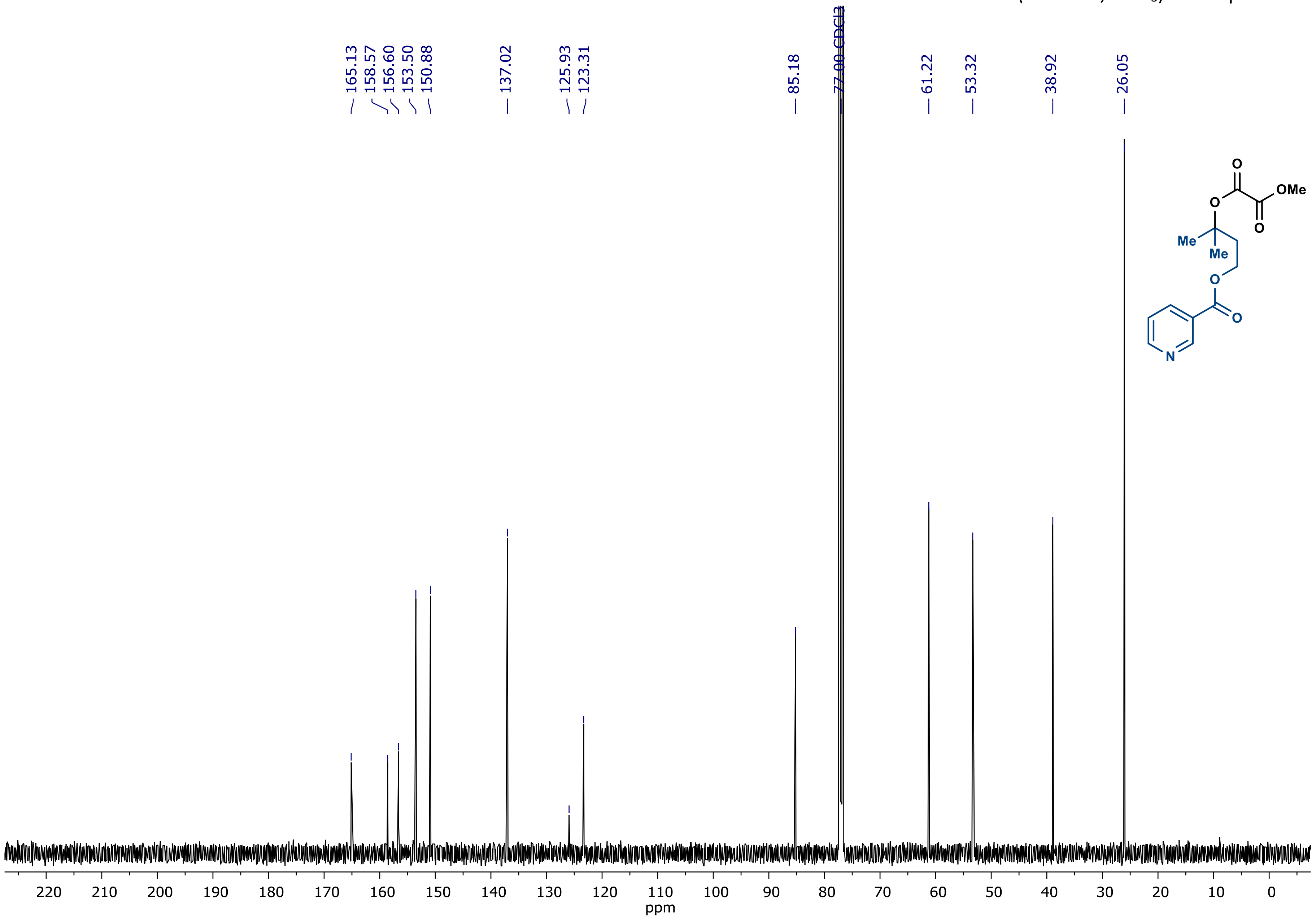

**$^1$H NMR** (500 MHz, $CDCl_3$) of compound **2v**

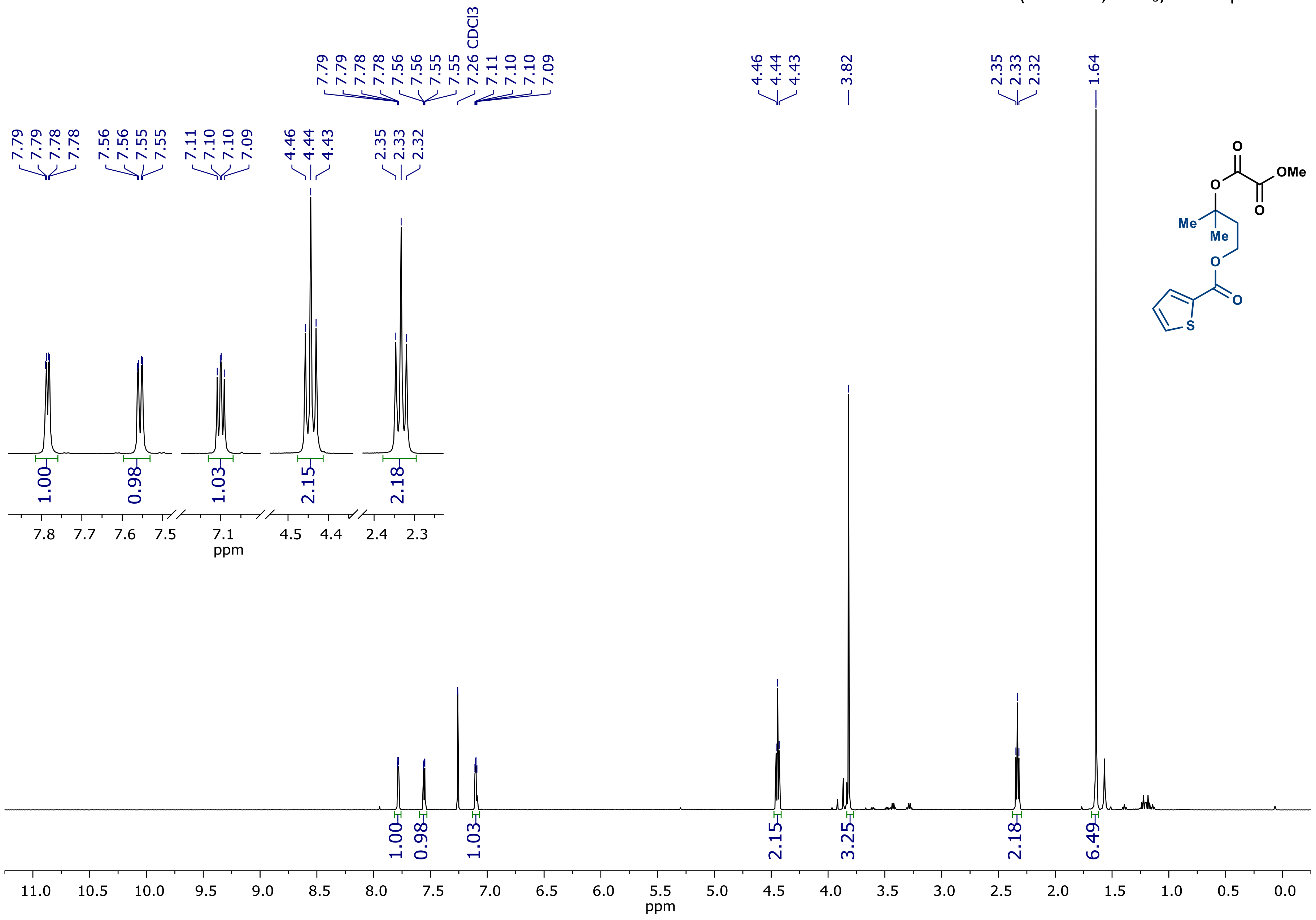

**$^{13}$C NMR** (126 MHz, $CDCl_3$) of compound **2v**

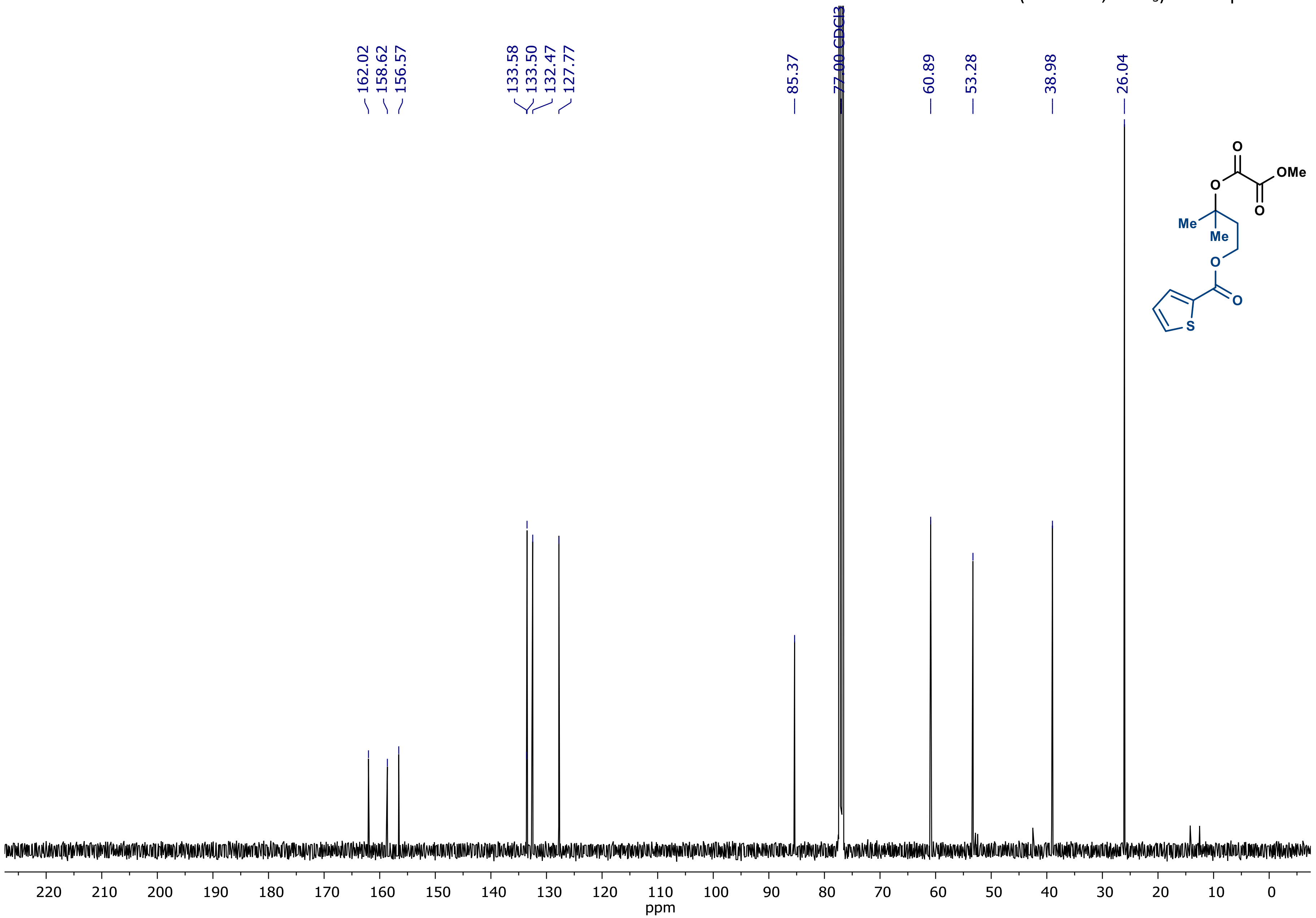

**$^1$H NMR** (500 MHz, $CDCl_3$) of compound **2w**

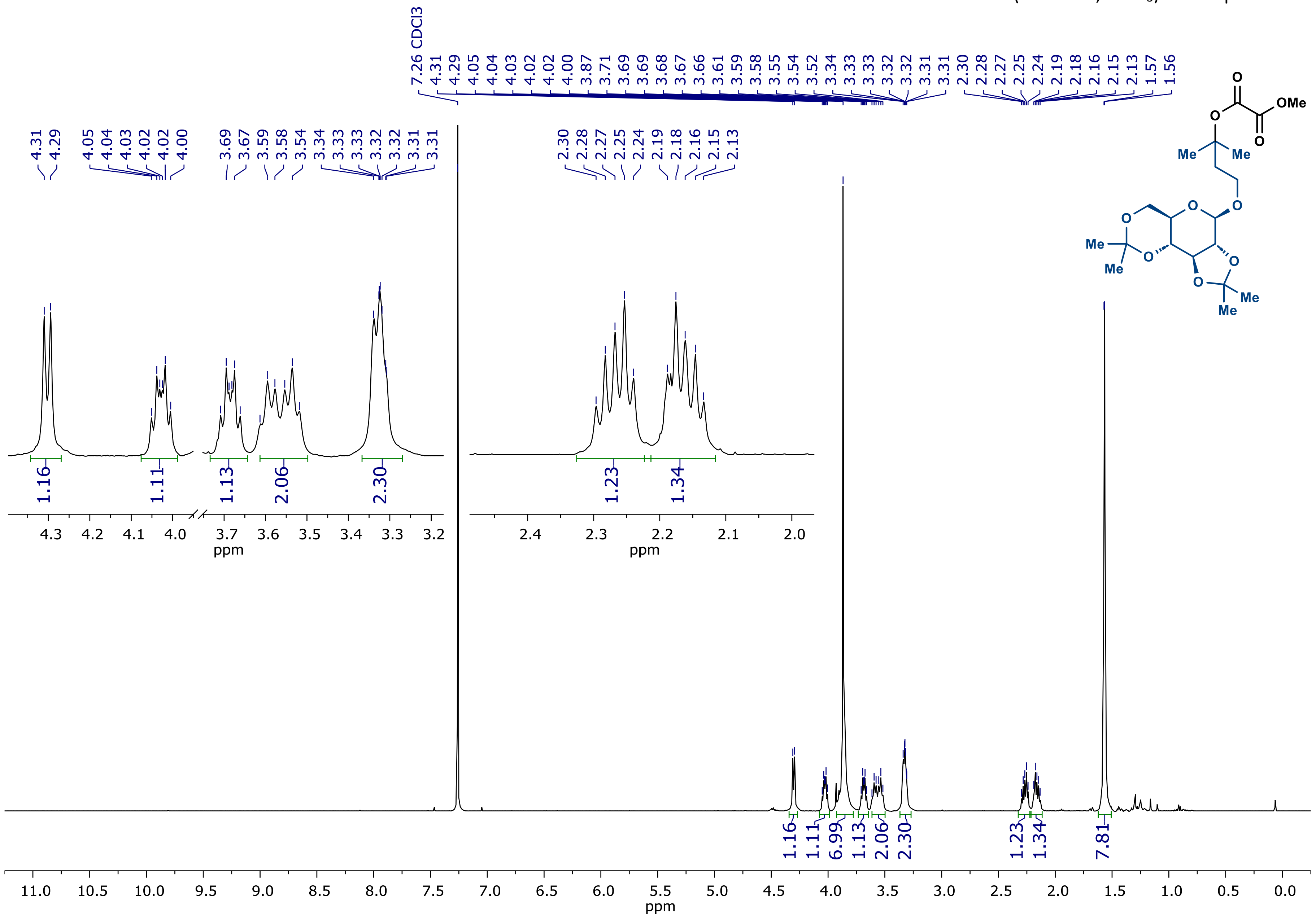

$^{13}$C NMR (126 MHz, CDCl$_3$) of compound **2w**

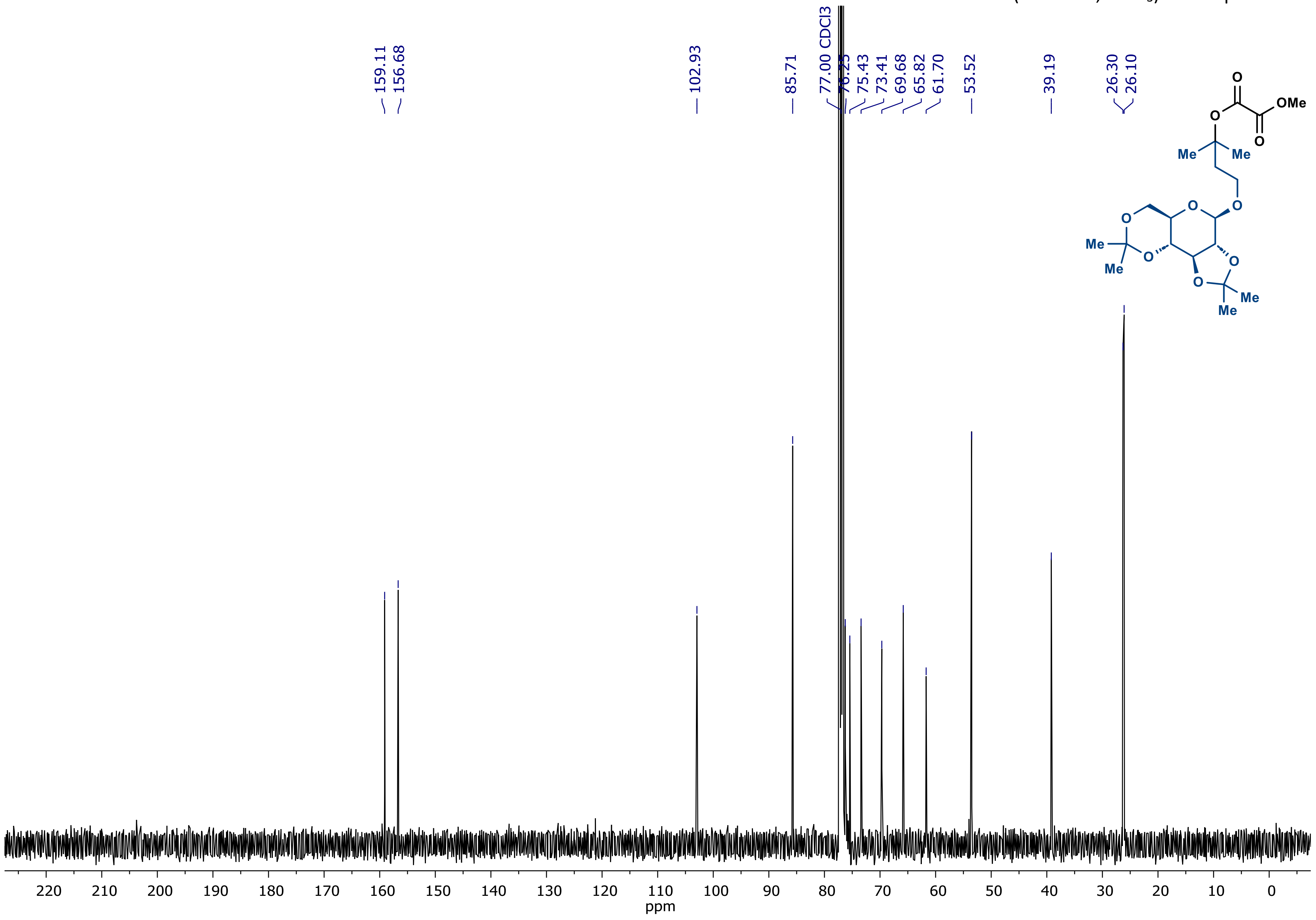

**COSY** of compound **2w**

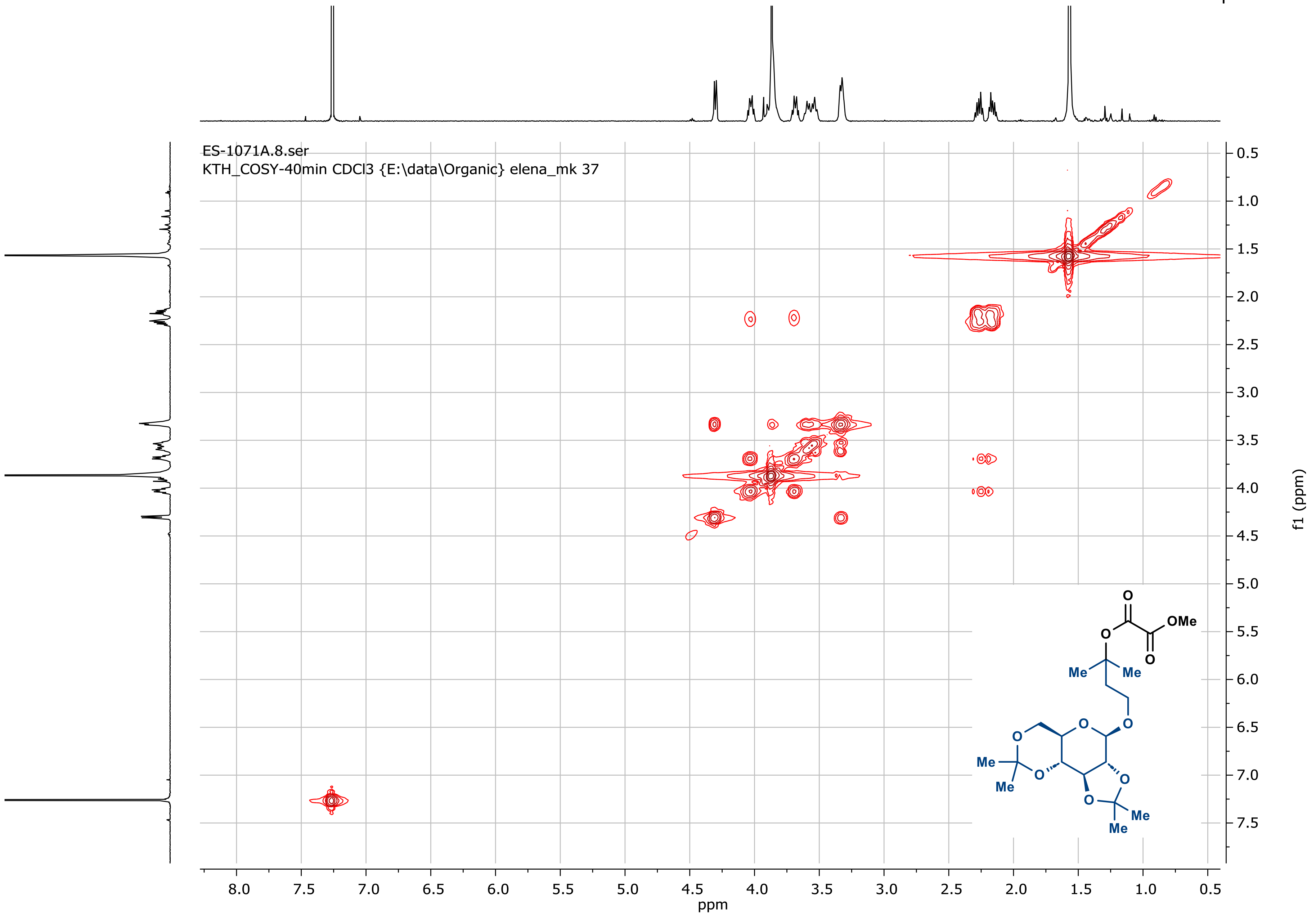

$^{1}$**H NMR** (500 MHz, $CDCl_3$) of compound **2x**

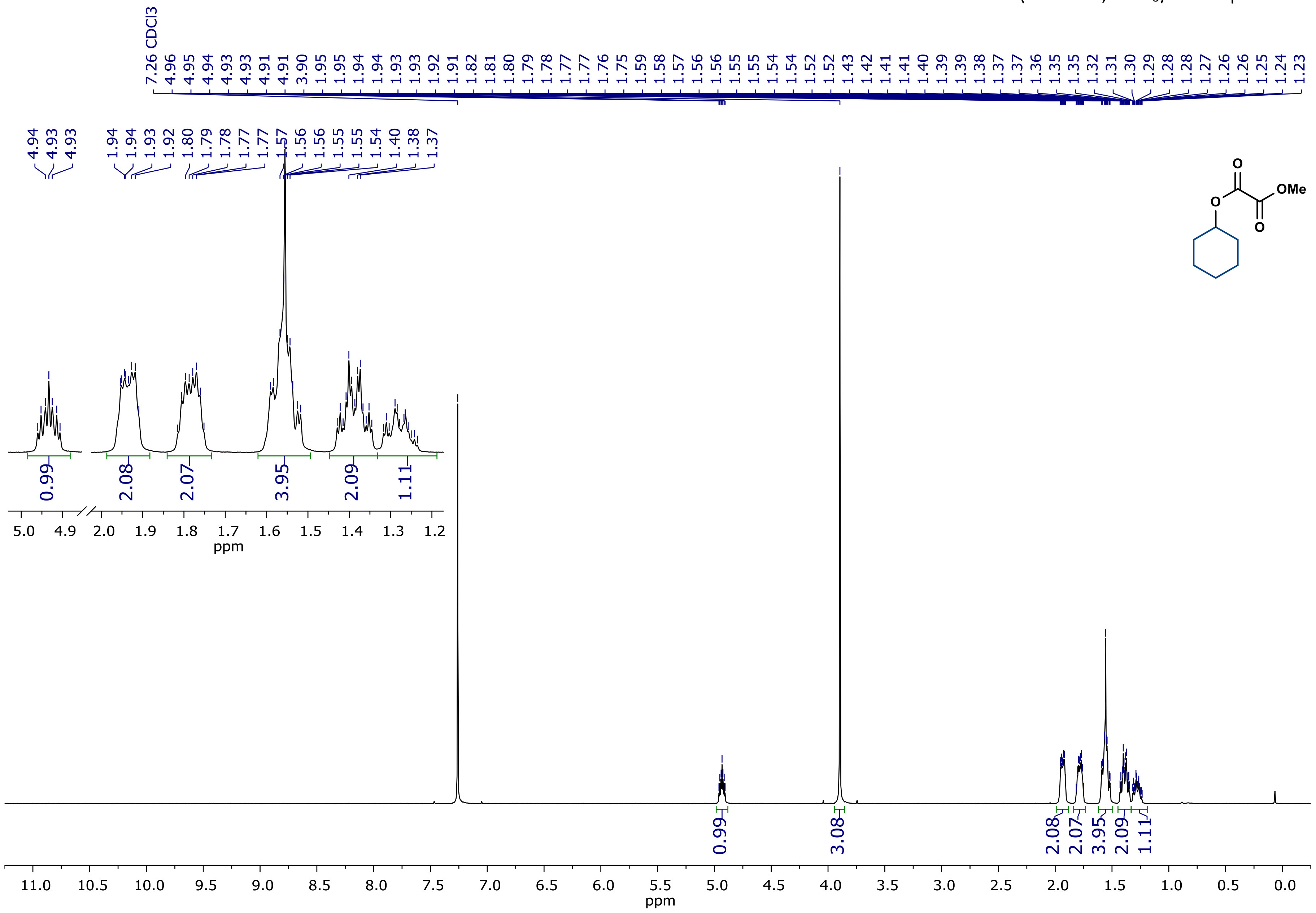

**$^{13}$C NMR** (126 MHz, $CDCl_3$) of compound **2x**

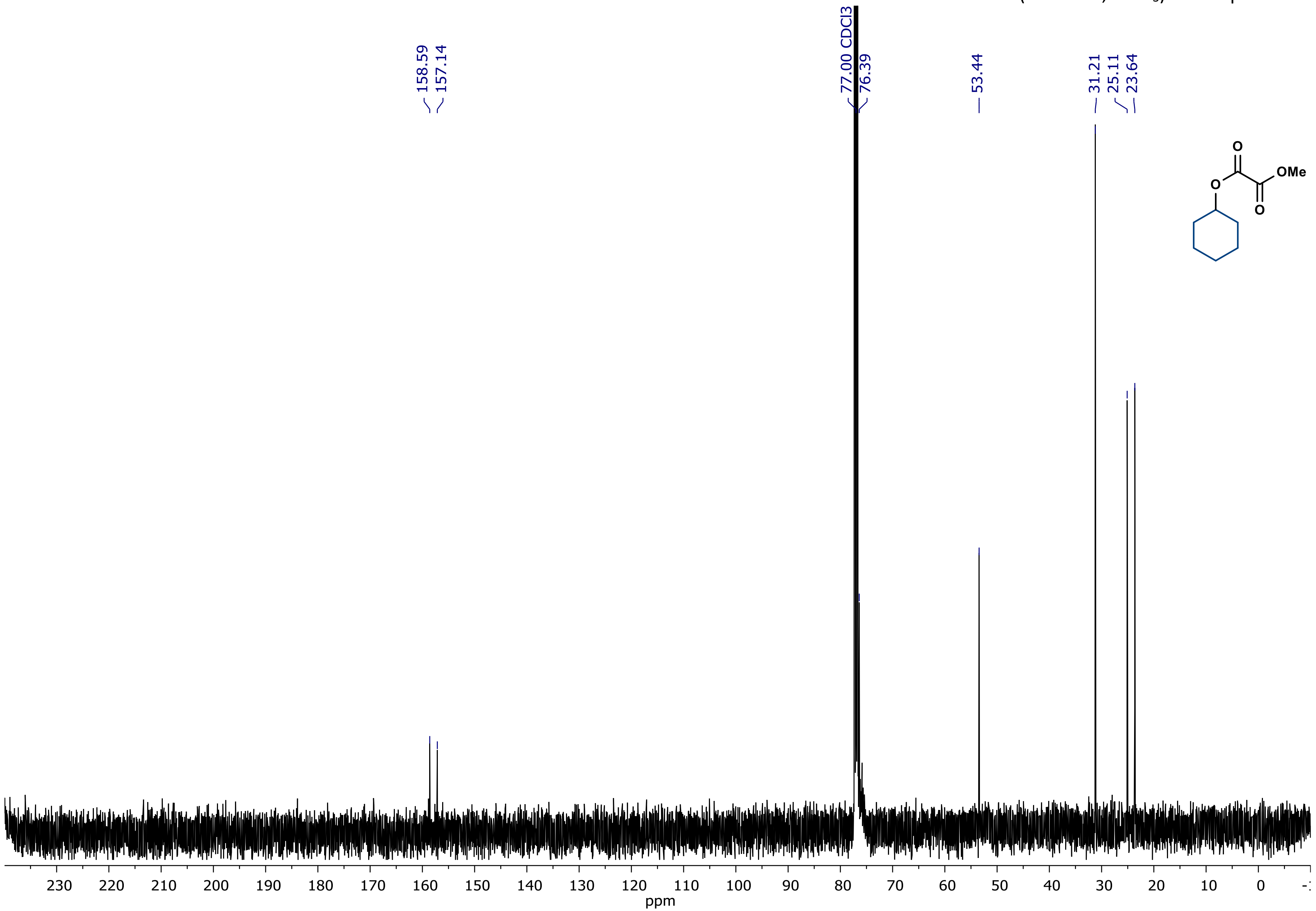

$^{1}$H NMR (500 MHz, $CDCl_3$) of compound **2y**

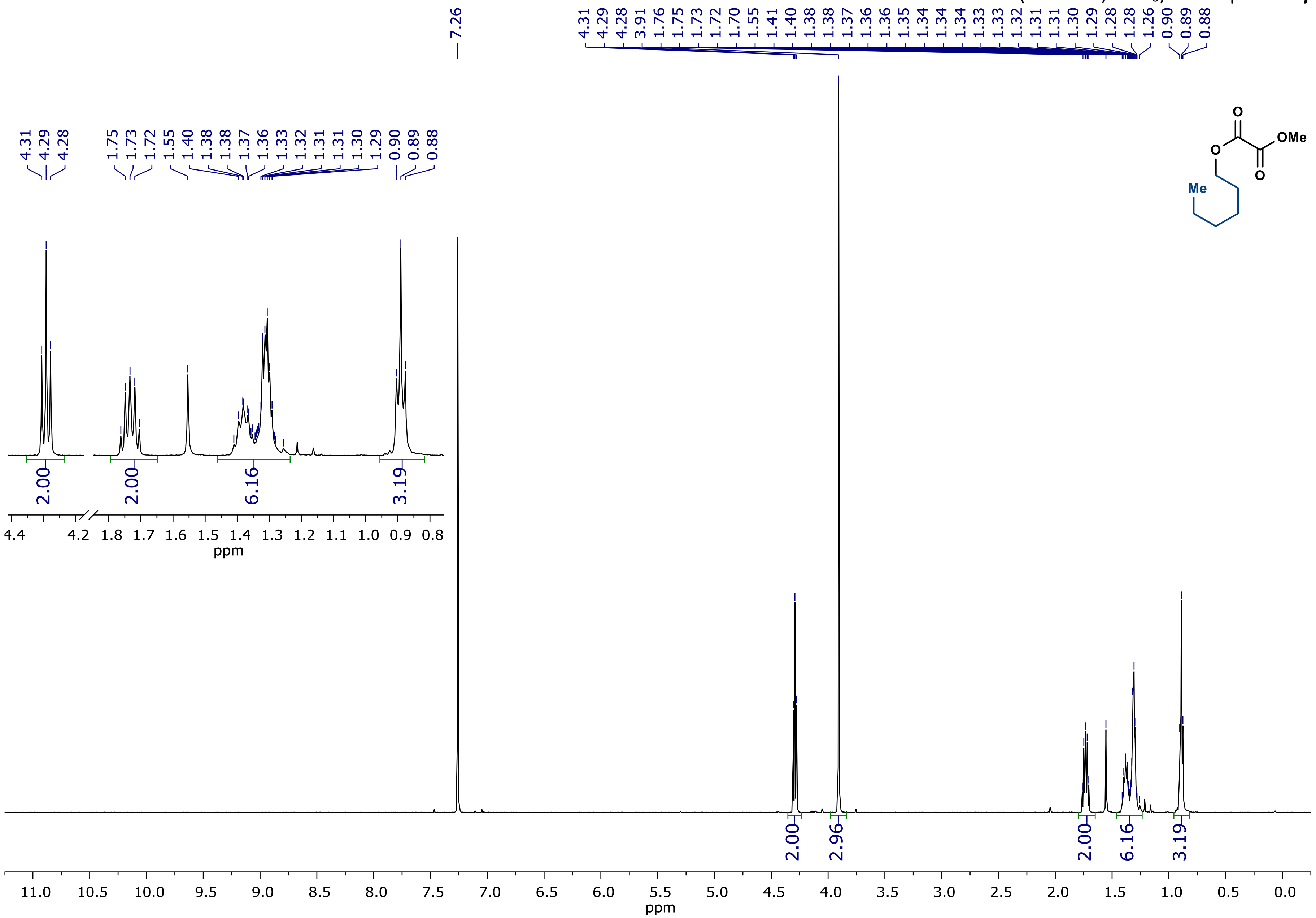

**$^{13}$C NMR** (126 MHz, $CDCl_3$) of compound **2y**

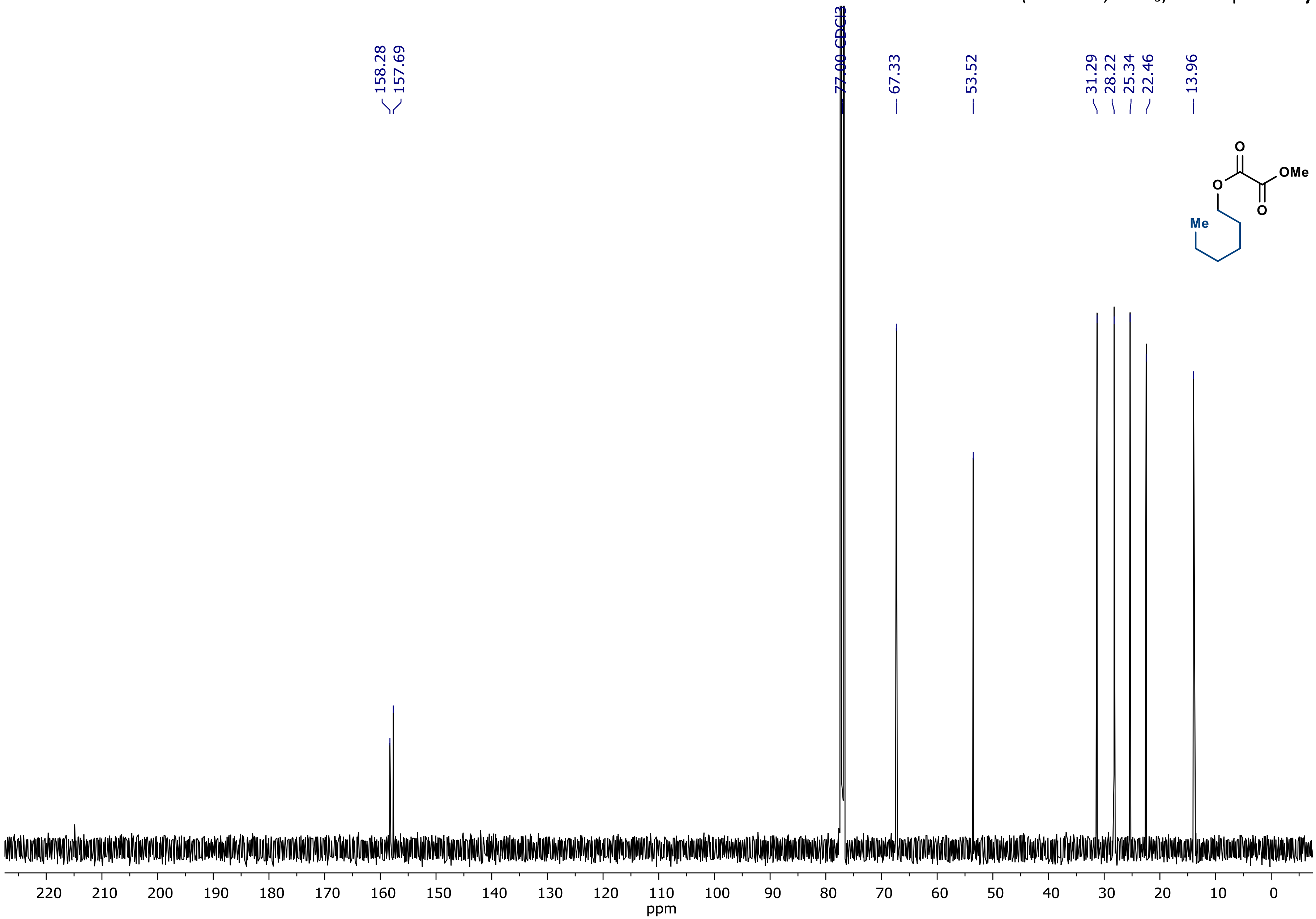

**$^{1}$H NMR** (400 MHz, DMSO) of compound **3a-Na**

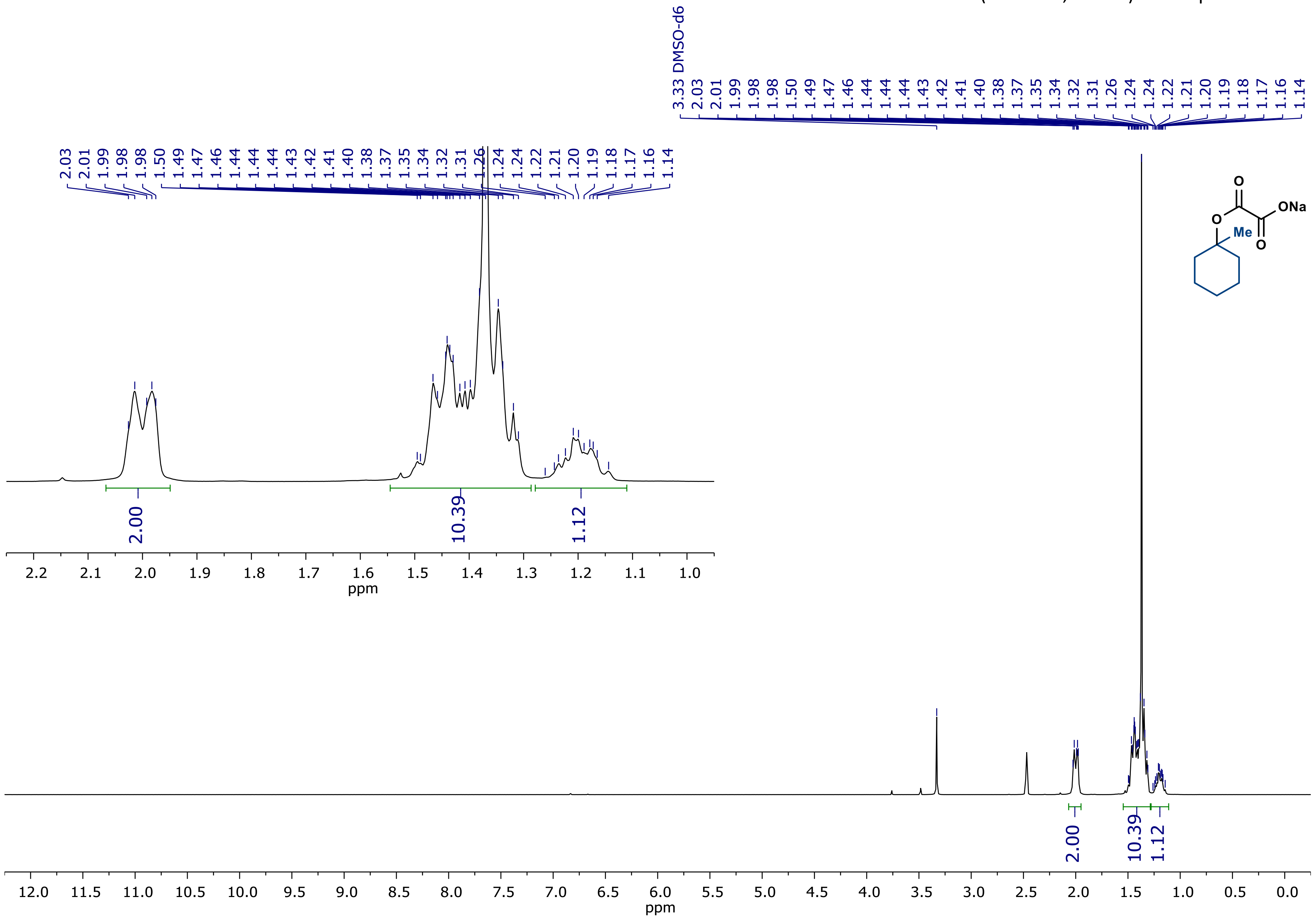

$^{13}$**C NMR** (101 MHz, DMSO) of compound **3a-Na**

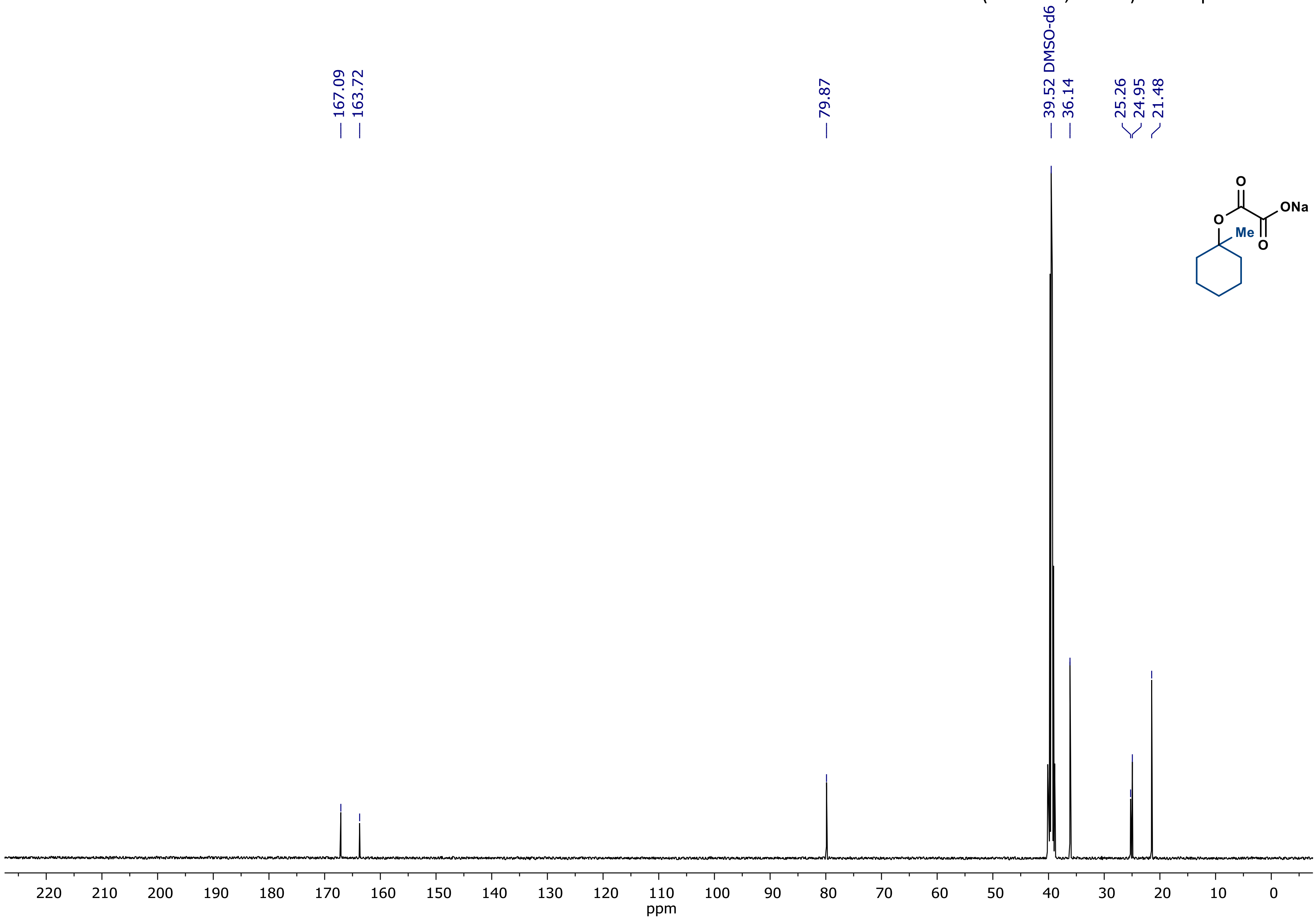

$^1$**H NMR** (500 MHz, DMSO) of compound **3x-Na**

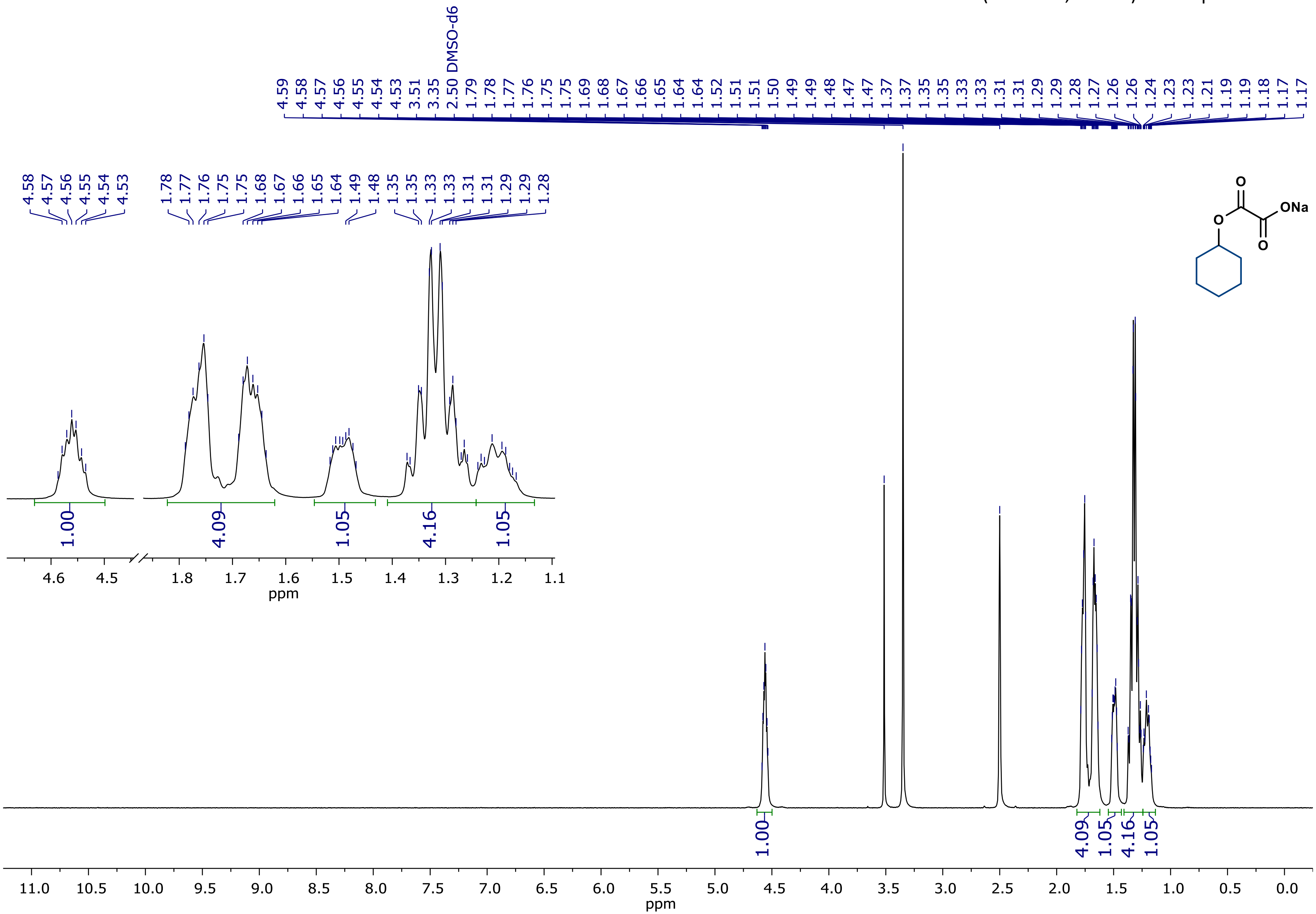

$^{13}$C NMR (126 MHz, DMSO) of compound **3x-Na**

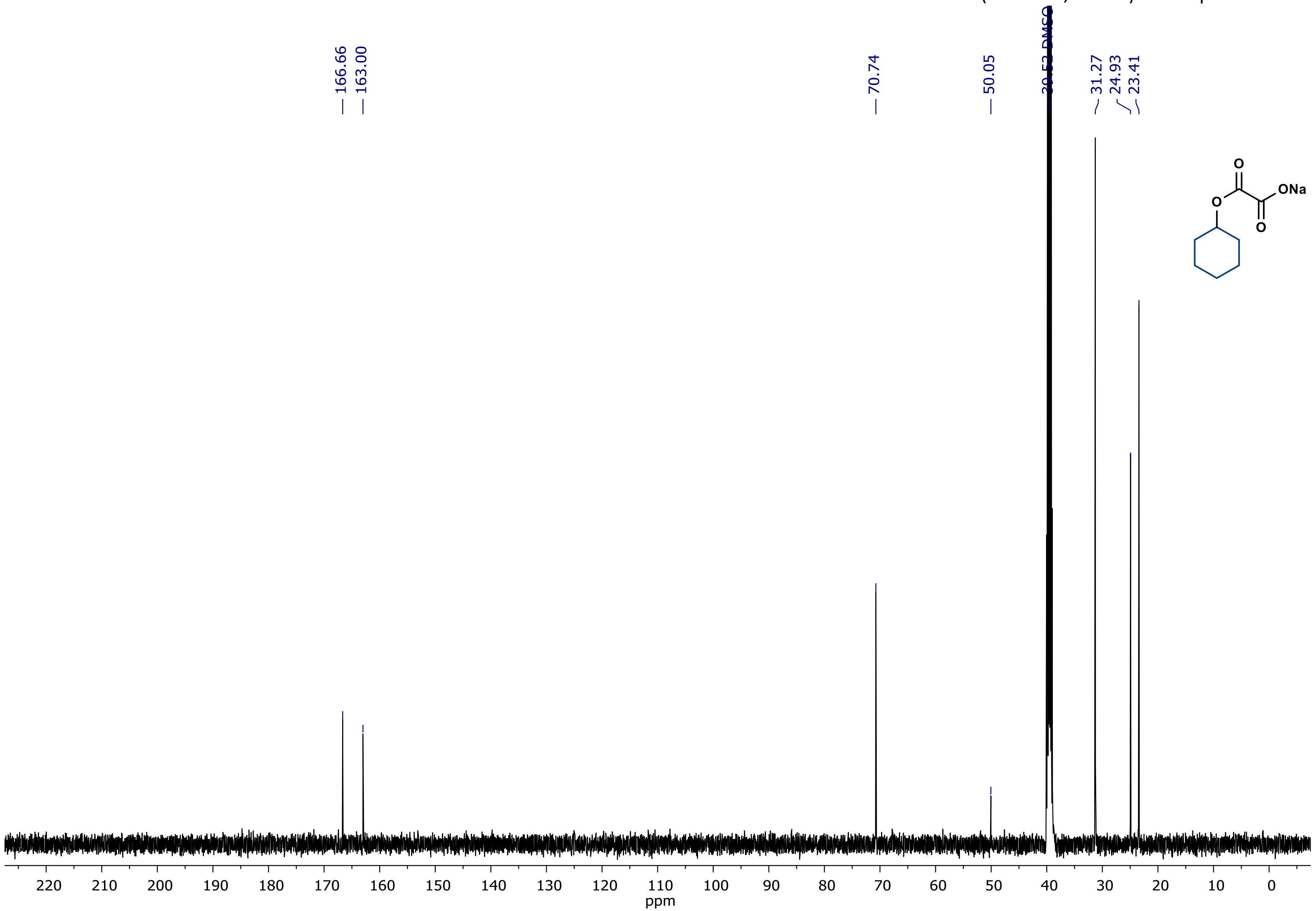

**$^1$H NMR** (500 MHz, DMSO) of compound **3y-Na**

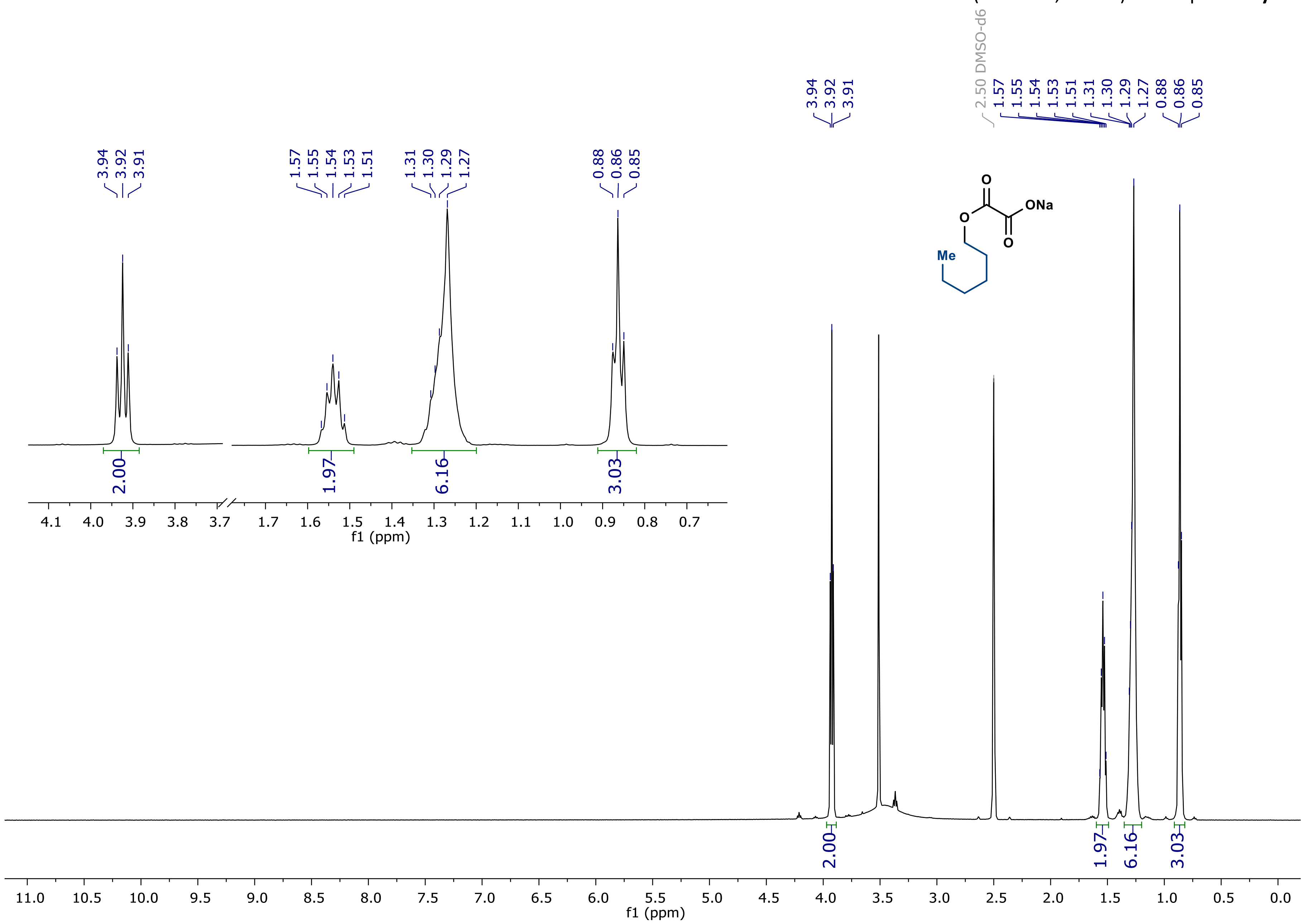

$^{13}$**C NMR** (126 MHz, DMSO) of compound **3y-Na**

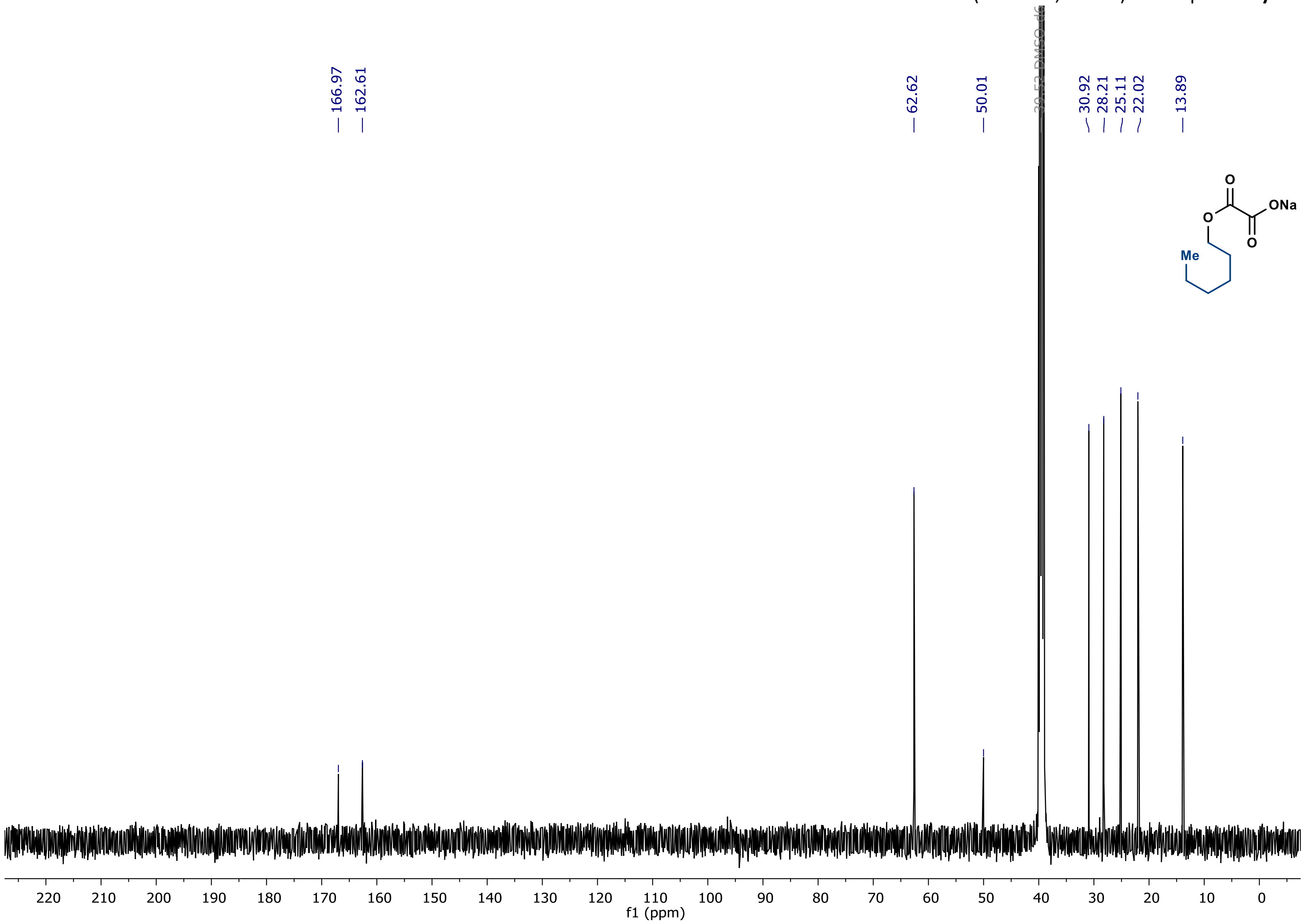

**$^{1}$H NMR** (500 MHz, $CDCl_3$) of compound **5a**

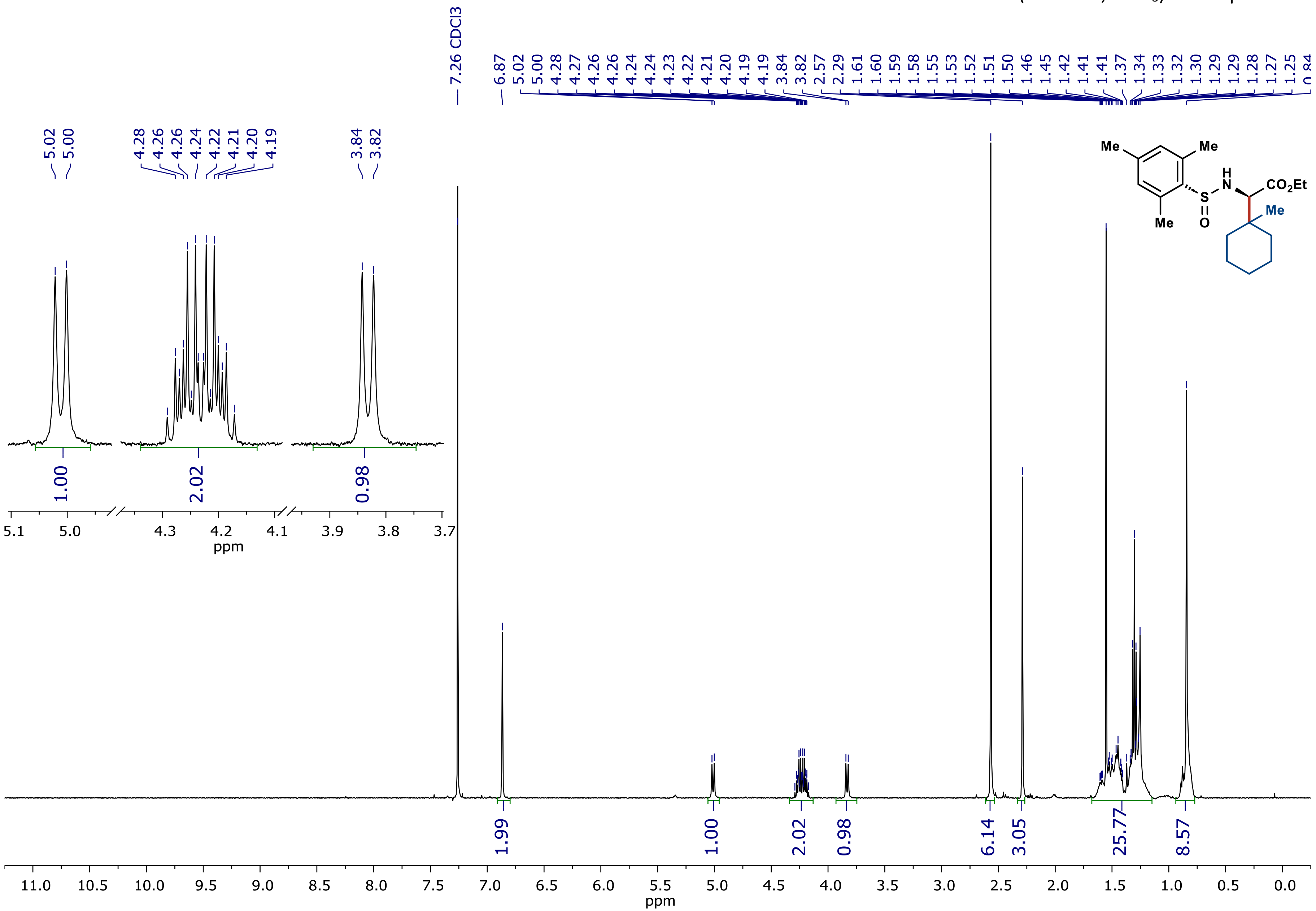

$^{13}$**C NMR** (126 MHz, $CDCl_3$) of compound **5a**

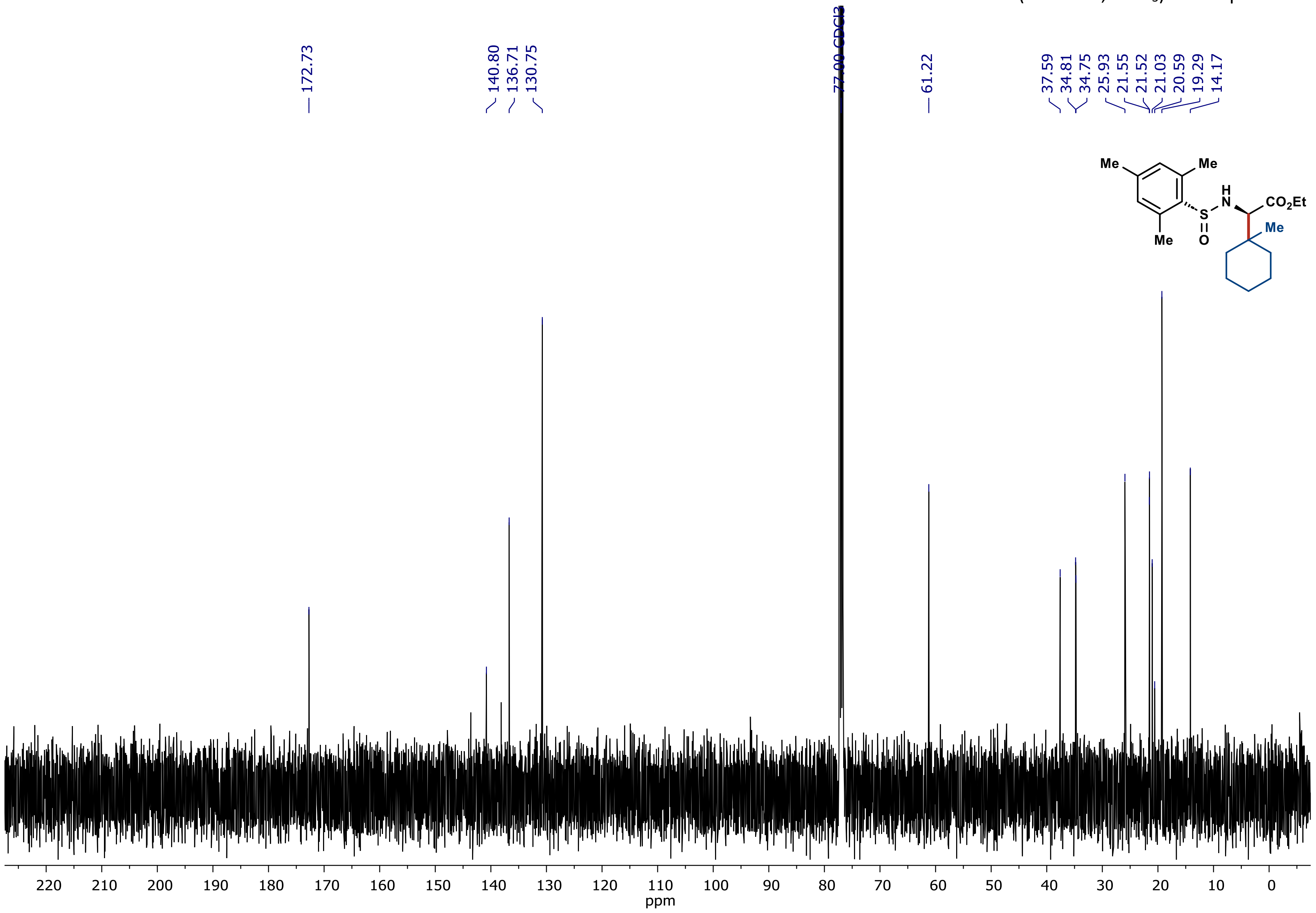

**¹H NMR** (500 MHz, $CDCl_3$) of compound **5b**

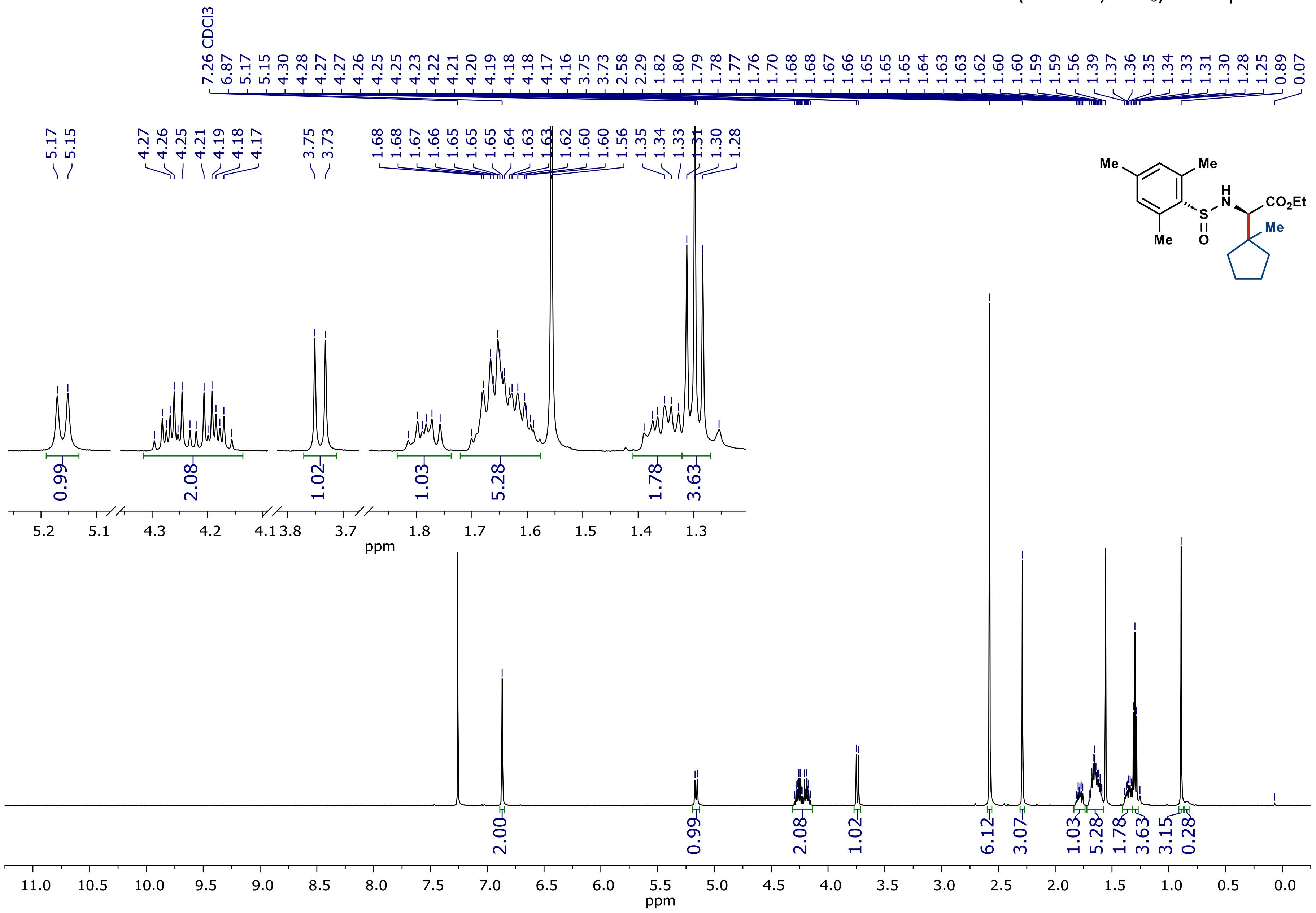

**$^{13}$C NMR** (126 MHz, $CDCl_3$) of compound **5b**

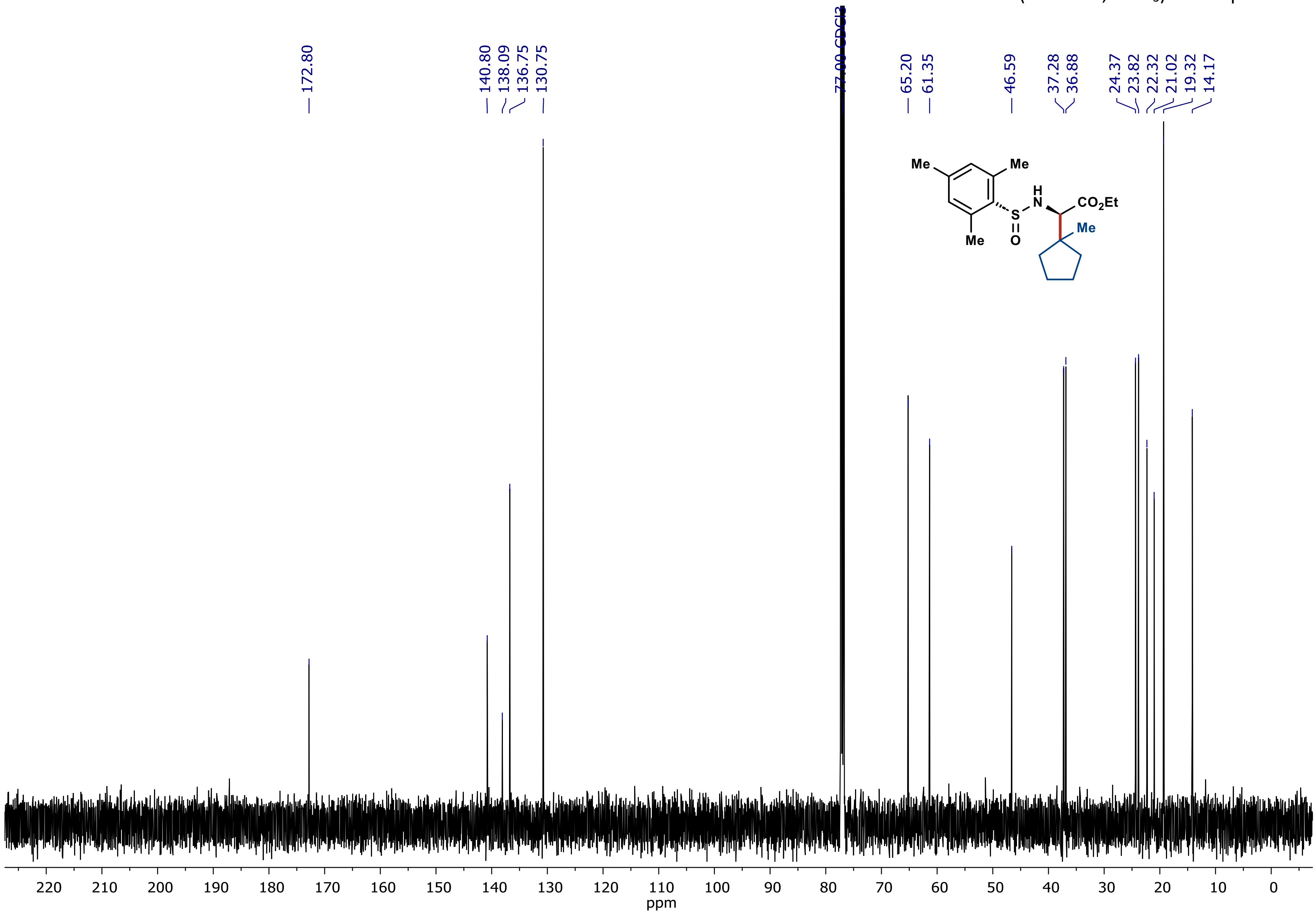

$^1$**H NMR** (500 MHz, $CDCl_3$) of compound **5c**

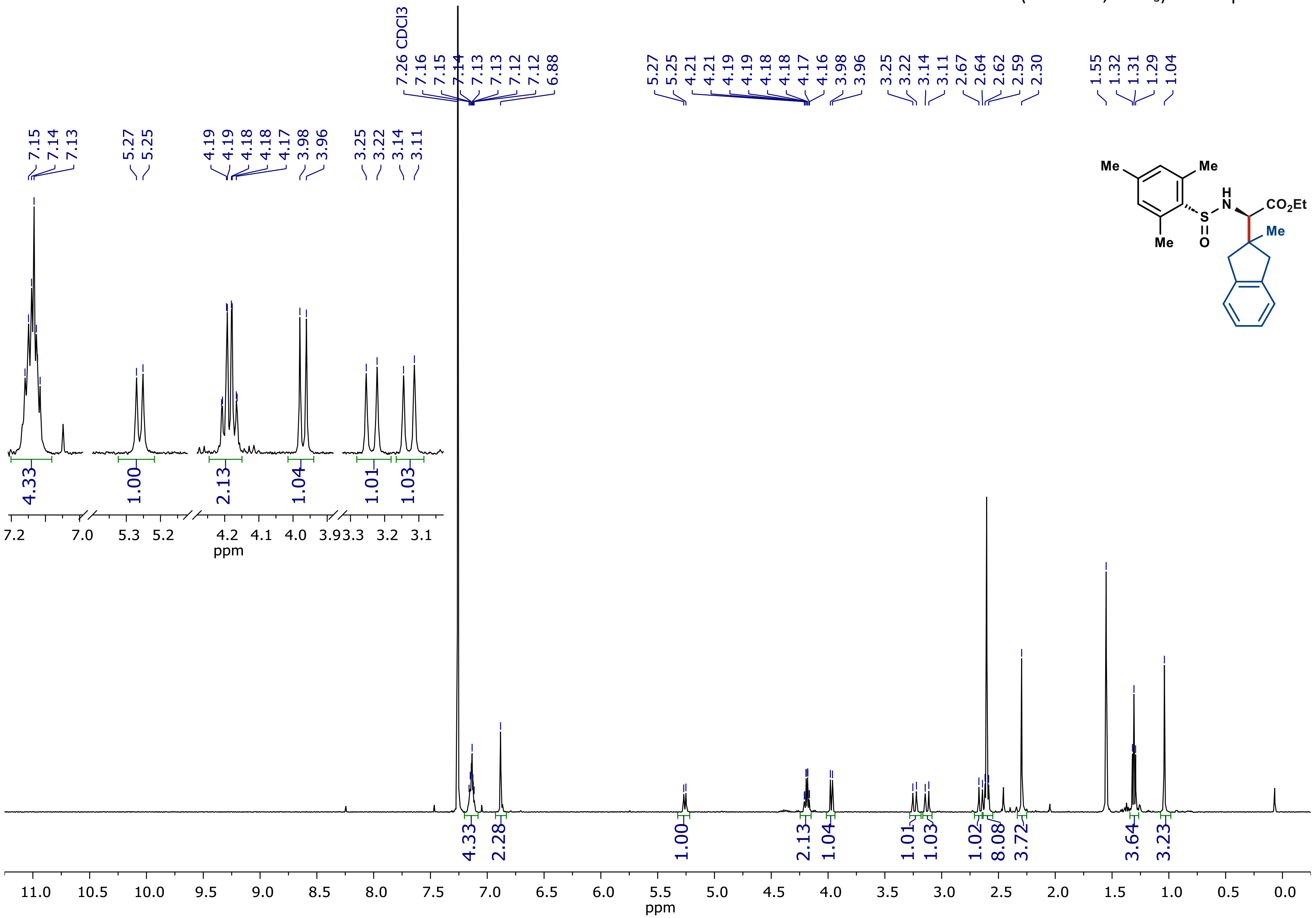

**$^{13}$C NMR** (126 MHz, $CDCl_3$) of compound **5c**

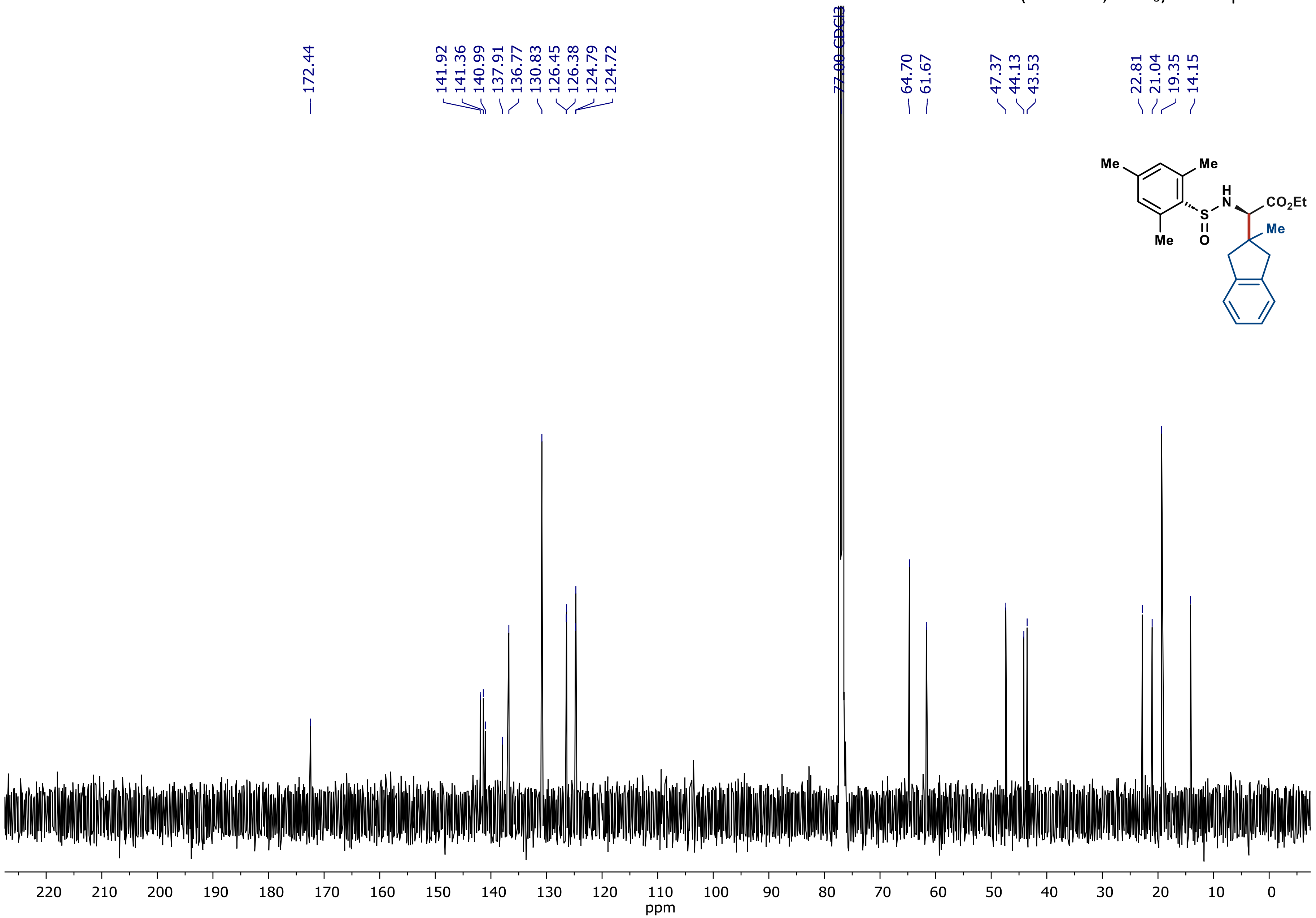

COSY of compound **5c**

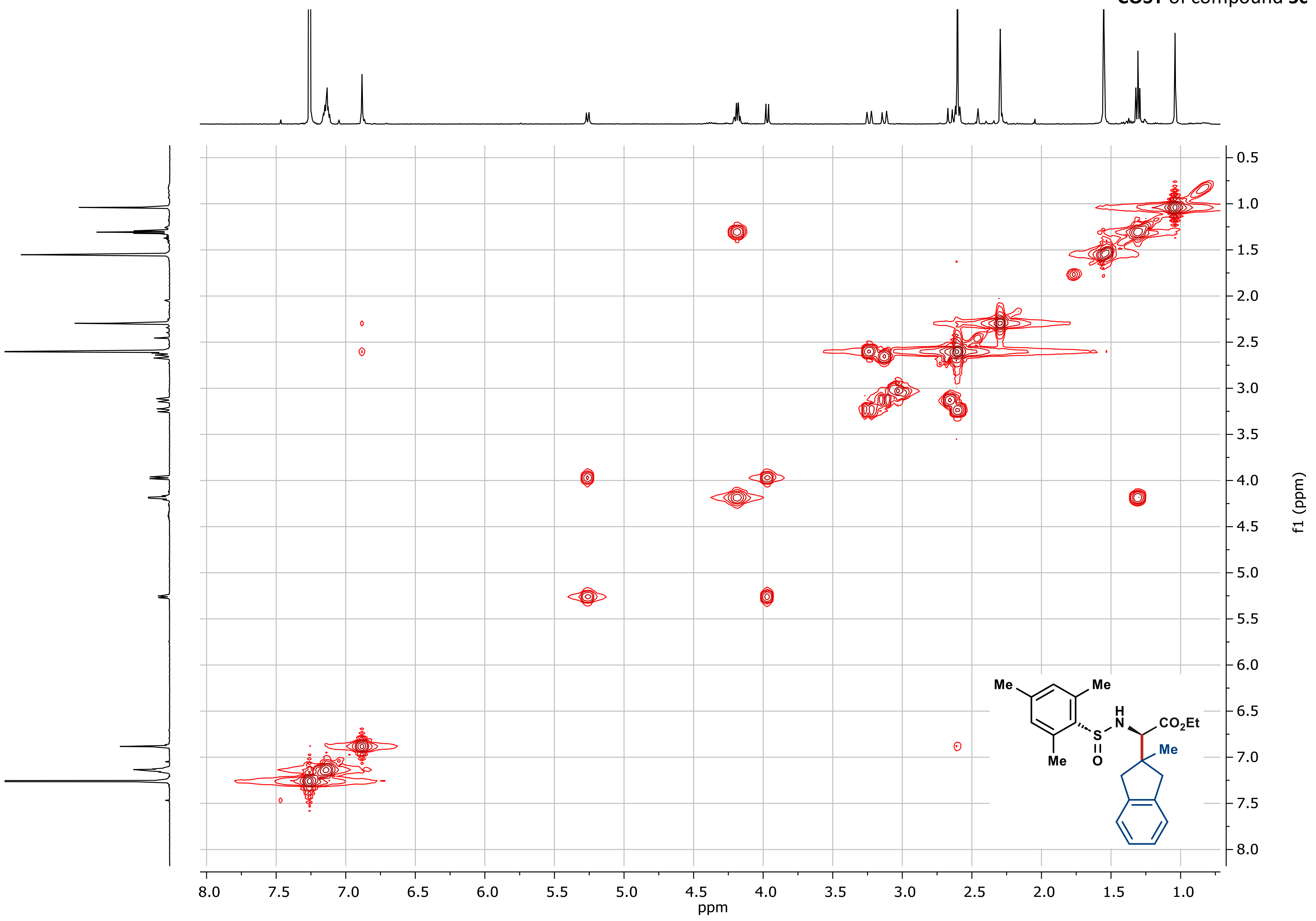

**HSQC** of compound **5c**

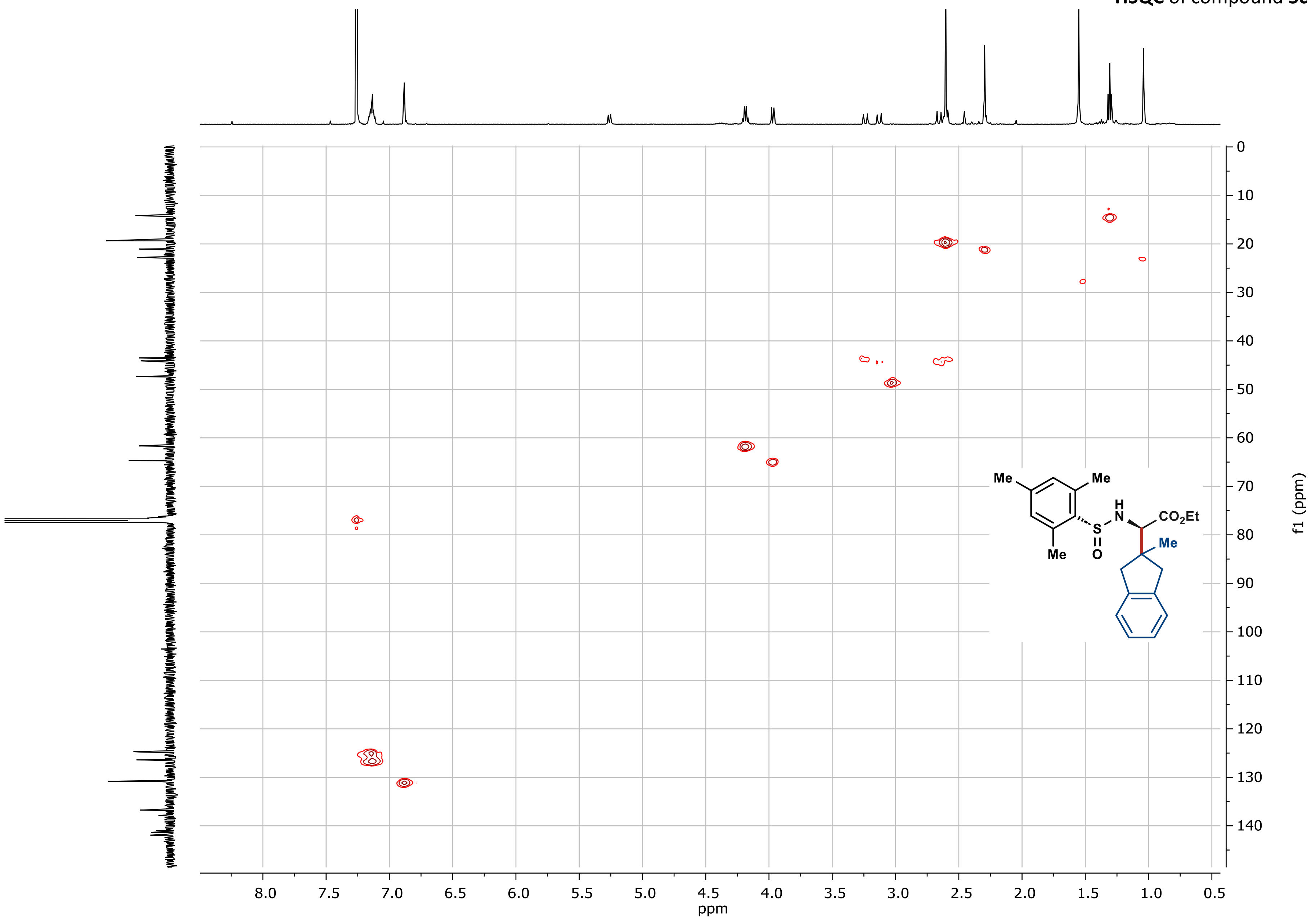

**¹H NMR** (500 MHz, $CDCl_3$) of compound **5d**

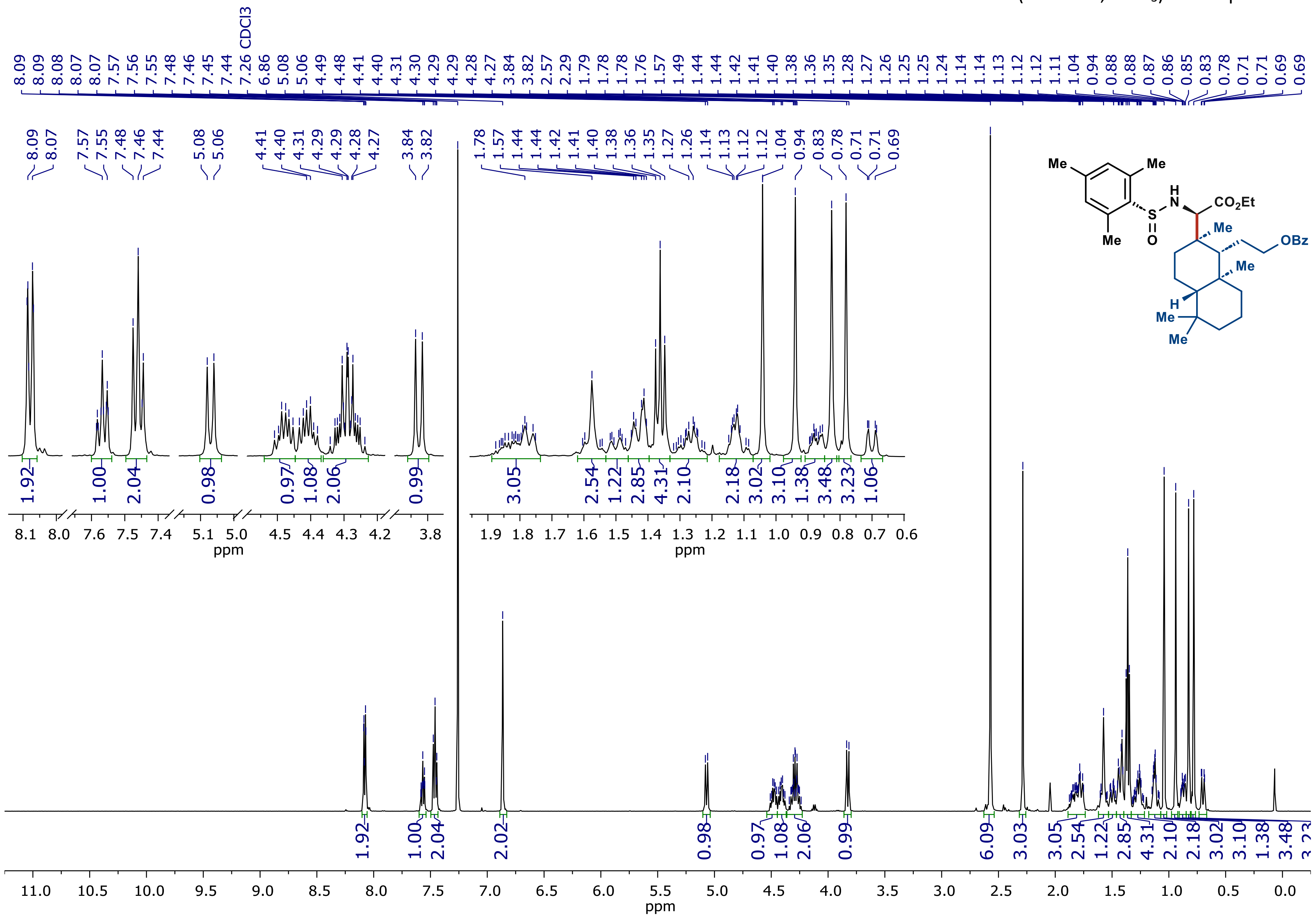

**$^{13}$C NMR** (126 MHz, $CDCl_3$) of compound **5d**

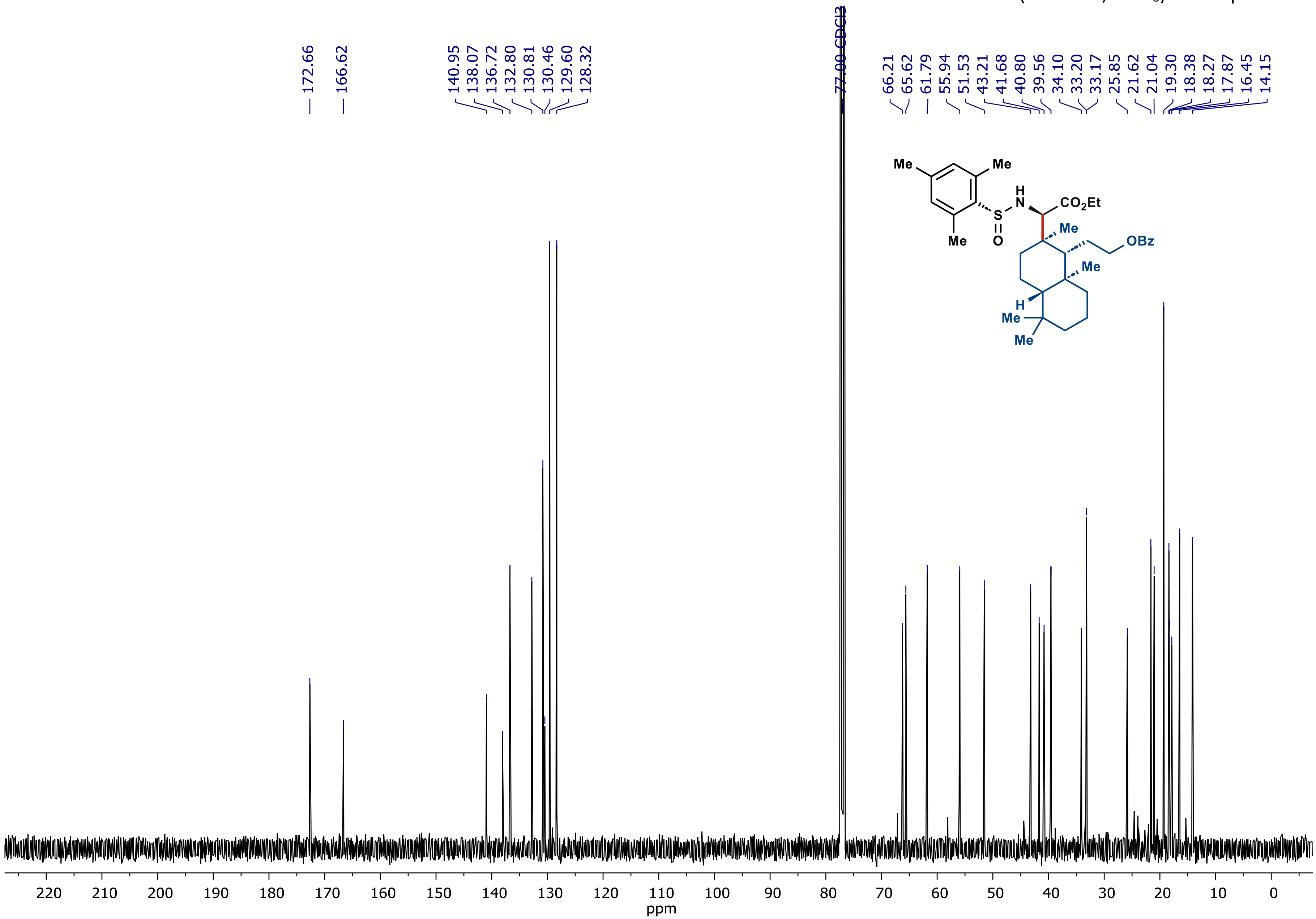

**COSY** of compound **5d**

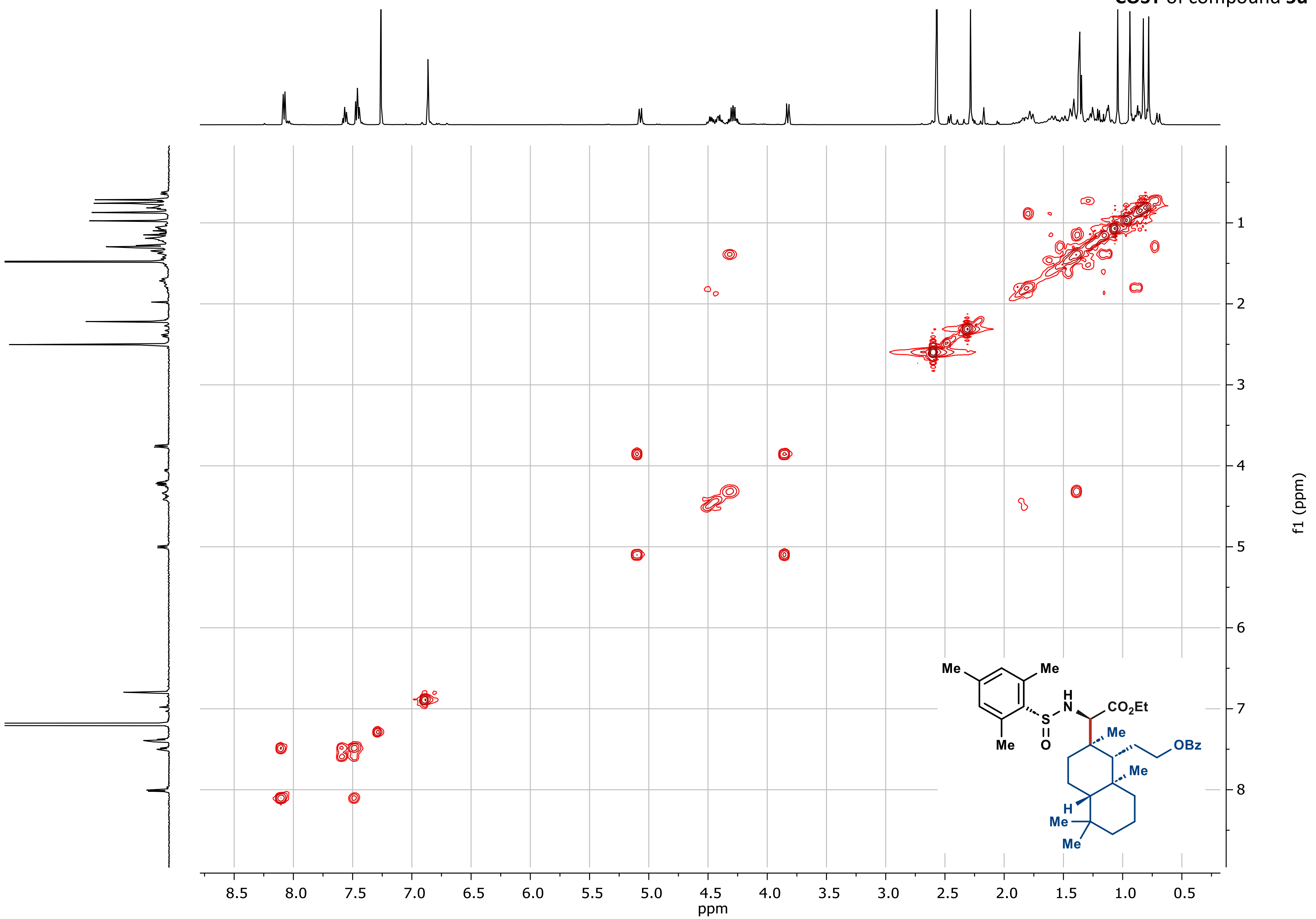

**HSQC** of compound **5d**

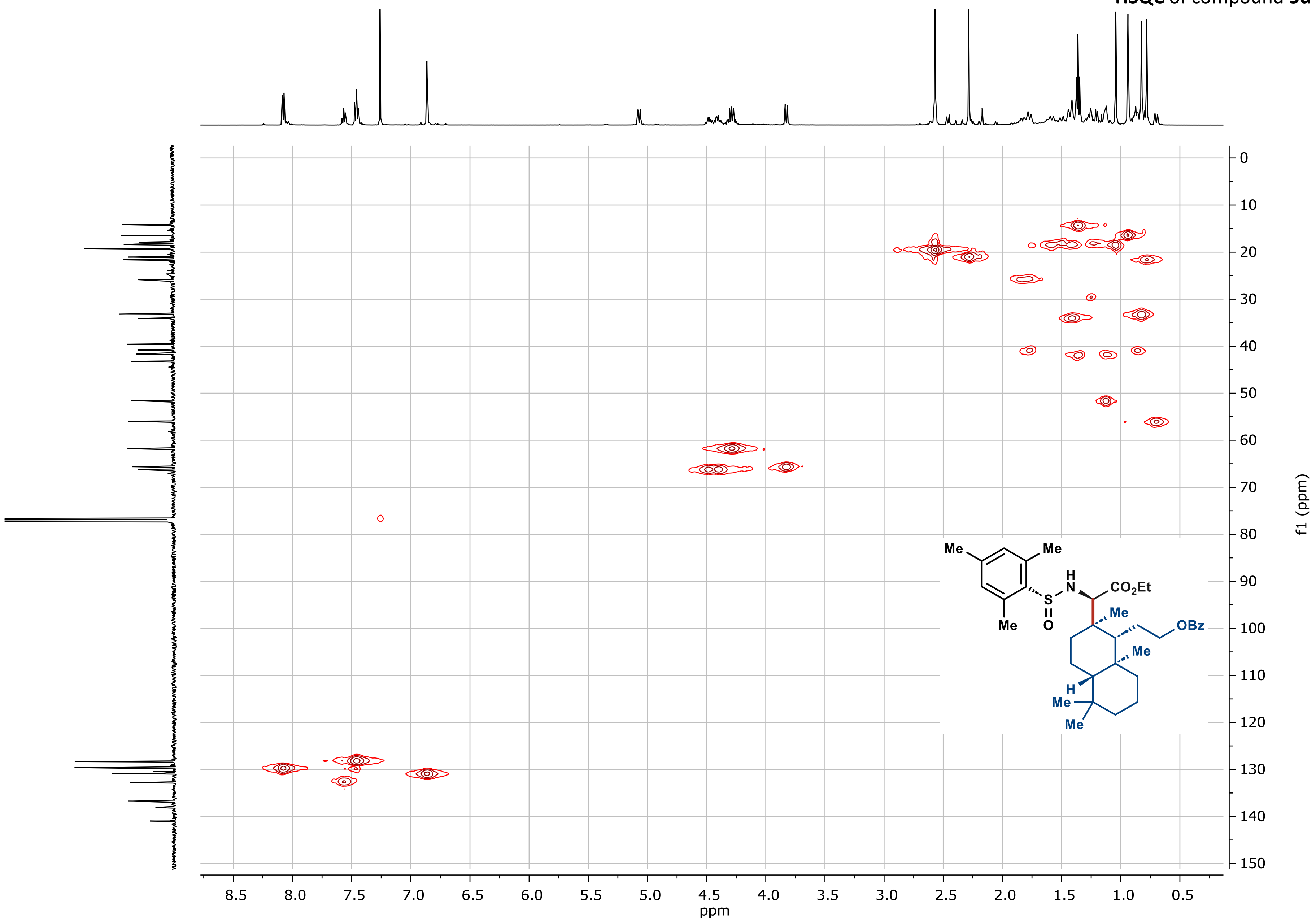

**HMBC** of compound **5d**

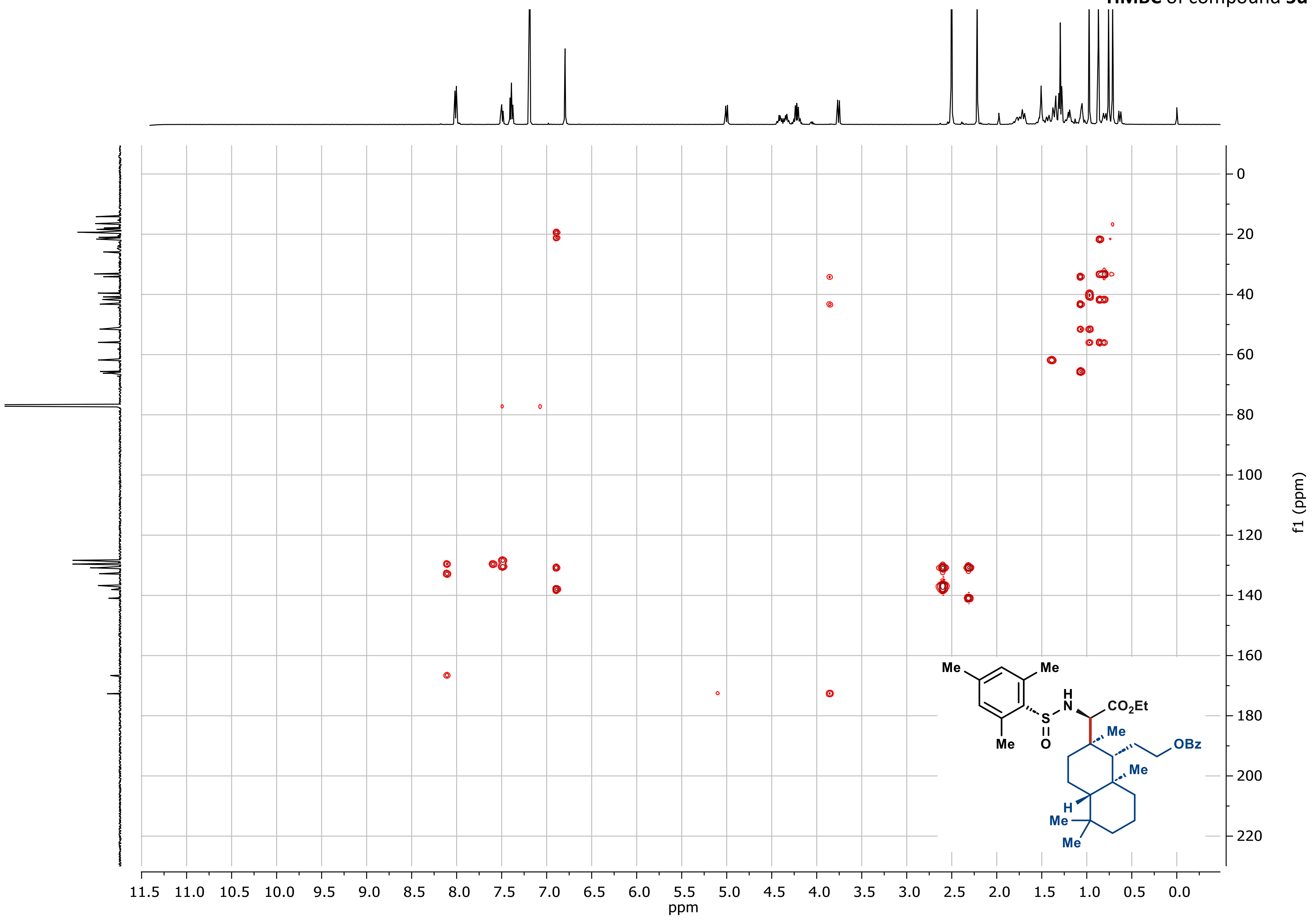

**$^1$H NMR** (500 MHz, $CDCl_3$) of compound **5e**

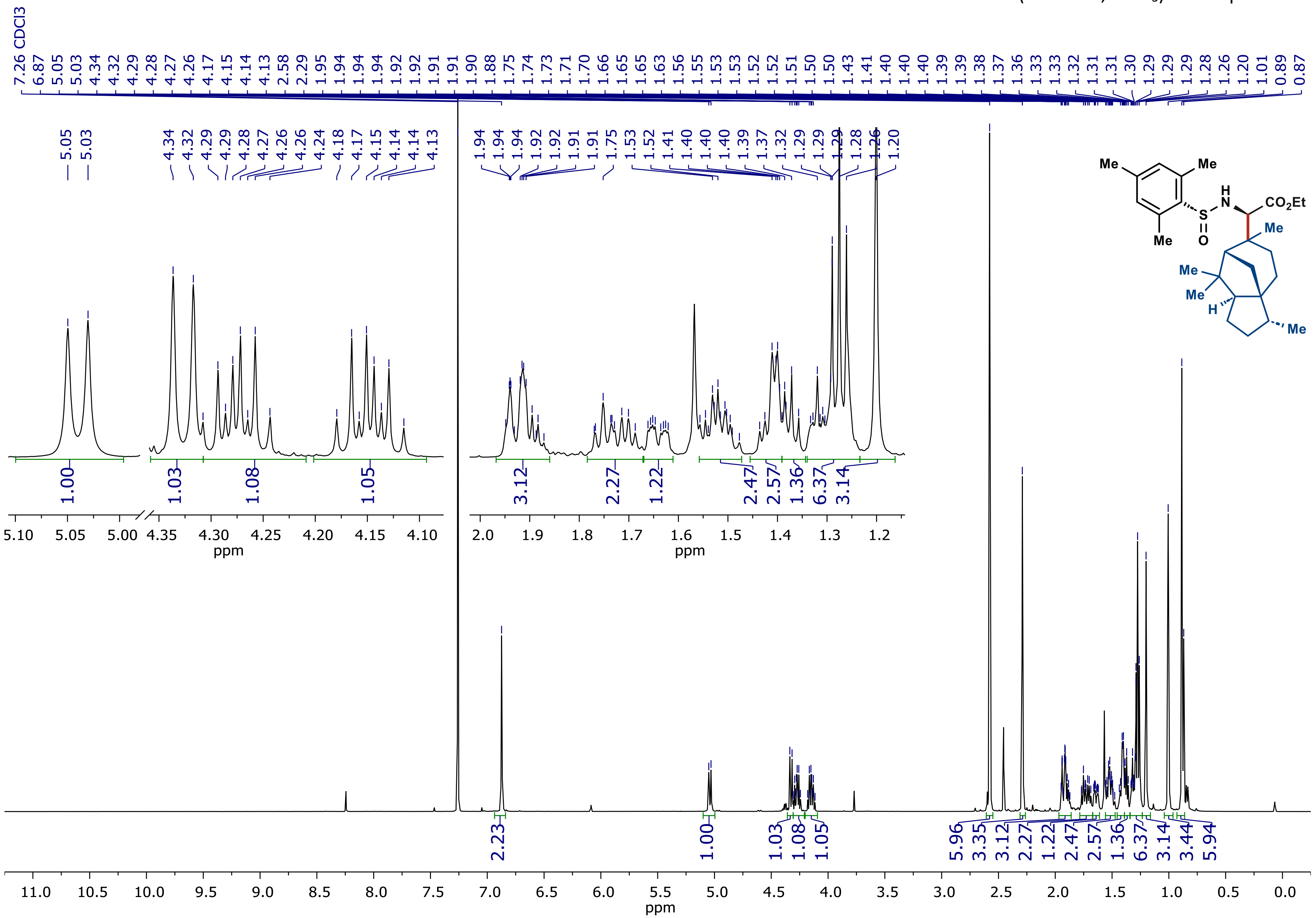

**$^{13}$C NMR** (126 MHz, $CDCl_3$) of compound **5e**

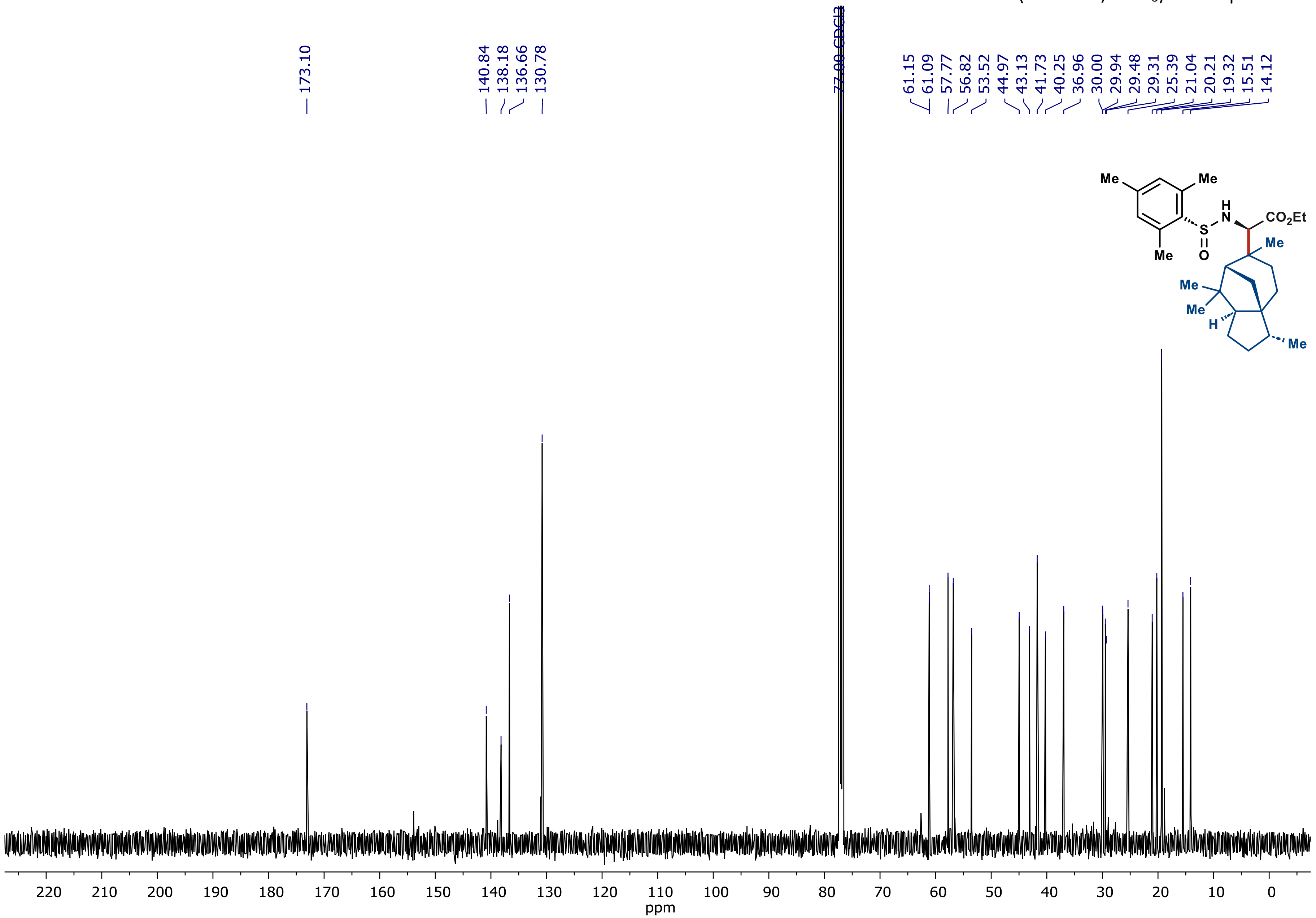

COSY of compound **5e**

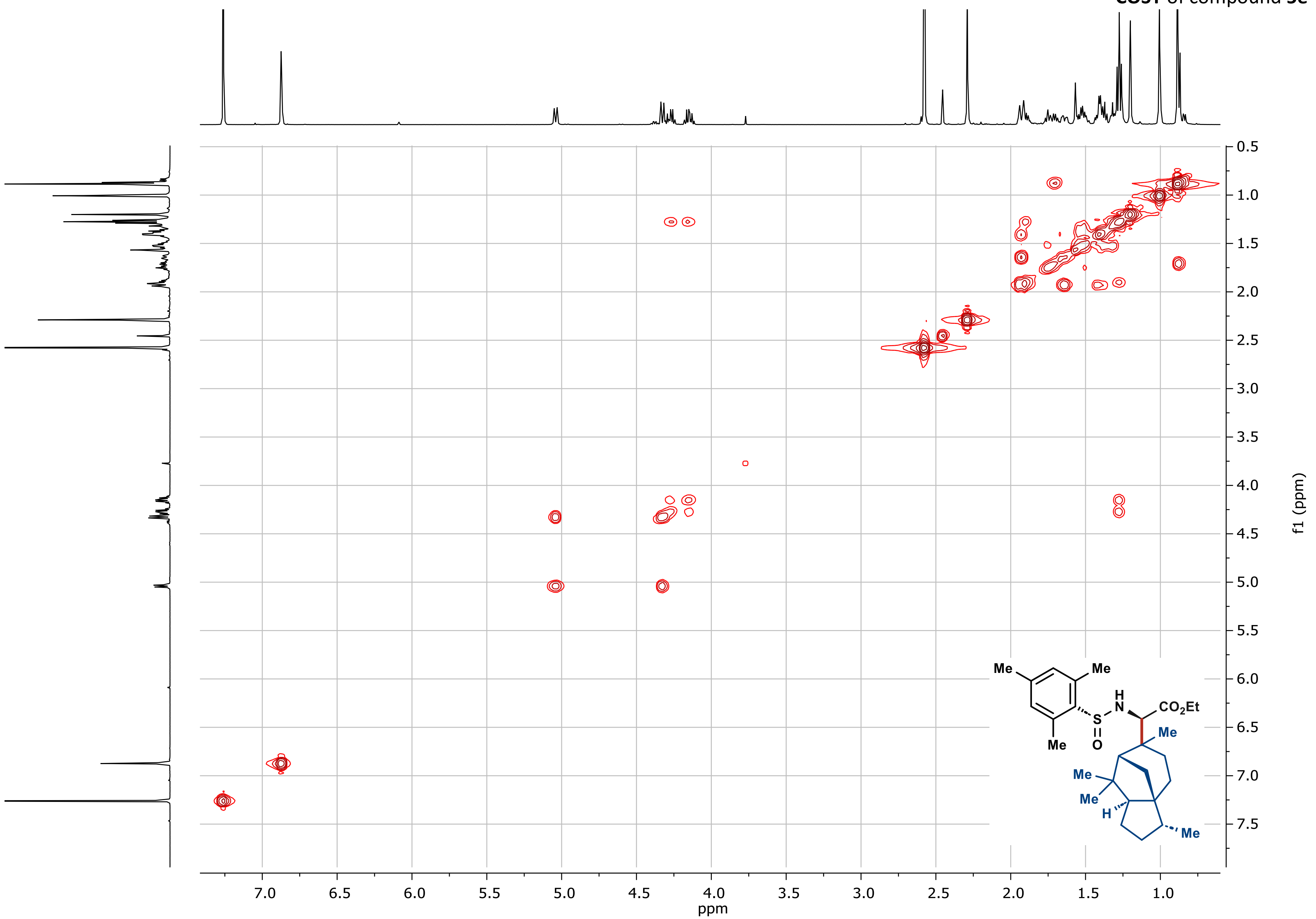

HSQC of compound **5e**

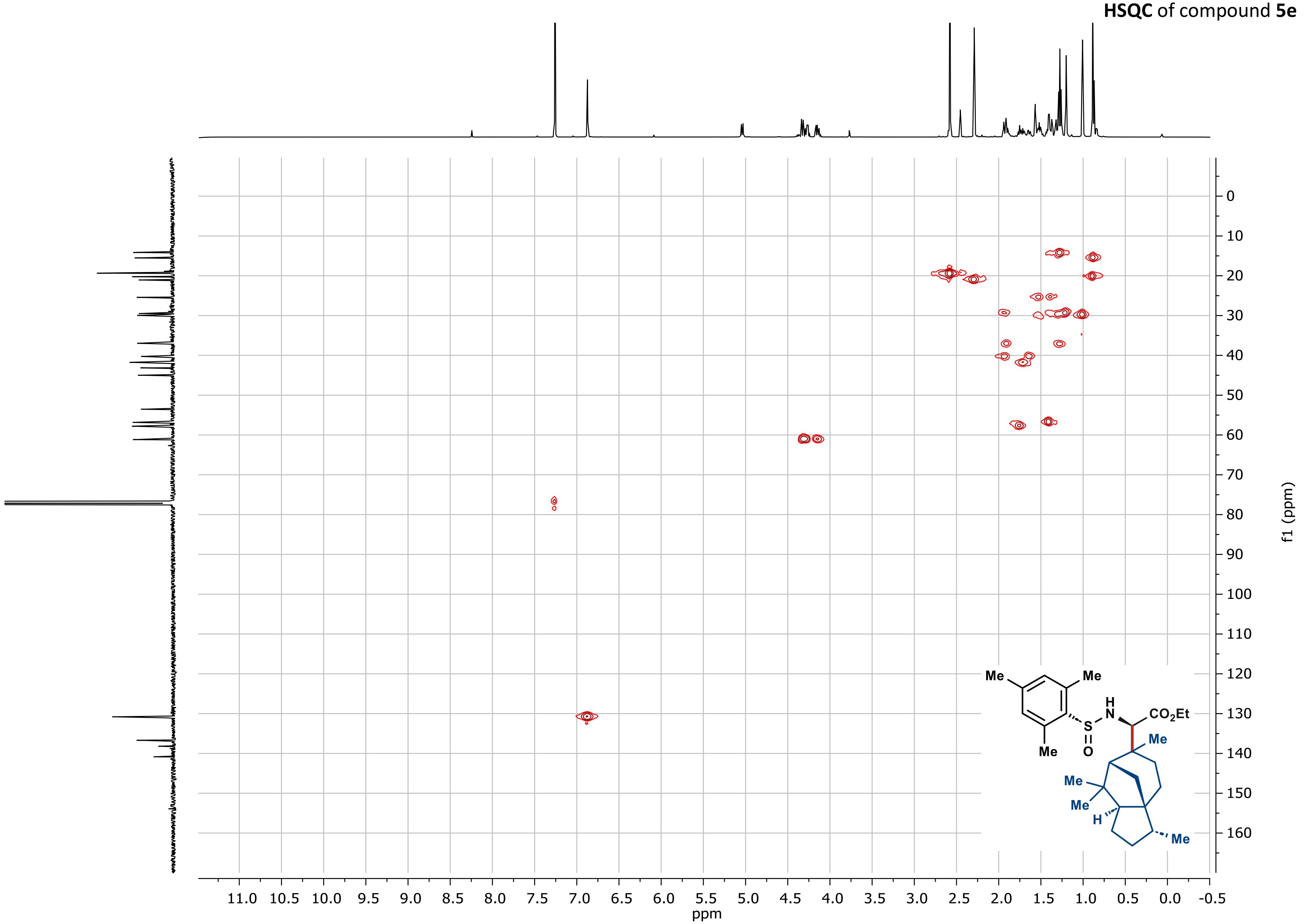

[1]**H NMR** (500 MHz, $CDCl_3$) of compound **5f**

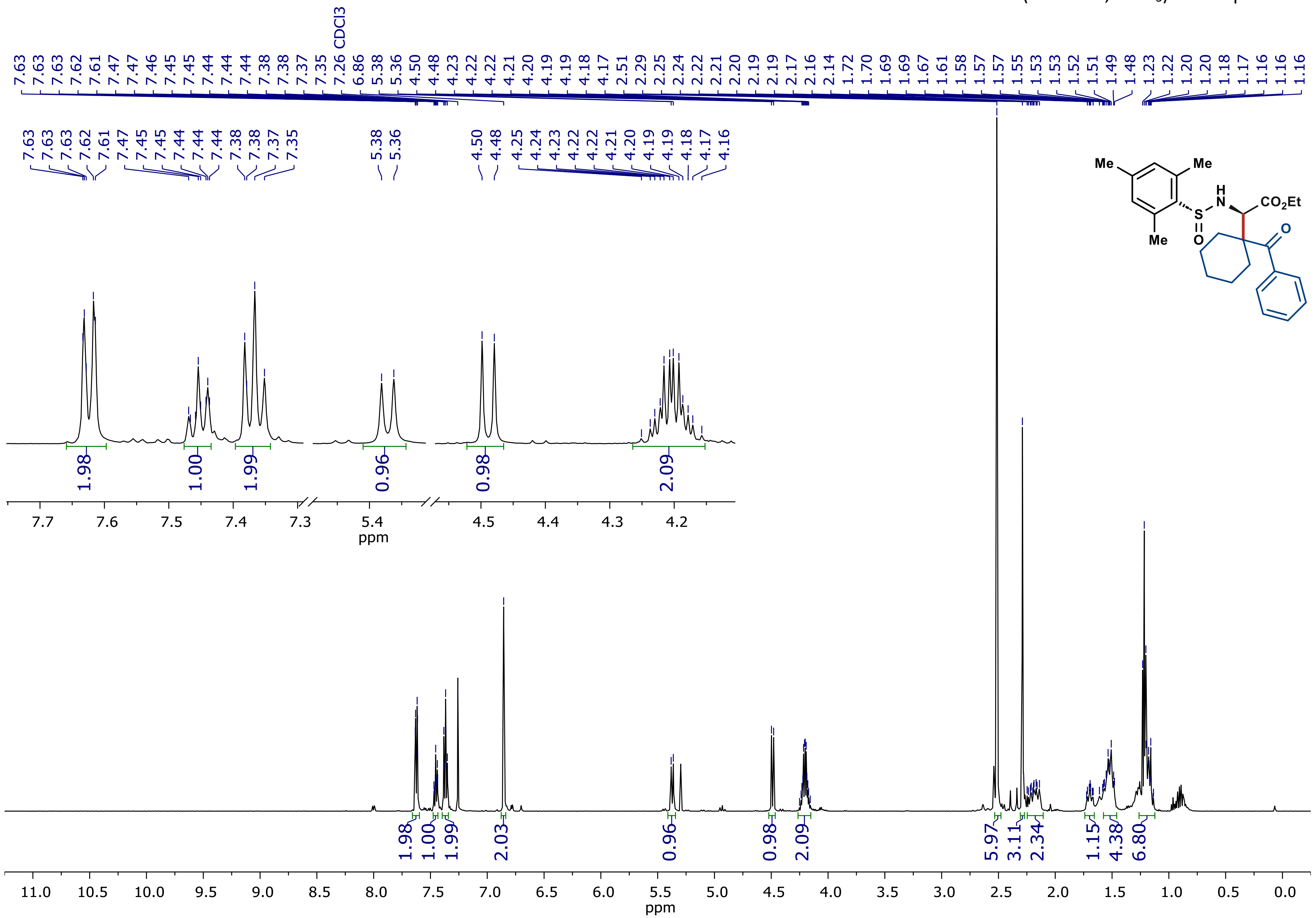

**$^{13}$C NMR** (126 MHz, $CDCl_3$) of compound **5f**

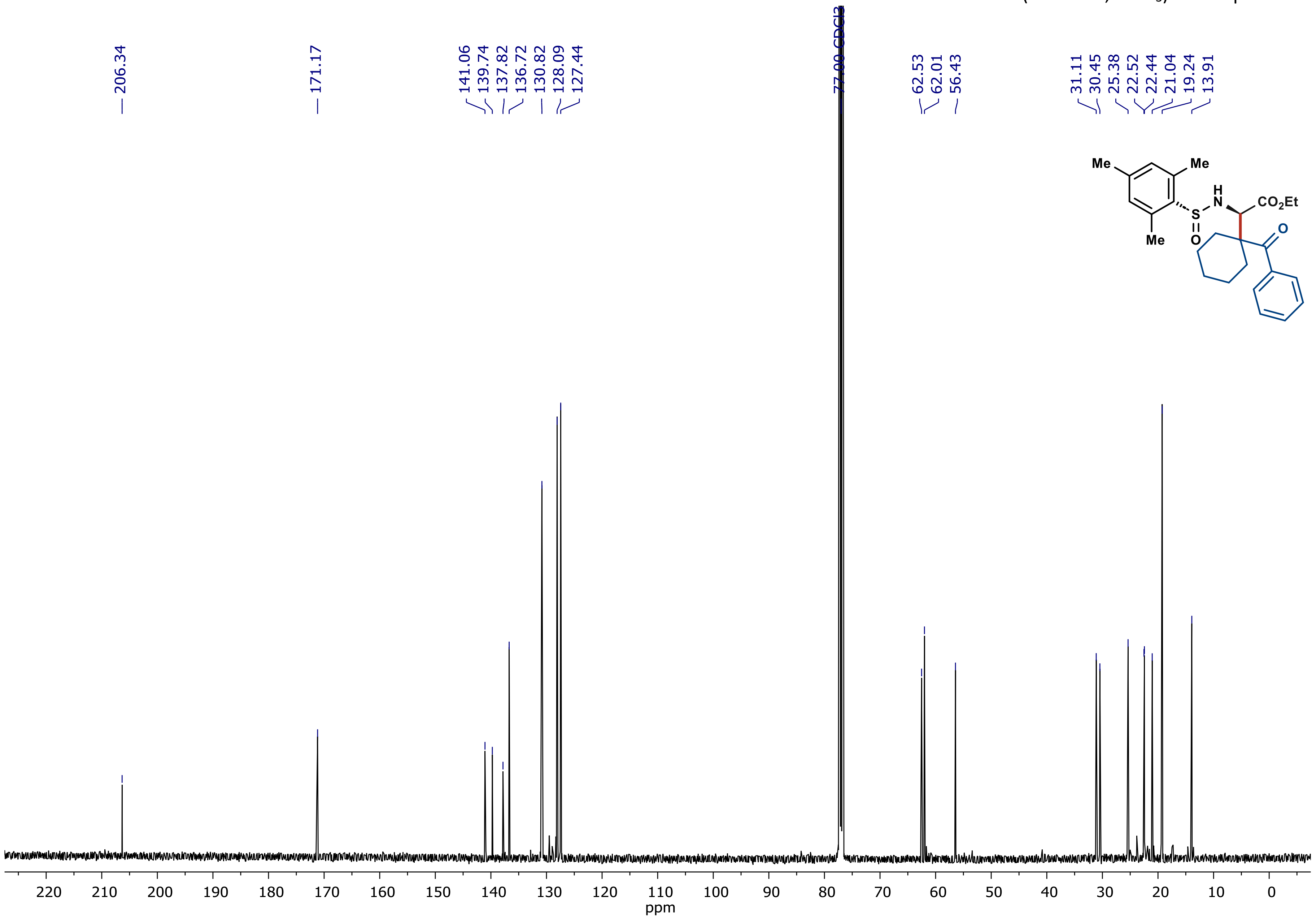

**COSY** of compound **5f**

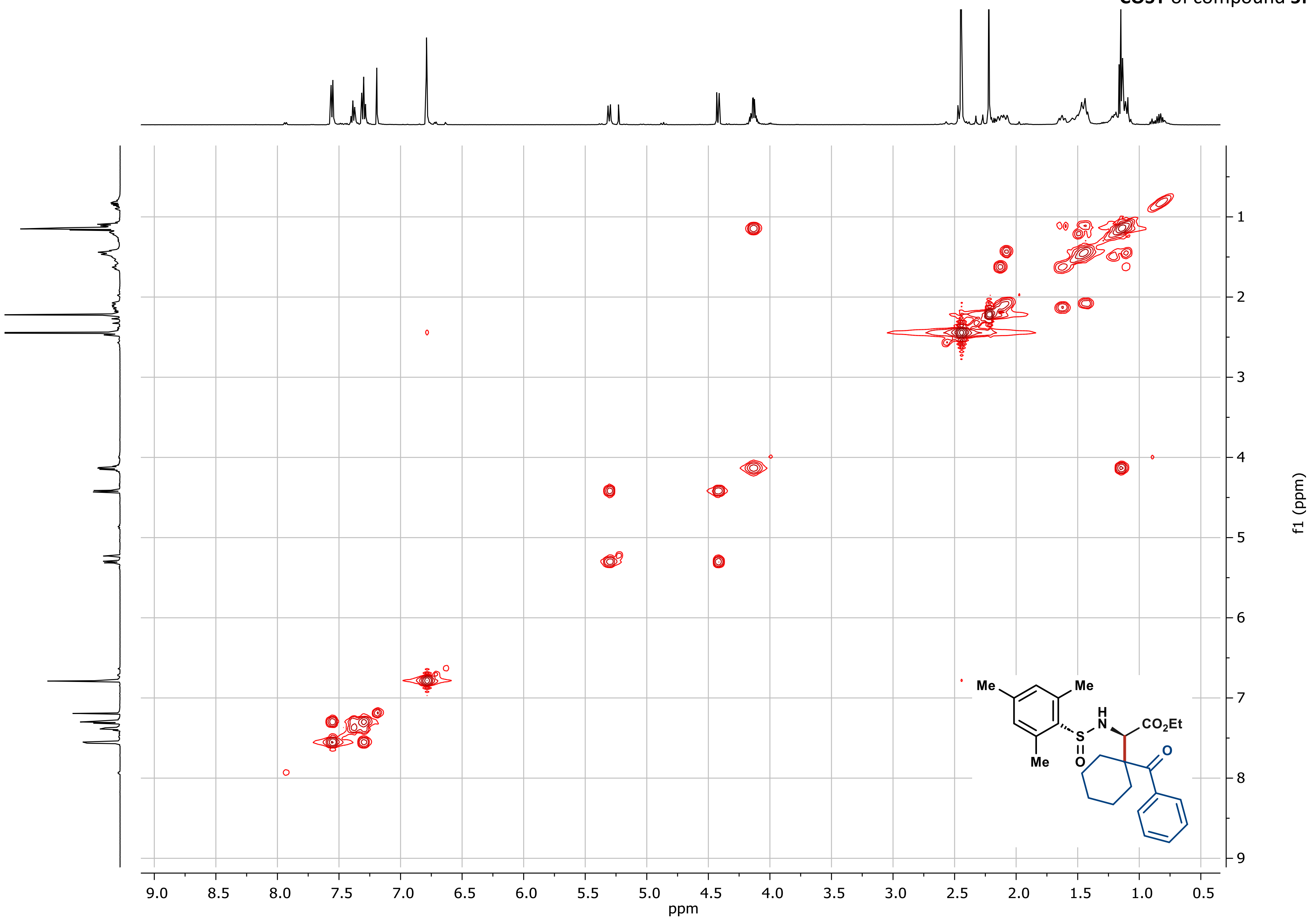

**HSQC** of compound **5f**

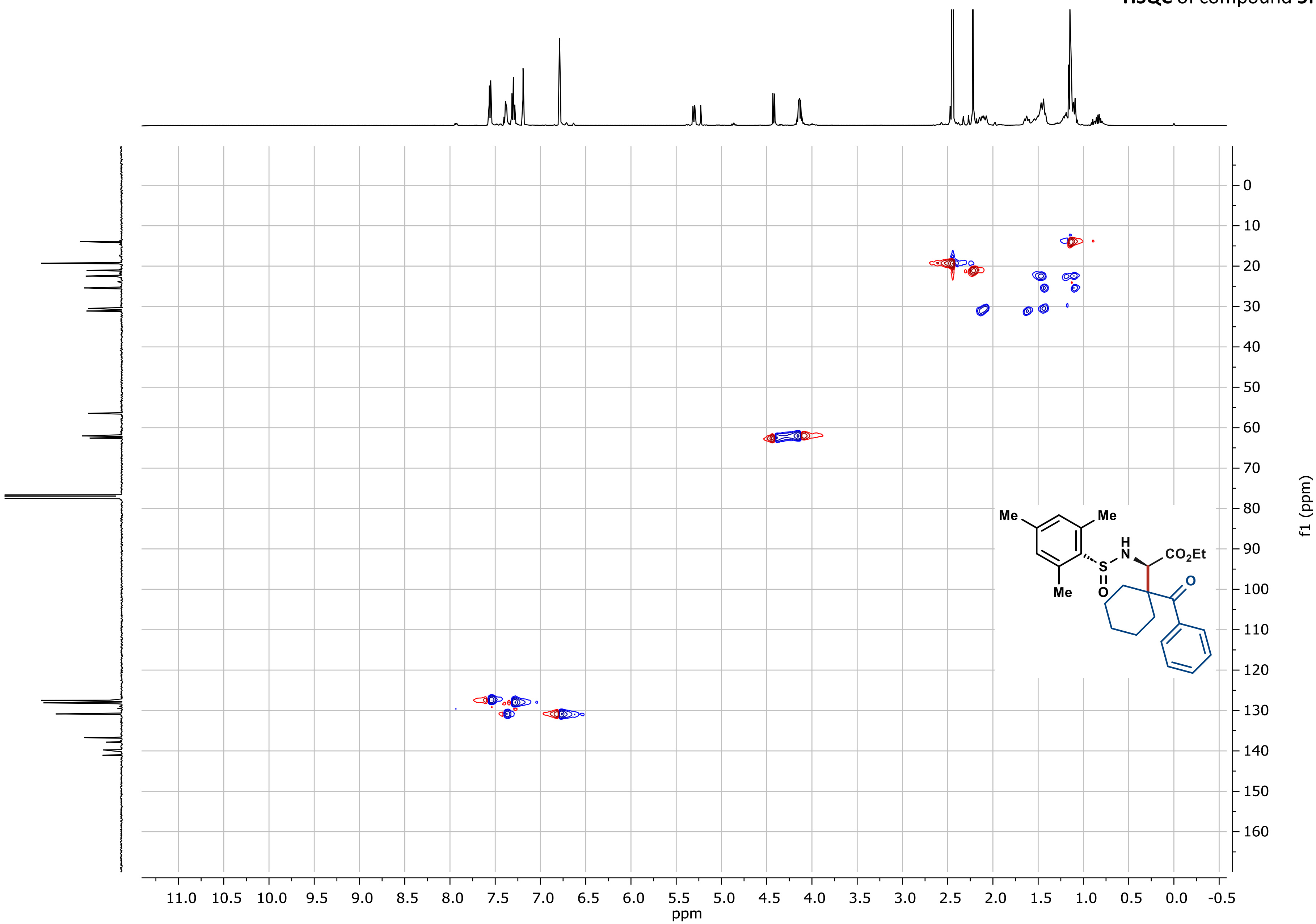

**$^1$H NMR** (500 MHz, $CDCl_3$) of compound **5g**

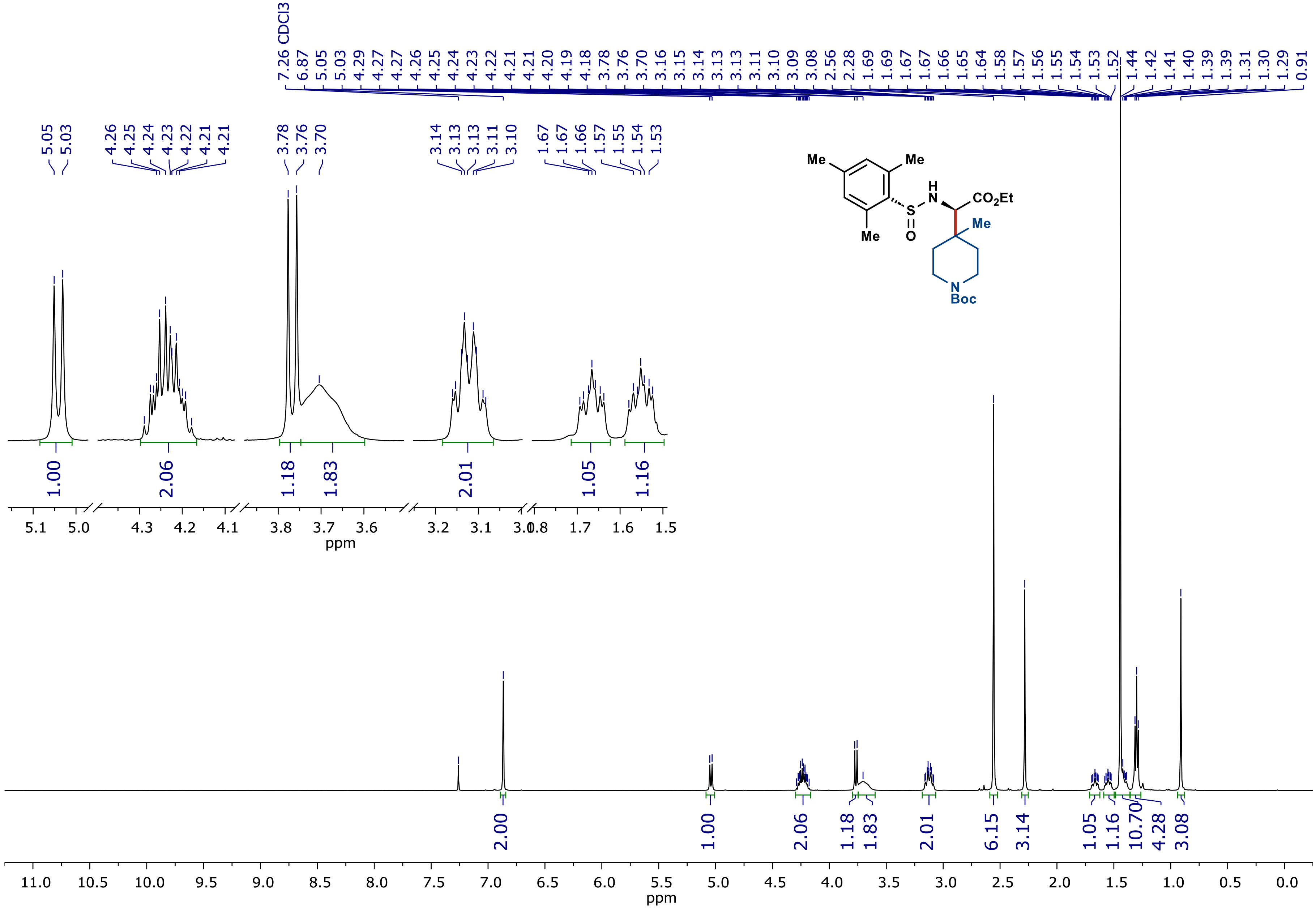

**$^{13}$C NMR** (126 MHz, $CDCl_3$) of compound **5g**

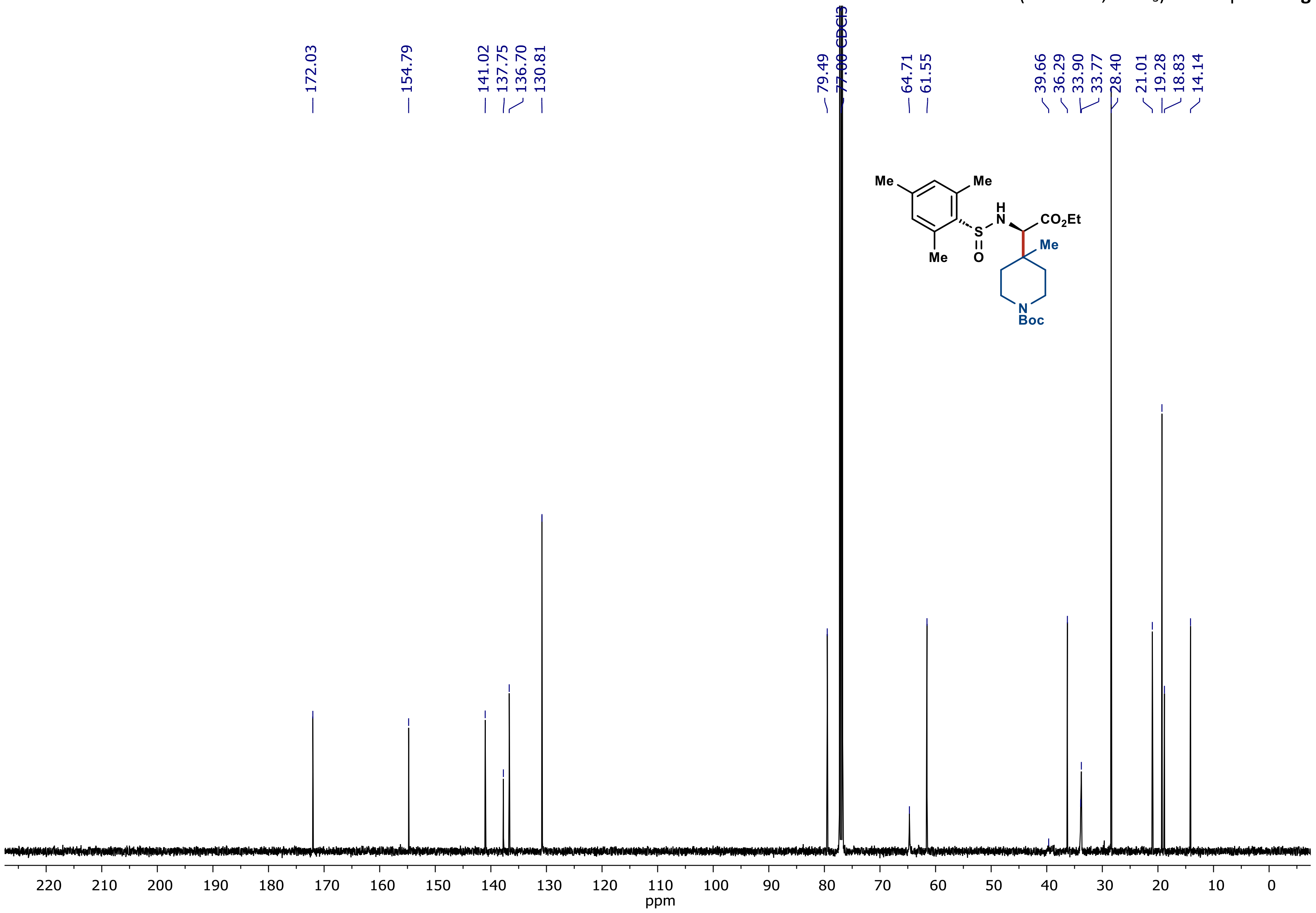

**$^1$H NMR** (500 MHz, $CDCl_3$) of compound **5h**

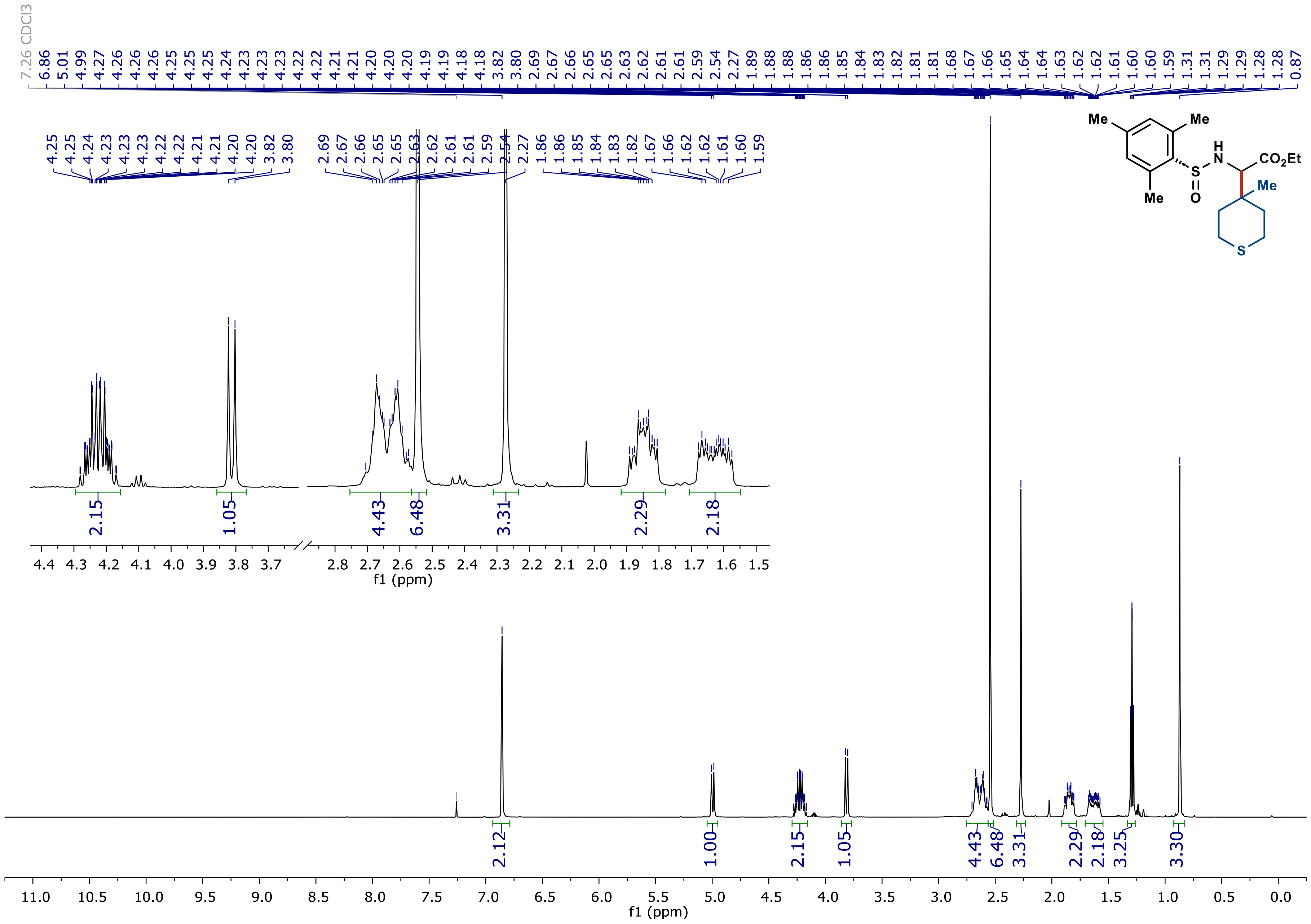

**$^{13}$C NMR** (126 MHz, $CDCl_3$) of compound **5h**

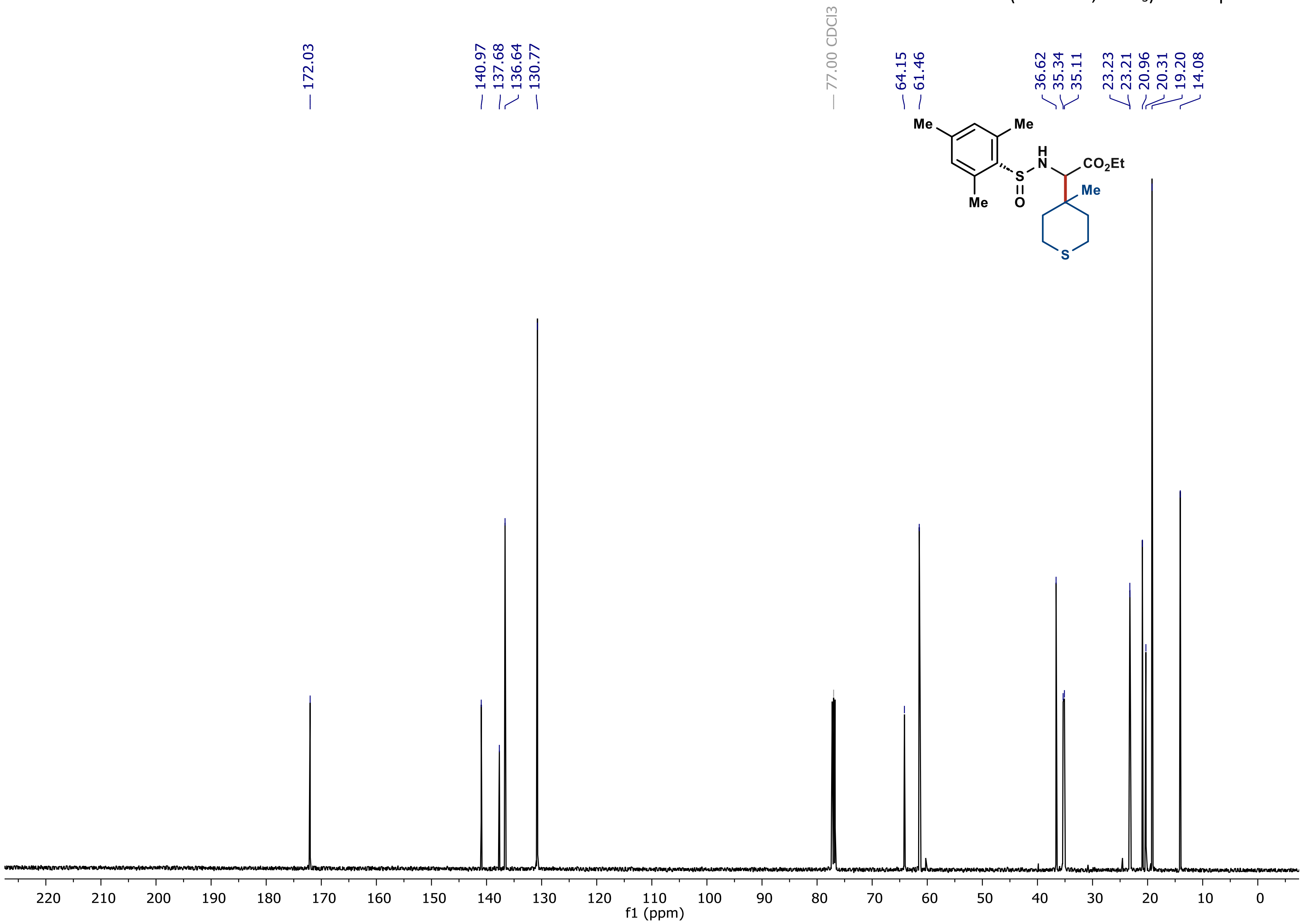

**COSY** of compound **5h**

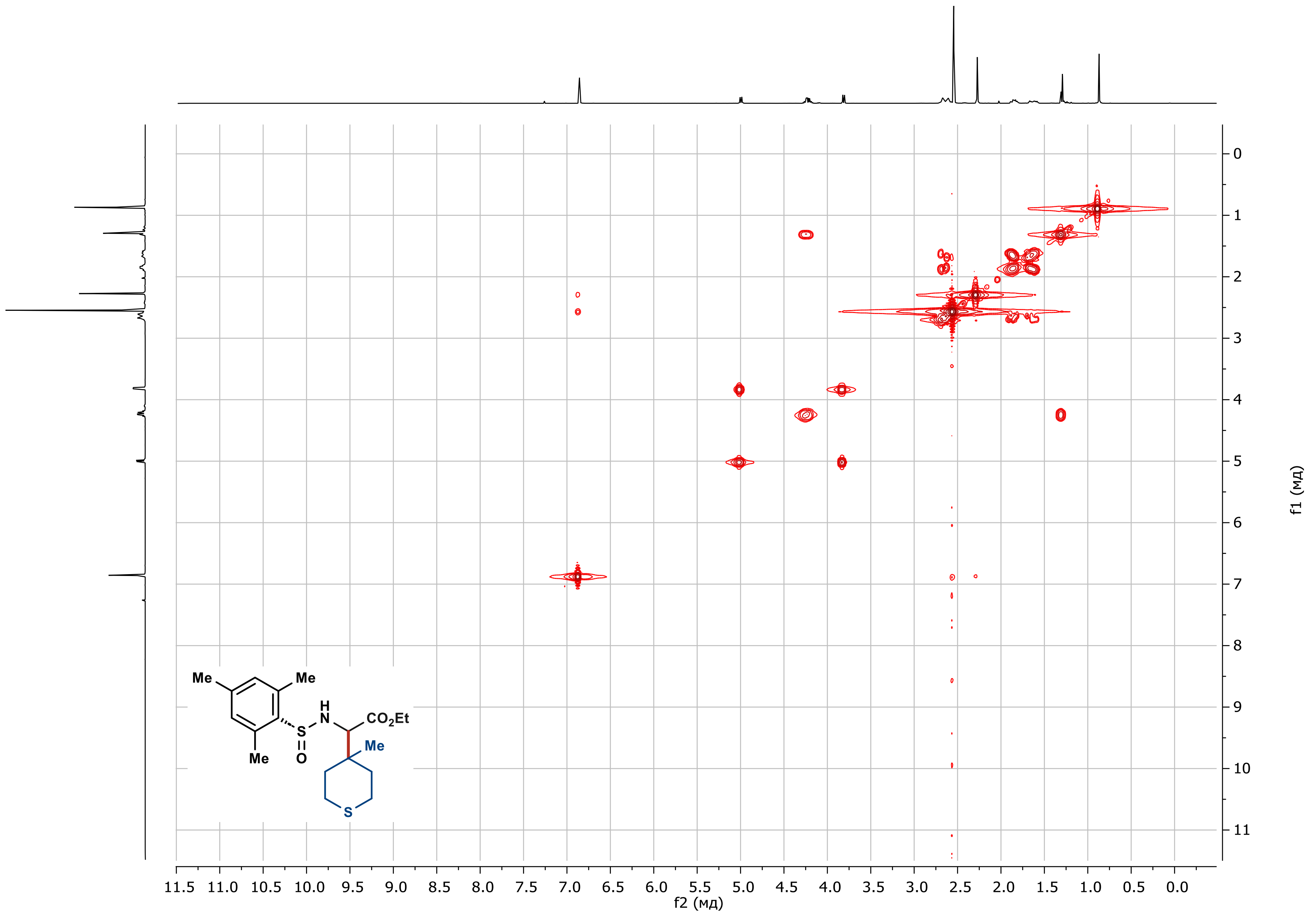

**HSQC** of compound **5h**

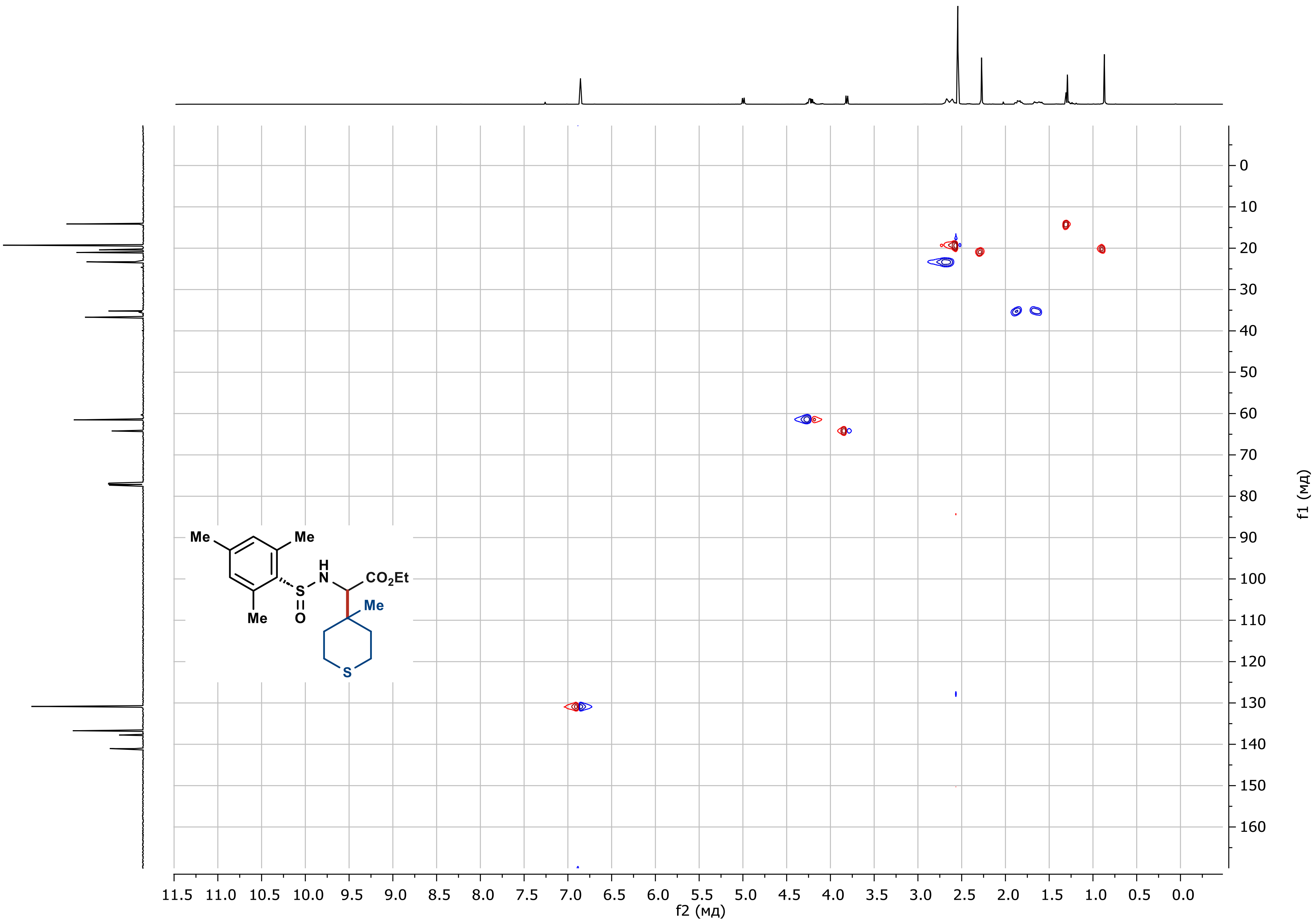

**$^1$H NMR** (500 MHz, $CDCl_3$) of compound **5i**

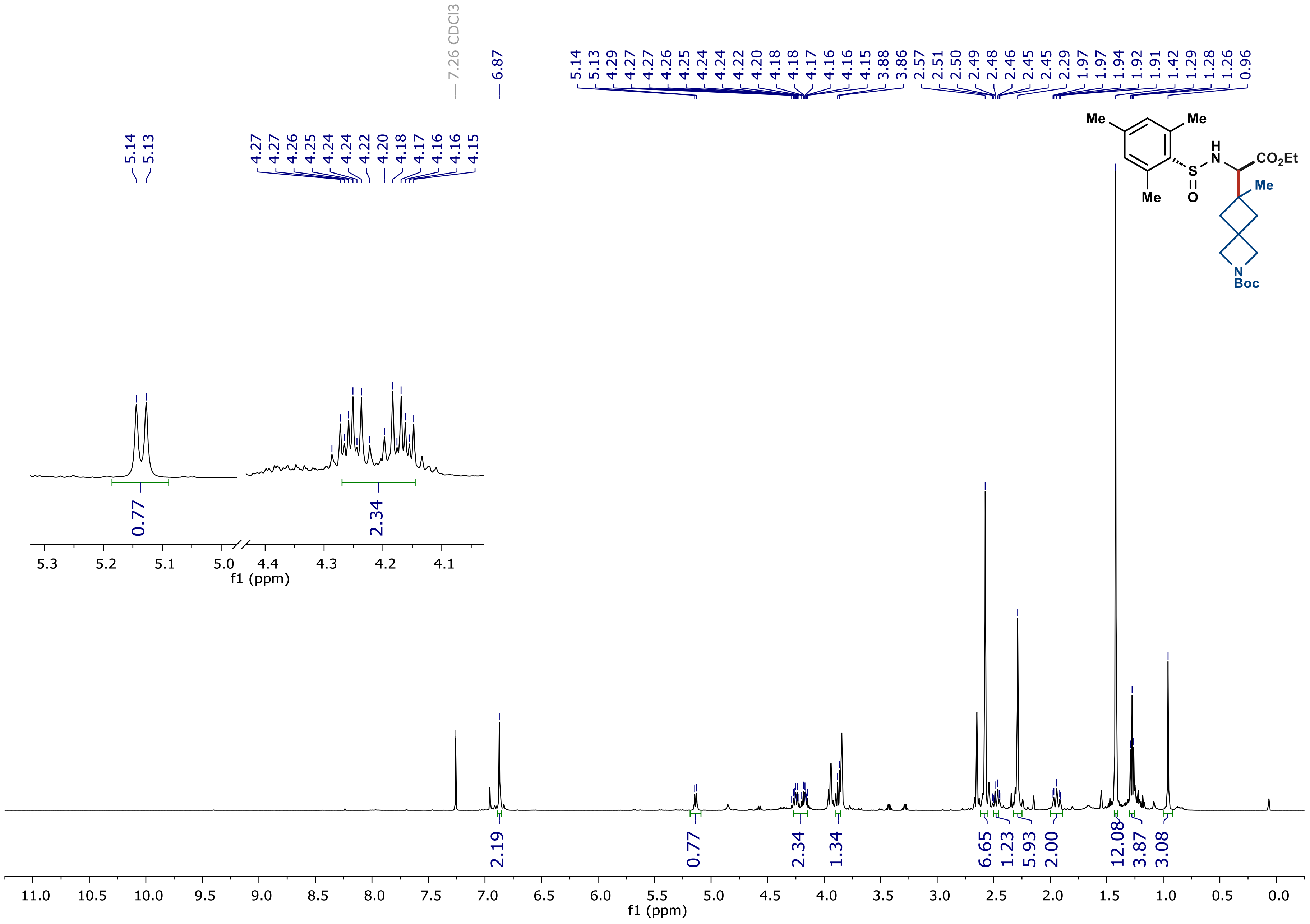

$^{13}$**C NMR** (126 MHz, $CDCl_3$) of compound **5i**

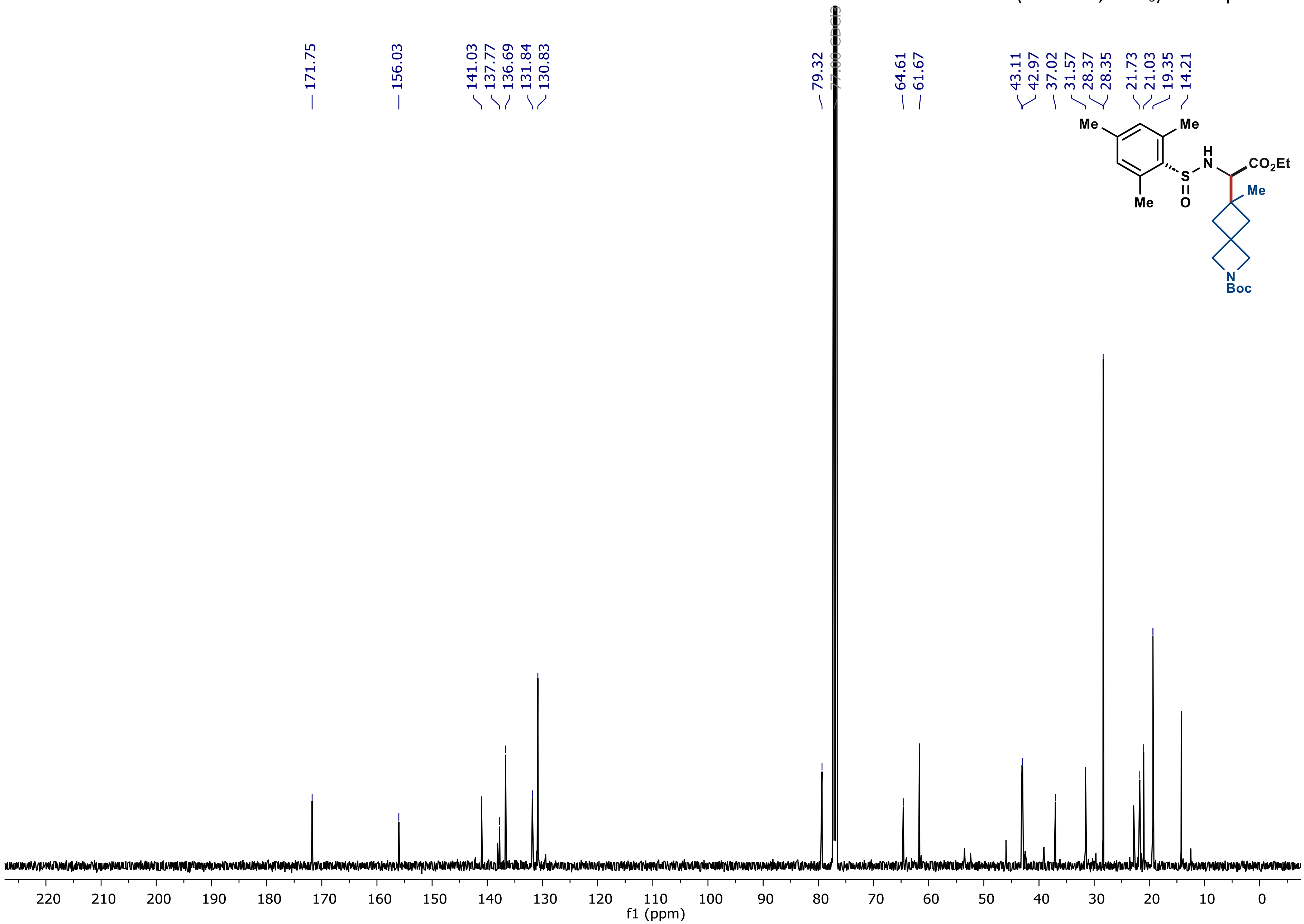

**COSY** of compound **5i**

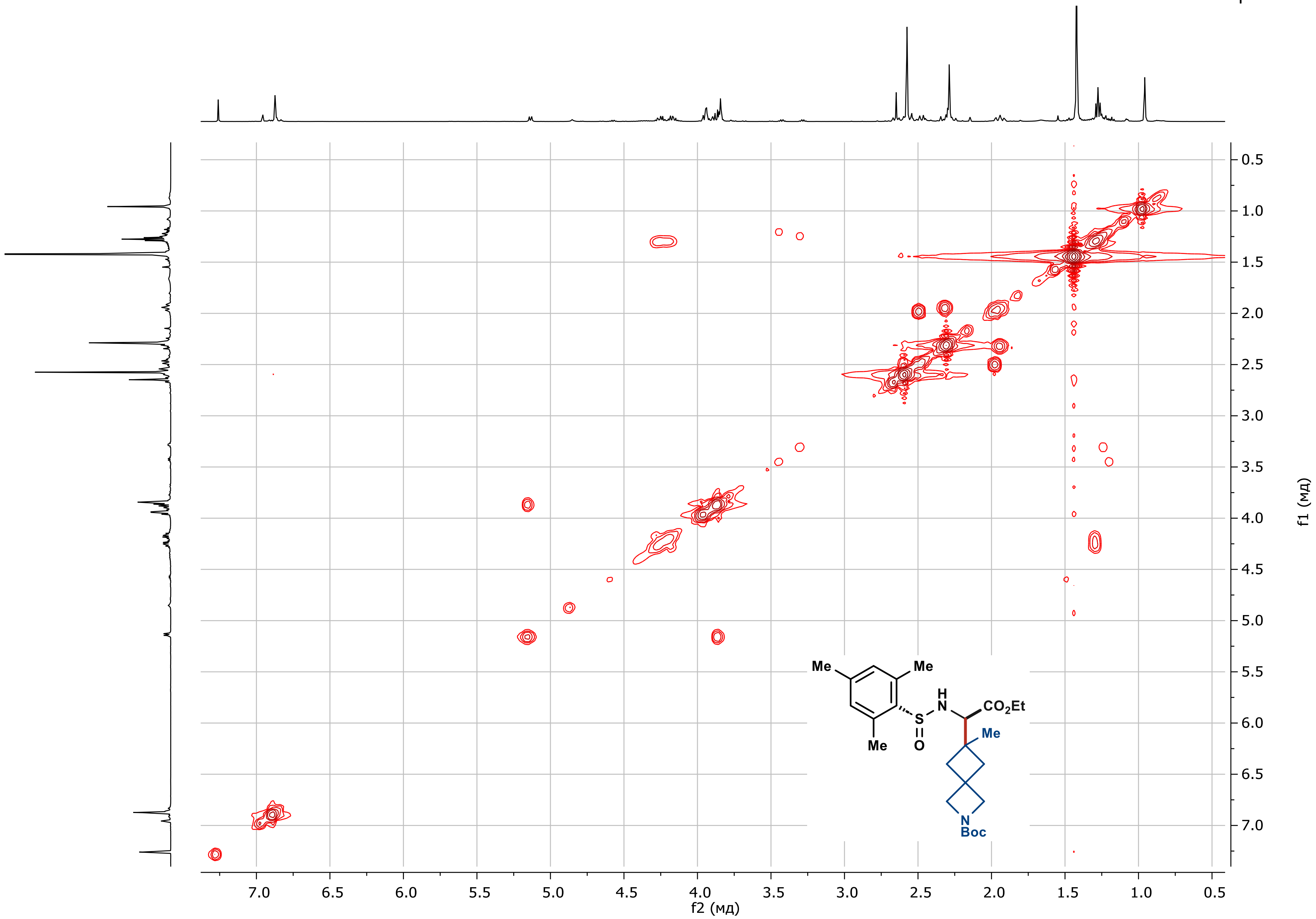

**HSQC** of compound **5i**

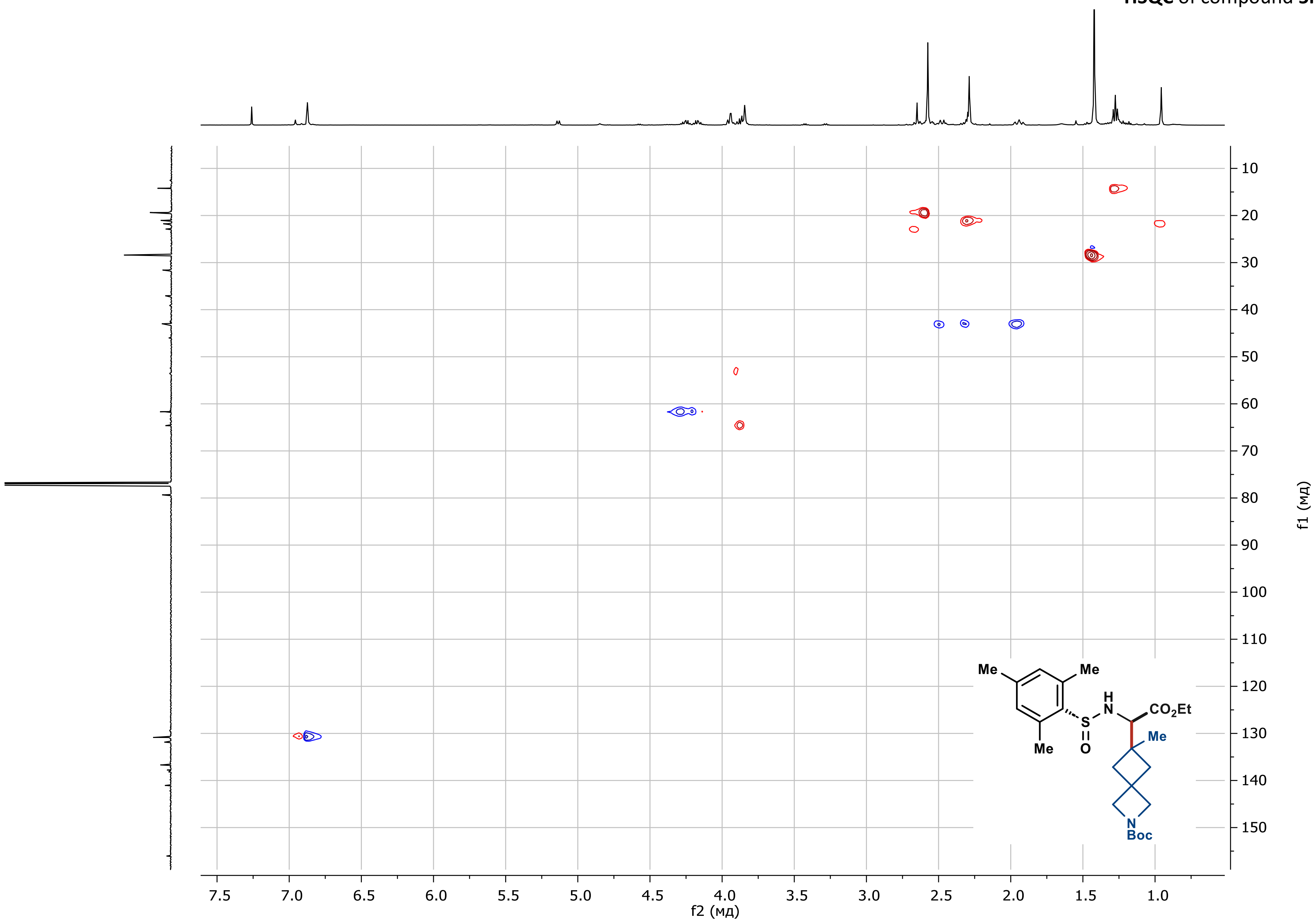

$^1$**H NMR** (500 MHz, $CDCl_3$) of compound **5j**

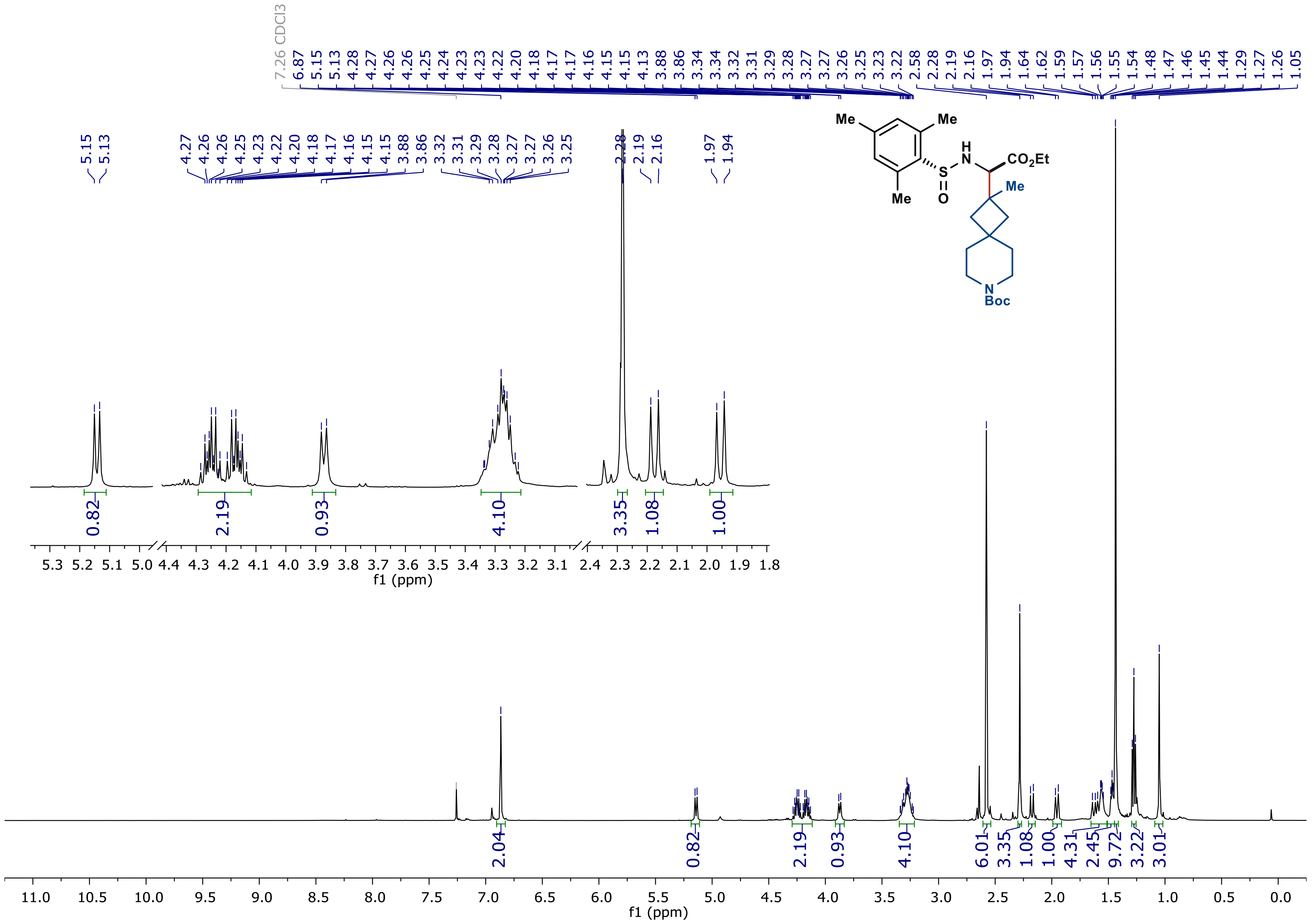

$^{13}$**C NMR** (126 MHz, $CDCl_3$) of compound **5j**

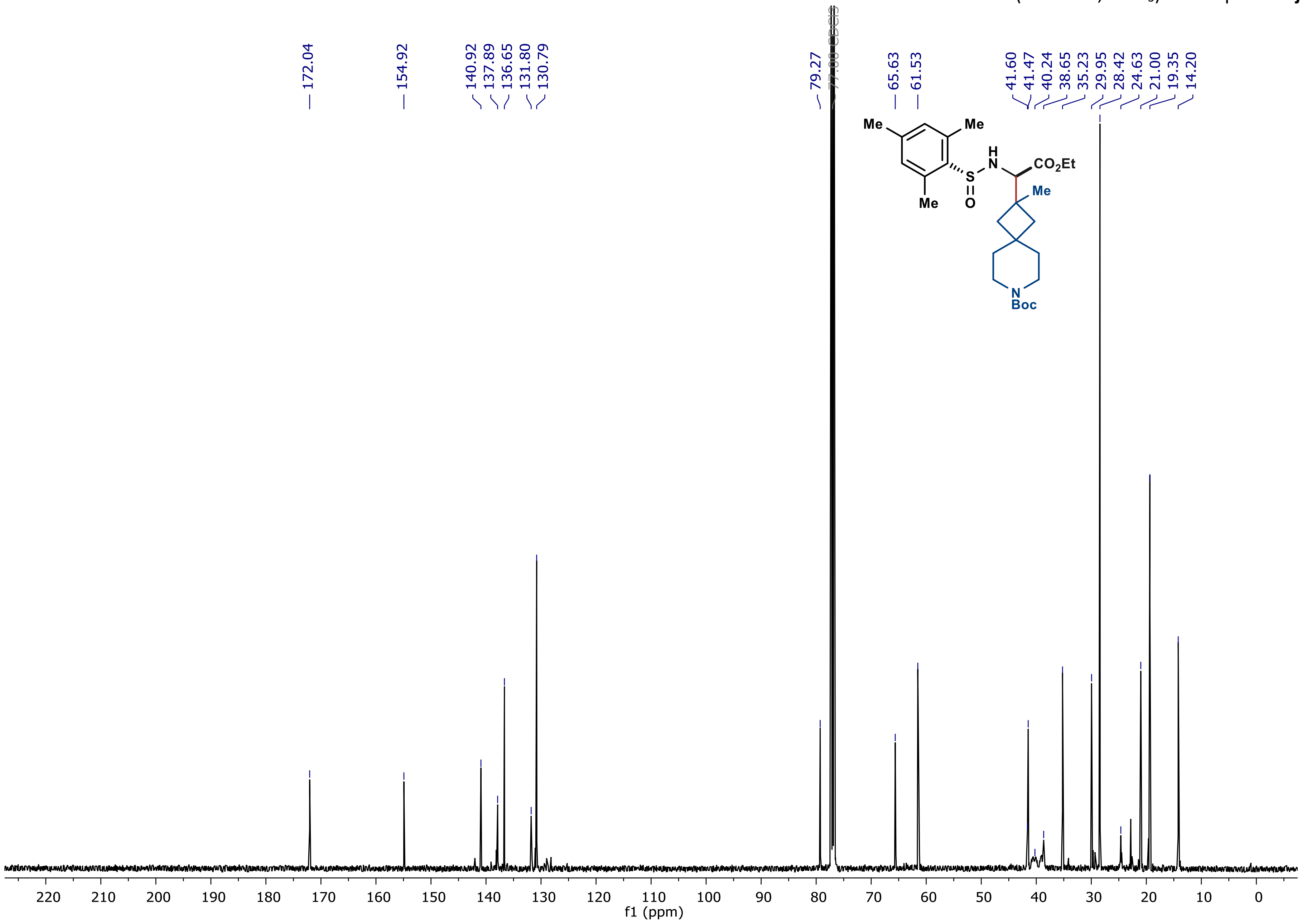

**COSY** of compound **5j**

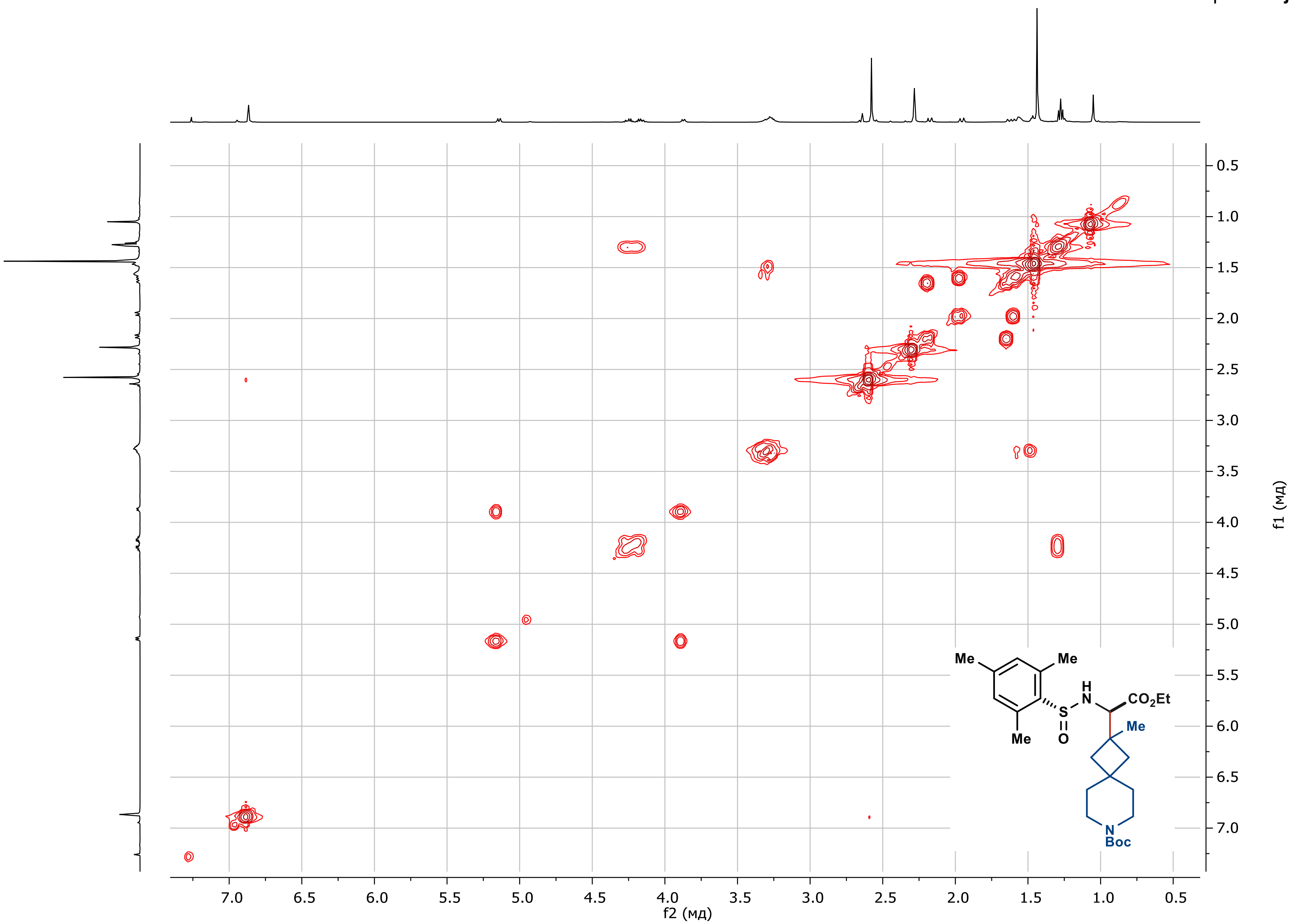

**HSQC** of compound **5j**

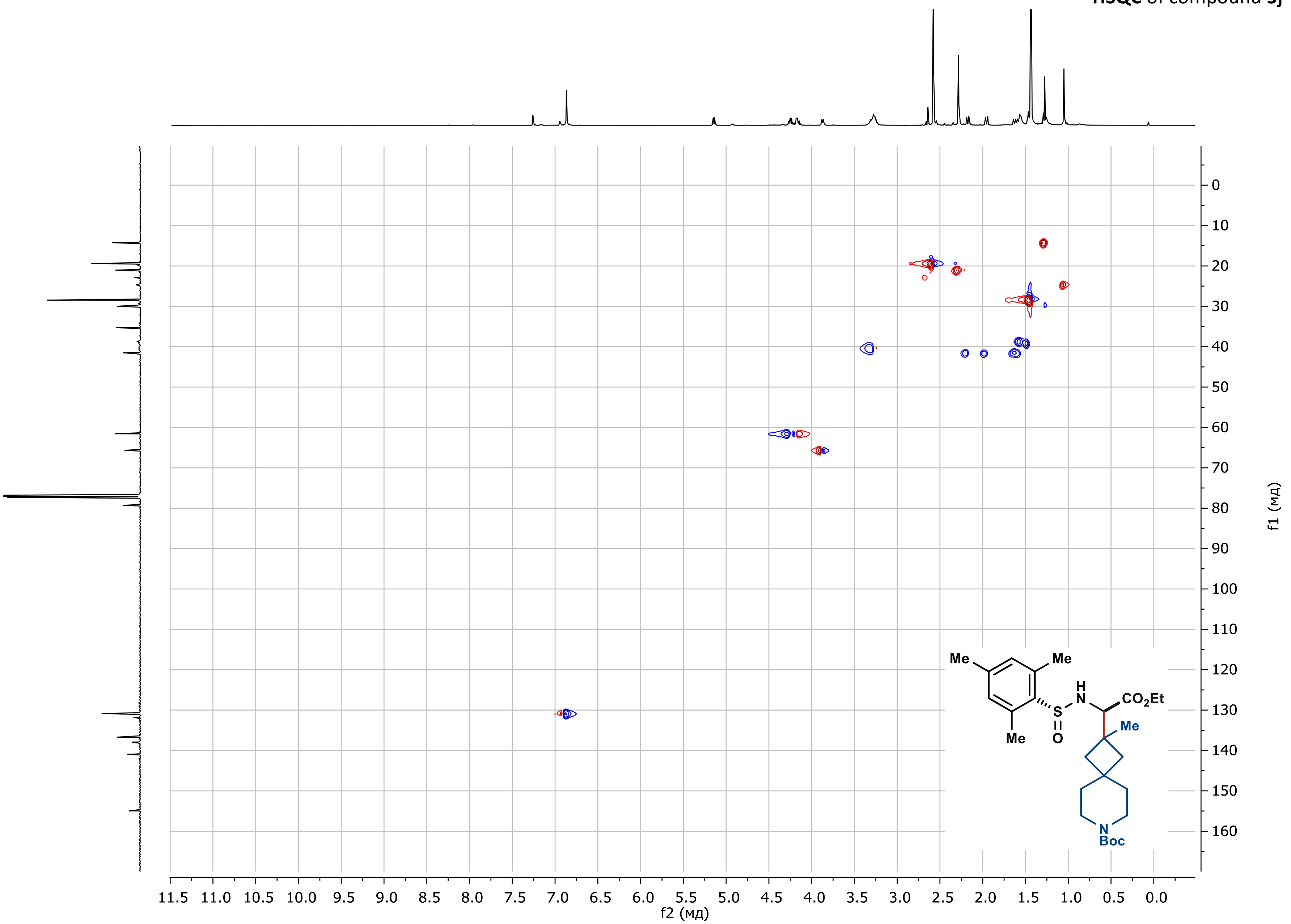

**$^1$H NMR** (500 MHz, $CDCl_3$) of compound **5k**, β-diastereomer **5k-1**

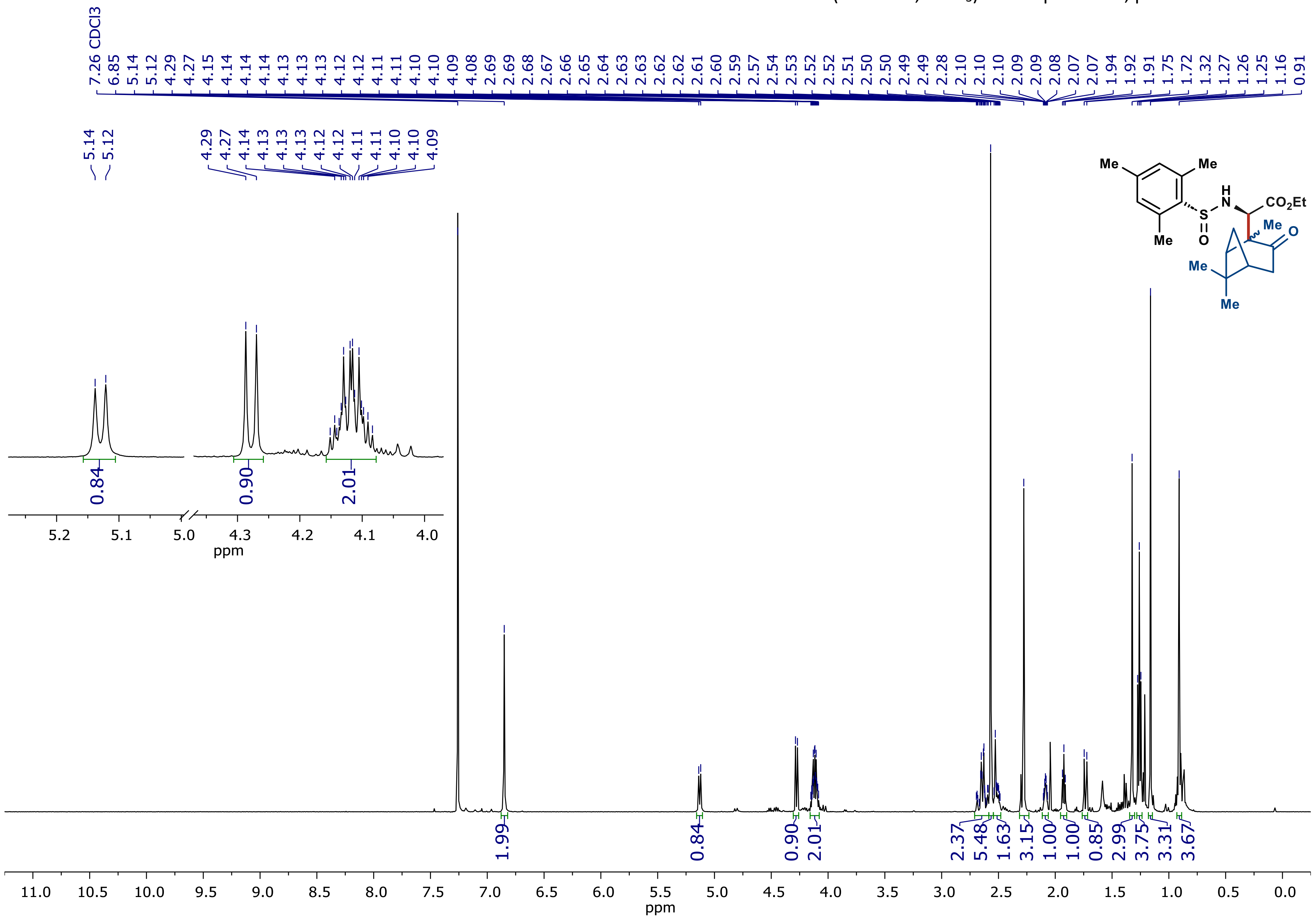

**$^{13}$C NMR** (126 MHz, $CDCl_3$) of compound **5k**, β-diastereomer **5k-1**

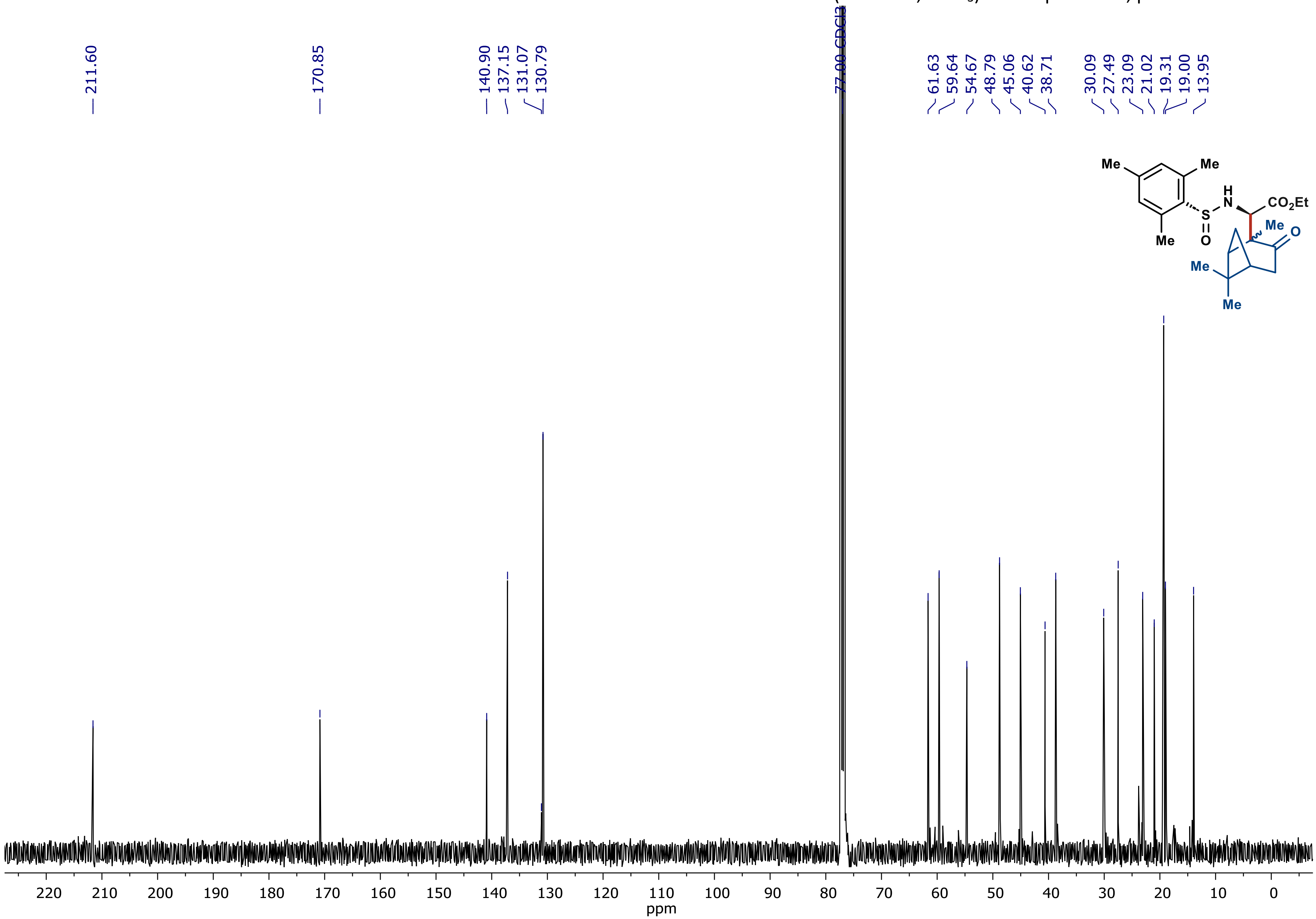

**COSY** of compound **5k**, β-diastereomer **5k-1**

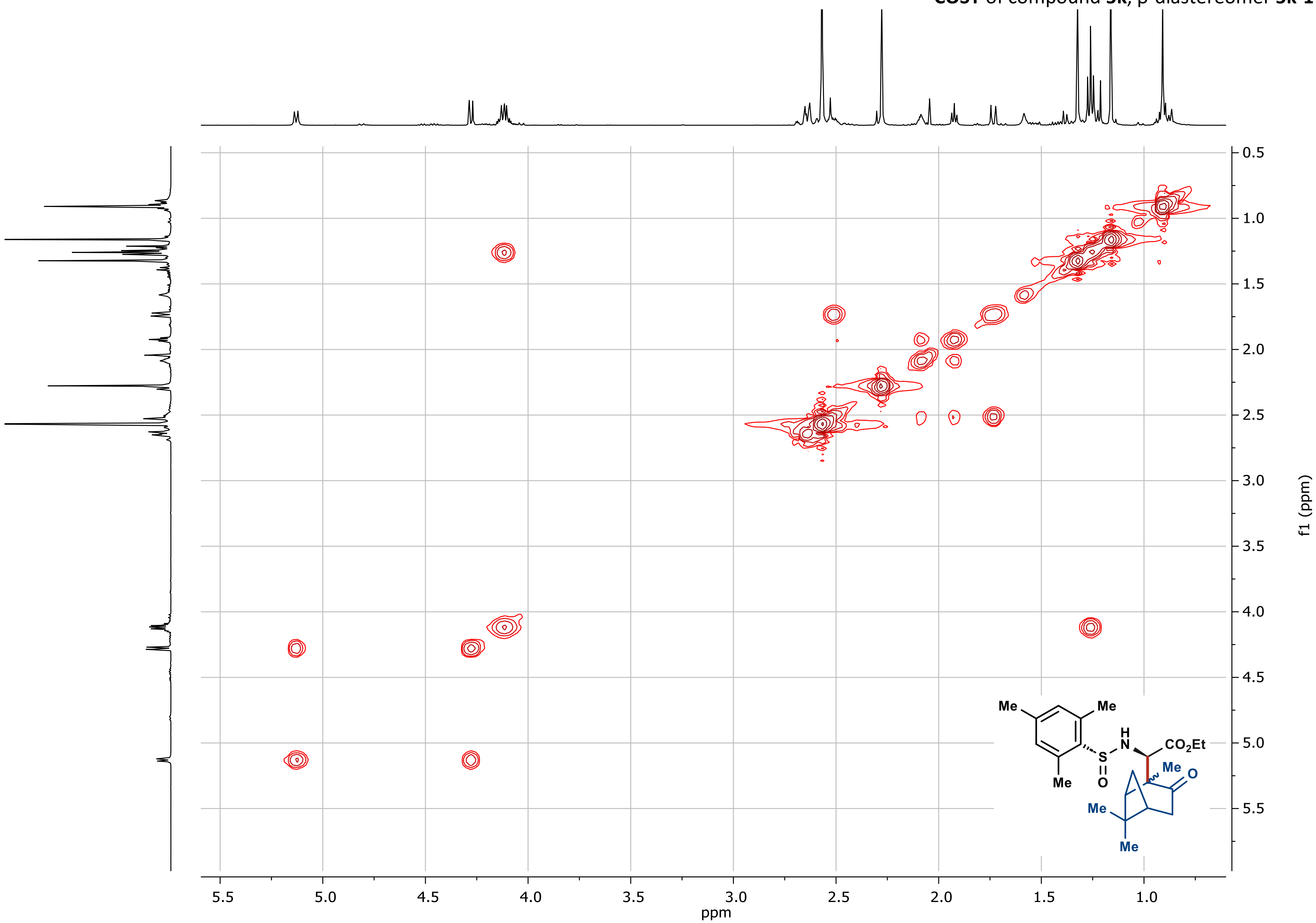

**HSQC** of compound **5k**, β-diastereomer **5k-1**

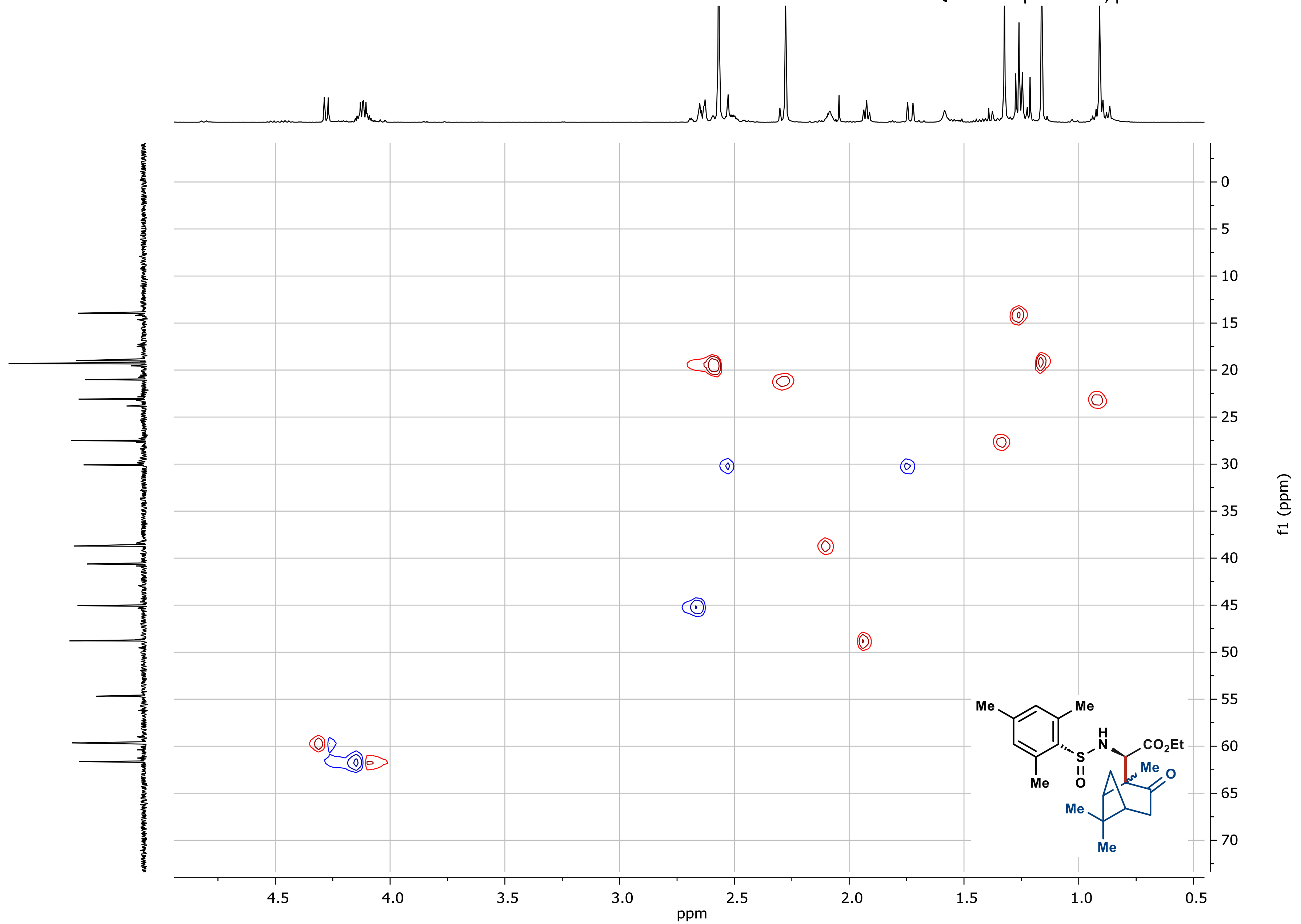

**HMBC** of compound **5k**, β-diastereomer **5k-1**

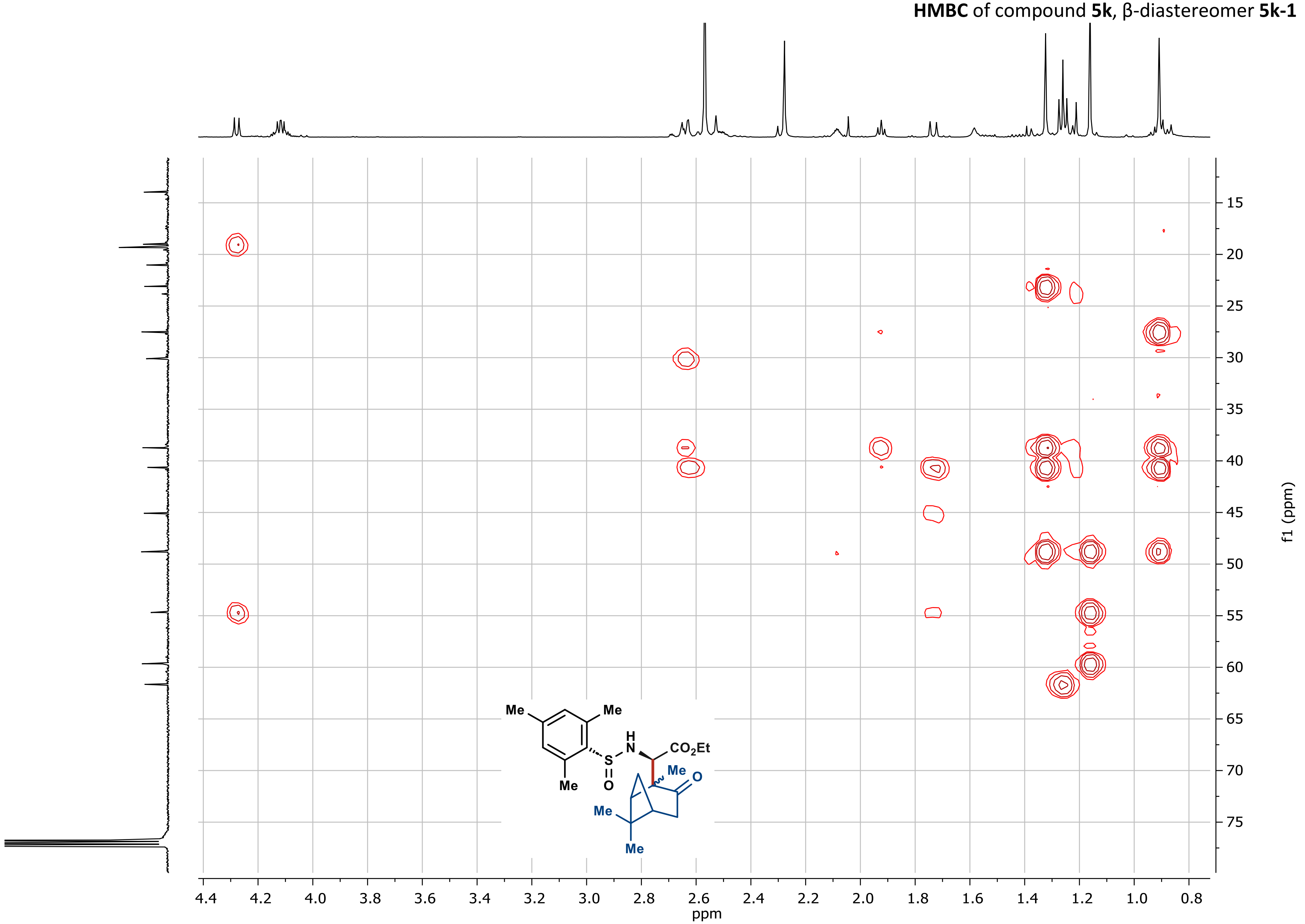

**$^{1}$H NMR** (500 MHz, $CDCl_3$) of compound **5k**, β-diastereomer **5k-2**

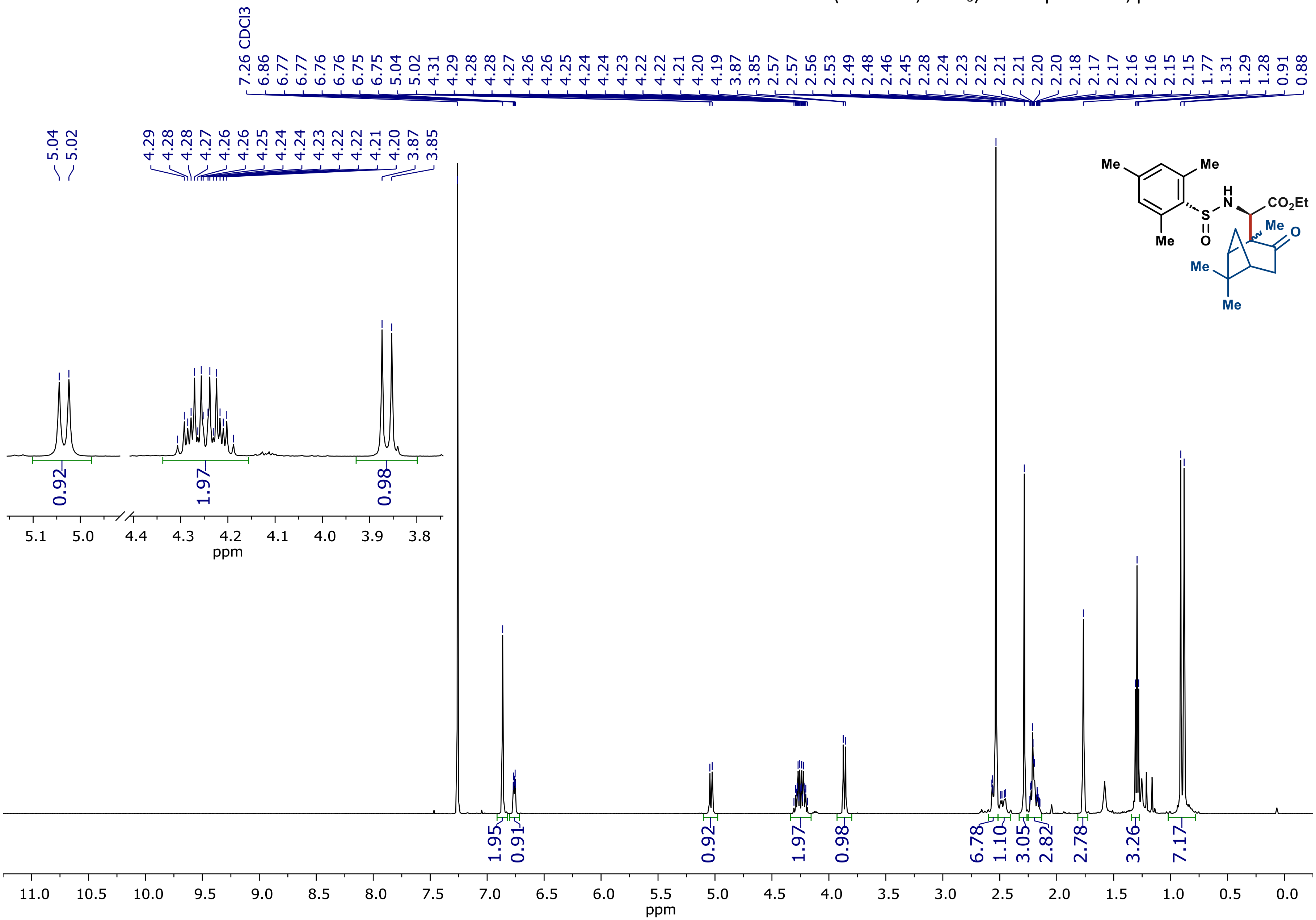

**$^{13}$C NMR** (126 MHz, $CDCl_3$) of compound **5k**, β-diastereomer **5k-2**

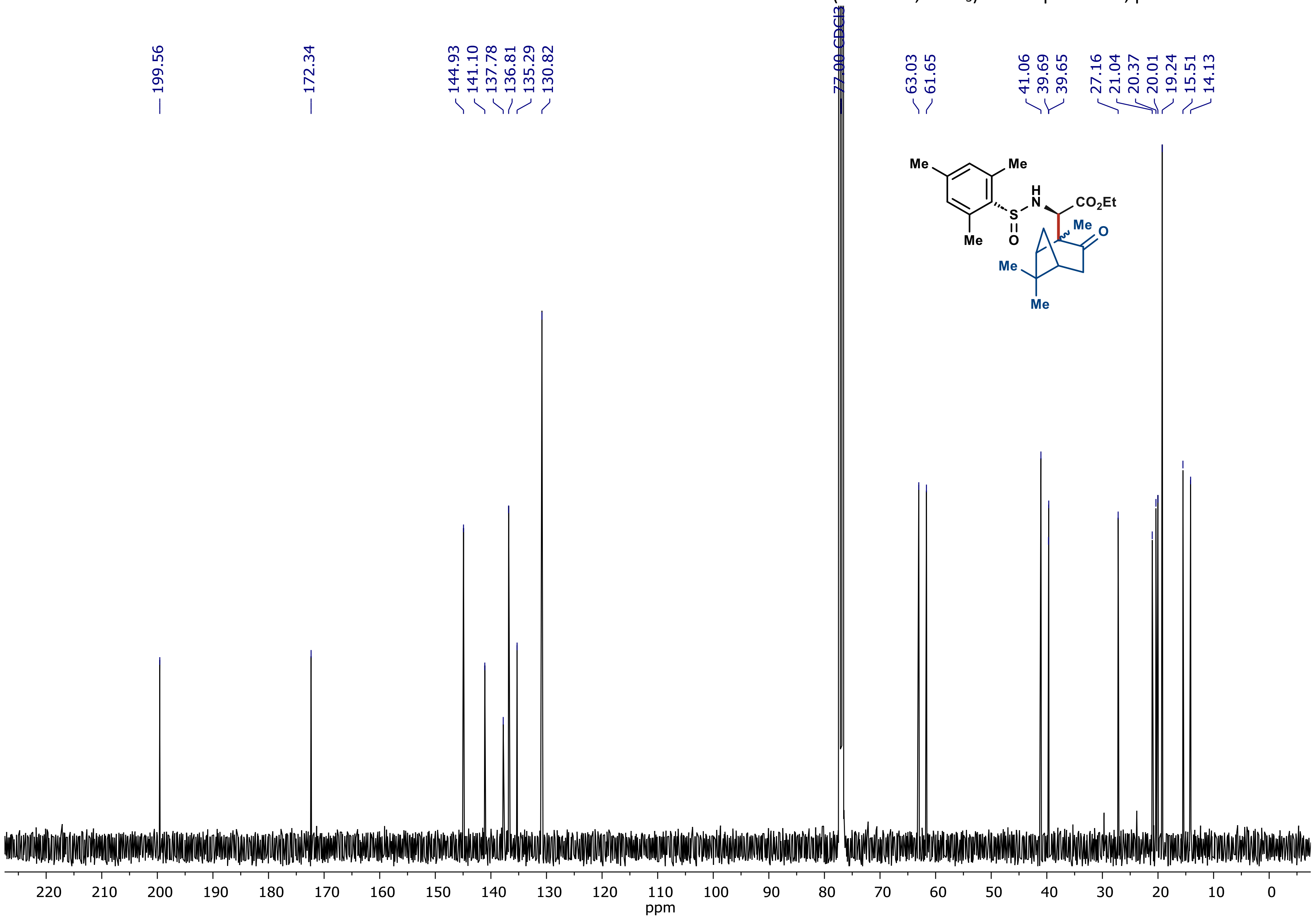

**COSY** of compound **5k**, β-diastereomer **5k-2**

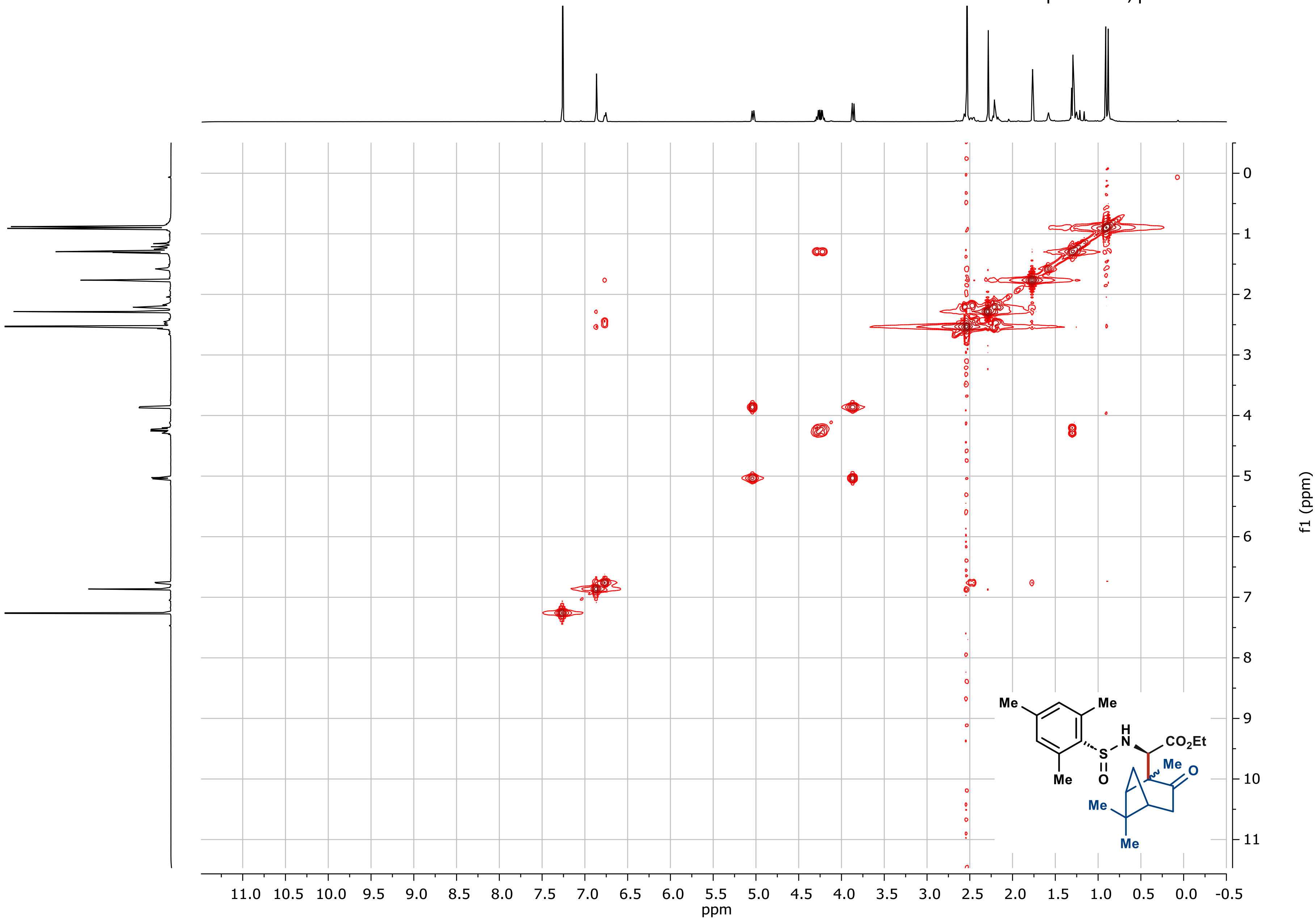

**HSQC** of compound **5k**, β-diastereomer **5k-2**

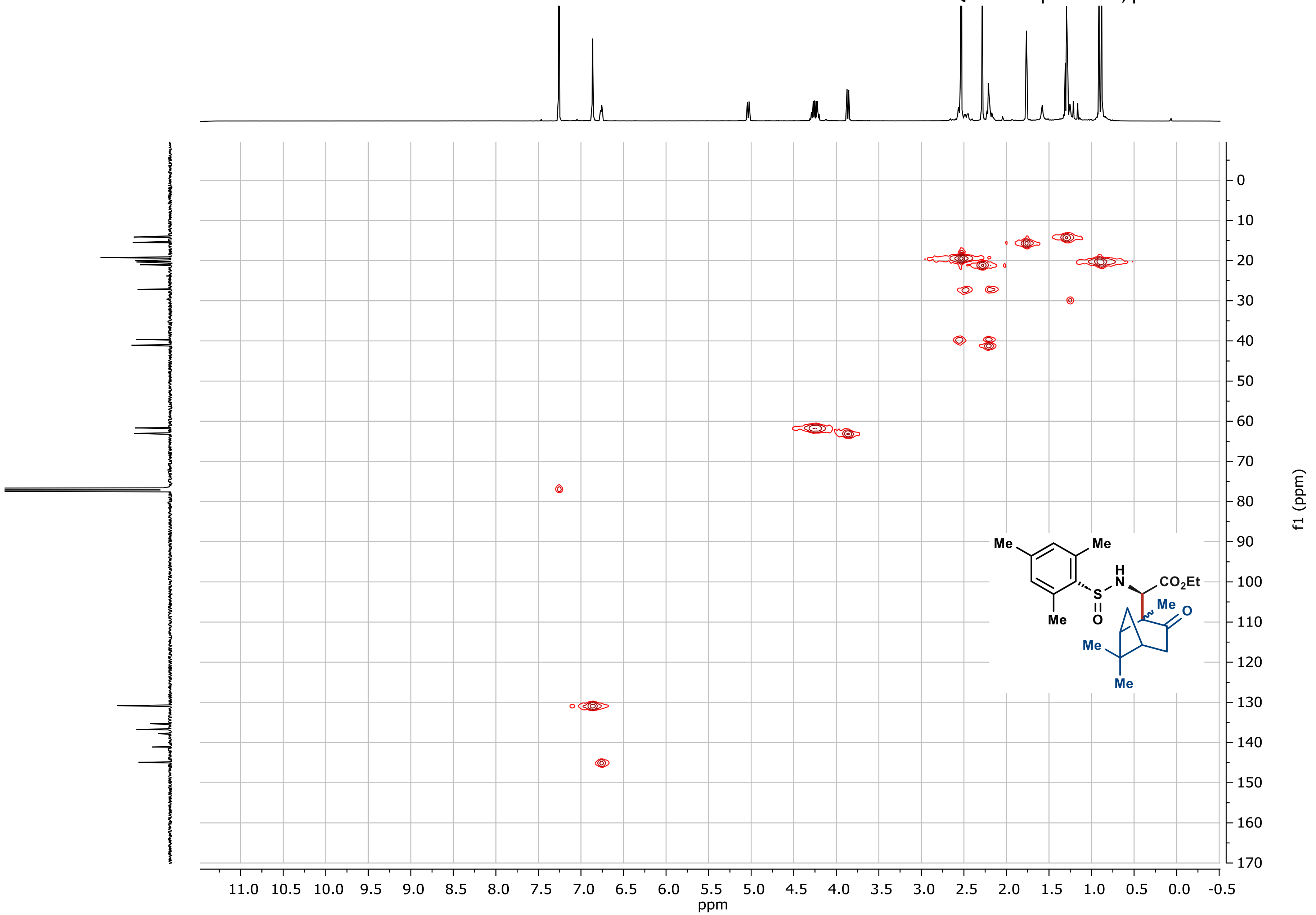

**HMBC** of compound **5k**, β-diastereomer **5k-2**

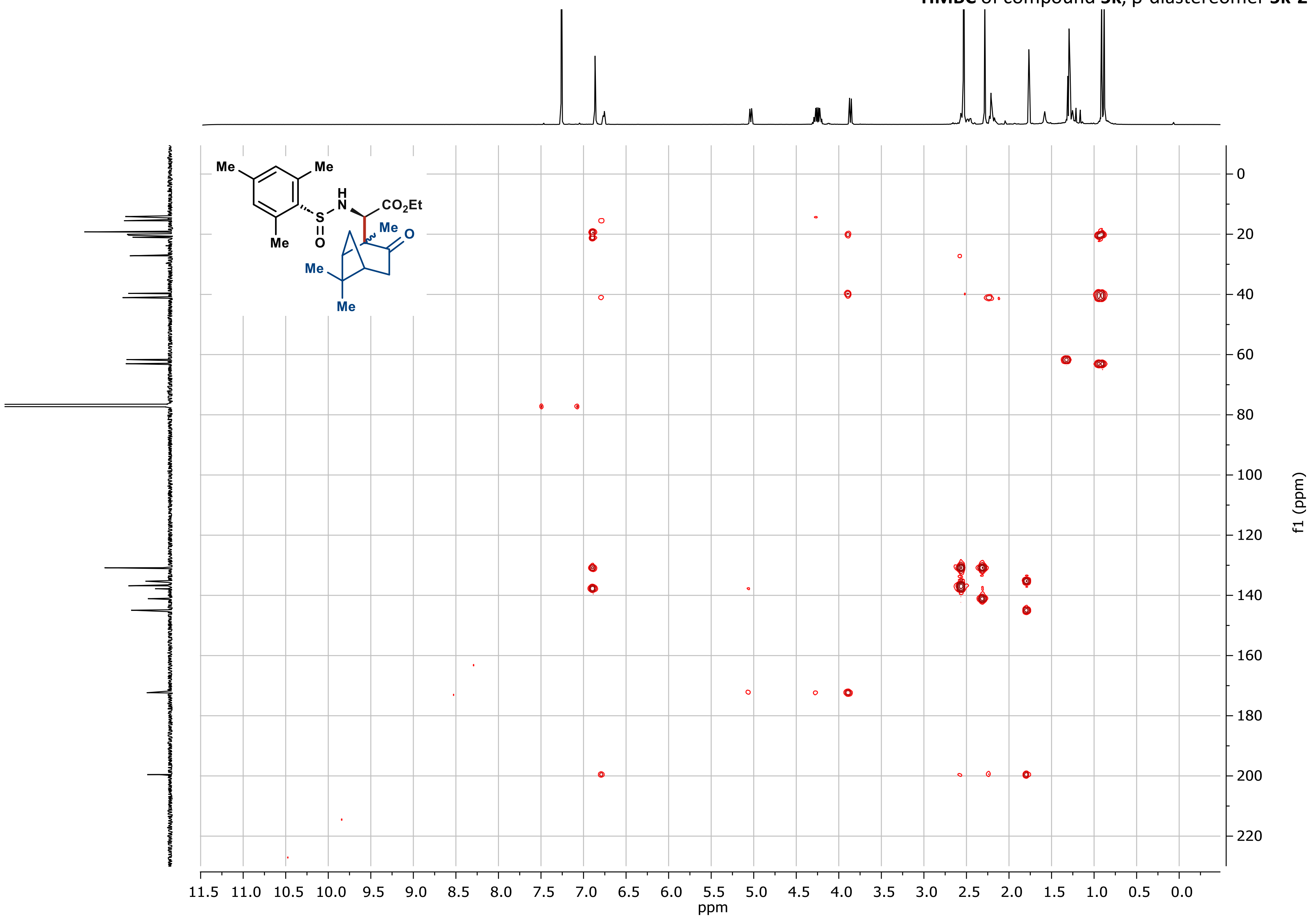

**$^{1}$H NMR** (500 MHz, $CDCl_3$) of compound **5l**

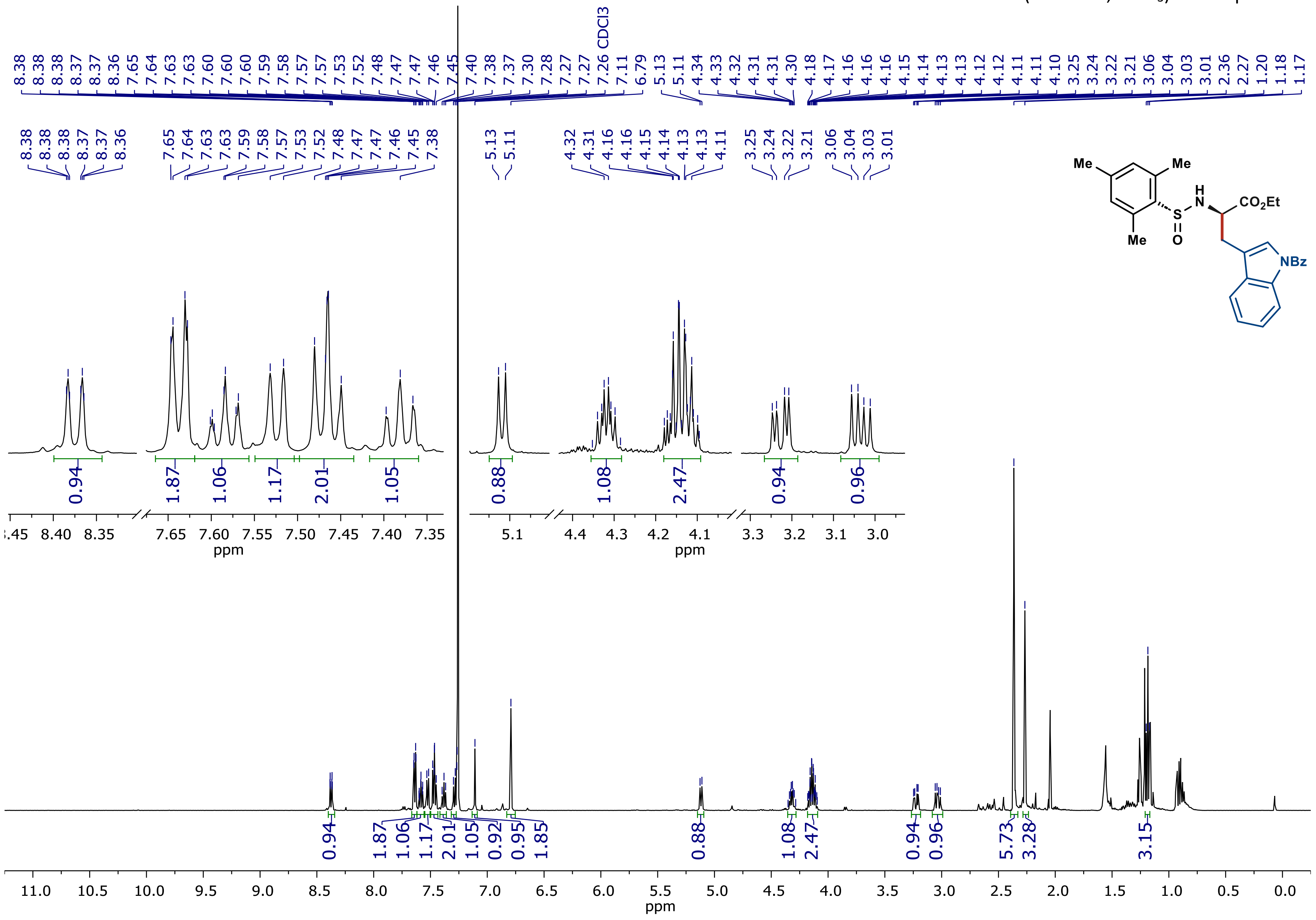

**$^{13}$C NMR** (126 MHz, $CDCl_3$) of compound **5l**

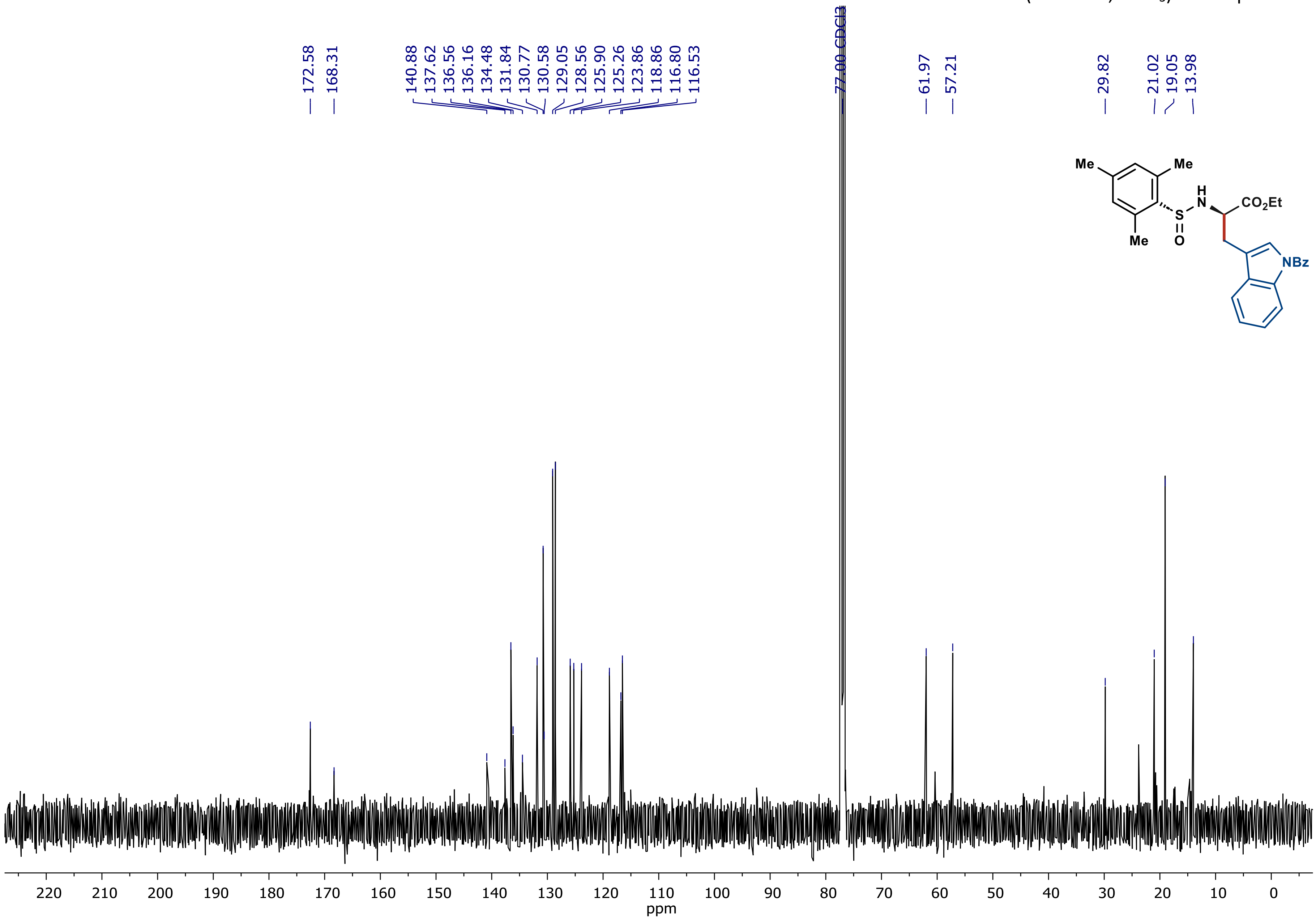

**COSY** of compound **5l**

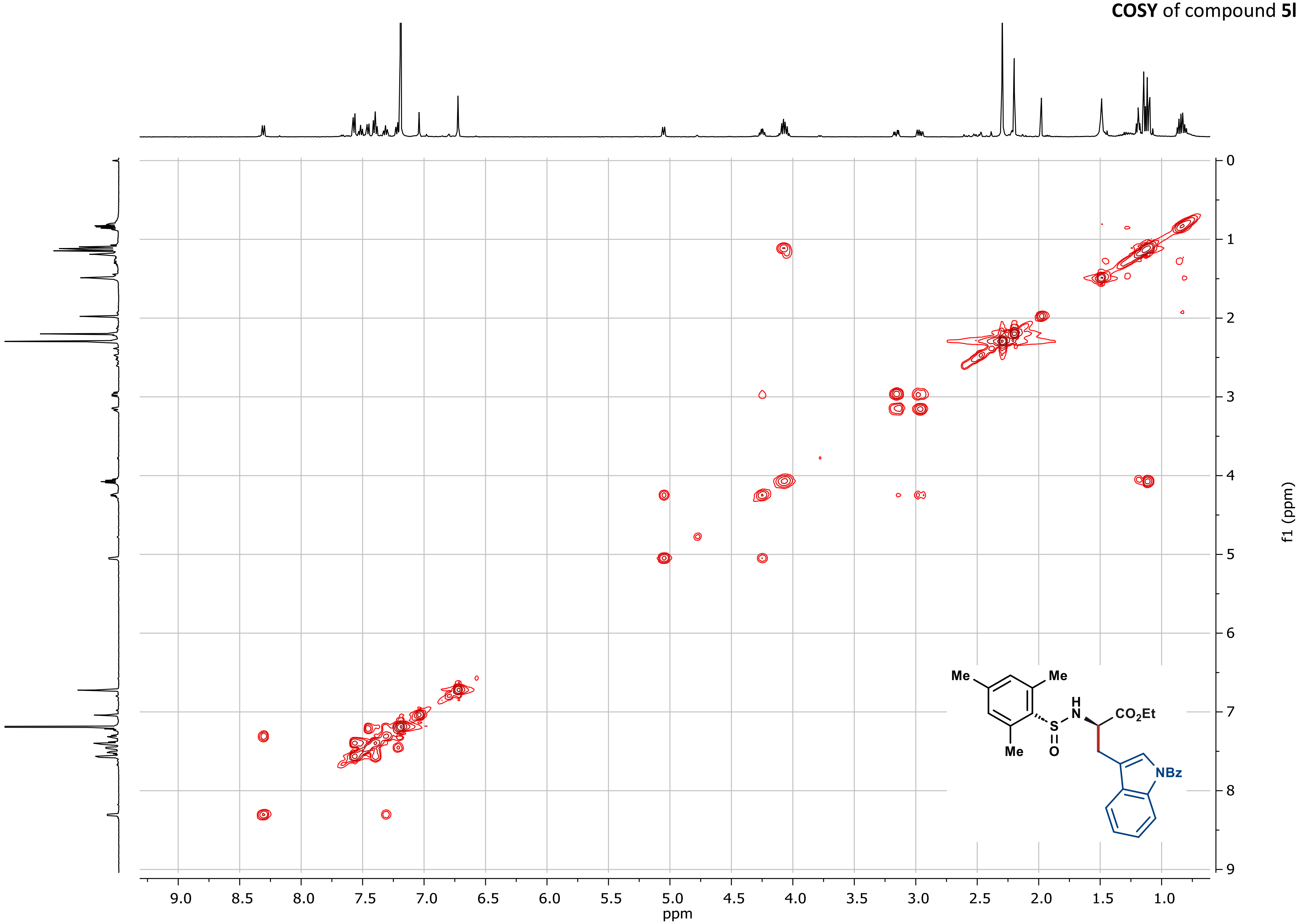

**HSQC** of compound **5l**

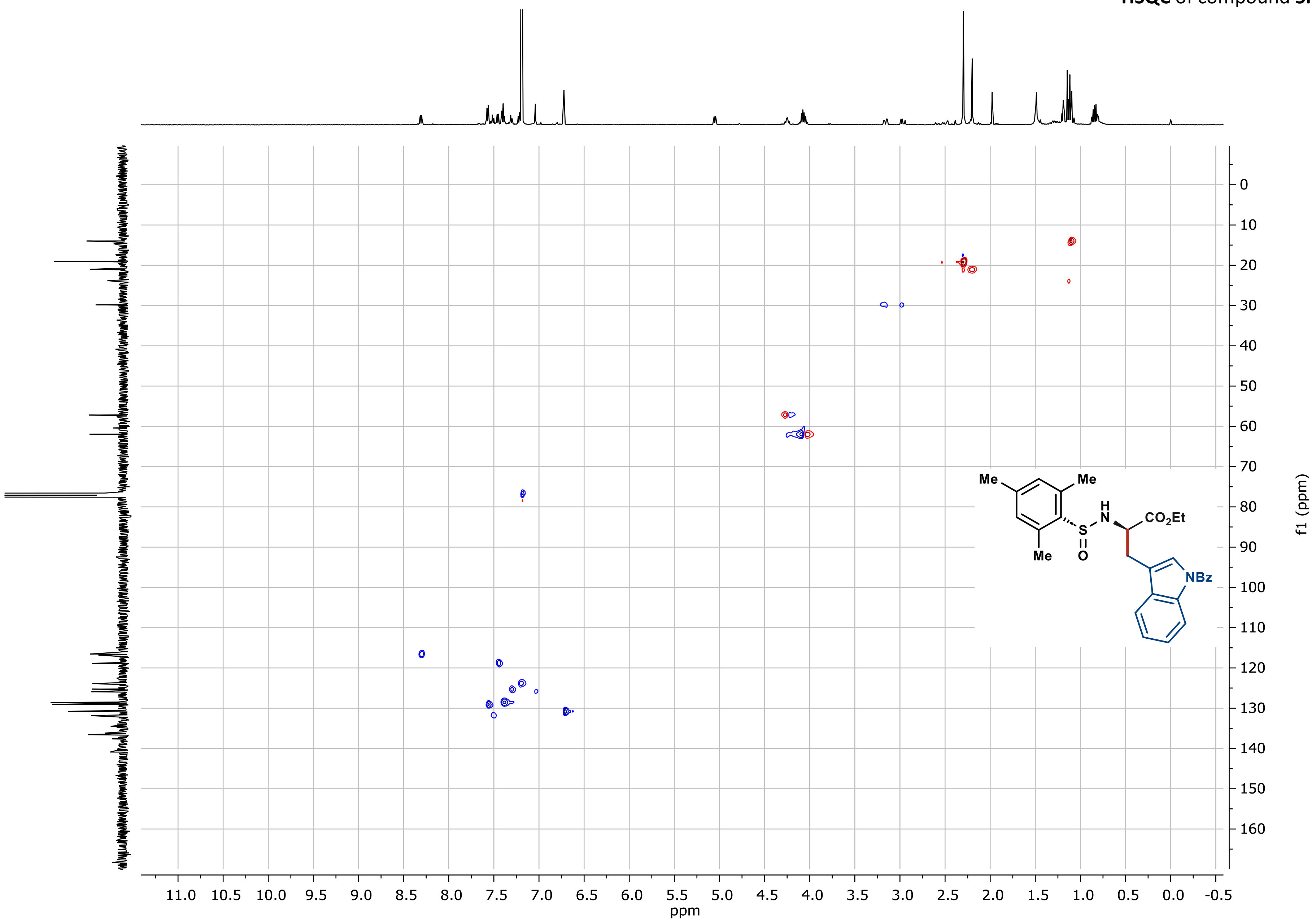

**HMBC** of compound **5l**

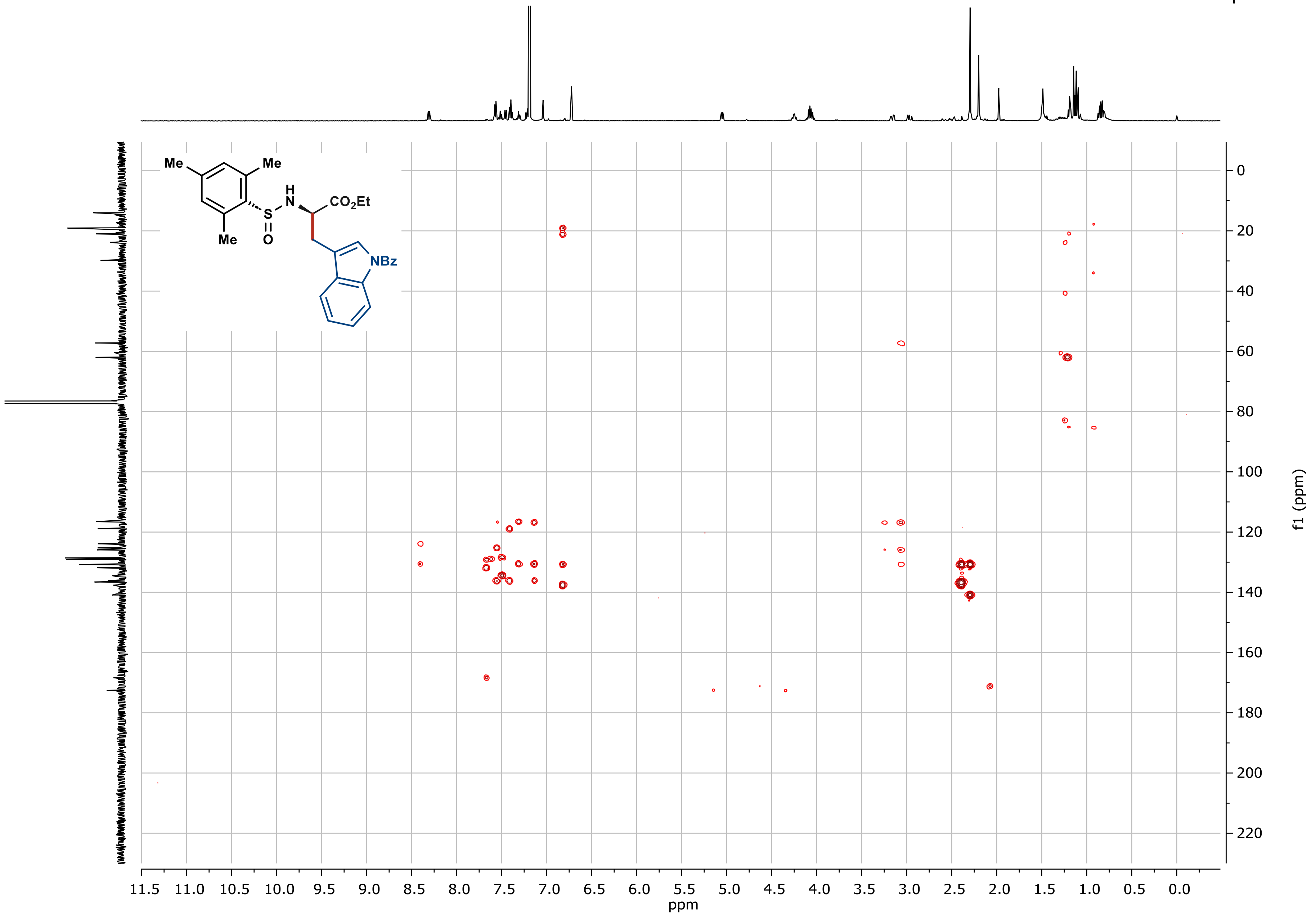

[1]**H NMR** (500 MHz, $CDCl_3$) of compound **5m**

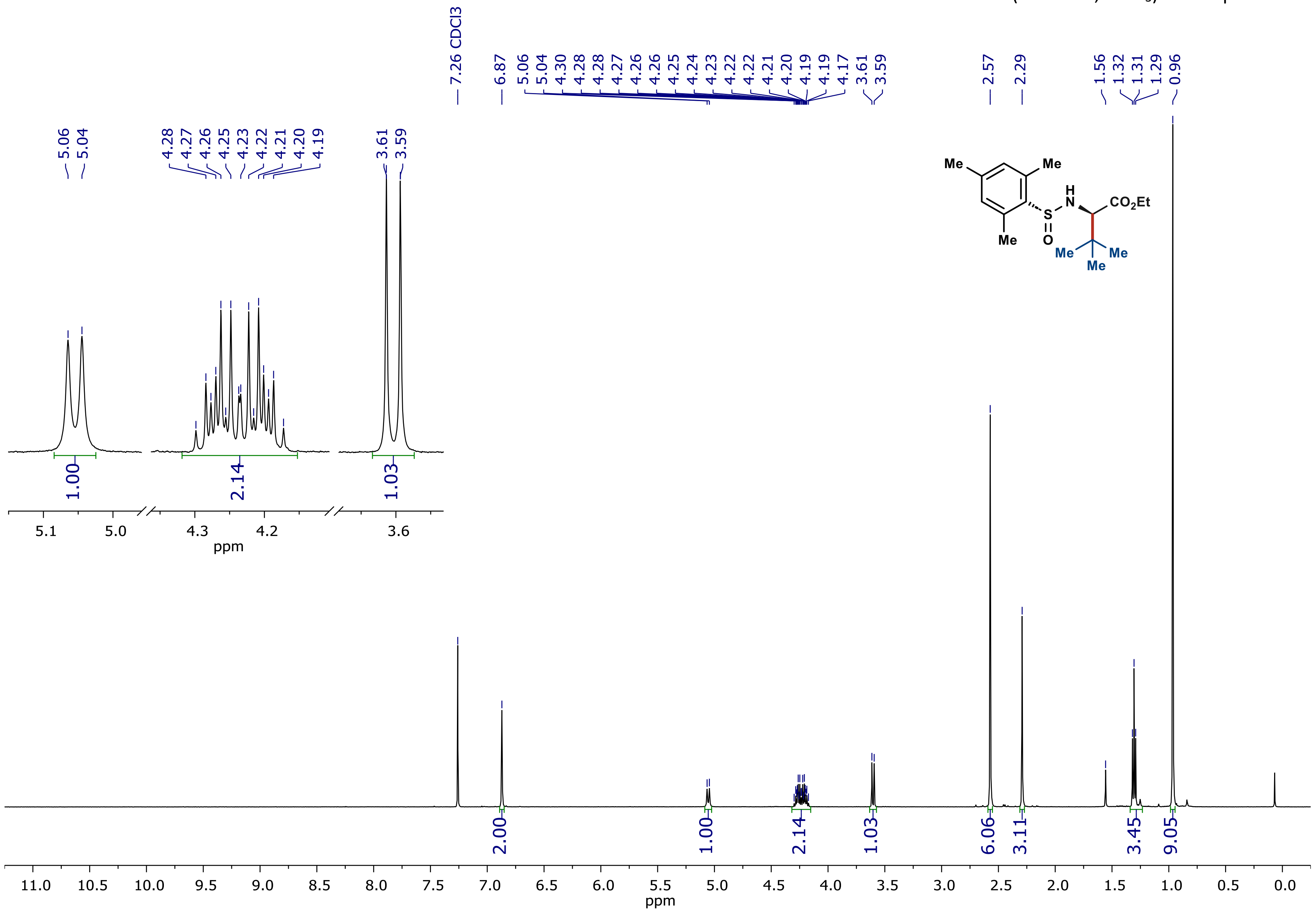

<sup></sup>

$^{13}$**C NMR** (126 MHz, $CDCl_3$) of compound **5m**

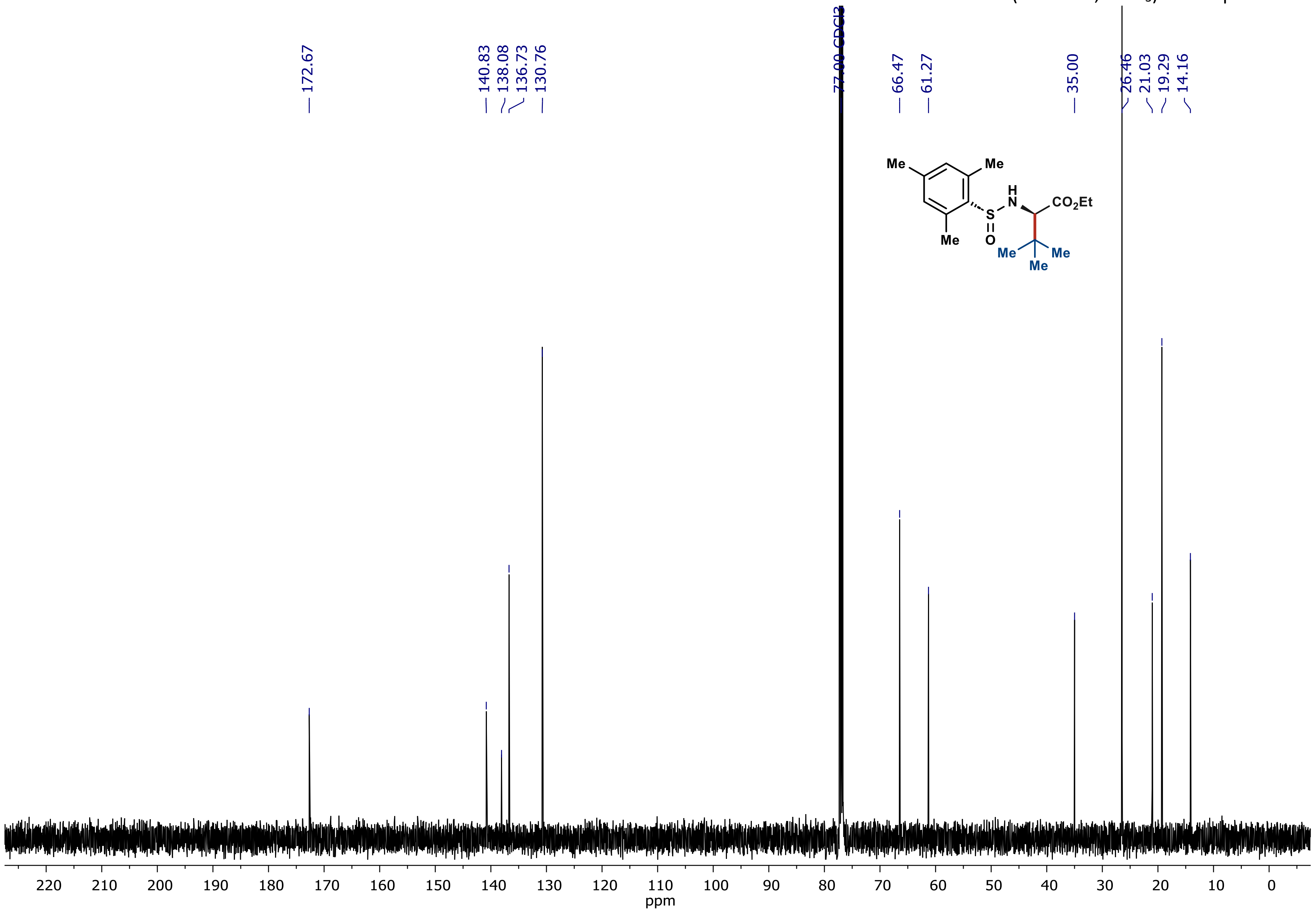

**$^{1}$H NMR** (500 MHz, $CDCl_3$) of compound **5n**

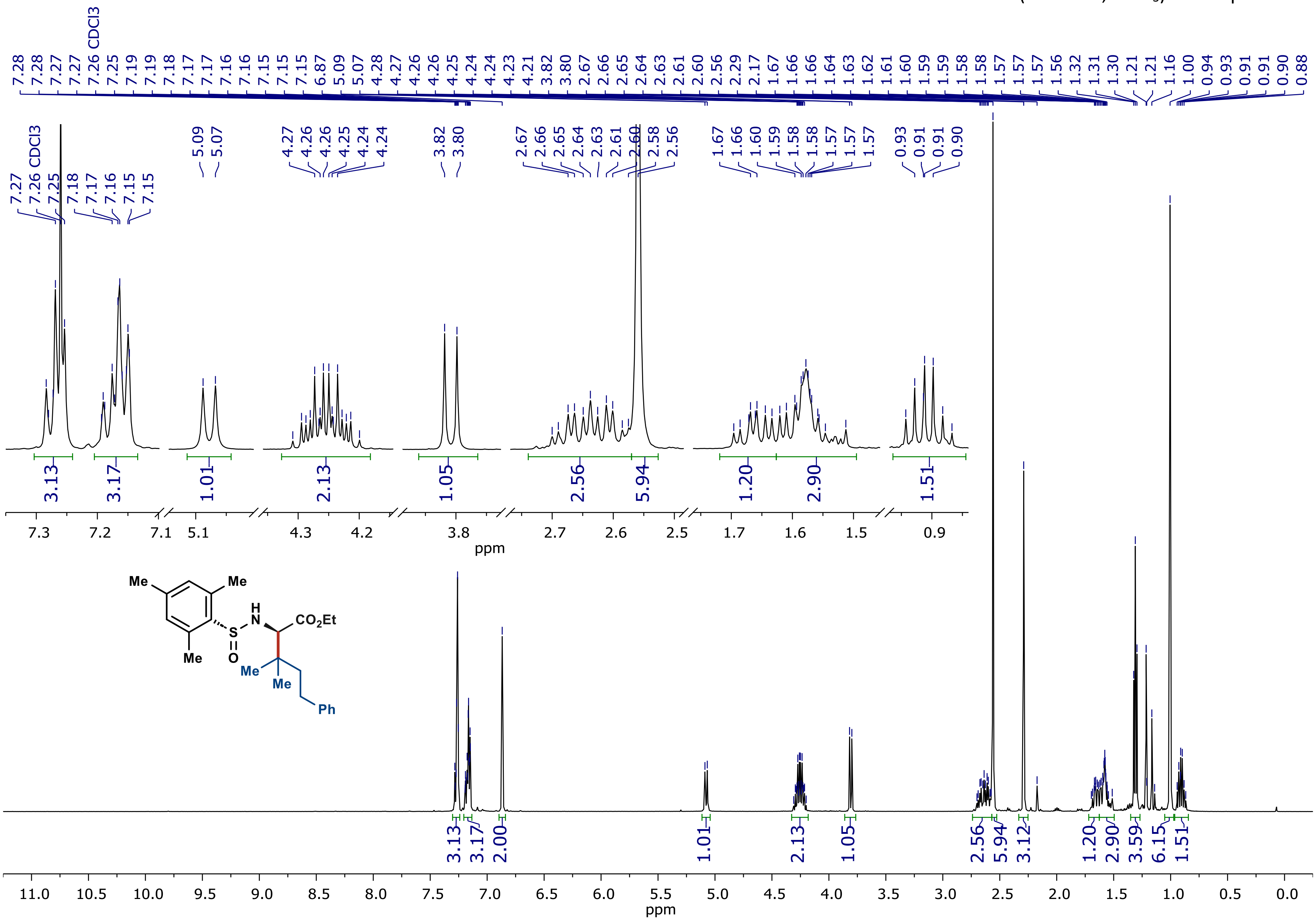

$^{13}$**C NMR** (126 MHz, $CDCl_3$) of compound **5n**

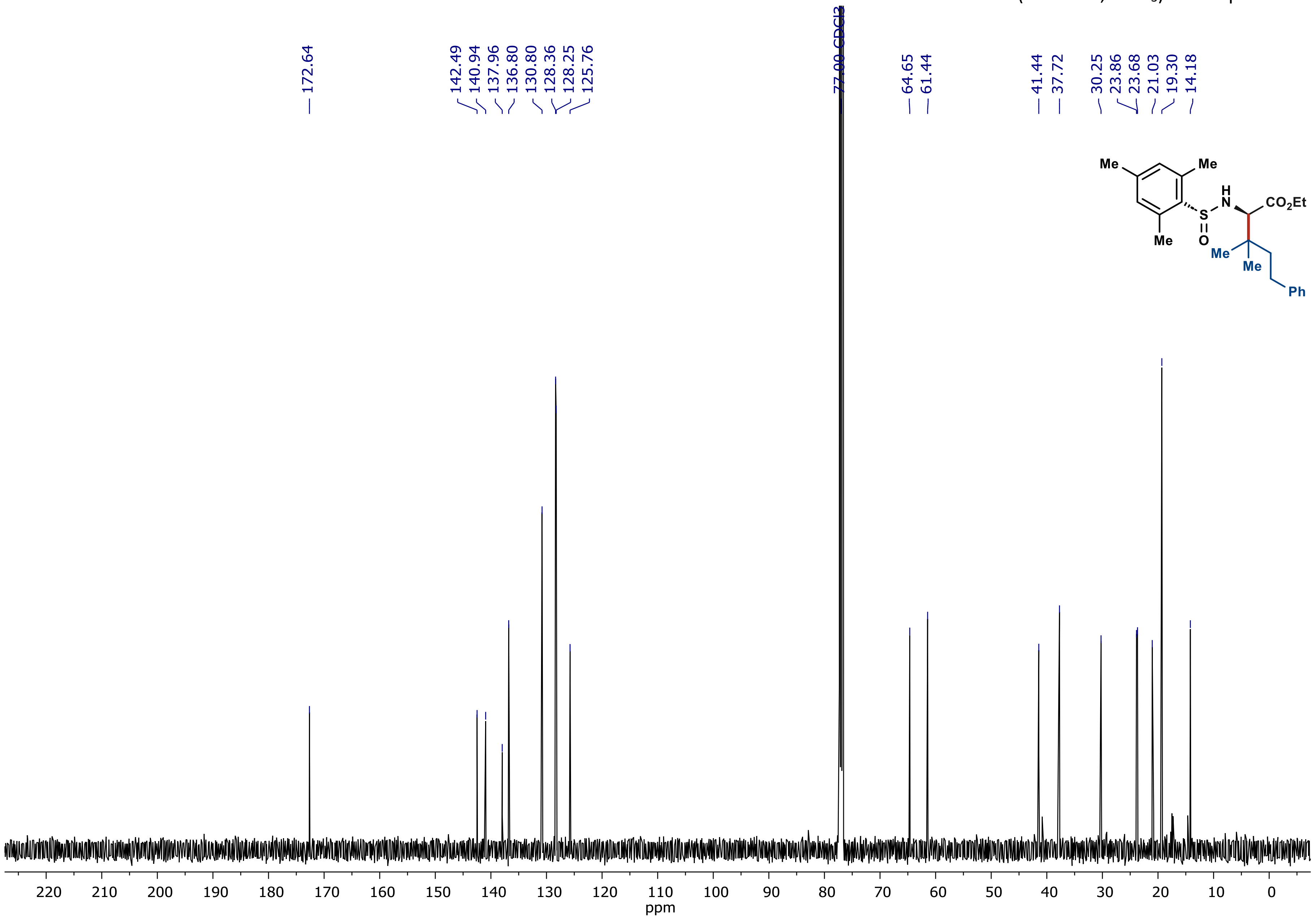

**COSY** of compound **5n**

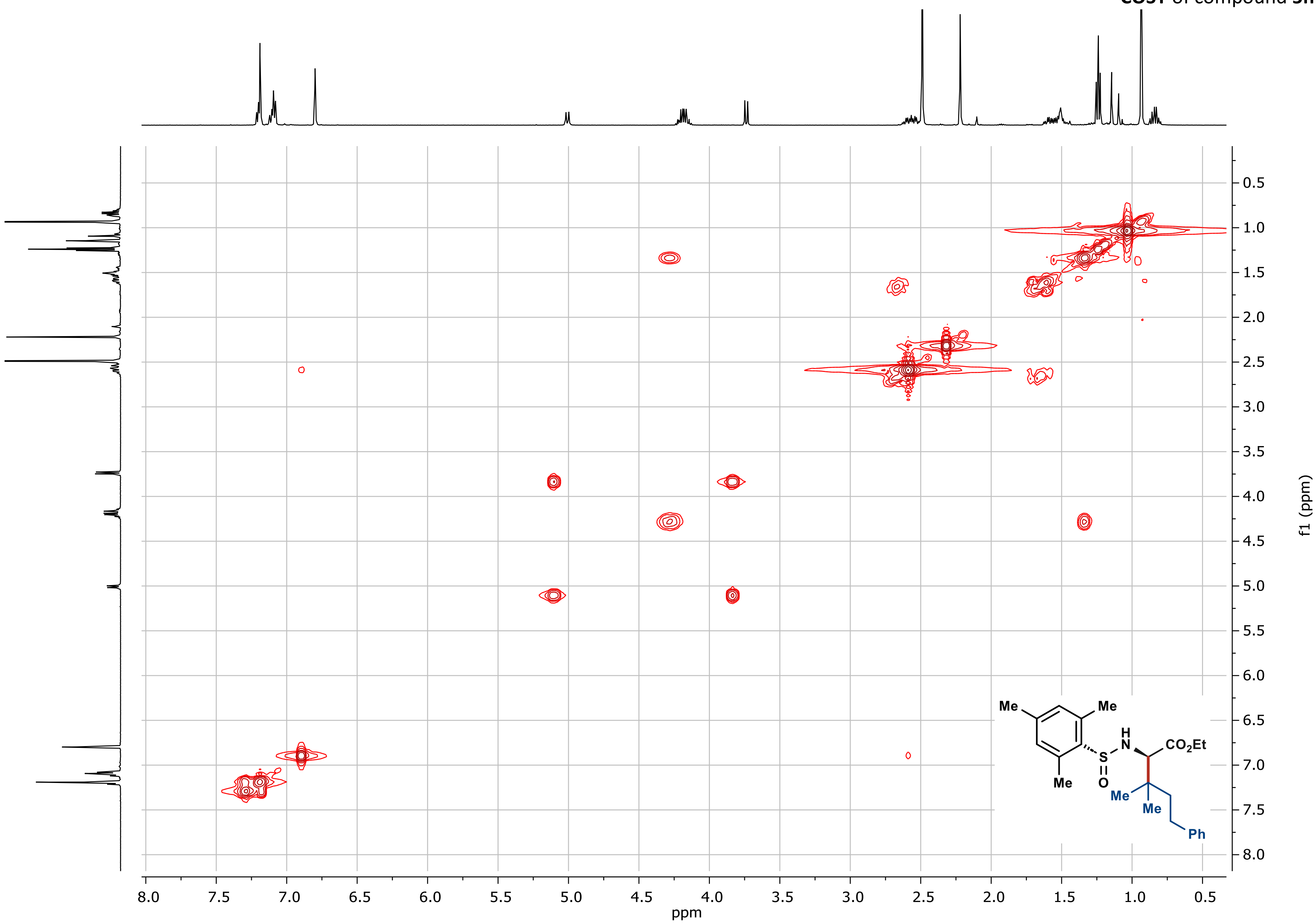

**HSQC** of compound **5n**

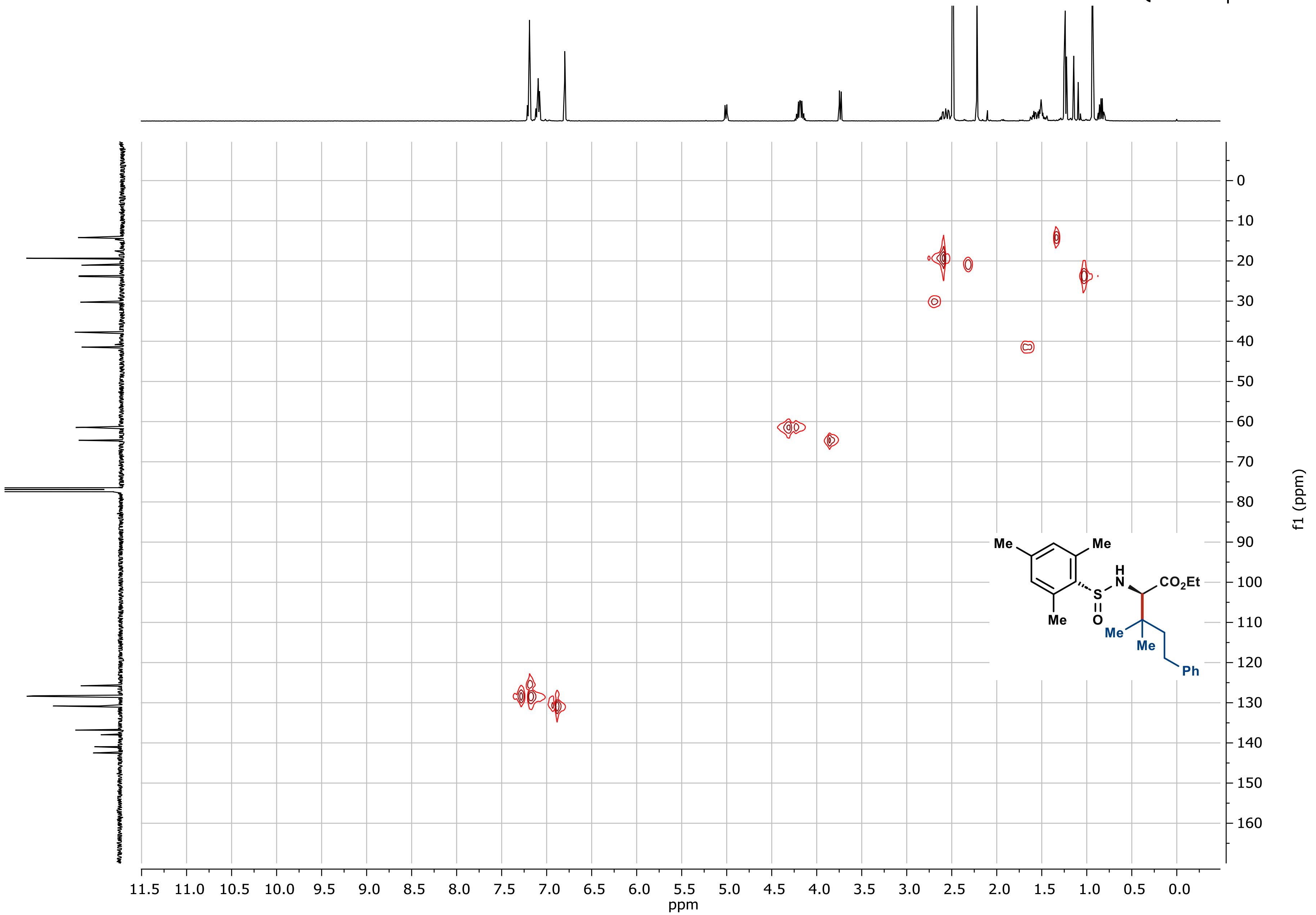

**$^1$H NMR** (500 MHz, $CDCl_3$) of compound **5o**

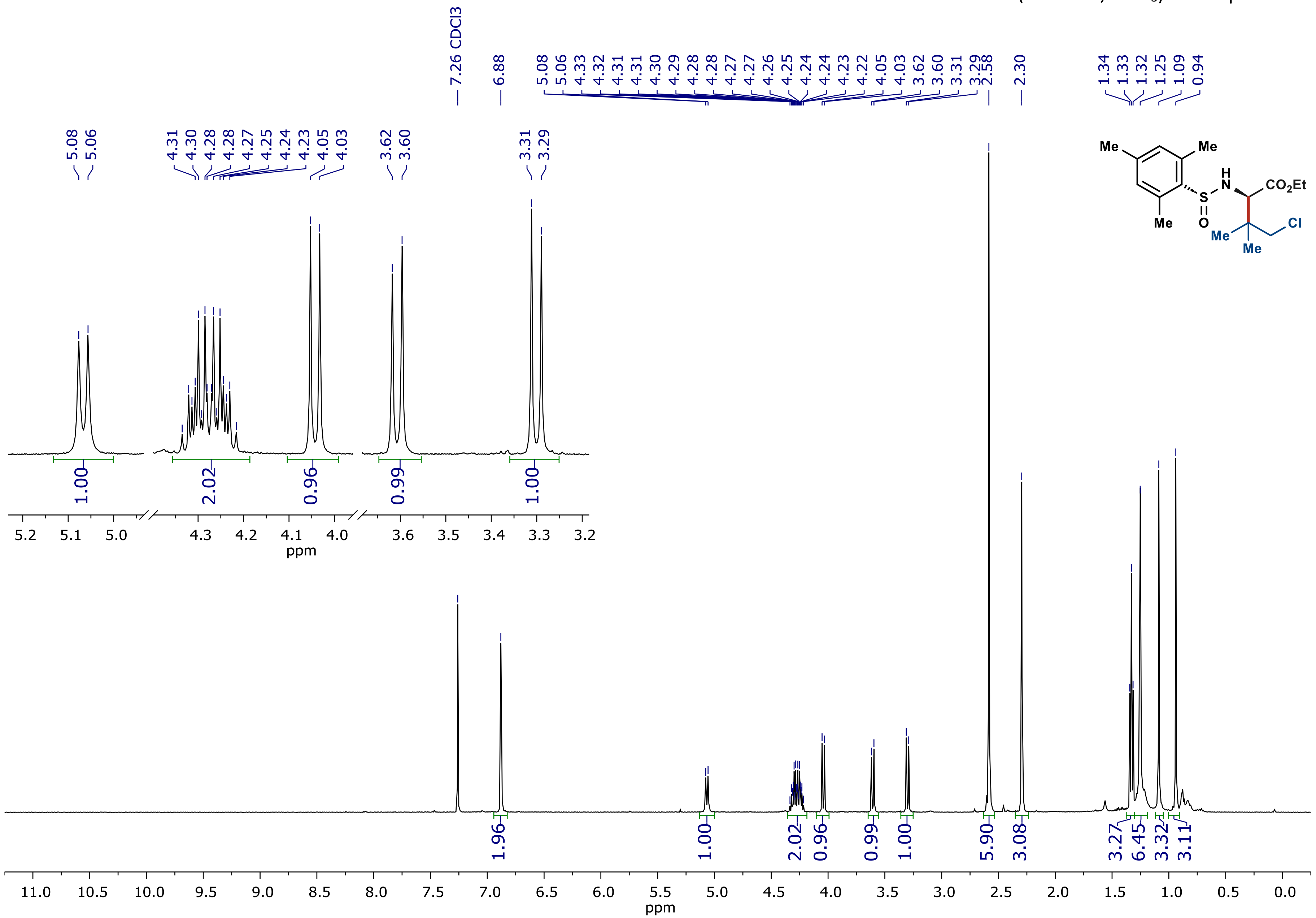

**$^{13}C$ NMR** (126 MHz, $CDCl_3$) of compound **5o**

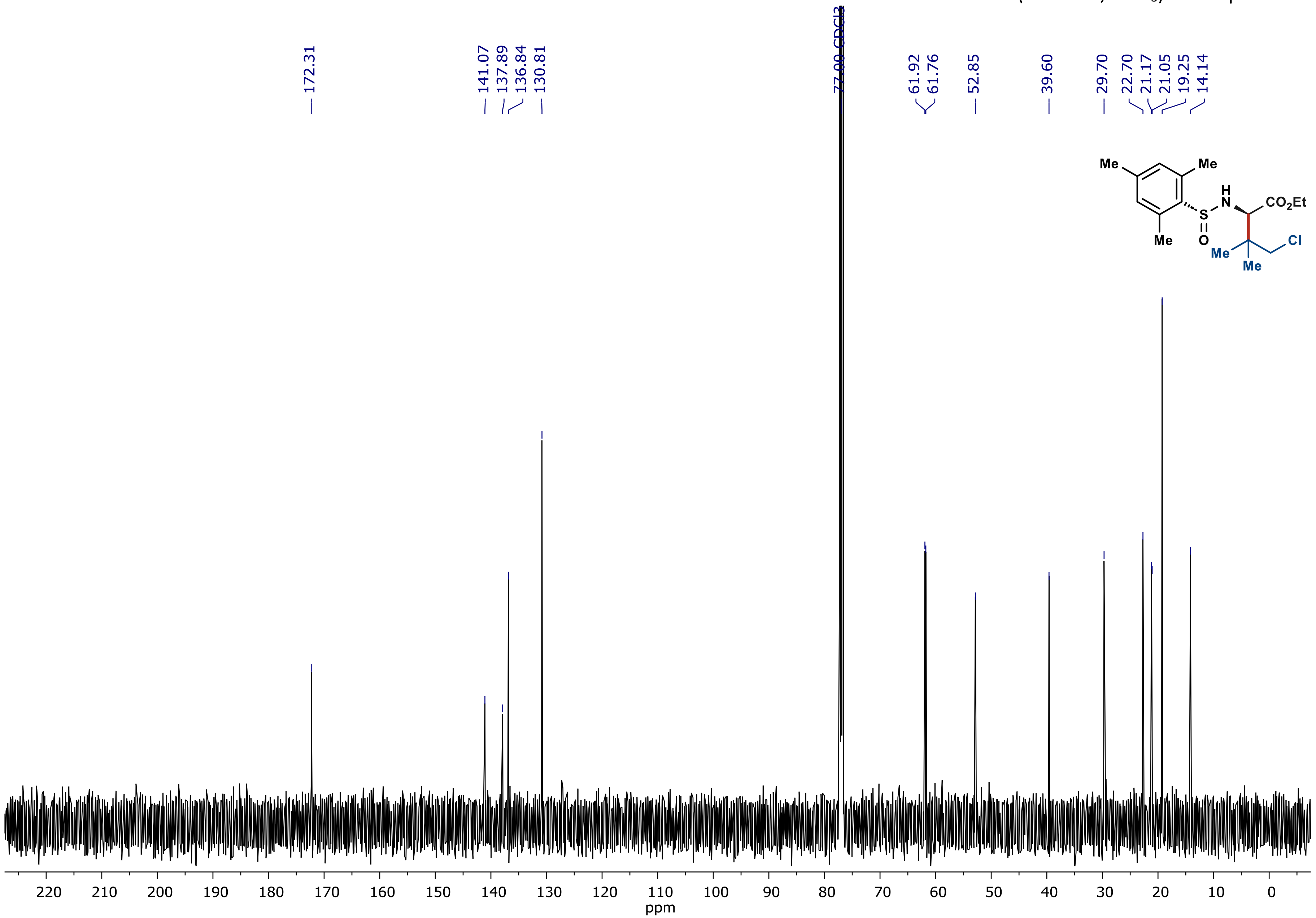

$^{1}$**H NMR** (500 MHz, $CDCl_3$) of compound **5p**

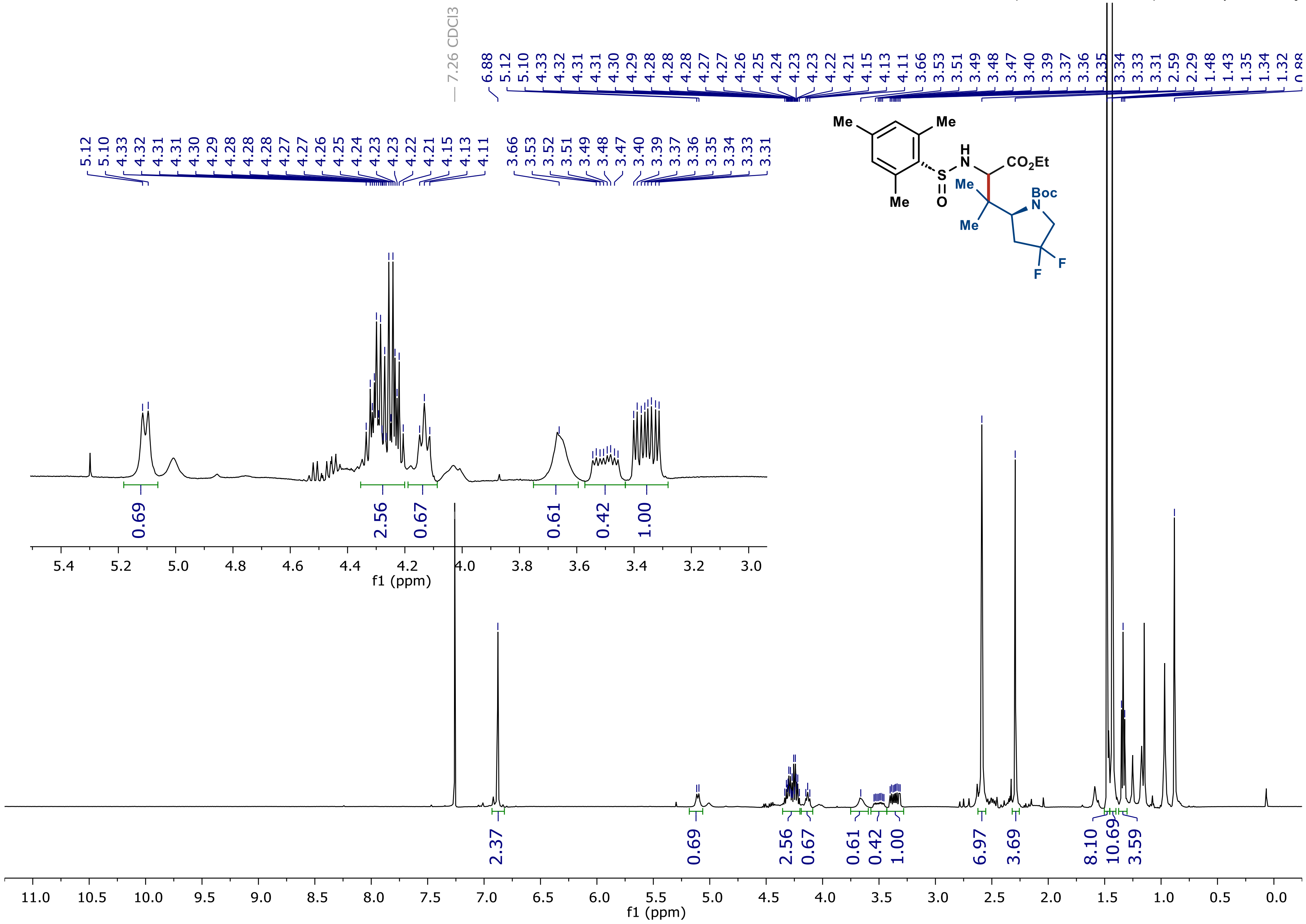

$^{13}$**C NMR** (126 MHz, $CDCl_3$) of compound **5p**

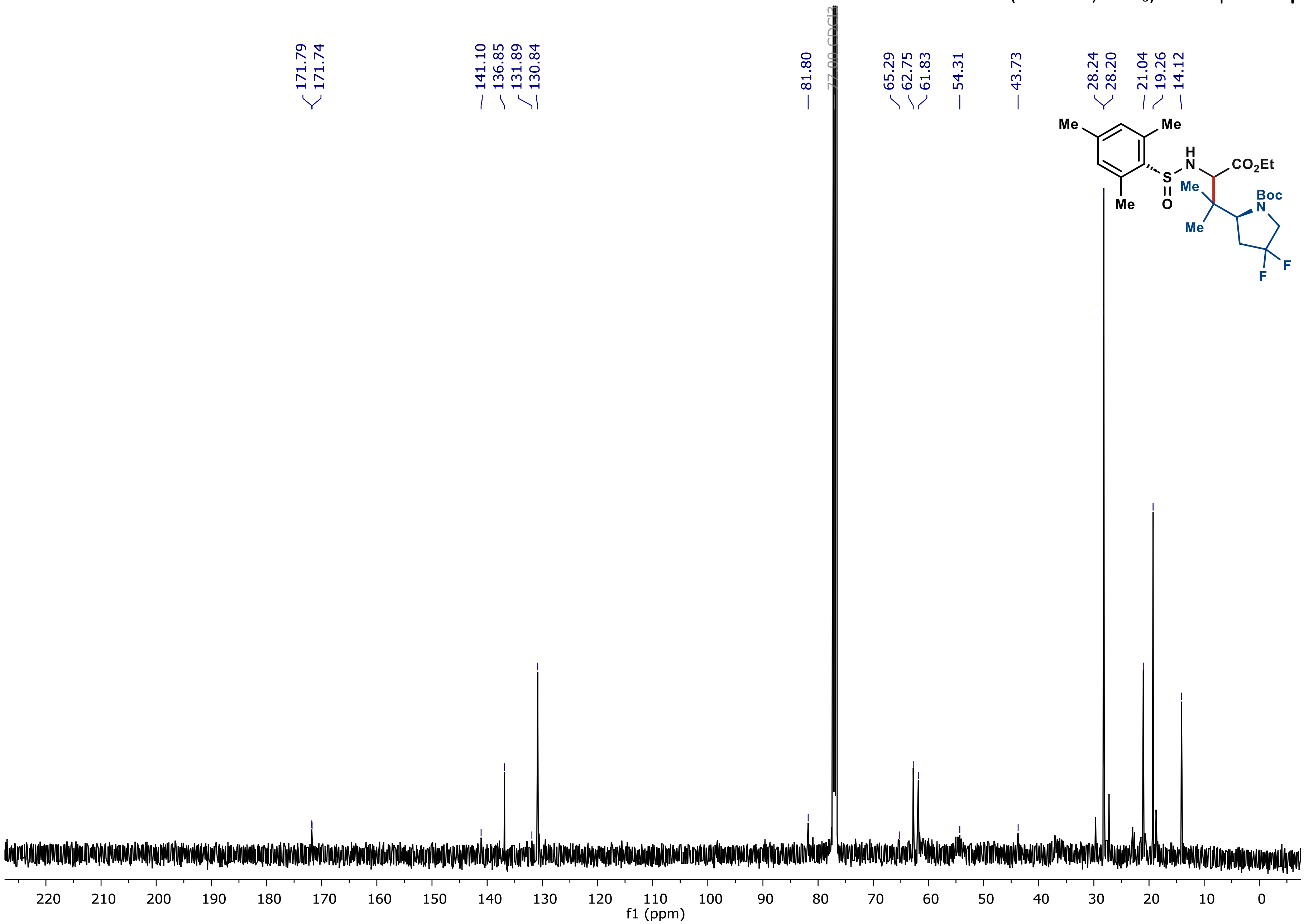

**$^{1}$H NMR** (500 MHz, $CDCl_3$) of compound **5q**

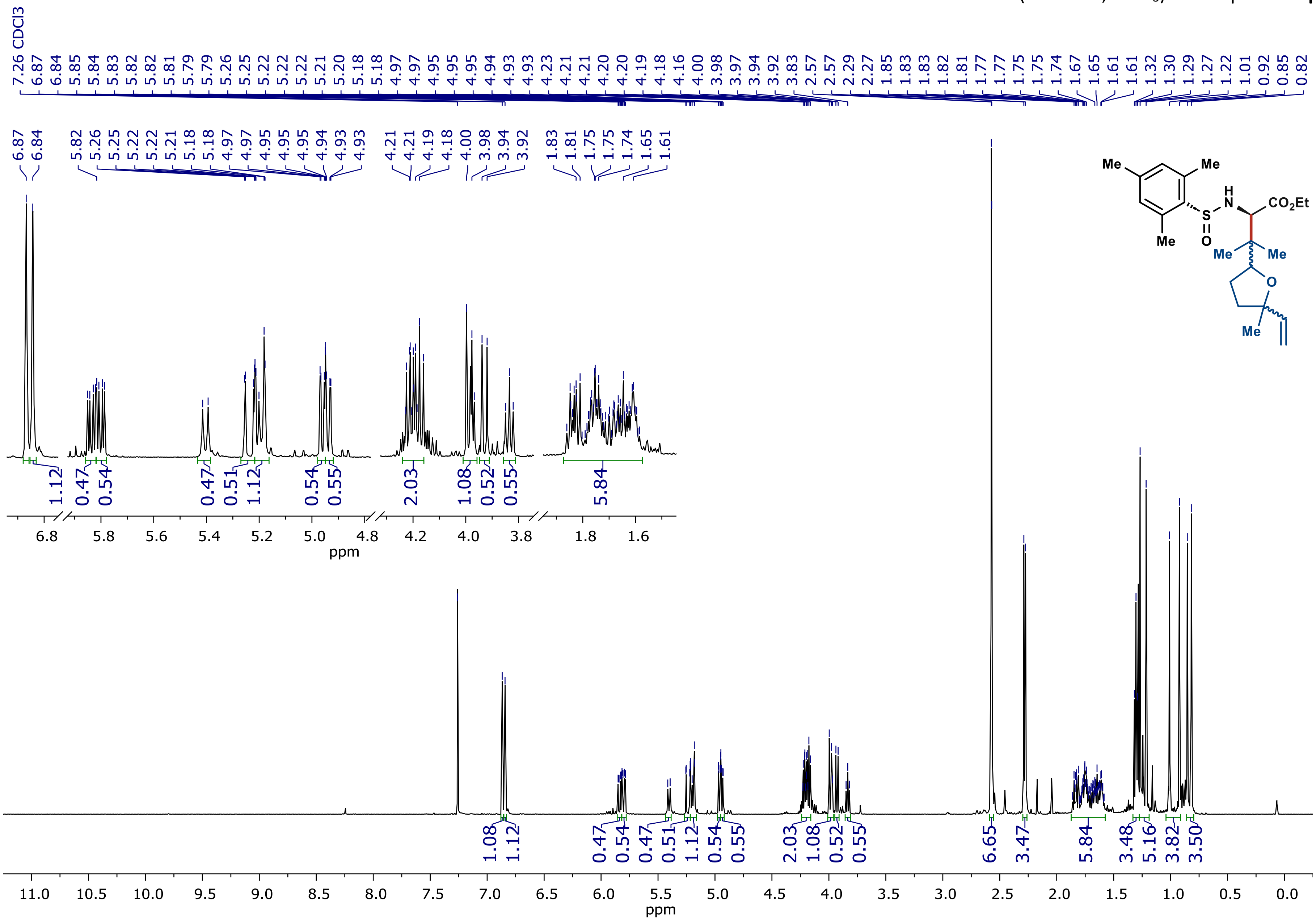

$^{13}$**C NMR** (126 MHz, $CDCl_3$) of compound **5q**

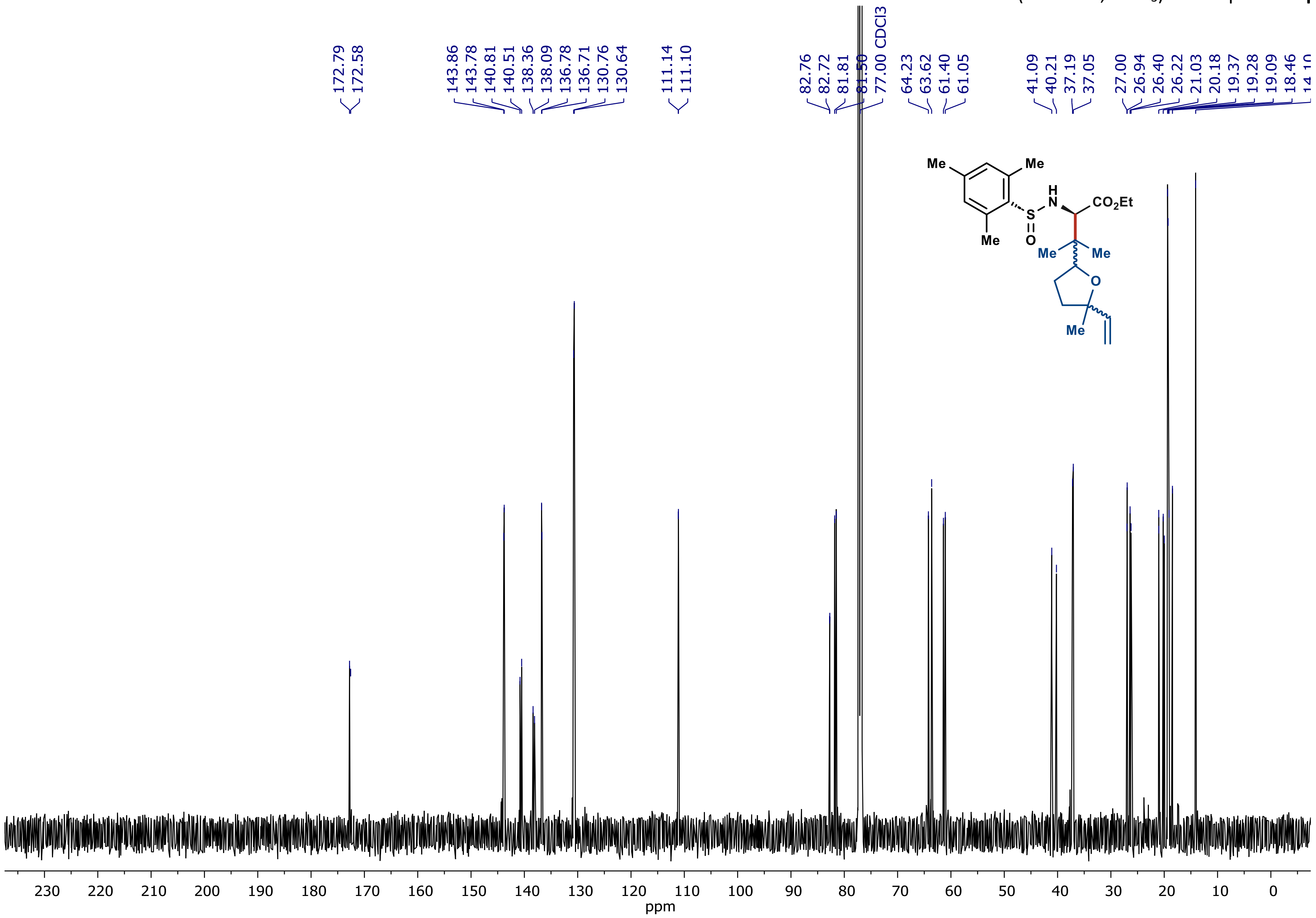

**COSY** of compound **5q**

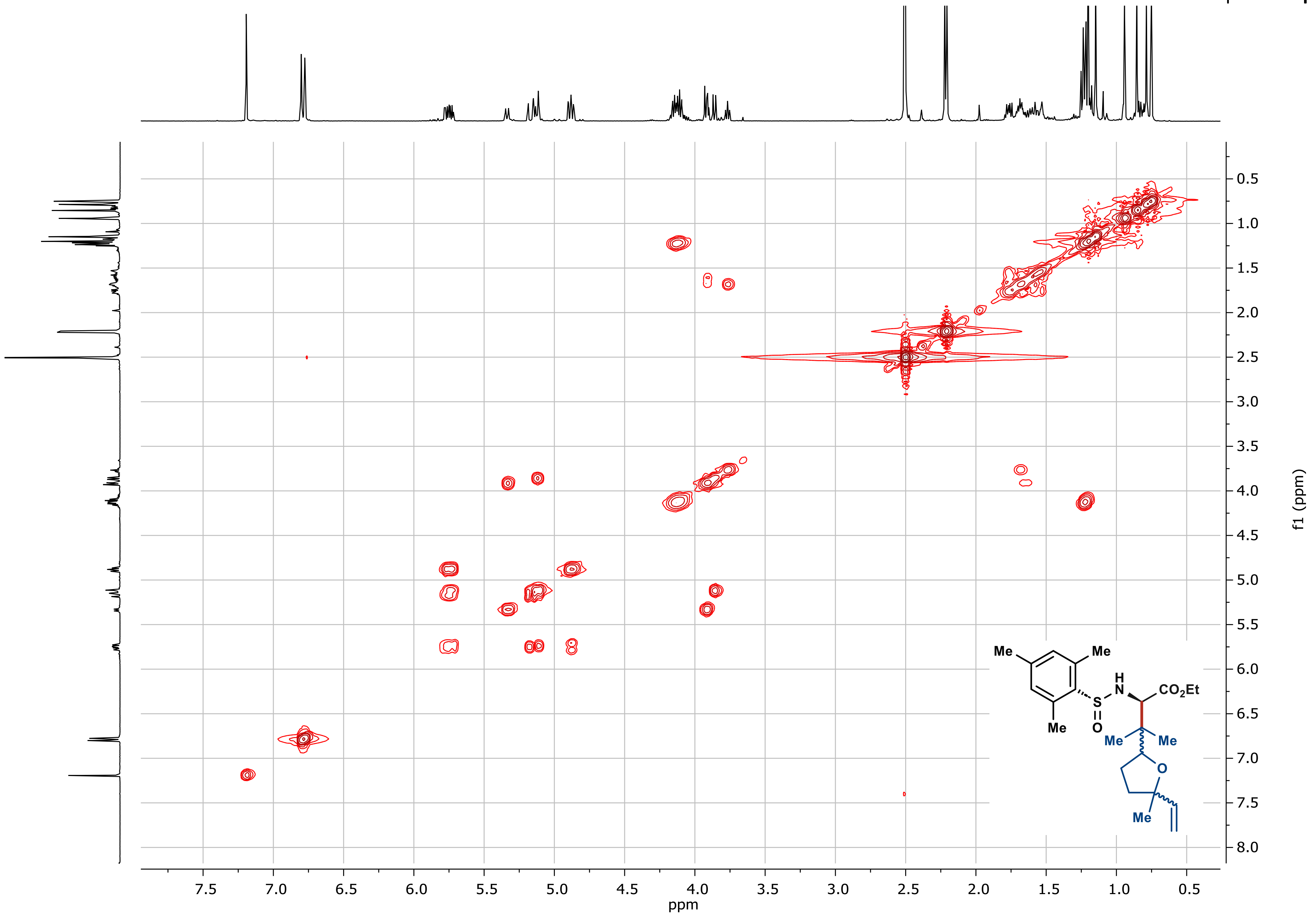

HSQC of compound **5q**

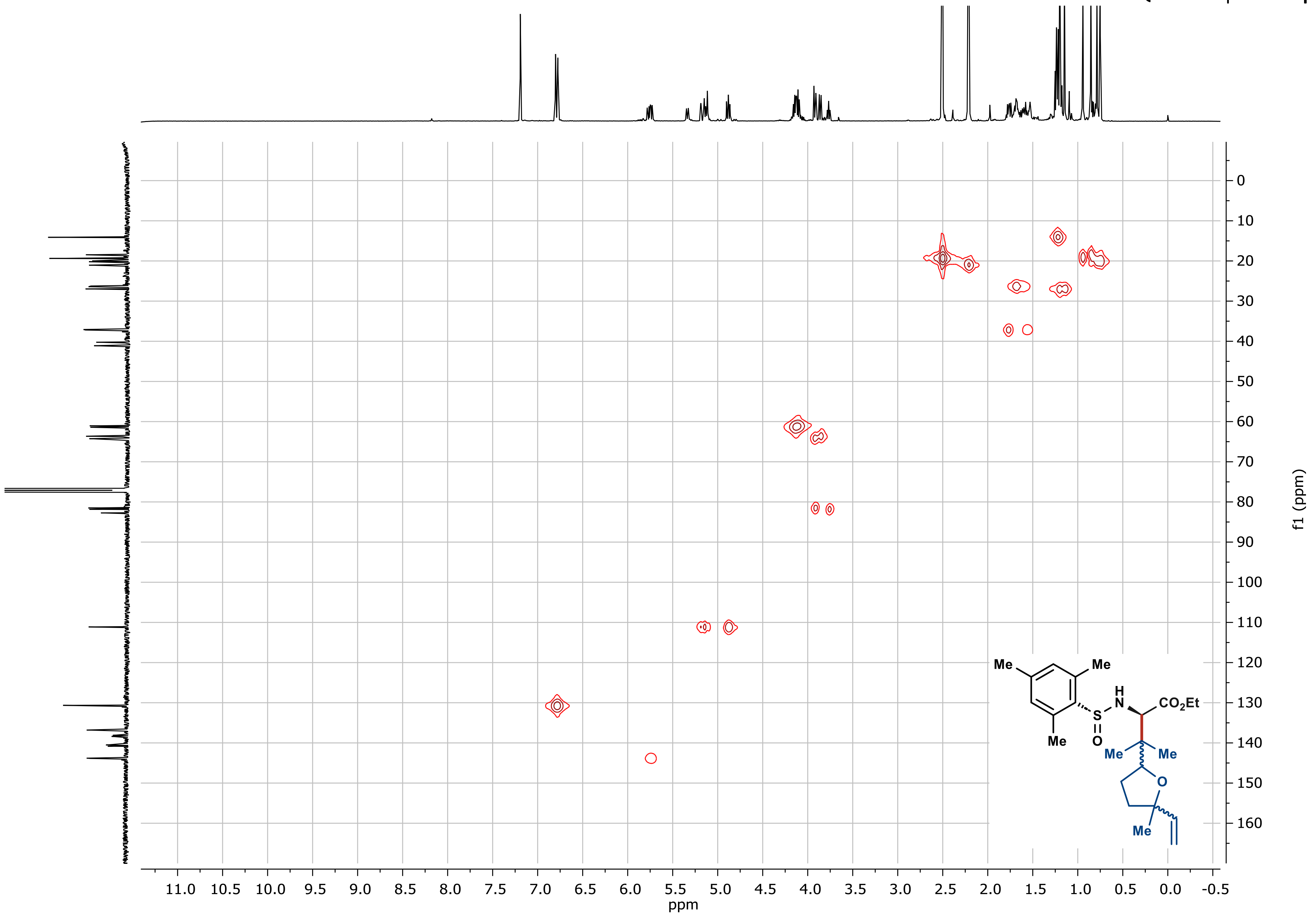

**HMBC** of compound **5q**

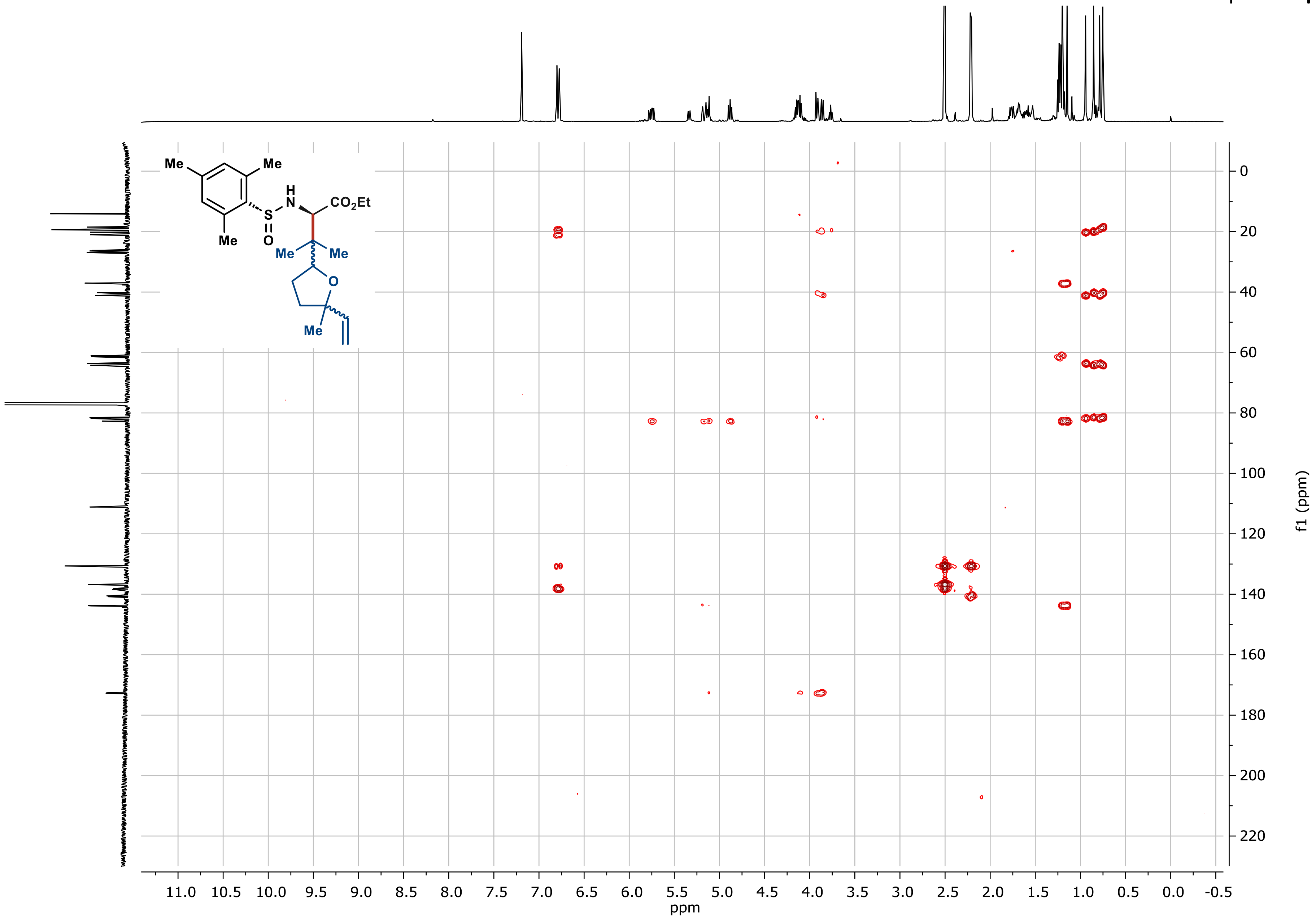

**$^1$H NMR** (500 MHz, $CDCl_3$) of compound **5r**

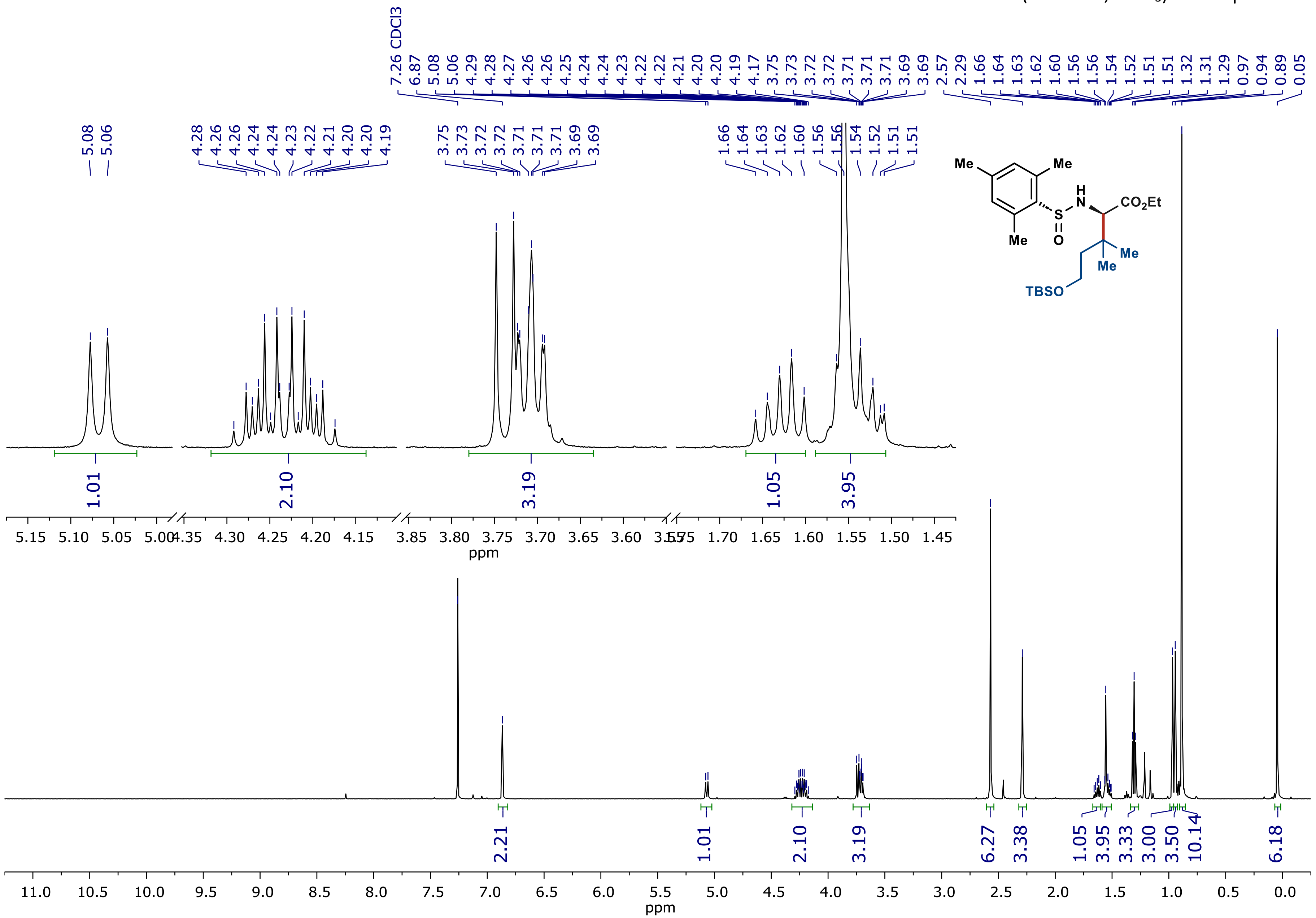

$^{13}$**C NMR** (126 MHz, $CDCl_3$) of compound **5r**

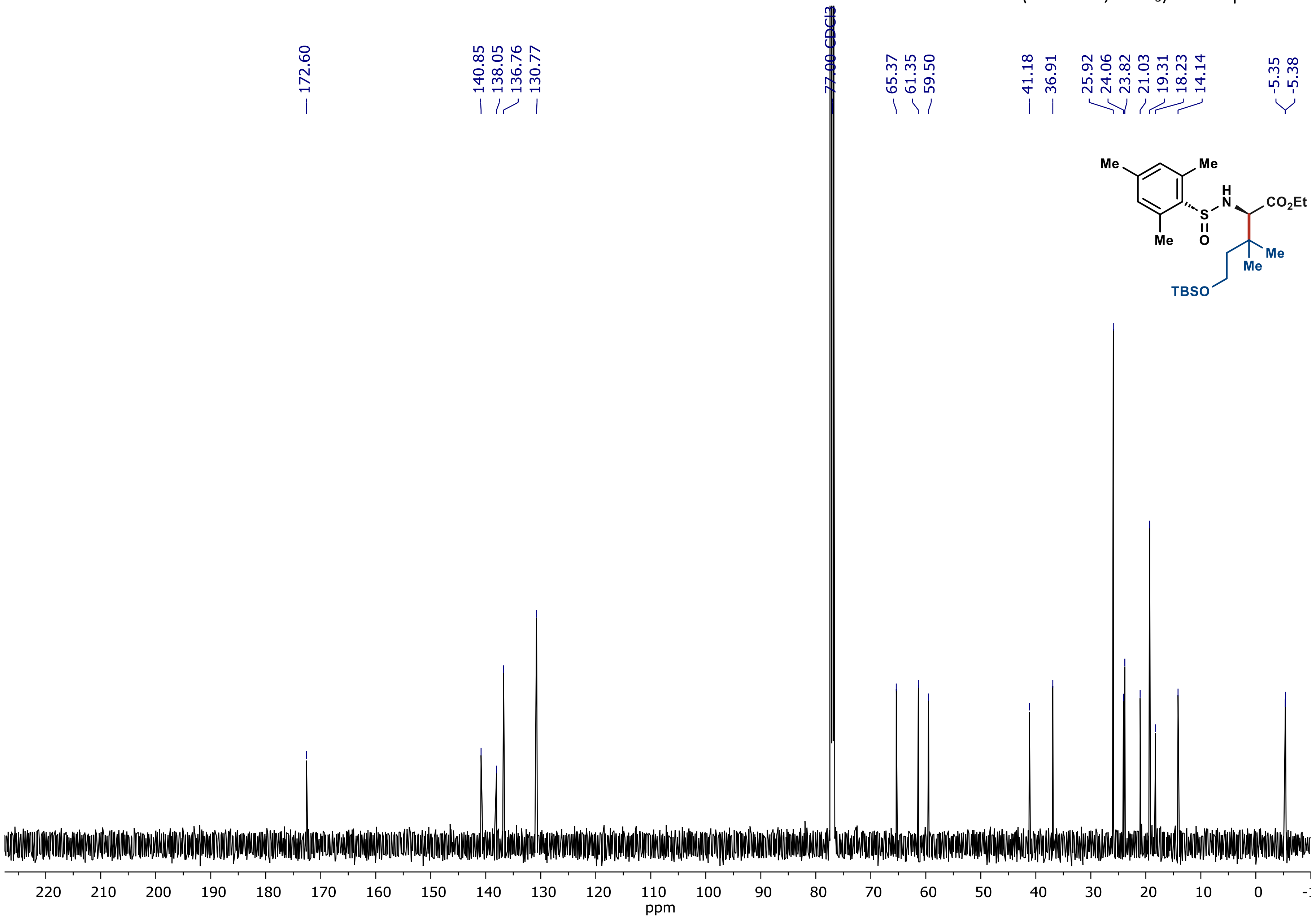

**COSY** of compound **5r**

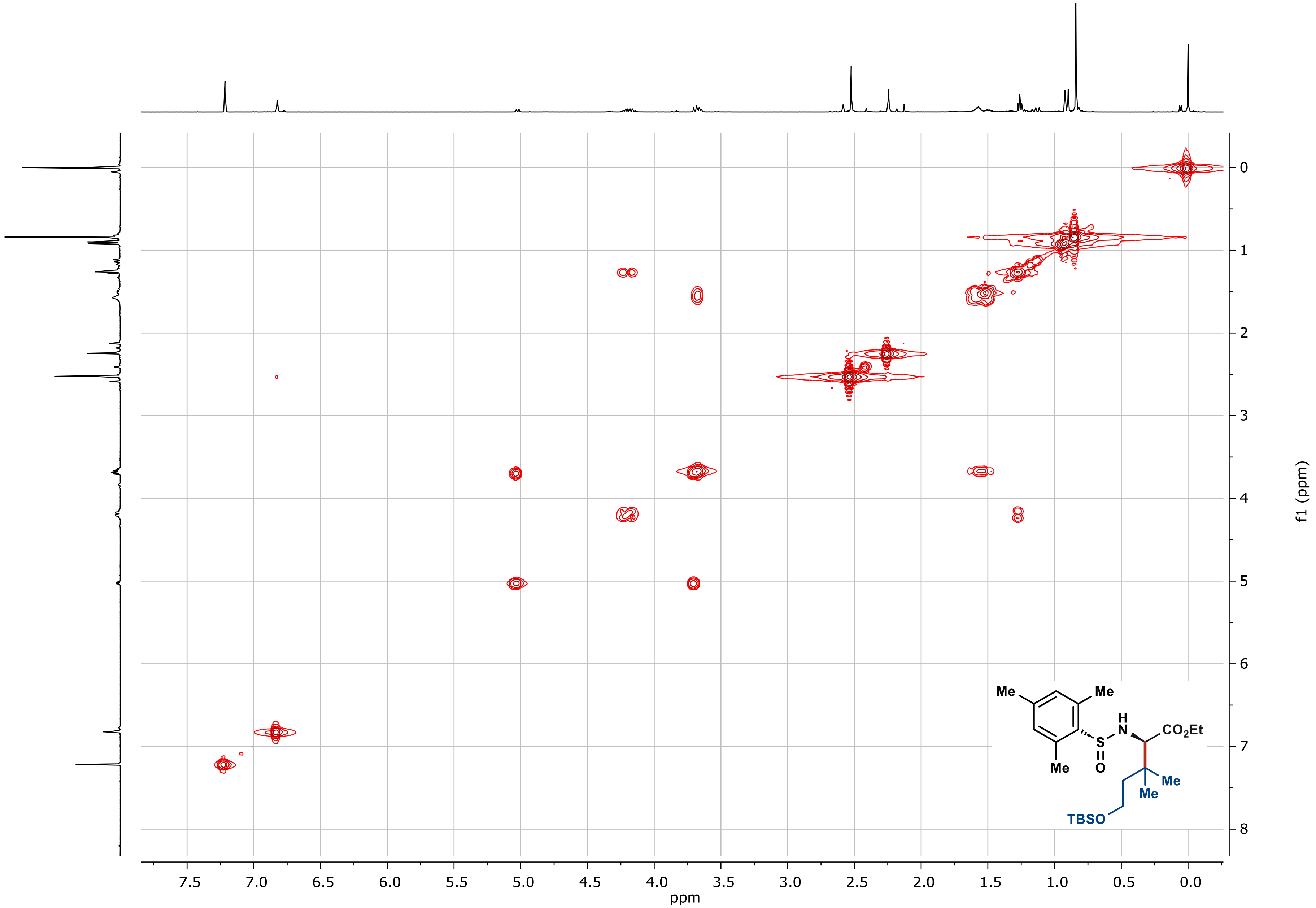

**HSQC** of compound **5r**

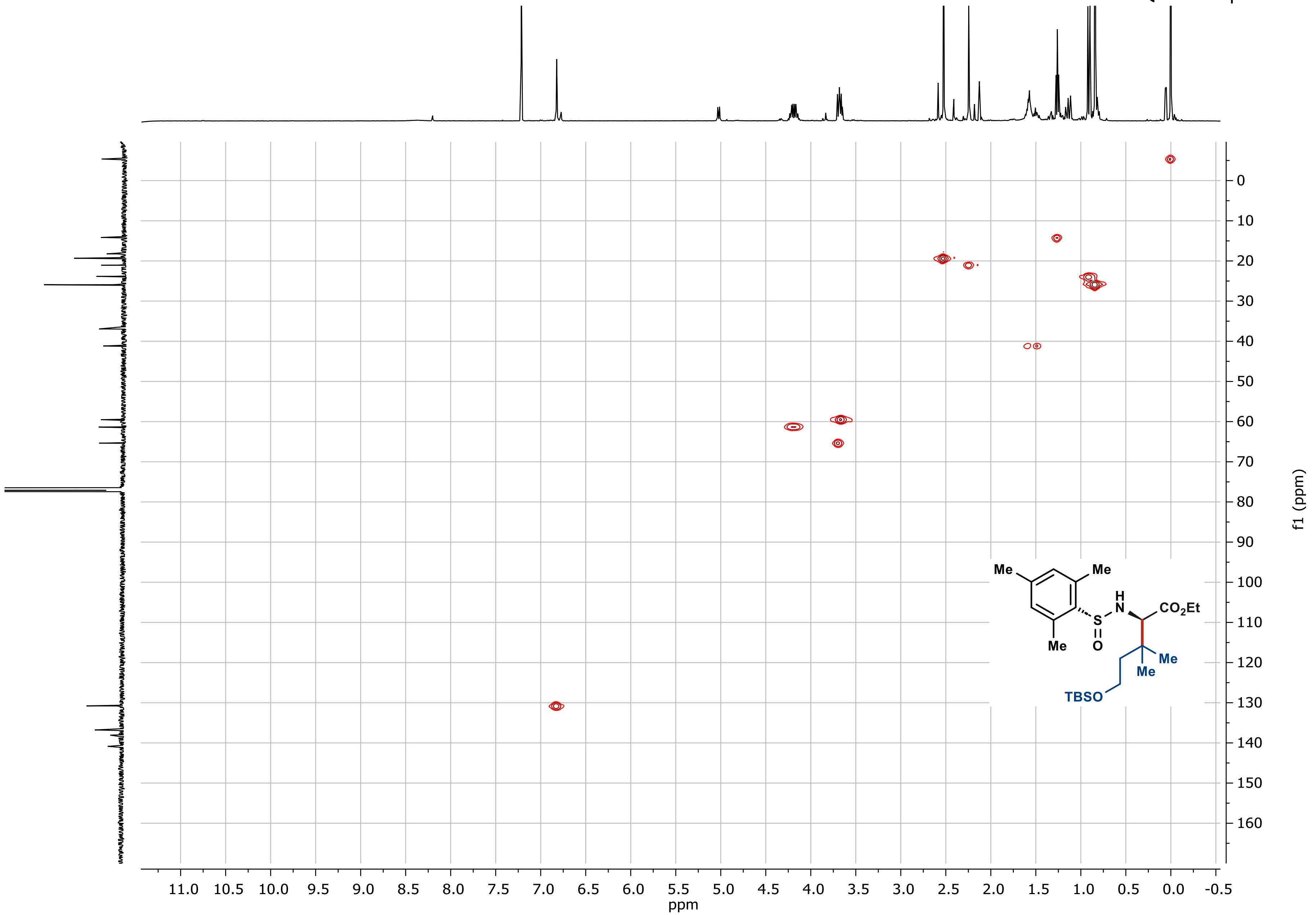

$^{1}$**H NMR** (500 MHz, $CDCl_3$) of compound **5s**

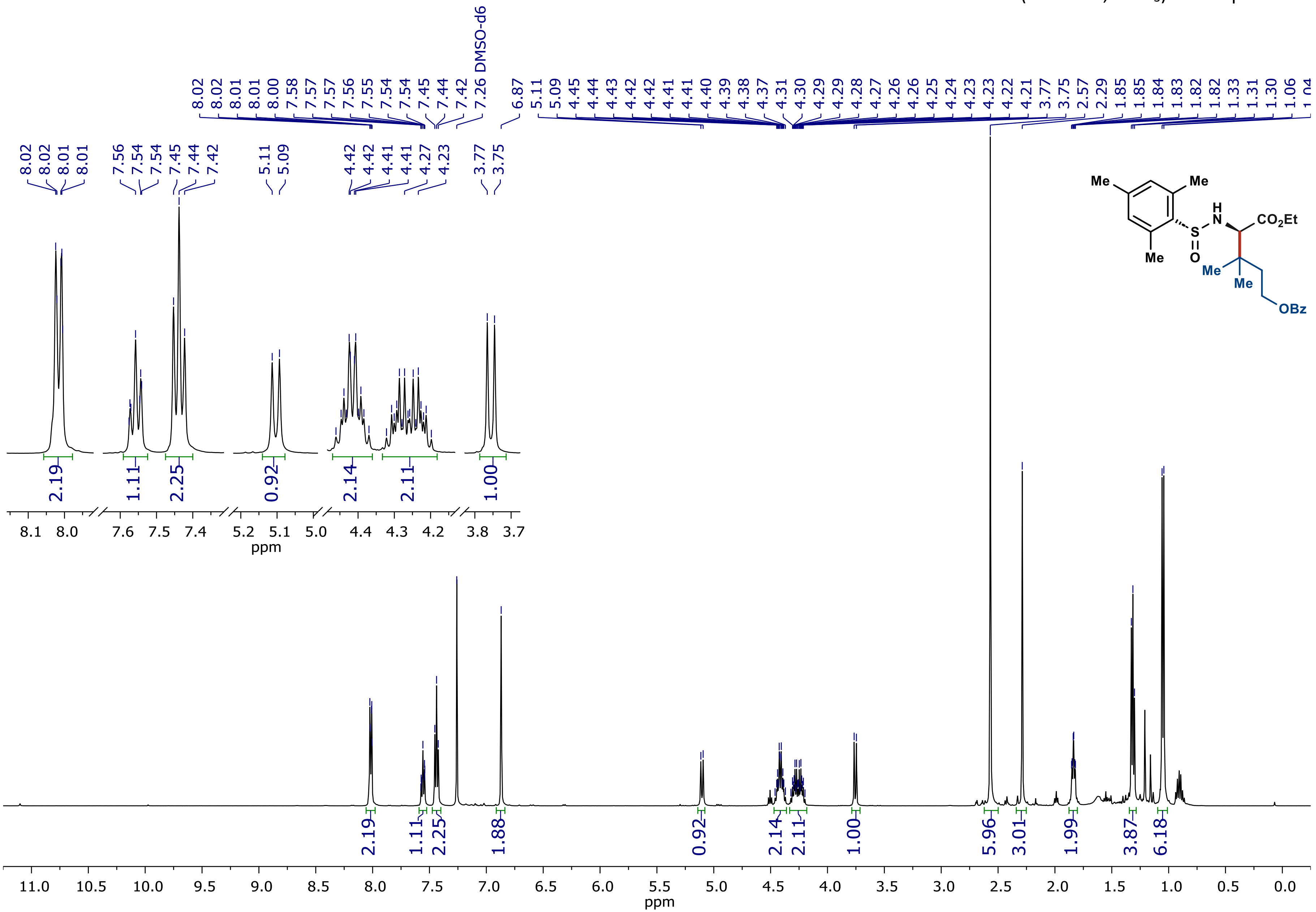

$^{13}$**C NMR** (126 MHz, $CDCl_3$) of compound **5s**

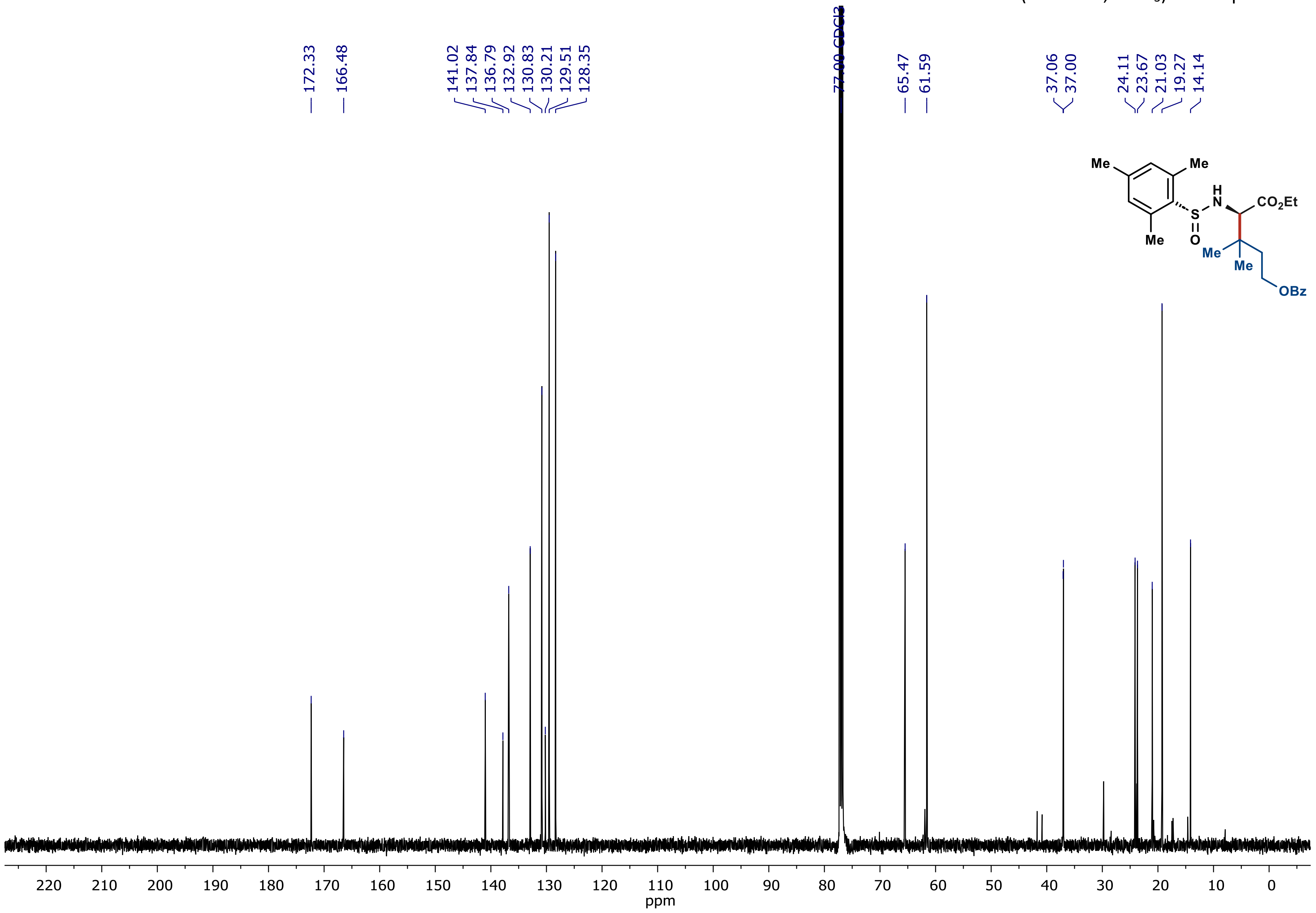

**COSY** of compound **5s**

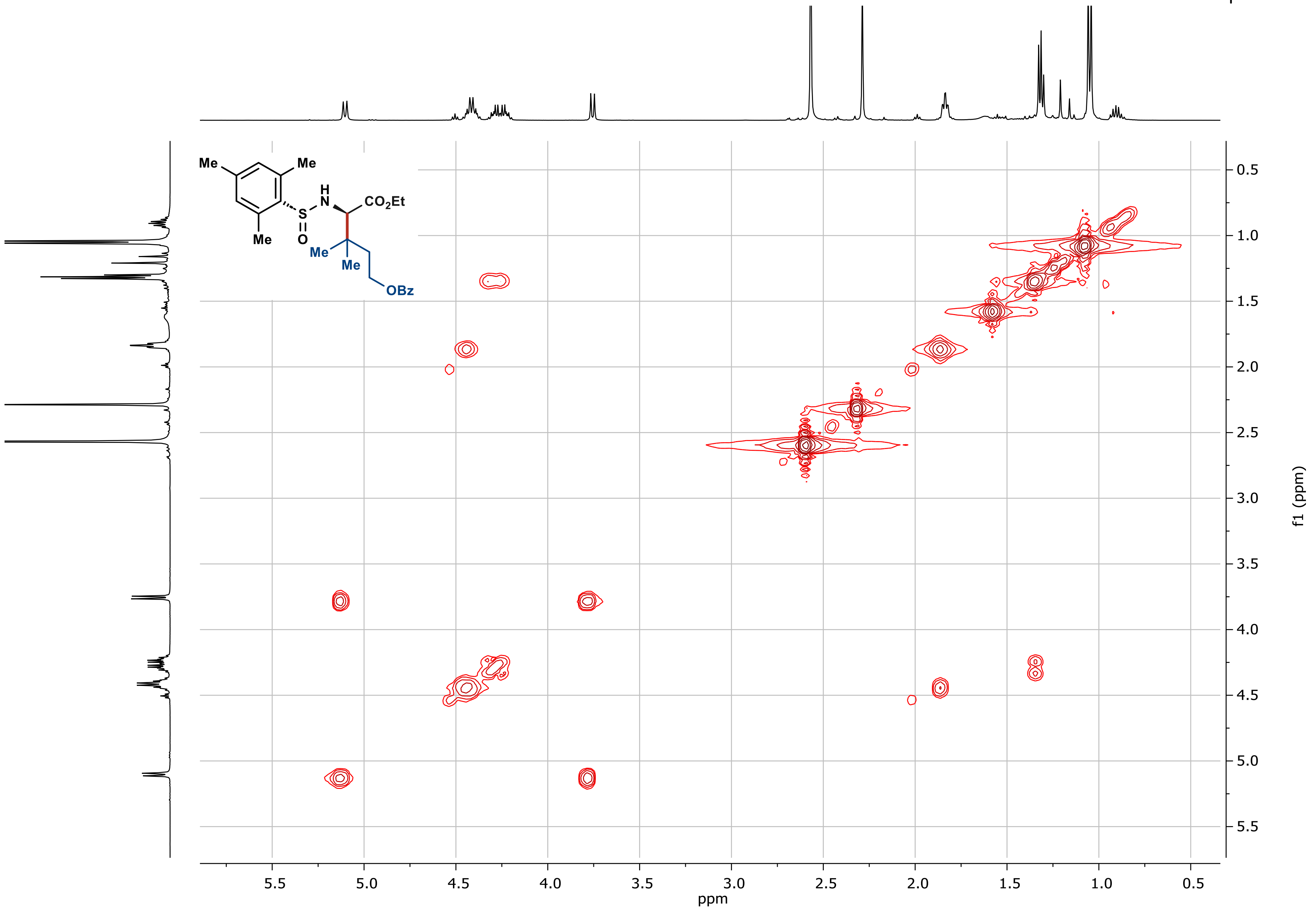

**HSQC** of compound **5s**

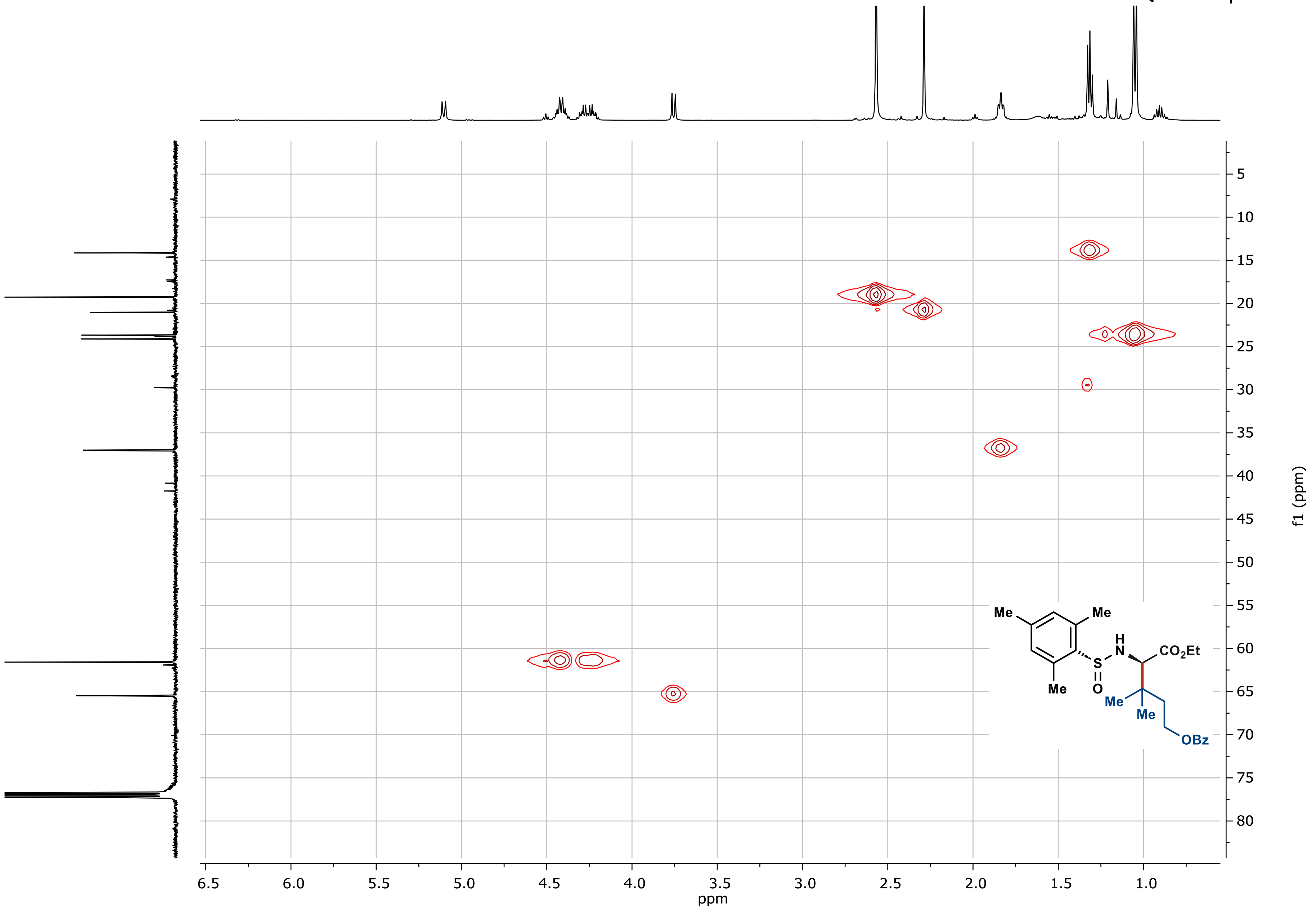

$^{1}$**H NMR** (500 MHz, $CDCl_3$) of compound **5t**

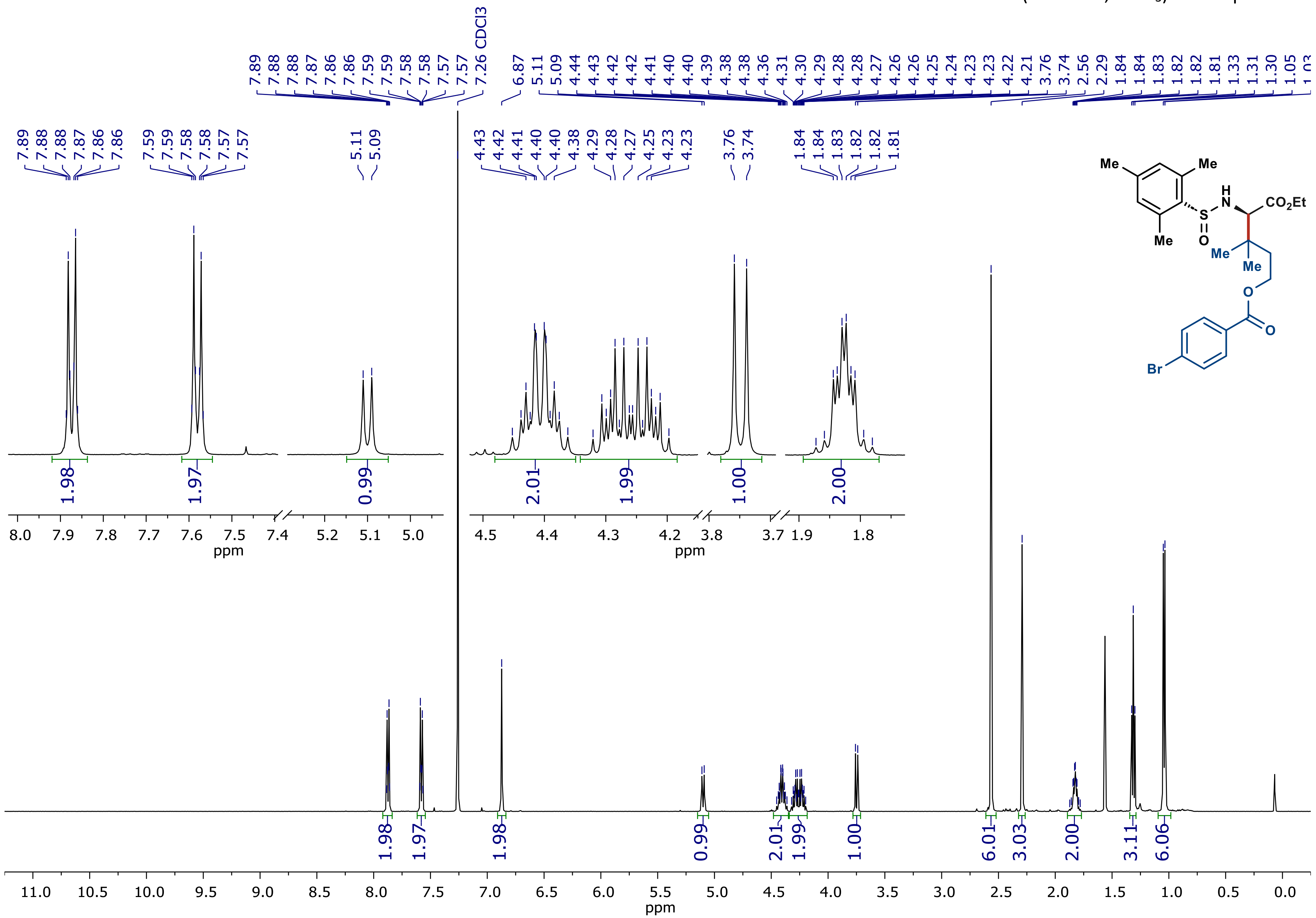

**$^{13}$C NMR** (126 MHz, $CDCl_3$) of compound **5t**

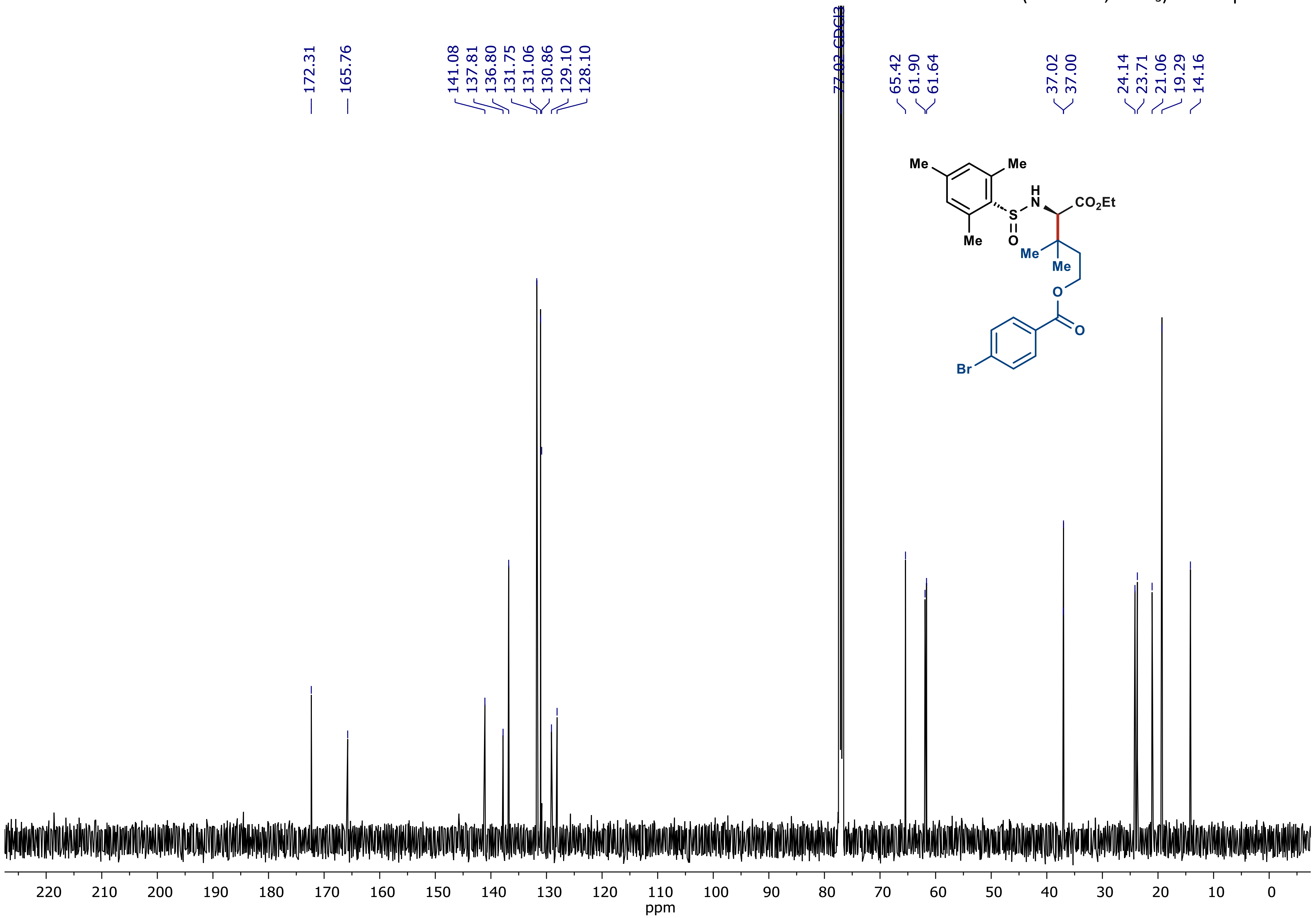

**COSY** of compound **5t**

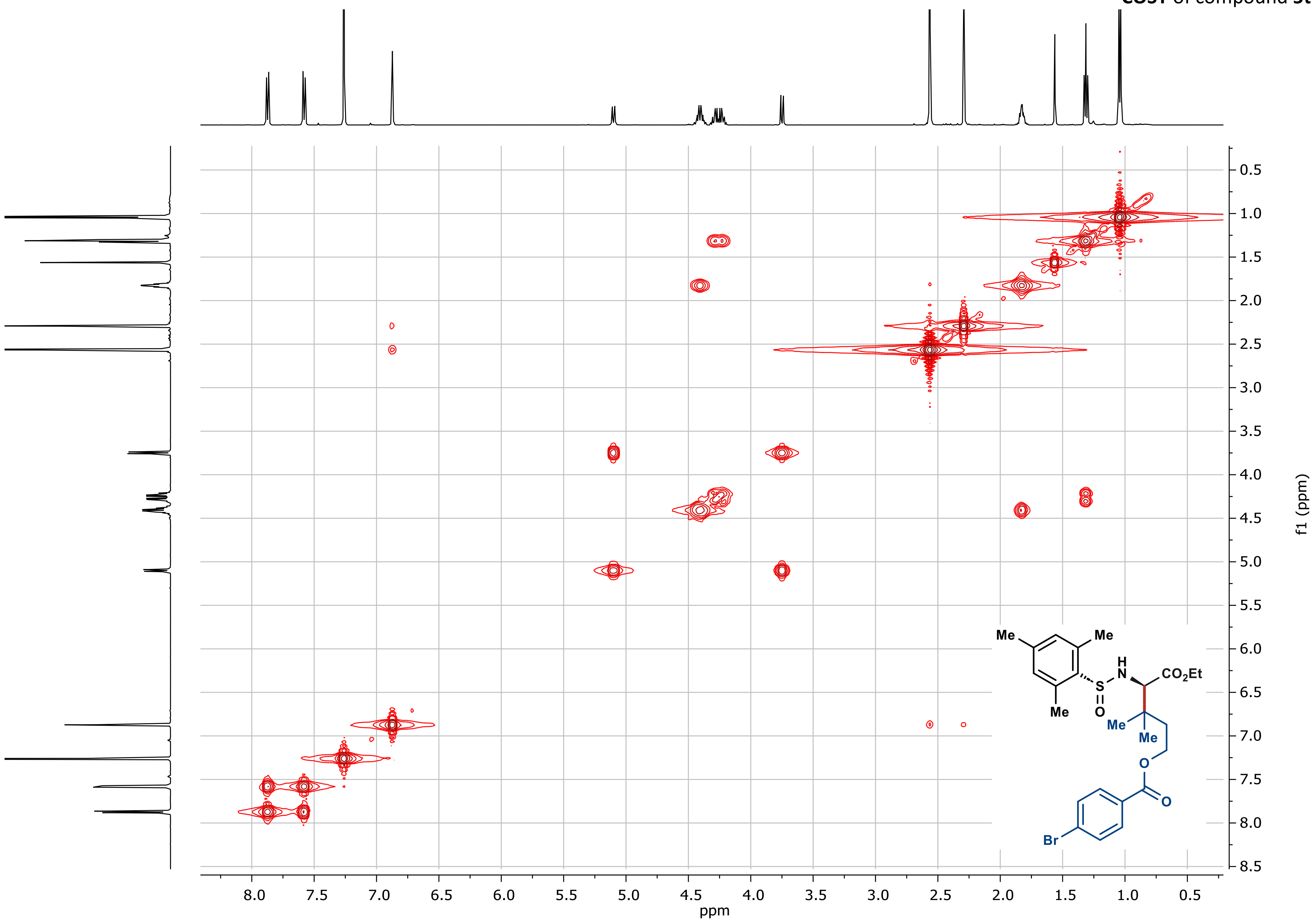

**HSQC** of compound **5t**

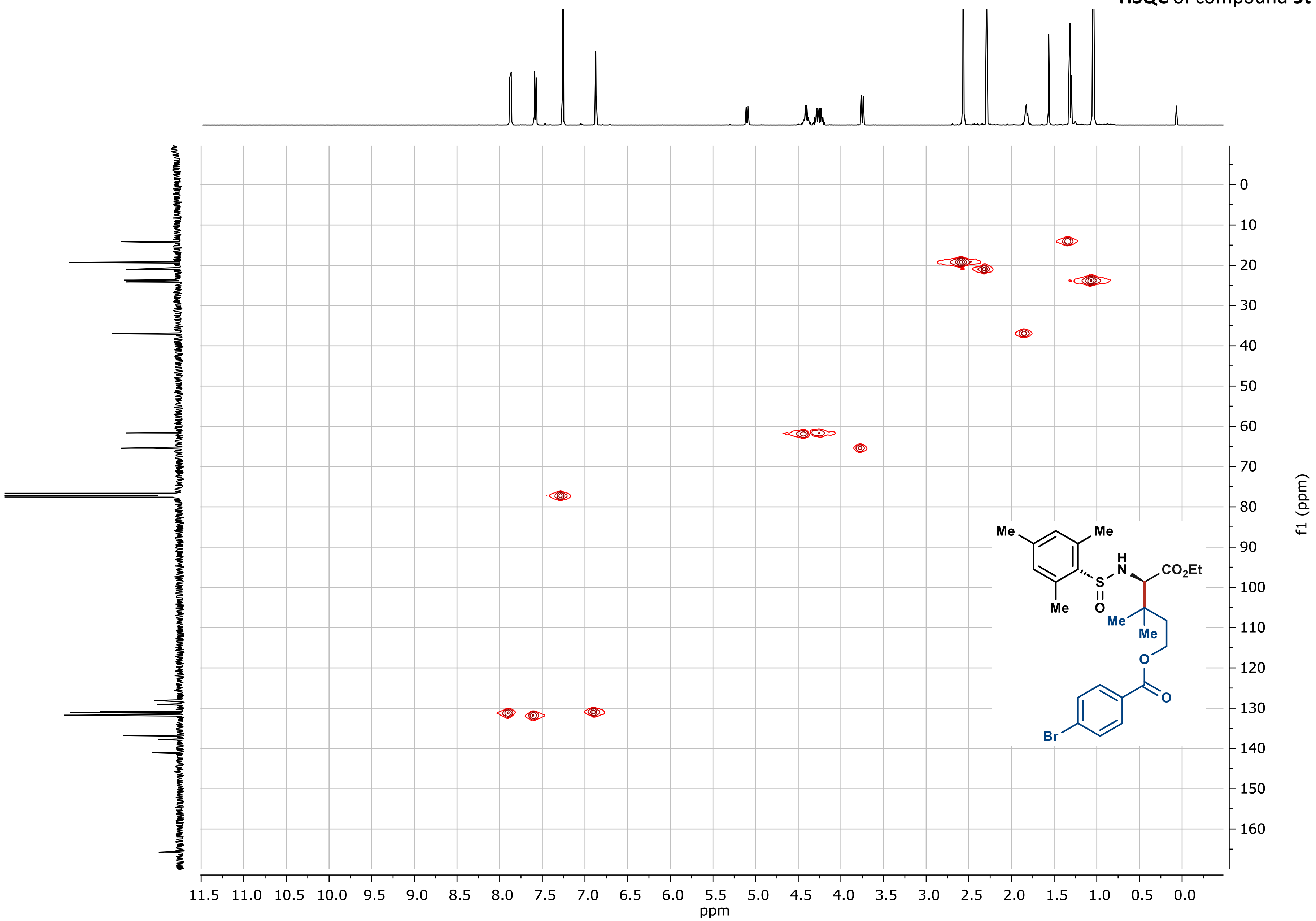

**$^1$H NMR** (500 MHz, $CDCl_3$) of compound **5u**

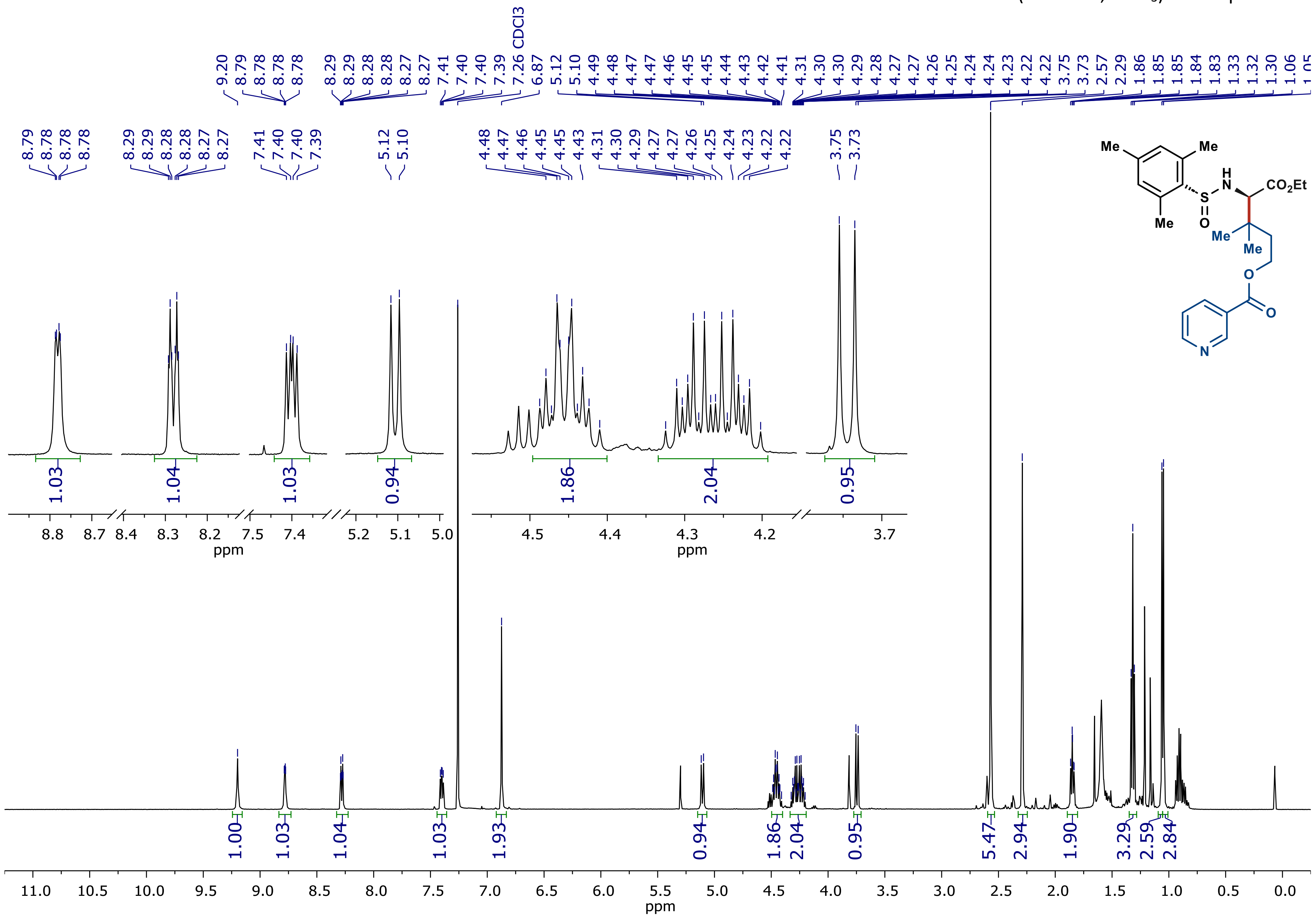

**$^{13}$C NMR** (126 MHz, $CDCl_3$) of compound **5u**

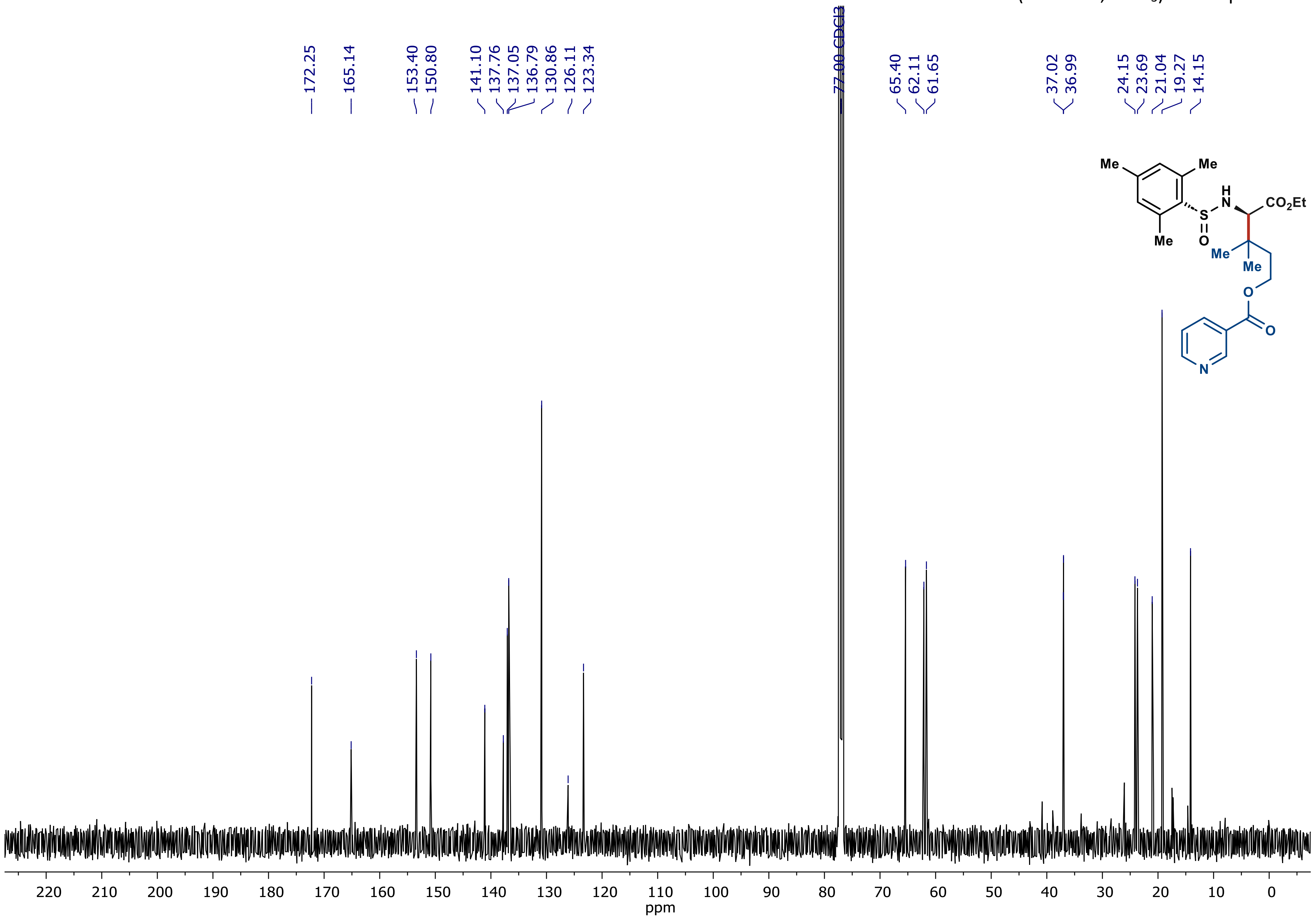

**COSY** of compound **5u**

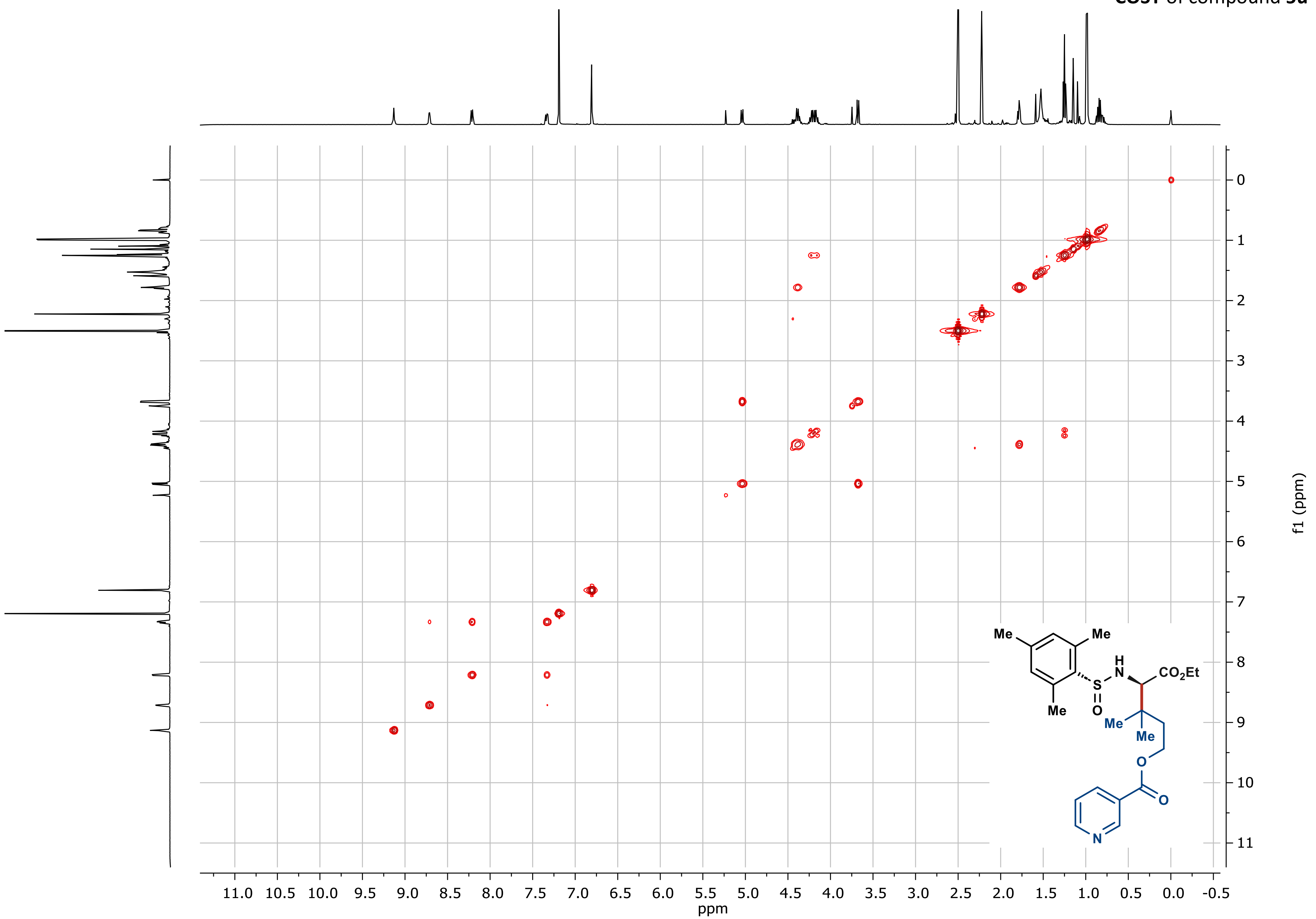

**HSQC** of compound **5u**

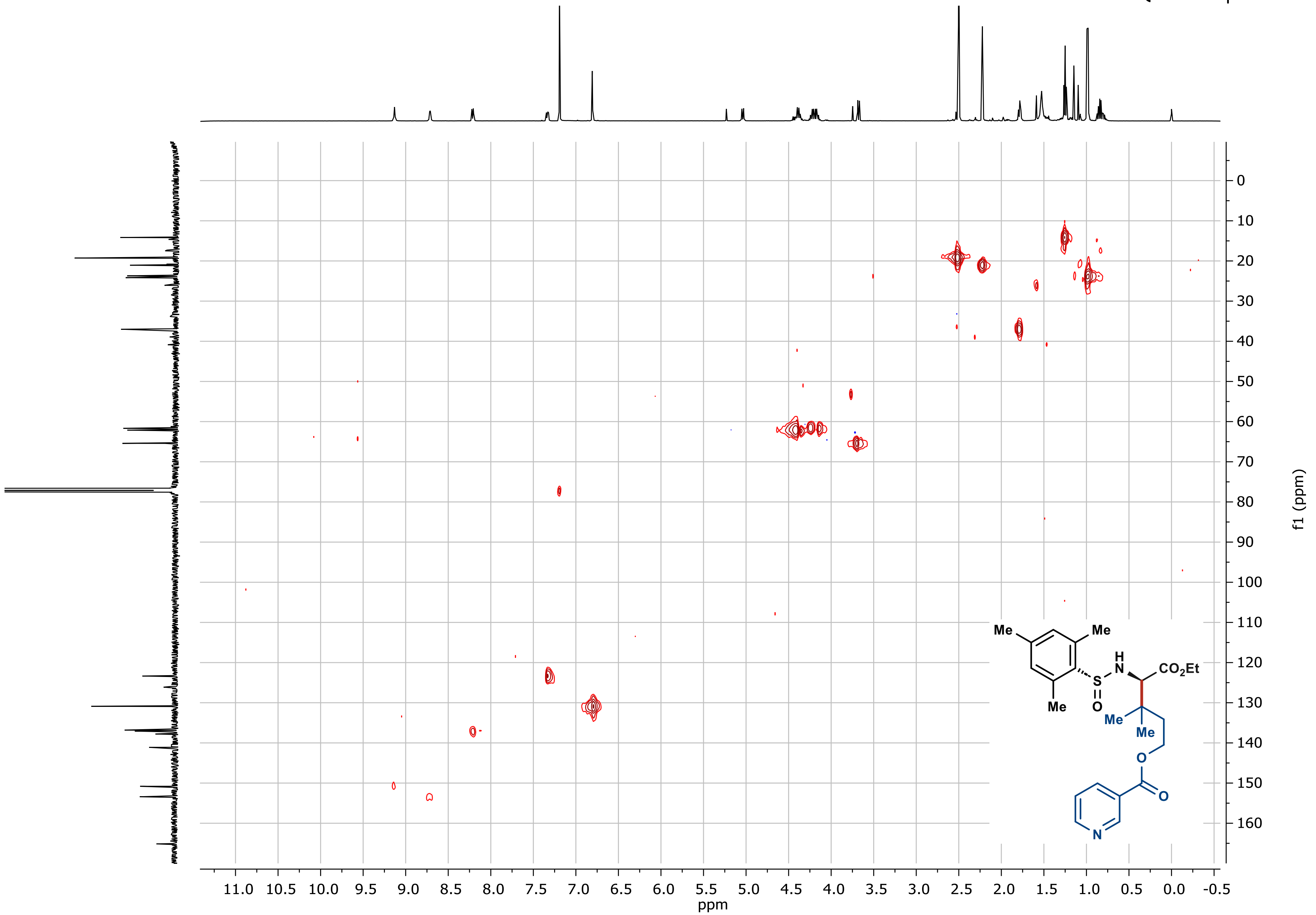

**$^1$H NMR** (500 MHz, $CDCl_3$) of compound **5v**

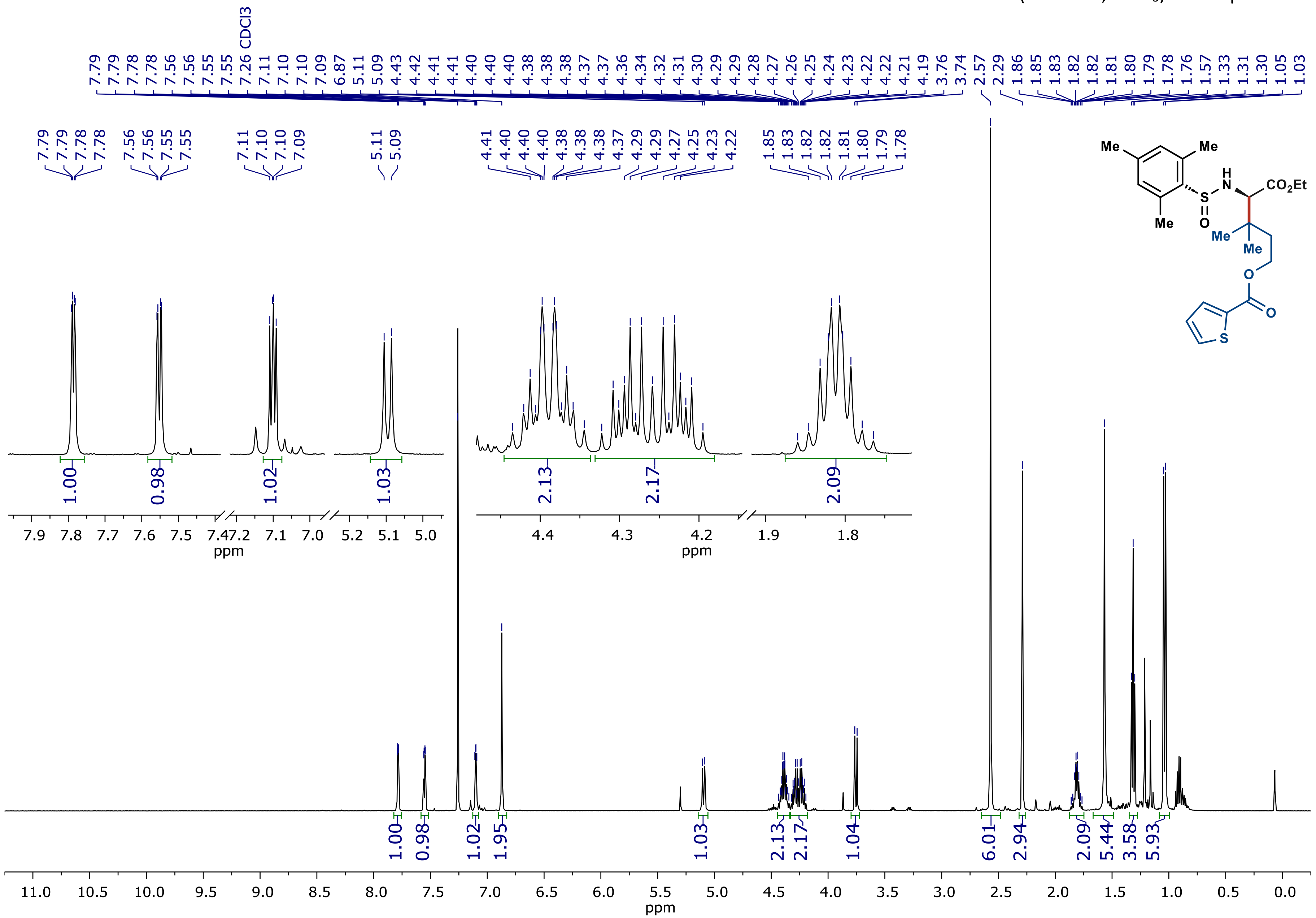

**$^{13}$C NMR** (126 MHz, $CDCl_3$) of compound **5v**

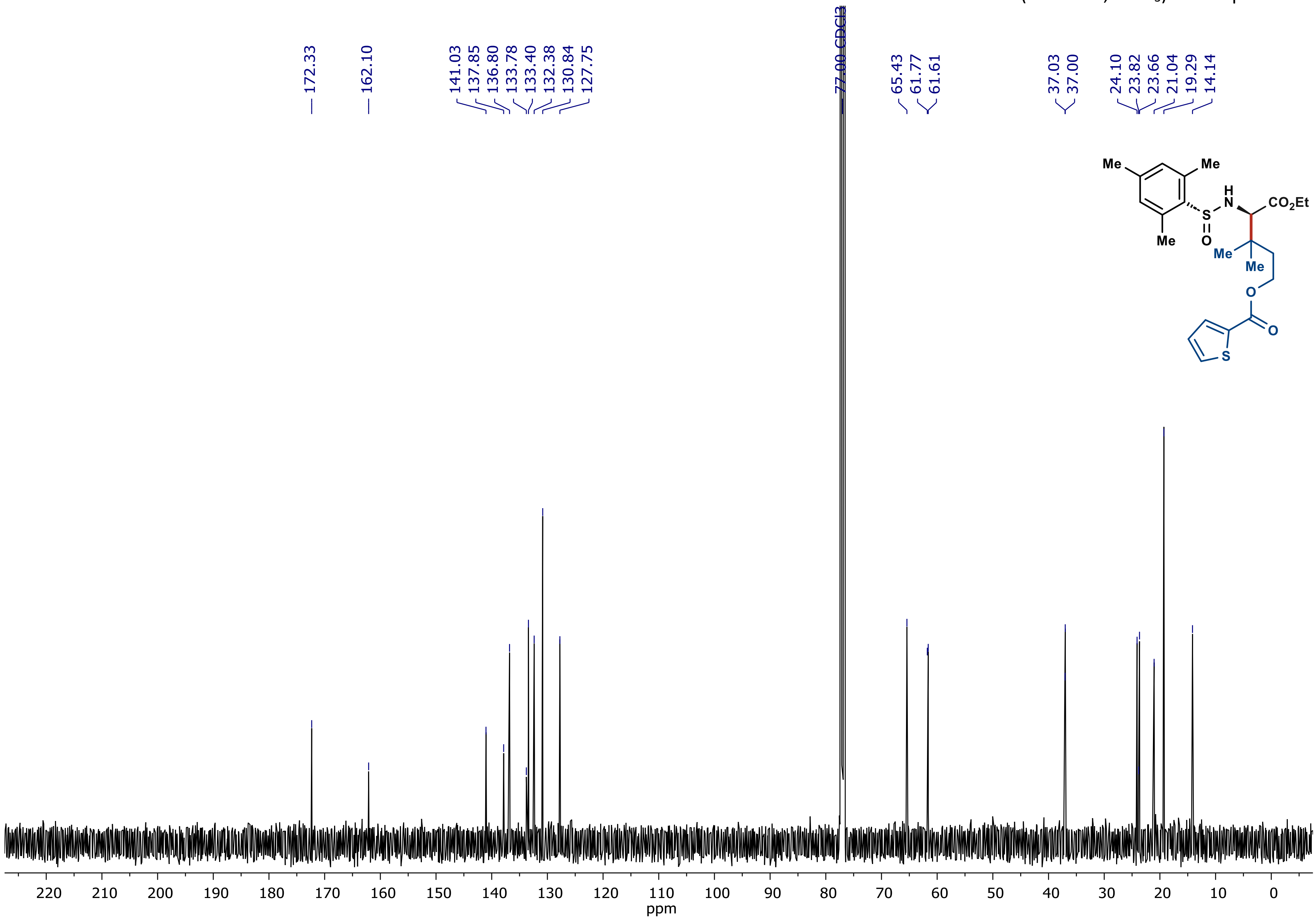

**COSY** of compound **5v**

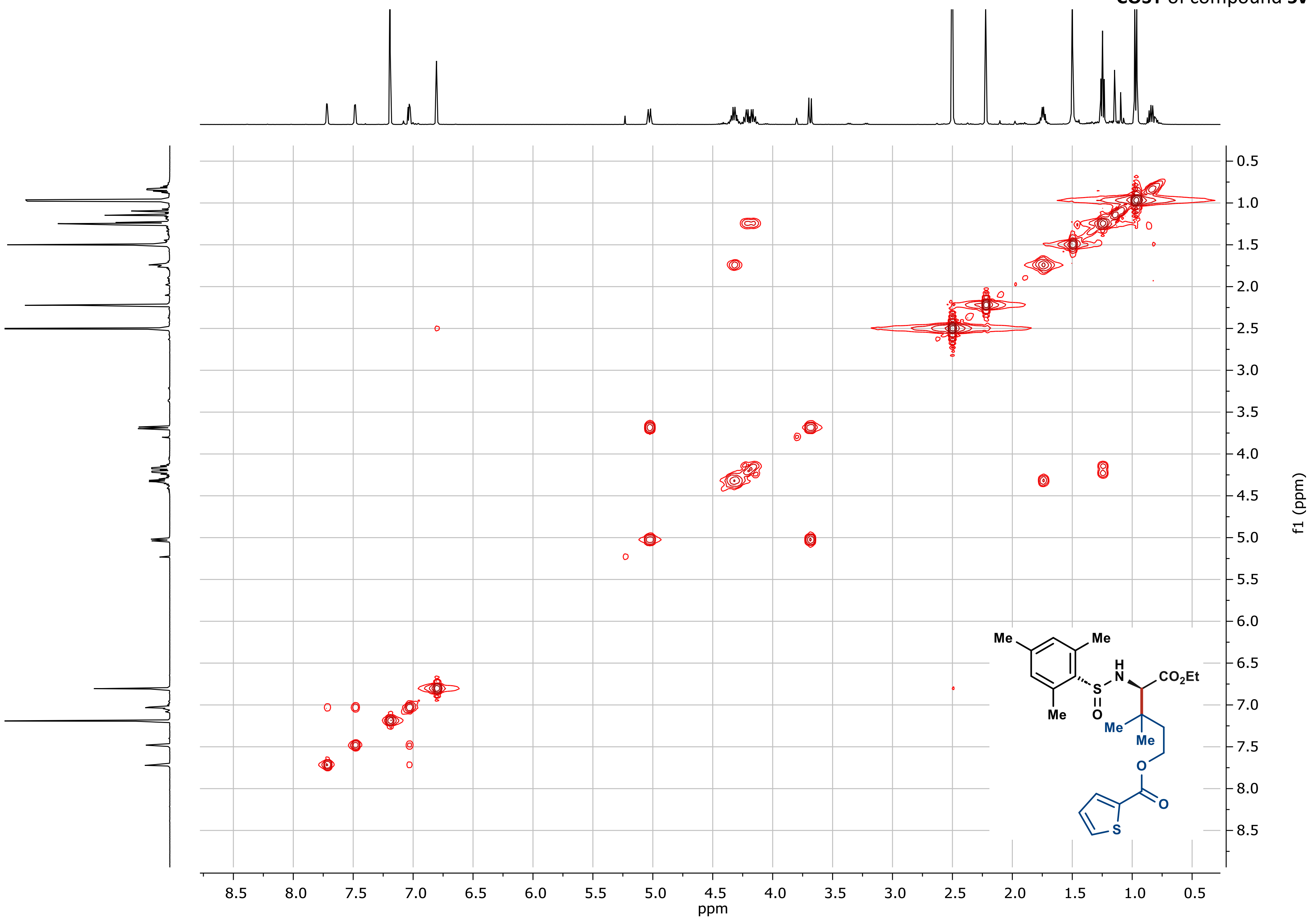

**HSQC** of compound **5v**

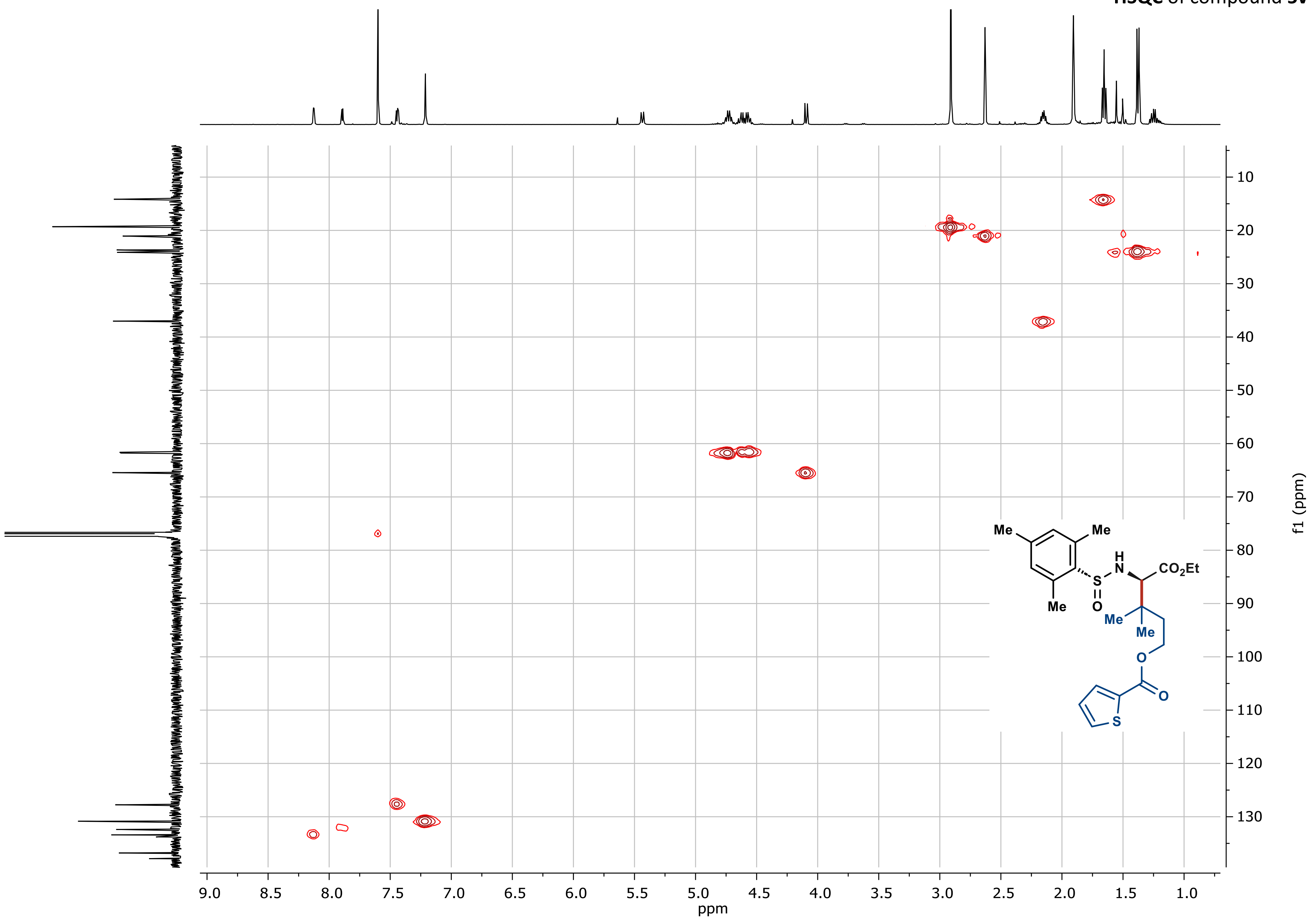

$^{1}$**H NMR** (500 MHz, $CDCl_3$) of compound **5w**

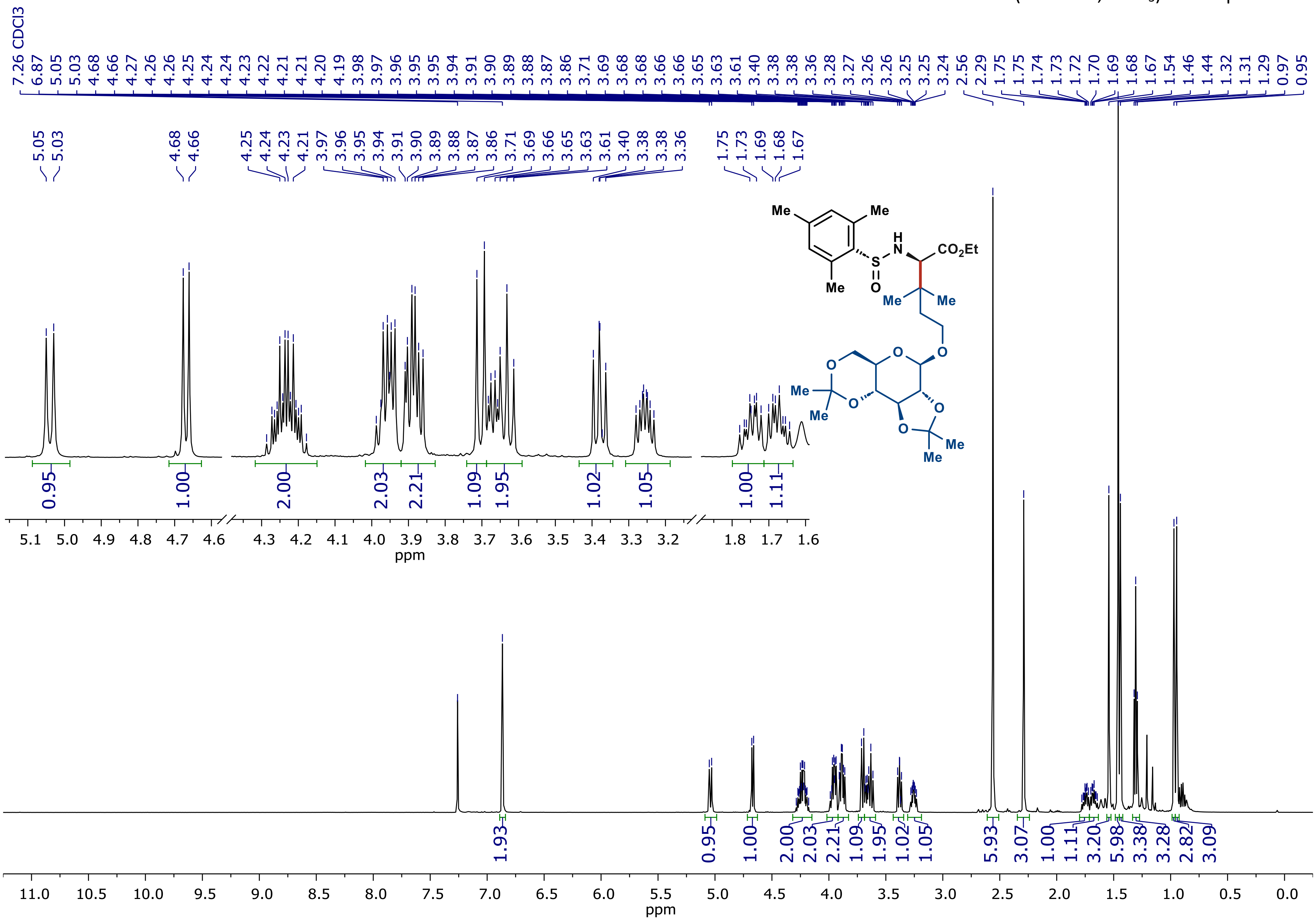

**$^{13}$C NMR** (126 MHz, $CDCl_3$) of compound **5w**

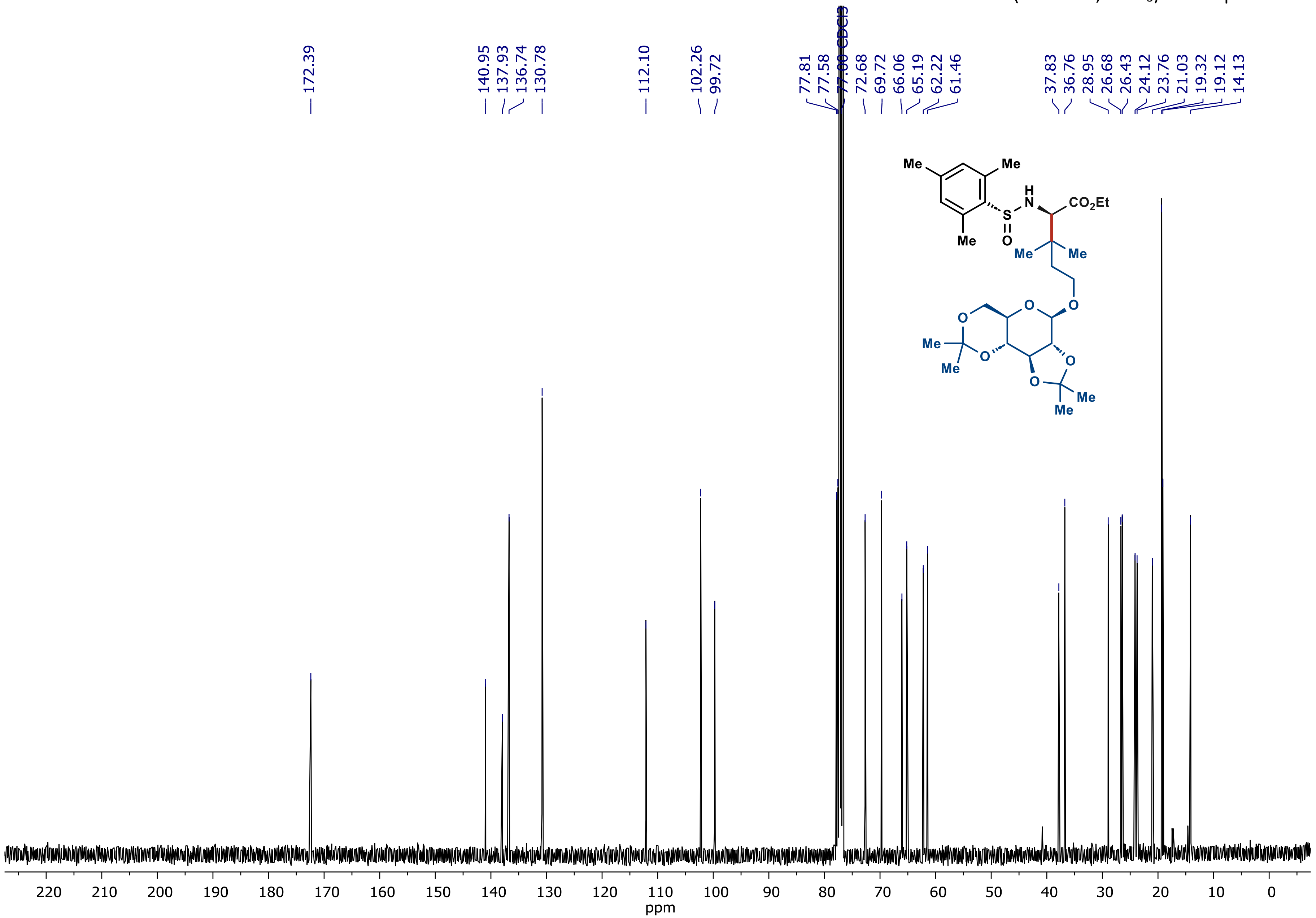

**COSY** of compound **5w**

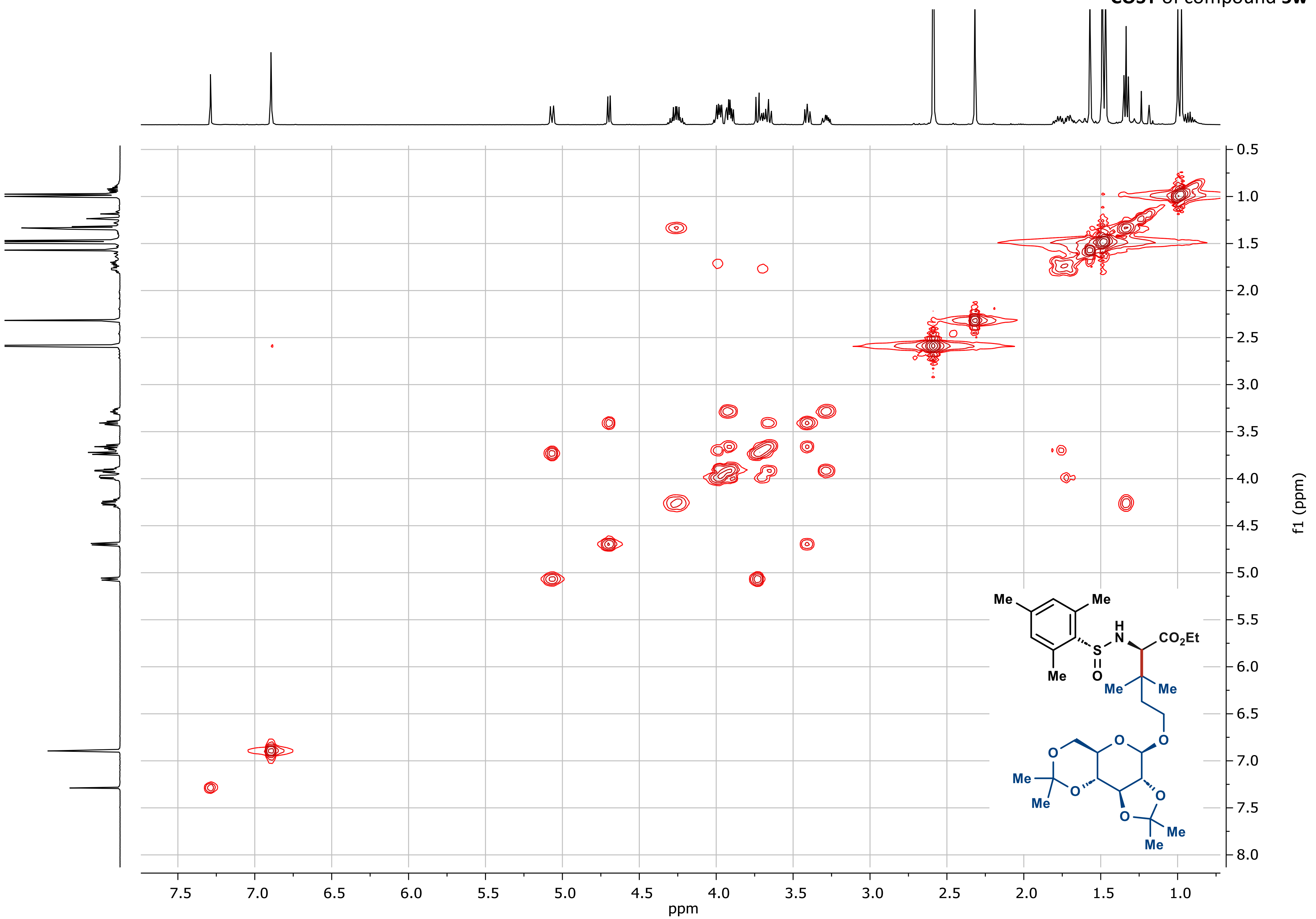

**HSQC** of compound **5w**

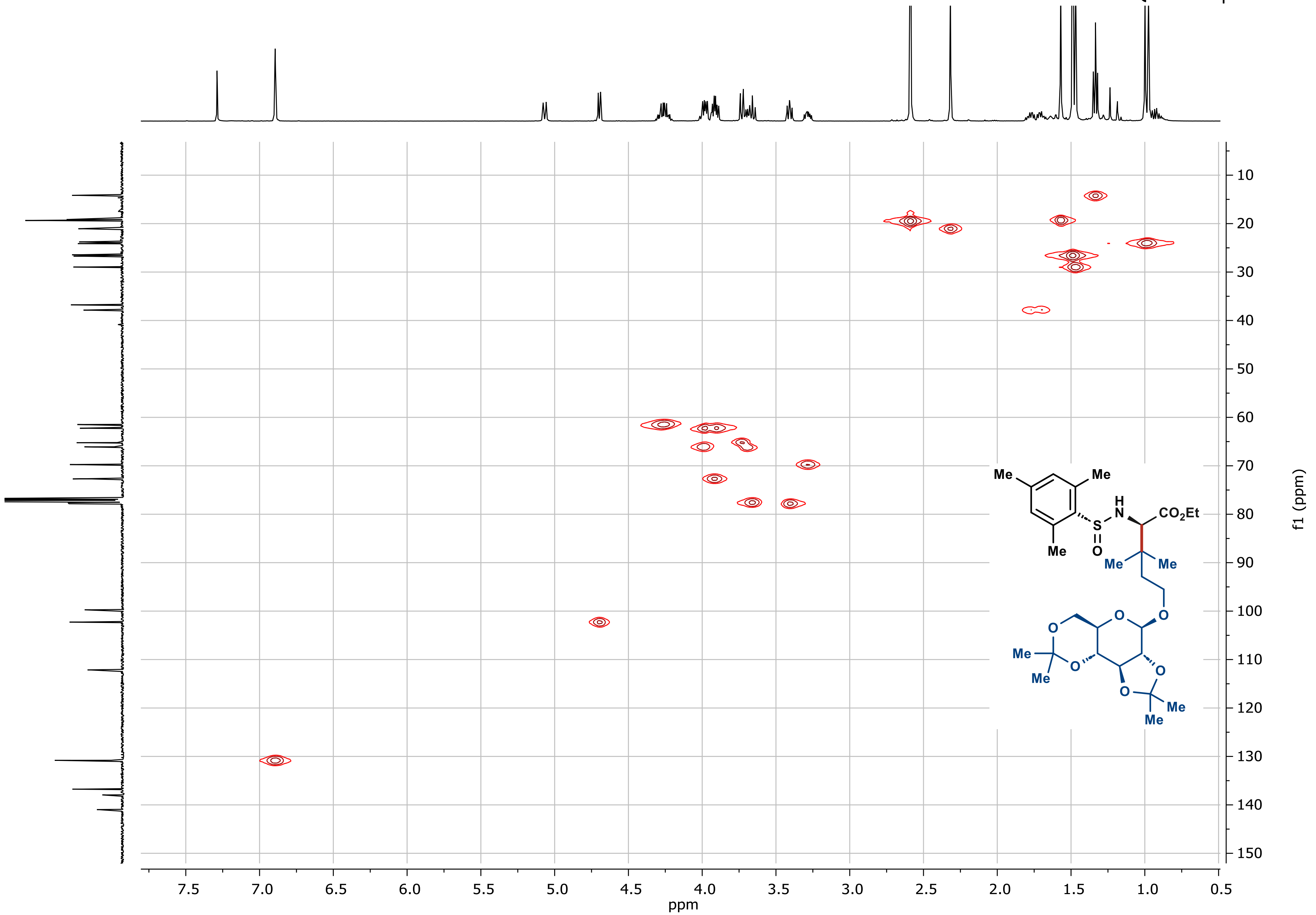

**$^{1}$H NMR** (500 MHz, $CDCl_3$) of compound **6a**

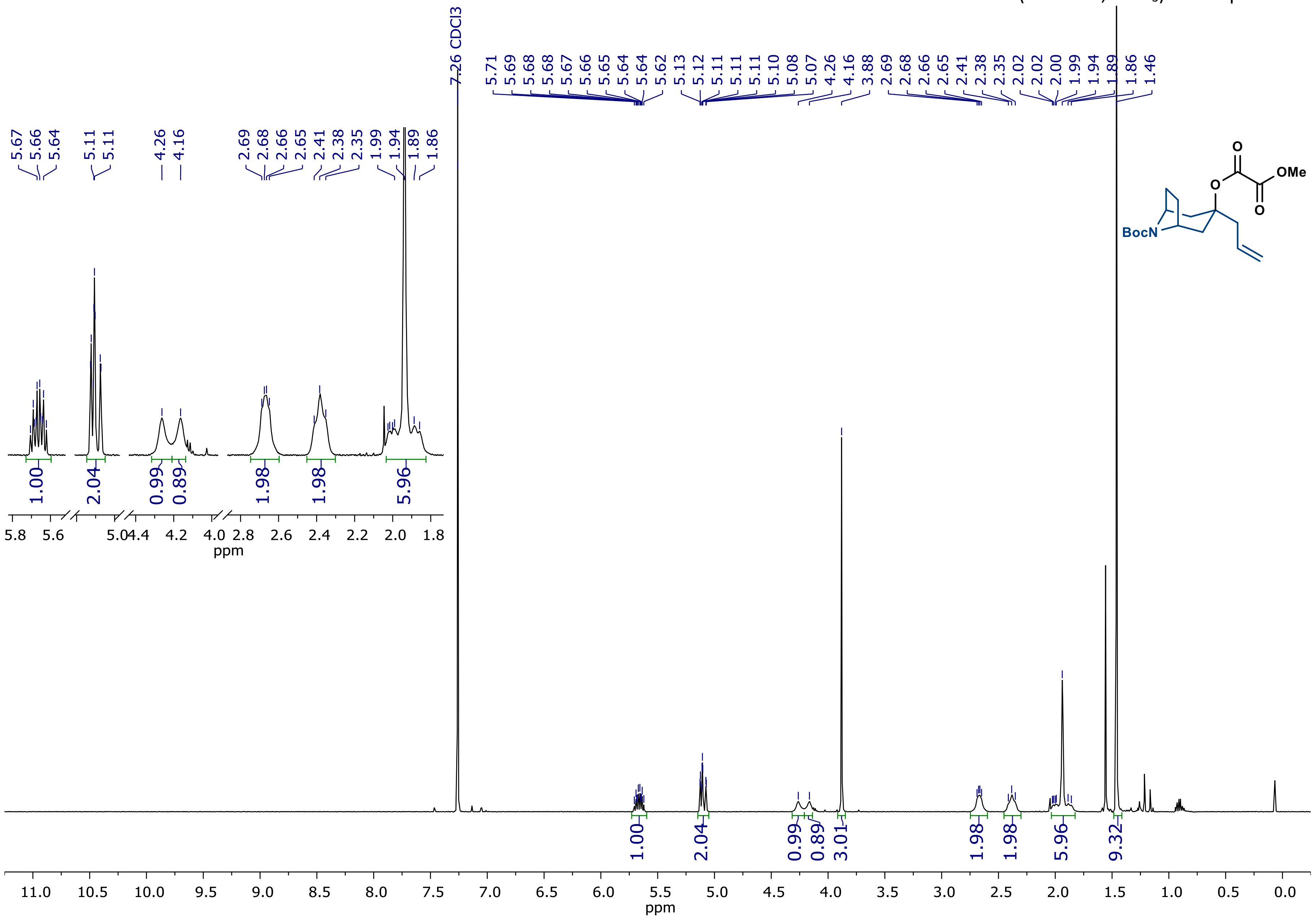

**$^{13}$C NMR** (126 MHz, $CDCl_3$) of compound **6a**

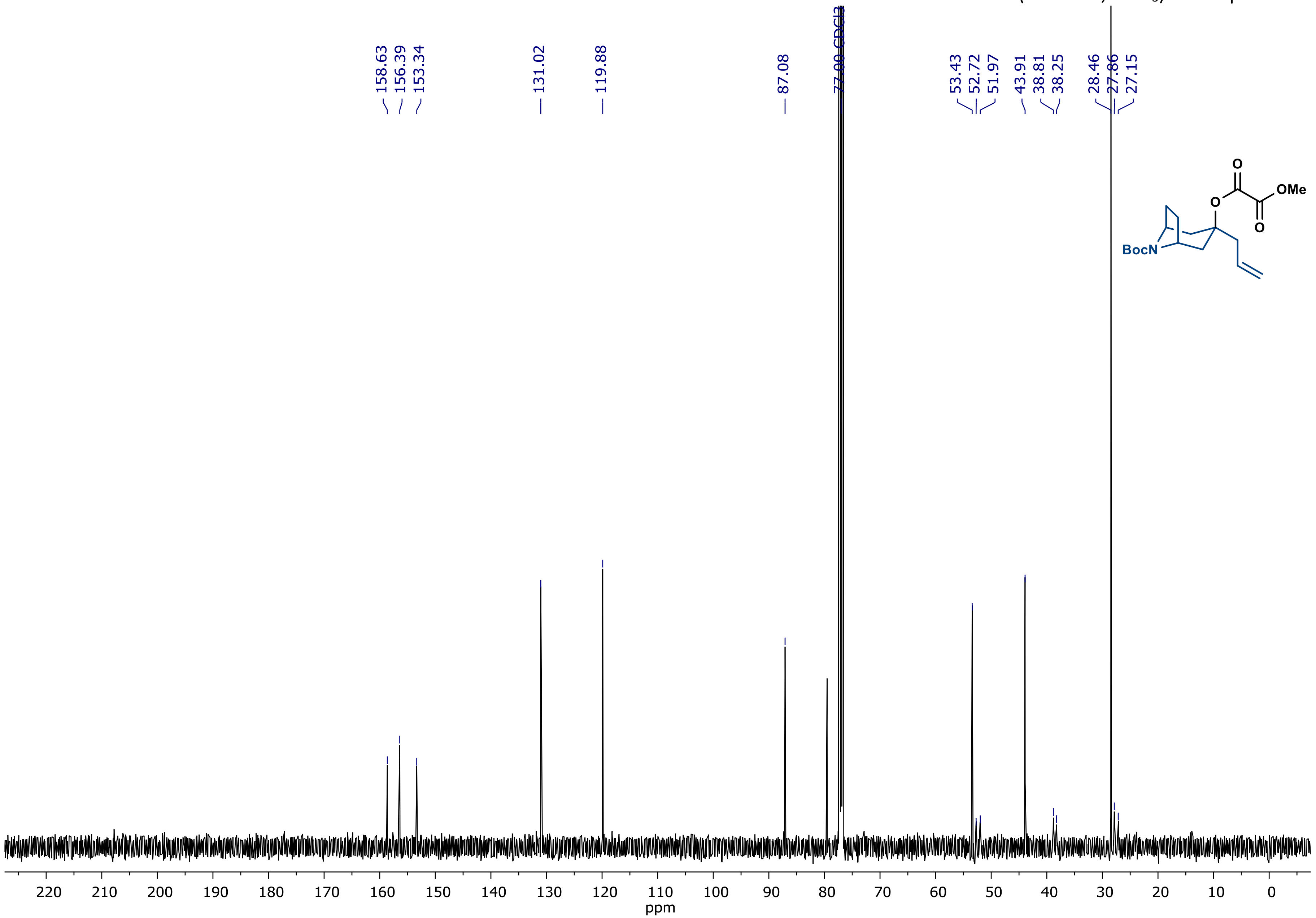

**$^1$H NMR** (500 MHz, $CDCl_3$) of compound **6b**

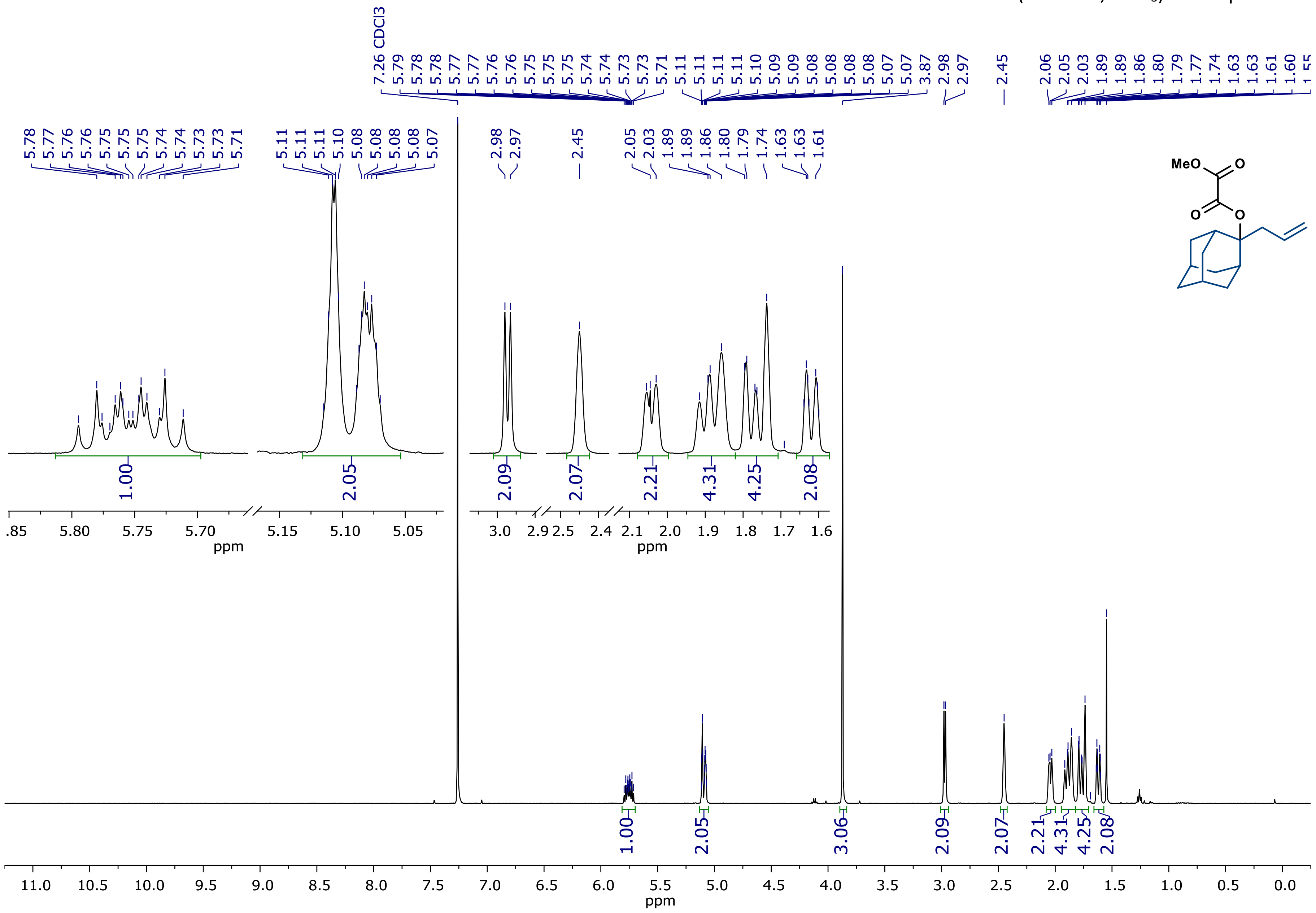

**$^{13}$C NMR** (126 MHz, $CDCl_3$) of compound **6b**

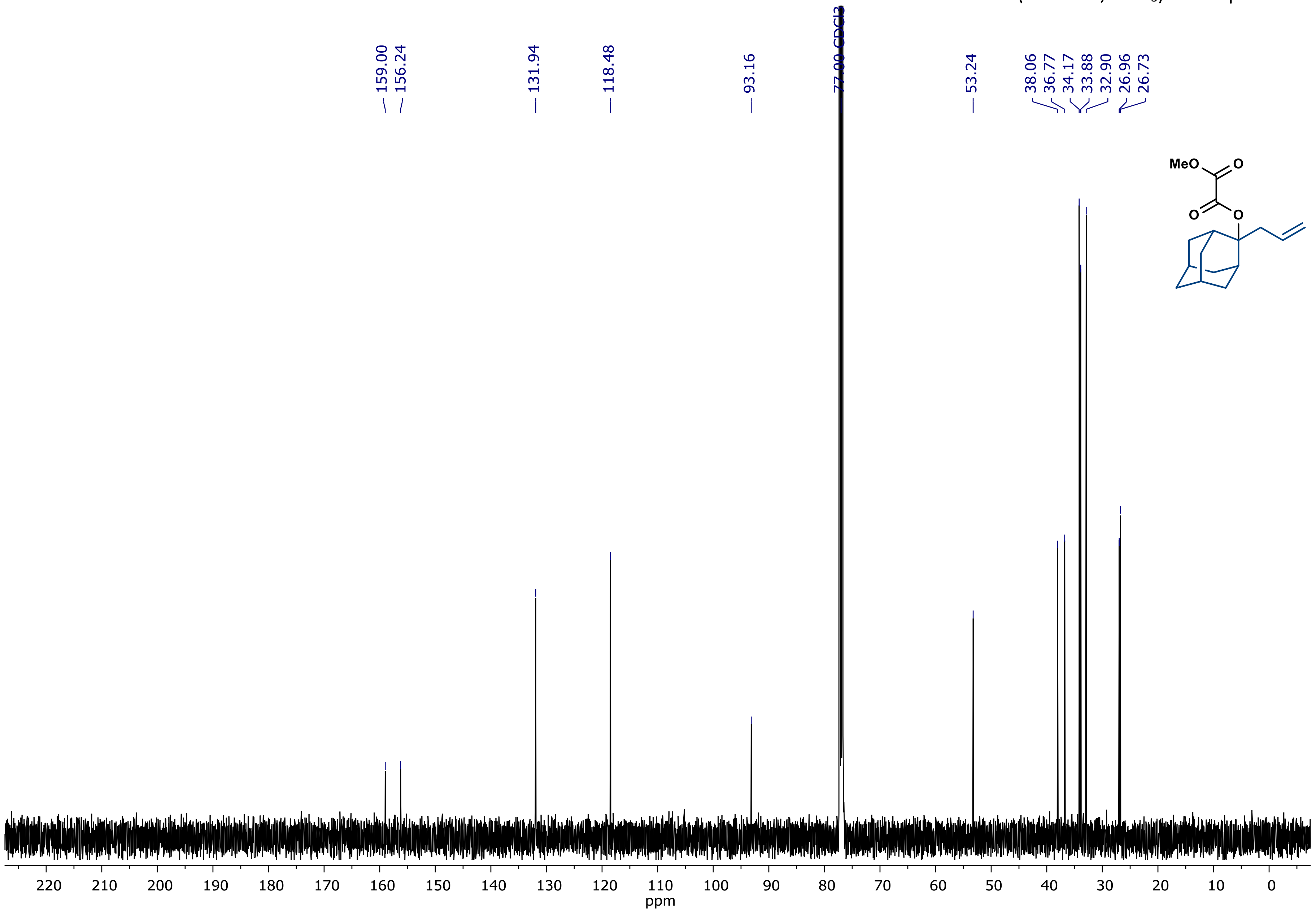

**$^{1}$H NMR** (500 MHz, $CDCl_3$) of compound **6c**

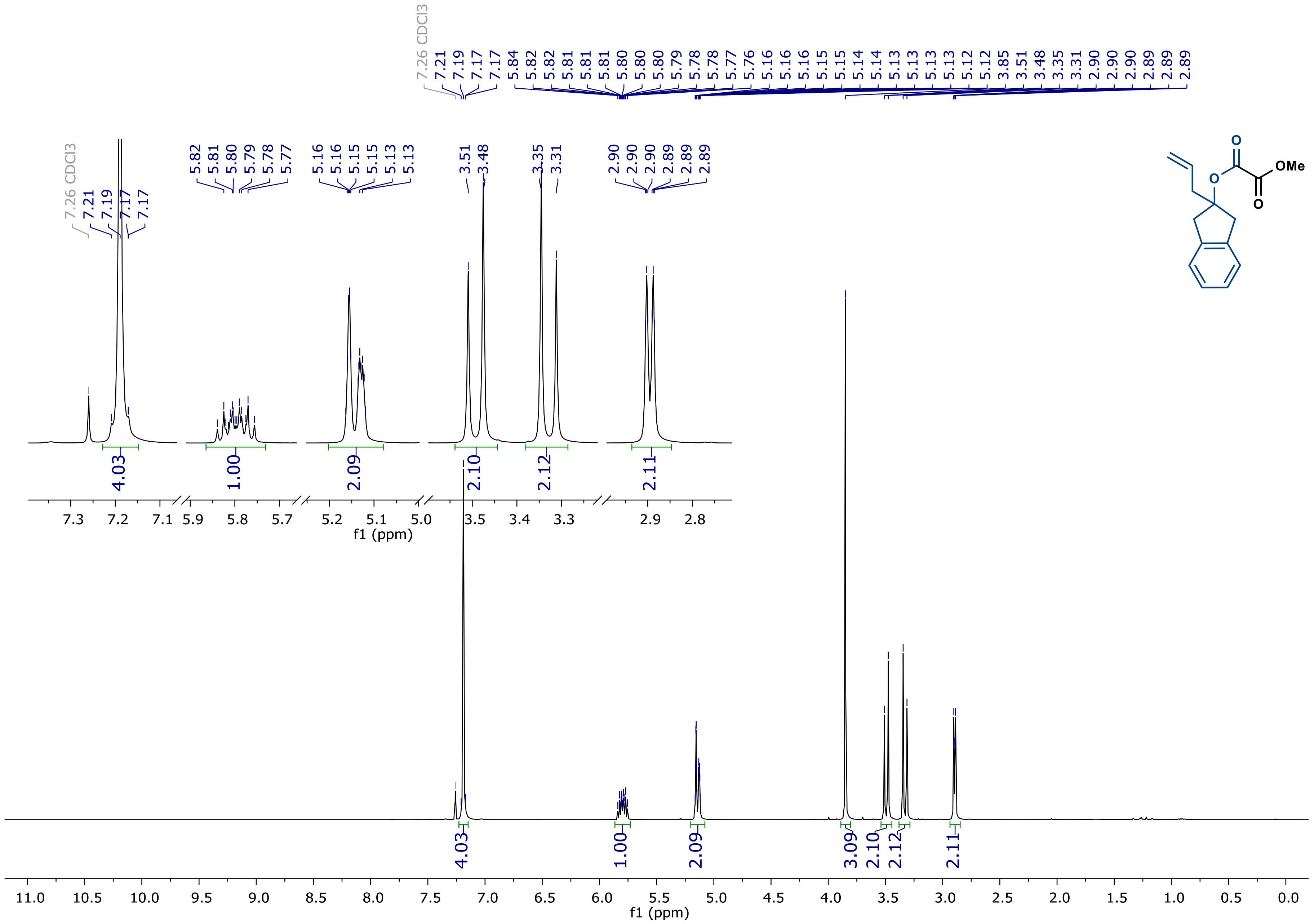

**$^{13}$C NMR** (126 MHz, $CDCl_3$) of compound **6c**

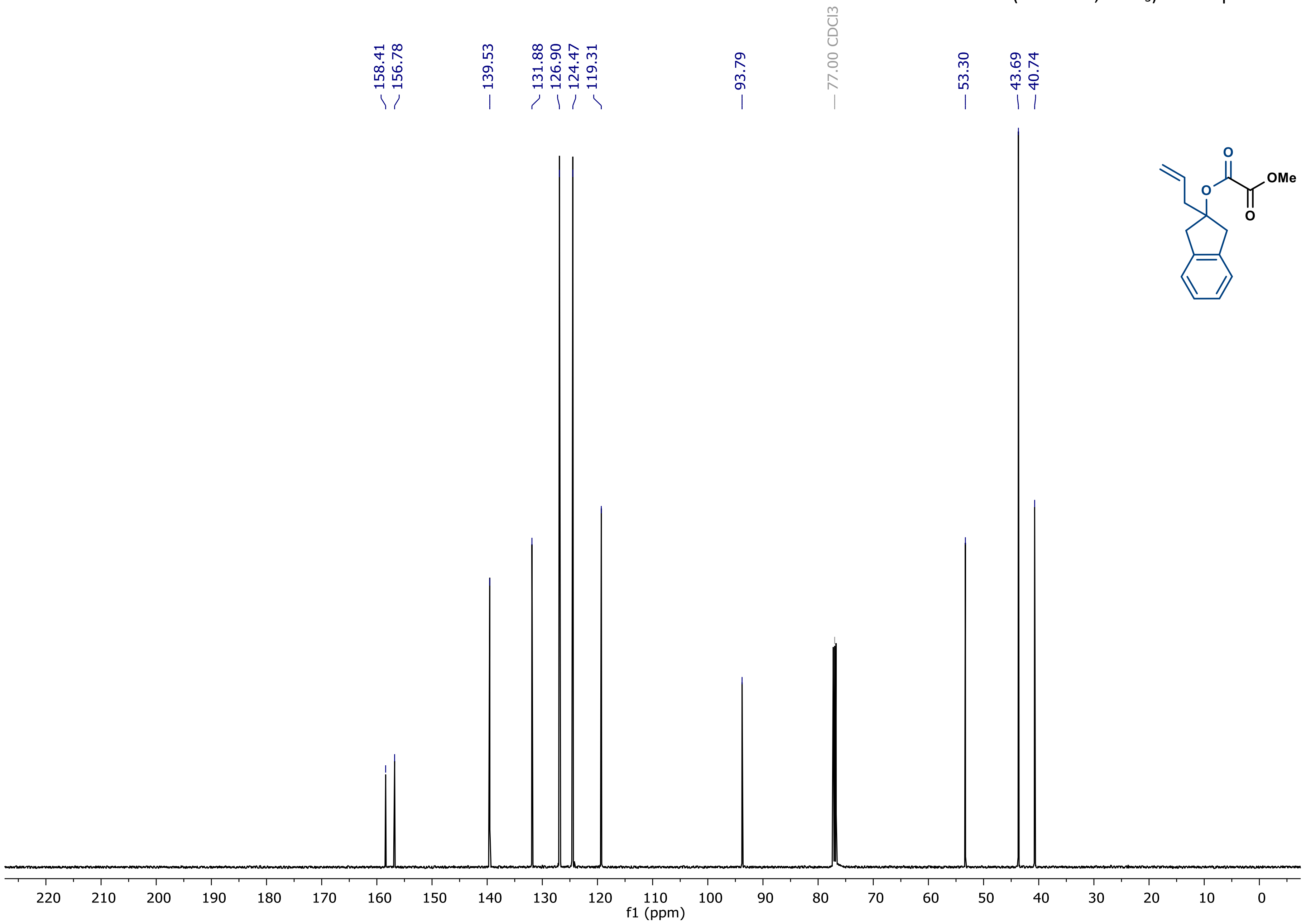

**$^1$H NMR** (500 MHz, $CDCl_3$) of compound **6d**

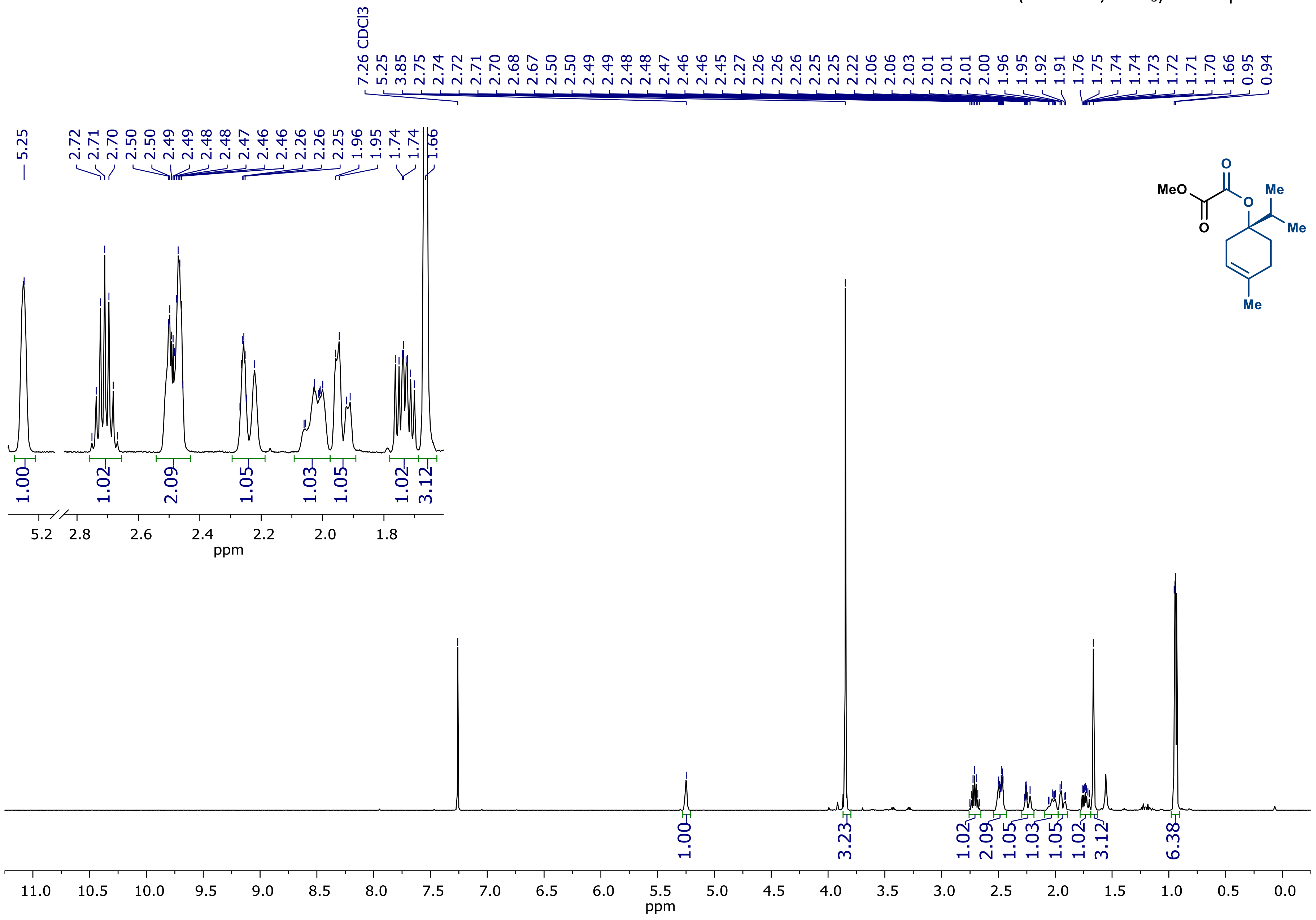

$^{13}$**C NMR** (126 MHz, $CDCl_3$) of compound **6d**

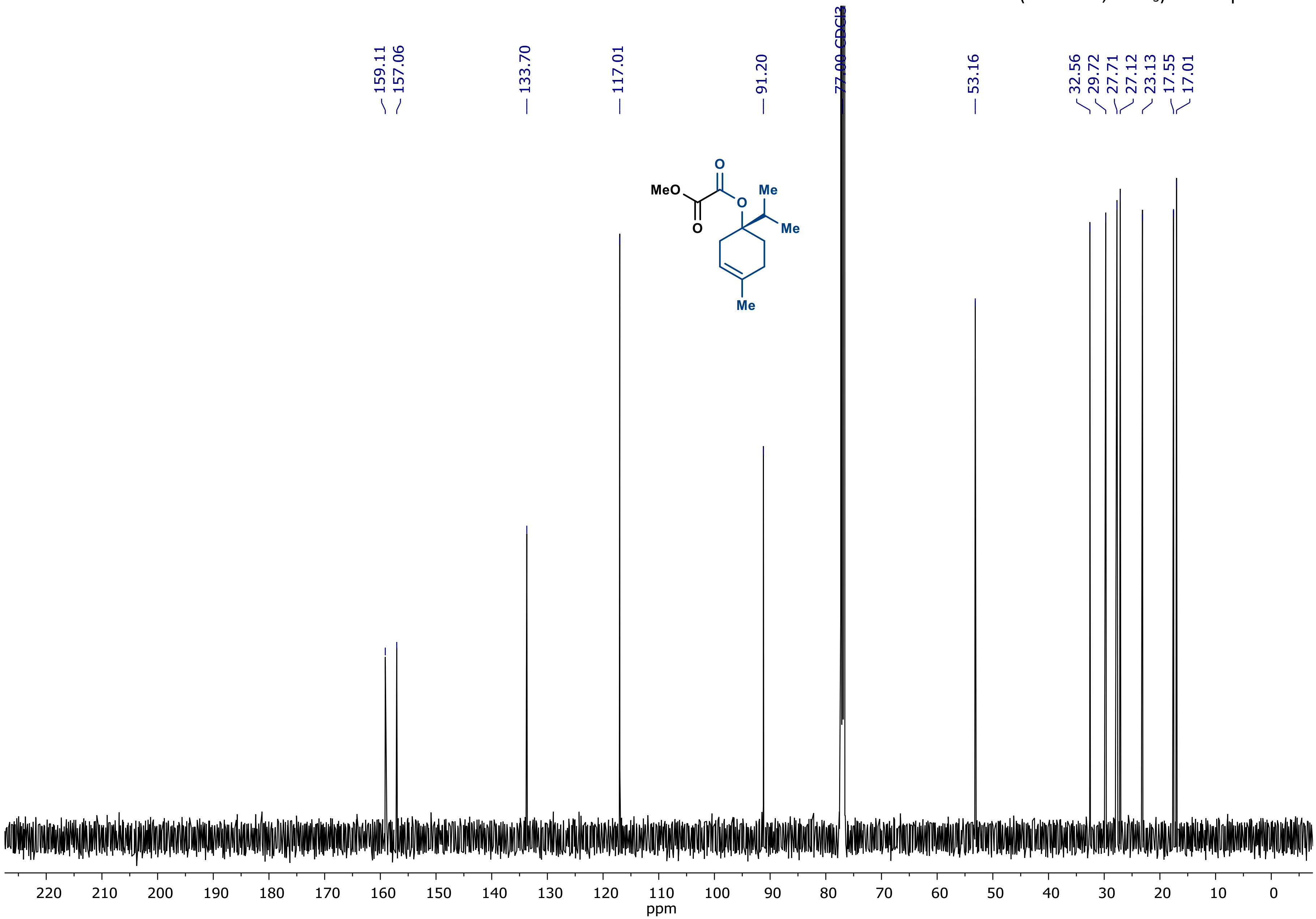

**$^1$H NMR** (500 MHz, $CDCl_3$) of compound **7a**

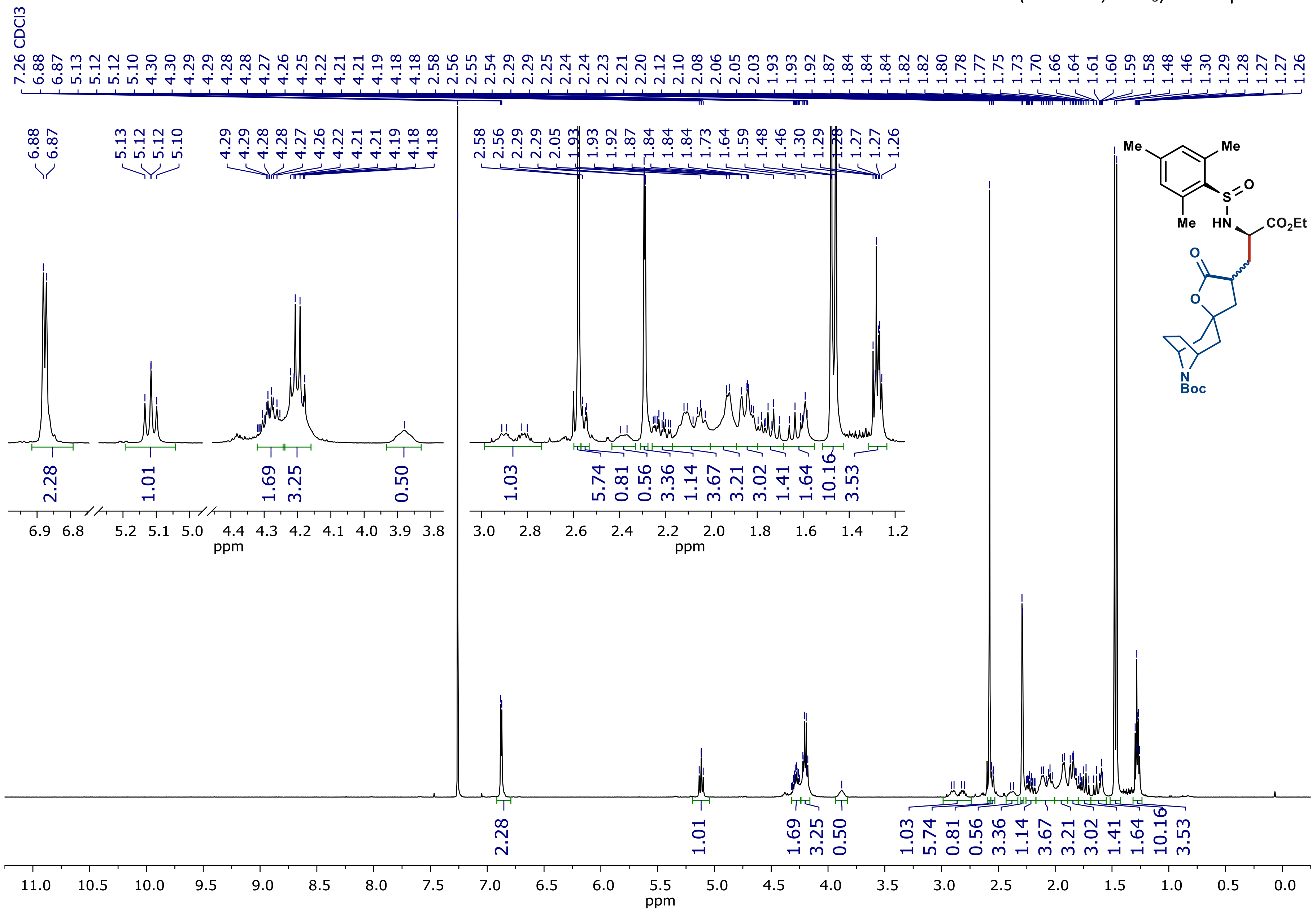

**$^{13}$C NMR** (126 MHz, $CDCl_3$) of compound **7a**

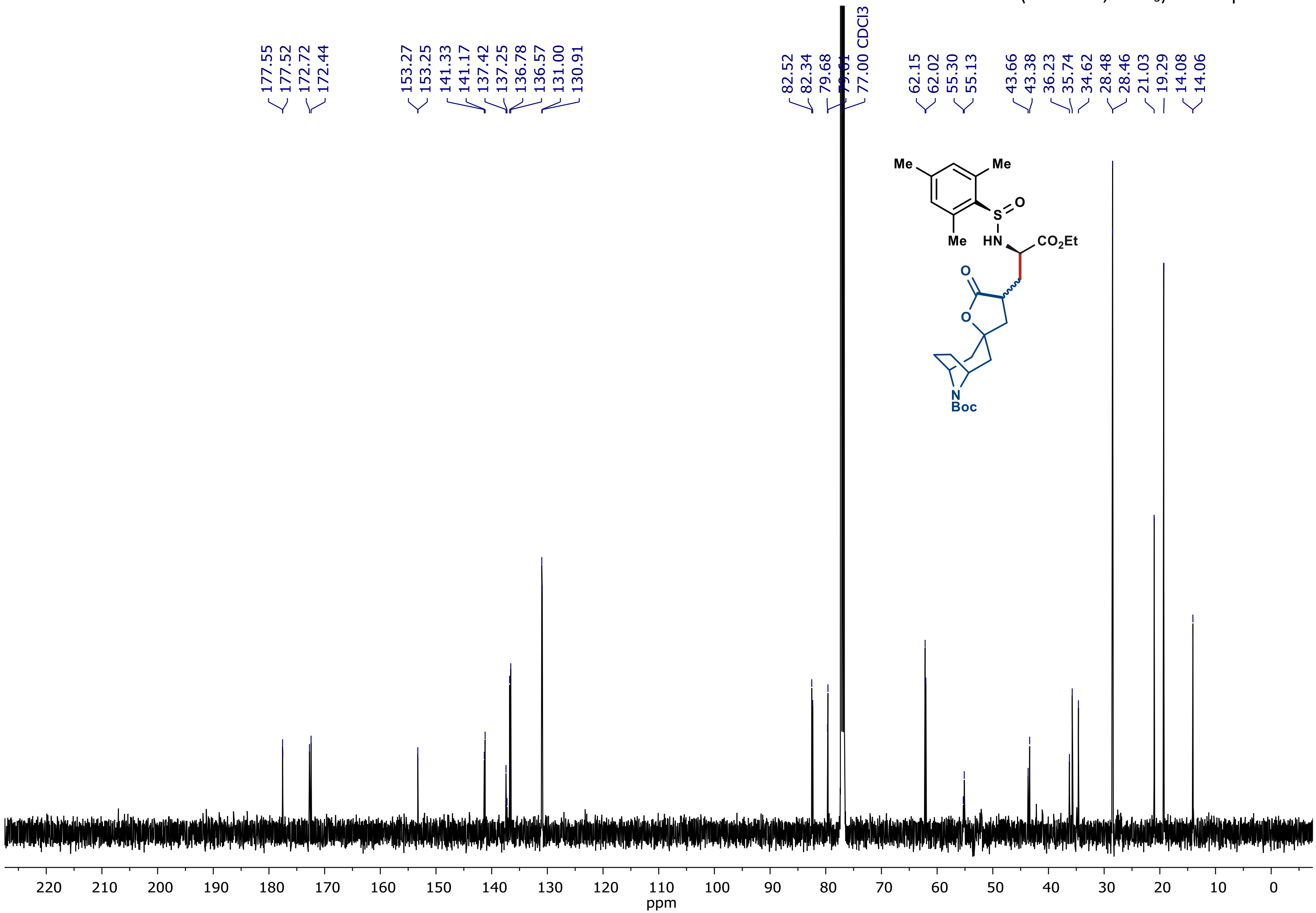

**COSY** of compound **7a**

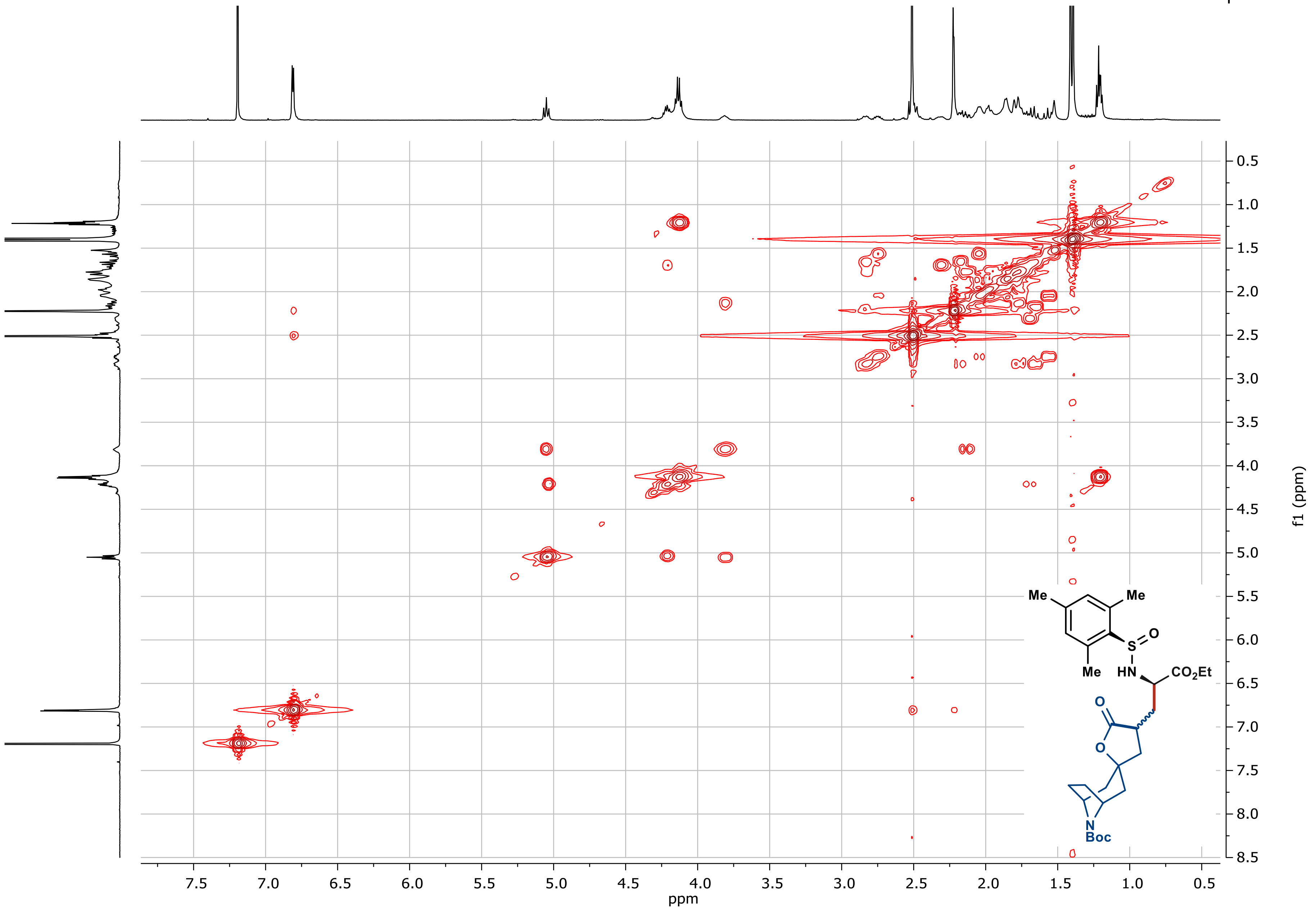

**HSQC** of compound **7a**

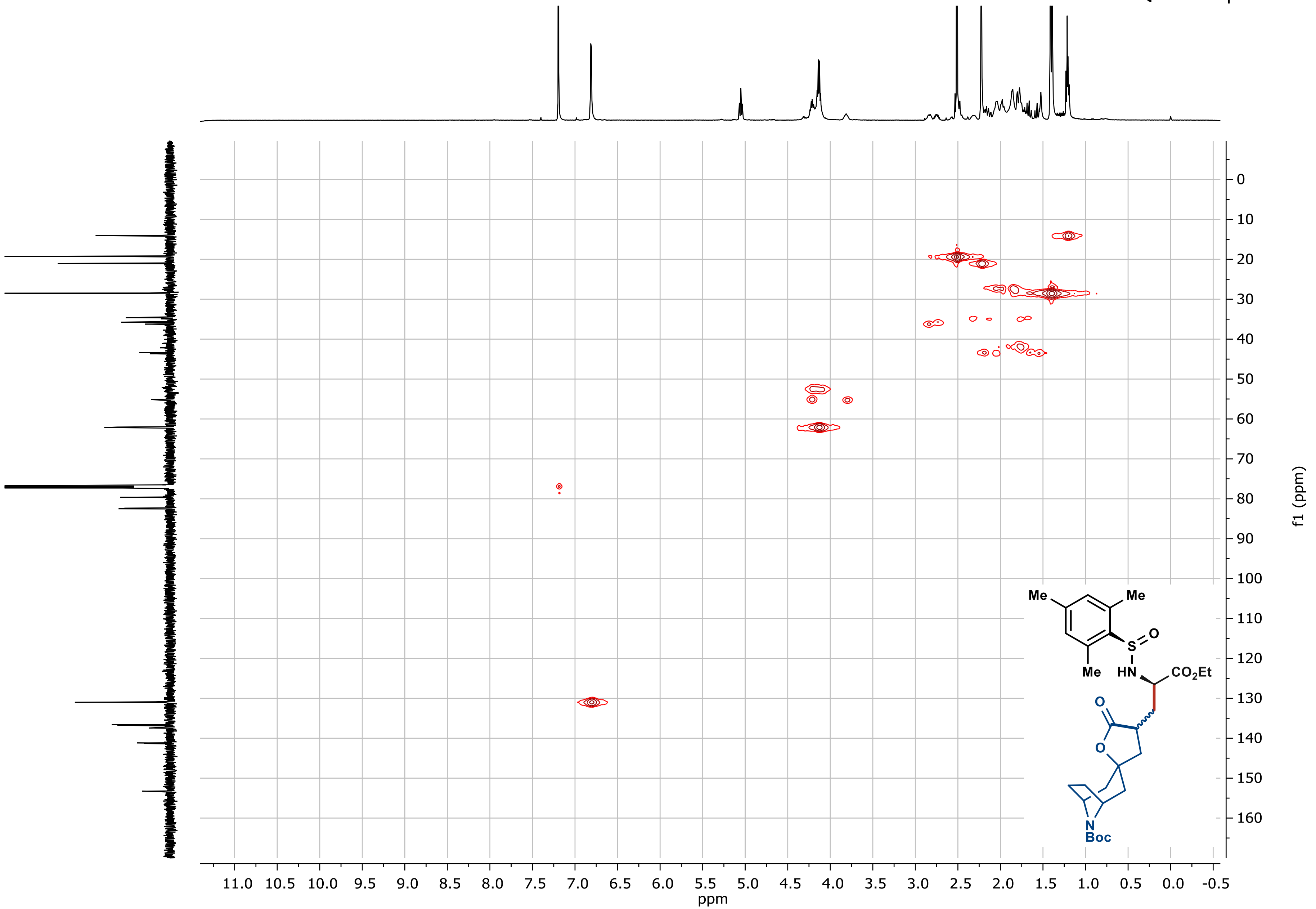

**$^{1}$H NMR** (500 MHz, $CDCl_3$) of compound **7b**

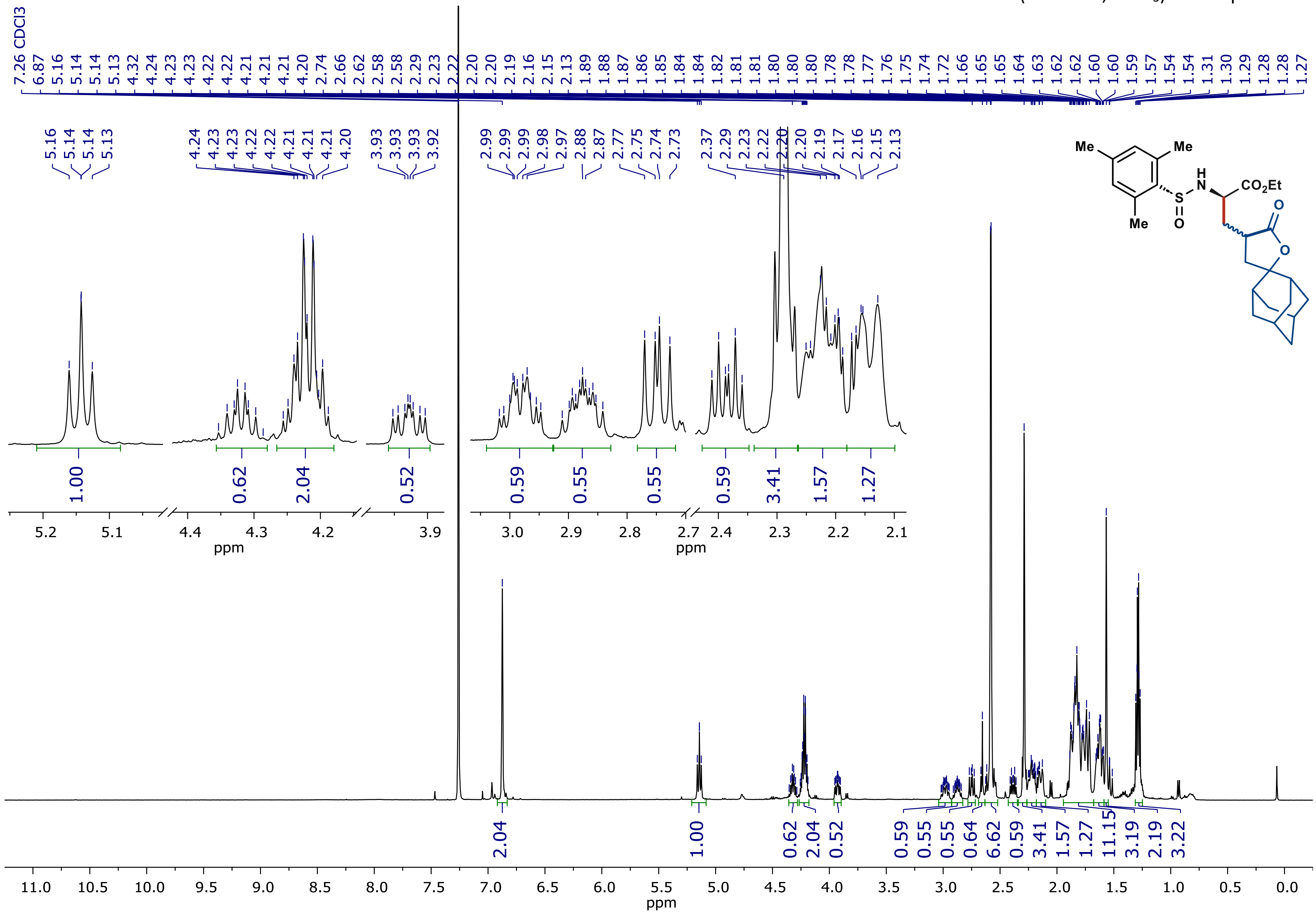

$^{13}$**C NMR** (126 MHz, $CDCl_3$) of compound **7b**

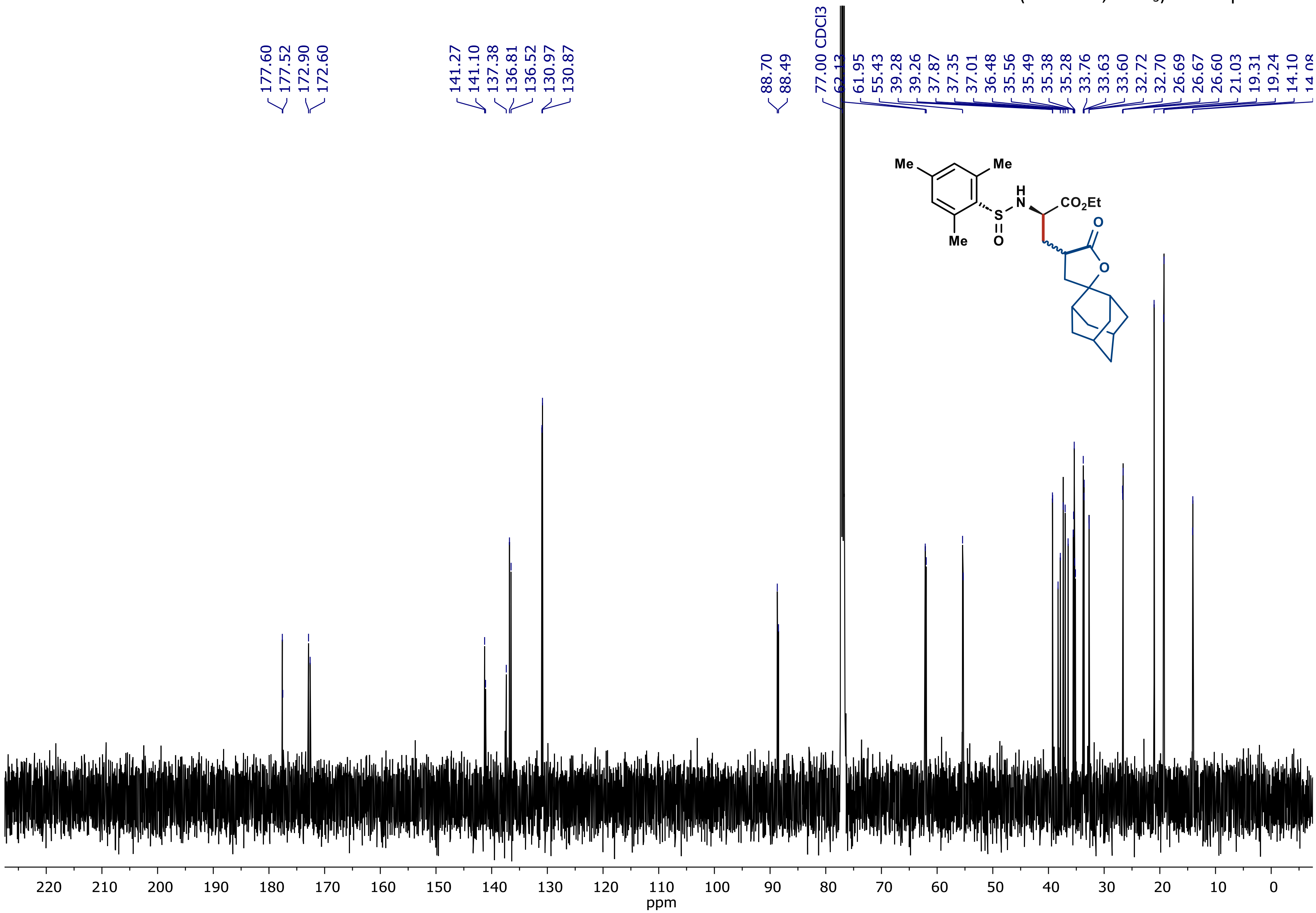

**COSY** of compound **7b**

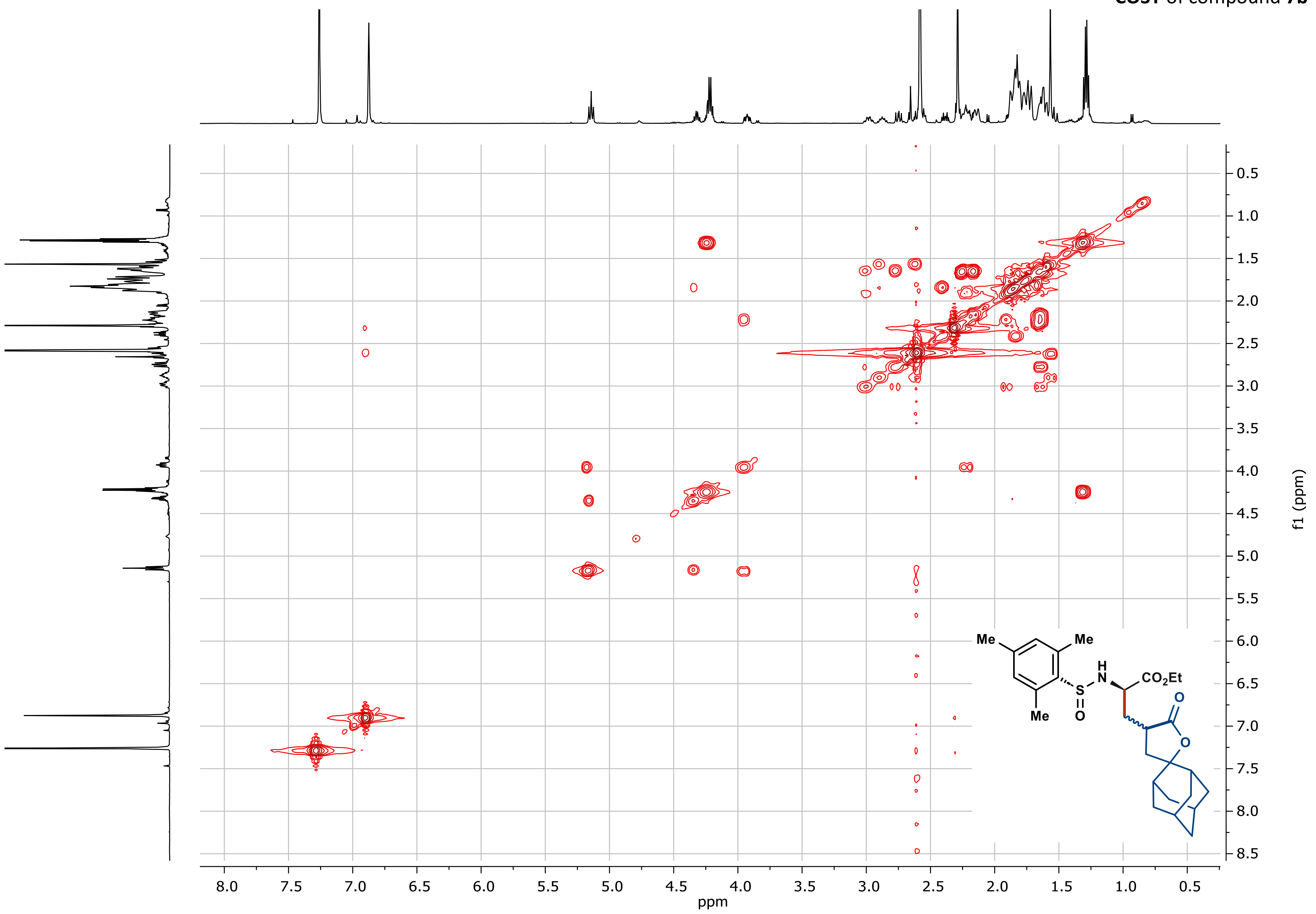

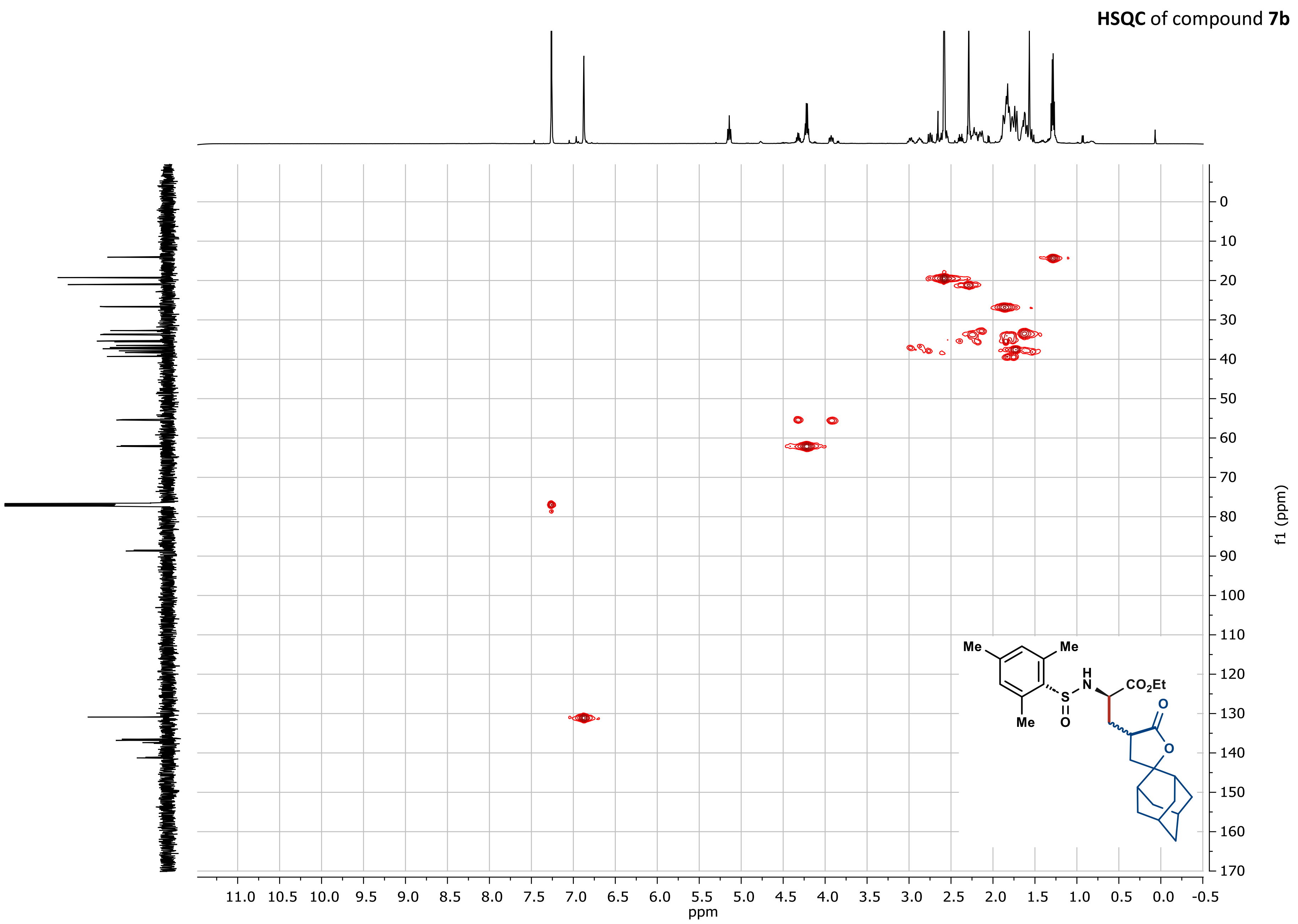
HSQC of compound 7b
ppm
f1 (ppm)
Me
Me
Me
H
N
S
O
CO2Et
O
O

$^{1}$H NMR (500 MHz, $CDCl_3$) of compound **7c**

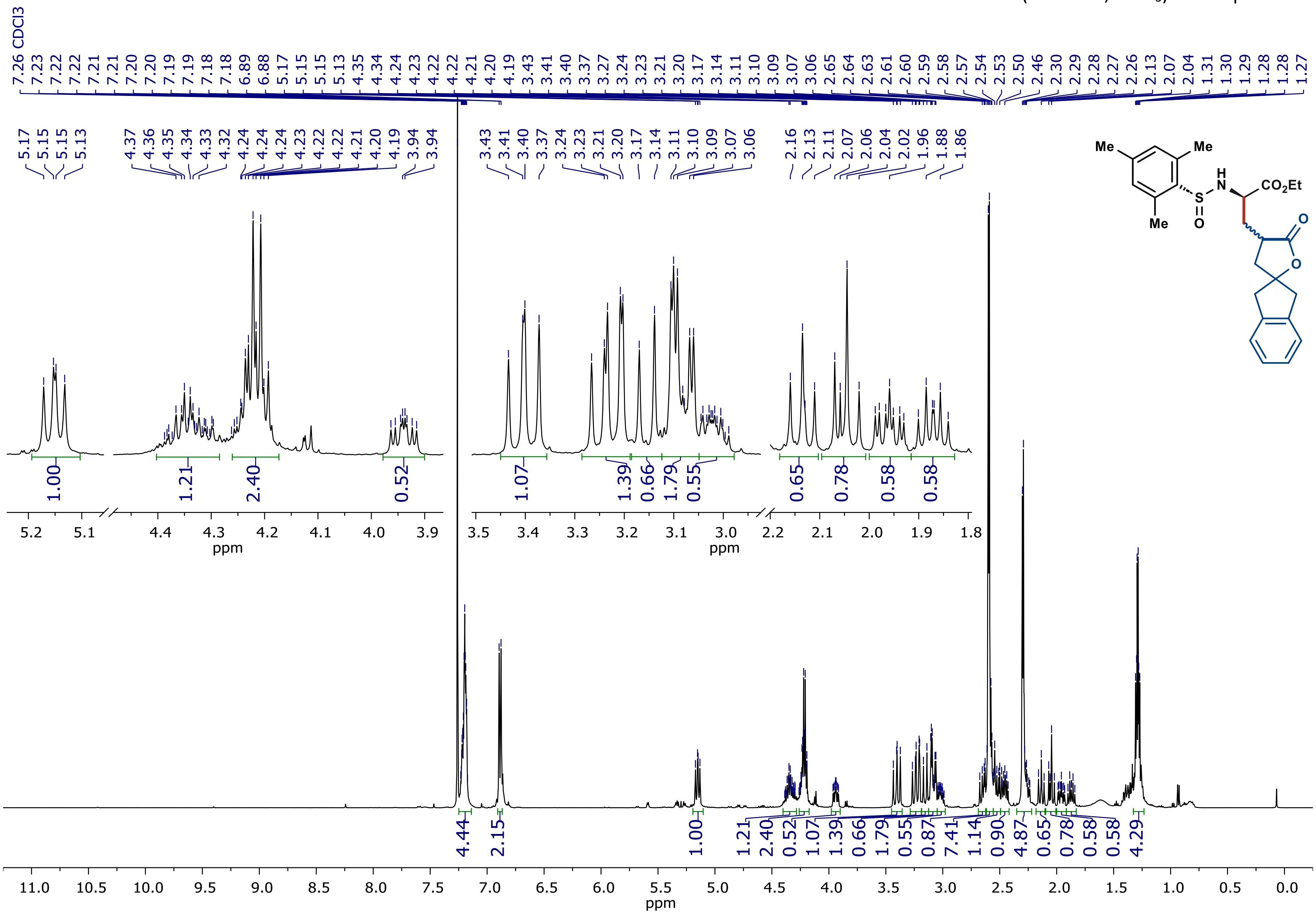

$^{13}$**C NMR** (126 MHz, $CDCl_3$) of compound **7c**

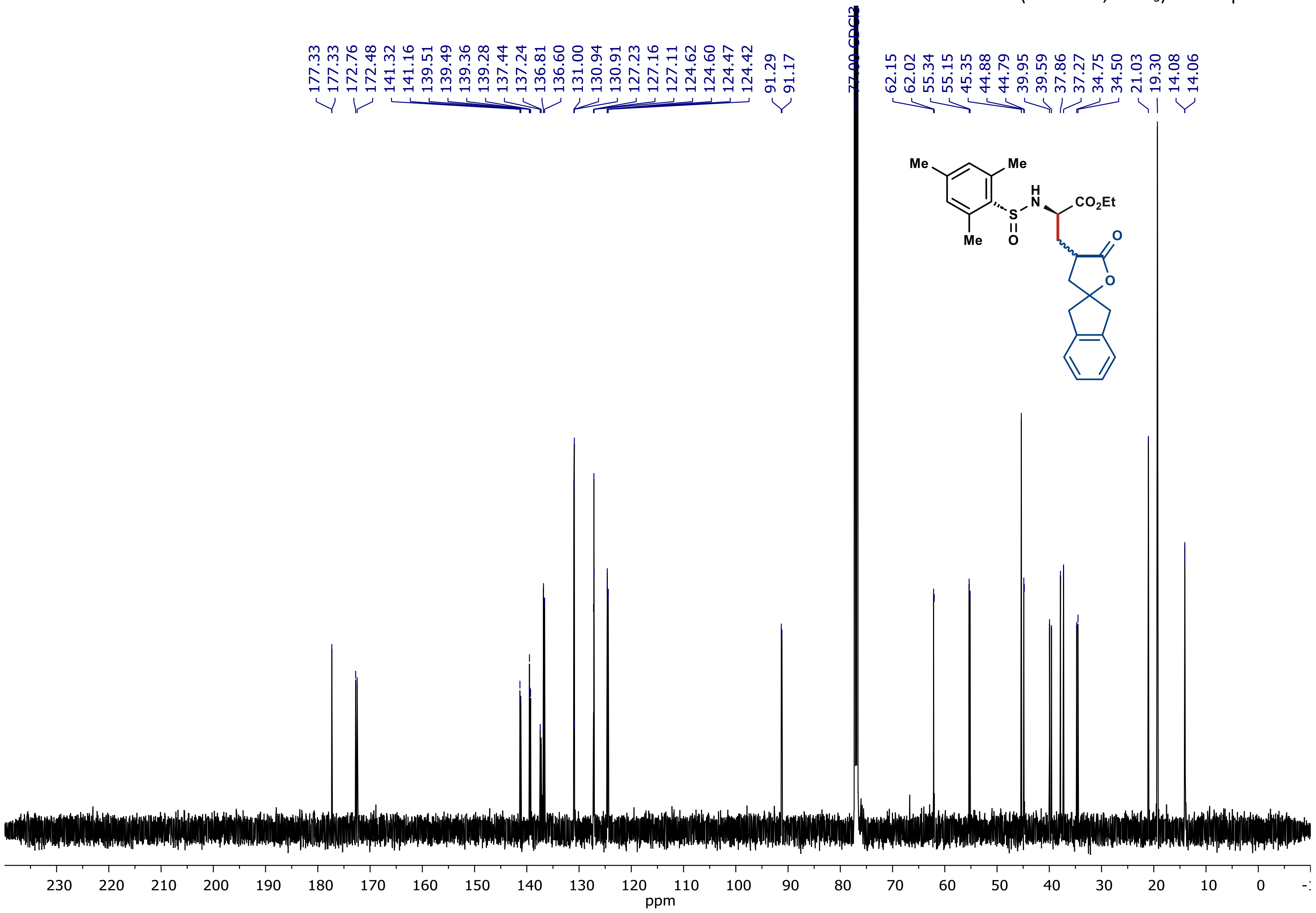

**COSY** of compound **7c**

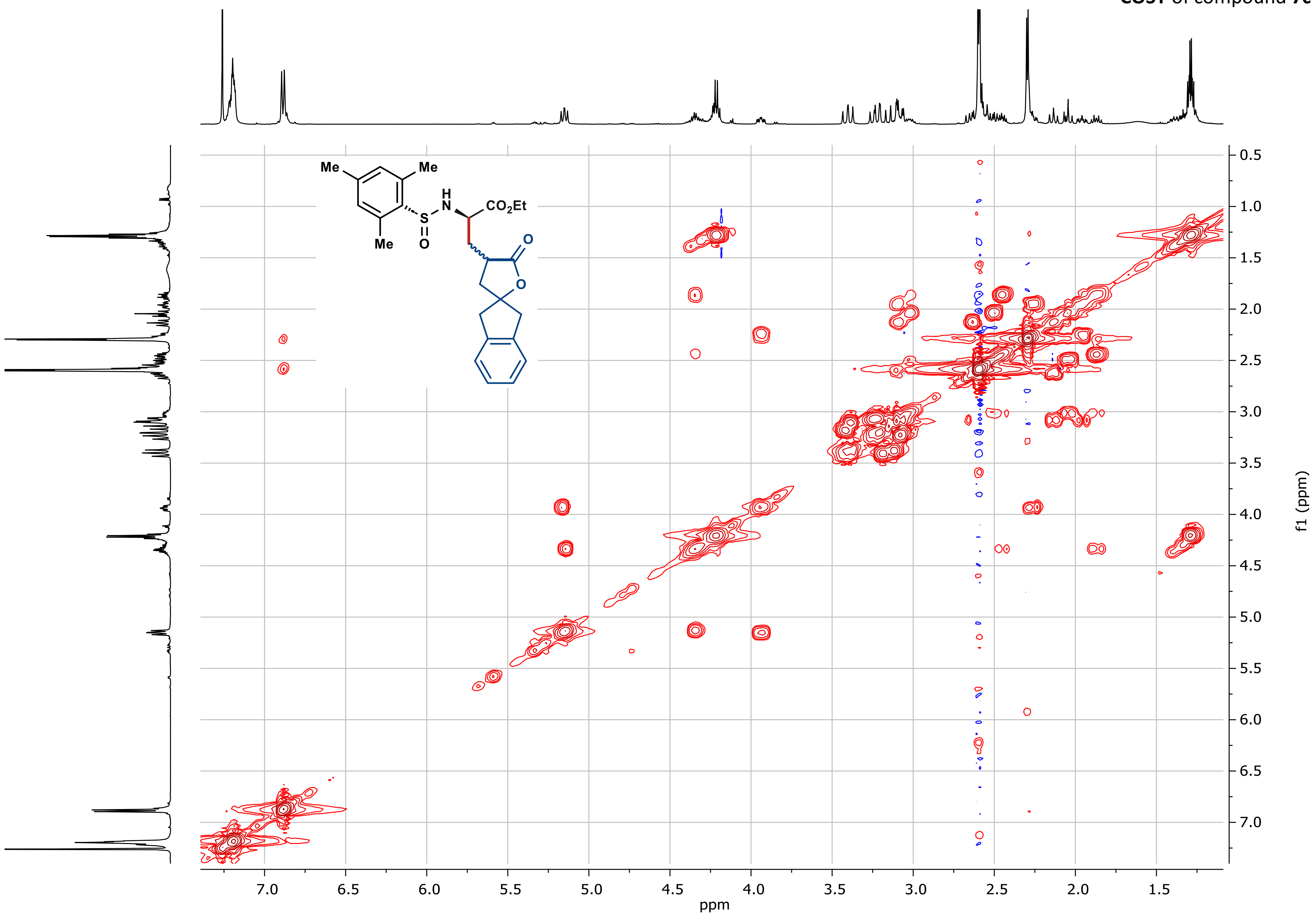

HSQC of compound **7c**

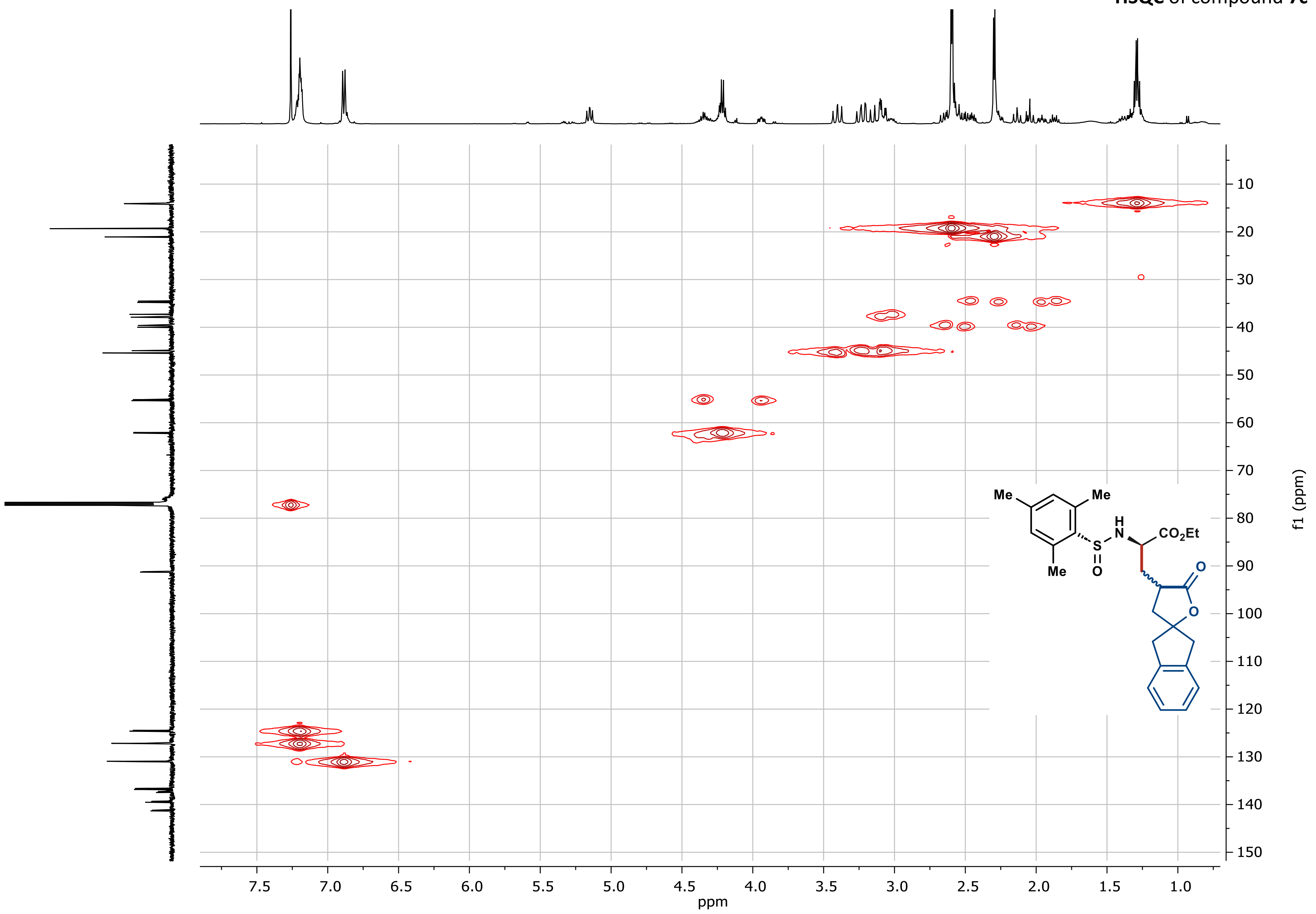

**$^1$H NMR** (500 MHz, $CDCl_3$) of compound **7d**

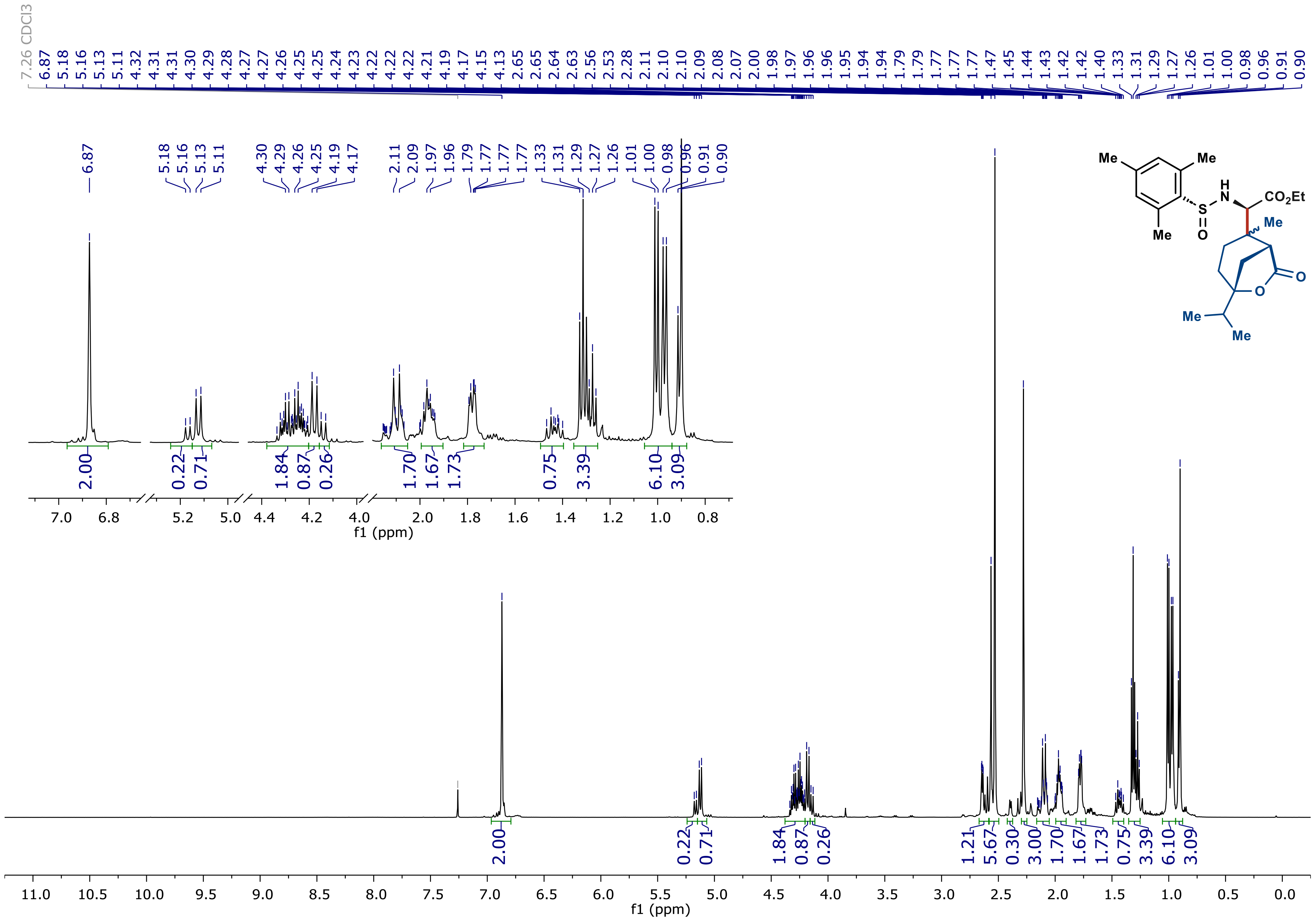

**$^{13}$C NMR** (126 MHz, $CDCl_3$) of compound **7d**

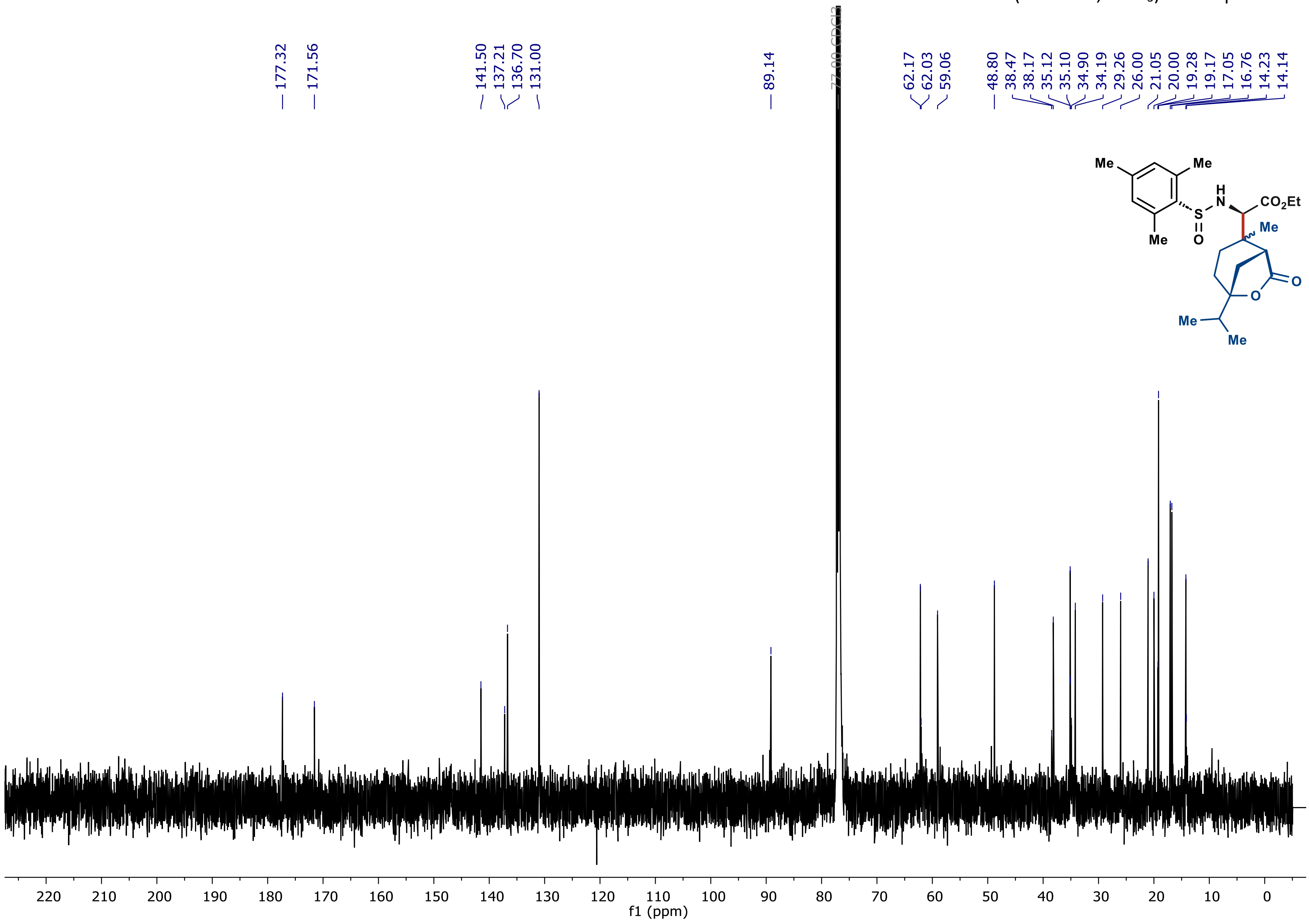

**COSY** of compound **7d**

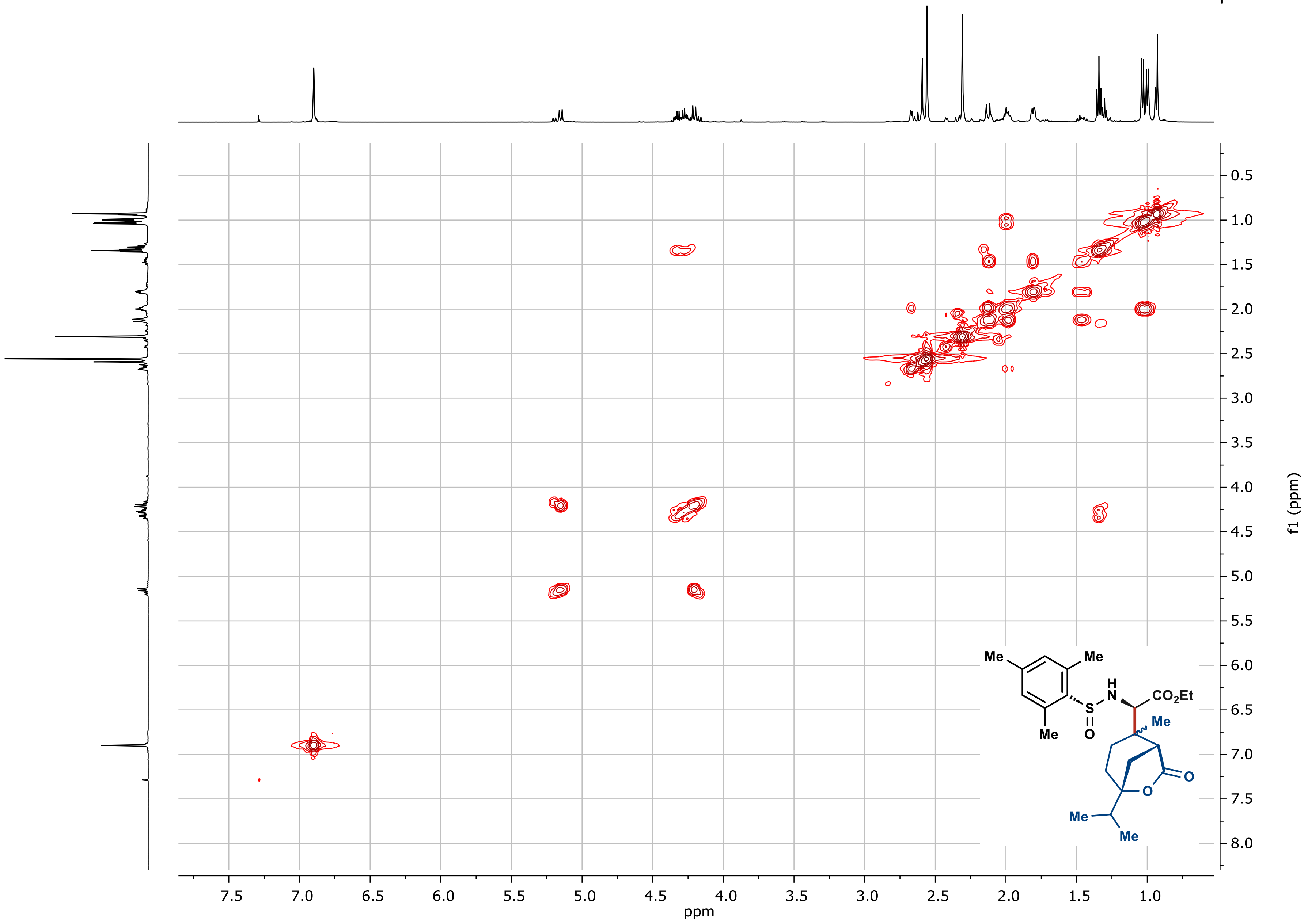

**HSQC** of compound **7d**

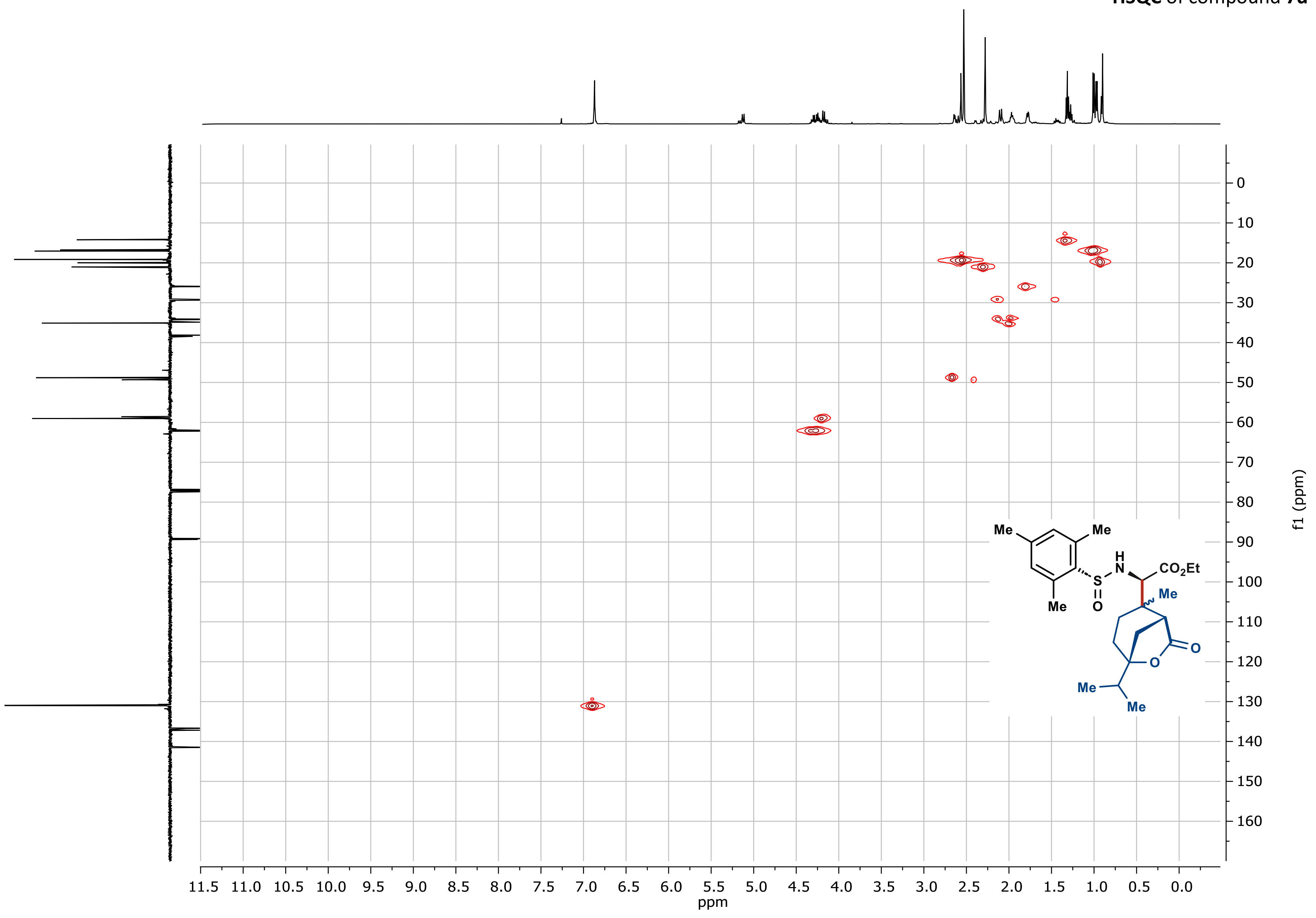

**HMBC** of compound **7d**

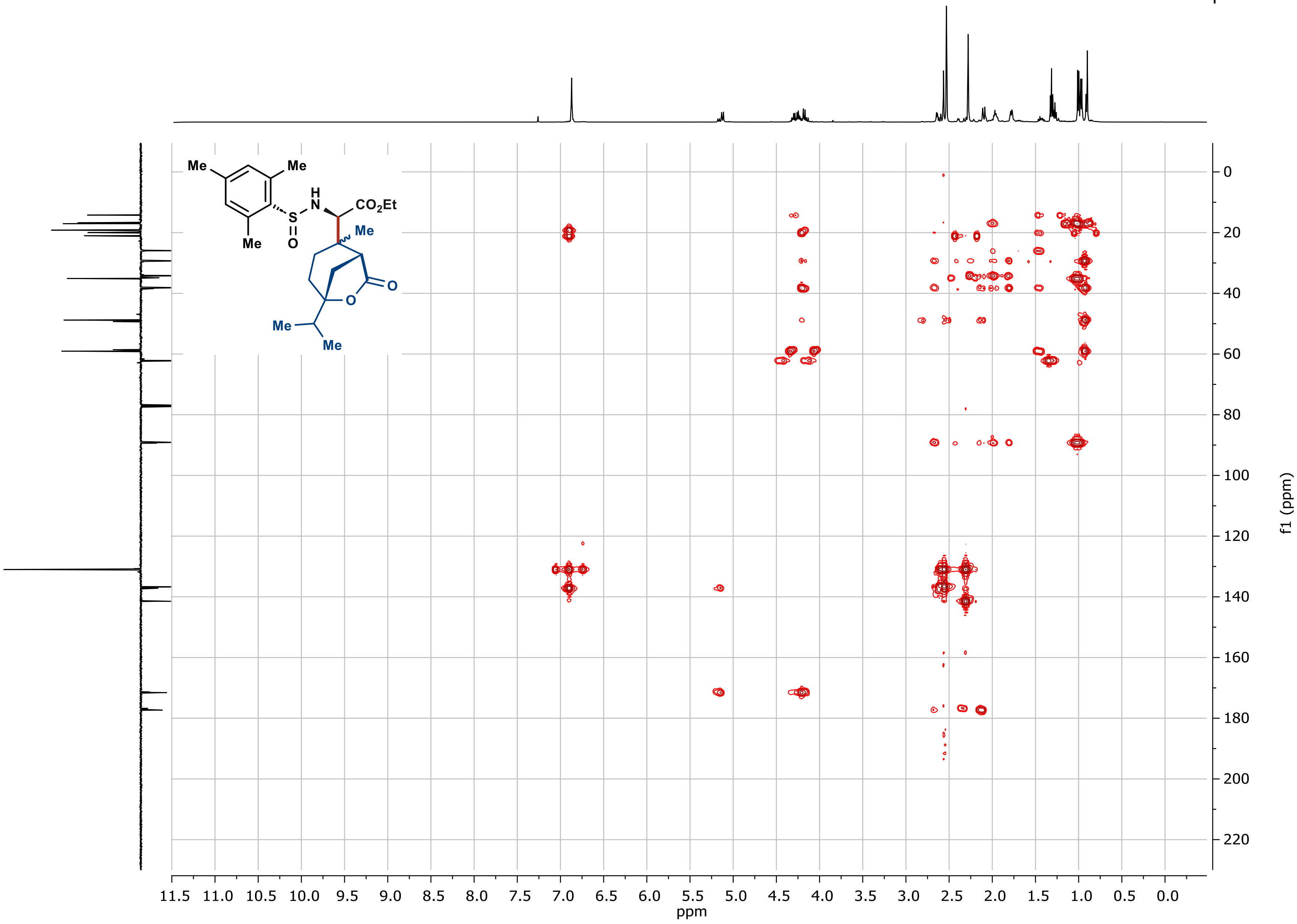

$^{1}$H NMR (500 MHz, MeOD) of compound **8a**

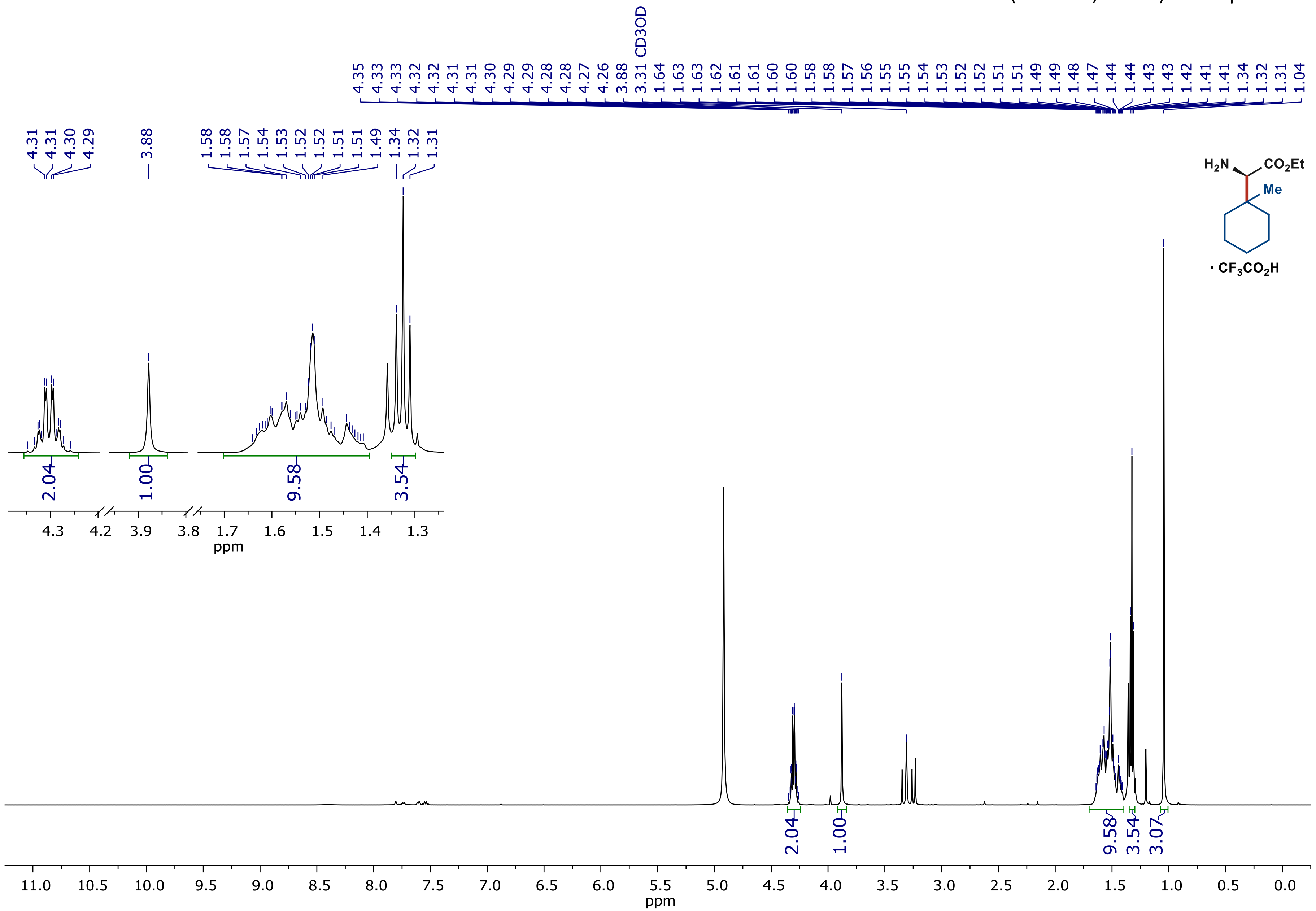

**$^{13}$C NMR** (126 MHz, MeOD) of compound **8a**

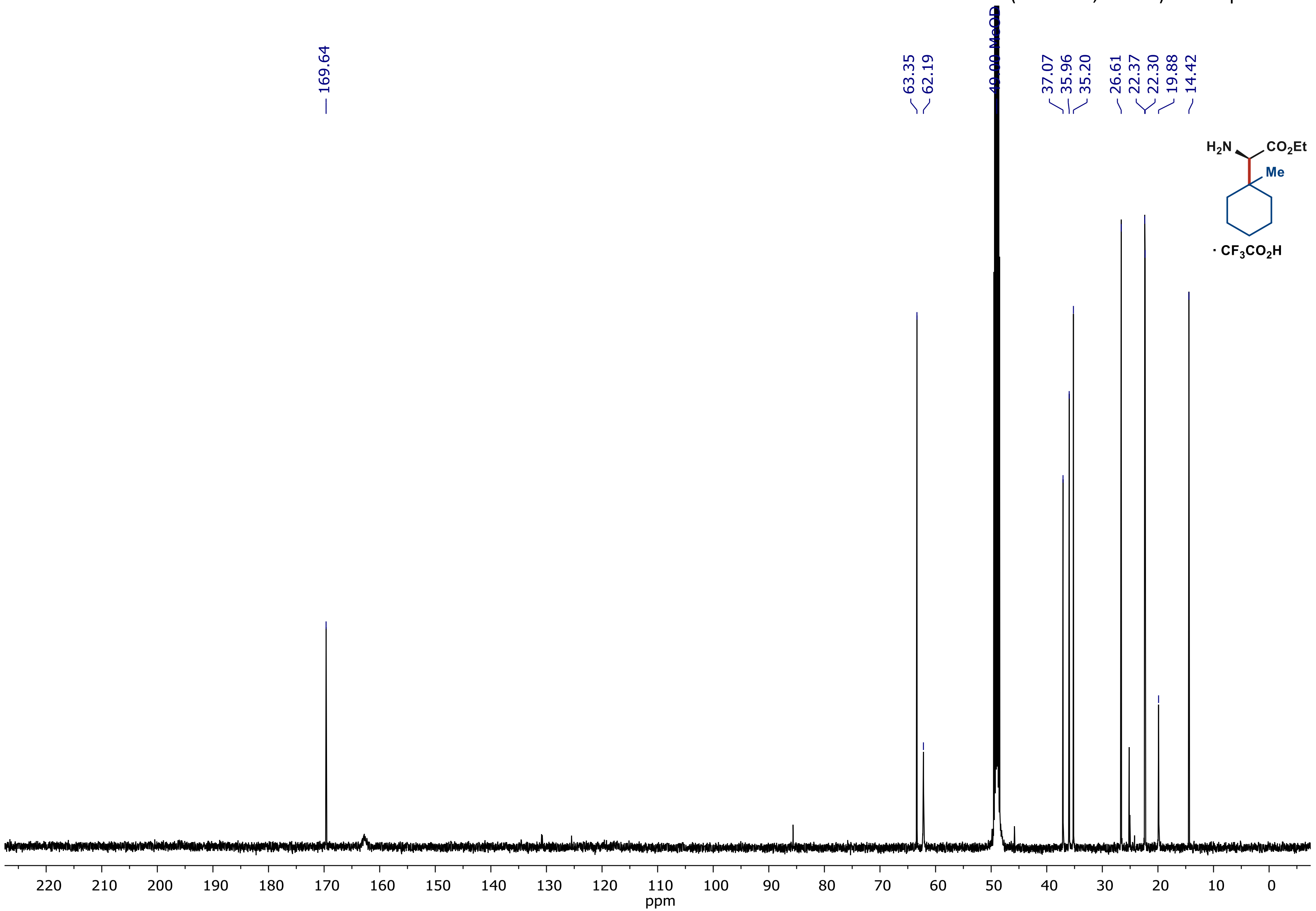

**$^{19}$F NMR** (377 MHz, MeOD) of compound **8a**

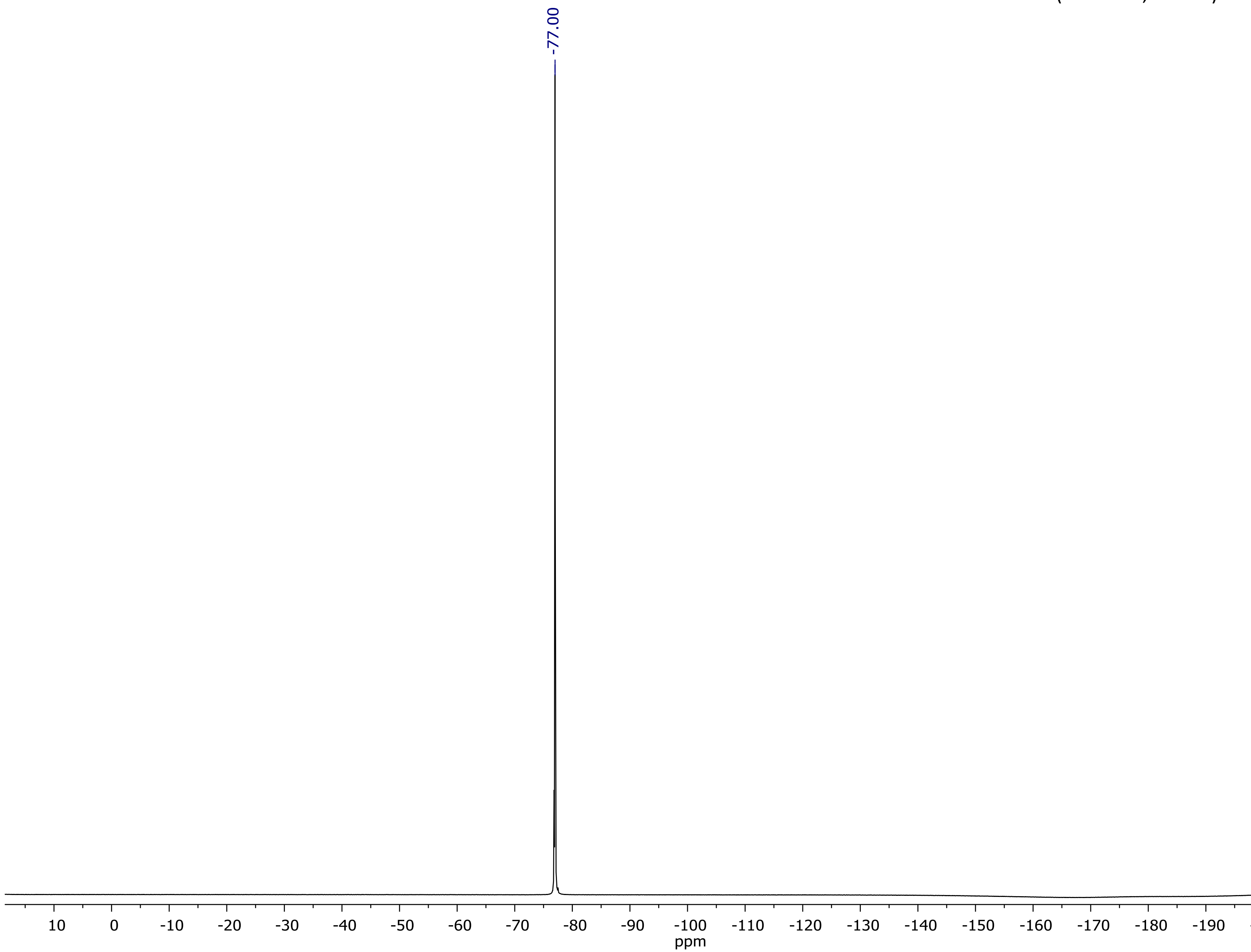


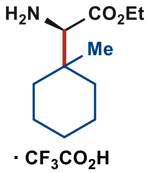

$^{1}$H NMR (500 MHz, $CDCl_3$) of compound **8d**

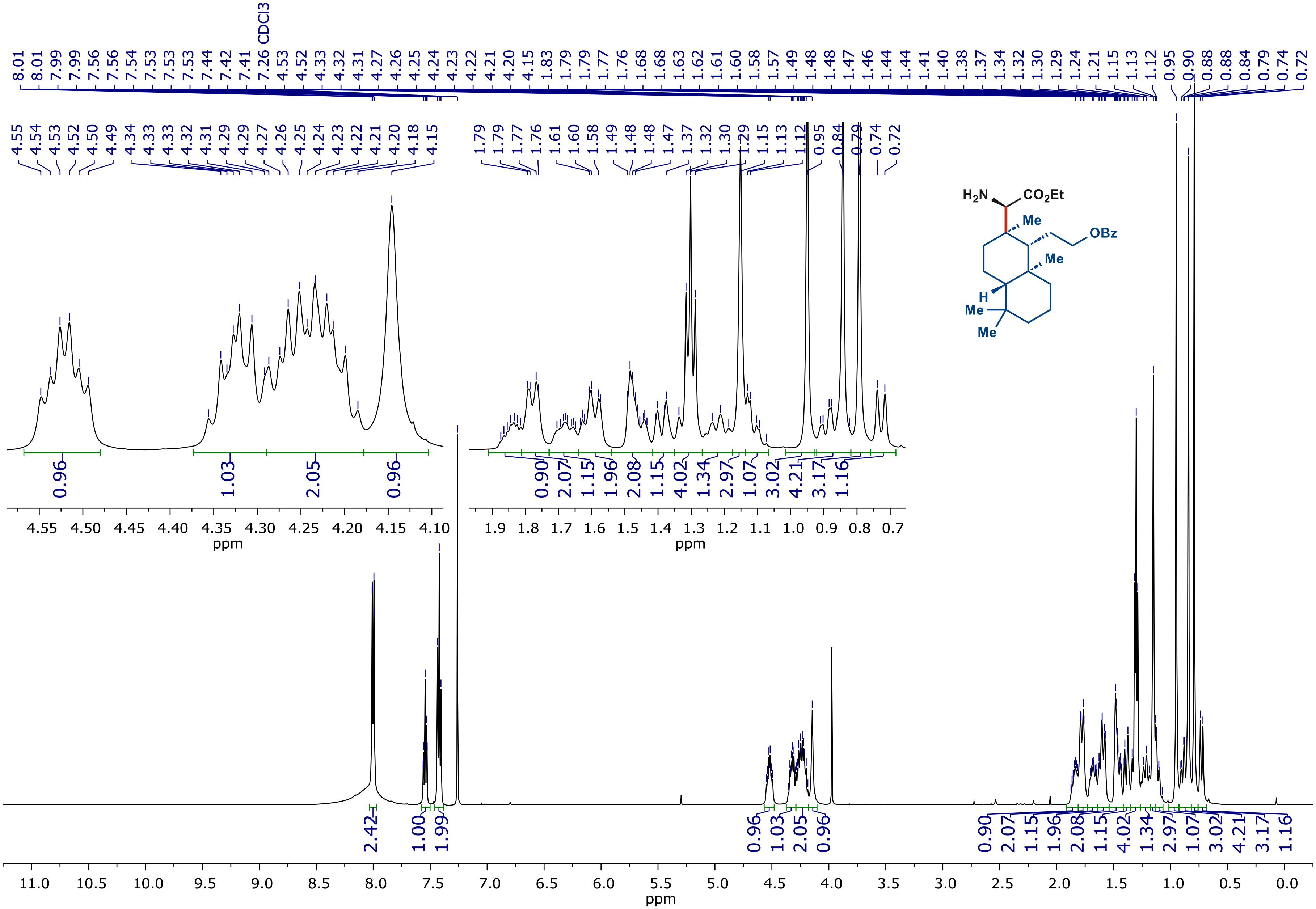

$^{13}$**C NMR** (126 MHz, $CDCl_3$) of compound **8d**

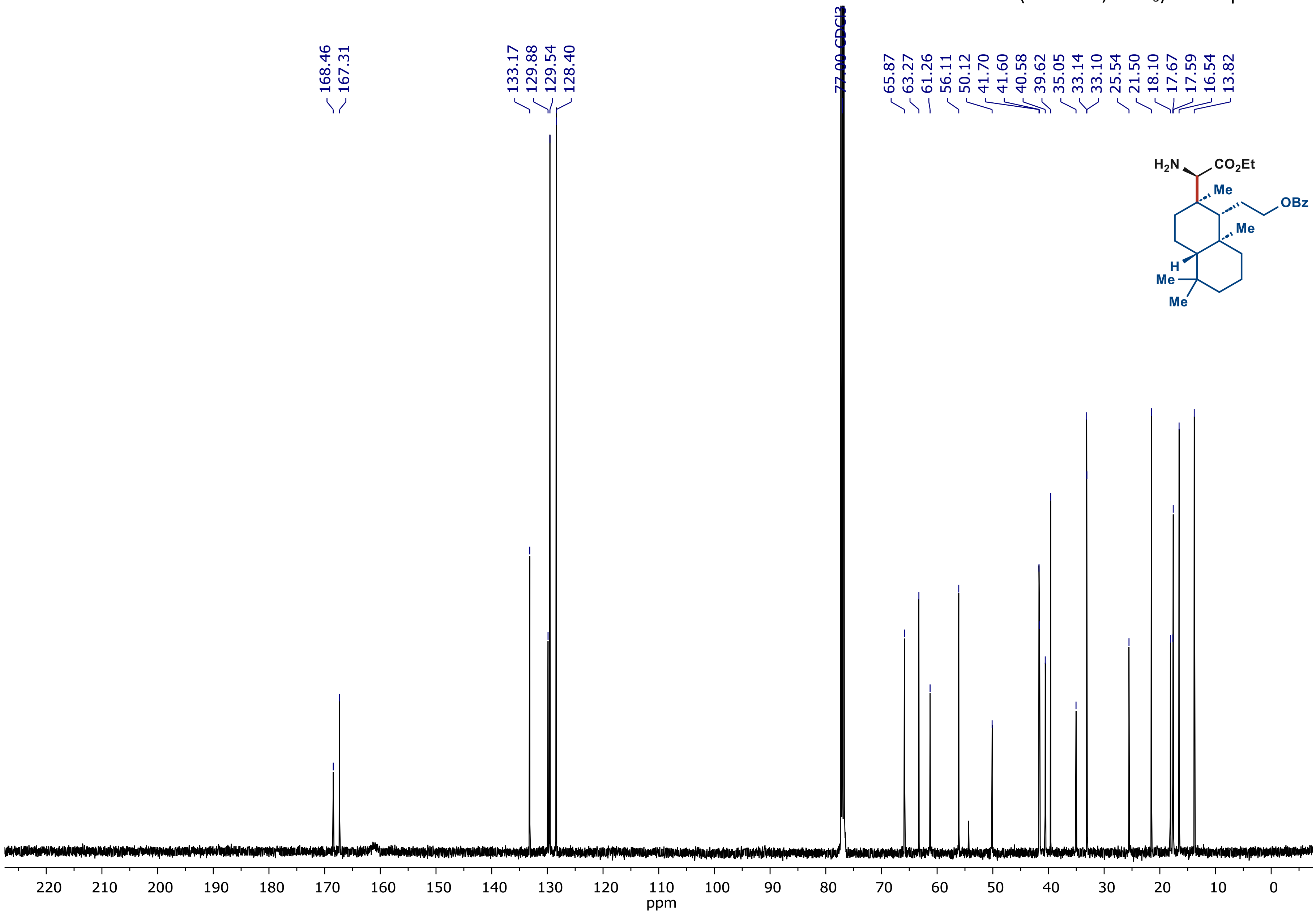

**COSY** of compound **8d**

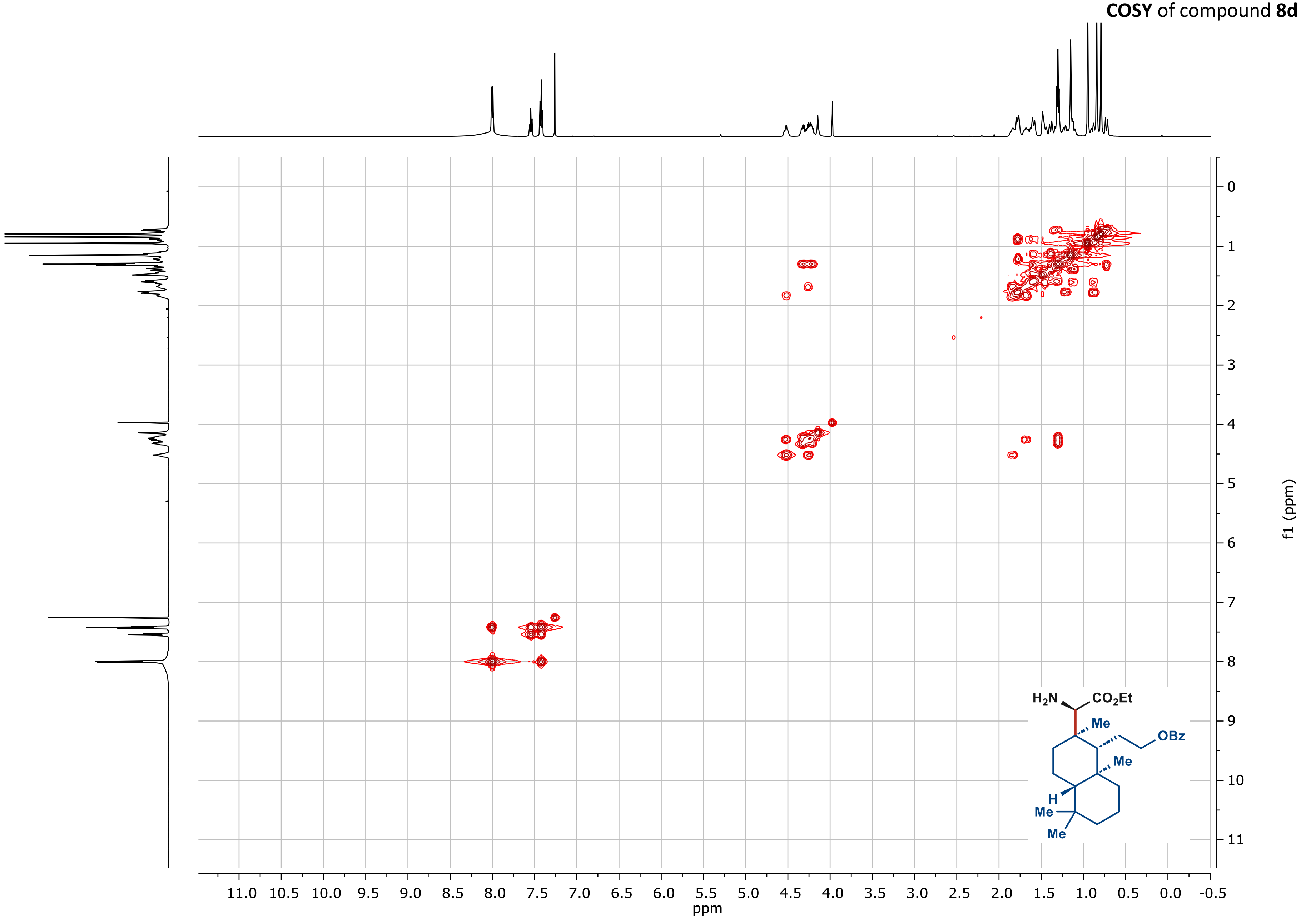

**HSQC** of compound **8d**

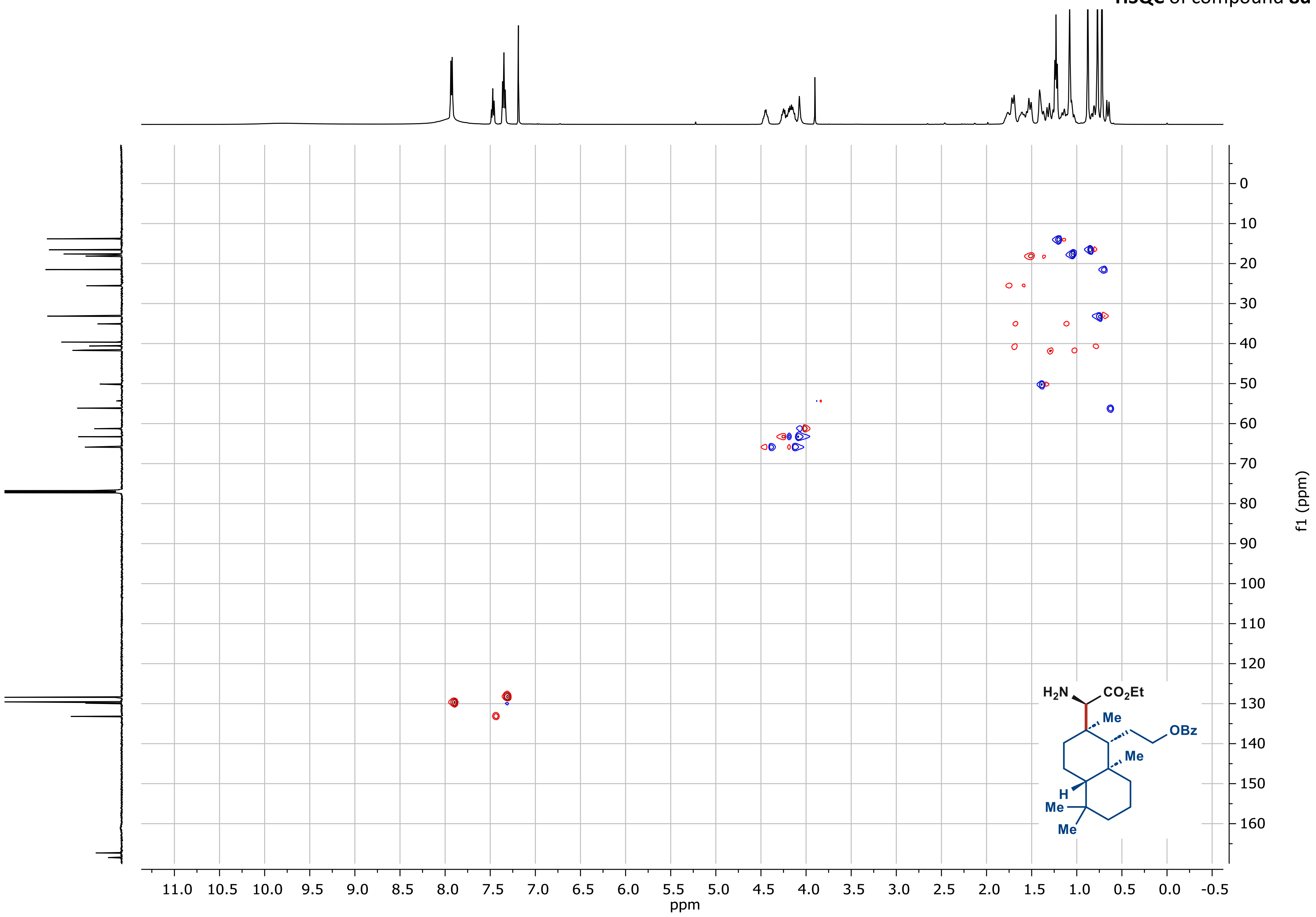

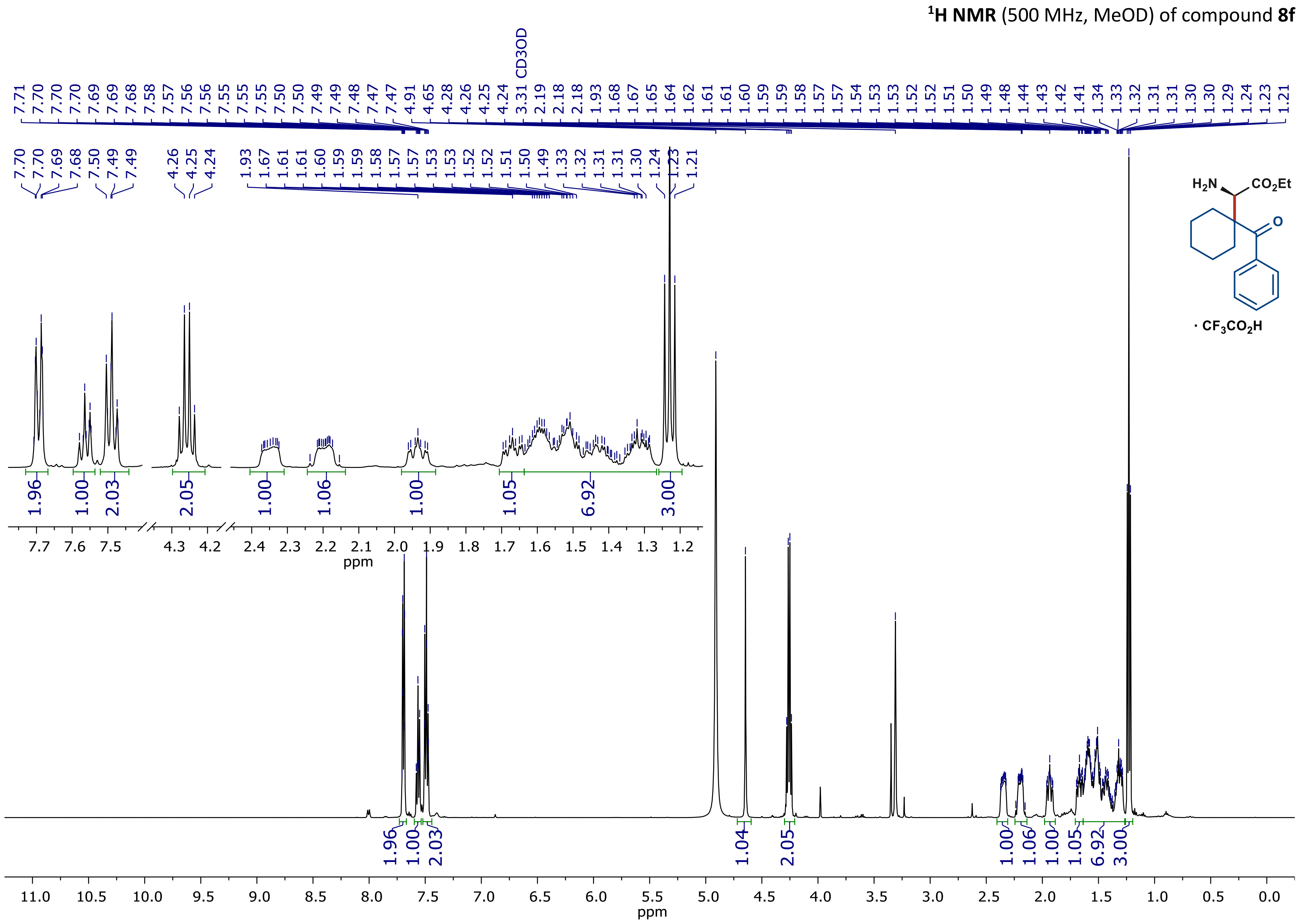

$^1$H NMR (500 MHz, MeOD) of compound 8f
CD3OD
ppm

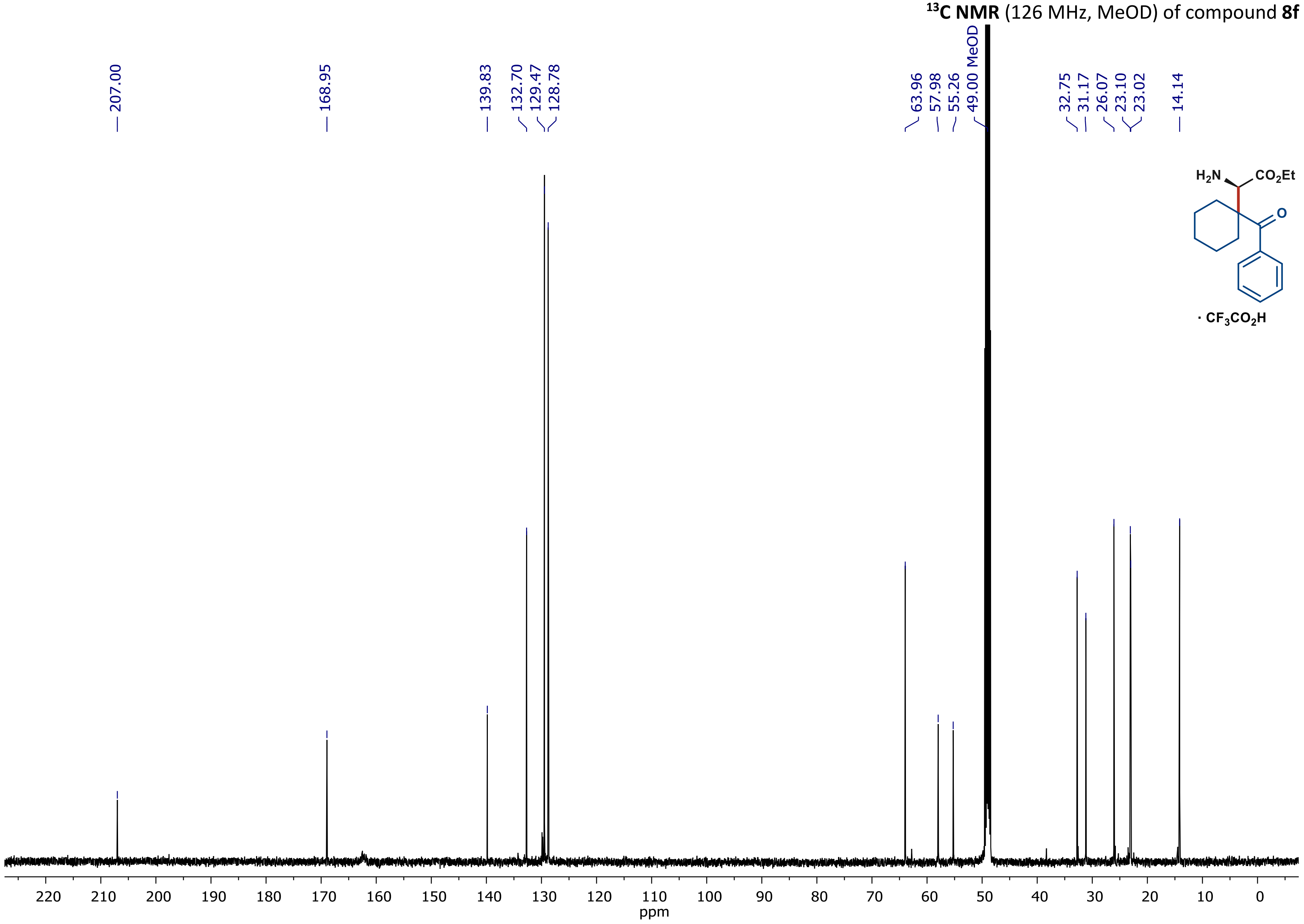
$^{13}$C NMR (126 MHz, MeOD) of compound 8f
207.00
168.95
139.83
132.70
129.47
128.78
63.96
57.98
55.26
49.00 MeOD
32.75
31.17
26.07
23.10
23.02
14.14
H2N
CO2Et
O
· CF3CO2H
220 210 200 190 180 170 160 150 140 130 120 110 100 90 80 70 60 50 40 30 20 10 0
ppm

**$^{19}$F NMR** (377 MHz, MeOD) of compound **8f**

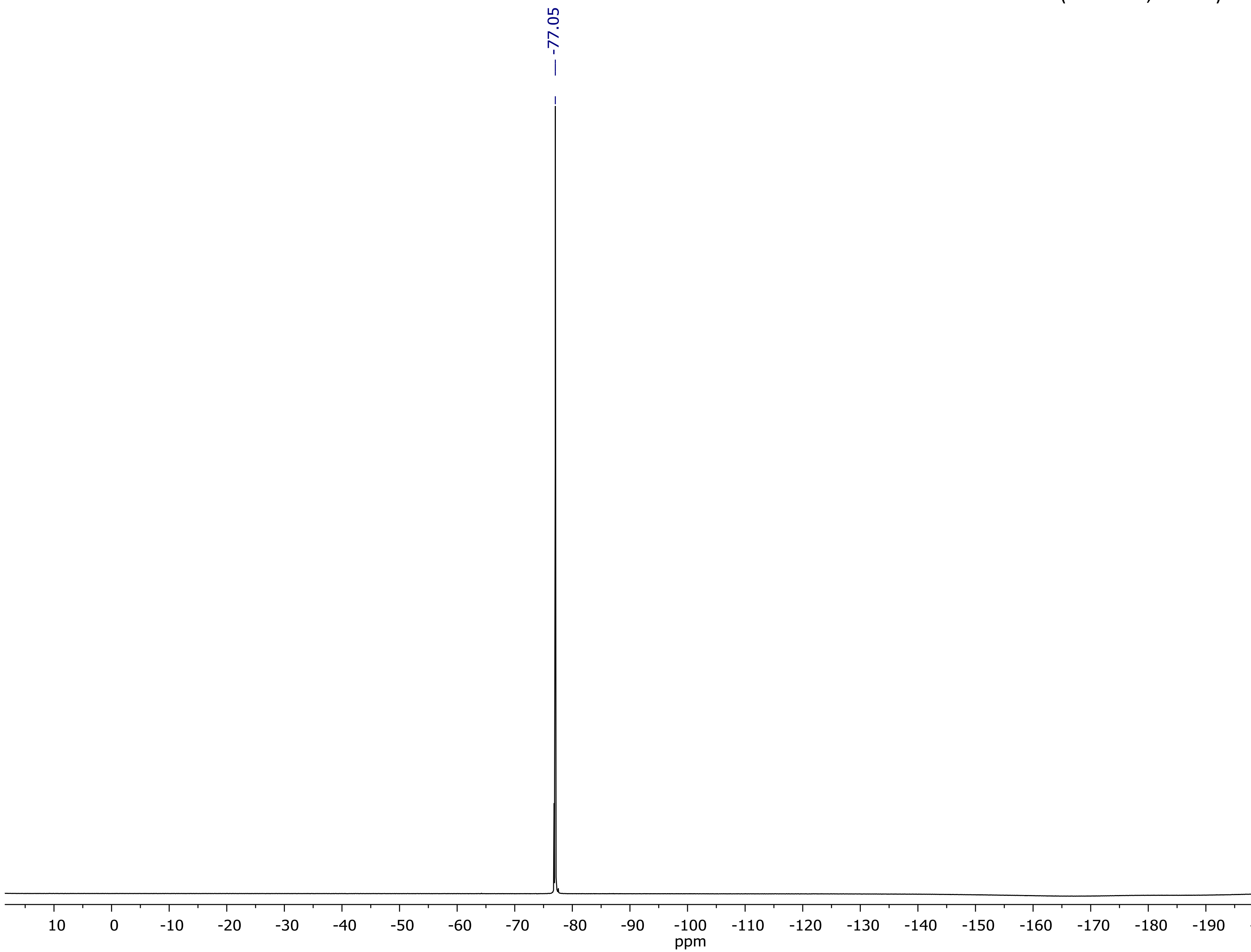


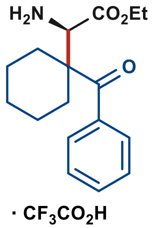

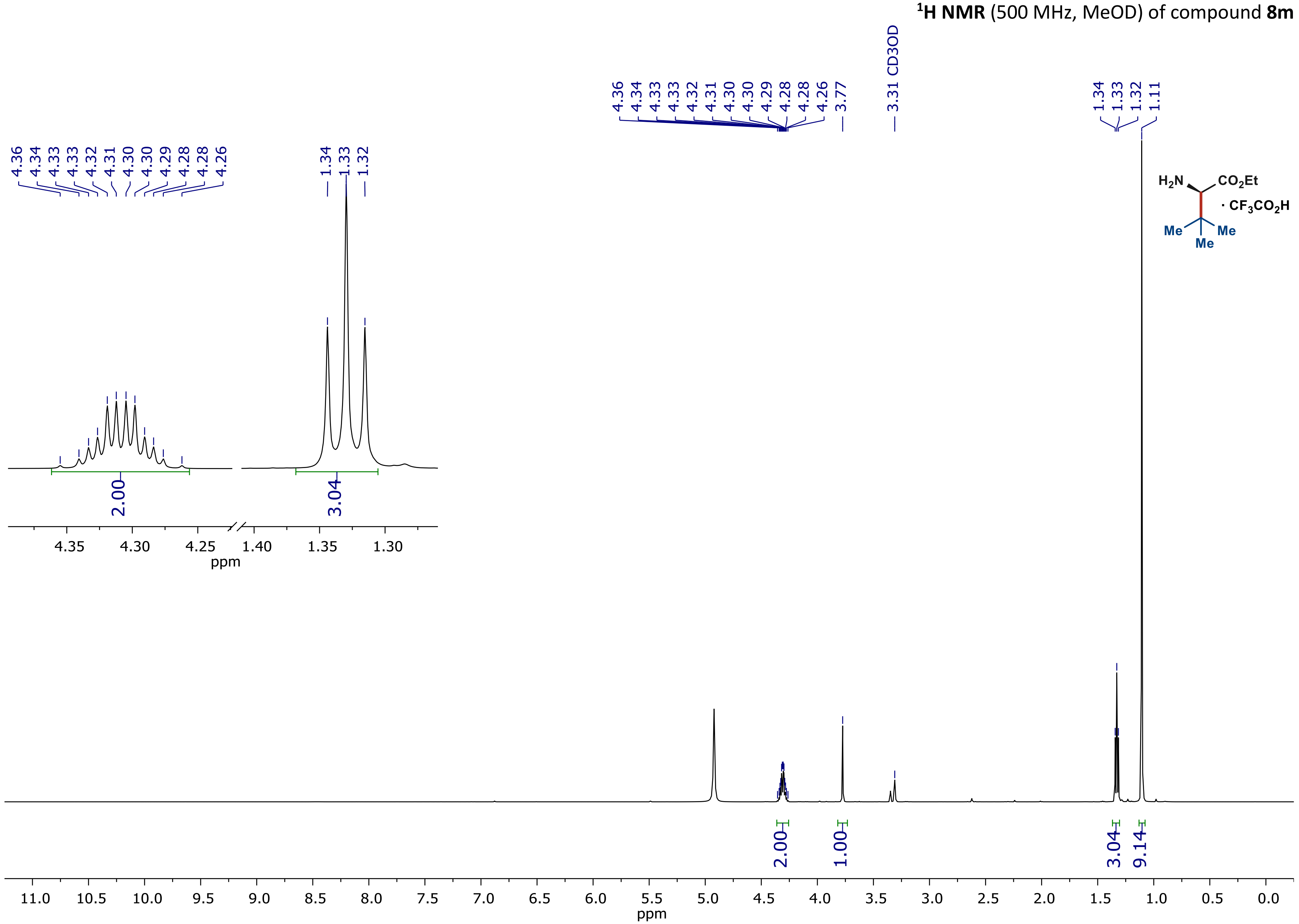

¹H NMR (500 MHz, MeOD) of compound 8m
4.36 4.34 4.33 4.33 4.32 4.31 4.30 4.30 4.29 4.28 4.28 4.26
1.34 1.33 1.32
2.00
3.04
4.35 4.30 4.25 1.40 1.35 1.30
ppm
3.77
3.31 CD3OD
1.11
H2N CO2Et
· CF3CO2H
Me Me Me
1.00
9.14
11.0 10.5 10.0 9.5 9.0 8.5 8.0 7.5 7.0 6.5 6.0 5.5 5.0 4.5 4.0 3.5 3.0 2.5 2.0 1.5 1.0 0.5 0.0

<sup></sup>**$^{13}$C NMR** (126 MHz, $CDCl_3$) of compound **8m**

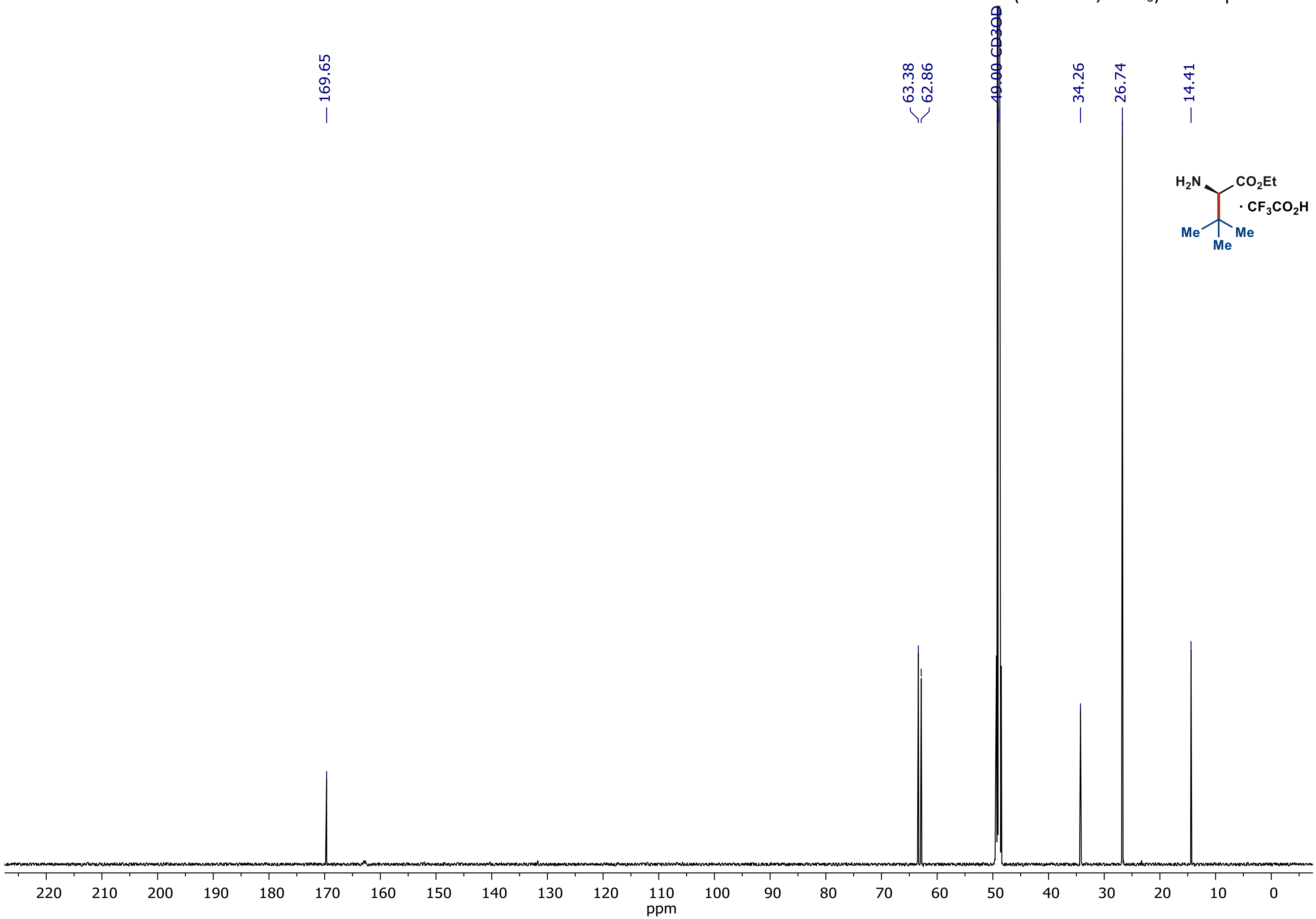

$^{19}$**F NMR** (377 MHz, MeOD) of compound **8m**

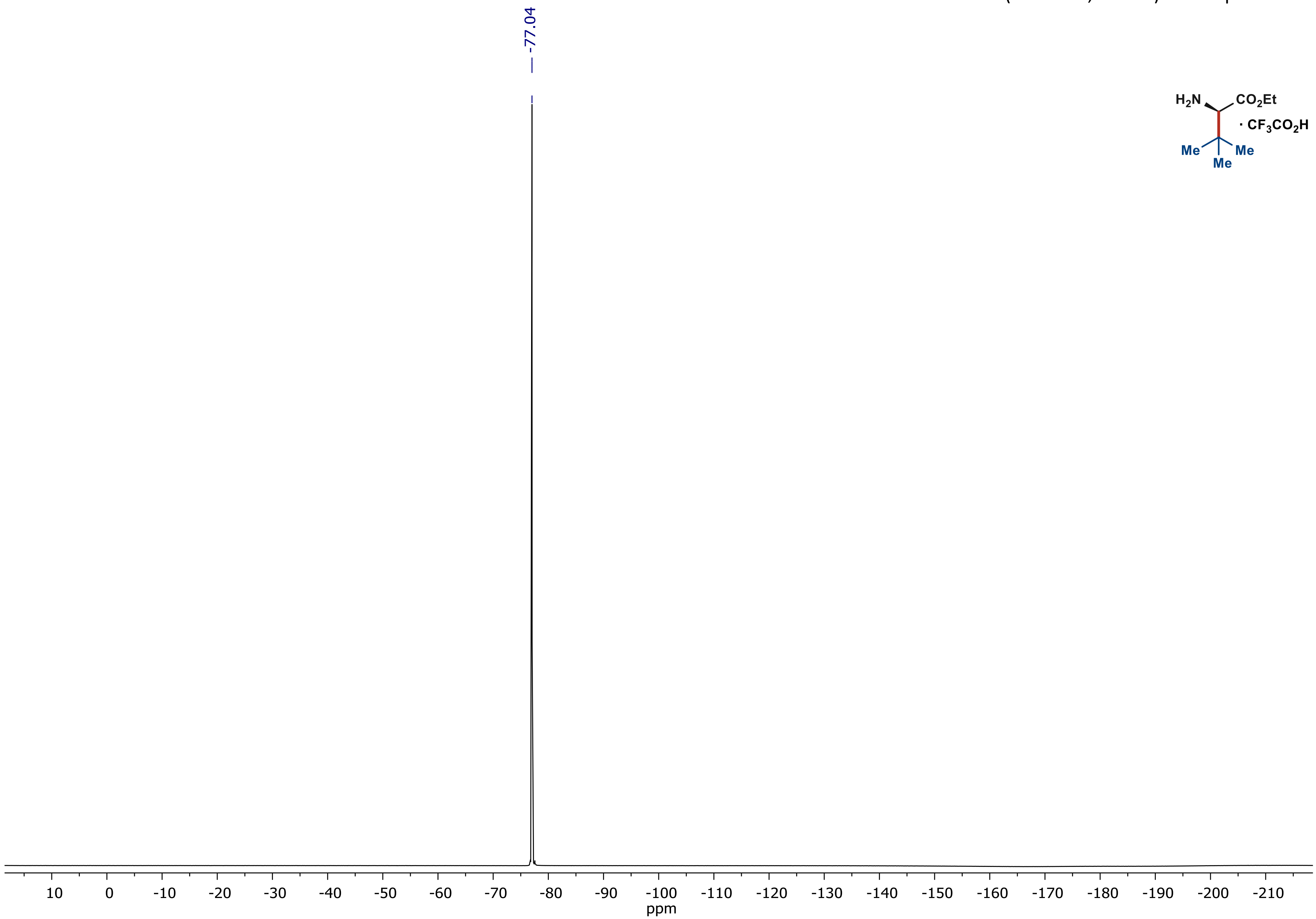

## 7. *Cartesian coordinates and energies*

***PC4***

*Charge:* 1

*Multiplicity:* 1

*E* = −3303.375752

*H* = −3302.863402

*G* = −3303.002736

*Cartesian coordinates:*

| | | | |
|---|---|---|---|
| Ir | -0.002752 | 0.001445 | 0.379864 |
| C | 0.597885 | 0.430411 | -2.570519 |
| C | 2.141203 | 1.590581 | -1.263331 |
| C | 1.189801 | 0.912904 | -3.737561 |
| C | 2.767064 | 2.102684 | -2.391234 |
| H | 2.482263 | 1.833363 | -0.266327 |
| C | 2.287291 | 1.757398 | -3.650698 |
| H | 0.802725 | 0.644607 | -4.709071 |
| C | -2.145032 | -1.594981 | -1.258612 |
| C | -0.580220 | -0.467195 | -2.568771 |
| C | -2.757997 | -2.127590 | -2.383743 |
| H | -2.500473 | -1.815019 | -0.261492 |
| C | -1.157696 | -0.972726 | -3.733561 |
| C | -2.258473 | -1.812023 | -3.643676 |
| H | -0.756816 | -0.725771 | -4.705120 |
| C | 1.094015 | 0.896587 | 1.823186 |
| C | 2.191991 | 0.345639 | 2.487730 |
| C | 0.690475 | 2.229076 | 2.125660 |
| C | 2.869442 | 1.111018 | 3.421902 |
| H | 2.532118 | -0.662458 | 2.293176 |
| C | 1.425543 | 2.942013 | 3.082012 |
| C | 2.515581 | 2.413071 | 3.744806 |
| H | 3.061848 | 2.991292 | 4.476483 |
| C | -1.101709 | -0.887634 | 1.825445 |
| C | -2.199938 | -0.333605 | 2.487001 |
| C | -0.699532 | -2.219375 | 2.132954 |
| C | -2.878404 | -1.094885 | 3.423827 |
| H | -2.539422 | 0.673904 | 2.288301 |
| C | -1.435378 | -2.927952 | 3.091827 |
| C | -2.525372 | -2.395764 | 3.752160 |
| H | -3.072091 | -2.970699 | 4.486088 |
| C | 2.030394 | -2.157434 | -0.255935 |
| C | 0.454159 | -2.725091 | 1.396101 |
| C | 2.647063 | -3.391180 | -0.155010 |

| | | | |
|---|---|---|---|
| H | 2.387736 | -1.409165 | -0.946820 |
| C | 1.051953 | -3.986634 | 1.529764 |
| C | 2.148555 | -4.322429 | 0.756697 |
| H | 0.654497 | -4.696198 | 2.236070 |
| H | 2.607804 | -5.297613 | 0.857377 |
| C | -2.039482 | 2.155166 | -0.263680 |
| C | -0.464195 | 2.730095 | 1.387245 |
| C | -2.663949 | 3.383946 | -0.159049 |
| H | -2.395524 | 1.403551 | -0.951486 |
| C | -1.068754 | 3.988716 | 1.522827 |
| C | -2.168764 | 4.317946 | 0.752863 |
| H | -0.676367 | 4.698484 | 2.231745 |
| H | -2.638084 | 5.287643 | 0.862061 |
| F | -3.944466 | -0.549712 | 4.058867 |
| F | -1.093724 | -4.201615 | 3.412576 |
| F | 1.083578 | 4.216985 | 3.397131 |
| F | 3.935452 | 0.569027 | 4.059682 |
| C | 3.856350 | -3.701883 | -0.988132 |
| C | -3.848089 | 3.717116 | -1.018742 |
| F | -4.896876 | 4.148881 | -0.277887 |
| F | -4.276236 | 2.661807 | -1.742495 |
| F | -3.564768 | 4.712409 | -1.896325 |
| F | 5.003640 | -3.591430 | -0.270704 |
| F | 3.822977 | -4.967009 | -1.465647 |
| F | 3.977042 | -2.873194 | -2.047726 |
| N | 1.085451 | 0.773693 | -1.351368 |
| N | -1.083938 | -0.784842 | -1.349592 |
| N | 0.968052 | -1.831232 | 0.494339 |
| N | -0.974785 | 1.834019 | 0.486589 |
| C | -3.972659 | -3.005506 | -2.236346 |
| C | 3.975457 | 2.988547 | -2.239930 |
| F | 4.035250 | 3.920829 | -3.213826 |
| F | 3.984575 | 3.634715 | -1.055535 |
| F | 5.124601 | 2.273452 | -2.308972 |

| | | | |
|---|---|---|---|
| F | -4.050772 | -3.564365 | -1.011196 |
| F | -5.115734 | -2.302953 | -2.425369 |
| F | -3.974930 | -4.006758 | -3.142219 |

| | | | |
|---|---|---|---|
| H | 2.752465 | 2.144694 | -4.547685 |
| H | -2.712230 | -2.217158 | -4.538723 |

***PC4****

*Charge:* 1

*Multiplicity:* 3

*E* = −3303.293702

*H* = −3302.784

*G* = −3302.925035

*Cartesian coordinates:*

| | | | |
|---|---|---|---|
| Ir | 0.000009 | -0.000037 | 0.461912 |
| C | 0.645940 | 0.294743 | -2.503990 |
| C | 2.429226 | 1.126378 | -1.190903 |
| C | 1.372979 | 0.635171 | -3.677686 |
| C | 3.157915 | 1.476293 | -2.307079 |
| H | 2.823667 | 1.299945 | -0.200243 |
| C | 2.607784 | 1.215356 | -3.591855 |
| H | 0.944161 | 0.436787 | -4.649495 |
| C | -2.429290 | -1.126208 | -1.190949 |
| C | -0.646050 | -0.294422 | -2.504002 |
| C | -3.158032 | -1.475963 | -2.307140 |
| H | -2.823691 | -1.299903 | -0.200296 |
| C | -1.373141 | -0.634689 | -3.677711 |
| C | -2.607950 | -1.214869 | -3.591906 |
| H | -0.944361 | -0.436185 | -4.649513 |
| C | 1.312923 | 0.689296 | 1.806070 |
| C | 2.315588 | -0.060832 | 2.437017 |
| C | 1.190904 | 2.093089 | 2.067900 |
| C | 3.160206 | 0.572433 | 3.327755 |
| H | 2.441699 | -1.117908 | 2.249394 |
| C | 2.077527 | 2.660912 | 2.982772 |
| C | 3.066159 | 1.928559 | 3.621830 |
| H | 3.739155 | 2.403226 | 4.322628 |
| C | -1.312871 | -0.689559 | 1.806010 |
| C | -2.315534 | 0.060480 | 2.437064 |
| C | -1.190828 | -2.093381 | 2.067665 |
| C | -3.160122 | -0.572903 | 3.327748 |
| H | -2.441667 | 1.117576 | 2.249566 |
| C | -2.077418 | -2.661324 | 2.982495 |
| C | -3.066045 | -1.929062 | 3.621664 |
| H | -3.739015 | -2.403819 | 4.322425 |

| | | | |
|---|---|---|---|
| C | 1.582545 | -2.490438 | -0.233933 |
| C | -0.136783 | -2.798883 | 1.342254 |
| C | 1.908537 | -3.835964 | -0.196815 |
| H | 2.111158 | -1.795199 | -0.868949 |
| C | 0.161061 | -4.163980 | 1.410661 |
| C | 1.188054 | -4.685325 | 0.640031 |
| H | -0.409076 | -4.809740 | 2.057521 |
| H | 1.418881 | -5.741951 | 0.686493 |
| C | -1.582532 | 2.490463 | -0.233568 |
| C | 0.136845 | 2.798693 | 1.342607 |
| C | -1.908506 | 3.835987 | -0.196277 |
| H | -2.111175 | 1.795308 | -0.868654 |
| C | -0.160983 | 4.163785 | 1.411191 |
| C | -1.187989 | 4.685237 | 0.640653 |
| H | 0.409180 | 4.809457 | 2.058115 |
| H | -1.418799 | 5.741862 | 0.687251 |
| F | -4.119726 | 0.140877 | 3.946263 |
| F | -2.006149 | -3.973785 | 3.284076 |
| F | 2.006287 | 3.973339 | 3.284508 |
| F | 4.119812 | -0.141435 | 3.946167 |
| C | 3.047415 | -4.360066 | -1.027934 |
| C | -3.047391 | 4.360213 | -1.027308 |
| F | -4.152653 | 4.577869 | -0.273464 |
| F | -3.400121 | 3.503792 | -2.007813 |
| F | -2.734919 | 5.539156 | -1.608480 |
| F | 4.152743 | -4.577651 | -0.274166 |
| F | 2.735008 | -5.539014 | -1.609126 |
| F | 3.400012 | -3.503579 | -2.008430 |
| N | 1.220014 | 0.546014 | -1.262835 |
| N | -1.220072 | -0.545854 | -1.262855 |
| N | 0.594437 | -1.990123 | 0.517241 |

| | | | |
|---|---|---|---|
| N | -0.594409 | 1.990043 | 0.517516 |
| C | -4.500992 | -2.101358 | -2.180164 |
| C | 4.500874 | 2.101685 | -2.180077 |
| F | -4.568068 | -3.311387 | -2.805622 |
| F | -4.871512 | -2.305792 | -0.895604 |
| F | -5.480646 | -1.344487 | -2.753920 |
| F | 4.567923 | 3.311775 | -2.805419 |
| F | 5.480519 | 1.344878 | -2.753933 |
| F | 4.871427 | 2.305996 | -0.895507 |
| H | -3.157137 | -1.472467 | -4.488630 |
| H | 3.156931 | 1.473077 | -4.488569 |

***PC4$^{red}$***

*Charge:* 1

*Multiplicity:* 2

*E* = −3303.514387

*H* = −3303.005126

*G* = −3303.144879

*Cartesian coordinates:*

| | | | |
|---|---|---|---|
| Ir | -0.000002 | 0.000054 | 0.400714 |
| C | -0.589870 | -0.398946 | -2.539740 |
| C | -2.217464 | -1.500355 | -1.228081 |
| C | -1.251199 | -0.853351 | -3.715566 |
| C | -2.886042 | -1.965795 | -2.346707 |
| H | -2.570298 | -1.732352 | -0.231947 |
| C | -2.379621 | -1.622385 | -3.629592 |
| H | -0.859256 | -0.592466 | -4.688617 |
| C | 2.217565 | 1.500242 | -1.228145 |
| C | 0.590116 | 0.398559 | -2.539757 |
| C | 2.886255 | 1.965467 | -2.346792 |
| H | 2.570291 | 1.732439 | -0.232019 |
| C | 1.251569 | 0.852732 | -3.715603 |
| C | 2.379975 | 1.621793 | -3.629663 |
| H | 0.859733 | 0.591647 | -4.688643 |
| C | -1.108854 | -0.892433 | 1.841098 |
| C | -2.196331 | -0.336314 | 2.522874 |
| C | -0.722263 | -2.234668 | 2.130746 |
| C | -2.875531 | -1.100577 | 3.454859 |
| H | -2.524262 | 0.678240 | 2.337787 |
| C | -1.458053 | -2.946336 | 3.086816 |
| C | -2.536094 | -2.410703 | 3.764040 |
| H | -3.083218 | -2.988234 | 4.495617 |
| C | 1.108760 | 0.892724 | 1.841053 |
| C | 2.196213 | 0.336698 | 2.522943 |
| C | 0.722134 | 2.234985 | 2.130529 |
| C | 2.875360 | 1.101080 | 3.454870 |
| H | 2.524166 | -0.677873 | 2.337987 |
| C | 1.457871 | 2.946775 | 3.086549 |
| C | 2.535891 | 2.411237 | 3.763883 |
| H | 3.082974 | 2.988862 | 4.495415 |
| C | -1.992097 | 2.169266 | -0.276030 |
| C | -0.419646 | 2.744857 | 1.377078 |
| C | -2.587608 | 3.416177 | -0.206628 |
| H | -2.348975 | 1.409087 | -0.954733 |
| C | -0.997020 | 4.019538 | 1.479548 |
| C | -2.081460 | 4.358041 | 0.690506 |
| H | -0.592042 | 4.737316 | 2.173292 |
| H | -2.524284 | 5.343087 | 0.766413 |
| C | 1.992107 | -2.169261 | -0.275670 |
| C | 0.419543 | -2.744645 | 1.377404 |
| C | 2.587562 | -3.416187 | -0.206131 |
| H | 2.349057 | -1.409151 | -0.954410 |
| C | 0.996864 | -4.019342 | 1.480016 |
| C | 2.081323 | -4.357957 | 0.691054 |
| H | 0.591824 | -4.737043 | 2.173804 |
| H | 2.524096 | -5.343020 | 0.767068 |
| F | 3.931514 | 0.552292 | 4.108841 |
| F | 1.128681 | 4.230606 | 3.390545 |
| F | -1.128894 | -4.230134 | 3.390980 |
| F | -3.931705 | -0.551697 | 4.108718 |
| C | -3.782002 | 3.730094 | -1.057498 |
| C | 3.781931 | -3.730311 | -1.056957 |
| F | 4.940864 | -3.648480 | -0.351805 |
| F | 3.904857 | -2.887215 | -2.105236 |
| F | 3.727495 | -4.987906 | -1.555157 |

| | | | | | | | |
|---|---|---|---|---|---|---|---|
| F | -4.941020 | 3.647202 | -0.352602 | F | 3.974312 | 4.015803 | -2.831947 |
| F | -3.728198 | 4.987977 | -1.555013 | F | 4.454359 | 3.047401 | -0.938385 |
| F | -3.904258 | 2.887476 | -2.106246 | F | 5.196776 | 2.224802 | -2.815732 |
| N | -1.119280 | -0.741924 | -1.299983 | F | -3.974096 | -4.016193 | -2.831607 |
| N | 1.119397 | 0.741783 | -1.300012 | F | -5.196527 | -2.225167 | -2.815802 |
| N | -0.943261 | 1.840833 | 0.491092 | F | -4.454290 | -3.047459 | -0.938251 |
| N | 0.943237 | -1.840722 | 0.491367 | H | 2.878677 | 1.964906 | -4.527714 |
| C | 4.106417 | 2.799041 | -2.222515 | H | -2.878228 | -1.965676 | -4.527628 |
| C | -4.106231 | -2.799324 | -2.222393 | | | | |

***Sodium 1-methylcyclohexyloxalate ($3_{tert}$·$Na^+$)***

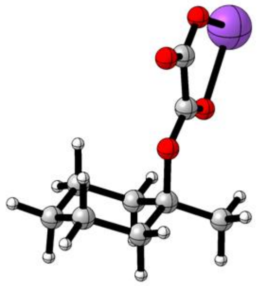

*Charge:* 0

*Multiplicity:* 0

*E* = -814.360512714

H = -814.130602

*G* = -814.188234

*Cartesian coordinates:*

| | | | | | | | |
|---|---|---|---|---|---|---|---|
| C | -0.787966 | -1.952466 | 0.968853 | C | -1.239258 | -4.911849 | 3.496594 |
| H | -0.057543 | -1.787257 | 0.172929 | H | -2.495071 | -3.510872 | 2.433217 |
| H | -1.785603 | -1.957969 | 0.523374 | H | -1.384895 | -2.737632 | 3.557584 |
| O | 0.888378 | -3.356482 | 2.091573 | C | -0.390802 | -5.824830 | 1.294683 |
| O | 0.788572 | -1.664265 | 3.581225 | H | -1.619126 | -4.420969 | 0.194597 |
| C | 1.398500 | -2.548693 | 3.001531 | H | 0.100944 | -4.279418 | -0.159685 |
| C | 2.928495 | -2.834577 | 3.292553 | C | -1.343828 | -6.052804 | 2.475527 |
| O | 3.401454 | -2.108938 | 4.204273 | H | -1.963040 | -5.053535 | 4.304799 |
| O | 3.492216 | -3.710559 | 2.618700 | H | -0.245266 | -4.925538 | 3.958603 |
| Na | 2.094360 | -0.483830 | 5.211855 | H | -0.518447 | -6.609849 | 0.543141 |
| H | -0.729014 | -1.131562 | 1.682481 | H | 0.644332 | -5.885139 | 1.646210 |
| C | -0.535499 | -3.295026 | 1.646667 | H | -1.128913 | -7.012888 | 2.955095 |
| C | -1.475716 | -3.548277 | 2.834781 | H | -2.375290 | -6.107348 | 2.10349 |
| C | -0.620514 | -4.453891 | 0.644038 | | | | |

***1-Methylcyclohexyl oxyacyl radical — $CO_2$ — sodium complex ($10_{tert}$·$CO_2$·Na)***

*Charge:* 1

*Multiplicity:* 2

*E* = -814.161127388

H = -813.931619

*G* = -813.996267

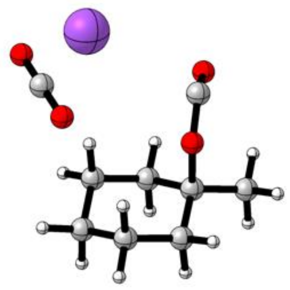

*Cartesian coordinates:*

| | | | |
|---|---|---|---|
| C | -1.553007 | -1.993989 | 0.630533 |
| H | -1.118531 | -1.835774 | -0.358645 |
| H | -2.596308 | -2.294225 | 0.504696 |
| O | 0.689572 | -2.688509 | 1.408892 |
| O | 0.558061 | -0.789529 | 2.681632 |
| C | 1.101730 | -1.635319 | 2.029322 |
| C | 3.141839 | -4.607004 | 4.220726 |
| O | 3.111792 | -4.173666 | 5.297980 |
| O | 3.166857 | -5.038765 | 3.144515 |
| Na | 1.055237 | -1.753577 | 5.720571 |
| H | -1.532859 | -1.055729 | 1.186330 |
| C | -0.811822 | -3.093197 | 1.370559 |
| C | -1.300771 | -3.299477 | 2.803751 |
| C | -0.769007 | -4.406072 | 0.591364 |
| C | -0.549939 | -4.416430 | 3.537594 |
| H | -2.362159 | -3.562966 | 2.724962 |
| H | -1.253279 | -2.358042 | 3.353985 |
| C | -0.028899 | -5.528020 | 1.332440 |
| H | -1.810689 | -4.700626 | 0.419944 |
| H | -0.322735 | -4.222966 | -0.389919 |
| C | -0.585475 | -5.730092 | 2.747772 |
| H | -0.975597 | -4.547568 | 4.536079 |
| H | 0.492624 | -4.114029 | 3.674637 |
| H | -0.106401 | -6.453198 | 0.754284 |
| H | 1.034865 | -5.278962 | 1.395386 |
| H | -0.012522 | -6.500831 | 3.271911 |
| H | -1.621119 | -6.087902 | 2.687394 |

***1-Methylcyclohexyl oxyacyl radical (axial) ($10_{tert}$-ax)***

*Charge:* 0

*Multiplicity:* 2

*E* = -463.250066377

*H* = -463.040389

*G* = -463.086175

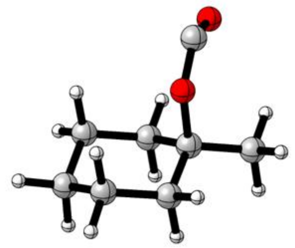

*Cartesian coordinates:*

| | | | |
|---|---|---|---|
| C | -0.944456 | -2.001374 | 0.876174 |
| H | -0.233751 | -1.845407 | 0.061891 |
| H | -1.953687 | -2.004412 | 0.456747 |
| O | 0.791552 | -3.345142 | 2.017291 |
| O | 0.752458 | -1.622913 | 3.526854 |
| C | 1.245497 | -2.511251 | 2.893770 |
| H | -0.870646 | -1.172402 | 1.581038 |
| C | -0.691872 | -3.334504 | 1.558446 |
| C | -1.583556 | -3.573312 | 2.777002 |
| C | -0.734693 | -4.511909 | 0.586235 |
| C | -1.329562 | -4.925622 | 3.455187 |
| H | -2.615001 | -3.538368 | 2.406116 |
| H | -1.477479 | -2.748296 | 3.483899 |
| C | -0.487378 | -5.866148 | 1.264552 |

| | | | | | | | |
|---|---|---|---|---|---|---|---|
| H | -1.731250 | -4.504421 | 0.129865 | H | -0.607804 | -6.665450 | 0.527606 |
| H | -0.013424 | -4.337065 | -0.216879 | H | 0.549225 | -5.909324 | 1.614369 |
| C | -1.435120 | -6.081378 | 2.451661 | H | -1.212460 | -7.032331 | 2.944923 |
| H | -2.044851 | -5.058705 | 4.271786 | H | -2.467643 | -6.147230 | 2.085155 |
| H | -0.331564 | -4.924974 | 3.907783 | | | | |

***1-Methylcyclohexyl oxyacyl radical (equatorial) ($10_{tert}$-eq)***

*Charge:* 0

*Multiplicity:* 2

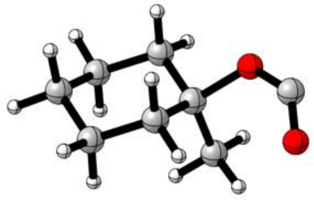

*E* = -463.248612971

*H* = -463.038714

*G* = -463.084422

*Cartesian coordinates:*

| | | | | | | | |
|---|---|---|---|---|---|---|---|
| C | 0.754276 | -2.992575 | 2.007687 | H | -1.596612 | -2.677619 | 3.374267 |
| H | 1.178482 | -3.831345 | 2.562337 | C | -0.476716 | -5.862665 | 1.312306 |
| H | 1.398728 | -2.793209 | 1.149263 | H | -1.678744 | -4.537252 | 0.101681 |
| O | -1.136293 | -2.180648 | 0.640757 | H | 0.051463 | -4.385279 | -0.206064 |
| O | -1.121530 | -0.473112 | 2.168225 | C | -1.486707 | -6.040642 | 2.453214 |
| C | -1.284576 | -0.977946 | 1.095507 | H | -2.216728 | -4.953150 | 4.188100 |
| H | 0.750360 | -2.122969 | 2.665199 | H | -0.484556 | -4.831103 | 3.942428 |
| C | -0.651908 | -3.341488 | 1.543857 | H | -0.564817 | -6.679835 | 0.591002 |
| C | -1.662875 | -3.515908 | 2.678627 | H | 0.542583 | -5.909358 | 1.711216 |
| C | -0.694344 | -4.527314 | 0.580578 | H | -1.287010 | -6.970951 | 2.993278 |
| C | -1.446709 | -4.849576 | 3.418624 | H | -2.495517 | -6.127691 | 2.030636 |
| H | -2.667321 | -3.507263 | 2.242472 | | | | |

***$CO_2$***

*Charge:* 0

*Multiplicity:* 1

*E* = −188.6499252

*H* = −188.634783

*G* = −188.659051

*Cartesian coordinates:*

| | | | | | | | |
|---|---|---|---|---|---|---|---|
| C | -2.707242 | -0.060464 | 0.062454 | O | -2.401194 | -0.567040 | 1.060950 |
| O | -3.013289 | 0.446113 | -0.936041 | | | | |

***TS for $CO_2$ elimination from 1-methylcyclohexyl oxyacyl radical (axial) ($TS1_{tert}$-ax)***

*Charge:* 0

Multiplicity: 2

*E* = -463.241405180

*H* = -463.034740

*G* = -463.082648

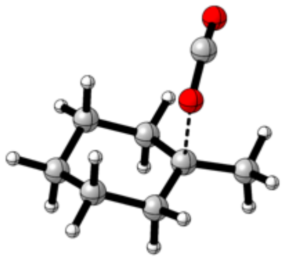

*Cartesian coordinates:*

| | | | |
|---|---|---|---|
| C | -1.919926 | -0.954166 | -0.002623 |
| C | -0.888150 | 0.135694 | -0.026082 |
| C | -0.749794 | 0.909499 | -1.299163 |
| H | 0.127969 | 1.557742 | -1.286794 |
| H | -1.834452 | -1.563557 | -0.906322 |
| H | -0.701693 | 0.248175 | -2.165962 |
| O | 0.714713 | -1.028422 | -0.142871 |
| O | 2.490356 | 0.383059 | 0.292802 |
| C | 1.846735 | -0.590500 | 0.029755 |
| H | -1.635325 | 1.549925 | -1.416801 |
| H | -2.896054 | -0.445830 | -0.065134 |
| C | -0.690417 | 0.890382 | 1.258494 |
| H | -1.552729 | 1.574910 | 1.330065 |
| H | 0.196352 | 1.524198 | 1.193475 |
| C | -1.881251 | -1.824041 | 1.262608 |
| H | -2.749889 | -2.488382 | 1.269915 |
| H | -0.990519 | -2.458783 | 1.227866 |
| C | -0.658286 | 0.001525 | 2.509433 |
| H | 0.277540 | -0.567482 | 2.522362 |
| H | -0.657760 | 0.634738 | 3.400919 |
| C | -1.845672 | -0.968635 | 2.534929 |
| H | -1.787863 | -1.611271 | 3.418459 |
| H | -2.779221 | -0.397418 | 2.618047 |

***TS for $CO_2$ elimination from 1-methylcyclohexyl oxyacyl radical (equatorial) ($TS1_{tert}$-eq)***

*Charge:* 0

Multiplicity: 2

*E* = -463.238581975

*H* = -463.031537

*G* = -463.079351

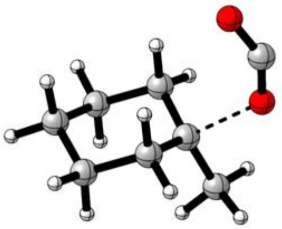

*Cartesian coordinates:*

| | | | |
|---|---|---|---|
| C | -1.966074 | -0.791303 | -0.005817 |
| C | -0.665494 | -0.042570 | 0.082983 |
| C | 0.565755 | -0.735812 | -0.407050 |
| H | 0.833154 | -1.541004 | 0.289803 |
| H | -2.015028 | -1.384734 | -0.921413 |
| H | 0.402800 | -1.187466 | -1.387172 |
| O | -0.782904 | 1.273960 | -1.401073 |
| O | -2.653994 | 2.543498 | -0.920604 |
| C | -1.675353 | 2.111154 | -1.455511 |
| H | 1.412708 | -0.049483 | -0.459832 |
| H | -2.800899 | -0.084891 | -0.012779 |
| C | -0.537105 | 0.817887 | 1.309043 |
| H | -1.342263 | 1.557711 | 1.328691 |
| H | 0.415610 | 1.351898 | 1.315186 |
| C | -2.107521 | -1.699912 | 1.245438 |
| H | -3.073680 | -2.210419 | 1.203612 |
| H | -1.335736 | -2.477105 | 1.220922 |
| C | -0.670622 | -0.080713 | 2.568409 |

| | | | |
|---|---|---|---|
| H | 0.182248 | -0.766494 | 2.619117 |
| H | -0.623323 | 0.551274 | 3.459580 |
| C | -1.979703 | -0.880597 | 2.536551 |
| H | -2.035560 | -1.541827 | 3.406540 |
| H | -2.827094 | -0.187682 | 2.608856 |

***1-Methylcyclohexyl radical — $CO_2$ complex ($11_{tert}$·$CO_2$)***

*Charge:* 0

*Multiplicity:* 2

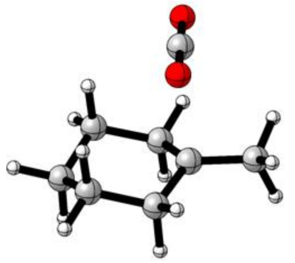

*E* = -463.279840345

*H* = -463.072085

*G* = -463.125917

*Cartesian coordinates:*

| | | | |
|---|---|---|---|
| C | 0.952948 | 1.975127 | 0.822783 |
| H | 1.301018 | 2.497780 | -0.074041 |
| H | 0.511591 | 2.742227 | 1.483739 |
| O | 1.720397 | 0.061099 | -2.142438 |
| O | 2.409034 | -1.131404 | -0.272336 |
| C | 2.049288 | -0.525970 | -1.195849 |
| H | 1.822968 | 1.570742 | 1.350104 |
| C | -0.041325 | 0.909568 | 0.493490 |
| C | -0.342086 | -0.138460 | 1.522644 |
| C | -1.098030 | 1.201496 | -0.529325 |
| C | -1.029450 | -1.384935 | 0.936978 |
| H | -1.011794 | 0.298965 | 2.288489 |
| H | 0.572999 | -0.420612 | 2.054593 |
| C | -1.773918 | -0.065709 | -1.083017 |
| H | -1.877675 | 1.834321 | -0.062346 |
| H | -0.679770 | 1.799936 | -1.345995 |
| C | -2.218442 | -1.001499 | 0.047633 |
| H | -1.352508 | -2.045472 | 1.747832 |
| H | -0.302123 | -1.948021 | 0.339754 |
| H | -2.626967 | 0.212947 | -1.709780 |
| H | -1.064901 | -0.596561 | -1.729614 |
| H | -2.686838 | -1.900169 | -0.366642 |
| H | -2.980999 | -0.498466 | 0.657255 |

***1-Methylcyclohexyl radical (equatorial) ($11_{tert}$-eq)***

*Charge:* 0

*Multiplicity:* 2

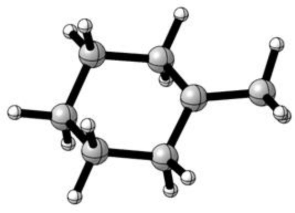

*E* = -274.624469948

*H* = -274.433392

*G* = -274.473826

*Cartesian coordinates:*

| | | | |
|---|---|---|---|
| C | -1.004721 | -1.996141 | 0.836455 |
| H | -0.225041 | -1.910360 | 0.072528 |
| H | -1.956919 | -1.722460 | 0.346490 |
| H | -0.821199 | -1.239592 | 1.606330 |
| C | -1.068211 | -3.372404 | 1.413486 |
| C | -1.805063 | -3.583558 | 2.702575 |
| C | -0.951611 | -4.549519 | 0.491490 |
| C | -1.429668 | -4.901740 | 3.404192 |

| | | | | | | | |
|---|---|---|---|---|---|---|---|
| H | -2.892801 | -3.602625 | 2.492762 | H | -2.090029 | -5.067564 | 4.261481 |
| H | -1.648999 | -2.733175 | 3.375838 | H | -0.409195 | -4.820203 | 3.798223 |
| C | -0.587387 | -5.855282 | 1.221635 | H | -0.648863 | -6.699140 | 0.526909 |
| H | -1.923112 | -4.699970 | -0.019483 | H | 0.453715 | -5.797197 | 1.562211 |
| H | -0.228252 | -4.341247 | -0.304860 | H | -1.216173 | -7.013628 | 2.947149 |
| C | -1.497422 | -6.088077 | 2.434248 | H | -2.532771 | -6.217678 | 2.091316 |

***1-Methylcyclohexyl radical (axial) ($11_{tert}$-ax)***

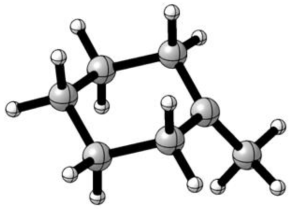

*Charge:* 0

*Multiplicity:* 2

*E* = -274.622668692

*H* = -274.431303

*G* = -274.471864

*Cartesian coordinates:*

| | | | | | | | |
|---|---|---|---|---|---|---|---|
| C | -0.631635 | -3.555096 | 1.662666 | H | -2.308661 | -5.161393 | 4.221196 |
| C | -1.614200 | -3.661965 | 2.792439 | H | -0.570604 | -5.189924 | 3.931336 |
| C | -0.775413 | -4.613969 | 0.608466 | H | -0.871556 | -6.792493 | 0.479296 |
| C | -1.544730 | -5.070192 | 3.441462 | H | 0.309556 | -6.188906 | 1.639616 |
| H | -2.637507 | -3.519814 | 2.413637 | H | -1.633780 | -7.156962 | 2.847843 |
| H | -1.439343 | -2.888803 | 3.547109 | H | -2.735289 | -6.105252 | 1.966359 |
| C | -0.701071 | -6.027736 | 1.244784 | C | 0.695080 | -2.903449 | 1.888209 |
| H | -1.754974 | -4.521466 | 0.115756 | H | 1.190567 | -2.656372 | 0.943617 |
| H | -0.012172 | -4.508620 | -0.168865 | H | 0.600529 | -1.986618 | 2.479222 |
| C | -1.721329 | -6.169013 | 2.383573 | H | 1.386880 | -3.563465 | 2.44191 |

***s-cis conformer of N-sulfinyl imine (4)***

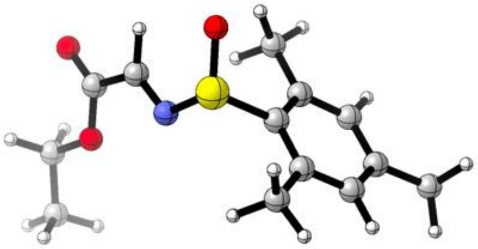

*Charge:* 0

*Multiplicity:* 1

*E* = −1184.479051

*H* = −1184.17913

*G* = −1184.251809

*Cartesian coordinates:*

| | | | | | | | |
|---|---|---|---|---|---|---|---|
| C | -2.352725 | 0.385213 | 1.305839 | C | -2.744721 | 0.479366 | -1.464839 |
| C | -3.344444 | 1.193599 | 0.745057 | C | -1.730833 | -0.341414 | -0.966591 |
| C | -3.560310 | 1.249518 | -0.632491 | C | -1.555487 | -0.375344 | 0.430393 |

| | | | |
|---|---|---|---|
| H | -3.964870 | 1.790755 | 1.405123 |
| H | -2.895652 | 0.520038 | -2.538959 |
| C | -2.172726 | 0.373855 | 2.808818 |
| H | -2.311545 | -0.626874 | 3.226350 |
| H | -1.175071 | 0.712371 | 3.099522 |
| H | -2.900905 | 1.035466 | 3.278879 |
| H | -1.315179 | -1.092673 | -2.933285 |
| H | 0.123848 | -0.655975 | -2.013861 |
| H | -0.730786 | -2.145080 | -1.630006 |
| C | -0.868557 | -1.109790 | -1.938631 |
| C | -4.665658 | 2.097258 | -1.209288 |
| H | -4.393477 | 2.490965 | -2.191020 |
| H | -5.575774 | 1.501356 | -1.336107 |
| H | -4.909357 | 2.935516 | -0.553564 |
| S | -0.329747 | -1.461086 | 1.234508 |
| O | -0.358936 | -2.831844 | 0.593994 |
| N | 1.071504 | -0.547472 | 0.688340 |
| C | 1.942558 | -1.204640 | 0.042040 |
| H | 1.835658 | -2.258639 | -0.226083 |
| C | 3.216753 | -0.567508 | -0.441089 |
| O | 4.023603 | -1.203454 | -1.086997 |
| H | 5.410642 | 0.866580 | -0.123054 |
| H | 4.597387 | 1.343057 | -1.624300 |
| O | 3.338604 | 0.707437 | -0.097757 |
| C | 4.550257 | 1.397552 | -0.535002 |
| C | 4.459690 | 2.821491 | -0.034026 |
| H | 5.352322 | 3.370017 | -0.344054 |
| H | 3.584055 | 3.325416 | -0.448660 |
| H | 4.399864 | 2.848193 | 1.056058 |

***1-Methylcyclohexyl radical (equatorial) — N-sulfinyl imine 4 re-precomplex ($11_{tert}$·4-eq)***

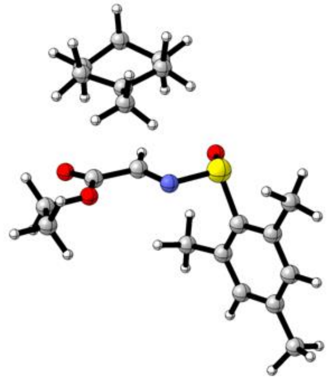

*Charge:* 0

*Multiplicity:* 2

*E* = -1459.11875175

*H* = -1458.625110

*G* = -1458.719000

*Cartesian coordinates:*

| | | | |
|---|---|---|---|
| C | -3.460447 | -0.707483 | 1.157327 |
| C | -4.767234 | -0.243537 | 0.984261 |
| C | -5.204279 | 0.297056 | -0.225738 |
| C | -4.294816 | 0.381833 | -1.282830 |
| C | -2.974263 | -0.057388 | -1.168593 |
| C | -2.578136 | -0.604761 | 0.066680 |
| H | -5.459358 | -0.311197 | 1.817283 |
| H | -4.618765 | 0.807603 | -2.227390 |
| C | -3.052723 | -1.282591 | 2.497202 |
| H | -2.717802 | -2.319409 | 2.408000 |
| H | -2.235443 | -0.714948 | 2.949194 |
| H | -3.896890 | -1.262300 | 3.187239 |
| H | -2.610859 | 0.330576 | -3.248806 |
| H | -1.353187 | 0.939719 | -2.171965 |
| H | -1.440874 | -0.783525 | -2.515096 |
| C | -2.042461 | 0.108396 | -2.344856 |
| C | -6.631471 | 0.751404 | -0.400734 |
| H | -6.697568 | 1.598959 | -1.086493 |
| H | -7.240361 | -0.056938 | -0.819525 |
| H | -7.078305 | 1.040230 | 0.552721 |
| S | -0.909043 | -1.289870 | 0.336705 |
| O | -0.535709 | -2.184975 | -0.830067 |
| N | -0.072454 | 0.236784 | 0.247792 |
| C | 0.908547 | 0.288693 | -0.572114 |
| H | 1.158690 | -0.512550 | -1.266764 |
| C | 1.687763 | 1.547246 | -0.779532 |
| O | 2.442151 | 1.677740 | -1.724147 |
| H | 3.238943 | 3.527103 | -0.014689 |
| H | 1.896782 | 4.187890 | -0.962669 |
| O | 1.448366 | 2.482599 | 0.136776 |
| C | 2.168488 | 3.742339 | -0.003555 |
| C | 1.773226 | 4.615068 | 1.167186 |
| H | 2.293563 | 5.573280 | 1.097223 |
| H | 0.697809 | 4.805444 | 1.165269 |
| H | 2.045565 | 4.142713 | 2.113493 |
| C | 3.128317 | -0.586045 | 0.969770 |
| C | 2.779218 | 0.283902 | 2.130356 |
| H | 3.042169 | 1.329395 | 1.959165 |

| | | | |
|---|---|---|---|
| H | 1.711208 | 0.228148 | 2.364087 |
| H | 3.321286 | -0.052055 | 3.029494 |
| C | 4.344171 | -0.258253 | 0.159258 |
| H | 4.391281 | 0.816969 | -0.040636 |
| H | 5.229076 | -0.482472 | 0.784920 |
| C | 2.703079 | -2.022226 | 1.043747 |
| H | 1.677210 | -2.096322 | 1.421080 |
| H | 3.328120 | -2.500565 | 1.821515 |
| C | 2.866936 | -2.811016 | -0.264816 |
| H | 2.726975 | -3.877540 | -0.063469 |
| H | 2.074340 | -2.522772 | -0.961996 |
| C | 4.452429 | -1.059086 | -1.148171 |
| H | 3.699643 | -0.690366 | -1.852846 |
| H | 5.430024 | -0.879611 | -1.605864 |
| C | 4.236136 | -2.557201 | -0.905330 |
| H | 5.026622 | -2.937383 | -0.244655 |
| H | 4.318632 | -3.108663 | -1.847232 |

***1-Methylcyclohexyl radical (axial) — N-sulfinyl imine 4 re-precomplex ($11_{tert}$·4-ax)***

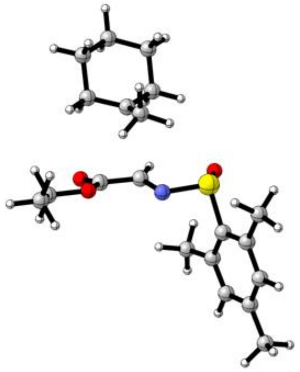

*Charge:* 0

*Multiplicity:* 2

*E* = -1459.11686737

*H* = -1458.622707

*G* = -1458.716876

*Cartesian coordinates:*

| | | | |
|---|---|---|---|
| C | -3.588743 | -0.855803 | 1.162337 |
| C | -4.910108 | -0.402634 | 1.118895 |
| C | -5.432036 | 0.243954 | -0.002003 |
| C | -4.594140 | 0.449534 | -1.101056 |
| C | -3.263092 | 0.028074 | -1.113977 |
| C | -2.780558 | -0.630063 | 0.033508 |
| H | -5.545493 | -0.563986 | 1.983634 |
| H | -4.984554 | 0.958999 | -1.976523 |
| C | -3.087176 | -1.549397 | 2.411226 |
| H | -2.745198 | -2.566100 | 2.200542 |
| H | -2.249809 | -1.012065 | 2.863647 |
| H | -3.884953 | -1.612033 | 3.152055 |
| H | -3.037487 | 0.638288 | -3.160239 |
| H | -1.710770 | 1.136978 | -2.109057 |
| H | -1.822209 | -0.540005 | -2.627613 |
| C | -2.410928 | 0.324996 | -2.324331 |
| C | -6.873404 | 0.685459 | -0.041060 |
| H | -6.992665 | 1.601872 | -0.623405 |
| H | -7.495442 | -0.083949 | -0.510769 |
| H | -7.265404 | 0.858423 | 0.963193 |
| S | -1.089905 | -1.307458 | 0.125925 |
| O | -0.791182 | -2.086359 | -1.142256 |
| N | -0.267644 | 0.228104 | 0.132296 |
| C | 0.659194 | 0.363136 | -0.741439 |
| H | 0.839181 | -0.353081 | -1.543400 |
| C | 1.456103 | 1.620048 | -0.844404 |
| O | 2.054544 | 1.911858 | -1.862698 |
| H | 3.210694 | 3.381866 | -0.062299 |
| H | 1.741713 | 4.265149 | -0.508364 |
| O | 1.428655 | 2.362070 | 0.261944 |
| C | 2.186444 | 3.606681 | 0.240868 |
| C | 2.119004 | 4.193410 | 1.633588 |
| H | 2.674735 | 5.133819 | 1.658899 |
| H | 1.085319 | 4.395587 | 1.922392 |
| H | 2.561340 | 3.510916 | 2.362637 |
| C | 2.954618 | -0.667813 | 0.375060 |
| C | 2.897721 | -1.953626 | -0.393347 |
| H | 2.021646 | -2.546323 | -0.118611 |
| H | 2.829332 | -1.744285 | -1.468964 |
| C | 2.377671 | -0.596573 | 1.748863 |
| H | 2.162762 | 0.436998 | 2.034164 |
| H | 1.461292 | -1.183481 | 1.841545 |
| H | 3.093701 | -0.993618 | 2.486275 |
| C | 4.128807 | 0.201682 | 0.043010 |
| H | 4.092004 | 0.496472 | -1.012962 |
| H | 4.127062 | 1.116767 | 0.641580 |
| C | 4.203184 | -2.760986 | -0.153755 |
| C | 5.444909 | -0.589841 | 0.278373 |

| | | | |
|---|---|---|---|
| C | 5.436047 | -1.914137 | -0.496879 |
| H | 6.350183 | -2.478215 | -0.285754 |
| H | 5.436839 | -1.700864 | -1.573613 |
| H | 4.247486 | -3.068306 | 0.897546 |
| H | 4.181484 | -3.675303 | -0.754923 |
| H | 5.553850 | -0.792620 | 1.350154 |
| H | 6.298114 | 0.027545 | -0.019432 |

***re-TS for 1-methylcyclohexyl radical (equatorial) addition to N-sulfinyl imine 4 (TS2$_{tert}$-eq)***

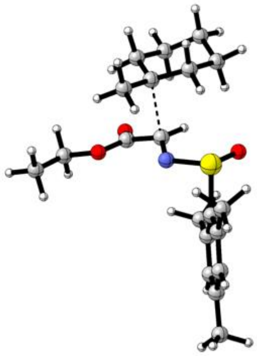

*Charge:* 0

*Multiplicity:* 2

*E* = -1459.11655137

*H* = -1458.623267

*G* = -1458.712555

*Cartesian coordinates:*

| | | | |
|---|---|---|---|
| C | -3.428852 | -0.897323 | 1.034540 |
| C | -4.724118 | -0.400818 | 0.860693 |
| C | -5.087641 | 0.332637 | -0.269322 |
| C | -4.115826 | 0.579823 | -1.242645 |
| C | -2.804265 | 0.117153 | -1.120534 |
| C | -2.482378 | -0.624357 | 0.031725 |
| H | -5.465716 | -0.597791 | 1.628075 |
| H | -4.383270 | 1.153962 | -2.124377 |
| C | -3.102501 | -1.690043 | 2.282348 |
| H | -2.752362 | -2.697324 | 2.041944 |
| H | -2.320888 | -1.207629 | 2.874596 |
| H | -3.989131 | -1.785445 | 2.910205 |
| H | -2.312362 | 0.783493 | -3.102515 |
| H | -1.155273 | 1.278675 | -1.864483 |
| H | -1.158684 | -0.386741 | -2.436224 |
| C | -1.800577 | 0.460043 | -2.195061 |
| C | -6.502291 | 0.821584 | -0.454307 |
| H | -6.526168 | 1.779956 | -0.977919 |
| H | -7.077925 | 0.107547 | -1.052945 |
| H | -7.013747 | 0.936498 | 0.503467 |
| S | -0.828423 | -1.358419 | 0.274268 |
| O | -0.465169 | -2.166917 | -0.962497 |
| N | 0.033948 | 0.122018 | 0.343312 |
| C | 1.131073 | 0.198067 | -0.380779 |
| H | 1.336946 | -0.516386 | -1.175508 |
| C | 1.721878 | 1.545514 | -0.637552 |
| O | 2.376226 | 1.779116 | -1.636094 |
| H | 3.063749 | 3.722225 | 0.019674 |
| H | 1.573270 | 4.185046 | -0.817865 |
| O | 1.450158 | 2.443958 | 0.309339 |
| C | 1.978006 | 3.788106 | 0.115348 |
| C | 1.559797 | 4.608671 | 1.315788 |
| H | 1.936361 | 5.628711 | 1.207770 |
| H | 0.471550 | 4.649630 | 1.399206 |
| H | 1.967584 | 4.185949 | 2.236630 |
| C | 2.865049 | -0.592825 | 0.908187 |
| C | 2.566780 | 0.118000 | 2.193806 |
| H | 2.698592 | 1.196969 | 2.108428 |
| H | 1.551207 | -0.086214 | 2.538156 |
| H | 3.261679 | -0.247097 | 2.963335 |
| C | 4.125341 | -0.185422 | 0.191965 |
| H | 4.176381 | 0.899663 | 0.073270 |
| H | 4.949075 | -0.444271 | 0.880268 |
| C | 2.570692 | -2.071194 | 0.903295 |
| H | 1.556342 | -2.264023 | 1.263628 |
| H | 3.232997 | -2.498042 | 1.676430 |
| C | 2.842140 | -2.791511 | -0.424516 |
| H | 2.771388 | -3.871308 | -0.263184 |
| H | 2.057289 | -2.537192 | -1.142158 |
| C | 4.357644 | -0.902738 | -1.145081 |
| H | 3.637353 | -0.533416 | -1.881165 |
| H | 5.351473 | -0.644896 | -1.522503 |
| C | 4.216772 | -2.422078 | -0.994144 |
| H | 5.002387 | -2.794129 | -0.323683 |
| H | 4.366967 | -2.912839 | -1.960550 |

***re-TS for 1-methylcyclohexyl radical (axial) addition to N-sulfinyl imine 4 (TS2$_{tert}$-ax)***

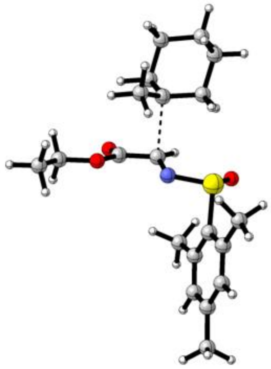

*Charge:* 0

*Multiplicity:* 2

*E* = -1459.11580089

*H* = -1458.622207

*G* = -1458.712723

*Cartesian coordinates:*

| | | | |
|---|---|---|---|
| C | -3.393794 | -1.176344 | 0.990206 |
| C | -4.725392 | -0.750406 | 0.977596 |
| C | -5.223939 | 0.082969 | -0.024145 |
| C | -4.352897 | 0.505280 | -1.032023 |
| C | -3.011513 | 0.119880 | -1.067959 |
| C | -2.551675 | -0.728014 | -0.042414 |
| H | -5.387311 | -1.083391 | 1.770468 |
| H | -4.725683 | 1.159294 | -1.814368 |
| C | -2.920045 | -2.083827 | 2.105770 |
| H | -2.542429 | -3.034923 | 1.721243 |
| H | -2.114742 | -1.625554 | 2.685215 |
| H | -3.742512 | -2.302140 | 2.787903 |
| H | -2.728936 | 1.053113 | -2.981722 |
| H | -1.494322 | 1.462219 | -1.788281 |
| H | -1.463594 | -0.117480 | -2.564468 |
| C | -2.124045 | 0.652452 | -2.167185 |
| C | -6.674421 | 0.495685 | -0.039904 |
| H | -6.793849 | 1.512353 | -0.421350 |
| H | -7.253333 | -0.167618 | -0.691380 |
| H | -7.115056 | 0.445395 | 0.957762 |
| S | -0.843097 | -1.371327 | -0.011361 |
| O | -0.523875 | -1.975458 | -1.371021 |
| N | -0.069151 | 0.150591 | 0.207455 |
| C | 0.930623 | 0.414172 | -0.597594 |
| H | 1.113921 | -0.167733 | -1.500665 |
| C | 1.456891 | 1.809320 | -0.671979 |
| O | 2.044822 | 2.222516 | -1.653788 |
| H | 2.774496 | 3.891452 | 0.249581 |
| H | 1.226468 | 4.442214 | -0.411433 |
| O | 1.207333 | 2.536943 | 0.417341 |
| C | 1.695627 | 3.909242 | 0.418154 |
| C | 1.337904 | 4.509727 | 1.760100 |
| H | 1.687807 | 5.544100 | 1.800487 |
| H | 0.256444 | 4.505268 | 1.912934 |
| H | 1.810337 | 3.953440 | 2.572691 |
| C | 2.864720 | -0.476837 | 0.321916 |
| C | 2.498805 | -1.936537 | 0.271742 |
| H | 1.730571 | -2.173616 | 1.012076 |
| H | 2.102821 | -2.197133 | -0.714395 |
| C | 2.875705 | 0.161089 | 1.677122 |
| H | 3.123563 | 1.222162 | 1.635425 |
| H | 1.910315 | 0.040659 | 2.174575 |
| H | 3.630340 | -0.332294 | 2.305903 |
| C | 3.934821 | -0.086652 | -0.658172 |
| H | 3.593998 | -0.281334 | -1.681242 |
| H | 4.171992 | 0.976405 | -0.587876 |
| C | 3.763738 | -2.797938 | 0.531633 |
| C | 5.206707 | -0.939801 | -0.400014 |
| C | 4.884274 | -2.437574 | -0.450521 |
| H | 5.781775 | -3.023181 | -0.228078 |
| H | 4.573292 | -2.705862 | -1.468031 |
| H | 4.108322 | -2.639338 | 1.559544 |
| H | 3.495099 | -3.854822 | 0.445734 |
| H | 5.620508 | -0.683079 | 0.581654 |
| H | 5.964518 | -0.678799 | -1.144548 |

***(R)-configured adduct of 4 with 1-methylcyclohexyl radical (equatorial) (12$_{tert}$-eq)***

*Charge:* 0

*Multiplicity:* 2

*E* = -1459.13999376

*H* = -1458.643184

*G* = -1458.731493

*Cartesian coordinates:*

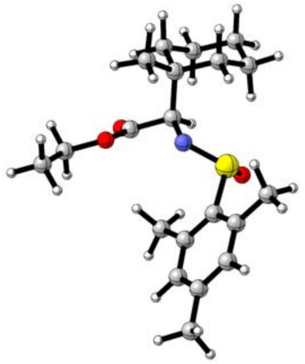

| | | | |
|---|---|---|---|
| C | -3.135864 | -1.570564 | 0.630270 |
| C | -4.465819 | -1.144582 | 0.684176 |
| C | -4.909709 | -0.019257 | -0.013061 |
| C | -3.987219 | 0.701737 | -0.776388 |
| C | -2.643812 | 0.331786 | -0.862150 |
| C | -2.248171 | -0.811095 | -0.147391 |
| H | -5.170437 | -1.711250 | 1.283774 |
| H | -4.318453 | 1.581753 | -1.318517 |
| C | -2.709201 | -2.800083 | 1.399861 |
| H | -2.298311 | -3.567195 | 0.737654 |
| H | -1.941957 | -2.563476 | 2.141425 |
| H | -3.561570 | -3.233560 | 1.923830 |
| H | -2.226058 | 1.804267 | -2.370926 |
| H | -1.122731 | 1.832669 | -0.993331 |
| H | -0.970643 | 0.567750 | -2.218588 |
| C | -1.684886 | 1.176600 | -1.661977 |
| C | -6.359184 | 0.394375 | 0.030001 |
| H | -6.464677 | 1.479749 | -0.031306 |
| H | -6.903310 | -0.035940 | -0.817469 |
| H | -6.845950 | 0.048931 | 0.944137 |
| S | -0.535229 | -1.428507 | -0.265274 |
| O | -0.200466 | -1.737550 | -1.711522 |
| N | 0.262665 | -0.162856 | 0.408607 |
| C | 1.565076 | 0.168280 | -0.140519 |
| H | 1.739393 | -0.323517 | -1.099025 |
| C | 1.609103 | 1.665724 | -0.468671 |
| O | 2.239590 | 2.103738 | -1.405887 |
| H | 1.926954 | 4.218483 | 0.218738 |
| H | 0.545115 | 4.036611 | -0.875031 |
| O | 0.904156 | 2.415925 | 0.375849 |
| C | 0.900079 | 3.856084 | 0.141621 |
| C | -0.007457 | 4.471790 | 1.183560 |
| H | -0.039180 | 5.554900 | 1.043297 |
| H | -1.023634 | 4.081956 | 1.092359 |
| H | 0.359925 | 4.264439 | 2.190888 |
| C | 2.734605 | -0.235964 | 0.855001 |
| C | 2.568490 | 0.494755 | 2.195588 |
| H | 2.667635 | 1.576578 | 2.081700 |
| H | 1.592160 | 0.288638 | 2.638495 |
| H | 3.340309 | 0.158336 | 2.892855 |
| C | 4.109774 | 0.114125 | 0.235484 |
| H | 4.166148 | 1.182210 | 0.011029 |
| H | 4.858949 | -0.077760 | 1.013305 |
| C | 2.683228 | -1.765232 | 1.093256 |
| H | 1.710111 | -2.048380 | 1.504628 |
| H | 3.419499 | -1.988251 | 1.874484 |
| C | 3.020975 | -2.605526 | -0.146872 |
| H | 3.008734 | -3.665970 | 0.124070 |
| H | 2.252124 | -2.478911 | -0.916826 |
| C | 4.472602 | -0.713767 | -1.006146 |
| H | 3.805260 | -0.459132 | -1.836545 |
| H | 5.482167 | -0.445783 | -1.333450 |
| C | 4.391339 | -2.219341 | -0.719805 |
| H | 5.169899 | -2.487233 | 0.006241 |
| H | 4.597610 | -2.790771 | -1.630312 |

***(R)-configured adduct of 4 with 1-methylcyclohexyl radical (axial) ($12_{tert}$-ax)***

*Charge:* 0

*Multiplicity:* 2

*E* = -1459.13909630

*H* = -1458.642388

*G* = -1458.731350

*Cartesian coordinates:*

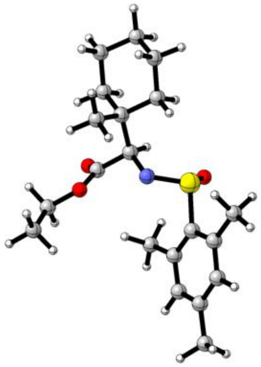

| | | | |
|---|---|---|---|
| C | -3.170409 | -1.701420 | 0.565014 |
| C | -4.495012 | -1.309897 | 0.779012 |
| C | -5.011899 | -0.129259 | 0.242397 |
| C | -4.169180 | 0.684023 | -0.520626 |
| C | -2.834871 | 0.351885 | -0.761035 |
| C | -2.364205 | -0.849931 | -0.205858 |
| H | -5.136923 | -1.948001 | 1.377301 |
| H | -4.557458 | 1.607673 | -0.938032 |
| C | -2.663054 | -2.992848 | 1.165705 |
| H | -2.297846 | -3.679177 | 0.396781 |
| H | -1.839983 | -2.814925 | 1.862502 |
| H | -3.462133 | -3.496823 | 1.710119 |
| H | -2.569918 | 1.977298 | -2.142105 |
| H | -1.351339 | 1.892300 | -0.868641 |
| H | -1.290280 | 0.759799 | -2.225664 |
| C | -1.960670 | 1.293430 | -1.550008 |
| C | -6.455971 | 0.249616 | 0.454264 |
| H | -6.573937 | 1.332240 | 0.538580 |
| H | -7.064853 | -0.080588 | -0.394046 |
| H | -6.863099 | -0.215588 | 1.354036 |
| S | -0.662111 | -1.420358 | -0.538859 |
| O | -0.465080 | -1.590603 | -2.032476 |
| N | 0.177147 | -0.198985 | 0.167302 |
| C | 1.400005 | 0.232150 | -0.481109 |
| H | 1.475229 | -0.133358 | -1.509915 |
| C | 1.384250 | 1.761548 | -0.609053 |
| O | 1.853019 | 2.336159 | -1.566819 |
| H | 1.799427 | 4.206116 | 0.377039 |
| H | 0.273008 | 4.139124 | -0.519966 |
| O | 0.821116 | 2.373386 | 0.430774 |
| C | 0.774252 | 3.831376 | 0.399864 |
| C | 0.025792 | 4.276652 | 1.636699 |
| H | -0.032833 | 5.367585 | 1.653093 |
| H | -0.990320 | 3.876121 | 1.639668 |
| H | 0.537939 | 3.944531 | 2.542290 |
| C | 2.688786 | -0.277452 | 0.290844 |
| C | 2.656156 | -1.826553 | 0.244342 |
| H | 1.823035 | -2.193313 | 0.850577 |
| H | 2.463030 | -2.141187 | -0.789088 |
| C | 2.678863 | 0.224668 | 1.743801 |
| H | 2.747108 | 1.313425 | 1.789227 |
| H | 1.761010 | -0.079509 | 2.250184 |
| H | 3.522744 | -0.183522 | 2.300751 |
| C | 3.939688 | 0.228008 | -0.468860 |
| H | 3.814546 | 0.011751 | -1.536714 |
| H | 4.016313 | 1.314932 | -0.378711 |
| C | 3.963118 | -2.482739 | 0.712913 |
| C | 5.244301 | -0.426426 | 0.010741 |
| C | 5.168588 | -1.955009 | -0.072786 |
| H | 6.094032 | -2.404478 | 0.300830 |
| H | 5.071845 | -2.253642 | -1.124826 |
| H | 4.114087 | -2.296804 | 1.782064 |
| H | 3.876690 | -3.567797 | 0.598212 |
| H | 5.457341 | -0.126456 | 1.042877 |
| H | 6.073681 | -0.051578 | -0.597195 |

***Reduced (R)-configured adduct of 4 with 1-methylcyclohexyl radical ($5_{tert}^{-}$)***

*Charge:* −1

*Multiplicity:* 1

*E* = -1459.29514860

*H* = -1458.799478

*G* = -1458.887306

*Cartesian coordinates:*

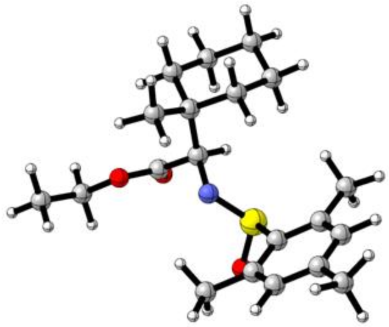

| | | | |
|---|---|---|---|
| C | -3.099684 | -0.951175 | -0.524884 |
| C | -4.348668 | -0.995210 | 0.106983 |
| C | -4.898639 | 0.123283 | 0.732106 |
| C | -4.158773 | 1.309032 | 0.724117 |
| C | -2.906711 | 1.405331 | 0.110190 |
| C | -2.383410 | 0.258960 | -0.520584 |
| H | -4.901187 | -1.930415 | 0.109156 |
| H | -4.564997 | 2.188253 | 1.216816 |
| C | -2.563894 | -2.211449 | -1.171313 |
| H | -2.392084 | -2.075351 | -2.242314 |
| H | -1.609413 | -2.512620 | -0.733524 |
| H | -3.270083 | -3.034105 | -1.043158 |
| H | -2.740953 | 3.468043 | 0.704766 |
| H | -1.197670 | 2.578429 | 0.668284 |
| H | -1.931831 | 3.074872 | -0.836275 |
| C | -2.158524 | 2.715577 | 0.168889 |
| C | -6.263491 | 0.066796 | 1.375407 |
| H | -6.313570 | 0.706451 | 2.260208 |
| H | -7.036195 | 0.412836 | 0.680009 |
| H | -6.522932 | -0.951799 | 1.673109 |
| S | -0.760644 | 0.262696 | -1.375535 |
| O | -0.839741 | 1.490524 | -2.335513 |
| N | 0.199154 | 0.495440 | -0.076451 |
| C | 1.496764 | -0.160710 | -0.239651 |
| H | 1.496663 | -0.847665 | -1.095701 |
| C | 2.549671 | 0.869731 | -0.648771 |
| O | 3.121877 | 0.864912 | -1.725977 |
| H | 4.662969 | 2.443335 | -0.298027 |
| H | 3.330196 | 3.386519 | -0.979058 |
| O | 2.760991 | 1.826693 | 0.268672 |
| C | 3.689160 | 2.886532 | -0.076198 |
| C | 3.752742 | 3.830237 | 1.106755 |
| H | 4.441834 | 4.649651 | 0.887107 |
| H | 2.768601 | 4.254780 | 1.318312 |
| H | 4.108164 | 3.310768 | 1.999690 |
| C | 1.894766 | -1.008654 | 1.020739 |
| C | 1.767145 | -0.192519 | 2.317162 |
| H | 2.470700 | 0.640690 | 2.336434 |
| H | 0.757948 | 0.211281 | 2.416232 |
| H | 1.971223 | -0.834909 | 3.180220 |
| C | 3.346841 | -1.532605 | 0.892873 |
| H | 4.038540 | -0.691334 | 0.767815 |
| H | 3.615308 | -2.004415 | 1.846934 |
| C | 0.933129 | -2.220580 | 1.109215 |
| H | -0.095764 | -1.849519 | 1.110777 |
| H | 1.098764 | -2.708232 | 2.078290 |
| C | 1.126467 | -3.267871 | 0.002635 |
| H | 0.458435 | -4.116767 | 0.184682 |
| H | 0.832000 | -2.851199 | -0.967166 |
| C | 3.548360 | -2.562360 | -0.228852 |
| H | 3.393645 | -2.089722 | -1.204968 |
| H | 4.584671 | -2.916733 | -0.217903 |
| C | 2.583002 | -3.745458 | -0.071792 |
| H | 2.829883 | -4.286057 | 0.851868 |
| H | 2.710808 | -4.454656 | -0.896509 |

***Sodium cyclohexyloxalate (axial) ($3_{sec}$·$Na^+$-ax)***

*Charge:* 0

*Multiplicity:* 1

*E* = -775.031095063

*H* = -774.830005

*G* = -774.887304

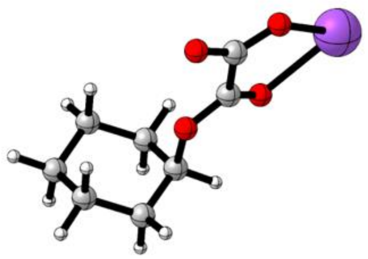

*Cartesian coordinates:*

| | | | | | | | |
|---|---|---|---|---|---|---|---|
| O | 0.883375 | -3.945626 | 2.625943 | H | -1.349074 | -3.544996 | 4.047418 |
| O | 0.943800 | -2.073563 | 3.868485 | C | -0.616354 | -5.862104 | 0.928183 |
| C | 1.449478 | -3.117280 | 3.488507 | H | -1.480552 | -3.985868 | 0.274059 |
| C | 2.865399 | -3.602410 | 3.990770 | H | 0.271814 | -4.043485 | 0.111441 |
| O | 3.461229 | -2.746659 | 4.693345 | C | -1.729041 | -6.273801 | 1.902923 |
| O | 3.231775 | -4.740633 | 3.661117 | H | -2.384712 | -5.838326 | 3.931226 |
| Na | 2.427619 | -0.767321 | 5.278450 | H | -0.639106 | -5.907475 | 3.732935 |
| C | -0.447184 | -3.606377 | 2.090463 | H | -0.766039 | -6.335751 | -0.046609 |
| C | -1.519489 | -4.043039 | 3.090067 | H | 0.347843 | -6.216090 | 1.309076 |
| C | -0.561082 | -4.337019 | 0.756416 | H | -1.728602 | -7.359673 | 2.039648 |
| C | -1.566295 | -5.568538 | 3.257195 | H | -2.703731 | -6.008590 | 1.472691 |
| H | -2.481684 | -3.682109 | 2.708239 | H | -0.472655 | -2.525264 | 1.947882 |

***Sodium cyclohexyloxalate (equatorial) ($3_{sec}$·$Na^+$-eq)***

*Charge:* 0

*Multiplicity:* 1

*E* = -775.031660733

*H* = -774.830536

*G* = -774.885745

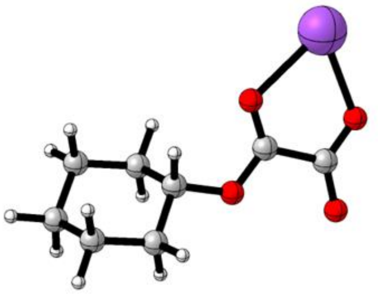

*Cartesian coordinates:*

| | | | | | | | |
|---|---|---|---|---|---|---|---|
| O | -0.952339 | -2.128458 | 1.219159 | C | -1.581559 | -5.249061 | 3.304591 |
| O | -0.008410 | -1.092114 | 2.976704 | H | -2.748869 | -3.742080 | 2.268330 |
| C | -0.583842 | -1.053976 | 1.901798 | H | -1.793612 | -3.100617 | 3.609348 |
| C | -0.959483 | 0.306404 | 1.193222 | C | -0.401432 | -5.860429 | 1.148146 |
| O | -0.635887 | 1.318039 | 1.865395 | H | -1.541337 | -4.368573 | 0.061306 |
| O | -1.517066 | 0.242693 | 0.087222 | H | 0.205588 | -4.135887 | -0.042165 |
| Na | 0.421786 | 1.088128 | 3.916644 | C | -1.497080 | -6.255730 | 2.148658 |
| C | -0.681695 | -3.450775 | 1.794941 | H | -2.397889 | -5.511141 | 3.983765 |
| C | -1.784806 | -3.817005 | 2.784613 | H | -0.656256 | -5.292828 | 3.892559 |
| C | -0.606157 | -4.427820 | 0.629864 | H | -0.383046 | -6.555884 | 0.304247 |

| | | | |
|---|---|---|---|
| H | 0.578204 | -5.932322 | 1.637077 |
| H | -1.308755 | -7.261493 | 2.536559 |
| H | -2.463438 | -6.290604 | 1.629939 |
| H | 0.280782 | -3.397062 | 2.309824 |

***Cyclohexyl oxyacyl radical (axial) – $CO_2$ – sodium complex ($10_{sec}$·$CO_2$·$Na^+$-ax)***

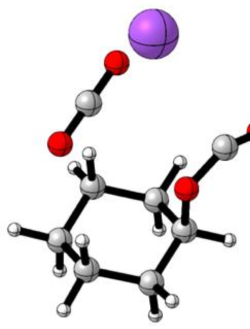

*Charge:* 1

*Multiplicity:* 2

*E* = -774.829121888

*H* = -774.628269

*G* = -774.689503

*Cartesian coordinates:*

| | | | |
|---|---|---|---|
| O | 0.902691 | -2.820189 | 1.792075 |
| O | 0.560132 | -0.687473 | 2.527123 |
| C | 1.218636 | -1.632908 | 2.207618 |
| C | 3.169418 | -4.184028 | 3.665597 |
| O | 3.228412 | -3.206233 | 4.289454 |
| O | 3.113214 | -5.162922 | 3.046381 |
| Na | 0.809887 | -1.876200 | 5.849445 |
| C | -0.570387 | -3.137582 | 1.551687 |
| C | -1.267398 | -3.354162 | 2.887303 |
| C | -0.571220 | -4.374205 | 0.669392 |
| C | -0.740515 | -4.592224 | 3.624251 |
| H | -2.331694 | -3.487819 | 2.658578 |
| H | -1.196014 | -2.451927 | 3.496825 |
| C | -0.063927 | -5.621739 | 1.408435 |
| H | -1.608280 | -4.527444 | 0.348310 |
| H | 0.016710 | -4.180695 | -0.231376 |
| C | -0.813055 | -5.839428 | 2.731641 |
| H | -1.311726 | -4.740430 | 4.544726 |
| H | 0.298788 | -4.423330 | 3.925704 |
| H | -0.173404 | -6.495791 | 0.760268 |
| H | 1.005028 | -5.509523 | 1.612206 |
| H | -0.399586 | -6.704412 | 3.258707 |
| H | -1.865406 | -6.068738 | 2.520973 |
| H | -0.965841 | -2.265045 | 1.030443 |

***Cyclohexyl oxyacyl radical (axial) ($10_{sec}$-ax)***

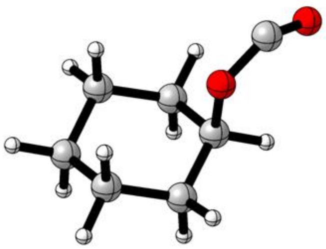

*Charge:* 0

*Multiplicity:* 2

*E* = -423.918307204

*H* = -423.737403

*G* = -423.781080

*Cartesian coordinates:*

| | | | |
|---|---|---|---|
| O | 0.800730 | -3.364889 | 2.191273 |
| O | 0.736394 | -1.207635 | 2.929917 |
| C | 1.242986 | -2.272435 | 2.730857 |
| C | -0.635017 | -3.388200 | 1.687085 |
| C | -1.563583 | -3.633280 | 2.869368 |
| C | -0.677814 | -4.492049 | 0.643835 |
| C | -1.382403 | -5.032603 | 3.474303 |
| H | -2.586628 | -3.521951 | 2.490136 |
| H | -1.421080 | -2.852078 | 3.620248 |
| C | -0.503648 | -5.888412 | 1.259338 |
| H | -1.656379 | -4.423028 | 0.153986 |
| H | 0.077139 | -4.298207 | -0.122431 |

| | | | |
|---|---|---|---|
| C | -1.502444 | -6.124147 | 2.401276 |
| H | -2.123680 | -5.185896 | 4.263612 |
| H | -0.396778 | -5.096396 | 3.948680 |
| H | -0.624626 | -6.646511 | 0.480407 |
| H | 0.517338 | -5.988243 | 1.643658 |
| H | -1.339721 | -7.110158 | 2.846751 |
| H | -2.522826 | -6.122998 | 1.996639 |
| H | -0.801403 | -2.405529 | 1.244239 |

***Cyclohexyl oxyacyl radical (equatorial) ($10_{sec}$-eq)***

*Charge:* 0

*Multiplicity:* 2

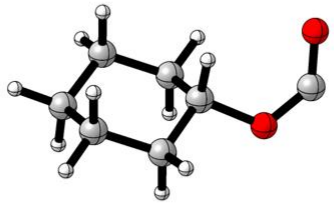

*E* = -423.918785154

*H* = -423.737782

*G* = -423.781655

*Cartesian coordinates:*

| | | | |
|---|---|---|---|
| O | -1.210559 | -2.143192 | 0.690232 |
| O | -0.209807 | -0.605635 | 2.047243 |
| C | -0.847126 | -0.966481 | 1.102682 |
| C | -0.804648 | -3.346308 | 1.510197 |
| C | -1.800218 | -3.536596 | 2.643631 |
| C | -0.769714 | -4.519940 | 0.549601 |
| C | -1.473429 | -4.829841 | 3.412635 |
| H | -2.806388 | -3.603828 | 2.215601 |
| H | -1.778474 | -2.672748 | 3.312498 |
| C | -0.445336 | -5.814182 | 1.317646 |
| H | -1.750872 | -4.610078 | 0.070438 |
| H | -0.032041 | -4.335832 | -0.235445 |
| C | -1.431813 | -6.041628 | 2.471462 |
| H | -2.215853 | -4.978494 | 4.201413 |
| H | -0.501761 | -4.720643 | 3.909536 |
| H | -0.461284 | -6.659201 | 0.623959 |
| H | 0.574195 | -5.752376 | 1.717225 |
| H | -1.156675 | -6.941785 | 3.029153 |
| H | -2.434383 | -6.215076 | 2.061143 |
| H | 0.192158 | -3.122890 | 1.897996 |

***TS for $CO_2$ elimination from cyclohexyl oxyacyl radical (axial) ($TS1_{sec}$-ax)***

*Charge:* 0

*Multiplicity:* 2

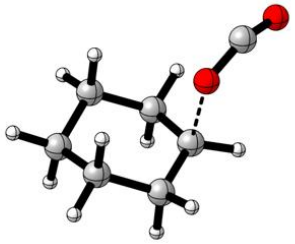

*E* = -423.905169774

*H* = -423.727627

*G* = -423.773202

*Cartesian coordinates:*

| | | | |
|---|---|---|---|
| O | 0.928723 | -3.351707 | 2.195931 |
| O | 1.022420 | -1.302779 | 3.255659 |
| C | 1.317534 | -2.369912 | 2.811540 |
| C | -0.933718 | -3.396647 | 1.558954 |
| C | -1.733964 | -3.627084 | 2.801791 |
| C | -0.839162 | -4.510652 | 0.567786 |
| C | -1.479029 | -5.004436 | 3.433642 |
| H | -2.792197 | -3.561054 | 2.496637 |
| H | -1.576000 | -2.817781 | 3.518422 |
| C | -0.598588 | -5.879280 | 1.226370 |

| | | | | | | | |
|---|---|---|---|---|---|---|---|
| H | -1.802820 | -4.529887 | 0.032192 | H | -0.692327 | -6.666328 | 0.473087 |
| H | -0.077391 | -4.286138 | -0.182885 | H | 0.428855 | -5.914637 | 1.602489 |
| C | -1.572569 | -6.123736 | 2.387311 | H | -1.364471 | -7.091375 | 2.853567 |
| H | -2.195513 | -5.171599 | 4.242505 | H | -2.598020 | -6.172989 | 1.998879 |
| H | -0.480218 | -5.009655 | 3.882871 | H | -0.941131 | -2.387746 | 1.157594 |

***TS for $CO_2$ elimination from cyclohexyl oxyacyl radical (equatorial) ($TS1_{sec}$-eq)***

*Charge:* 0

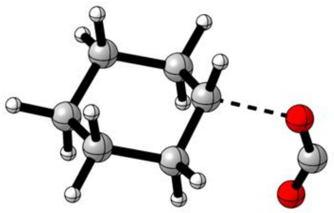

*Multiplicity:* 2

*E* = -423.902449587

*H* = -423.724539

*G* = -423.769742

*Cartesian coordinates:*

| | | | | | | | |
|---|---|---|---|---|---|---|---|
| C | -2.011489 | -0.773970 | 0.008461 | C | -2.101339 | -1.707685 | 1.247638 |
| C | -0.736882 | 0.002424 | 0.105665 | H | -3.054254 | -2.243959 | 1.219143 |
| H | -2.054792 | -1.354818 | -0.914934 | H | -1.307196 | -2.460783 | 1.190076 |
| O | -0.724274 | 1.236669 | -1.418598 | C | -0.663746 | -0.088342 | 2.570767 |
| O | -2.572118 | 2.579687 | -1.054511 | H | 0.199169 | -0.763973 | 2.576500 |
| C | -1.590728 | 2.093357 | -1.525632 | H | -0.600455 | 0.520064 | 3.477566 |
| H | -2.867673 | -0.091985 | 0.024965 | C | -1.961137 | -0.908171 | 2.550282 |
| C | -0.574604 | 0.844578 | 1.330941 | H | -1.987782 | -1.586013 | 3.408826 |
| H | -1.384068 | 1.579188 | 1.390444 | H | -2.818101 | -0.231112 | 2.652746 |
| H | 0.375347 | 1.382562 | 1.321717 | H | 0.157032 | -0.507135 | -0.241944 |

***Cyclohexyl radical — $CO_2$ complex ($11_{sec}\cdot CO_2$)***

*Charge:* 0

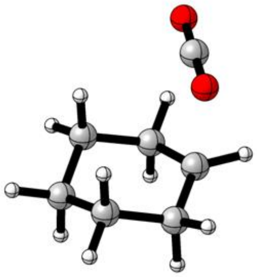

*Multiplicity:* 2

*E* = -423.945113006

*H* = -423.766817

*G* = -423.817706

*Cartesian coordinates:*

| | | | | | | | |
|---|---|---|---|---|---|---|---|
| O | 1.717265 | 0.081765 | -2.128994 | C | -0.386246 | -0.121339 | 1.557480 |
| O | 2.413380 | -1.098909 | -0.254368 | C | -1.145681 | 1.225342 | -0.504159 |
| C | 2.053440 | -0.500040 | -1.181891 | C | -1.034993 | -1.380610 | 0.946569 |
| C | -0.106823 | 0.925619 | 0.527895 | H | -1.084919 | 0.288088 | 2.310951 |

| | | | |
|---|---|---|---|
| H | 0.525329 | -0.383175 | 2.103016 |
| C | -1.780263 | -0.057283 | -1.080400 |
| H | -1.952600 | 1.825448 | -0.043464 |
| H | -0.730543 | 1.843879 | -1.305569 |
| C | -2.215954 | -1.015042 | 0.036939 |
| H | -1.359738 | -2.055116 | 1.745094 |
| H | -0.283309 | -1.921473 | 0.359357 |
| H | -2.631868 | 0.203492 | -1.716647 |
| H | -1.046828 | -0.561749 | -1.720569 |
| H | -2.654326 | -1.921277 | -0.393433 |
| H | -3.001871 | -0.537798 | 0.637477 |
| H | 0.701419 | 1.631725 | 0.691535 |

***Cycloxehyl radical ($11_{sec}$)***

*Charge:* 0

*Multiplicity:* 2

*E* = -235.290758250

*H* = -235.129144

*G* = -235.165865

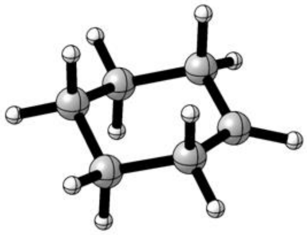

*Cartesian coordinates:*

| | | | |
|---|---|---|---|
| C | -1.030131 | -3.392864 | 1.437102 |
| C | -1.785464 | -3.576131 | 2.714228 |
| C | -0.927865 | -4.546903 | 0.492167 |
| C | -1.428456 | -4.906290 | 3.411065 |
| H | -2.870532 | -3.590018 | 2.498476 |
| H | -1.624159 | -2.730694 | 3.390153 |
| C | -0.583570 | -5.862670 | 1.221946 |
| H | -1.899460 | -4.689238 | -0.017595 |
| H | -0.200220 | -4.342541 | -0.299305 |
| C | -1.503137 | -6.085261 | 2.430798 |
| H | -2.096991 | -5.071733 | 4.262041 |
| H | -0.409881 | -4.838065 | 3.812316 |
| H | -0.654833 | -6.704205 | 0.525375 |
| H | 0.456863 | -5.819188 | 1.566563 |
| H | -1.236765 | -7.016888 | 2.940613 |
| H | -2.538105 | -6.200537 | 2.081717 |
| H | -0.787063 | -2.392537 | 1.093891 |

***Cyclohexyl radical (axial) — N-sulfinyl imine 4 re-precomplex ($11_{sec}$·4-ax)***

*Charge:* 0

*Multiplicity:* 2

*E* = -1419.78229489

*H* = -1419.318052

*G* = -1419.408936

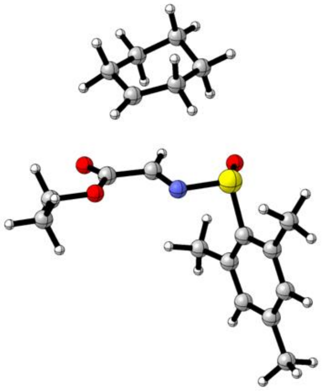

*Cartesian coordinates:*

| | | | |
|---|---|---|---|
| C | -3.280278 | -0.877590 | 1.078920 |
| C | -4.596059 | -0.414726 | 0.993619 |
| C | -5.059829 | 0.290306 | -0.117693 |
| C | -4.168721 | 0.544051 | -1.163419 |
| C | -2.840314 | 0.115364 | -1.132907 |
| C | -2.416770 | -0.599987 | 0.003634 |
| H | -5.273840 | -0.615698 | 1.816814 |
| H | -4.514219 | 1.097600 | -2.030904 |

| | | | |
|---|---|---|---|
| C | -2.843975 | -1.637728 | 2.313449 |
| H | -2.489527 | -2.642004 | 2.066874 |
| H | -2.032718 | -1.126416 | 2.837828 |
| H | -3.679660 | -1.739218 | 3.006608 |
| H | -2.517786 | 0.801133 | -3.141616 |
| H | -1.253978 | 1.277171 | -2.006590 |
| H | -1.316546 | -0.381943 | -2.589884 |
| C | -1.930741 | 0.464845 | -2.286173 |
| C | -6.495759 | 0.742382 | -0.204683 |
| H | -6.583833 | 1.682492 | -0.753760 |
| H | -7.098904 | -0.003375 | -0.733461 |
| H | -6.932816 | 0.876669 | 0.786810 |
| S | -0.736315 | -1.291222 | 0.146202 |
| O | -0.381189 | -2.015445 | -1.139206 |
| N | 0.084006 | 0.246462 | 0.241398 |
| C | 1.057986 | 0.409077 | -0.570006 |
| H | 1.318290 | -0.305120 | -1.352493 |
| C | 1.822917 | 1.693547 | -0.634642 |
| O | 2.553101 | 1.945616 | -1.573337 |
| H | 3.379834 | 3.569380 | 0.350538 |
| H | 2.026488 | 4.332899 | -0.500923 |
| O | 1.595095 | 2.507299 | 0.393808 |

| | | | |
|---|---|---|---|
| C | 2.309096 | 3.778920 | 0.396461 |
| C | 1.922788 | 4.506390 | 1.665181 |
| H | 2.440176 | 5.468063 | 1.703071 |
| H | 0.846973 | 4.692127 | 1.695730 |
| H | 2.205837 | 3.927398 | 2.546937 |
| C | 3.360121 | -0.321068 | 0.848814 |
| C | 4.483887 | -0.383842 | -0.130488 |
| H | 4.566049 | 0.560061 | -0.677768 |
| H | 5.426665 | -0.483215 | 0.438698 |
| C | 2.804700 | -1.574113 | 1.438297 |
| H | 1.820688 | -1.384027 | 1.880698 |
| H | 3.452468 | -1.863504 | 2.286109 |
| C | 2.749512 | -2.747180 | 0.442644 |
| H | 2.530690 | -3.673482 | 0.982220 |
| H | 1.925266 | -2.590311 | -0.260375 |
| C | 4.371756 | -1.575164 | -1.098064 |
| H | 3.572393 | -1.376748 | -1.822027 |
| H | 5.298757 | -1.672122 | -1.670733 |
| C | 4.058220 | -2.876109 | -0.347663 |
| H | 4.882916 | -3.107694 | 0.339279 |
| H | 3.989588 | -3.710723 | -1.052354 |
| H | 3.216468 | 0.600909 | 1.403841 |

***Cyclohexyl radical (equatorial) — N-sulfinyl imine 4 re-precomplex ($11_{sec}$·4-eq)***

*Charge:* 0

*Multiplicity:* 2

*E* = -1419.77974483

*H* = -1419.315239

*G* = -1419.407580

*Cartesian coordinates:*

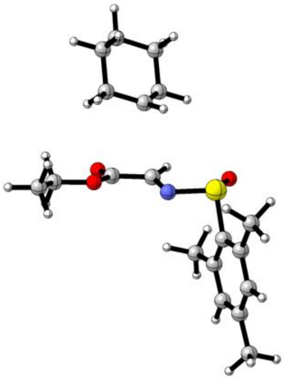

| | | | |
|---|---|---|---|
| C | -3.600894 | -0.730433 | 1.259921 |
| C | -4.901750 | -0.234768 | 1.138925 |
| C | -5.373210 | 0.318143 | -0.052387 |
| C | -4.504530 | 0.382740 | -1.144585 |
| C | -3.191081 | -0.087176 | -1.082955 |
| C | -2.759382 | -0.645151 | 0.135747 |
| H | -5.561933 | -0.287726 | 1.998475 |
| H | -4.855549 | 0.817028 | -2.075442 |
| C | -3.156076 | -1.319414 | 2.581777 |
| H | -2.844978 | -2.362101 | 2.476302 |
| H | -2.312297 | -0.769681 | 3.006298 |
| H | -3.974250 | -1.286177 | 3.301928 |

| | | | |
|---|---|---|---|
| H | -2.902833 | 0.302415 | -3.174368 |
| H | -1.579824 | 0.860502 | -2.149968 |
| H | -1.742561 | -0.856511 | -2.497566 |
| C | -2.304561 | 0.054787 | -2.296764 |
| C | -6.794805 | 0.807030 | -0.169897 |
| H | -6.869149 | 1.651809 | -0.858169 |
| H | -7.440817 | 0.011549 | -0.556456 |
| H | -7.193175 | 1.112567 | 0.799701 |
| S | -1.098678 | -1.371070 | 0.338637 |
| O | -0.793612 | -2.271165 | -0.843751 |
| N | -0.221712 | 0.135190 | 0.223423 |
| C | 0.715737 | 0.159061 | -0.643266 |

| | | | | | | | |
|---|---|---|---|---|---|---|---|
| H | 0.888058 | -0.637510 | -1.367994 | H | 3.453623 | -1.244384 | -1.599877 |
| C | 1.572326 | 1.370057 | -0.842809 | C | 4.062590 | 0.129551 | 0.802426 |
| O | 2.233104 | 1.516211 | -1.852468 | H | 4.203844 | 0.809253 | -0.050082 |
| H | 3.350091 | 3.161301 | -0.155774 | H | 3.758308 | 0.733417 | 1.660170 |
| H | 1.946251 | 4.012691 | -0.821624 | C | 4.737303 | -2.506603 | -0.407681 |
| O | 1.516275 | 2.236140 | 0.164858 | C | 5.429278 | -0.559821 | 1.071685 |
| C | 2.315560 | 3.449767 | 0.038103 | C | 5.823486 | -1.465689 | -0.103086 |
| C | 2.170309 | 4.217483 | 1.333131 | H | 6.771861 | -1.967828 | 0.113597 |
| H | 2.754272 | 5.139149 | 1.275300 | H | 5.989282 | -0.846734 | -0.994301 |
| H | 1.126367 | 4.481356 | 1.515590 | H | 4.638205 | -3.191125 | 0.442783 |
| H | 2.537854 | 3.628183 | 2.176023 | H | 5.013627 | -3.111076 | -1.277430 |
| C | 3.037769 | -0.898669 | 0.457771 | H | 5.355753 | -1.157557 | 1.987568 |
| C | 3.366890 | -1.821577 | -0.667546 | H | 6.194098 | 0.203890 | 1.245248 |
| H | 2.585141 | -2.570235 | -0.816469 | H | 2.325669 | -1.211068 | 1.21420 |

***re-TS for cyclohexyl radical (axial) addition to N-sulfinyl imine 4 (TS2$_{sec}$-ax)***

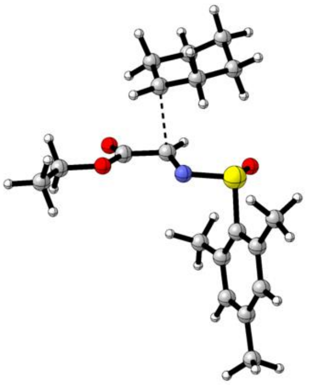

*Charge:* 0

*Multiplicity:* 2

*E* = -1419.78131868

*H* = -1419.317569

*G* = -1419.405182

*Cartesian coordinates:*

| | | | | | | | |
|---|---|---|---|---|---|---|---|
| C | -3.200540 | -1.023054 | 0.986626 | H | -6.863763 | 0.718409 | 0.760787 |
| C | -4.518739 | -0.564839 | 0.907338 | S | -0.620906 | -1.312072 | 0.094658 |
| C | -4.955144 | 0.249762 | -0.138153 | O | -0.259289 | -1.962979 | -1.232040 |
| C | -4.034388 | 0.619191 | -1.122141 | N | 0.185804 | 0.199266 | 0.293361 |
| C | -2.703138 | 0.199412 | -1.092896 | C | 1.230643 | 0.398208 | -0.461121 |
| C | -2.307218 | -0.627390 | -0.024572 | H | 1.471773 | -0.246935 | -1.305692 |
| H | -5.220008 | -0.856614 | 1.682382 | C | 1.826084 | 1.764808 | -0.554816 |
| H | -4.358648 | 1.258065 | -1.937758 | O | 2.495723 | 2.105760 | -1.511461 |
| C | -2.794583 | -1.907002 | 2.146799 | H | 3.178506 | 3.825044 | 0.382577 |
| H | -2.424280 | -2.877487 | 1.806143 | H | 1.712530 | 4.407765 | -0.423781 |
| H | -2.003124 | -1.449730 | 2.745817 | O | 1.543295 | 2.547274 | 0.486754 |
| H | -3.649365 | -2.086149 | 2.799928 | C | 2.091897 | 3.897512 | 0.463810 |
| H | -2.320541 | 1.068637 | -3.019214 | C | 1.658870 | 4.578216 | 1.743633 |
| H | -1.121808 | 1.478452 | -1.789960 | H | 2.052162 | 5.597511 | 1.763360 |
| H | -1.105893 | -0.123292 | -2.520151 | H | 0.569758 | 4.627582 | 1.809220 |
| C | -1.759120 | 0.675475 | -2.170655 | H | 2.040509 | 4.044330 | 2.616709 |
| C | -6.392844 | 0.696835 | -0.224052 | C | 3.155235 | -0.299289 | 0.729323 |
| H | -6.474069 | 1.690666 | -0.669969 | C | 4.252208 | -0.400873 | -0.282703 |
| H | -6.969670 | 0.008687 | -0.851182 | H | 4.291808 | 0.498762 | -0.902572 |

| | | | |
|---|---|---|---|
| H | 5.198248 | -0.408499 | 0.287263 |
| C | 2.701911 | -1.537789 | 1.433323 |
| H | 1.735012 | -1.369973 | 1.917835 |
| H | 3.415353 | -1.696049 | 2.260900 |
| C | 2.683472 | -2.794317 | 0.547677 |
| H | 2.537880 | -3.676959 | 1.176975 |
| H | 1.832400 | -2.749837 | -0.137960 |
| C | 4.183324 | -1.672434 | -1.142257 |
| H | 3.356456 | -1.585915 | -1.856880 |
| H | 5.099414 | -1.760913 | -1.732917 |
| C | 3.971501 | -2.920537 | -0.276053 |
| H | 4.830130 | -3.047316 | 0.396107 |
| H | 3.927283 | -3.814036 | -0.906021 |
| H | 3.121392 | 0.612281 | 1.319327 |

***re-TS for cyclohexyl radical (equatorial) addition to N-sulfinyl imine 4 (TS2$_{sec}$-eq)***

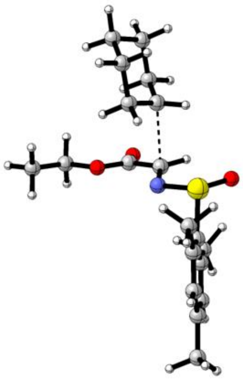

*Charge:* 0

*Multiplicity:* 2

*E* = -1419.78024846

*H* = -1419.316370

*G* = -1419.405507

*Cartesian coordinates:*

| | | | |
|---|---|---|---|
| C | -3.360671 | -1.187116 | 0.903746 |
| C | -4.645991 | -0.702034 | 1.162751 |
| C | -5.206437 | 0.336612 | 0.418889 |
| C | -4.449295 | 0.900270 | -0.611806 |
| C | -3.157536 | 0.464717 | -0.911956 |
| C | -2.630862 | -0.586456 | -0.137069 |
| H | -5.222957 | -1.155001 | 1.962368 |
| H | -4.875028 | 1.705870 | -1.201951 |
| C | -2.815987 | -2.320490 | 1.746917 |
| H | -2.566444 | -3.194154 | 1.138989 |
| H | -1.909131 | -2.026679 | 2.281389 |
| H | -3.556411 | -2.628309 | 2.486068 |
| H | -3.058350 | 1.760391 | -2.621875 |
| H | -1.619770 | 1.802455 | -1.599556 |
| H | -1.887546 | 0.428188 | -2.665090 |
| C | -2.388766 | 1.147128 | -2.017555 |
| C | -6.585803 | 0.859697 | 0.732006 |
| H | -7.117327 | 1.146785 | -0.178217 |
| H | -7.184003 | 0.117091 | 1.263552 |
| H | -6.521531 | 1.749859 | 1.366792 |
| S | -0.991368 | -1.309363 | -0.476309 |
| O | -0.886972 | -1.634317 | -1.957917 |
| N | -0.062417 | 0.090194 | -0.065703 |
| C | 0.832118 | 0.437670 | -0.935114 |
| H | 0.842164 | 0.065532 | -1.959228 |
| C | 1.679525 | 1.647223 | -0.714245 |
| O | 2.225736 | 2.222901 | -1.635572 |
| H | 3.595818 | 2.995391 | 0.462115 |
| H | 2.166044 | 4.037107 | 0.366422 |
| O | 1.770477 | 2.006838 | 0.565021 |
| C | 2.597307 | 3.168497 | 0.868300 |
| C | 2.612327 | 3.324124 | 2.373020 |
| H | 3.222617 | 4.189472 | 2.642448 |
| H | 1.602648 | 3.480537 | 2.758868 |
| H | 3.038562 | 2.438965 | 2.850245 |
| C | 2.720353 | -1.062890 | -0.541590 |
| C | 3.964807 | -0.532062 | -1.171621 |
| H | 3.880509 | -0.487031 | -2.259591 |
| H | 4.173196 | 0.482553 | -0.812033 |
| C | 2.726695 | -1.186393 | 0.945234 |
| H | 2.860233 | -0.197607 | 1.399045 |
| H | 1.784890 | -1.595736 | 1.316747 |
| C | 5.159032 | -1.437493 | -0.751146 |
| C | 3.921505 | -2.085522 | 1.368809 |
| C | 5.237317 | -1.563255 | 0.776200 |
| H | 6.062109 | -2.227189 | 1.053860 |
| H | 5.462150 | -0.579745 | 1.207868 |
| H | 5.031811 | -2.430755 | -1.196398 |
| H | 6.085313 | -1.018516 | -1.156188 |
| H | 3.743258 | -3.109431 | 1.020596 |
| H | 3.974054 | -2.121690 | 2.461343 |
| H | 2.160174 | -1.806638 | -1.10260 |

***(R)-configured adduct of 4 with cycloxehyl radical ($12_{sec}$)***

*Charge:* 0

*Multiplicity:* 2

*E* = -1419.81171359

*H* = -1419.344208

*G* = -1419.431395

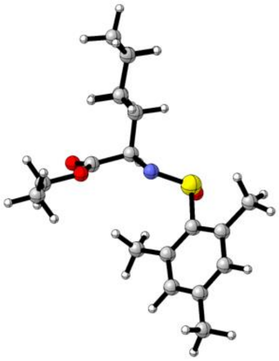

*Cartesian coordinates:*

| | | | |
|---|---|---|---|
| C | -3.243099 | -1.548452 | 0.633444 |
| C | -4.517740 | -1.034232 | 0.887018 |
| C | -4.938024 | 0.189429 | 0.362582 |
| C | -4.051657 | 0.913227 | -0.440323 |
| C | -2.763172 | 0.456580 | -0.722475 |
| C | -2.386436 | -0.779111 | -0.168838 |
| H | -5.198623 | -1.610526 | 1.504624 |
| H | -4.369876 | 1.861967 | -0.860599 |
| C | -2.842291 | -2.883702 | 1.218554 |
| H | -2.569792 | -3.600151 | 0.438617 |
| H | -1.983978 | -2.786846 | 1.888227 |
| H | -3.667793 | -3.310277 | 1.789070 |
| H | -2.401268 | 2.048239 | -2.120574 |
| H | -1.123645 | 1.824840 | -0.921646 |
| H | -1.271624 | 0.696734 | -2.275035 |
| C | -1.837199 | 1.300044 | -1.562430 |
| C | -6.308894 | 0.734508 | 0.674665 |
| H | -6.711132 | 1.303108 | -0.166646 |
| H | -7.010350 | -0.064920 | 0.921152 |
| H | -6.262719 | 1.411020 | 1.534706 |
| S | -0.758739 | -1.507608 | -0.557589 |
| O | -0.656504 | -1.760750 | -2.049785 |
| N | 0.206794 | -0.312258 | 0.030741 |
| C | 1.413276 | -0.030407 | -0.720020 |
| H | 1.322601 | -0.317573 | -1.773180 |
| C | 1.697722 | 1.473246 | -0.729337 |
| O | 2.350200 | 1.996761 | -1.606454 |
| H | 2.513559 | 3.713555 | 0.451150 |
| H | 1.068086 | 4.018710 | -0.527474 |
| O | 1.184792 | 2.121534 | 0.313483 |
| C | 1.434723 | 3.556963 | 0.391324 |
| C | 0.710992 | 4.067472 | 1.617667 |
| H | 0.872944 | 5.143822 | 1.713369 |
| H | -0.363021 | 3.885658 | 1.537880 |
| H | 1.084543 | 3.580458 | 2.521034 |
| C | 2.626383 | -0.825604 | -0.118398 |
| C | 3.864148 | -0.765526 | -1.026835 |
| H | 3.603412 | -1.095200 | -2.037712 |
| H | 4.207134 | 0.270942 | -1.109308 |
| C | 2.973464 | -0.402216 | 1.316512 |
| H | 3.291691 | 0.647647 | 1.317291 |
| H | 2.085795 | -0.464652 | 1.950579 |
| C | 4.996778 | -1.634380 | -0.458061 |
| C | 4.106943 | -1.268864 | 1.883791 |
| C | 5.346876 | -1.229502 | 0.980388 |
| H | 6.128511 | -1.884249 | 1.378735 |
| H | 5.757110 | -0.211331 | 0.977580 |
| H | 4.686231 | -2.686892 | -0.472032 |
| H | 5.878528 | -1.557326 | -1.101933 |
| H | 3.757172 | -2.305324 | 1.973594 |
| H | 4.359553 | -0.932998 | 2.894371 |
| H | 2.284559 | -1.868103 | -0.092928 |

***Reduced (R)-configured adduct of 4 with cycloxehyl radical ($5_{sec}^{-}$)***

*Charge:* –1

*Multiplicity:* 1

*E* = -1419.96650123

*H* = -1419.499996

*G* = -1419.586283

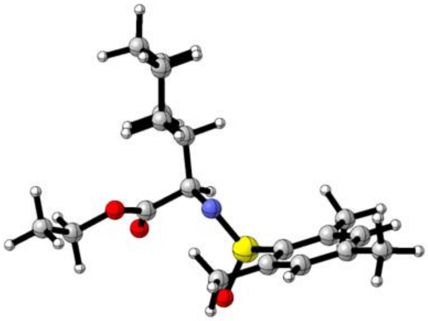

*Cartesian coordinates:*

| | | | |
|---|---|---|---|
| C | -3.327597 | -0.820168 | -0.955847 |
| C | -4.501723 | -1.201745 | -0.294840 |
| C | -4.910746 | -0.592845 | 0.890900 |
| C | -4.109220 | 0.424292 | 1.416854 |
| C | -2.927697 | 0.841673 | 0.797520 |
| C | -2.544096 | 0.206328 | -0.400442 |
| H | -5.108375 | -1.995004 | -0.721859 |
| H | -4.410854 | 0.909339 | 2.341238 |
| C | -2.941024 | -1.537336 | -2.232234 |
| H | -2.888657 | -0.851618 | -3.081982 |
| H | -1.958424 | -2.008803 | -2.144714 |
| H | -3.670092 | -2.315018 | -2.467717 |
| H | -2.540009 | 2.225517 | 2.399896 |
| H | -1.080612 | 1.618399 | 1.574998 |
| H | -2.073464 | 2.815735 | 0.782955 |
| C | -2.113151 | 1.941633 | 1.435591 |
| C | -6.163805 | -1.036846 | 1.606920 |
| H | -6.689400 | -0.188180 | 2.052246 |
| H | -6.851071 | -1.548748 | 0.929622 |
| H | -5.922547 | -1.731359 | 2.419100 |
| S | -1.043473 | 0.701933 | -1.327893 |
| O | -1.154571 | 2.254071 | -1.443136 |
| N | 0.069374 | 0.179589 | -0.248099 |
| C | 1.354202 | -0.089517 | -0.892950 |
| H | 1.243496 | -0.315381 | -1.967656 |
| C | 2.253884 | 1.151384 | -0.896869 |
| O | 2.672734 | 1.688796 | -1.907889 |
| H | 4.288684 | 2.647347 | -0.058833 |
| H | 2.816472 | 3.626776 | -0.122873 |
| O | 2.523629 | 1.621384 | 0.330807 |
| C | 3.324344 | 2.827572 | 0.422241 |
| C | 3.477939 | 3.153951 | 1.893231 |
| H | 4.076000 | 4.061493 | 2.008185 |
| H | 2.503452 | 3.322104 | 2.357556 |
| H | 3.980681 | 2.340377 | 2.421285 |
| C | 2.047089 | -1.328560 | -0.269210 |
| C | 3.419230 | -1.606306 | -0.909008 |
| H | 3.325649 | -1.641269 | -2.000261 |
| H | 4.101875 | -0.776951 | -0.678672 |
| C | 2.166874 | -1.310461 | 1.262832 |
| H | 2.805453 | -0.474849 | 1.566279 |
| H | 1.181578 | -1.133291 | 1.699938 |
| C | 4.033796 | -2.912970 | -0.386906 |
| C | 2.766575 | -2.624110 | 1.785803 |
| C | 4.131543 | -2.911893 | 1.144999 |
| H | 4.528937 | -3.868467 | 1.501078 |
| H | 4.844126 | -2.137143 | 1.457913 |
| H | 3.407033 | -3.755821 | -0.706472 |
| H | 5.022137 | -3.069302 | -0.832104 |
| H | 2.081762 | -3.452241 | 1.558591 |
| H | 2.860947 | -2.585825 | 2.876530 |
| H | 1.382316 | -2.164322 | -0.531051 |

***Sodium n-hexyloxalate ($3_{prim}$·$Na^+$)***

*Charge:* 0

*Multiplicity:* 1

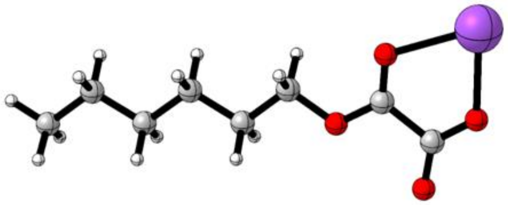

*E* = -776.234782022

*H* = -776.011915

*G* = -776.074672

*Cartesian coordinates:*

| | | | | | | | |
|---|---|---|---|---|---|---|---|
| O | 0.822524 | -3.999957 | 2.704431 | C | -2.364753 | -4.434860 | 0.717472 |
| O | 1.011024 | -2.025772 | 3.751212 | H | -2.300101 | -3.516137 | 0.121311 |
| C | 1.460318 | -3.123392 | 3.467305 | H | -3.082539 | -4.231914 | 1.521957 |
| C | 2.866048 | -3.627770 | 3.979231 | C | -2.897931 | -5.570501 | -0.161394 |
| O | 3.479129 | -2.768570 | 4.661174 | H | -2.961907 | -6.490104 | 0.434325 |
| O | 3.209426 | -4.778194 | 3.669191 | H | -2.178590 | -5.774293 | -0.964945 |
| Na | 2.535780 | -0.704078 | 5.088485 | C | -4.268888 | -5.270425 | -0.775635 |
| C | -0.482085 | -3.608650 | 2.183426 | H | -4.203946 | -4.349819 | -1.368313 |
| H | -0.362181 | -2.685920 | 1.610733 | H | -4.987026 | -5.068034 | 0.028286 |
| H | -1.143634 | -3.405144 | 3.028960 | C | -4.791219 | -6.410320 | -1.654958 |
| C | -0.992288 | -4.748212 | 1.323550 | H | -5.770102 | -6.172026 | -2.080614 |
| H | -0.268764 | -4.943155 | 0.524874 | H | -4.105591 | -6.611170 | -2.484577 |
| H | -1.052498 | -5.656371 | 1.932609 | H | -4.893374 | -7.335289 | -1.078179 |

***n-Hexyl oxoacyl radical – $CO_2$ – sodium complex ($10_{prim}$·$CO_2$·$Na^+$)***

*Charge:* 0

*Multiplicity:* 2

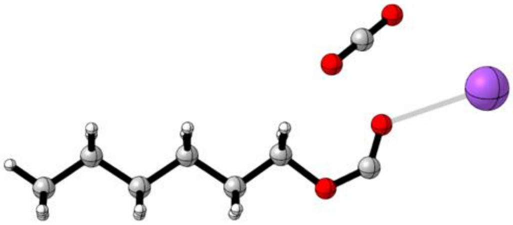

*E* = -776.028670875

*H* = -775.806094

*G* = -775.874700

*Cartesian coordinates:*

| | | | | | | | |
|---|---|---|---|---|---|---|---|
| O | 1.383253 | -3.186368 | 1.129348 | H | -0.503771 | -2.528418 | 1.758995 |
| O | 1.931170 | -2.180285 | 3.096321 | H | 0.032235 | -4.079432 | 2.458485 |
| C | 2.139327 | -2.575450 | 1.986182 | C | -0.686293 | -4.228753 | 0.408007 |
| C | 1.132030 | -3.777398 | 5.473588 | H | -0.664925 | -3.605570 | -0.491585 |
| O | 1.396534 | -2.895638 | 6.180754 | H | -0.112949 | -5.135976 | 0.192397 |
| O | 0.865684 | -4.666058 | 4.776402 | C | -2.135226 | -4.598506 | 0.754671 |
| Na | 4.149978 | -1.552572 | 5.056662 | H | -2.701056 | -3.687093 | 0.982615 |
| C | -0.027058 | -3.486377 | 1.545967 | H | -2.147923 | -5.208793 | 1.665819 |

| | | | |
|---|---|---|---|
| C | -2.835764 | -5.360699 | -0.374488 |
| H | -2.265796 | -6.269470 | -0.605885 |
| H | -2.825060 | -4.749408 | -1.285773 |
| C | -4.280840 | -5.742292 | -0.037897 |
| H | -4.848497 | -4.833103 | 0.194429 |
| H | -4.288885 | -6.351121 | 0.874321 |
| C | -4.975109 | -6.505376 | -1.169681 |
| H | -6.003433 | -6.766535 | -0.904287 |
| H | -5.007761 | -5.905468 | -2.084899 |
| H | -4.443027 | -7.433919 | -1.400549 |

***n-Hexyl oxyacyl radical ($10_{prim}$)***

*Charge:* 0

*Multiplicity:* 2

*E* = -425.121330071

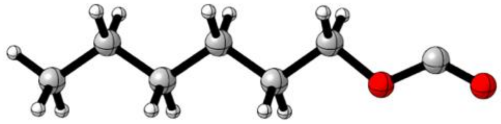

*H* = -424.918536

*G* = -424.970868

*Cartesian coordinates:*

| | | | |
|---|---|---|---|
| C | -0.285774 | -0.321248 | -0.035113 |
| C | -1.518103 | 0.558122 | -0.048817 |
| H | -0.323092 | -0.973691 | 0.843331 |
| H | -0.296491 | -0.965808 | -0.920109 |
| O | -2.692383 | -0.335440 | -0.070479 |
| O | -4.939053 | -0.314001 | 0.033754 |
| C | -3.877060 | 0.223162 | 0.036579 |
| H | -1.562135 | 1.186837 | -0.939622 |
| H | -1.584722 | 1.182565 | 0.843350 |
| C | 1.000779 | 0.512885 | -0.012003 |
| H | 0.999044 | 1.163753 | 0.870839 |
| H | 1.023738 | 1.174915 | -0.886191 |
| C | 2.264581 | -0.352678 | 0.000273 |
| H | 2.265635 | -1.004588 | -0.882568 |
| H | 2.240162 | -1.016886 | 0.873581 |
| C | 3.558659 | 0.466859 | 0.024865 |
| H | 3.555252 | 1.118463 | 0.907078 |
| H | 3.580709 | 1.131060 | -0.847617 |
| C | 4.816983 | -0.405953 | 0.036900 |
| H | 4.859339 | -1.045157 | -0.850941 |
| H | 5.725923 | 0.202006 | 0.054741 |
| H | 4.833364 | -1.058187 | 0.916084 |

***TS for $CO_2$ elimination from n-hexyl oxyacyl radical ($TS1_{prim}$)***

*Charge:* 0

*Multiplicity:* 2

*E* = -425.104396975

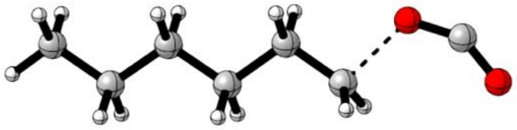

*H* = -424.905031

*G* = -424.957884

*Cartesian coordinates:*

| | | | |
|---|---|---|---|
| C | -0.673518 | 0.198141 | 0.447314 |
| O | 0.903059 | 0.649729 | -0.547725 |
| O | 2.425295 | -0.935310 | 0.166143 |
| C | 1.913603 | -0.018859 | -0.391703 |
| C | -1.645830 | 1.223776 | -0.019700 |
| H | -1.262187 | 2.225239 | 0.198581 |

| | | | |
|---|---|---|---|
| H | -1.774969 | 1.146131 | -1.103698 |
| H | -0.275801 | 0.293682 | 1.452256 |
| H | -0.806369 | -0.822802 | 0.104906 |
| C | -3.019567 | 1.047165 | 0.667561 |
| H | -3.404483 | 0.042639 | 0.458327 |
| H | -2.894277 | 1.116354 | 1.754081 |
| C | -4.038458 | 2.093260 | 0.201914 |
| H | -4.156152 | 2.024449 | -0.886871 |
| H | -3.646395 | 3.097228 | 0.407761 |
| C | -5.408051 | 1.937089 | 0.870595 |
| H | -5.796603 | 0.932321 | 0.665274 |
| H | -5.287381 | 2.003981 | 1.958552 |
| C | -6.421908 | 2.984901 | 0.401538 |
| H | -6.582846 | 2.918522 | -0.679326 |
| H | -7.390140 | 2.852415 | 0.892557 |
| H | -6.070060 | 3.997684 | 0.623005 |

***n*-Hexyl radical — $CO_2$ complex ($11_{prim}\cdot CO_2$)**

*Charge:* 0

*Multiplicity:* 2

*E* = -425.147232466

*H* = -424.947708

*G* = -425.007897

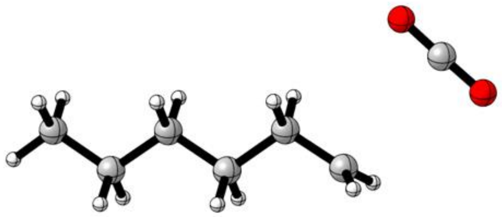

*Cartesian coordinates:*

| | | | |
|---|---|---|---|
| C | -1.623656 | -0.213090 | 0.816775 |
| O | 1.070177 | 0.723659 | -1.019164 |
| O | 1.616191 | -1.116233 | 0.287812 |
| C | 1.331698 | -0.196400 | -0.361251 |
| C | -2.189736 | 0.948396 | 0.076733 |
| H | -1.608365 | 1.852418 | 0.293562 |
| H | -2.120523 | 0.776137 | -1.003913 |
| H | -1.220152 | -0.091693 | 1.815891 |
| H | -1.759022 | -1.224555 | 0.450160 |
| C | -3.673330 | 1.233544 | 0.425827 |
| H | -4.270410 | 0.339902 | 0.209404 |
| H | -3.759460 | 1.413599 | 1.503834 |
| C | -4.241544 | 2.430557 | -0.342773 |
| H | -4.146042 | 2.247334 | -1.420908 |
| H | -3.635511 | 3.320185 | -0.127475 |
| C | -5.707503 | 2.725151 | -0.008499 |
| H | -6.311597 | 1.835260 | -0.223329 |
| H | -5.801508 | 2.907167 | 1.069008 |
| C | -6.267267 | 3.923315 | -0.781356 |
| H | -6.212737 | 3.753107 | -1.861539 |
| H | -7.313691 | 4.112262 | -0.525067 |
| H | -5.699042 | 4.832634 | -0.560003 |

***n*-Hexyl radical ($11_{prim}$)**

*Charge:* 0

*Multiplicity:* 2

*E* = -236.494728406

*H* = -236.311832

*G* = -236.355504

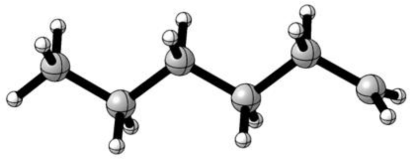

*Cartesian coordinates:*

| | | | | | | | |
|---|---|---|---|---|---|---|---|
| C | 2.254252 | -1.492371 | -0.001601 | H | 3.885939 | -3.573485 | -0.882257 |
| C | 1.764772 | -0.085519 | -0.000026 | H | 3.886064 | -3.575408 | 0.874263 |
| H | 1.876692 | -2.025297 | 0.879557 | C | 5.818602 | -3.180123 | -0.003703 |
| H | 1.876566 | -2.023366 | -0.883871 | H | 6.222376 | -2.659339 | -0.880652 |
| H | 1.665010 | 0.468151 | -0.927025 | H | 6.222501 | -2.661260 | 0.874327 |
| H | 1.665154 | 0.466123 | 0.928198 | C | 6.299675 | -4.634418 | -0.005329 |
| C | 3.801693 | -1.603837 | -0.001834 | H | 5.934464 | -5.167004 | -0.889672 |
| H | 4.197345 | -1.080380 | -0.880305 | H | 7.391975 | -4.695737 | -0.005474 |
| H | 4.197470 | -1.082304 | 0.877726 | H | 5.934590 | -5.168938 | 0.877898 |
| C | 4.291450 | -3.055140 | -0.003457 | | | | |

***n-Hexyl radical — N-sulfinyl imine 4 re-precomplex ($11_{prim}$·4)***

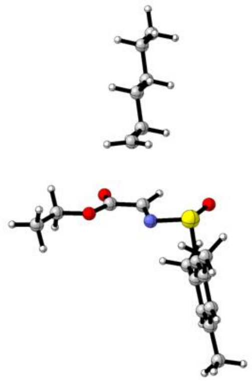

*Charge:* 0

*Multiplicity:* 2

*E* = -1420.98086882

*H* = -1420.495624

*G* = -1420.596941

*Cartesian coordinates:*

| | | | | | | | |
|---|---|---|---|---|---|---|---|
| C | -3.950272 | -0.973997 | 1.185307 | N | -0.687315 | 0.449098 | 0.436954 |
| C | -5.281810 | -0.824135 | 0.789142 | C | 0.353745 | 0.530147 | -0.291432 |
| C | -5.637222 | -0.625568 | -0.545495 | H | 0.812488 | -0.325037 | -0.790551 |
| C | -4.620920 | -0.565178 | -1.502184 | C | 0.977410 | 1.851837 | -0.633506 |
| C | -3.271893 | -0.698681 | -1.167975 | O | 1.771931 | 1.951086 | -1.546469 |
| C | -2.958771 | -0.905519 | 0.189408 | H | 2.216804 | 4.097398 | -0.042595 |
| H | -6.058762 | -0.868502 | 1.545280 | H | 0.891002 | 4.438728 | -1.168163 |
| H | -4.882608 | -0.404390 | -2.543352 | O | 0.563050 | 2.853010 | 0.134724 |
| C | -3.636838 | -1.189037 | 2.650722 | C | 1.131380 | 4.169738 | -0.137753 |
| H | -3.109628 | -2.131959 | 2.818187 | C | 0.530995 | 5.132658 | 0.862092 |
| H | -3.009199 | -0.389503 | 3.052232 | H | 0.934968 | 6.132827 | 0.687891 |
| H | -4.559237 | -1.214570 | 3.231736 | H | -0.555166 | 5.176479 | 0.757049 |
| H | -2.704664 | -0.655496 | -3.237733 | H | 0.775197 | 4.835272 | 1.884156 |
| H | -1.726800 | 0.385069 | -2.203036 | C | 2.688846 | 0.192708 | 1.317533 |
| H | -1.469699 | -1.355836 | -2.174689 | C | 3.552548 | -0.431613 | 0.281060 |
| C | -2.232838 | -0.583044 | -2.257200 | H | 3.122826 | -1.385406 | -0.044606 |
| C | -7.084321 | -0.509126 | -0.952704 | H | 3.601331 | 0.219716 | -0.600344 |
| H | -7.205293 | 0.169982 | -1.799583 | H | 2.807269 | 1.241272 | 1.568714 |
| H | -7.474087 | -1.485990 | -1.258195 | H | 2.088769 | -0.412998 | 1.986371 |
| H | -7.703366 | -0.150401 | -0.128025 | C | 5.001194 | -0.686046 | 0.771404 |
| S | -1.249706 | -1.177410 | 0.763328 | H | 5.439596 | 0.263734 | 1.099182 |
| O | -0.562652 | -2.178951 | -0.143656 | H | 4.974011 | -1.339452 | 1.650922 |

| | | | |
|---|---|---|---|
| C | 5.883967 | -1.315049 | -0.310881 |
| H | 5.433693 | -2.259763 | -0.641932 |
| H | 5.902417 | -0.658133 | -1.190058 |
| C | 7.319694 | -1.575908 | 0.156101 |
| H | 7.298926 | -2.231353 | 1.035253 |
| H | 7.767817 | -0.631310 | 0.487566 |
| C | 8.195616 | -2.205042 | -0.931628 |
| H | 9.214052 | -2.381300 | -0.573710 |
| H | 7.784765 | -3.165758 | -1.258678 |
| H | 8.257388 | -1.554473 | -1.810073 |

***re-TS for n-hexyl radical addition to N-sulfinyl imine 4 (TS2$_{prim}$)***

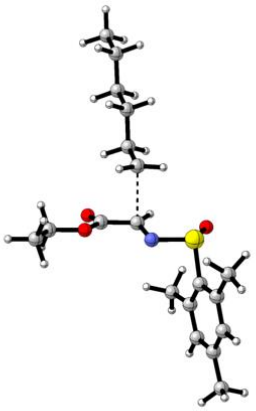

*Charge:* 0

*Multiplicity:* 2

*E* = -1420.98010710

*H* = -1420.495207

*G* = -1420.592202

*Cartesian coordinates:*

| | | | |
|---|---|---|---|
| C | -3.002715 | -0.854302 | 0.104340 |
| C | -3.919806 | -1.072546 | 1.147919 |
| C | -5.264896 | -0.781039 | 0.904194 |
| C | -5.704263 | -0.300702 | -0.329889 |
| C | -4.759263 | -0.097785 | -1.339050 |
| C | -3.401089 | -0.361619 | -1.153137 |
| C | -3.512703 | -1.592044 | 2.510384 |
| C | -7.166846 | -0.033864 | -0.582106 |
| C | -2.436855 | -0.077478 | -2.279485 |
| S | -1.275845 | -1.324550 | 0.455574 |
| N | -0.610202 | 0.273168 | 0.359132 |
| C | 0.428630 | 0.395447 | -0.400736 |
| C | 1.064304 | 1.734894 | -0.614253 |
| O | 0.792577 | 2.613915 | 0.346739 |
| C | 1.382575 | 3.941148 | 0.211579 |
| C | 0.956607 | 4.745212 | 1.419989 |
| O | -0.759856 | -2.207988 | -0.667122 |
| O | 1.754329 | 1.960539 | -1.588537 |
| C | 2.340806 | -0.335905 | 0.879730 |
| C | 3.469678 | -0.488718 | -0.076015 |
| H | 1.805752 | -1.218935 | 1.212200 |
| H | -5.985016 | -0.938519 | 1.700480 |
| H | -5.085902 | 0.282857 | -2.301763 |
| H | -3.034874 | -2.573015 | 2.444195 |
| H | -2.807497 | -0.919379 | 3.005125 |
| H | -4.388933 | -1.690280 | 3.152064 |
| H | -2.979342 | 0.067506 | -3.214489 |
| H | -1.871705 | 0.836372 | -2.076425 |
| H | -1.717178 | -0.884754 | -2.411115 |
| H | -7.305483 | 0.816819 | -1.253180 |
| H | -7.637194 | -0.902860 | -1.054468 |
| H | -7.702560 | 0.166390 | 0.347897 |
| H | 0.727377 | -0.363335 | -1.124586 |
| H | 2.467031 | 3.828320 | 0.151718 |
| H | 1.028192 | 4.380074 | -0.723164 |
| H | 1.380591 | 5.750100 | 1.354058 |
| H | -0.131092 | 4.832265 | 1.466305 |
| H | 1.312718 | 4.280438 | 2.341949 |
| H | 3.216665 | -1.226051 | -0.845489 |
| H | 3.666010 | 0.460755 | -0.584587 |
| H | 2.325967 | 0.510417 | 1.558434 |
| C | 4.770207 | -0.942865 | 0.635897 |
| H | 4.590058 | -1.895855 | 1.145757 |
| H | 5.028159 | -0.213534 | 1.412067 |
| C | 5.942182 | -1.093127 | -0.339669 |
| H | 5.675067 | -1.819108 | -1.118011 |
| H | 6.110490 | -0.137906 | -0.852990 |
| C | 7.240141 | -1.536849 | 0.343098 |
| H | 7.069261 | -2.490740 | 0.856523 |
| H | 7.503549 | -0.811134 | 1.122003 |
| C | 8.407918 | -1.683806 | -0.637092 |
| H | 9.321488 | -2.001241 | -0.126384 |
| H | 8.180737 | -2.426110 | -1.409058 |
| H | 8.618533 | -0.734972 | -1.141024 |

***(R)-configured adduct of 4 with n-hexyl radical (12$_{prim}$)***

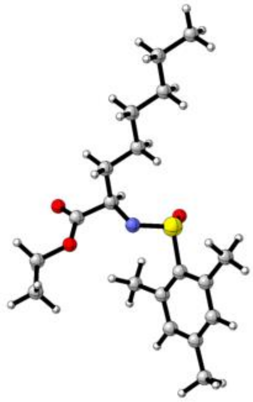

*Charge:* 0

*Multiplicity:* 2

*E* = -1421.01932445

*H* = -1420.530232

*G* = -1420.624796

*Cartesian coordinates:*

| | | | |
|---|---|---|---|
| C | 2.263646 | -2.463363 | -0.501033 |
| C | 3.610467 | -2.728297 | -0.764611 |
| C | 4.630278 | -1.889509 | -0.311354 |
| C | 4.284051 | -0.749266 | 0.419525 |
| C | 2.956921 | -0.426258 | 0.707471 |
| C | 1.967975 | -1.304286 | 0.232408 |
| H | 3.865080 | -3.615051 | -1.335585 |
| H | 5.067878 | -0.087369 | 0.773817 |
| C | 1.198786 | -3.408815 | -1.008933 |
| H | 0.600449 | -3.818013 | -0.190134 |
| H | 0.512635 | -2.908785 | -1.697186 |
| H | 1.656241 | -4.245338 | -1.537962 |
| H | 3.530183 | 1.219320 | 1.965269 |
| H | 2.284819 | 1.607961 | 0.774368 |
| H | 1.865082 | 0.677175 | 2.216313 |
| C | 2.639499 | 0.838550 | 1.464213 |
| C | 6.077575 | -2.220263 | -0.575467 |
| H | 6.673812 | -1.315047 | -0.709776 |
| H | 6.503103 | -2.769940 | 0.270832 |
| H | 6.188668 | -2.844772 | -1.463996 |
| S | 0.208897 | -1.017695 | 0.630963 |
| O | 0.020807 | -1.007466 | 2.134814 |
| N | -0.011267 | 0.420873 | -0.131655 |
| C | -0.854868 | 1.398351 | 0.526286 |
| H | -0.935595 | 1.222257 | 1.605276 |
| C | -0.276149 | 2.810117 | 0.383187 |
| O | -0.658814 | 3.724571 | 1.080095 |
| H | 0.435384 | 4.930667 | -1.072701 |
| H | 1.648267 | 4.581595 | 0.171854 |
| O | 0.640560 | 2.923132 | -0.573157 |
| C | 1.230539 | 4.243239 | -0.778159 |
| C | 2.288994 | 4.093922 | -1.848545 |
| H | 2.757586 | 5.063615 | -2.032898 |
| H | 3.063201 | 3.390855 | -1.533489 |
| H | 1.850455 | 3.739205 | -2.783738 |
| C | -2.287546 | 1.402717 | -0.078275 |
| C | -3.055021 | 0.105846 | 0.180727 |
| H | -2.538762 | -0.734320 | -0.296495 |
| H | -3.064473 | -0.101175 | 1.257637 |
| H | -2.211463 | 1.592534 | -1.153500 |
| H | -2.819863 | 2.245663 | 0.372092 |
| C | -4.494372 | 0.164708 | -0.340738 |
| H | -5.020913 | 0.998731 | 0.139910 |
| H | -4.481201 | 0.386049 | -1.415420 |
| C | -5.271968 | -1.132662 | -0.099842 |
| H | -4.744236 | -1.965974 | -0.581659 |
| H | -5.280513 | -1.355651 | 0.974989 |
| C | -6.713595 | -1.082976 | -0.615990 |
| H | -7.239950 | -0.250352 | -0.133553 |
| H | -6.703715 | -0.858766 | -1.689637 |
| C | -7.481329 | -2.385358 | -0.371304 |
| H | -8.506197 | -2.323887 | -0.748358 |
| H | -6.991654 | -3.228517 | -0.869355 |
| H | -7.531497 | -2.615921 | 0.697850 |

***Reduced (R)-configured adduct of 4 with n-hexyl radical ($5_{prim}^{-}$)***

*Charge:* –1

*Multiplicity:* 1

*E* = -1421.17690774

*H* = -1420.688804

*G* = -1420.781411

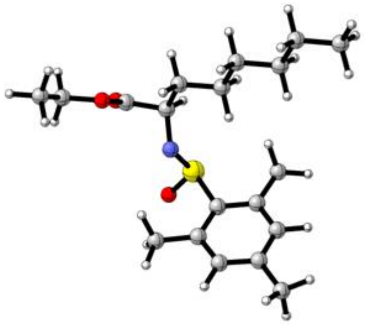

*Cartesian coordinates:*

| | | | |
|---|---|---|---|
| C | -2.334696 | -1.423736 | 0.849424 |
| C | -3.506160 | -1.780473 | 0.169967 |
| C | -3.490720 | -2.649123 | -0.920571 |
| C | -2.257358 | -3.159663 | -1.335356 |
| C | -1.059196 | -2.834029 | -0.693489 |
| C | -1.110760 | -1.958289 | 0.409322 |
| H | -4.452095 | -1.364457 | 0.504414 |
| H | -2.223770 | -3.828770 | -2.190895 |
| C | -2.432338 | -0.469349 | 2.021318 |
| H | -2.078530 | -0.930706 | 2.947210 |
| H | -1.828559 | 0.426977 | 1.861522 |
| H | -3.467838 | -0.158202 | 2.172266 |
| H | 0.054019 | -4.016499 | -2.106595 |
| H | 0.936683 | -2.612169 | -1.459437 |
| H | 0.720469 | -4.020789 | -0.451503 |
| C | 0.234832 | -3.411365 | -1.215711 |
| C | -4.767556 | -3.049038 | -1.620246 |
| H | -4.616346 | -3.147647 | -2.698311 |
| H | -5.126301 | -4.016876 | -1.253073 |
| H | -5.561652 | -2.318344 | -1.450671 |
| S | 0.388916 | -1.462643 | 1.341476 |
| O | 1.111536 | -2.810025 | 1.642779 |
| N | 1.110014 | -0.557911 | 0.186004 |
| C | 1.853325 | 0.574159 | 0.753650 |
| H | 1.679854 | 0.691647 | 1.835892 |
| C | 3.338084 | 0.252698 | 0.634390 |
| O | 4.024227 | -0.194141 | 1.536796 |
| H | 5.845226 | 0.615001 | -0.183116 |
| H | 5.294293 | -1.001644 | -0.644836 |
| O | 3.816349 | 0.456411 | -0.605626 |
| C | 5.190114 | 0.064220 | -0.862019 |
| C | 5.486892 | 0.377986 | -2.313487 |
| H | 6.516660 | 0.095753 | -2.546912 |
| H | 4.818941 | -0.177193 | -2.976170 |
| H | 5.369198 | 1.445761 | -2.512637 |
| C | 1.468490 | 1.893293 | 0.064526 |
| C | 0.022602 | 2.291819 | 0.362656 |
| H | -0.639033 | 1.485198 | 0.031804 |
| H | -0.112329 | 2.374940 | 1.449649 |
| H | 1.612734 | 1.783372 | -1.014959 |
| H | 2.147976 | 2.689119 | 0.397543 |
| C | -0.400563 | 3.606220 | -0.297753 |
| H | 0.265375 | 4.414374 | 0.032388 |
| H | -0.268119 | 3.525436 | -1.384688 |
| C | -1.851398 | 3.991266 | 0.007547 |
| H | -2.517145 | 3.184194 | -0.325700 |
| H | -1.983918 | 4.065836 | 1.095070 |
| C | -2.288853 | 5.307182 | -0.643424 |
| H | -1.621793 | 6.112039 | -0.310782 |
| H | -2.159371 | 5.231568 | -1.730099 |
| C | -3.739795 | 5.680050 | -0.324756 |
| H | -4.027016 | 6.622253 | -0.800495 |
| H | -4.429456 | 4.904839 | -0.674486 |
| H | -3.887482 | 5.791707 | 0.754446 |

***2-Allyladamantan-2-yl oxyacyl radical (10')***

*Charge:* 0

*Multiplicity:* 2

*E* = -695.557897876

*H* = -695.238011

*G* = -695.292585

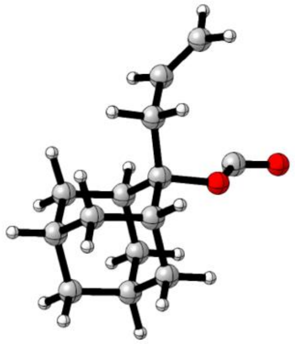

*Cartesian coordinates:*

| | | | |
|---|---|---|---|
| C | -1.949149 | -0.320647 | -0.644725 |
| C | -2.658261 | -0.615717 | -1.980639 |
| C | -1.606094 | -0.683069 | -3.102825 |
| C | -0.848440 | 0.659837 | -3.197354 |
| C | -1.199041 | 1.026670 | -0.735223 |
| C | -3.666354 | 0.507815 | -2.291883 |
| C | -1.866815 | 1.783230 | -3.503416 |
| C | -2.923143 | 1.855199 | -2.382568 |
| C | -2.225666 | 2.141969 | -1.038663 |
| C | 3.145294 | 2.273782 | -2.628444 |
| C | 1.841457 | 2.241498 | -2.904522 |
| C | 1.585060 | -0.666431 | -2.338472 |
| O | 0.755271 | -0.154754 | -1.457855 |
| C | -0.141320 | 0.976677 | -1.860528 |
| C | 0.756172 | 2.231502 | -1.865706 |
| O | 2.384249 | -1.534537 | -2.153098 |
| H | -4.181233 | 0.298949 | -3.236407 |
| H | -4.430760 | 0.554681 | -1.508239 |
| H | -0.913252 | -1.509354 | -2.922054 |
| H | -2.089558 | -0.880154 | -4.065159 |
| H | -3.181773 | -1.574551 | -1.912821 |
| H | -1.254860 | -1.127247 | -0.397493 |
| H | -2.681487 | -0.262393 | 0.167203 |
| H | -0.687751 | 1.233785 | 0.209873 |
| H | -3.634070 | 2.657223 | -2.603849 |
| H | -1.751065 | 3.126498 | -1.054245 |
| H | -2.963161 | 2.164535 | -0.230056 |
| H | -1.365075 | 2.747840 | -3.620676 |
| H | -2.349578 | 1.562360 | -4.460639 |
| H | 1.193379 | 2.324065 | -0.867312 |
| H | 1.521812 | 2.246337 | -3.943620 |
| H | 0.120445 | 3.106311 | -2.015188 |
| H | 3.891435 | 2.298410 | -3.414946 |
| H | 3.508158 | 2.272414 | -1.604685 |
| H | -0.105982 | 0.604384 | -3.997019 |

***TS for $CO_2$ elimination from 2-allyladamantan-2-yl oxyacyl radical (TS3)***

*Charge:* 0

*Multiplicity:* 2

*E* = -695.548151204

*H* = -695.230749

*G* = -695.285962

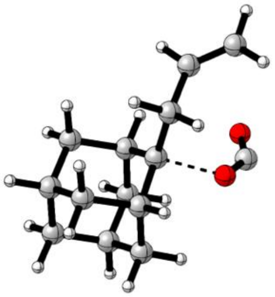

*Cartesian coordinates:*

| | | | |
|---|---|---|---|
| C | 1.684189 | 0.661460 | 1.380828 |
| C | 2.230952 | 1.058304 | -0.003045 |
| C | 1.048857 | 1.295008 | -0.958961 |
| C | 0.195227 | 0.013374 | -1.074835 |
| C | 0.842549 | -0.627955 | 1.262550 |
| C | 3.118846 | -0.072749 | -0.557218 |

| | | | |
|---|---|---|---|
| C | 1.101892 | -1.132084 | -1.633205 |
| C | 2.289061 | -1.366255 | -0.678844 |
| C | 1.759426 | -1.764771 | 0.711354 |
| C | -3.810202 | -1.138199 | -0.155898 |
| C | -2.558183 | -1.317296 | -0.570166 |
| C | -1.944043 | 1.823502 | 0.485579 |
| O | -1.272376 | 0.996049 | 1.104482 |
| C | -0.289853 | -0.464836 | 0.273710 |
| C | -1.385428 | -1.504617 | 0.351141 |
| O | -2.254143 | 2.193409 | -0.609714 |
| H | 3.518178 | 0.209815 | -1.537546 |
| H | 3.974193 | -0.239785 | 0.106607 |
| H | 0.435986 | 2.128549 | -0.610470 |
| H | 1.414433 | 1.561089 | -1.956140 |
| H | 2.817332 | 1.978103 | 0.086517 |

| | | | |
|---|---|---|---|
| H | 1.074122 | 1.466939 | 1.794680 |
| H | 2.509434 | 0.481150 | 2.077369 |
| H | 0.443545 | -0.916433 | 2.238613 |
| H | 2.915265 | -2.172652 | -1.073268 |
| H | 1.215635 | -2.711969 | 0.660649 |
| H | 2.588513 | -1.909161 | 1.411245 |
| H | 0.523506 | -2.051596 | -1.762021 |
| H | 1.457731 | -0.833827 | -2.624285 |
| H | -1.721996 | -1.570276 | 1.389125 |
| H | -2.350591 | -1.349466 | -1.636734 |
| H | -0.917441 | -2.473091 | 0.121582 |
| H | -4.628439 | -1.020412 | -0.857733 |
| H | -4.058366 | -1.100333 | 0.900934 |
| H | -0.642615 | 0.181873 | -1.751694 |

***2-Allyladamantan-2-yl radical — $CO_2$ complex (11·$CO_2$)***

*Charge:* 0

*Multiplicity:* 2

*E* = -695.588167042

*H* = -695.269738

*G* = -695.330835

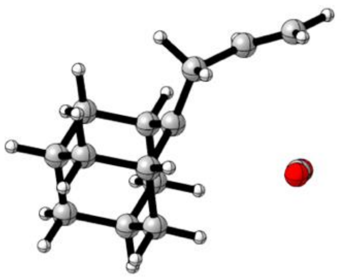

*Cartesian coordinates:*

| | | | |
|---|---|---|---|
| C | 1.418292 | 0.896817 | 1.141583 |
| C | 2.085138 | 1.163643 | -0.221462 |
| C | 1.032999 | 1.035728 | -1.340406 |
| C | 0.425081 | -0.386224 | -1.323679 |
| C | 0.803609 | -0.522608 | 1.153494 |
| C | 3.207330 | 0.130745 | -0.450096 |
| C | 1.565755 | -1.422532 | -1.558100 |
| C | 2.614515 | -1.293194 | -0.435083 |
| C | 1.947532 | -1.558424 | 0.930127 |
| C | -3.773498 | -1.138164 | 0.046055 |
| C | -2.581919 | -1.231827 | -0.542839 |
| C | -2.159557 | 1.658550 | 0.974077 |
| O | -2.137071 | 1.178835 | 2.031100 |
| C | -0.183476 | -0.675692 | 0.025564 |
| C | -1.311767 | -1.664868 | 0.147036 |
| O | -2.190874 | 2.170662 | -0.067527 |
| H | 3.700945 | 0.319923 | -1.410294 |
| H | 3.970063 | 0.226790 | 0.331244 |

| | | | |
|---|---|---|---|
| H | 0.240949 | 1.777941 | -1.202418 |
| H | 1.497000 | 1.226243 | -2.315053 |
| H | 2.507755 | 2.174056 | -0.230497 |
| H | 0.642897 | 1.644612 | 1.336094 |
| H | 2.157949 | 0.983767 | 1.945873 |
| H | 0.318731 | -0.711844 | 2.116239 |
| H | 3.413101 | -2.025157 | -0.597555 |
| H | 1.541411 | -2.575176 | 0.963071 |
| H | 2.684773 | -1.474658 | 1.737405 |
| H | 1.151876 | -2.436569 | -1.575435 |
| H | 2.030104 | -1.244169 | -2.535126 |
| H | -1.520152 | -1.867131 | 1.202278 |
| H | -2.492535 | -0.976787 | -1.596544 |
| H | -0.996839 | -2.628547 | -0.295772 |
| H | -4.653637 | -0.817729 | -0.501242 |
| H | -3.908041 | -1.377177 | 1.097209 |
| H | -0.317012 | -0.480240 | -2.121869 |

***TS for intramolecular cyclization of 2-allyladamantan-2-yl oxyacyl radical (TS4)***

*Charge:* 0

*Multiplicity:* 2

*E* = -695.553022764

*H* = -695.234797

*G* = -695.286598

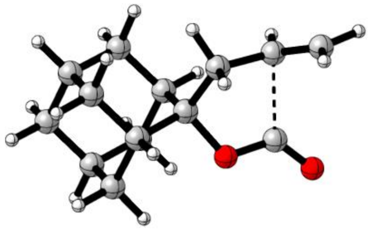

*Cartesian coordinates:*

| | | | |
|---|---|---|---|
| C | -1.976436 | -0.338181 | -0.661594 |
| C | -2.731093 | -0.599099 | -1.979271 |
| C | -1.714351 | -0.678334 | -3.134765 |
| C | -0.935299 | 0.651141 | -3.240305 |
| C | -1.191886 | 0.987849 | -0.761848 |
| C | -3.720542 | 0.553767 | -2.239791 |
| C | -1.939054 | 1.795779 | -3.505755 |
| C | -2.947140 | 1.883317 | -2.343129 |
| C | -2.187988 | 2.140225 | -1.025659 |
| C | 3.235790 | 2.278846 | -2.446118 |
| C | 1.981879 | 1.912433 | -2.827351 |
| C | 1.810030 | -0.294696 | -2.321018 |
| O | 0.690248 | -0.249593 | -1.623358 |
| C | -0.178641 | 0.924697 | -1.919716 |
| C | 0.768972 | 2.135943 | -1.957716 |
| O | 2.629331 | -1.164194 | -2.296643 |
| H | -4.275151 | 0.371276 | -3.167178 |
| H | -4.454261 | 0.609053 | -1.427726 |
| H | -1.025656 | -1.511977 | -2.975483 |
| H | -2.231580 | -0.861414 | -4.082300 |
| H | -3.276306 | -1.545570 | -1.907453 |
| H | -1.296488 | -1.165086 | -0.442921 |
| H | -2.683614 | -0.268764 | 0.171731 |
| H | -0.643585 | 1.168661 | 0.167917 |
| H | -3.645570 | 2.705520 | -2.527342 |
| H | -1.673187 | 3.104697 | -1.062406 |
| H | -2.892788 | 2.191730 | -0.189681 |
| H | -1.423617 | 2.750908 | -3.638794 |
| H | -2.464008 | 1.590263 | -4.444201 |
| H | 1.088745 | 2.355591 | -0.934754 |
| H | 1.798365 | 1.737700 | -3.883573 |
| H | 0.225056 | 3.010492 | -2.317593 |
| H | 4.069764 | 2.233371 | -3.136481 |
| H | 3.451913 | 2.561484 | -1.420896 |
| H | -0.213004 | 0.589285 | -4.060081 |

***Intramolecular cyclization product from 2-allyladamantan-2-yl oxyacyl radical (11')***

*Charge:* 0

*Multiplicity:* 2

*E* = -695.590987928

*H* = -695.271356

*G* = -695.323992

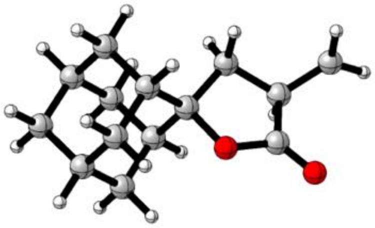

*Cartesian coordinates:*

| | | | |
|---|---|---|---|
| C | -1.946908 | -0.363265 | -0.695332 |
| C | -2.759441 | -0.602163 | -1.982486 |
| C | -1.794405 | -0.662323 | -3.183768 |
| C | -1.023988 | 0.670183 | -3.302608 |
| C | -1.169446 | 0.964117 | -0.808781 |
| C | -3.758924 | 0.555200 | -2.176385 |

| | | | |
|---|---|---|---|
| C | -2.038340 | 1.817974 | -3.503956 |
| C | -2.991205 | 1.886692 | -2.295104 |
| C | -2.166555 | 2.126137 | -1.013998 |
| C | 3.311848 | 2.193093 | -2.290252 |
| C | 2.050840 | 1.508019 | -2.670575 |
| C | 1.957885 | 0.049773 | -2.185700 |
| O | 0.701643 | -0.229490 | -1.809893 |
| C | -0.213152 | 0.921572 | -2.010910 |
| C | 0.760465 | 2.111344 | -2.094392 |
| O | 2.840733 | -0.773922 | -2.135472 |
| H | -4.357625 | 0.388453 | -3.079046 |
| H | -4.452995 | 0.596386 | -1.329227 |
| H | -1.094765 | -1.493599 | -3.064971 |
| H | -2.353083 | -0.835603 | -4.109588 |
| H | -3.301658 | -1.549791 | -1.902745 |
| H | -1.254209 | -1.191116 | -0.523788 |
| H | -2.616057 | -0.312513 | 0.170329 |
| H | -0.576814 | 1.130126 | 0.096442 |
| H | -3.697568 | 2.711268 | -2.433237 |
| H | -1.637920 | 3.082956 | -1.076797 |
| H | -2.827995 | 2.184366 | -0.143641 |
| H | -1.528854 | 2.776095 | -3.640023 |
| H | -2.605050 | 1.630621 | -4.421839 |
| H | 0.951900 | 2.497748 | -1.088956 |
| H | 1.981180 | 1.439345 | -3.763268 |
| H | 0.380054 | 2.929548 | -2.701022 |
| H | 4.165942 | 2.198388 | -2.953856 |
| H | 3.427988 | 2.583306 | -1.286041 |
| H | -0.334674 | 0.628193 | -4.152369 |

## 8. *References*


(1) A. Shatskiy, A. Axelsson, E. V. Stepanova, J.-Q. Liu, A. Z. Temerdashev, B. P. Kore, B. Blomkvist, J. M. Gardner, P. Dinér and M. D. Kärkäs, *Chem. Sci.*, 2021, **12**, 5430–5437. https://doi.org/10.1039/D1SC00658D

(2) J. Luo and J. Zhang, *ACS Catal.*, 2016, **6**, 873–877. https://doi.org/10.1021/acscatal.5b02204

(3) A. D. Becke, *J. Chem. Phys.*, 1993, **98**, 5648–5652. https://doi.org/10.1063/1.464913

(4) C. Lee, W. Yang and R. G. Parr, *Phys. Rev. B*, 1988, **37**, 785–789. https://doi.org/10.1103/PhysRevB.37.785

(5) R. Krishnan, J. S. Binkley, R. Seeger and J. A. Pople, *J. Chem. Phys.*, 1980, **72**, 650–654. https://doi.org/10.1063/1.438955

(6) M. J. Frisch, J. A. People and J. S. Binkley, *J. Chem. Phys.*, 1984, **80**, 3265–3269. https://doi.org/10.1063/1.447079

(7) V. Barone and M. Cossi, *J. Phys. Chem. A*, 1998, **102**, 1995–2001. https://doi.org/10.1021/jp9716997

(8) V. Barone, M. Cossi and J. Tomasi, *J. Comput. Chem.*, 1998, **19**, 404–417. https://doi.org/10.1002/(SICI)1096-987X(199803)19:4<404::AID-JCC3>3.0.CO;2-W

(9) Gaussian 16, Revision C.01, M. J. Frisch, G. W. Trucks, H. B. Schlegel, G. E. Scuseria, M. A. Robb, J. R. Cheeseman, G. Scalmani, V. Barone, G. A. Petersson, H. Nakatsuji, X. Li, M. Caricato, A. V. Marenich, J. Bloino, B. G. Janesko, R. Gomperts, B. Mennucci, H. P. Hratchian, J. V. Ortiz, A. F. Izmaylov, J. L. Sonnenberg, D. Williams-Young, F. Ding, F. Lipparini, F. Egidi, J. Goings, B. Peng, A. Petrone, T. Henderson, D. Ranasinghe, V. G. Zakrzewski, J. Gao, N. Rega, G. Zheng, W. Liang, M. Hada, M. Ehara,

K. Toyota, R. Fukuda, J. Hasegawa, M. Ishida, T. Nakajima, Y. Honda, O. Kitao, H. Nakai, T. Vreven, K. Throssell, J. A. Montgomery, Jr., J. E. Peralta, F. Ogliaro, M. J. Bearpark, J. J. Heyd, E. N. Brothers, K. N. Kudin, V. N. Staroverov, T. A. Keith, R. Kobayashi, J. Normand, K. Raghavachari, A. P. Rendell, J. C. Burant, S. S. Iyengar, J. Tomasi, M. Cossi, J. M. Millam, M. Klene, C. Adamo, R. Cammi, J. W. Ochterski, R. L. Martin, K. Morokuma, O. Farkas, J. B. Foresman and D. J. Fox, Gaussian, Inc., Wallingford CT, 2016.

(10) F. Grosjean, E. Cros-Perrial, A. Braka, J. Uttaro, L. Chaloin, L. P. Jordheim, S. Peyrottes and C. Mathé, *Eur. J. Org. Chem*., 2022, **2022**, e202101175. https://doi.org/10.1002/ejoc.202101175

(11) N. A. Weires, Y. Slutskyy and L. E. Overman, *Angew. Chem. Int. Ed*., 2019, **58**, 8561–8565. https://doi.org/10.1002/anie.201903353

(12) N. Jiang, Q. Hu, C. S. Reid, Y. Lu and C.-J. Li, *Chem. Commun*., 2003, 2318–2319. https://doi.org/10.1039/B305161G

(13) S. G. E. Amos, D. Cavalli, F. Le Vaillant and J. Waser, *Angew. Chem. Int. Ed*., 2021, **60**, 23827–23834. https://doi.org/10.1002/anie.202110257

(14) W. Zhang, Y.-C. Gu, J.-H. Lin and J.-C. Xiao, *Org. Lett*., 2020, **22**, 6642–6646. https://doi.org/10.1021/acs.orglett.0c02438

(15) K. Kiyokawa, R. Ito, K. Takemoto and S. Minakata, *Chem. Commun*., 2018, **54**, 7609–7612. https://doi.org/10.1039/C8CC03735C

(16) C. C. Nawrat, C. R. Jamison, Y. Slutskyy, D. W. C. MacMillan and L. E. Overman, *J. Am. Chem. Soc*., 2015, **137**, 11270–11273. https://doi.org/10.1021/jacs.5b07678

(17) L. Guo, F. Song, S. Zhu, H. Li and L. Chu, *Nat. Commun*., 2018, **9**, 4543. https://doi.org/10.1038/s41467-018-06904-9

(18) Y. Ye, G. Ma, K. Yao and H. Gong, *Synlett*, 2021, **32**, 1625–1628. https://doi.org/10.1055/a-1328-0352

(19) É. Vincent and J. Brioche, *Eur. J. Org. Chem*., 2021, **2021**, 2421–2430. https://doi.org/10.1002/ejoc.202100344

(20) S. P. Pitre, M. Muuronen, D. A. Fishman and L. E. Overman, *ACS Catal*., 2019, **9**, 3413–3418. https://doi.org/10.1021/acscatal.9b00405

(21) F. W. Friese and A. Studer, *Angew. Chem. Int. Ed*., 2019, **58**, 9561–9564. https://doi.org/10.1002/anie.201904028

(22) Y. Ye, H. Chen, J. L. Sessler and H. Gong, *J. Am. Chem. Soc*., 2019, **141**, 820–824. https://doi.org/10.1021/jacs.8b12801

(23) Y. Ye, H. Chen, K. Yao and H. Gong, *Org. Lett.*, 2020, **22**, 2070–2075. https://doi.org/10.1021/acs.orglett.0c00561

(24) J. L. M. Matos, S. Vásquez-Céspedes, J. Gu, T. Oguma and R. A. Shenvi, *J. Am. Chem. Soc.*, 2018, **140**, 16976–16981. https://doi.org/10.1021/jacs.8b11699
